# Dirhodium‐Catalyzed Enantioselective Synthesis of Difluoromethylated Cyclopropanes via Enyne Cycloisomerization

**DOI:** 10.1002/advs.202306404

**Published:** 2023-12-13

**Authors:** Chuntao Wang, Dong Zhu, Rui Wu, Shifa Zhu

**Affiliations:** ^1^ Key Laboratory of Functional Molecular Engineering of Guangdong Province School of Chemistry and Chemical Engineering South China University of Technology Guangzhou 510640 China; ^2^ School of Chemistry and Chemical Engineering Zhejiang Sci‐Tech University Hangzhou 310018 China; ^3^ State Key Laboratory of Elemento‐Organic Chemistry Nankai University Tianjin 300071 China

**Keywords:** carbene, cycloisomerization, difluoromethylated cyclopropane, dirhodium catalysis, enantioselective synthesis

## Abstract

(Difluoromethylated cyclopropane represents an important motif, which is widely found in bioactive and functional molecules. Despite significant progress in modern chemistry, the atom‐economic and enantioselective synthesis of difluoromethylated cyclopropanes is still challenging. Herein, an Rh_2_(II)‐catalyzed asymmetric enyne cycloisomerization is described to construct chiral difluoromethylated cyclopropane derivatives with up to 99% yield and 99% ee in low catalyst loading (0.2 mol%), which can be easily transformed into highly functionalized difluoromethylated cyclopropanes with vicinal all‐carbon quaternary stereocenters by ozonolysis. Mechanistic studies and the crystal structures of alkyne‐dirhodium complexes reveal that the cooperative weak hydrogen bondings between the substrates and the dirhodium catalyst may play key roles in this reaction.)

## Graphical abstract

A Rh_2_(II)‐catalyzed asymmetric enyne cycloisomerization to construct chiral difluoromethylated cyclopropane derivatives is reported, which can be easily transformed into highly functionalized difluoromethylated cyclopropanes with vicinal all‐carbon quaternary stereocenters through ozonolysis. Mechanistic studies and the X‐ray crystal structures of alkyne‐dirhodium complexes reveal that the cooperative weak hydrogen bondings between the substrates and the dirhodium catalyst may play key roles in this reaction.

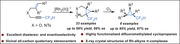

## 1 Introduction

**Scheme 1:**
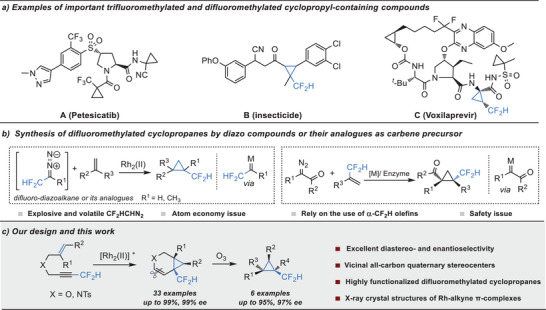
Synthetic approaches toward difluoromethylated cyclopropanes.

Fluorinated cyclopropyl motifs constitute attractive synthons in various bioactive and functional molecules as they combine the conformational rigidity of three‐membered rings with the unique and often highly beneficial feature of the fluorinated substituent.^[^ ^1^ ^]^ In particular, the trifluoromethylated^[^ ^2^ ^]^ and difluoromethylated^[^ ^3^ ^]^ cyclopropyl motifs are the most prominent, which are prevalent in a wide range of pharmaceutical and agrochemical fields, such as Petesicatib (A),^[^ ^1^, ^4^ ^]^ insecticides (B)^[^ ^1^, ^5^ ^]^ and Voxilaprevir (C)^[^ ^6^ ^]^ (**Scheme** **1**a). However, despite the advantageous effects of introducing difluoromethylated cyclopropyl in molecules, the synthetic methods of difluoromethylated cyclopropyl motifs have so far received relatively less attention than trifluoromethylated ones, with only a few methods to construct difluoromethylated cyclopropanes, especially for the asymmetric version.^[^ ^3^ ^]^ As early as 1980, Huff and Savins described the first synthesis of difluoromethylated cyclopropanes through the thermal ring contraction of difluoromethylated ^1^Δ‐pyrazolines.^[^ ^7^ ^]^ And then, piecemeal methods have been reported to synthesize achiral difluoromethylated cyclopropane,^[^ ^3^, ^8^ ^]^ such as the Corey−Chaykovsky reaction of difluoromethylated alkenes,^[^ ^9^ ^]^ 1,3‐β‐silyl‐elimination of homoallylic alcohols^[^ ^10^ ^]^ and the cyclopropanation from the β‐difluoromethyl vinylsulfonium salt,^[^ ^11^ ^]^ etc.^[^ ^8^, ^12^ ^]^ Among different methods, the cyclopropanation of carbene with alkene represents one of the most important ones to construct difluoromethylated cyclopropanes, which could be generally divided into two classes according to the types of the substrate (Scheme 1b).^[^ ^8^, ^13^ ^]^ The first one is using the difluoro‐diazoalkane or its analogs as the carbene sources to react with alkenes. However, the volatile low‐molecular‐weight difluoro‐diazoalkane severely impedes its application in organic synthesis. More importantly, so far, no asymmetric example has been reported using this method.^[^ ^14^ ^]^ As a complementary route, the cyclopropanation of difluoromethylated olefins with non‐fluorinated carbene is appealing (Scheme 1b, right). Jubault and Charette reported the first catalytic asymmetric synthesis of difluoromethylated cyclopropanes based on a dirhodium‐catalyzed cyclopropanation of difluoromethylated alkenes and α‐aryldiazoacetates or α‐nitrodiazoketone.^[^ ^15^ ^]^ Later, using diazooxindoles as carbene precursors, Ma and Zhou disclosed examples of chiral digold complex‐catalyzed cyclopropanation of α‐difluoromethyl styrenes for the synthesis of chiral difluoromethylated cyclopropanes.^[^ ^13b^ ^]^ The biocatalytic method was also applicable with ethyl diazoacetate as a carbene precursor to construct chiral difluoromethyl‐containing trisubstituted cyclopropanes as reported by Fasan et. al.^[^ ^13a^ ^]^ Despite all the above achievements, the development of an efficient, atom‐economical, and safe method for the asymmetric synthesis of difluoromethylated cyclopropane is still highly desirable.

In the past decades, the transition metal‐catalyzed enyne cycloisomerization reaction has evolved as a powerful and convenient strategy for the rapid assembly of carbocyclic and heterocyclic compounds from relatively simple acyclic substrates.^[^ ^16^ ^]^ In particular, this strategy has been widely used in the construction of cyclopropane‐fused polycyclic skeletons in an economical manner,^[^ ^16^, ^17^ ^]^ which is distinguished from traditional diazo‐based strategy.^[^ ^18^ ^]^ However, to the best of our knowledge, there is no report on constructing chiral difluoromethylated cyclopropanes using this method. Until recently, we developed a Rh_2_(II)‐catalyzed asymmetric cycloisomerization reactions of benzo‐fused CF_2_H‐substituted enynes,^[^ ^19^ ^]^ in which the dirhodium‐carbene‐involved cascaded cyclopropanations occurred, giving the enantioenriched CF_2_H‐substituted biscyclopropane products. Inspired by the above results and our previous studies on alkyne‐involved carbene chemistry,^[^ ^20^ ^]^ we envisioned that if a tethered rather than benzo‐fused difluoromethyl‐substituted enyne was used as substrate, it might undergo the cascaded enyne cycloisomerization and [1,2]‐H shift to give difluoromethylated vinylcyclopropane, which can be easily further transformed into highly functionalized difluoromethylated cyclopropane via the oxidative cleavage of the alkenyl (Scheme 1c). Here we describe the realization of such an Rh_2_(II)‐catalyzed intramolecular cycloisomerization of CF_2_H‐substituted enynes followed by ozonolysis, allowing the efficient and practical synthesis of a range of tetrahydropyridine‐ or dihydropyran‐fused difluoromethylated cyclopropanes and highly functionalized difluoromethylated cyclopropyl motifs with vicinal all‐carbon quaternary stereocenters in good yields and excellent stereoselectivities. Moreover, X‐ray crystal structures of dirhodium‐alkyne π‐complexes were obtained successfully to elucidate the possible reaction mechanism.

## 2 Results and Discussion

The investigation began with TsN‐tethered enyne **1a** as the model substrate. As shown in **Table** **1**, under the catalysis of 1 mol% Rh_2_(OPiv)_4_ at room temperature, the enyne cycloisomerization of **1a** occurred smoothly to give the difluoromethylated cyclopropane **2a** in 62% yield (entry 1). Then, a systematic examination of various chiral dirhodium(II) catalysts was conducted (entries 2−9). The desired product **2a** could be obtained in 95% yield and 43% ee when dirhodium *N*‐sulfonylprolinate Rh_2_(*S*‐DOSP)_4_ was used (entry 2). When sterically more crowded Rh_2_(*S*‐NTTL)_4_ was tested, large reduction in yield (10%) and moderate enantioselectivity (55% ee) were observed (entry 3). Subsequently, the phthalimide based dirhodium(II) complexes, such as Rh_2_(*S*‐PTPA)_4_, Rh_2_(*S*‐PTAD)_4_, Rh_2_(*S*‐PTTL)_4_, Rh_2_(*S*‐TCPTTL)_4_ and Rh_2_(*S*‐TFPTTL)_4_ were screened (entries 4−8). Among them, electron‐deficient dirhodium catalysts Rh_2_(*S*‐TCPTTL)_4_ and Rh_2_(*S*‐TFPTTL)_4_ gave excellent yields (85%, 93%) but moderate ees (75%, 60%) (entries 7–8). Pleasingly, *D_2_*‐symmetric dirhodium triarylcyclopropane carboxylate catalysts Rh_2_(*S*‐BTPCP)_4_ developed by Davies et. al.^[^ ^16^, ^21^ ^]^ gave the highest enantioselectivity of 99% ee (entry 9). Furthermore, the catalyst loading could be reduced to 0.2 mol% with 92% yield and no erosion of ee value (entry 11). However, the reaction efficiency dropped sharply with 0.1 mol% of catalyst loading (entry 12).

**Table 1:** Optimization of the reaction conditions.

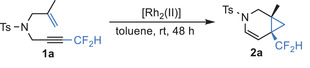			
Entrya)	[Rh2(II)] (%)	Yielld%b)	ee%c)
1	Rh_2_(OPiv)_4_ (1)	62	‐
2	Rh_2_(*S*‐DOSP)_4_ (1)	95	43
3	Rh_2_(*S*‐NTTL)_4_ (1)	10	55
4	Rh_2_(*S*‐PTPA)_4_ (1)	94	5
5	Rh_2_(*S*‐PTAD)_4_ (1)	31	29
6	Rh_2_(*S*‐PTTL)_4_ (1)	26	0
7	Rh_2_(*S*‐TCPTTL)_4_ (1)	85	75
8	Rh_2_(*S*‐TFPTTL)_4_ (1)	93	60
9	Rh_2_(*S*‐BTPCP)_4_ (1)	90	99
10	Rh_2_(*S*‐BTPCP)_4_ (0.5)	95^d)^	99
**11**	**Rh_2_(*S*‐BTPCP)_4_ (0.2)**	**92** ^d)^	**99**
12	Rh_2_(*S*‐BTPCP)4 (0.1)	5	‐
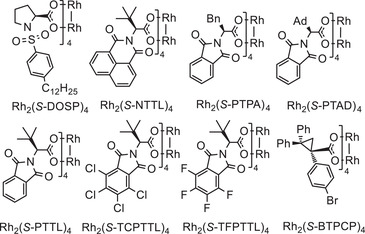			

^a)^ The reaction was conducted with **1a** (0.05 mmol) in toluene (0.5 mL), RT, under N_2_, 48 h;

^b)^ NMR yield;

^c)^ The ee was determined by chiral HPLC;

^d)^ Isolated yield.

**Scheme 2:**
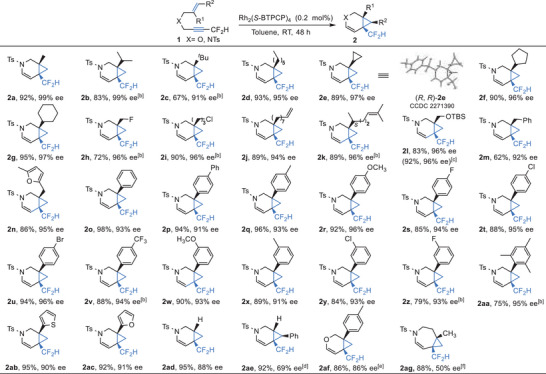
Scope of the asymmetric cyclopropanation. Reaction conditions: ^[a]^Rh_2_(*S*‐BTPCP)_4_ (0.2 mol%), Toluene (0.5 mL), **1** (0.05 mmol), RT, 48 h, Isolated yield; ^[b]^Rh_2_(*S*‐BTPCP)_4_ (1 mol%); ^[c]^ **1l**: 1.1 g, 15 mL Toluene, 72 h; ^[d]^ *(E)*−**1ae** was used, Rh_2_(*S*‐TCPTTL)_4_ (1 mol%). ^[e]^Rh_2_(*S*‐TCPTTL)_4_ (1 mol%); ^[f]^Rh_2_(*S*‐BTPCP)_4_ (2 mol%), 100 °C.

With the optimized reaction condition (Table 1, entry 11) in hand, the substrate scope was then examined. As shown in **Scheme** **2**, the catalytic system could be successfully applied to a variety of CF_2_H‐substituted enynes **1**. The CF_2_H‐substituted enyne derivatives with different substituents on the C═C double bond could be used as effective substrates. Enynes with alkyl substituents at 2‐position of the C═C double bond gave the desired products **2a**–**2n** in high yields (62–95%) and excellent ees (91–99%). The absolute configuration of (*R*, *R*)−**2e** was determined by the X‐ray crystallographic analysis. It is worth mentioning that alkyl substituents bearing rich functional groups, such as halide (**1h**, **1i**), alkenyl (**1j**, **1k**), TBS‐protected hydroxymethyl (**1l**), and aryl (**1m**, **1n**) groups, all worked well and furnished the corresponding difluoromethylated cyclopropane products (72‐89%, 92–96% ees). This cycloisomerization reaction of **1l** could also be easily scaled up to gram scale without the loss of the yield and enantioselectivity (92%, 96% ee). In addition, the substrates with aryl group at 2‐position of the C═C double bond furnished the desired products **2p**–**2ac** in high yields (75–98%) and excellent ees (90–96%). Both electron‐rich and ‐deficient aryl groups have little impact on yield and enantioselectivity. Notably, heterocyclic substituents like thienyl and furyl were proved compatible, affording products **2ab** (95%, 90% ee) and **2ac** (92%, 91% ee). Moreover, the enyne **1ad** with monosubstituted alkenyl was also feasible to give product **2ad** in 95% yield and 88% ee. When the substrate with a phenyl group at 1‐position of the C═C double bond, product **2ae** was isolated in good yield and moderate ee (92%, 69% ee). Delightfully, the ether‐tether enyne was also an effective substrate, the desired difluoromethylated cyclopropane‐fused dihydropyran (**2af**) could be obtained in 86% yield and 86% ee by changing the catalyst to Rh_2_(*S*‐TCPTTL)_4_. Furthermore, this catalytic reaction could be extended to 1,7‐enyne, giving the desired cyclopropane‐fused tetrahydroazepine **2ag** (88%, 50% ee).

**Scheme 3:**
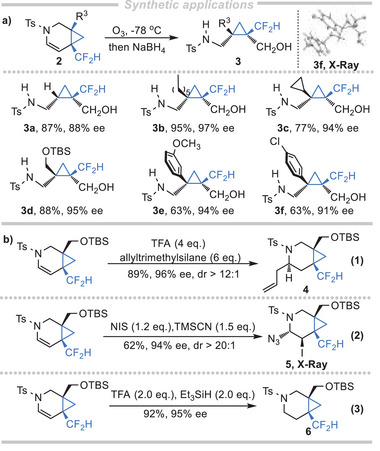
Synthetic applications of products.

With the establishment of an efficient route to synthesize the enantioenriched difluoromethylated fused‐cyclopropane products **2**, we were eager to know whether a collection of simple difluoromethylated cyclopropanes could be synthesized to meet the needs of diverse synthesis. With this in mind, we then searched for the subsequent ring‐opening conditions to transform **2** into highly functionalized difluoromethylated cyclopropanes **3**. Gratifyingly, as shown in **Scheme** **3**, the electron‐rich C═C double bond of the bicyclic compounds **2** could be selectively cleaved through ozonolysis followed by in situ reduction with NaBH_4_ to give highly functionalized CF_2_H‐substituted cyclopropanes containing vicinal all‐carbon quaternary stereocenters. It is worth mentioning that the functional groups of **3**, such as amide and hydroxy groups, could serve as useful handles for further manipulations. Then, the versatility of this reaction was tested and the representative examples were listed in Scheme 3a. The variations of R^3^ (H, alkyl, aryl) group in product **2** were investigated, which led to the desired CF_2_H‐substituted cyclopropane product **3** in moderate to good yields with retention of chiral integrity. It is worth mentioning that this kind of obtained highly‐functionalized chiral difluoromethylated cyclopropanes, especially with vicinal all‐carbon quaternary stereocenters, were difficult to synthesize with the previous methods.^[^ ^8^, ^13^, ^15^ ^]^ Additionally, the C═C double bond of **2l** could undergo allylation by treatment with allyltrimethylsilane in the presence of trifluoroacetic acid, giving the allylation product **4** in 89% yield and 96% ee. Interestingly, the reaction of **2l** with NIS and TMSN_3_ in an ice bath overnight generated the difunctionalized product **5** in 62% yield and 94% ee. Finally, the C═C double bond in **2l** could also be reduced to give cyclopropane‐fused piperidine **6** (92%, 95% ee) under the reduction of Et_3_SiH.

**Scheme 4:**
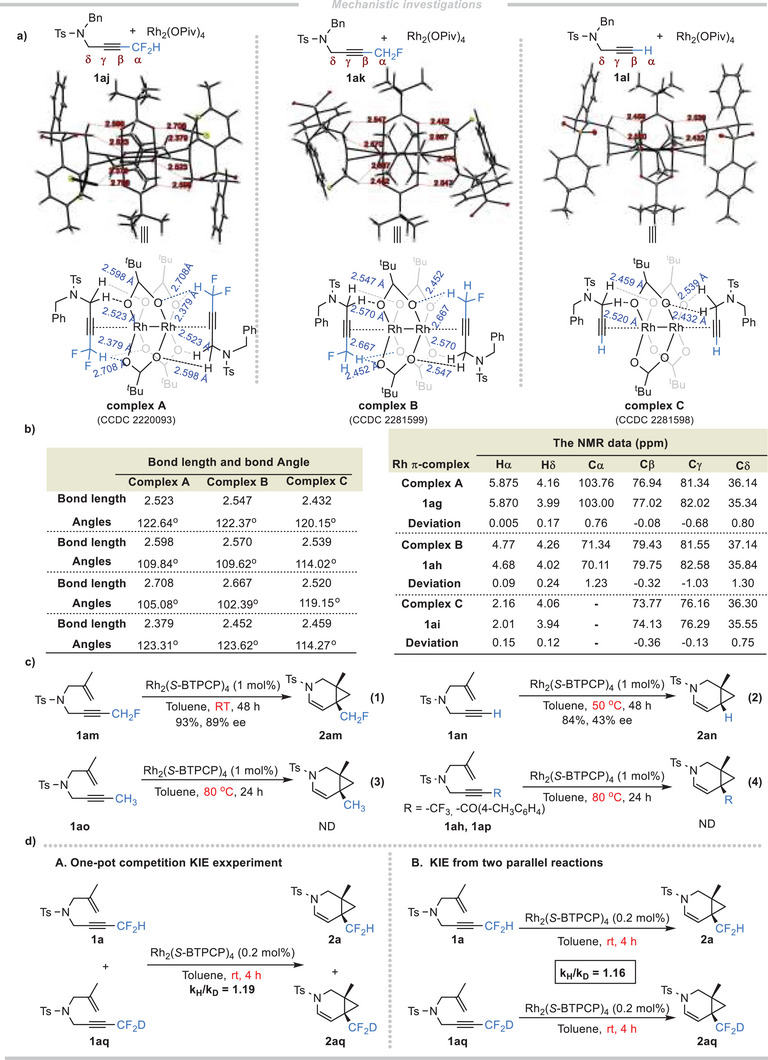
Mechanistic investigations.

To gain further insights into this asymmetric cycloisomerization reaction, three stable Rh_2_(OPiv)_4_ π‐complexes **A**‐**C** were obtained as shown in **Scheme** **4**a.^[^ ^19^ ^]^ Scrutiny of the crystal data of these alkyne‐dirhodium(II) π‐complexes show that a molecule of Rh_2_(II) catalyst was coordinated axially with two alkyne molecules. In complex **A** and complex **B**, the coordinated alkynes point toward the opposite directions, while in complex **C** toward the same directions. Moreover, there are close contacts between the carboxylate oxygen of the catalyst and the substrates **1aj**‐**1al**, indicating cooperative weak hydrogen bondings (C−H…O < 2.72 Å) between the substrates (NCH_2_/CF_2_H/CFH_2_) and the carboxyl ligands of the Rh_2_(II) catalyst (Scheme 4a), which could partially explain why the dirhodium(II) as weak alkynophilic catalyst can activate the carbon‐carbon triple bond. As shown in Scheme 4b, the bond angles related to the hydrogen bondings range from 102.39^o^–123.62^o^ (bond lengths range from 2.379–2.708 Å), indicating that these are weak hydrogen bondings.^[^ ^22^ ^]^ These unique weak hydrogen bondings might also be the reason for the successful isolation of these weakly coordinated complexes. In addition, Rh_2_(OPiv)_4_ π‐complexes **A**‐**C** were fully characterized by NMR spectroscopy. There are slightly downfield shifts for the atoms on terminal groups (CF_2_H, CFH_2_, H) and δ‐position of alkyne‐Rh complexes in the ^1^H NMR and ^13^C NMR spectra. Importantly, the ^13^C signals of the alkynes (β, γ position) in complexes **A**‐**C** were shifted to a higher frequency because of the feedback effect of the rhodium.^[^ ^23^ ^]^ These NMR data support the existence of weak hydrogen bondings and coordination interactions between the dirhodium catalysts and the alkyne substrates **1aj**‐**1al** in solution. As a result, the enynes **1am** and **1an** which correspond to **1ak** and **1al** were subjected to the reaction, giving the target products **2am** (93%, 89% ee) and **2an** (84%, 43% ee), although an elevated temperature was required for the formation of **2an** (Scheme 4c). These experimental results are consistent with the crystallographic and spectral data. However, when replacing the capping group of the C≡C with the neutral ‐CH_3_ group or the electron‐deficient ‐CF_3,_ ‐COAr groups, no desired products were observed even at elevated temperature, which could be attributed to the increasing steric bulk. In addition, **1a** and the deuterated counterparts **1aq** were subjected to the one‐pot competitive kinetic isotope effect (KIE) experiment and the parallel KIE experiment, the k_H_/k_D_ are 1.19 and 1.16, respectively. That's to say, when ‐CF_2_H was replaced by ‐CF_2_D, a slower reaction rate was observed because of the weaker hydrogen bondings between the substrate and the dirhodium catalyst (Scheme 4d). These results also suggested the key roles of weak hydrogen bondings in our catalytic system.^[^ ^24^ ^]^

## 3 Conclusion

In summary, we have developed an efficient method for the synthesis of chiral difluoromethylated cyclopropanes via dirhodium‐catalyzed cycloisomerization of 1, 6‐enynes. Moreover, a series of highly functionalized difluoromethylated cyclopropanes with vicinal all‐carbon quaternary stereocenters could be obtained through the subsequent ozonolysis process. This protocol features high atom economy, low catalyst loading, good functional group tolerance, and excellent enantioselectivities. Moreover, the cooperative weak hydrogen bondings between the substrates and the dirhodium catalyst were found in single crystal structures of alkyne‐dirhodium complexes and NMR experiments. These cooperative weak hydrogen bondings possibly account for the efficient activation of alkyne by a weak alkynophilic dirhodium catalyst, which was consistent with the control experiments. We believe that this method is a significant and valuable alternative in the preparation of enantiomerically enriched difluoromethylated cyclopropane derivatives.[Supplementary-material advs7028-supitem-0001]

## Conflict of Interest

The authors declare no conflict of interest.

## Supporting information

Supporting Information

## Data Availability

The data that support the findings of this study are available in the supplementary material of this article.
