# Supplementary material for: Dirhodium‐Catalyzed Enantioselective Synthesis of Difluoromethylated Cyclopropanes via Enyne Cycloisomerization
Source: Adv Sci (Weinh). 2023 Dec 13;11(7):2306404. doi: 10.1002/advs.202306404 (PMC10870034; doi:10.1002/advs.202306404)
Supplement: Supplementary file 1 — Supporting Information [file ADVS-11-2306404-s001.pdf]

ADVANCED SCIENCE
Open Access

Supporting Information

for *Adv. Sci.*, DOI 10.1002/advs.202306404

Dirhodium-Catalyzed Enantioselective Synthesis of Difluoromethylated Cyclopropanes via Enyne Cycloisomerization

Chuntao Wang, Dong Zhu, Rui Wu and Shifa Zhu**

Supporting Information

Dirhodium-catalyzed enantioselective synthesis of difluoromethylated cyclopropanes *via* enyne cycloisomerization

Chuntao Wang, Dong Zhu, Rui Wu * and Shifa Zhu *

Contents

1. General information 3

2. Preparation of 1, 6-enyne 4

3. General procedure for Rh-catalyzed enyne cycloisomerization 31

4. The derivatizations of difluoromethylated cyclopropane compounds 63

5. The NOE of compound **4** 75

6. The X-ray diffraction analysis 76

7. The NMR spectra of compounds **1aj-al** and complexes **A-C**. 82

8. The NMR spectra of compounds **1-6** 91

9. References 220

1. General information

All reactions were carried out under an inert atmosphere of dry N_2 in Schlenk tube. Tetrahydrofuran and toluene were distilled from sodium and benzophenone prior to use. Dichloromethane and dichloroethane were distilled from CaH_2 prior to use. ^{1}H, ^{13}C, ^{19}F NMR spectra were recorded on Bruker AVANCE 400 MHz, 500 MHz or 600 MHz, ^{1}H NMR and ^{13}C NMR chemical shifts were determined relative to internal standard TMS at δ 0.0 and ^{19}F NMR chemical shifts were determined relative to $CFCl_3$ as external standard. Chemical shifts (δ) are reported in ppm, and coupling constants (*J*) are in Hertz (Hz). The following abbreviations were used to explain the multiplicities: s = singlet, d = doublet, t = triplet, q = quartet, m = multiplet, br = broad. Optical rotations were measured on ADP440+B+S. HRMS (EI) and HRMS (ESI) were determined on Waters Micromass GCT Premier, Agilent Technologies 6224 TOF LC/MS, and APEX III 7.0 TESLA FTMS spectrometers, respectively. All other reagents and solvents were used as received from commercial sources, unless specified otherwise, or prepared as described in the literature. The spectral data of the known compounds were in consistence with data reported in the literature. Melting points were determined using a hot stage apparatus. Specific rotation [a] was determined using a polarimeter ZhuoGuang GP30. Diastereotopic ratios were determined by ^{1}H NMR. Enantiomeric ratios were determined by HPLC, using a Daicel OD-H and OJH or Phenomenex INA and INC or Regis (R, R) Whelk-O1 column with n-hexane and *i*-PrOH as eluent.

2. Preparation of 1, 6-enyne[1]

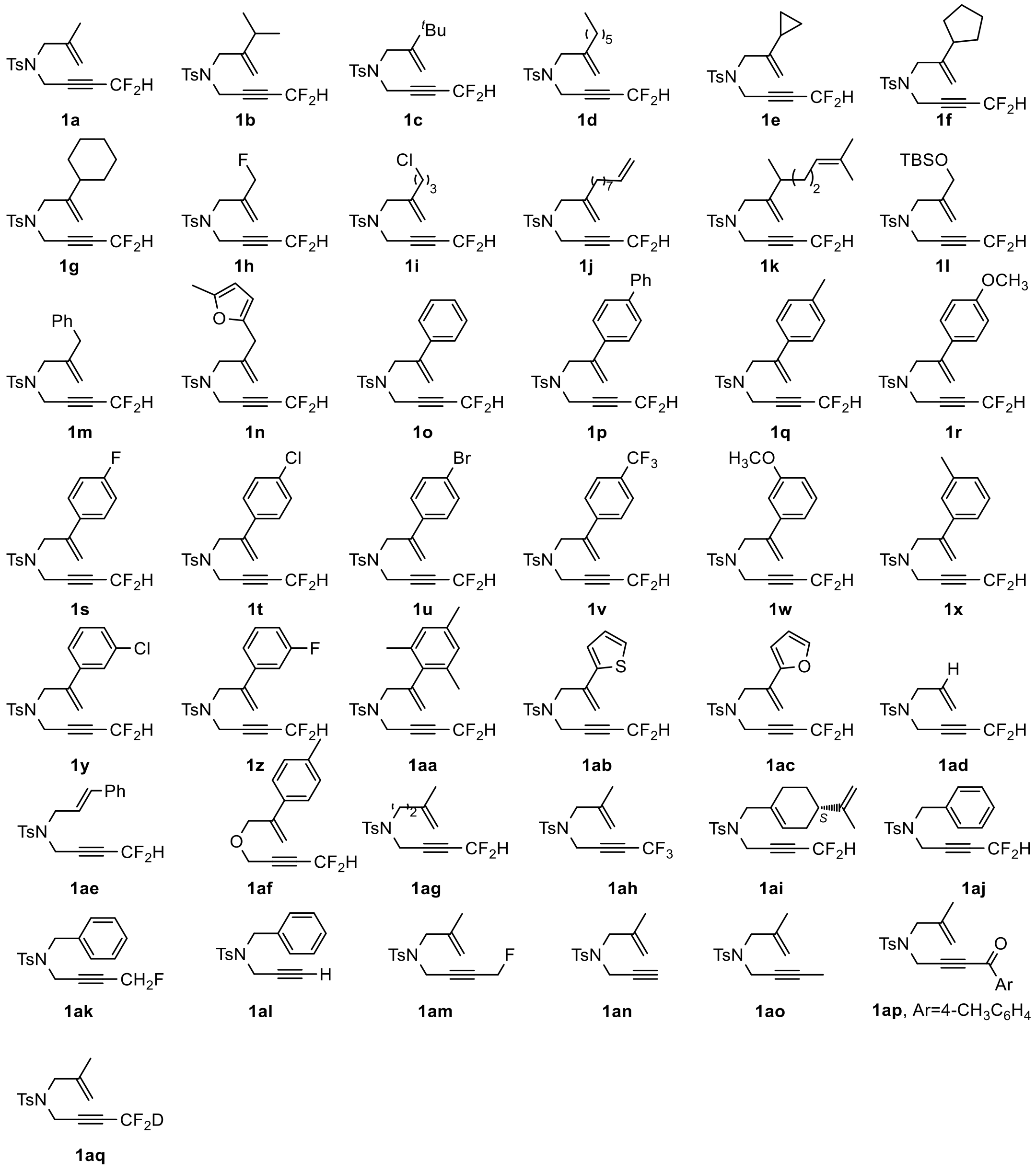

General Procedure A:

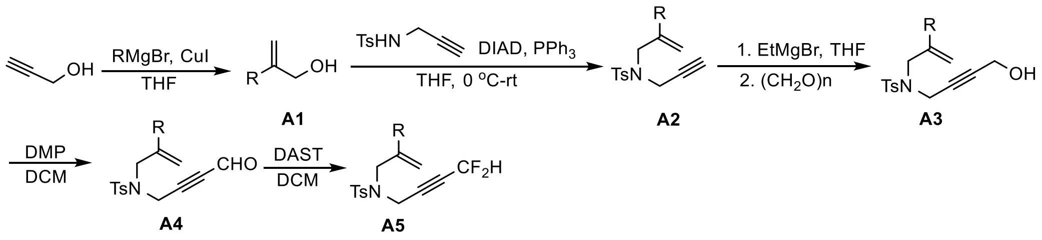

To a dried flask was added CuI (1.8 mmol, 15 mol%) and RMgBr (0. 5 M, 29.3 mmol, 2.5 equiv.) at room temperature, the mixture was then allowed to stir for 30 min. Propargyl alcohol (11.7 mmol) in THF (10 mL) was added dropwise at room temperature. The reaction mixture was then heated to reflux for 24 h. After cooling to room temperature, saturated aqueous NH_4Cl was added dropwise carefully. The organic phase was separated and the aqueous phase was extracted with EtOAc. The combined organic

phases were dried over anhydrous Na_2SO_4 and the solvent was removed under reduced pressure. The obtained crude product was purified by flash column chromatography (PE: EtOAc = 3/1) to give allylic alcohol **A1**.

To a suspension of 4-methyl-N-(prop-2-yn-1-yl)benzenesulfonamide (1.1 equiv.), PPh_3 (1.3 equiv.) and allylic alcohol (1.0 equiv. in THF (0.25 M) was added DIAD (1.3 equiv.) dropwise at 0 °C under N_2, the mixture was stirred at room temperature until alcohol was completely consumed. The mixture was transferred to flask and evaporated under reduced pressure. The residue was purified by silica gel column chromatography (PE: EtOAc = 20:1) to give enyne **A2**.

The obtained enyne **A2** (4.0 mmol, 1.0 equiv.) was dissolved in THF (15 mL), and EtMgBr (6 mmol, 1.5 equiv., 1 M in THF) was slowly added into the corresponding solution at 0 °C under N_2. After 1 h, the $(CH_2O)_n$ (20.0 mmol, 5.0 equiv.) was then added. The resulting mixture was stirred overnight at room temperature and was then quenched with saturated aqueous NH_4Cl. The mixture was extracted with EtOAc. Combined organic layers were washed with brine, dried over $MgSO_4$, and evaporated. Crude products were purified by column chromatography (PE: EtOAc = 2: 1) to give **A3**

The DMP (1.2 equiv.) was added into the solution of alkynol **A3** in DCM. The reaction was monitored by TLC until disappearance of the starting material. The reaction mixture was quenched with saturated aqueous $Na_2S_2O_3$ and $NaHCO_3$. The mixture was extracted with DCM. Combined organic layers were washed with brine, dried over $MgSO_4$, and evaporated. Crude products were purified by column chromatography (PE: EtOAc = 5: 1) to give alkynal **A4**.

To a solution of alkynal **A4** (1.0 equiv.) in anhydrous DCM was added DAST (1.25 equiv.) dropwise under nitrogen at -10°C, and the reaction mixture was stirred at this temperature and monitored by TLC until the alcohol was completely consumed. The reaction mixture was quenched with saturated aqueous $NaHCO_3$. The resulting mixture was extracted with DCM for three times. The combined organic phase was washed with brine, dried over Na_2SO_4 and evaporated under reduced pressure. The residue was purified by silica gel column chromatography (PE: EtOAc = 10:1) to give the final product **A5**.

The substrates such as **1e-1g, 1o-1t, 1v-1z, 1aa ,1ag and 1ai** were synthesized according to **General procedure A.**

General procedure B:

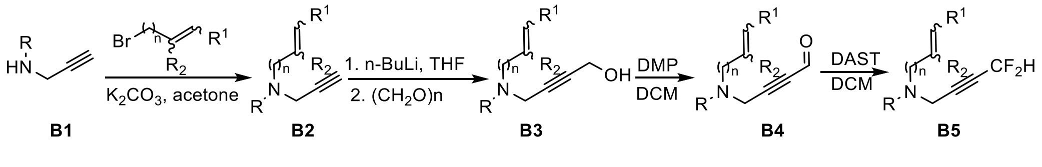

To the solution of **B1** (5.0 mmol, 1.0 equiv.) in acetone (20 mL), K_2CO_3 (25.0 mmol, 5.0 equiv.) and 3-bromoprop-1-ene (6.0 mmol, 1.2 equiv.) was added and stirred at 80 °C under N_2. After 12 h, the reaction mixture was extracted with EtOAc and water. Combined organic layers were washed with brine, dried over $MgSO_4$, and evaporated. Crude products were purified by column chromatography to give **B2** (PE: EtOAc = 5:1).

The obtained **B2** (4.0 mmol, 1.0 equiv.) was dissolved in THF (15 mL), and EtMgBr(6 mmol, 1.5 equiv., 1 M in THF) was slowly added into the corresponding solution at -78 °C under N_2. After 1 h, the $(CH_2O)_n$ (20.0 mmol, 5.0 equiv.) was then added. The resulting mixture was stirred overnight under room

temperature and was then quenched with saturated aqueous NH_4Cl. The mixture was extracted with EtOAc. Combined organic layers were washed with brine, dried over $MgSO_4$, and evaporated. Crude products were purified by column chromatography (PE: EtOAc = 2:1). The DMP (1.2 equiv.) was added into the solution of **B3** in DCM at 0 °C. The reaction was monitored by TLC until disappearance of the starting material. The reaction mixture was quenched with saturated aqueous $Na_2S_2O_3$ and $NaHCO_3$. The mixture was extracted with DCM. Combined organic layers were washed with brine, dried over $MgSO_4$, and evaporated. Crude products were purified by column chromatography (PE: EtOAc= 5:1) to give alkynal **B4**.

To a solution of alkynal **B4** (1.0 equiv.) in anhydrous DCM was added DAST (1.25 equiv.) dropwise under nitrogen at -10°C, and the reaction mixture was stirred at this temperature and monitored by TLC until the alcohol was completely consumed. The reaction mixture was quenched with saturated aqueous $NaHCO_3$. The resulting mixture was extracted with DCM for three times. The combined organic phase was washed with brine, dried over Na_2SO_4 and evaporated under reduced pressure. The residue was purified by silica gel column chromatography (PE: EtOAc = 10:1) to give **B5**.

The substrates such as **1a**, **1ad-1ae 1aj-1al** and **1ao** were synthesized according to **General procedure B**.

General procedure C:

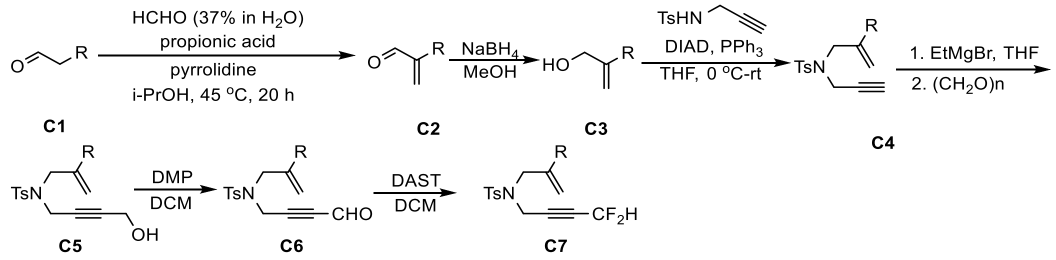

To a solution of formaldehyde (20 mmol, 37% in water, 1.0 equiv.) and **C1**(20 mmol, 100 mol %, 1.0 equiv.) in *i*-PrOH (20 mL) was added propionic acid (2 mmol, 10 mol%) and pyrrolidine (2 mmol, 10 mol%). The reaction mixture was stirred at 45 °C for 20 hours. Saturated aqueous $NaHCO_3$ (6 mL) was then added and the mixture was extracted with DCM for three times. The combined organic extracts were washed with brine, dried (Na_2SO_4), and concentrated in vacuo. Purification of the residue by flash chromatography (PE: EtOAc = 30:1) afforded the corresponding aldehyde **C2**.

The $NaBH_4$ (1.2 equiv.) was added into the solution of **C2** (10 mmol, 1.0 equiv.) in MeOH (10 mL) at 0 °C. The reaction was monitored by TLC until disappearance of the starting material. The reaction mixture was quenched with 10% HCl solution. The mixture was extracted with DCM. Combined organic layers were washed with brine, dried over $MgSO_4$, and evaporated. Crude products were used directly for the next step without further purification.

The subsequent procedures were operated according to **General procedure A**.

The substrates such as **1b-1d**, **1i-1k** and **1m-1n** were synthesized according to **General procedure C.**

General Procedure D:

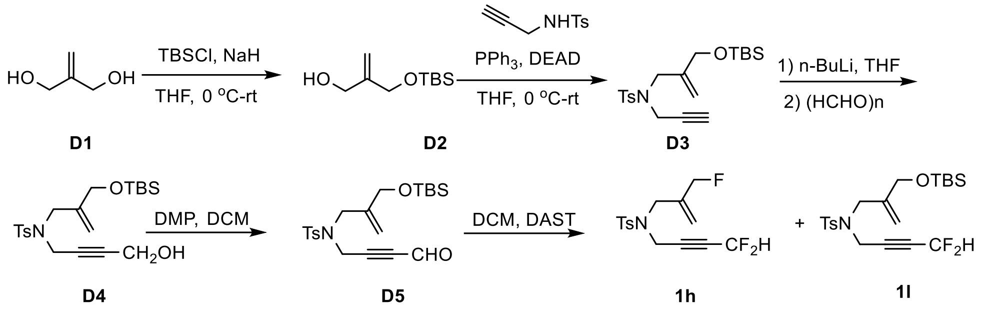

To a dried flask was added 50 mL dry THF under N_2, followed by addition of NaH (33 mmol, 1.1 equiv.). To this solution was added 2-methylenepropane-1,3-diol (2.64 g, 1.0 equiv.) dropwise at 0 °C. The mixture was then brought to room temperature and stirred for 45 min. TBSCl (4.98 g, 1.1 equiv.) was added in one portion and the mixture was stirred overnight. The reaction was quenched with saturated aqueous NH_4Cl and extracted with EtOAc for three times. The organic layer was washed with brine, dried over Na_2SO_4, filtered and evaporated under reduced pressure. The residue was purified by silica gel column chromatography (PE: EtOAc = 4:1) to give the **D2** as clear oil, 3.2 g, 52% yield.

To a suspension of 4-methyl-N-(prop-2-yn-1-yl)benzenesulfonamide (6.9 g, 1,1 equiv.), PPh_3 (10.3 g, 1.3 equiv.) and **D2** (6.0 g, 1.0 equiv.) in THF was added DEAD (6,79 g, 1.3 equiv.) dropwise at 0 °C under N_2, the mixture was stirred at room temperature until **D2** was completely consumed. The mixture was transferred to flask and evaporated under reduced pressure. The residue was purified by silica gel column chromatography (PE: EtOAc = 20:1) to give **D3** as white solid, 64% yield.

The obtained **D3** (1.0 equiv.) was dissolved in THF, and n-BuLi (1.2 equiv., 2.5 M in THF) was slowly added into the corresponding solution at -78 °C under N_2. After 1 h, the $(CH_2O)_n$ (3.0 equiv.) was then added in one portion. The resulting mixture was stirred overnight at room temperature and was then quenched with saturated aqueous NH_4Cl. The mixture was extracted with EtOAc for three times. Combined organic layers were washed with brine, dried over $MgSO_4$, and evaporated. The crude products were purified by column chromatography (PE: EtOAc = 2:1) to give 7.9 g **D4** as yellow oil, 98% yield.

The DMP (1.2 equiv.) was added into the solution of **D4** in DCM under air. The reaction was monitored by TLC until disappearance of the starting material. The reaction mixture was quenched with saturated aqueous $Na_2S_2O_3$ and $NaHCO_3$. The mixture was extracted with DCM. Combined organic layers were washed with brine, dried over $MgSO_4$, and evaporated. Crude products were purified by flash chromatography (PE: EtOAc = 5: 1) to give yellow liquid **D5** in 87% yield.

To a solution of alkynal **D5** (1.0 equiv.) in anhydrous DCM was added DAST (1.25 equiv.) dropwise under nitrogen at -10°C, and the reaction mixture was stirred at this temperature and monitored by TLC until the alcohol was completely consumed. The reaction mixture was quenched with saturated aqueous $NaHCO_3$. The resulting mixture was extracted with DCM for three times. The combined organic phase was washed with brine, dried over Na_2SO_4, evaporated under reduced pressure. The residue was purified by silica gel column chromatography (PE: EtOAc = 10: 1) to give **1h** (35%) **and 1l** (46%).

General Procedure E

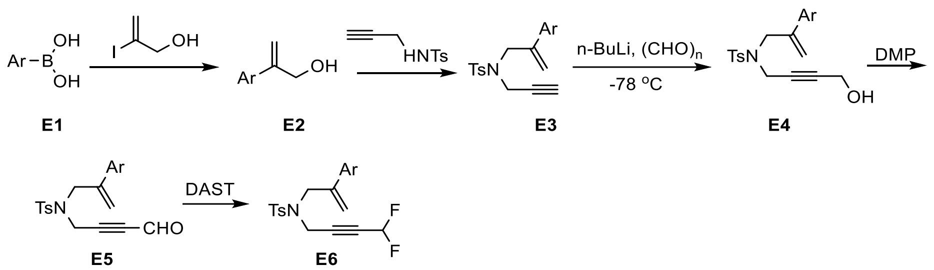

Stir the mixture of the arylboronic acid **E1** (5.0 mmol, 1.0 equiv.), 2-iodoprop-2- en-1-ol (6.0 mmol, 1.2 equiv.), $Pd(PPh_3)_4$ (0.025 mmol, 5 mol%) and Cs_2CO_3 (10.0 mmol, 2.0 equiv.) in 20 mL EtOH under nitrogen. The reaction mixture was stirred at 70 °C for 12 hours. Then, remove EtOH under vacuum and extract the mixture with EtOAc and H_2O. The combined organic phase was washed with brine, dried over Na_2SO_4, filtered and evaporated under reduced pressure. The residue was purified by silica gel column chromatography (PE: EtOAc = 15: 1) to give **E2.**

The subsequent procedures were operated according to **General procedure A**.

The substrates such as **1u** and **1ab-1ac** were synthesized according to **General procedure E.**

Procedure F:

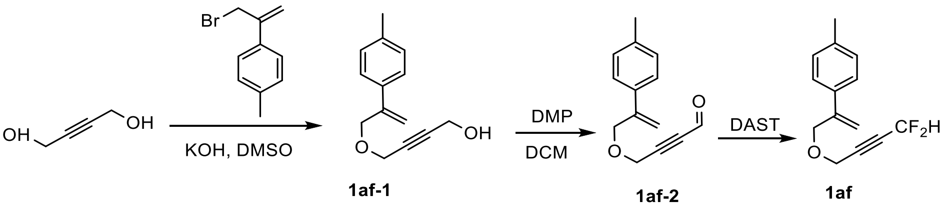

To a suspension of KOH (2.24 g, 40 mmol, 5.0 equiv.) in DMSO (20 mL) were added 3-bromo-2-methylprop-1-ene (1.07g, 8 mmol, 1.0 equiv.) and 2-Butyne-1,4-diol (3.44g, 40 mmol, 5.0 equiv.). The mixture was then stirred for 2 h, poured into water and extracted with DCM. The aqueous phase was then acidified with aqueous HCl (6 M) and further extracted with DCM. The combined organic phases were washed with water, dried with $MgSO_4$, and concentrated in vacuo. Crude products were purified by column chromatography (PE: EtOAc = 3: 1).

The subsequent procedures were operated according to **General procedure A**.

Procedure G:

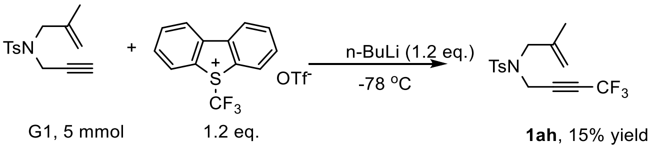

Under N_2 atmosphere, the solution of n-BuLi (2.4 mL, 6 mmol, 2.5 M in hexane) was added dropwise into the solution of **G1** (1.3 g, 5 mmol) in anhydrous THF (20 mL) at -78 °C. After 1 h, *S* -(trifluoromethyl)-dibenzothiophenium (1.2 eq.) dissolved in THF (5 mL) was added. The mixture was allowed to stirred at room temperature overnight. The reaction was quenched with water and extracted with EtOAc. The organic layer was dried with anhydrous Na_2SO_4. The solvent was removed under reduced pressure. The resulting residue was purified by neutral alumina column chromatography using

PE/EA = 10:1 as eluent, 0.24g, 12%.

Procedure H:

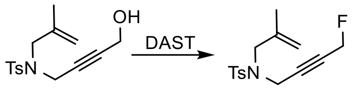

1ai

To a solution of alcohol (1.0 equiv.) in anhydrous DCM was added DAST (1.25 equiv.) dropwise under nitrogen at -10 °C, and the reaction mixture was stirred at this temperature and monitored by TLC until the alcohol was completely consumed. Water was added and the resulting mixture was extracted with DCM for three times. The combined organic phase was washed with brine, dried over Na_2SO_4, filtered and evaporated under reduced pressure. The residue was purified by silica gel column chromatography (PE: EtOAc = 5: 1) to give **1ai** as yellow oil.

6N-(4,4-difluorobut-2-yn-1-yl)-4-methyl-N-(2-methylallyl)benzenesulfonamide (1a)

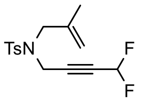

Colorless oil, 68% yield, Rf = 0.2 (PE: EtOAc = 15: 1). **^{1}H NMR** (500 MHz, $CDCl_3$) δ 7.67 – 7.63 (m, 2H), 7.24 (d, J = 8.4 Hz, 2H), 5.90 – 5.66 (m, 1H), 4.93 – 4.87 (m, 2H), 4.04 (td, J = 4.8, 1.2 Hz, 2H), 3.64 (s, 2H), 2.35 (s, 3H), 1.69 (s, 3H). **^{13}C NMR** (126 MHz, $CDCl_3$) δ 144.10, 138.84, 135.43, 129.65, 127.66, 116.06, 102.96 (t, J = 232.6 Hz), 82.07 (t, J = 7.4 Hz), 76.69 (t, J = 34.0 Hz), 52.98, 35.19, 21.45, 19.58. **^{19}F NMR** (471 MHz, $CDCl_3$) δ -106.57. **HRMS**: Calculation for $C_{15}H_{17}F_2NO_2S$, $[M+Na]^+$, 336.0840, Found: 336.0846.

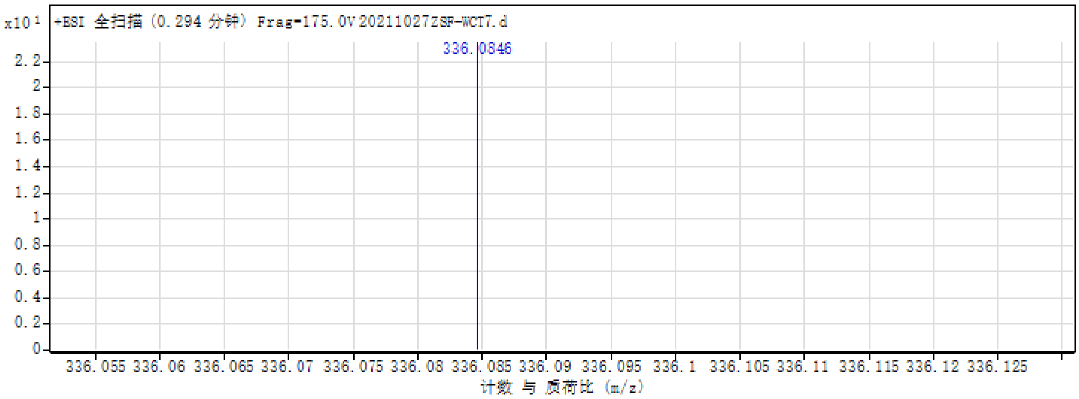

N-(4,4-difluorobut-2-yn-1-yl)-4-methyl-N-(3-methyl-2-methylenebutyl)benzenesulfonamide (1b)

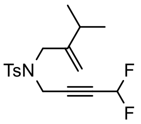

Colorless oil, 70% yield, Rf = 0.2 (PE: EtOAc = 15: 1). **^{1}H NMR** (500 MHz, Chloroform-*d*) δ 7.73 (d, J = 7.9 Hz, 2H), 7.32 (d, J = 8.0 Hz, 2H), 5.84 (s, 1H), 5.02 (s, 1H), 4.98 (s, 1H), 4.10 (t, J = 4.9 Hz, 2H), 3.78 (s, 2H), 2.42 (s, 3H), 2.36 (hept, J = 7.7, 6.9 Hz, 1H), 1.08 (d, J = 6.9 Hz, 6H). **^{13}C NMR** (126 MHz, $CDCl_3$) δ 148.73, 144.12, 135.32, 129.66, 127.69, 112.95, 102.95 (t, J = 232.6 Hz), 82.08 (t, J = 7.3 Hz), 76.81 (t, J = 32.1, 31.2 Hz), 50.95, 35.14, 30.39, 21.49, 21.46. **^{19}F NMR** (471 MHz, $CDCl_3$) δ -106.52. **HRMS**: Calculation for $C_{17}H_{21}F_2NO_2S$, $[M+H]^+$, 342.1334, Found: 342.1333.

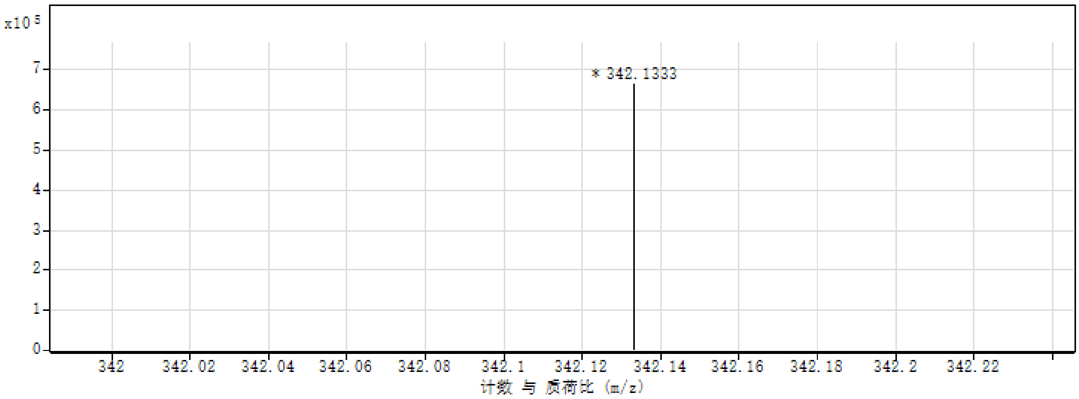

N-(4,4-difluorobut-2-yn-1-yl)-N-(3,3-dimethyl-2-methylenebutyl)-4-methylbenzenesulfonamide (1c)

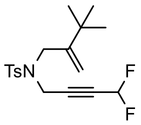

Colorless oil, 66% yield, Rf = 0.2 (PE: EtOAc = 15: 1). **^{1}H NMR** (500 MHz, Chloroform-*d*) δ 7.66 (d, *J* = 8.1 Hz, 2H), 7.25 (d, *J* = 8.0 Hz, 2H), 5.76 (t, *J* = 54.6 Hz, 1H), 5.04 (s, 1H), 4.97 (s, 1H), 4.08 (t, *J* = 4.9 Hz, 2H), 3.77 (s, 2H), 2.35 (s, 3H), 1.04 (s, 9H). **^{13}C NMR** (126 MHz, $CDCl_3$) δ 149.89, 144.09, 135.41, 129.67, 127.68, 111.81, 102.93 (t, *J* = 232.9 Hz), 82.27 (t, *J* = 7.1 Hz), 76.99 (t, *J* = 35.0 Hz), 48.29, 35.44, 35.11, 29.15, 21.46. **^{19}F NMR** (471 MHz, $CDCl_3$) δ -106.54. **HRMS**: Calculation for $C_{18}H_{23}F_2NO_2S$, $[M+H]^+$, 356.1490, Found: 356.1488.

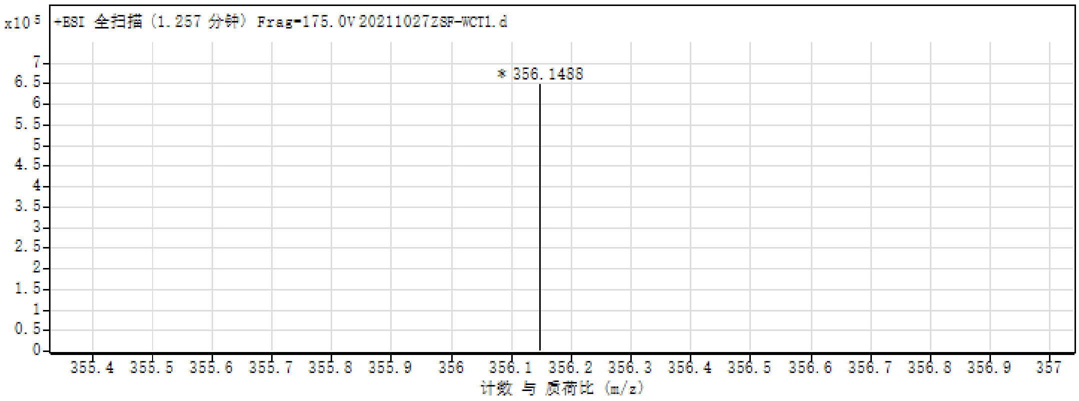

N-(4,4-difluorobut-2-yn-1-yl)-4-methyl-N-(2-methyleneoctyl)benzenesulfonamide (1d)

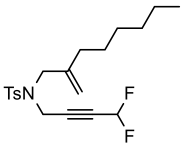

Colorless solid, 82% yield, m.p. = 50.1-50.3 °C, Rf = 0.2 (PE: EtOAc = 20: 1). **^{1}H NMR** (500 MHz, Chloroform-*d*) δ 7.72 (d, *J* = 7.9 Hz, 2H), 7.32 (d, *J* = 7.9 Hz, 2H), 5.85 (t, *J* = 54.7 Hz, 1H), 4.99 (s, 2H), 4.11 (t, *J* = 4.8 Hz, 2H), 3.73 (s, 2H), 2.42 (s, 3H), 2.04 (t, *J* = 7.7 Hz, 2H), 1.45 (q, *J* = 7.3 Hz, 2H), 1.33 – 1.25 (m, 6H), 0.89 (t, *J* = 6.6 Hz, 3H). **^{13}C NMR** (126 MHz, $CDCl_3$) δ 144.10, 142.94, 135.38, 129.65, 127.69, 115.06, 103.88 (t, *J* = 232.6 Hz), 82.08 (t, *J* = 7.0 Hz), 76.74 (t, *J* = 34.4 Hz), 51.68, 35.16, 32.88, 31.66, 28.97, 27.31, 22.61, 21.45, 14.06. **^{19}F NMR** (471 MHz, $CDCl_3$) δ -106.51. **HRMS**: Calculation for $C_{20}H_{27}F_2NO_2S$, $[M+H]^+$, 384.1803, Found: 384.1802.

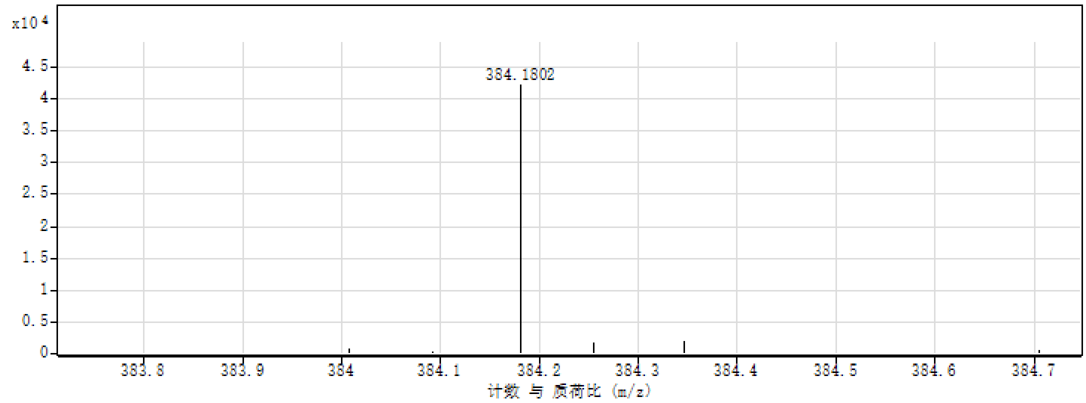

N-(2-cyclopropylallyl)-N-(4,4-difluorobut-2-yn-1-yl)-4-methylbenzenesulfonamide (1e)

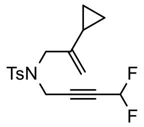

Colorless oil, 55% yield, Rf = 0.2 (PE: EtOAc = 15: 1). ^{1}**H NMR** (500 MHz, Chloroform-*d*) δ 7.74 (d, *J* = 8.3 Hz, 2H), 7.32 (d, *J* = 8.0 Hz, 2H), 5.85 (t, *J* = 54.7 Hz, 1H), 4.86 (s, 1H), 4.80 (s, 1H), 4.16 (t, *J* = 4.6 Hz, 2H), 3.81 (s, 2H), 2.43 (s, 3H), 1.38 (dd, *J* = 9.3, 4.1 Hz, 1H), 0.79 – 0.64 (m, 2H), 0.51 (dt, *J* = 5.3, 3.0 Hz, 2H). ^{13}**C NMR** (126 MHz, $CDCl_3$) δ 144.42, 144.09, 135.40, 129.64, 127.68, 111.23, 102.96 (t, *J* = 232.9 Hz), 82.15 (t, *J* = 7.1 Hz), 76.91 (t, *J* = 36.0 Hz), 52.25, 35.25, 21.47, 13.44, 7.09. ^{19}**F NMR** (471 MHz, $CDCl_3$) δ -106.52. **HRMS**: Calculation for $C_{17}H_{19}F_2NO_2S$, $[M+H]^+$, 340.1177, Found: 340.1171.

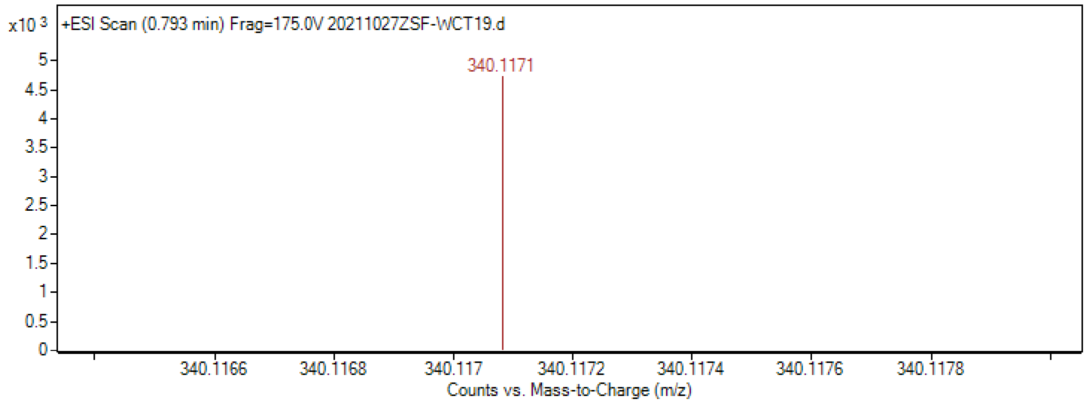

N-(2-cyclopentylallyl)-N-(4,4-difluorobut-2-yn-1-yl)-4-methylbenzenesulfonamide (1f)

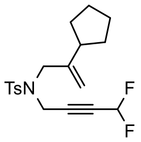

Colorless oil, 75% yield, Rf = 0.2 (PE: EtOAc = 20: 1). ^{1}**H NMR** (500 MHz, Chloroform-*d*) δ 7.72 (d, *J* = 8.1 Hz, 2H), 7.32 (d, *J* = 8.0 Hz, 2H), 5.84 (t, *J* = 54.7 Hz, 1H), 5.03 (s, 1H), 4.97 (s, 1H), 4.12 (t, *J* = 4.6 Hz, 2H), 3.77 (s, 2H), 2.42 (s, 4H), 1.95 – 1.85 (m, 2H), 1.74 – 1.64 (m, 2H), 1.64 – 1.51 (m, 2H), 1.43 – 1.30 (m, 2H). ^{13}**C NMR** (126 MHz, $CDCl_3$) δ 146.22, 144.07, 135.36, 129.63, 127.69, 112.80, 102.95 (t, *J* = 232.9 Hz), 82.12 (t, *J* = 7.2 Hz), 76.94 (t, *J* = 32.2 Hz), 51.98, 42.80, 35.20, 31.47, 24.81, 21.45. ^{19}**F NMR** (471 MHz, $CDCl_3$) δ -106.51. **HRMS**: Calculation for $C_{19}H_{23}F_2NO_2S$, $[M+H]^+$, 390.1310, Found: 390.1303.

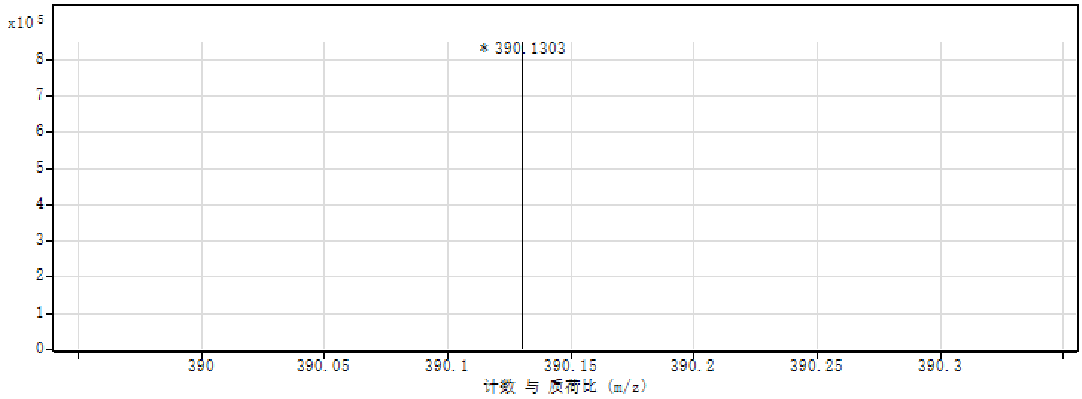

N-(2-cyclohexylallyl)-N-(4,4-difluorobut-2-yn-1-yl)-4-methylbenzenesulfonamide (1g)

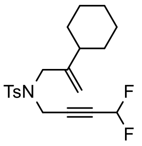

Colorless oil, 80% yield, Rf = 0.2 (PE: EtOAc = 20: 1). **^{1}H NMR** (500 MHz, Chloroform-*d*) δ 7.72 (d, *J* = 8.2 Hz, 2H), 7.32 (d, *J* = 8.0 Hz, 2H), 5.85 (t, *J* = 54.6 Hz, 1H), 4.98 (d, *J* = 5.2 Hz, 2H), 4.10 (t, *J* = 4.5 Hz, 2H), 3.76 (s, 2H), 2.42 (s, 3H), 1.95 (t, *J* = 11.5 Hz, 1H), 1.78 (t, *J* = 14.8 Hz, 4H), 1.68 (d, *J* = 12.4 Hz, 1H), 1.28 (q, *J* = 12.8 Hz, 2H), 1.15 (q, *J* = 12.1 Hz, 3H). **^{13}C NMR** (126 MHz, $CDCl_3$) δ 147.93, 144.08, 135.36, 129.63, 127.68, 113.44, 102.98 (t, *J* = 232.7 Hz), 82.17 (t, *J* = 7.2 Hz), 76.78 (t, *J* = 34.5 Hz), 51.01, 40.48, 35.14, 32.17, 26.57, 26.24, 21.42. **^{19}F NMR** (471 MHz, $CDCl_3$) δ -106.52. **HRMS**: Calculation for $C_{20}H_{25}F_2NO_2S$, $[M+H]^+$, 382.1647, Found: 382.1647.

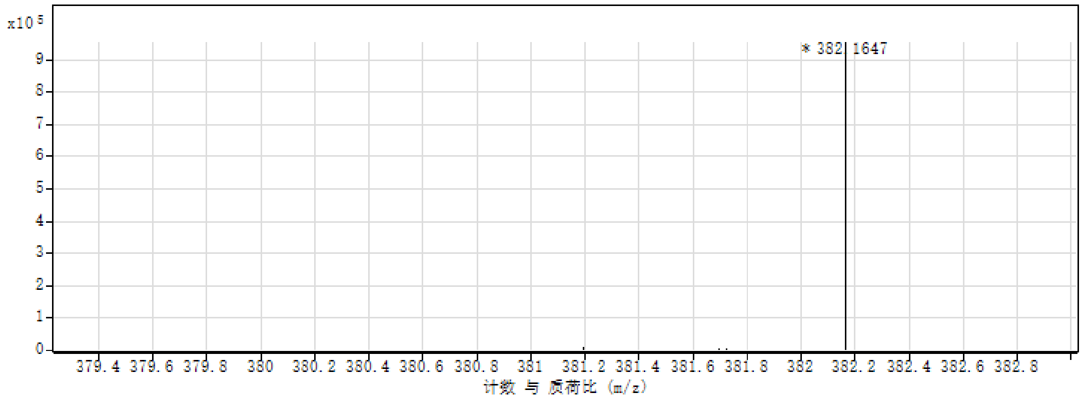

N-(4,4-difluorobut-2-yn-1-yl)-N-(2-(fluoromethyl)allyl)-4-methylbenzenesulfonamide (1h)

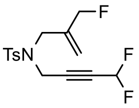

Colorless oil, 35% yield, Rf = 0.2 (PE: EtOAc = 10: 1). **^{1}H NMR** (500 MHz, Chloroform-*d*) δ 7.65 (d, *J* = 7.9 Hz, 2H), 7.26 (d, *J* = 8.0 Hz, 2H), 5.80 (t, *J* = 54.5 Hz, 1H), 5.32 (s, 1H), 5.22 (s, 1H), 4.84 (s, 1H), 4.75 (s, 1H), 4.08 (s, 2H), 3.77 (s, 2H), 2.35 (s, 3H). **^{13}C NMR** (126 MHz, $CDCl_3$) δ 144.43, 138.16 (d, *J* = 14.8 Hz), 135.04, 129.78, 127.68, 118.42 (d, *J* = 9.4 Hz), 102.94 (t, *J* = 233.0 Hz), 82.73 (d, *J* = 168.2 Hz), 81.76 (t, *J* = 7.3 Hz), 77.02 (t, *J* = 34.5 Hz), 48.64 (d, *J* = 2.7 Hz), 35.64 (t, *J* = 2.6 Hz), 21.47. **^{19}F NMR** (471 MHz, $CDCl_3$) δ -106.69, -218.41.

HRMS: Calculation for $C_{15}H_{16}F_3NO_2S$, $[M+Na]^+$, 354.0746, Found: 354.0744.

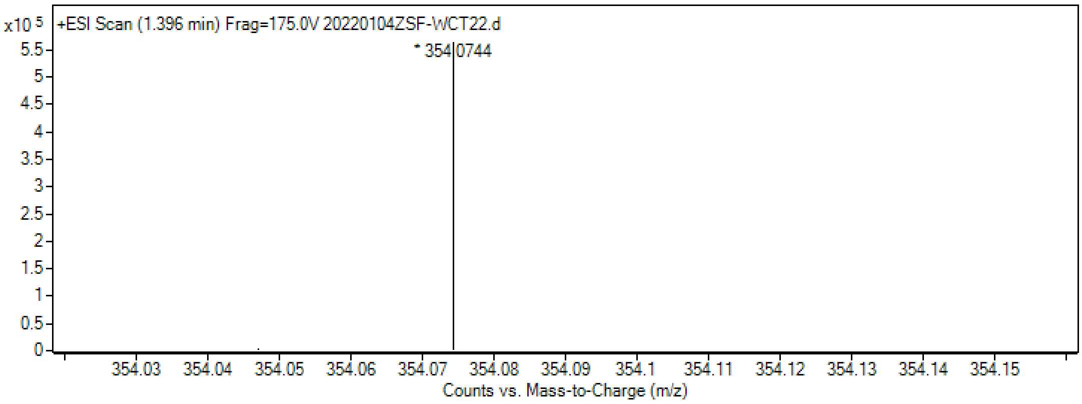

N-(5-chloro-2-methylenepentyl)-N-(4,4-difluorobut-2-yn-1-yl)-4-methylbenzenesulfonamide (1i)

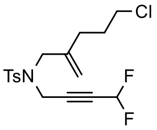

Colorless oil, 57% yield, Rf = 0.2 (PE: EtOAc = 10: 1). **^{1}H NMR** (500 MHz, Chloroform-*d*) δ 7.72 (d, *J* = 7.9 Hz, 2H), 7.33 (d, *J* = 7.9 Hz, 2H), 5.86 (t, *J* = 54.6 Hz, 1H), 5.06 (d, *J* = 2.9 Hz, 2H), 4.11 (t, *J* = 4.8 Hz, 2H), 3.74 (s, 2H), 3.54 (t, *J* = 6.5 Hz, 2H), 2.43 (s, 3H), 2.23 (t, *J* = 7.6 Hz, 2H), 2.00 – 1.91 (m, 2H). **^{13}C NMR** (126 MHz, $CDCl_3$) δ 144.28, 141.22, 135.21, 129.73, 127.67, 116.33, 102.02 (t, *J* = 233.2 Hz), 81.88 (t, *J* = 7.2 Hz), 76.87 (t, *J* = 34.4 Hz), 51.53, 44.30, 35.26, 30.08, 29.92, 21.48. **^{19}F NMR** (471 MHz, $CDCl_3$) δ -106.56. **HRMS**: Calculation for $C_{17}H_{20}ClF_2NO_2S$, $[M+H]^+$, 376.0944, Found: 376.0941.

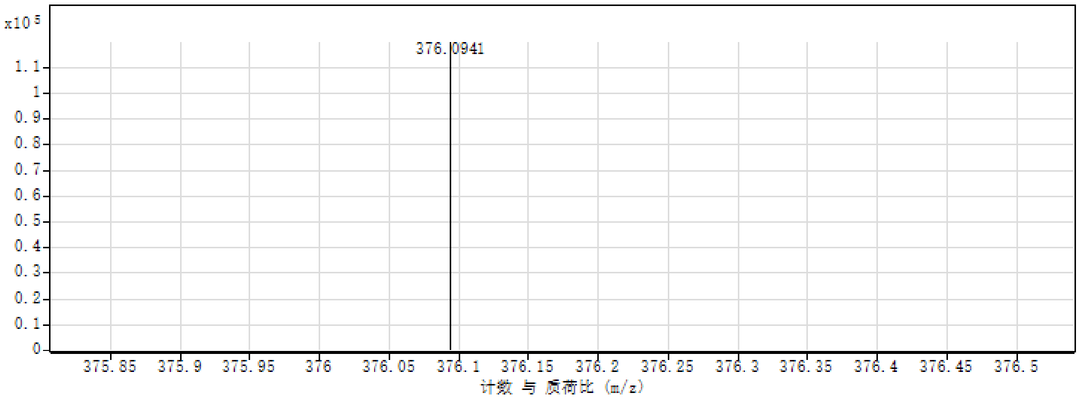

N-(4,4-difluorobut-2-yn-1-yl)-N-(4,4,4,4,4,4-hexamethyl-2-methylene-4l9-pent-4-en-1-yl)-4-methylbenzenesulfonamide (1j)

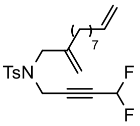

Colorless oil, 45% yield, Rf = 0.2 (PE: EtOAc = 10: 1). **^{1}H NMR** (500 MHz, Chloroform-*d*) δ 7.65 (d, *J* = 8.0 Hz, 2H), 7.24 (d, *J* = 8.0 Hz, 2H), 5.95 – 5.32 (m, 2H), 5.08 – 4.58 (m, 4H), 4.03 (t, *J* = 4.8 Hz, 2H), 3.65 (s, 2H), 2.35 (s, 3H), 1.97 (t, *J* = 7.4 Hz, 4H), 1.38 (t, *J* = 7.4 Hz, 2H), 1.34 – 1.27 (m, 2H), 1.22 (d, *J* = 6.8 Hz, 6H). **^{13}C NMR** (126 MHz, $CDCl_3$) δ 144.10, 142.91, 139.21, 135.37, 129.65, 127.70, 115.10, 114.16, 102.02 (t, *J* = 233.3 Hz), 82.07 (t, *J* = 7.3 Hz), 76.91 (t,

J = 38.3 Hz), 51.68, 35.16, 33.79, 32.86, 29.29, 29.24, 29.07, 28.90, 27.32, 21.47. **^{19}F NMR** (471 MHz, $CDCl_3$) δ -106.50. **HRMS**: Calculation for $C_{23}H_{31}F_2NO_2S$, $[M+H]^+$, 424.2116, Found: 424.2114.

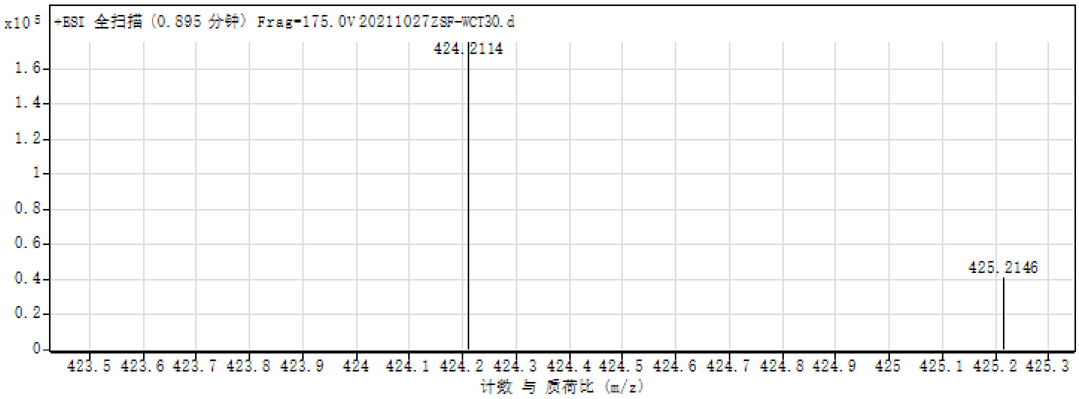

(*S*)-N-(4,4-difluorobut-2-yn-1-yl)-N-(3,7-dimethyl-2-methyleneoct-6-en-1-yl)-4-methylbenzenesulfonamide (2k)

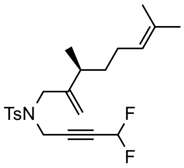

Colorless oil, 47% yield, Rf = 0.2 (PE: EtOAc = 10: 1). **^{1}H NMR** (500 MHz, Chloroform-*d*) δ 7.73 (d, J = 7.9 Hz, 2H), 7.32 (d, J = 8.0 Hz, 2H), 5.84 (t, J = 54.6 Hz, 1H), 5.10 (t, J = 7.3 Hz, 1H), 5.02 (d, J = 6.8 Hz, 2H), 4.23 – 4.04 (m, 2H), 3.76 (d, J = 3.7 Hz, 2H), 2.42 (s, 3H), 2.24 (q, J = 6.9 Hz, 1H), 1.95 (t, J = 7.0 Hz, 2H), 1.69 (s, 3H), 1.60 (s, 3H), 1.51 (dq, J = 8.5, 6.6 Hz, 1H), 1.44 – 1.31 (m, 1H), 1.06 (d, J = 6.9 Hz, 3H). **^{13}C NMR** (126 MHz, $CDCl_3$) δ 147.47, 144.10, 135.41, 131.62, 129.65, 127.69, 124.30, 113.59, 102.95 (t, J = 232.9 Hz), 82.08 (t, J = 7.3 Hz), 76.83 (t, J = 32.8 Hz), 50.72, 35.93, 35.46, 35.21, 35.19, 25.71 (d, J = 7.4 Hz), 21.46, 19.65, 17.67. **^{19}F NMR** (471 MHz, $CDCl_3$) δ -106.51. **HRMS**: Calculation for C22H29F2NO2S, $[M+Na]^+$, 432.1780, Found: 432.1779.

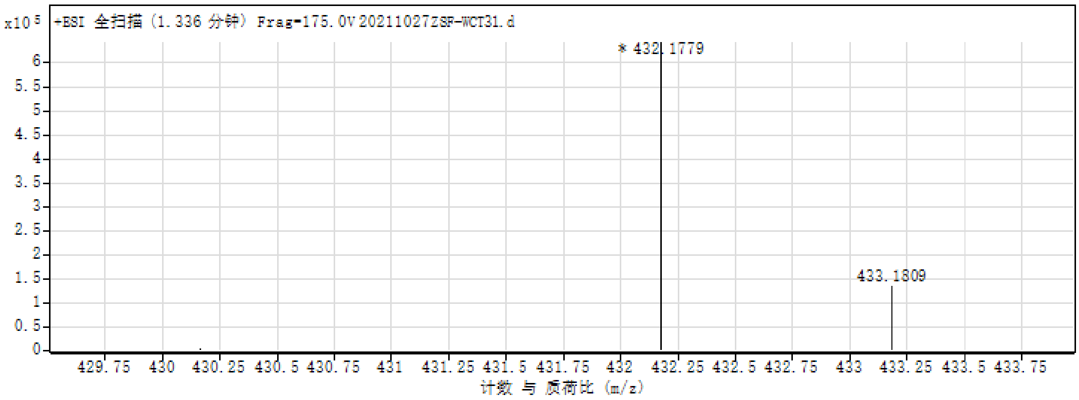

N-(2-(((tert-butyldimethylsilyl)oxy)methyl)allyl)-N-(4,4-difluorobut-2-yn-1-yl)-4-methylbenzenesulfonamide (2l)

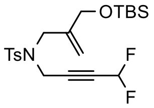

Colorless oil, 46% yield, Rf = 0.2 (PE: EtOAc = 10: 1). **^{1}H NMR** (500 MHz, Chloroform-*d*) δ 7.64 (d, *J* = 8.0 Hz, 2H), 7.24 (d, *J* = 8.0 Hz, 2H), 5.78 (t, *J* = 54.6 Hz, 1H), 5.25 (s, 1H), 5.03 (s, 1H), 4.06 (d, *J* = 7.5 Hz, 4H), 3.71 (s, 2H), 2.34 (s, 3H), 0.84 (s, 9H), -0.00 (s, 6H). **^{13}C NMR** (126 MHz, $CDCl_3$) δ 144.19, 141.82, 135.21, 129.68, 127.71, 114.76, 102.94 (t, *J* = 232.7 Hz), 82.00 (t, *J* = 7.3 Hz), 76.87 (t, *J* = 34.2 Hz), 63.48, 49.14, 35.39, 25.85, 21.46, 18.30, -5.47. **^{19}F NMR** (471 MHz, $CDCl_3$) δ -106.56. **HRMS**: Calculation for $C_{21}H_{31}F_2NO_3SSi$, $[M+Na]^+$, 466.1654, Found: 466.1653.

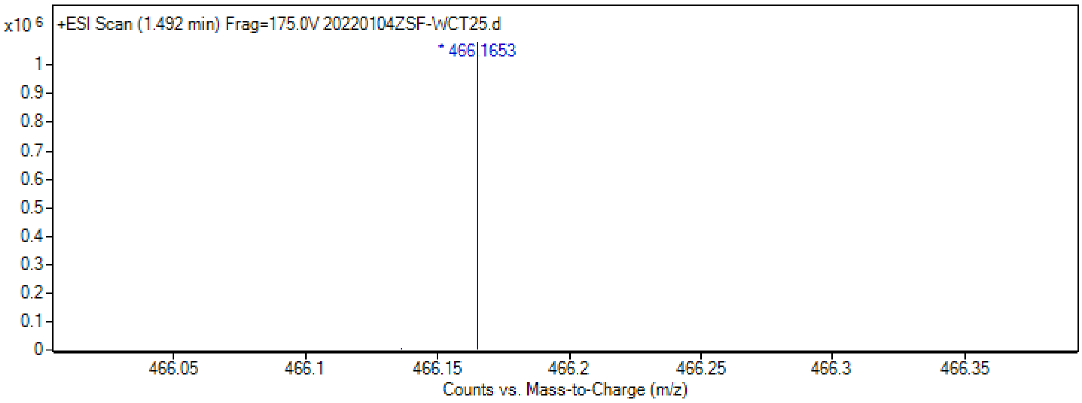

N-(2-benzylallyl)-N-(4,4-difluorobut-2-yn-1-yl)-4-methylbenzenesulfonamide (1m)

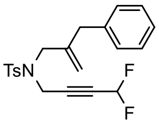

Colorless oil, 76% yield, Rf = 0.2 (PE: EtOAc = 10: 1). **^{1}H NMR** (500 MHz, Chloroform-*d*) δ 7.68 (d, *J* = 7.9 Hz, 2H), 7.29 (d, *J* = 7.0 Hz, 4H), 7.23 – 7.21 (m, 3H), 5.81 (t, *J* = 54.6 Hz, 1H), 5.08 (s, 1H), 5.01 (s, 1H), 4.09 (s, 2H), 3.70 (s, 2H), 3.39 (s, 2H), 2.41 (s, 3H). **^{13}C NMR** (126 MHz, $CDCl_3$) δ 144.18, 142.40, 138.48, 135.22, 129.67, 129.21, 128.48, 127.72, 126.46, 117.09, 102.00 (t, *J* = 232.6, 0.0 Hz), 81.95 (t, *J* = 7.0 Hz), 76.57 (t, *J* = 34.4 Hz), 51.27, 39.76, 35.39, 21.49. **^{19}F NMR** (471 MHz, $CDCl_3$) δ -106.53. **HRMS**: Calculation for $C_{21}H_{21}F_2NO_2S$, $[M+H]^+$, 390.1334, Found: 390.1330.

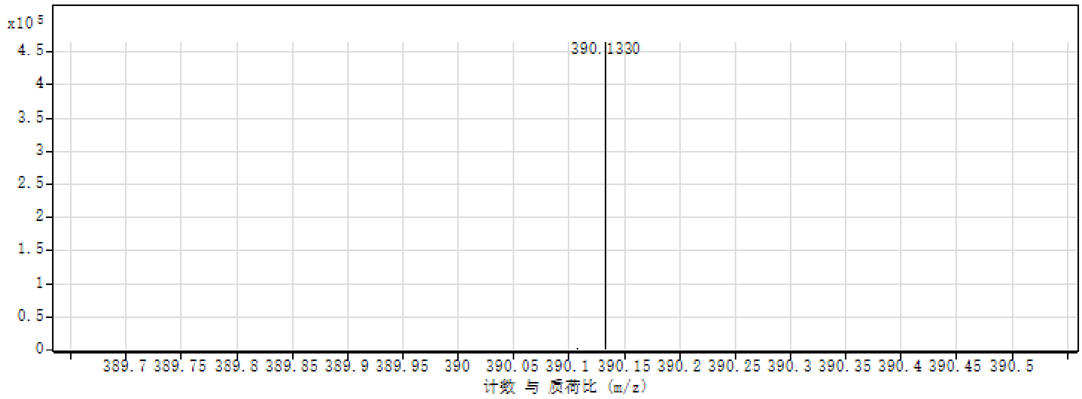

N-(4,4-difluorobut-2-yn-1-yl)-4-methyl-N-(2-((5-methylfuran-2-yl)methyl)allyl)benzenesulfonamide (1n)

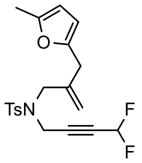

Colorless solid, 50% yield, m.p. = 71.4 - 71.6 °C, Rf = 0.2 (PE: EtOAc = 10: 1). **^{1}H NMR** (500 MHz, Chloroform-*d*) δ 7.71 (d, *J* = 8.2 Hz, 2H), 7.30 (d, *J* = 8.1 Hz, 2H), 5.99 (d, *J* = 2.8 Hz, 1H), 5.96 – 5.68 (m, 2H), 5.14 – 5.03 (m, 2H), 4.14 – 4.04 (m, 2H), 3.76 (d, *J* = 12.3 Hz, 2H), 3.35 (s, 2H), 2.41 (s, 3H), 2.24 (s, 3H). **^{13}C NMR** (126 MHz, $CDCl_3$) δ 151.13, 150.21, 144.17, 139.64, 135.27, 129.68, 127.72, 117.37, 107.82, 106.17, 102.00 (t, *J* = 233.0 Hz), 81.98 (t, *J* = 7.3 Hz), 76.92 (t, *J* = 32.0 Hz), 51.25, 35.36, 32.14, 21.51, 13.55. **^{19}F NMR** (471 MHz, $CDCl_3$) δ -106.55. **HRMS**: Calculation for $C_{20}H_{21}F_2NO_3S$, $[M+Na]^+$, 416.1102, Found: 416.1102.

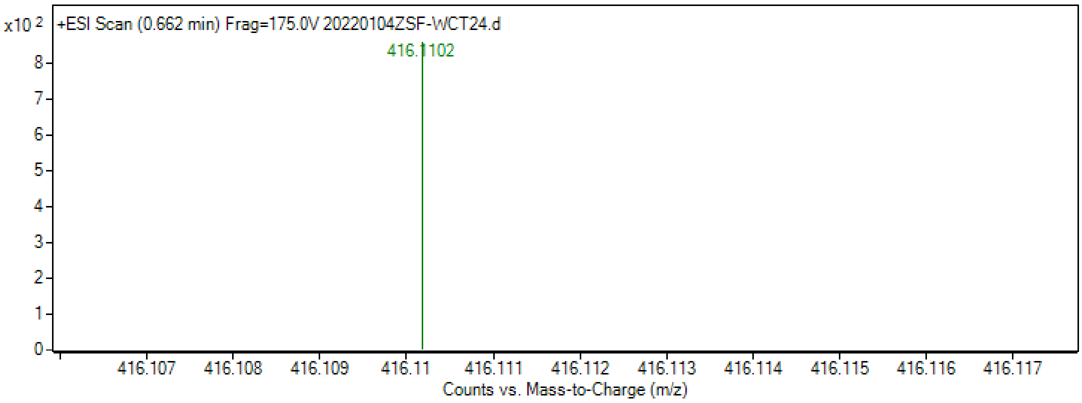

N-(4,4-difluorobut-2-yn-1-yl)-4-methyl-N-(2-phenylallyl)benzenesulfonamide (1o)

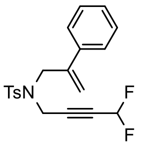

Colorless oil, 77% yield, Rf = 0.2 (PE: EtOAc = 10: 1). **^{1}H NMR** (500 MHz, Chloroform-*d*) δ 7.75 – 7.70 (m, 2H), 7.54 – 7.49 (m, 2H), 7.38 – 7.27 (m, 5H), 5.84 (t, *J* = 54.7 Hz, 1H), 5.59 (s, 1H), 5.31 (s, 1H), 4.24 (s, 2H), 4.06 – 4.00 (m, 2H), 2.43 (s, 3H). **^{13}C NMR** (126 MHz, $CDCl_3$) δ 144.30, 141.04, 137.25, 134.92, 129.72, 128.58, 128.39, 127.85, 126.39, 117.72, 102.97 (t, *J* = 232.7 Hz),), 81.94 (t, *J* = 6.9 Hz), 77.22 (t, *J* = 31.3 Hz), 50.55, 35.25 (t, *J* = 2.8 Hz), 21.51. **^{19}F NMR** (471 MHz, $CDCl_3$) δ -106.46. **HRMS**: Calculation for $C_{20}H_{18}BrF_2NO_2S$, $[M+H]^+$, 376.1177,

Found: 376.1173.

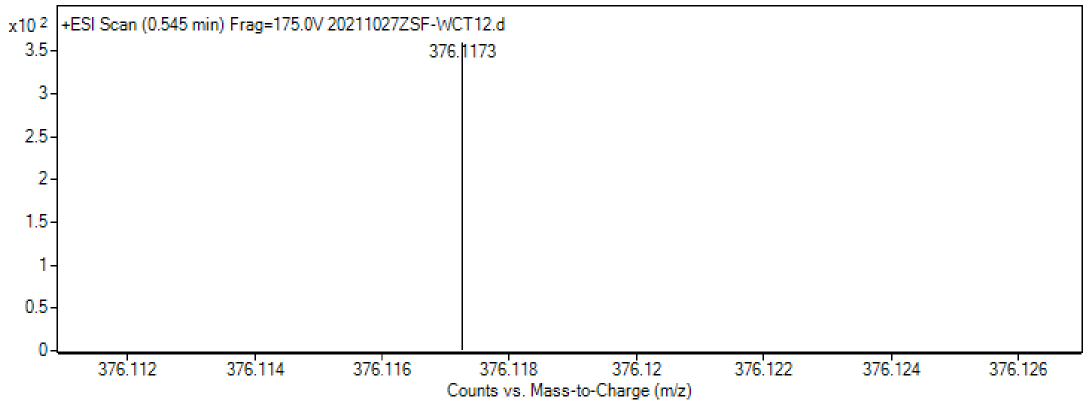

N-(2-([1,1'-biphenyl]-4-yl)allyl)-N-(4,4-difluorobut-2-yn-1-yl)-4-methylbenzenesulfonamide (1p)

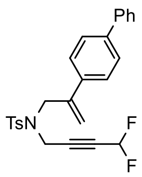

Yellow solid, 84% yield, m.p. = 135.8 - 136 °C, Rf = 0.2 (PE: EtOAc = 10: 1). **^{1}H NMR** (500 MHz, Chloroform-*d*) δ 7.75 (d, *J* = 8.2 Hz, 2H), 7.64 – 7.56 (m, 6H), 7.44 (t, *J* = 7.7 Hz, 2H), 7.34 (dd, *J* = 13.7, 7.7 Hz, 3H), 5.84 (t, *J* = 54.7 Hz, 1H), 5.65 (s, 1H), 5.33 (s, 1H), 4.28 (s, 2H), 4.06 (t, *J* = 4.6 Hz, 2H), 2.43 (s, 3H). **^{13}C NMR** (126 MHz, $CDCl_3$) δ 144.30, 141.18, 140.58, 140.52, 136.07, 134.95, 129.72, 128.84, 127.87, 127.49, 127.24, 127.03, 126.81, 117.70, 102.96 (t, *J* = 232.9 Hz), 81.95 (t, *J* = 6.9 Hz), 77.14 (t, *J* = 34.0 Hz), 50.60, 35.28, 21.51. **^{19}F NMR** (471 MHz, $CDCl_3$) δ -106.43. **HRMS**: Calculation for $C_{26}H_{23}F_2NO_2S$, $[M+H]^+$, 452.1490, Found: 452.1488.

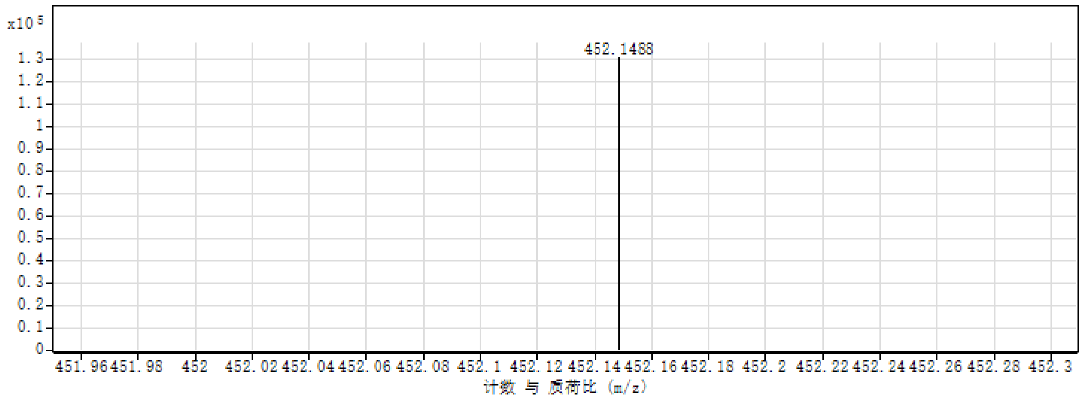

N-(4,4-difluorobut-2-yn-1-yl)-4-methyl-N-(2-(p-tolyl)allyl)benzenesulfonamide (1q)

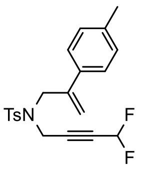

Yellow solid, 80% yield, m.p. = 76.1 - 76.3 °C, Rf = 0.2 (PE: EtOAc = 10: 1). **^{1}H NMR** (500 MHz, Chloroform-*d*) δ 7.73 (d, *J* = 8.3 Hz, 2H), 7.42 (d, *J* = 8.1 Hz, 2H), 7.32 (d, *J* = 8.1 Hz, 2H), 7.15 (d, *J* = 8.0 Hz, 2H), 5.83 (t, *J* = 54.7 Hz, 1H), 5.55 (s, 1H), 5.25 (s, 1H), 4.22 (s, 2H), 4.02 (t, *J* = 4.6 Hz, 2 H), 2.43 (s, 3H), 2.34 (s, 3H). **^{13}C NMR** (126 MHz, $CDCl_3$) δ 144.23, 140.85, 138.28, 134.98, 134.33, 129.69, 129.26, 127.85, 126.28, 116.87, 102.97 (t, *J* = 232.7 Hz),

81.94 (t, J = 6.9 Hz), 77.22 (t, J = 31.3 Hz), 50.59, 35.20, 21.51, 21.16. **^{19}F NMR** (471 MHz, $CDCl_3$) δ -106.45. **HRMS**: Calculation for $C_{21}H_{21}F_2NO_2S$, $[M+H]^+$, 390.1334 Found: 390.1332.

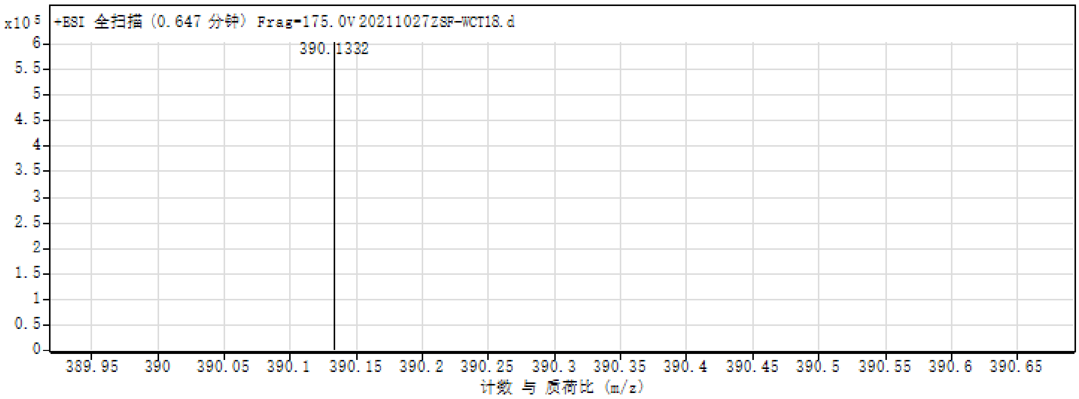

N-(4,4-difluorobut-2-yn-1-yl)-N-(2-(4-methoxyphenyl)allyl)-4-methylbenzenesulfonamide (1r)

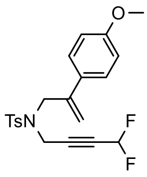

Yellow solid, 72% yield, m.p. = 101.8 - 101.9 ºC, Rf = 0.4 (PE: EtOAc = 10: 1). **^{1}H NMR** (500 MHz, Chloroform-d) δ 7.73 (d, J = 8.2 Hz, 2H), 7.48 (d, J = 8.8 Hz, 2H), 7.32 (d, J = 8.1 Hz, 2H), 6.88 (d, J = 8.8 Hz, 2H), 5.83 (t, J = 54.6 Hz, 1H), 5.50 (s, 1H), 5.20 (s, 1H), 4.20 (s, 2H), 4.02 (t, J = 4.6 Hz, 2H), 3.80 (s, 3H), 2.42 (s, 3H). **^{13}C NMR** (126 MHz, $CDCl_3$) δ 159.80, 144.26, 140.24, 134.93, 129.69, 129.55, 127.84, 127.61, 116.01, 113.93, 102.98 (t, J = 232.8 Hz), 81.99 (t, J = 7.2 Hz), 77.05 (t, J = 34.0 Hz). 55.28, 50.69, 35.15, 21.48. **^{19}F NMR** (471 MHz, $CDCl_3$) δ -106.44. **HRMS**: Calculation for $C_{21}H_{21}F_2NO_3S$, $[M+H^+]^+$, 406.1283 Found: 406.1277.

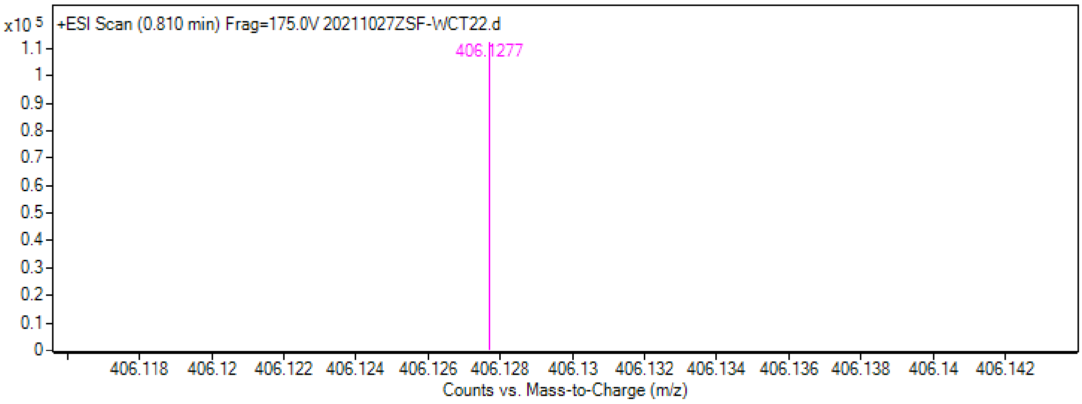

N-(4,4-difluorobut-2-yn-1-yl)-N-(2-(4-fluorophenyl)allyl)-4-methylbenzenesulfonamide (1s)

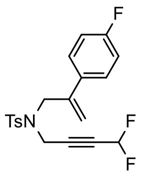

White solid, 62% yield, m.p. = 95.9 - 96.1 ºC, Rf = 0.2 (PE: EtOAc = 10: 1). **^{1}H NMR** (500 MHz, Chloroform-d) δ 7.75 – 7.68 (m, 2H), 7.54 – 7.47 (m, 2H), 7.33 (d, J = 8.1 Hz, 2H), 7.03 (t, J = 8.7 Hz, 2H), 5.84 (t, J = 54.6 Hz, 1H), 5.54 (s, 1H), 5.29 (s, 1H), 4.21 (s, 2H), 4.02 (td, J = 4.8, 1.2 Hz, 2H), 2.43 (s, 3H). **^{13}C NMR** (126 MHz, $CDCl_3$) δ 162.82 (d, J =

248.1 Hz), 144.40, 140.03, 134.81, 133.25 (d, J = 3.4 Hz), 129.74, 128.18 (d, J = 8.2 Hz), 127.81, 117.69, 115.44 (d, J = 21.9 Hz), 102.95 (t, J = 233.0 Hz), 81.76 (t, J = 7.1 Hz), 77.16 (t, J = 34.0 Hz), 50.68, 35.20, 21.48. **^{19}F NMR** (471 MHz, $CDCl_3$) δ -106.49, -113.55. **HRMS**: Calculation for $C_{20}H_{18}F_3NO_2S$, $[M+H]^+$, 394.1083 Found: 394.1073.

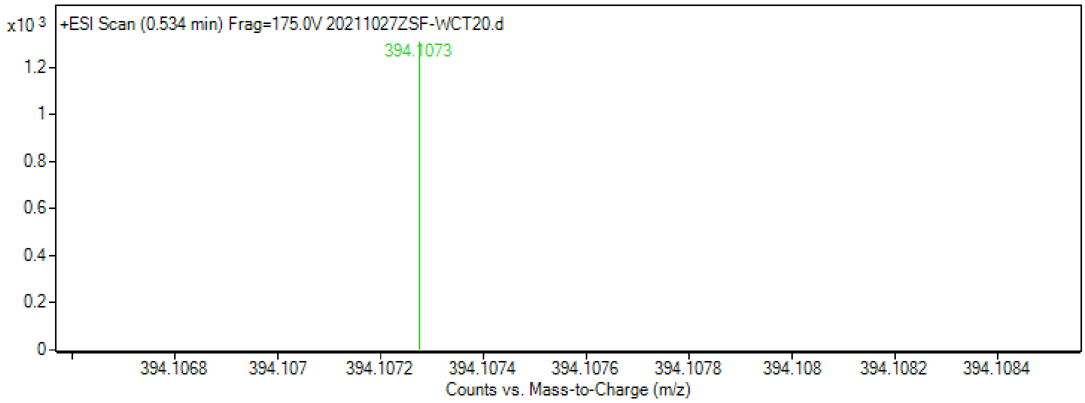

N-(2-(4-chlorophenyl)allyl)-N-(4,4-difluorobut-2-yn-1-yl)-4-methylbenzenesulfonamide (1t)

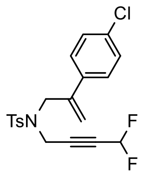

White solid, 77% yield, m.p. = 79.7 - 79.9 °C, Rf = 0.2 (PE: EtOAc = 10: 1). **^{1}H NMR** (500 MHz, Chloroform-*d*) δ 7.72 (d, J = 8.3 Hz, 2H), 7.46 (d, J = 8.6 Hz, 2H), 7.32 (t, J = 8.8 Hz, 4H), 5.84 (t, J = 54.6 Hz, 1H), 5.58 (s, 1H), 5.32 (s, 1H), 4.21 (s, 2H), 4.01 (t, J = 4.6 Hz, 2H), 2.43 (s, 3H). **^{13}C NMR** (126 MHz, $CDCl_3$) δ 144.42, 140.02, 135.60, 134.78, 134.28, 129.75, 128.73, 127.80, 127.76, 118.30, 102.93 (t, J = 232.9 Hz), 81.71 (t, J = 7.1 Hz), 77.19 (t, J = 34.2 Hz), 50.54, 35.23, 21.51. **^{19}F NMR** (471 MHz, $CDCl_3$) δ -106.48. **HRMS**: Calculation for $C_{20}H_{18}ClF_2NO_2S$, $[M+H]^+$, 410.0788 Found: 410.0784.

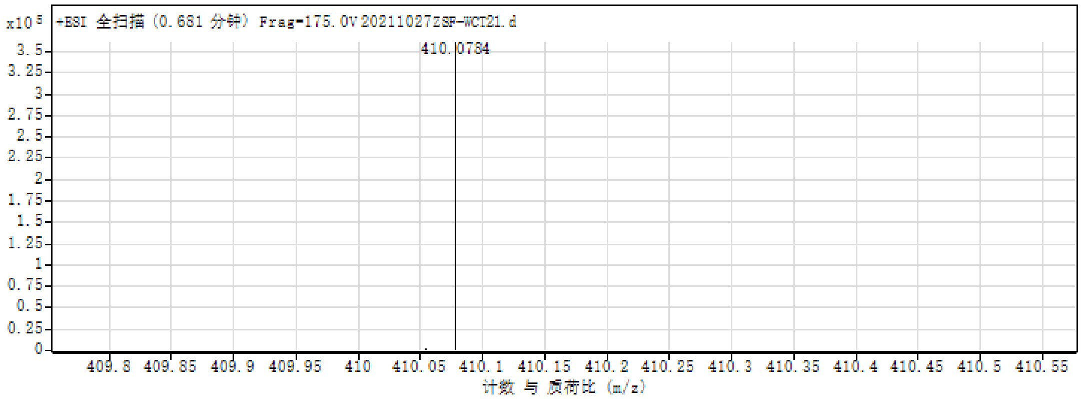

N-(2-(4-bromophenyl)allyl)-N-(4,4-difluorobut-2-yn-1-yl)-4-methylbenzenesulfonamide (1u)

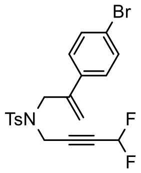

Yellow solid, 72% yield, m.p. = 62.1 - 62.2 ºC, Rf = 0.2 (PE: EtOAc = 10: 1). **^{1}H NMR** (500 MHz, Chloroform-*d*) δ 7.71 (d, *J* = 8.0 Hz, 2H), 7.46 (d, *J* = 8.2 Hz, 2H), 7.39 (d, *J* = 8.3 Hz, 2H), 7.32 (d, *J* = 8.0 Hz, 2H), 5.84 (t, *J* = 54.6 Hz, 1H), 5.59 (s, 1H), 5.32 (s, 1H), 4.21 (s, 2H), 4.01 (t, *J* = 4.9 Hz, 2H), 2.43 (s, 3H). **^{13}C NMR** (126 MHz, $CDCl_3$) δ 144.44, 140.11, 136.09, 134.78, 131.70, 129.77, 128.08, 127.81, 122.52, 118.40, 102.95 (t, *J* = 232.9 Hz), 81.72 (t, *J* = 7.0 Hz), 77.20 (t, *J* = 34.4 Hz), 50.49, 35.24, 21.52. **^{19}F NMR** (471 MHz, $CDCl_3$) δ -106.46. **HRMS**: Calculation for $C_{20}H_{18}BrF_2NO_2S$, $[M+Na]^+$, 476.0102, Found: 476.0104.

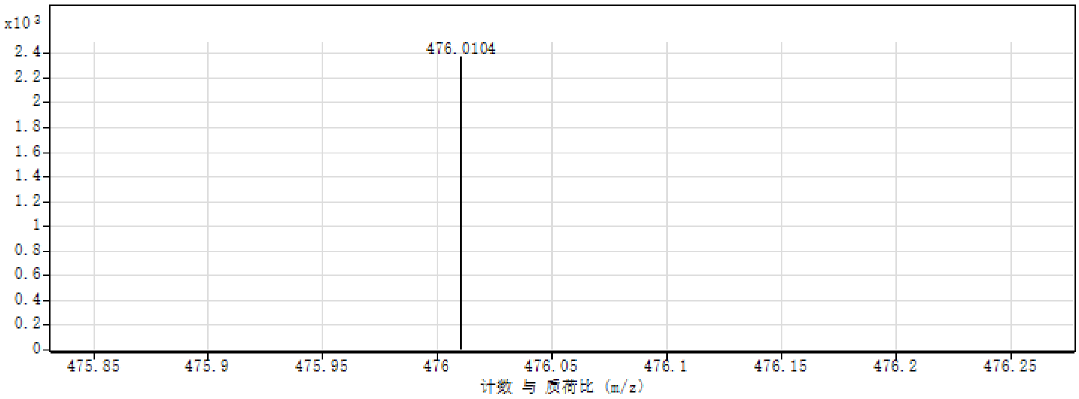

N-(4,4-difluorobut-2-yn-1-yl)-4-methyl-N-(2-(4-(trifluoromethyl)phenyl)allyl)benzenesulfonamide (1v)

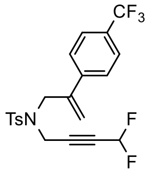

Yellow solid, 57% yield, m.p. = 73.7 - 73.9 ºC, Rf = 0.2 (PE: EtOAc = 10: 1). **^{1}H NMR** (500 MHz, Chloroform-*d*) δ 7.72 (d, *J* = 8.0 Hz, 2H), 7.64 (d, *J* = 8.3 Hz, 2H), 7.59 (d, *J* = 8.3 Hz, 2H), 7.33 (d, *J* = 8.0 Hz, 2H), 5.85 (t, *J* = 54.6 Hz, 1H), 5.67 (s, 1H), 5.42 (s, 1H), 4.26 (s, 2H), 4.02 (t, *J* = 4.8 Hz, 2H), 2.43 (s, 3H). **^{13}C NMR** (126 MHz, $CDCl_3$) δ 144.53, 140.79, 140.14, 134.73, 130.21 (q, *J* = 32.3 Hz), 129.78, 127.79,125.80, 125.49 (q, *J* = 3.9 Hz), 124.12 (d, *J* = 272.0 Hz), 119.84, 102.93 (t, *J* = 232.7 Hz), 81.57 (t, *J* = 7.2 Hz), 77.28 (t, *J* = 34.4 Hz), 50.49, 35.27 (d, *J* = 2.5 Hz), 21.44. **^{19}F NMR** (471 MHz, CDCl3) δ -62.64, -106.57. **HRMS**: Calculation for $C_{21}H_{18}F_5NO_2S$, $[M+H]^+$, 444.1051, Found: 444.1048.

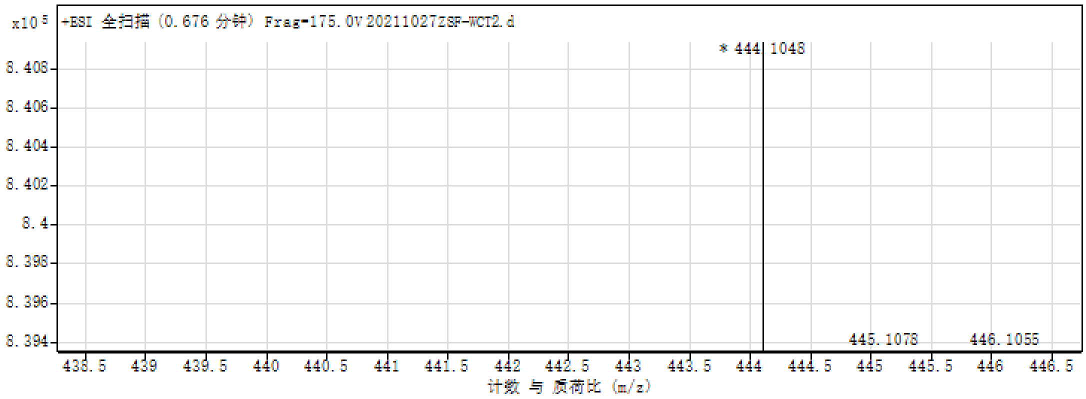

N-(4,4-difluorobut-2-yn-1-yl)-N-(2-(3-methoxyphenyl)allyl)-4-methylbenzenesulfonamide (1w)

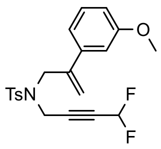

Yellow solid, 70% yield, m.p. = 108.7 - 108.8 ºC, Rf = 0.4 (PE: EtOAc = 10: 1). ^{1}**H NMR** (500 MHz, Chloroform-*d*) δ 7.72 (d, *J* = 8.0 Hz, 2H), 7.32 (d, *J* = 8.0 Hz, 2H), 7.25 (t, *J* = 8.1 Hz, 1H), 7.13 – 7.09 (m, 2H), 6.86 (dd, *J* = 8.2, 2.4 Hz, 1H), 5.84 (t, *J* = 54.6 Hz, 1H), 5.61 (s, 1H), 5.30 (s, 1H), 4.22 (s, 2H), 4.04 (t, *J* = 4.9 Hz, 2H), 3.82 (d, *J* = 2.1 Hz, 3H), 2.42 (s, 3H). ^{13}**C NMR** (126 MHz, $CDCl_3$) δ 159.75, 144.31, 140.89, 138.67, 134.95, 129.73, 129.52, 127.81, 118.76, 117.91, 114.31, 111.86, 103.00 (t, *J* = 232.7 Hz), 81.92 (t, *J* = 7.2 Hz), 77.02 (t, *J* = 32.2 Hz), 55.34, 50.60, 35.26 (d, *J* = 2.7 Hz). 21.50. ^{19}**F NMR** (471 MHz, $CDCl_3$) δ -106.44. **HRMS**: Calculation for C21H21F2NO3S, $[M+H]^+$, 406.1283, Found: 406.1289

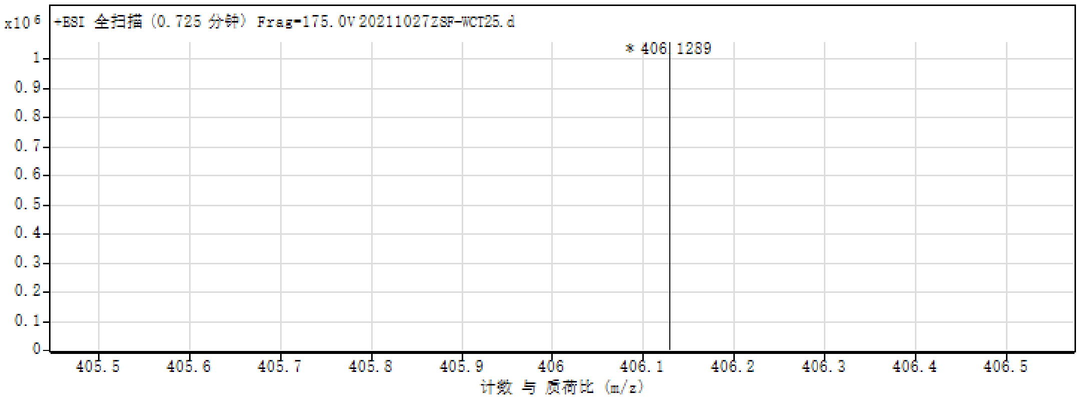

N-(4,4-difluorobut-2-yn-1-yl)-4-methyl-N-(2-(m-tolyl)allyl)benzenesulfonamide (1x)

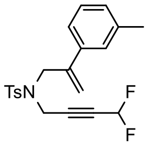

Yellow solid, 77% yield, m.p. = 68.9 – 69.0 ºC, Rf = 0.2 (PE: EtOAc = 10: 1). ^{1}**H NMR** (500 MHz, $CDCl_3$) δ 7.73 (d, *J* = 7.9 Hz, 2H), 7.32 (d, *J* = 8.6 Hz, 4H), 7.25 (d, *J* = 7.4 Hz, 1H), 7.13 (d, *J* = 7.1 Hz, 1H), 5.85(t, *J* = 55.1 Hz, 1H), 5.57 (s, 1H), 5.29 (s, 1H), 4.23 (s, 2H), 4.05 (s, 2H), 2.43 (s, 3H), 2.36 (s, 3H). ^{13}**C NMR** (126 MHz, $CDCl_3$) δ 144.22, 141.20, 138.12, 137.30, 135.02, 129.69, 129.14, 128.46, 127.83, 127.13, 123.50, 117.44, 102.96 (d, *J* = 233.1 Hz), 82.01 (d, *J* = 6.9 Hz), 79.69 – 72.72 (m), 50.56, 35.27, 21.49 (d, *J* = 5.2 Hz). ^{19}**F NMR** (471 MHz,

CDCl$_3$) δ -106.45. **HRMS**: Calculation for $C_{21}H_{21}F_2NO_2S$, $[M+H]^+$, 390.1334, Found: 390.1331

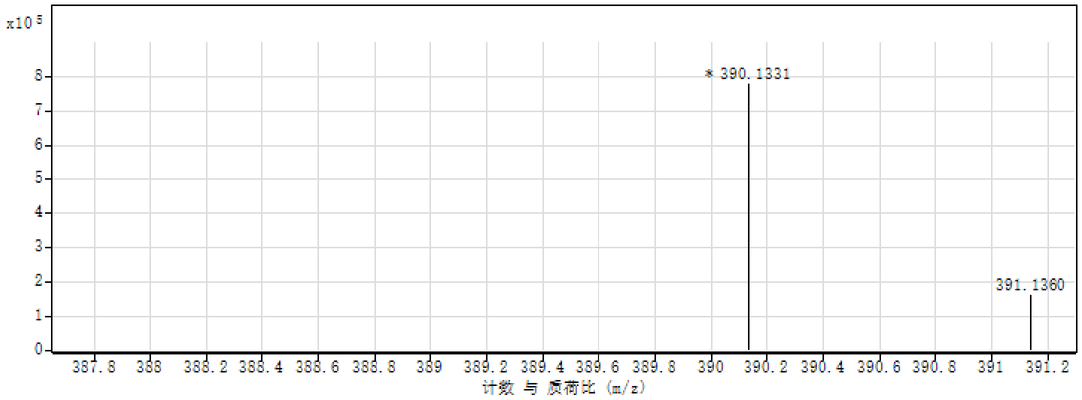

N-(2-(3-chlorophenyl)allyl)-N-(4,4-difluorobut-2-yn-1-yl)-4-methylbenzenesulfonamide (1y)

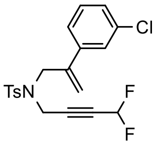

Yellow solid, 73% yield, m.p. = 87.8-88.0 ºC, Rf = 0.2 (PE: EtOAc = 10: 1). **^{1}H NMR** (500 MHz, Chloroform-*d*) δ 7.73 (d, *J* = 8.2 Hz, 2H), 7.46 (s, 1H), 7.43 – 7.40 (m, 1H), 7.33 (d, *J* = 8.1 Hz, 2H), 7.29 (d, *J* = 4.9 Hz, 2H), 5.86 (t, *J* = 54.6 Hz, 1H), 5.60 (s, 1H), 5.36 (s, 1H), 4.21 (s, 2H), 4.04 (t, *J* = 4.5 Hz, 2H), 2.44 (s, 3H). **^{13}C NMR** (126 MHz, CDCl$_3$) δ 144.40, 140.08, 139.17, 134.83, 134.49, 129.86, 129.76, 128.40, 127.81, 126.54, 124.64, 118.85, 102.92 (t, *J* = 233.0 Hz), 81.76 (t, *J* = 7.1 Hz), 77.20 (t, *J* = 34.5 Hz), 50.42, 35.31, 21.52. **^{19}F NMR** (471 MHz, CDCl$_3$) δ -106.51. **HRMS**: Calculation for $C_{20}H_{18}ClF_2NO_2S$, $[M+H]^+$, 410.0788, Found: 410.0784.

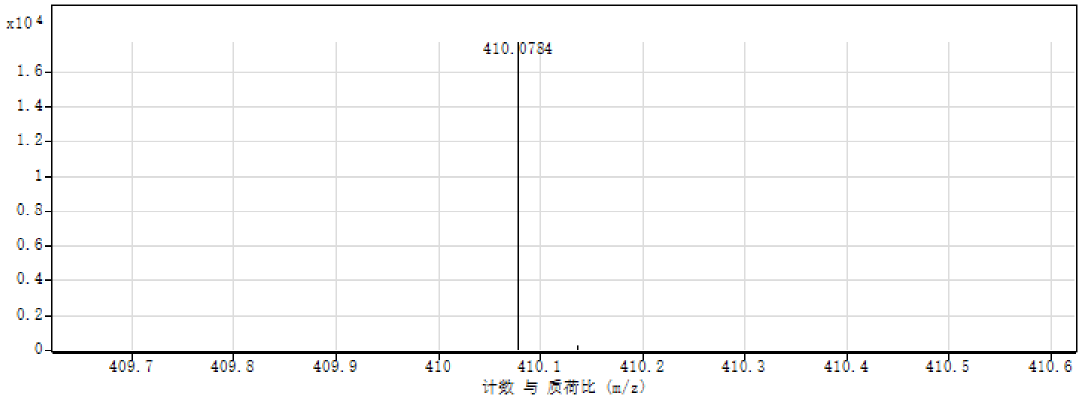

N-(4,4-difluorobut-2-yn-1-yl)-N-(2-(3-fluorophenyl)allyl)-4-methylbenzenesulfonamide (1z)

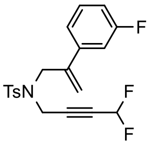

Colorless oil, 60% yield, Rf = 0.2 (PE: EtOAc = 10: 1). **^{1}H NMR** (400 MHz, Chloroform-*d*) δ 7.76 (d, *J* = 8.3 Hz, 2H), 7.39 – 7.29 (m, 4H), 7.26 – 7.18 (m, 1H), 7.03 (ddt, *J* = 8.9, 5.7, 2.8 Hz, 1H), 5.88 (t, *J* = 54.6 Hz, 1H), 5.64 (s, 1H), 5.39 (s, 1H), 4.24 (s, 2H), 4.07 (t, *J* = 4.7 Hz, 2H), 2.46 (s, 3H). **^{13}C NMR** (101 MHz, CDCl$_3$) δ 162.89 (d, *J* = 245.7 Hz), 144.43, 140.07 (d, *J* = 2.3 Hz), 139.52 (d, *J* = 7.6 Hz), 134.77, 130.09 (d, *J* = 8.3 Hz), 129.76, 127.82, 122.13 (d, *J* = 2.8 Hz), 118.81, 115.23 (d, *J* = 21.2 Hz), 113.35 (d, *J* = 22.3 Hz), 104.09 (t, *J* =

232.9 Hz), 81.74 (t, J = 7.1 Hz), 77.36 (t, J = 34.2 Hz), 50.45, 35.28, 21.51. **^{19}F NMR** (376 MHz, $CDCl_3$) δ -106.51, -112.79. **HRMS**: Calculation for $C_{20}H_{18}F_3NO_2S$, $[M+H]^+$, 394.1085, Found: 394.1085.

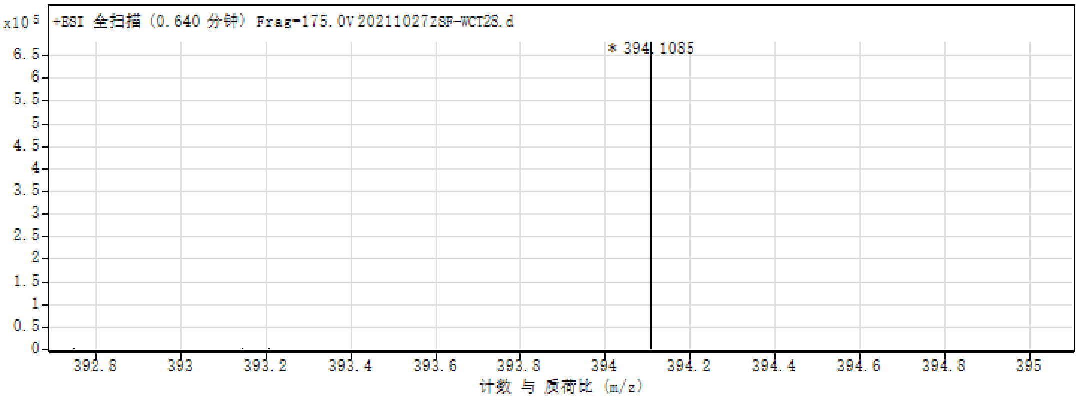

N-(4,4-difluorobut-2-yn-1-yl)-N-(2-mesitylallyl)-4-methylbenzenesulfonamide (1aa)

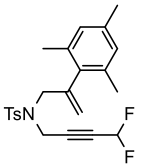

Yellow solid, 73% yield, m.p. = 74.8 - 75.0 °C, Rf = 0.2 (PE: EtOAc = 10: 1). **^{1}H NMR** (500 MHz, Chloroform-*d*) δ 7.69 (d, J = 8.0 Hz, 2H), 7.26 (d, J = 7.9 Hz, 2H), 6.86 (s, 2H), 5.84 (t, J = 54.5 Hz, 1H), 5.57 (s, 1H), 5.06 (s, 1H), 4.33 (t, J = 4.1 Hz, 2H), 3.86 (s, 2H), 2.38 (s, 3H), 2.26 (s, 3H), 2.21 (s, 6H). **^{13}C NMR** (126 MHz, $CDCl_3$) δ 144.15, 141.98, 137.11, 135.95, 135.85, 135.64, 129.71, 128.40, 127.58, 114.79, 102.97 (t, J = 233.0 Hz), 82.49 (t, J = 7.1 Hz), 76.94 (t, J = 34.5 Hz), 50.73, 36.54, 21.46, 20.97, 19.55. **^{19}F NMR** (471 MHz, $CDCl_3$) δ -106.66. **HRMS**: Calculation for $C_{23}H_{25}F_2NO_2S$, $[M+H]^+$, 418.1647 Found: 418.1649.

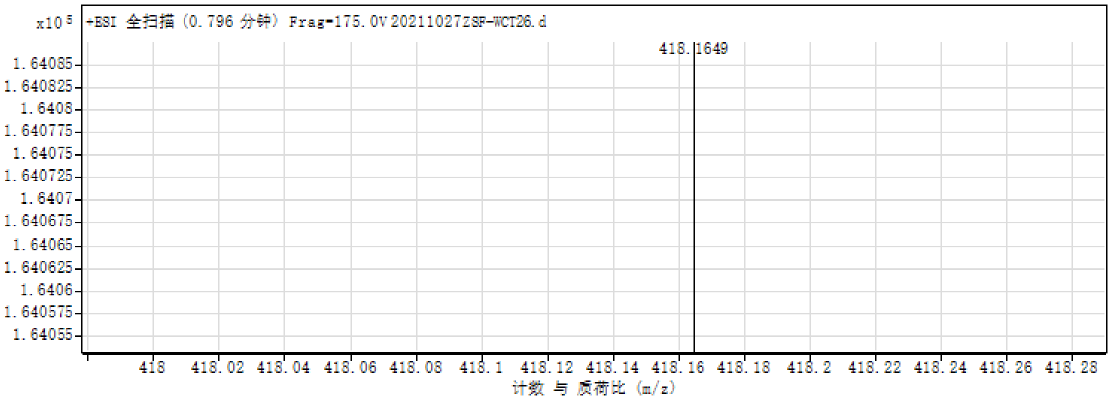

N-(4,4-difluorobut-2-yn-1-yl)-4-methyl-N-(2-(thiophen-2-yl)allyl)benzenesulfonamide (1ab)

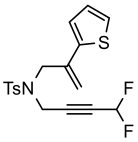

Yellow solid, 82% yield, m.p. = 91.4 - 91.6 °C, Rf = 0.2 (PE: EtOAc = 10: 1). **^{1}H NMR** (500 MHz, Chloroform-*d*) δ 7.75 (d, J = 8.2 Hz, 2H), 7.38 (d, J = 3.5 Hz, 1H), 7.34 (d, J = 8.1 Hz, 2H), 7.20 (d, J = 5.1 Hz, 1H), 7.06 – 6.97 (m, 1H), 5.83 (t, J = 54.6 Hz, 1H), 5.60 (s, 1H), 5.17 (s, 1H), 4.17 (s, 2H), 4.12 (t, J = 4.7 Hz, 2H), 2.44 (s, 3H). **^{13}C NMR** (126 MHz, $CDCl_3$) δ 144.40, 141.03, 134.79, 134.74, 129.76, 127.92, 127.83, 125.75, 125.10, 116.19, 102.92 (t, J = 232.8

Hz), 81.74 (t, J = 7.2 Hz), 77.27 (t, J = 34.0 Hz), 50.84, 35.29, 21.51. ^{19}**F NMR** (471 MHz, $CDCl_3$) δ -106.49. **HRMS**: Calculation for $C_{18}H_{17}F_2NO_2S_2$, $[M+H]^+$, 382.0742 Found: 382.0742.

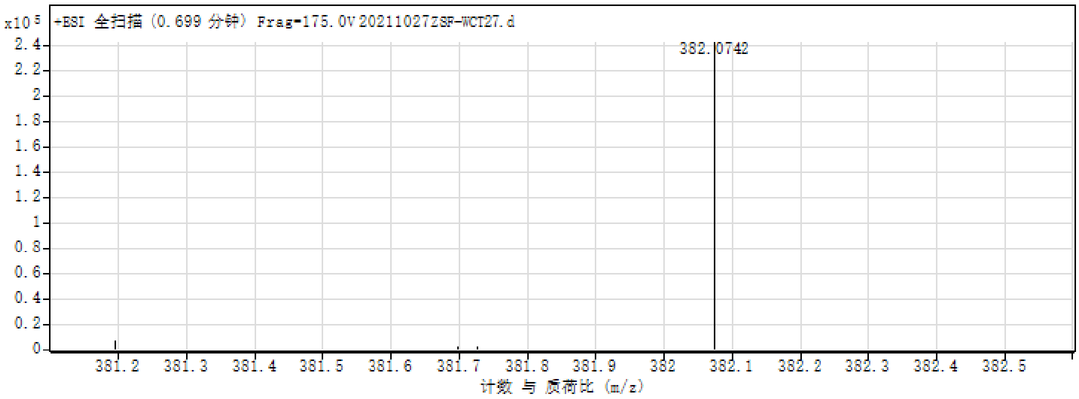

N-(4,4-difluorobut-2-yn-1-yl)-N-(2-(furan-2-yl)allyl)-4-methylbenzenesulfonamide (1ac)

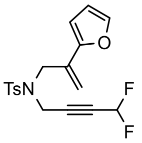

Colorless oil, 80% yield, Rf = 0.2 (PE: EtOAc = 15: 1). ^{1}**H NMR** (400 MHz, Chloroform-*d*) δ 7.78 (d, J = 8.3 Hz, 2H), 7.42 – 7.34 (m, 3H), 6.77 (d, J = 3.4 Hz, 1H), 6.43 (dd, J = 3.4, 1.8 Hz, 1H), 5.93 (d, J = 54.6 Hz, 1H), 5.72 (s, 1H), 5.20 (s, 1H), 4.15 (t, J = 4.8 Hz, 2H), 4.10 (s, 2H), 2.46 (s, 3H). ^{13}**C NMR** (101 MHz, $CDCl_3$) δ 151.49, 144.40, 142.46, 134.77, 130.48, 129.76, 127.80, 114.59, 111.65, 108.69, 104.05 (t, J = 232.9 Hz), 81.70 (d, J = 7.3 Hz), 77.09 (t, J = 34.5 Hz), 49.30, 35.14, 21.53. ^{19}**F NMR** (376 MHz, $CDCl_3$) δ -106.52. **HRMS**: Calculation for $C_{18}H_{17}F_2NO_3S$, $[M+Na]^+$, 388.0790 Found: 388.0781.

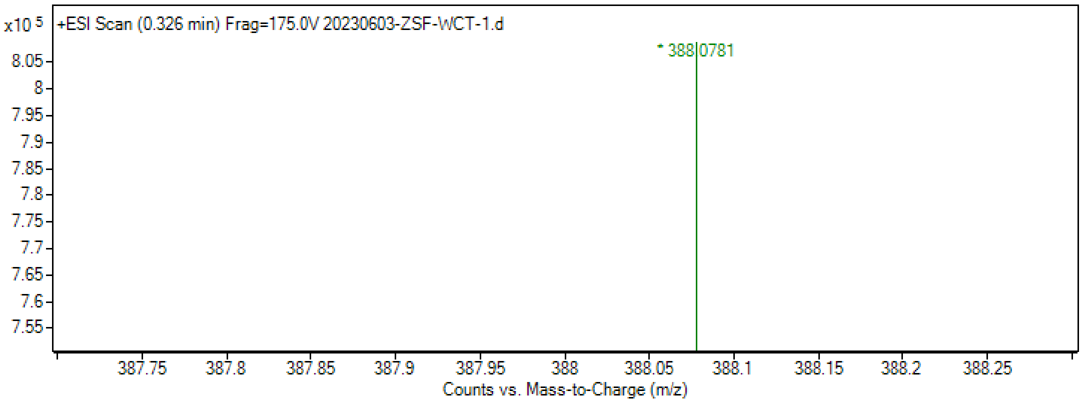

N-allyl-N-(4,4-difluorobut-2-yn-1-yl)-4-methylbenzenesulfonamide (1ad)

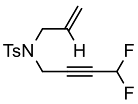

Colorless oil, 78% yield, Rf = 0.2 (PE: EtOAc = 20: 1). ^{1}**H NMR** (400 MHz, Chloroform-*d*) δ 7.72 (d, J = 8.2 Hz, 2H), 7.32 (d, J = 8.1 Hz, 2H), 6.09 – 5.63 (m, 2H), 5.34 – 5.23 (m, 2H), 4.17 (t, J = 4.8 Hz, 2H), 3.81 (d, J = 6.5 Hz, 2H), 2.43 (s, 3H). ^{13}**C NMR** (101 MHz, $CDCl_3$) δ 144.18, 135.34, 131.52, 129.71, 127.63, 120.49, 103.01 (t, J = 232.6 Hz), 82.19 (d, J = 7.2 Hz), 76.68 (t, J = 34.2 Hz), 49.52, 35.51, 21.47. ^{19}**F NMR** (376 MHz, $CDCl_3$) δ -106.58. **HRMS**: Calculation

for $C_{14}H_{15}F_2NO_2S$, $[M+H]^+$, 300.0864 Found: 300.0867.

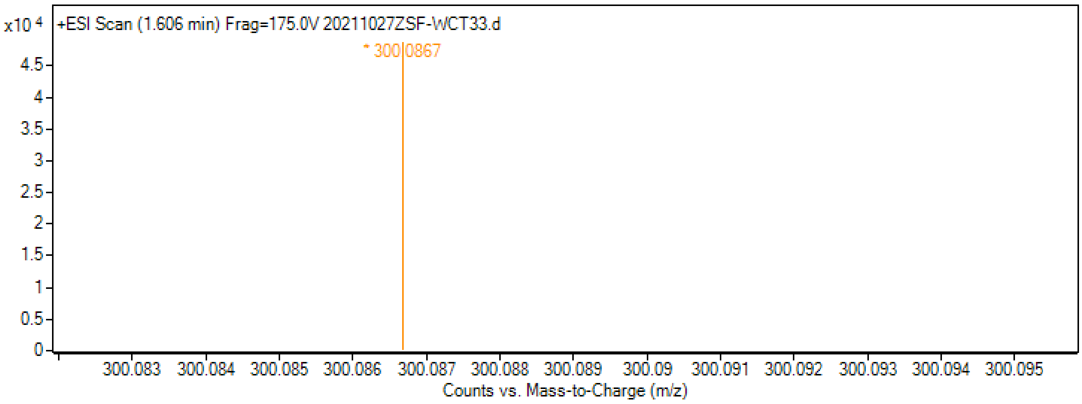

N-cinnamyl-N-(4,4-difluorobut-2-yn-1-yl)-4-methylbenzenesulfonamide (1ae)

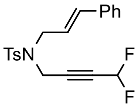

Yellow solid, 73% yield, m.p. = 76.4 - 76.6 ºC, Rf = 0.2 (PE: EtOAc = 10: 1). **^{1}H NMR** (500 MHz, Chloroform-*d*) δ 7.75 (d, J = 8.2 Hz, 2H), 7.33 (s, 6H), 7.28 – 7.22 (m, 1H), 6.56 (d, J = 15.8 Hz, 1H), 6.12 – 6.03 (m, 1H), 5.90 (t, J = 54.6 Hz, 1H), 4.20 (t, J = 4.5 Hz, 2H), 3.97 (d, J = 6.8 Hz, 2H), 2.43 (s, 3H). **^{13}C NMR** (126 MHz, $CDCl_3$) δ 144.20, 135.90, 135.50, 135.40, 129.75, 128.68, 128.26, 127.71, 126.60, 122.45, 103.04 (t, J = 232.8 Hz), 82.38 (t, J = 6.9 Hz), 76.72 (t, J = 44.1 Hz), 49.22, 35.70, 21.50. **^{19}F NMR** (471 MHz, $CDCl_3$) δ -106.41. **HRMS**: Calculation for $C_{20}H_{19}F_2NO_2S$, $[M+H]^+$, 376.1177 Found: 376.1170.

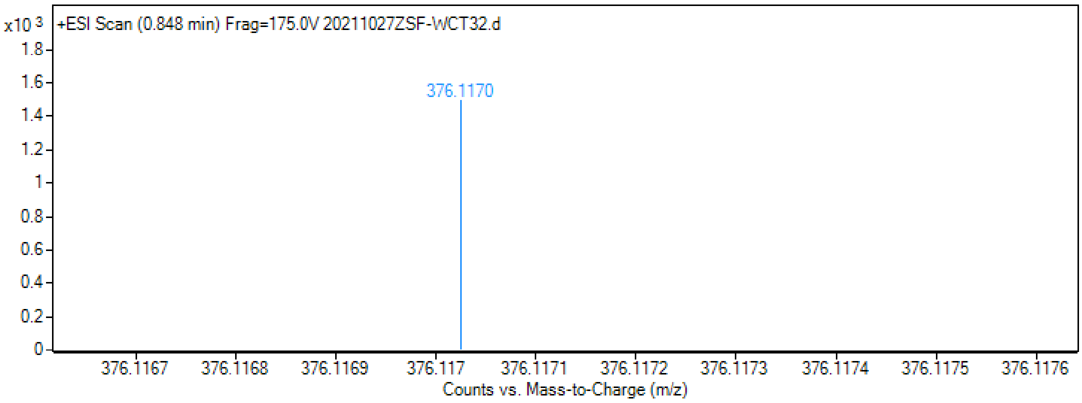

1-(3-((4,4-difluorobut-2-yn-1-yl)oxy)prop-1-en-2-yl)-4-methylbenzene (1af)

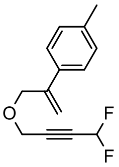

Colorless oil, 88% yield, Rf = 0.5 (PE: EtOAc = 50:1). **^{1}H NMR** (500 MHz, Chloroform-*d*) δ 7.37 (d, J = 7.7 Hz, 2H), 7.15 (d, J = 7.9 Hz, 2H), 6.21 (t, J = 54.7 Hz, 1H), 5.55 (s, 1H), 5.31 (s, 1H), 4.46 (s, 2H), 4.25 (t, J = 4.9 Hz, 2H), 2.34 (s, 3H). **^{13}C NMR** (126 MHz, $CDCl_3$) δ 142.84, 137.91, 135.32, 129.19, 125.94, 115.02, 103.52 (t, J = 232.4 Hz), 85.21 (t, J = 7.1 Hz), 77.43 (t, J = 31.2 Hz), 76.02, 56.39, 21.14. **^{19}F NMR** (471 MHz, $CDCl_3$) δ -106.17. **HRMS**: Calculation for $C_{14}H_{14}F_2O$, $[M+H]^+$, 237.1086 Found: 237.1073.

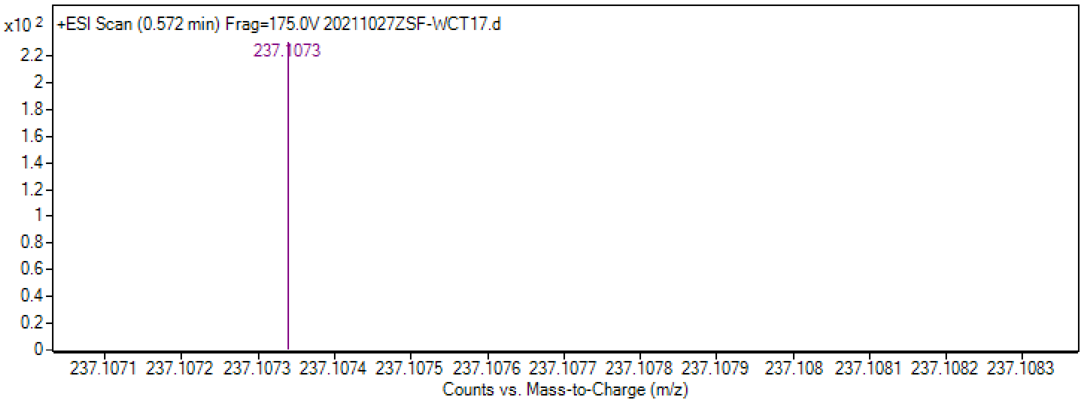

N-(4,4-difluorobut-2-yn-1-yl)-4-methyl-N-(3-methylbut-3-en-1-yl)benzenesulfonamide (1ag)

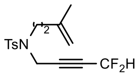

Light yellow oil, 68% yield, Rf = 0.5 (PE: EtOAc = 20:1). **^{1}H NMR** (400 MHz, $CDCl_3$) δ 7.77 – 7.69 (m, 2H), 7.33 (d, *J* = 5.6 Hz, 2H), 6.22 – 5.64 (m, 1H), 4.84 (s, 1H), 4.76 (s, 1H), 4.23 (s, 2H), 3.61 – 3.23 (m, 2H), 2.43 (s, 3H), 2.29 (d, *J* = 7.5 Hz, 2H), 1.78 (s, 3H). **^{13}C NMR** (101 MHz, Chloroform-*d*) δ 144.07, 141.88, 135.26, 129.66, 127.61, 112.76, 102.99 (t, *J* = 232.8 Hz), 82.21 (t, *J* = 7.1 Hz), 76.48 (t, *J* = 34.5 Hz), 44.92, 35.99, 35.87, 22.02, 21.47. **^{19}F NMR** (376 MHz, Chloroform-*d*) δ -106.68. HRMS: Calculation for $C_{16}H_{19}F_2NO_2S$, [M+Na]+, 350.0997 Found: 350.1003.

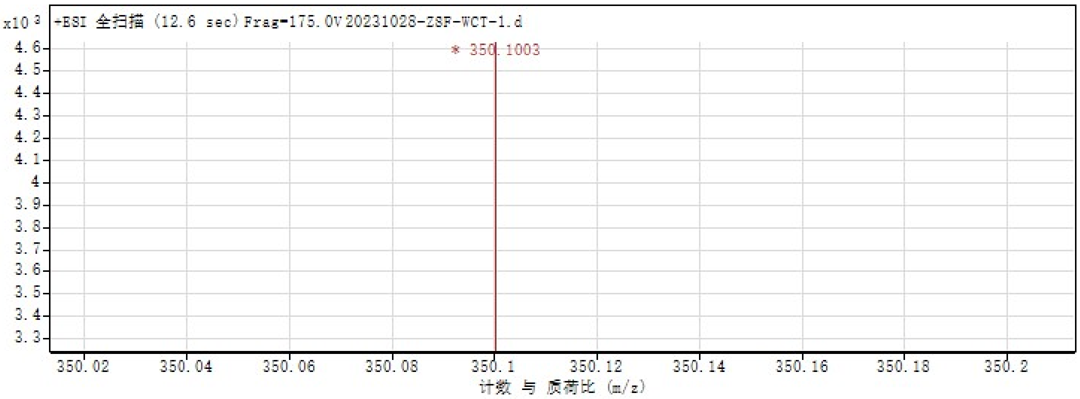

4-methyl-N-(2-methylallyl)-N-(4,4,4-trifluorobut-2-yn-1-yl)benzenesulfonamide (1ah)

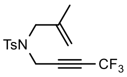

Light yellow oil, 15% yield, Rf = 0.5 (PE: EtOAc = 20:1). **^{1}H NMR** (400 MHz, CDCl3) δ 7.73 (d, *J* = 7.9 Hz, 2H), 7.35 (d, *J* = 7.9 Hz, 2H), 5.05 – 4.91 (m, 2H), 4.15 (d, *J* = 3.3 Hz, 2H), 3.73 (s, 2H), 2.44 (s, 3H), 1.79 (s, 3H). **^{13}C NMR** (101 MHz, Chloroform-*d*) δ 144.42, 138.64, 135.05, 129.79, 127.52, 120.47 – 105.51 (m), 80.56 (q, *J* = 6.6 Hz), 72.61 (q, *J* = 53.2 Hz), 53.19, 34.89, 21.44, 19.56. **^{19}F NMR** (376 MHz, Chloroform-*d*) δ -50.84. HRMS: Calculation for $C_{15}H_{16}F_3NO_2S$, [M+Na]+, 354.0746 Found: 354.0750.

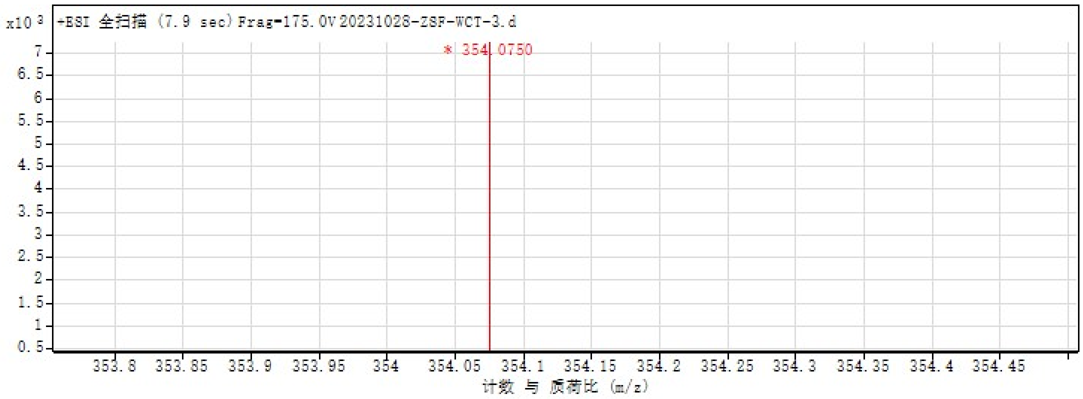

(*S*)-N-(4,4-difluorobut-2-yn-1-yl)-4-methyl-N-((4-(prop-1-en-2-yl)cyclohex-1-en-1-yl)methyl)benzenesulfonamide (1ai)

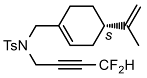

Light yellow oil, 60% yield, Rf = 0.5 (PE: EtOAc = 20:1).^{1}**H NMR** (500 MHz, CDCl3) δ 7.73 – 7.62 (m, 2H), 7.31 (d, J = 8.0 Hz, 2H), 6.08 – 5.71 (m, 1H), 5.69 (d, J = 4.5 Hz, 1H), 4.84 – 4.57 (m, 2H), 4.15 – 3.90 (m, 2H), 3.82 – 3.52 (m, 2H), 2.42 (s, 3H), 2.20 – 2.08 (m, 3H), 2.08 – 1.91 (m, 2H), 1.88 – 1.80 (m, 1H), 1.73 (s, 3H), 1.47 (qd, J = 10.9, 6.0 Hz, 1H). ^{13}**C NMR** (126 MHz, Chloroform-*d*) δ 149.29, 143.99, 135.51, 131.20, 129.62, 127.69, 127.65, 108.89, 102.09 (t, *J* = 232.6 Hz), 82.37 (t, *J* = 7.3 Hz), 76.67 (t, *J* = 34.9 Hz), 53.03, 40.74, 35.08, 30.61, 27.24, 26.20, 21.47, 20.76. ^{19}**F NMR** (471 MHz, Chloroform-*d*) δ -106.40. HRMS: Calculation for $C_{21}H_{25}F_2NO_2S$, [M+H]+, 416.1466 Found: 416.1468.

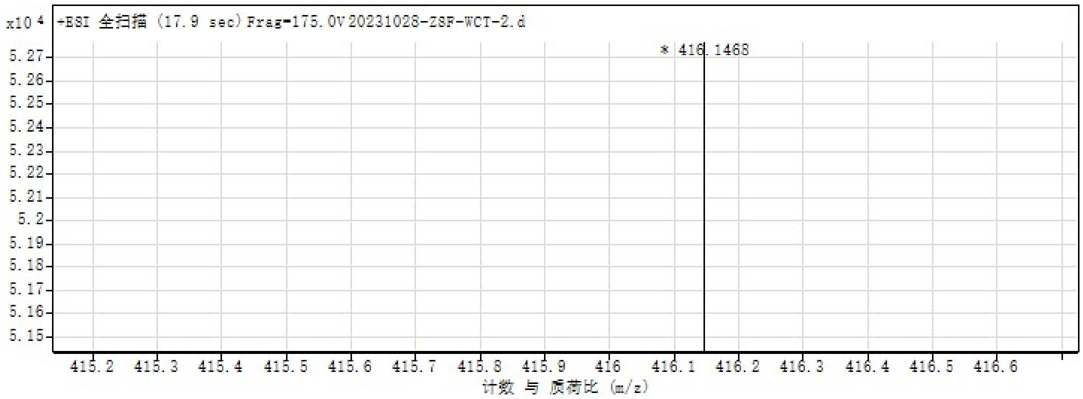

N-benzyl-N-(4,4-difluorobut-2-yn-1-yl)-4-methylbenzenesulfonamide (1aj)[1b]

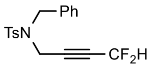

Yield: 72%, colorless oil, Rf = 0.2 (PE: EtOAc = 10: 1). ^{1}**H NMR** (500 MHz, Chloroform-d) δ 7.78 (d, *J* = 8.2 Hz, 2H), 7.33 (q, *J* = 6.5, 4.9 Hz, 7H), 5.87 (t, *J* = 54.6 Hz, 1H), 4.32 (s, 2H), 3.99 (t, *J* = 4.6 Hz, 2H), 2.44 (s, 3H). ^{13}**C NMR** (126 MHz, CDCl3) δ 144.24, 135.41, 134.45, 129.77, 128.86, 128.78, 128.37, 127.74, 103.00 (t, *J* = 232.9 Hz), 82.02 (t, *J* = 7.0 Hz), 77.02 (t, *J* = 34.5 Hz), 50.43, 35.34, 21.50. ^{19}**F NMR** (471 MHz, $CDCl_3$) δ -106.48.

N-benzyl-N-(4-fluorobut-2-yn-1-yl)-4-methylbenzenesulfonamide (1ak)

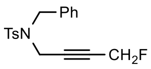

Yield: 75%, colorless oil, Rf = 0.2 (PE: EtOAc = 10: 1). **^{1}H NMR** (500 MHz, Chloroform-*d*) δ 7.78 (d, *J* = 8.2 Hz, 2H), 7.39 – 7.29 (m, 7H), 4.68 (d, *J* = 47.5 Hz, 2H) 4.33 (s, 2H), 4.02 (d, *J* = 7.0 Hz, 2H), 2.43 (s, 3H). **^{13}C NMR** (126 MHz, $CDCl_3$) δ 143.84, 135.82, 134.85, 129.56, 128.76, 128.20, 127.87, 82.58 (d, *J* = 11.9 Hz), 79.75 (d, *J* = 22.2 Hz), 70.11 (d, *J* = 166.2 Hz), 50.19, 35.84 (d, *J* = 2.8 Hz), 21.53. **^{19}F NMR** (471 MHz, $CDCl_3$) δ -215.63. **HRMS**: Calculation for $C_{19}H_{19}FO_2S$, $[M+H]^+$, 332.1115 Found: 332.1108.

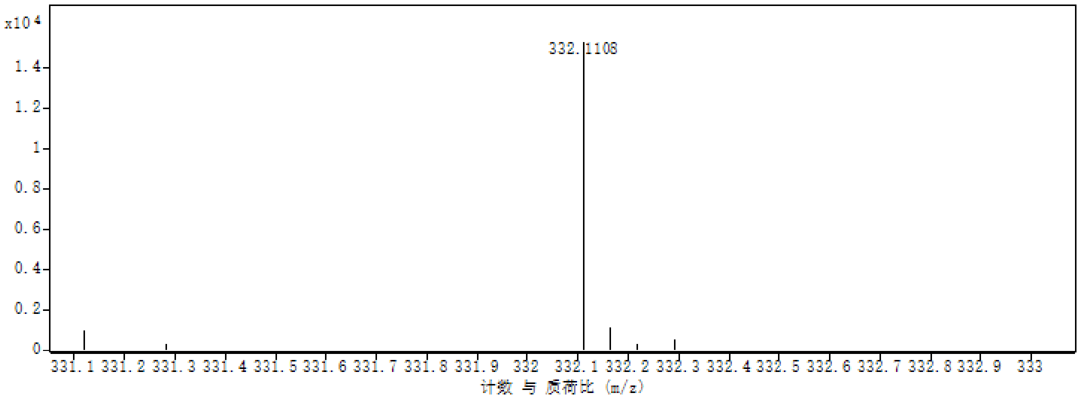

N-benzyl-4-methyl-N-(prop-2-yn-1-yl)benzenesulfonamide (1al)[2]

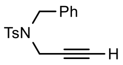

Known compound. Yield: 88%, white solid, Rf = 0.2 (PE: EtOAc = 20: 1).**^{1}H NMR** (500 MHz, Chloroform-*d*) δ 7.79 (d, *J* = 8.2 Hz, 2H), 7.58 – 7.27 (m, 7H), 4.35 (s, 2H), 3.94 (d, *J* = 2.4 Hz, 2H), 2.44 (s, 3H), 2.01 (s, 1H). **^{13}C NMR** (126 MHz, $CDCl_3$) δ 143.65, 136.05, 134.93, 129.54, 128.80, 128.71, 128.14, 127.87, 76.29, 74.13, 49.81, 35.55, 21.58.

N-(4-fluorobut-2-yn-1-yl)-4-methyl-N-(2-methylallyl)benzenesulfonamide (1am)

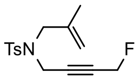

Colorless oil, 73% yield, Rf = 0.2 (PE: EtOAc = 15: 1). **^{1}H NMR** (500 MHz, $CDCl_3$) δ 7.73 (d, *J* = 8.4 Hz, 2H), 7.30 (d, *J* = 8.0 Hz, 2H), 4.98 (t, *J* = 1.7 Hz, 1H), 4.95 (s, 1H), 4.68 (t, *J* = 1.9 Hz, 1H), 4.58 (t, *J* = 1.8 Hz, 1H), 4.10 (dt, *J* = 7.1, 1.9 Hz, 2H), 3.71 (s, 2H), 2.42 (s, 3H), 1.76 (s, 3H). **^{13}C NMR** (126 MHz, $CDCl_3$) δ 143.68, 139.11, 135.83, 129.44, 127.80, 115.65, 82.65 (d, *J* = 11.9 Hz), 79.41 (d, *J* = 22.1 Hz), 70.07 (d, *J* = 165.5 Hz), 52.73, 35.67 (d, *J* = 2.9 Hz), 21.48, 19.65. **^{19}F NMR** (471 MHz, $CDCl_3$) δ -215.69. **HRMS**: Calculation for $C_{15}H_{18}FNO_2S$, $[M+H]^+$, 296.1115 Found: 296.1114.

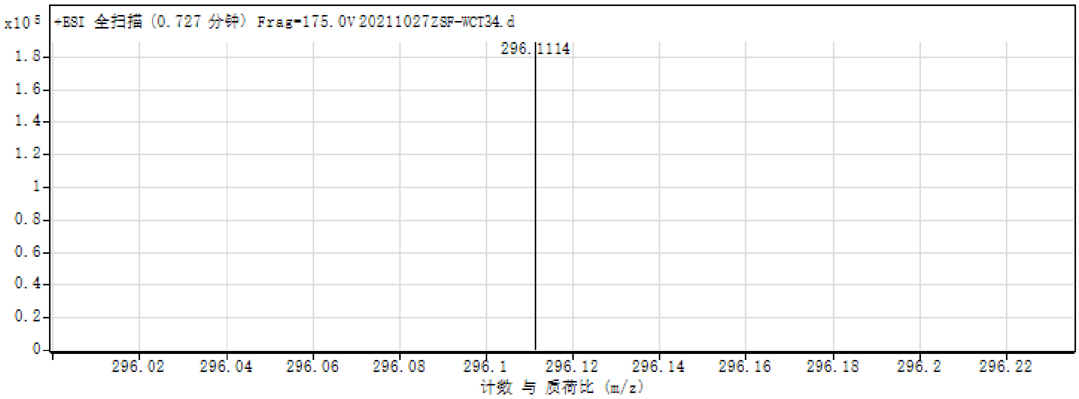

4-methyl-N-(2-methylallyl)-N-(prop-2-yn-1-yl)benzenesulfonamide (1an)[3]

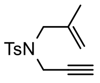

Known compound. 73% yield, Rf = 0.2 (PE: EtOAc = 15: 1). **^{1}H NMR** (500 MHz, Chloroform-*d*) δ 7.74 (d, *J* = 8.2 Hz, 2H), 7.29 (d, *J* = 8.0 Hz, 2H), 4.97 (s, 2H), 4.05 (d, *J* = 2.4 Hz, 2H), 3.74 (s, 2H), 2.42 (s, 3H), 1.96 (d, *J* = 2.4 Hz, 1H), 1.76 (s, 3H). **^{13}C NMR** (126 MHz, $CDCl_3$) δ 143.49, 139.16, 136.08, 129.42, 127.80, 115.54, 76.38, 73.71, 52.40, 35.46, 21.53, 19.65.

N-(but-2-yn-1-yl)-4-methyl-N-(2-methylallyl)benzenesulfonamide (1ao)[4]

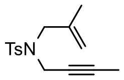

Known compound. 73% yield, Rf = 0.2 (PE: EtOAc = 15: 1). **^{1}H NMR** (500 MHz, Chloroform-*d*) δ 7.74 (d, *J* = 8.0 Hz, 1H), 7.29 (d, *J* = 8.0 Hz, 1H), 4.95 (s, 1H), 3.97 (q, *J* = 2.4 Hz, 1H), 3.70 (s, 1H), 2.42 (s, 1H), 1.76 (s, 2H), 1.50 (t, *J* = 2.3 Hz, 2H). **^{13}C NMR** (126 MHz, $CDCl_3$) δ 143.19, 139.47, 136.23, 129.17, 127.90, 115.09, 81.54, 71.48, 52.41, 36.03, 21.46, 19.70, 3.14.

4-methyl-N-(2-methylallyl)-N-(4-oxo-4-(p-tolyl)but-2-yn-1-yl)benzenesulfonamide (1ap)

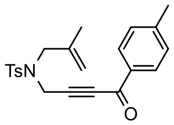

88% yield, White solid. m.p. = 66.0 - 66.9 °C. **^{1}H NMR** (500 MHz, Chloroform-*d*) δ 7.76 (d, *J* = 8.1 Hz, 2H), 7.70 (d, *J* = 8.1 Hz, 2H), 7.21 (t, *J* = 7.0 Hz, 4H), 5.01 (d, *J* = 7.5 Hz, 2H), 4.32 (s, 2H), 3.82 (s, 2H), 2.43 (s, 3H), 2.20 (s, 3H), 1.80 (s, 3H). **^{13}C NMR** (126 MHz, $CDCl_3$) δ 176.44, 145.44, 144.07, 138.87, 135.48, 133.82, 129.79, 129.51, 129.27, 127.68, 116.18, 86.30, 83.56, 53.17, 35.88, 21.81, 21.34, 19.67. **HRMS**: Calculation for $C_{22}H_{23}NO_3S$, $[M+H]^+$, 382.1471 Found: 382.1474.

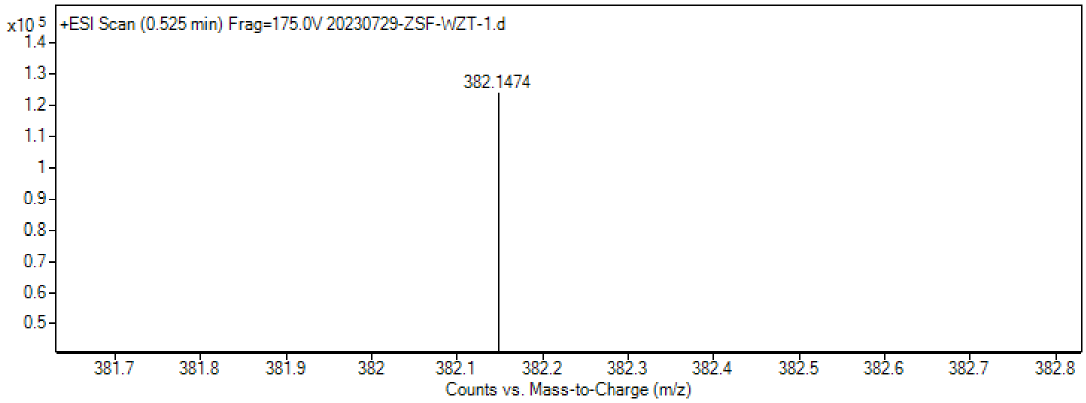

N-(4,4-difluorobut-2-yn-1-yl-4-d)-4-methyl-N-(2-methylallyl)benzenesulfonamide (1aq)

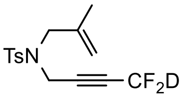

Colorless oil, 73% yield, Rf = 0.2 (PE: EtOAc = 25: 1). **^{1}H NMR** (400 MHz, $CDCl_3$) δ 7.71 (d, *J* = 8.4 Hz, 2H), 7.30 (d, *J* = 8.3 Hz, 2H), 4.96 (d, *J* = 11.0 Hz, 2H), 4.09 (t, *J* = 4.8 Hz, 2H), 3.70 (s, 2H), 2.40 (s, 3H), 1.74 (s, 3H). **^{13}C NMR** (101 MHz, Chloroform-d) δ 144.13, 138.81, 135.34, 129.65, 127.62, 116.04, 107.13 – 98.77 (m), 82.00 (t, J = 7.1 Hz), 76.63 (t, J = 34.5 Hz), 52.96, 35.17, 21.40, 19.53. **^{19}F NMR** (376 MHz, $CDCl_3$) δ -107.30 (t, *J* = 8.2 Hz). **HRMS**: Calculation for $C_{15}H_{16}DF_2NO_2S$, $[M+H]^+$, 315.1084 Found: 315.1087.

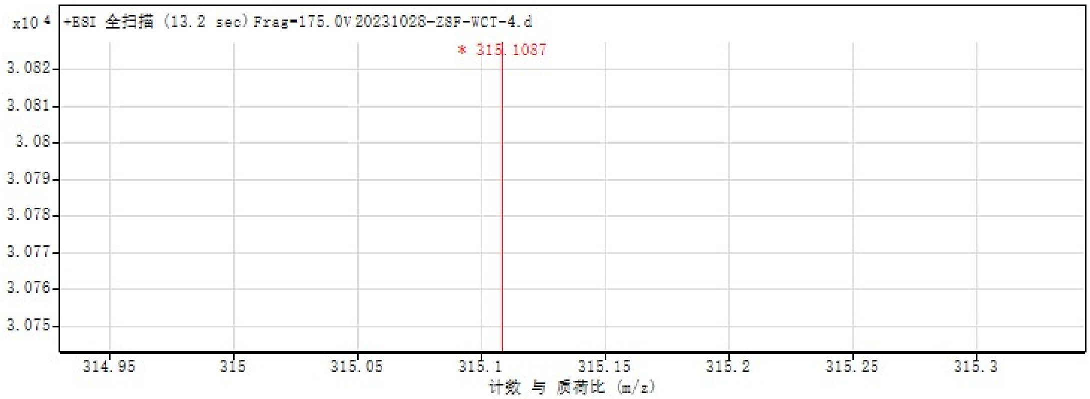

3. General procedure for Rh-catalyzed enyne cycloisomerization

To a toluene solution of **1** (0.05 mmol, 0.4 ml) in Schlenk tube with a magnetic bar was added 0.2 mol% (0.18 mg) Rh_2(*S*-BTPCP)$_4$ (2% Rh_2(*S*-BTPCP)$_4$ catalyst was dissolved in 1 mL toluene and take two tenths of it) under N_2. The sealed tube was then stirred at room temperature under nitrogen atmosphere for 48 h. The mixture was then concentrated and the residue was purified by chromatography on silica gel (eluent: PE: EtOAc) to afford the desired product **2**.

(1*R*,6*S*)-6-(difluoromethyl)-1-methyl-3-tosyl-3-azabicyclo[4.1.0]hept-4-ene (2a)

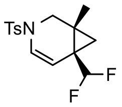

Colorless oil, 14 mg, 92% yield, Rf = 0.2 (PE: EtOAc = 15: 1). **^{1}H NMR** (500 MHz, Chloroform-*d*) δ 7.65 (d, *J* = 8.3 Hz, 2H), 7.33 (d, *J* = 8.0 Hz, 2H), 6.46 (d, *J* = 8.1 Hz, 1H), 5.72 – 5.28 (m, 2H), 3.83 (d, *J* = 2.9 Hz, 1H), 2.71 (dd, *J* = 11.6, 1.2 Hz, 1H), 2.43 (s, 3H), 1.26 (s, 3H), 1.06 (t, *J* = 4.7 Hz, 1H), 0.96 (d, *J* = 5.3 Hz, 1H). **^{13}C NMR** (126 MHz, $CDCl_3$) δ 144.08, 134.72,

129.94, 127.04, 122.96, 117.06 (t, J = 239.9 Hz), 107.10 (t, J = 4.6 Hz), 45.99, 30.47 (d, J = 6.3 Hz), 25.12 (t, J = 25.6 Hz), 22.87 (d, J = 6.4 Hz), 21.56, 17.12. ^{19}**F NMR** (471 MHz, $CDCl_3$) δ -111.37 (d, J = 284.4 Hz), -119.67 (d, J = 286.1 Hz). **HRMS**: Calculation for $C_{15}H_{17}F_2NO_2S$, $[M+Na]^+$, 336.0840 Found: 336.0840. HPLC: INA column, 99:1 hexane: isopropanol, 1.0 mL/min, t_R = major: 27.3 min, minor: 29.4 min. 99% ee. $[\alpha]_D^{29}$ = 32° (c 0.3, CH_2Cl_2).

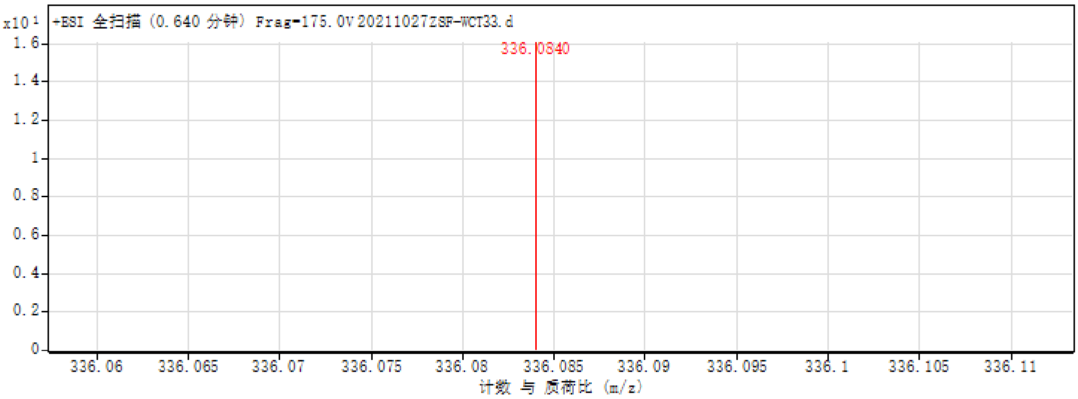

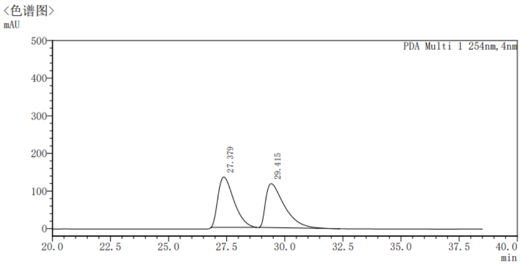

<峰表>
PDA Ch1 254nm

峰号	保留时间	面积	高度	高度%	面积%
1	27.379	6382941	133675	53.451	49.471
2	29.415	6519518	116413	46.549	50.529
总计		12902459	250088	100.000	100.000

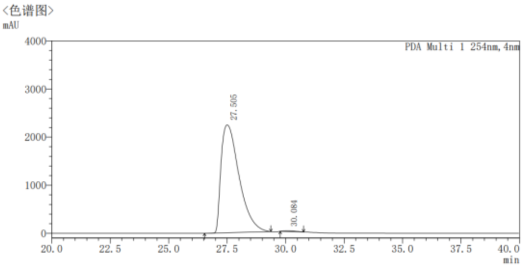

<峰表>
PDA Ch1 254nm

峰号	保留时间	面积	高度	面积%	高度%
1	27.505	120280532	2244109	99.594	99.424
2	30.084	490251	13011	0.406	0.576
总计		120770783	2257120	100.000	100.000

(1*S*,6*S*)-6-(difluoromethyl)-1-isopropyl-3-tosyl-3-azabicyclo[4.1.0]hept-4-ene (2b)

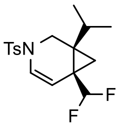

Colorless oil, 14 mg, 83% yield, Rf = 0.2 (PE: EtOAc = 10: 1). ^{1}**H NMR** (500 MHz, Chloroform-*d*) δ 7.65 (d, J = 8.1 Hz, 2H), 7.33 (d, J = 8.0 Hz, 2H), 6.45 (d, J = 8.1 Hz, 1H), 5.66 – 5.41 (m, 2H), 3.92 – 3.68 (m, 1H), 2.89 (d, J = 11.6 Hz, 1H), 2.43 (s, 3H), 1.54 – 1.45 (m, 1H), 1.09 (d, J = 7.0 Hz, 3H), 0.99 (d, J = 7.0 Hz, 3H), 0.94 – 0.89 (m, 1H), 0.82 (d, J = 5.3 Hz, 1H). ^{13}**C NMR** (126 MHz, $CDCl_3$) δ 144.05, 134.88, 129.95, 126.95, 123.02, 116.60 (t, J = 239.4 Hz), 108.17 (t, J = 4.3 Hz), 40.02, 39.92 (d, J = 6.5 Hz), 31.33, 22.43 (d, J = 5.9 Hz), 21.55, 19.72, 18.54 (d, J = 2.5 Hz). ^{19}**F NMR** (471 MHz, Chloroform-*d*) δ -107.53 (d, J = 286.0 Hz), -119.33 (d, J = 285.8 Hz). **HRMS**: Calculation for $C_{17}H_{19}F_2NO_2S$, $[M+Na^+]^+$, 364.1153 Found: 364.1144. HPLC: INC column, 95:5 hexane: isopropanol, 1.0 mL/min, t_R = major: 12.1 min, minor: 13.6 min. 99% ee. $[\alpha]_D^{29}$ = 32° (c 0.4, CH_2Cl_2).

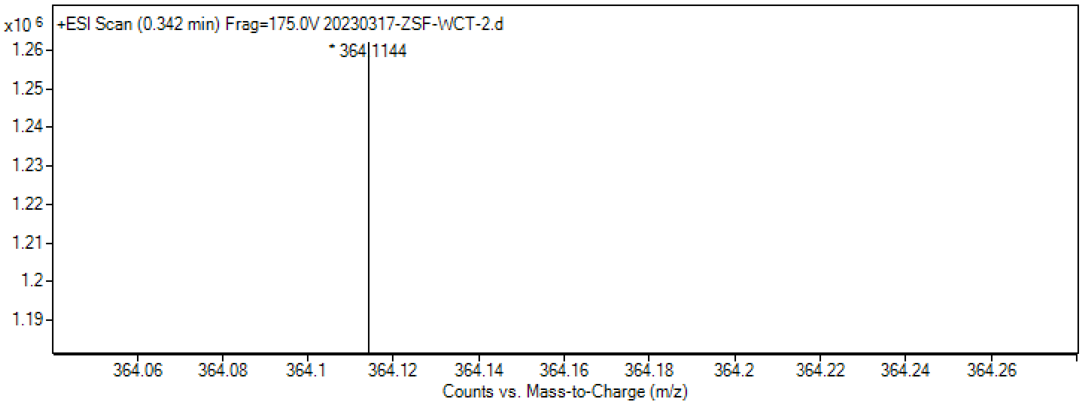

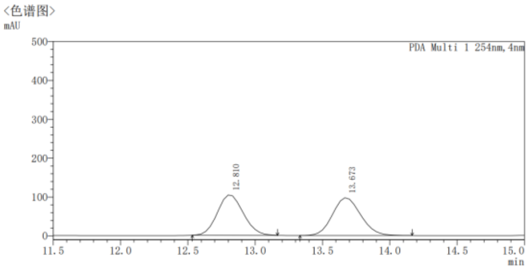

<峰表>
PDA Ch1 254nm

峰号	保留时间	面积	高度	高度%	面积%
1	12.810	1387692	103811	51.699	49.589
2	13.673	1410700	96989	48.301	50.411
总计		2798392	200801	100.000	100.000

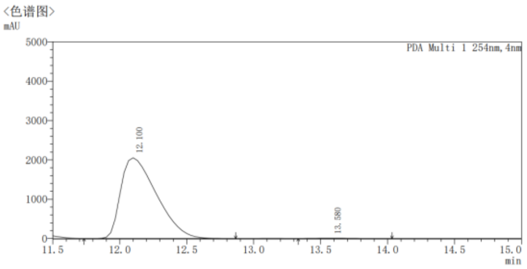

<峰表>
PDA Ch1 254nm

峰号	保留时间	面积	高度	高度%	面积%
1	12.100	38111508	2051382	99.646	99.671
2	13.580	125826	7292	0.354	0.329
总计		38237334	2058675	100.000	100.000

(1*S*,6*S*)-1-(tert-butyl)-6-(difluoromethyl)-3-tosyl-3-azabicyclo[4.1.0]hept-4-ene (2c)

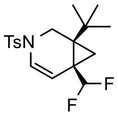

Colorless oil, 12 mg, 67% yield, Rf = 0.2 (PE: EtOAc = 10: 1). **^{1}H NMR** (500 MHz, Chloroform-*d*) δ 7.65 (d, *J* = 7.8 Hz, 2H), 7.33 (d, *J* = 7.9 Hz, 2H), 6.46 (d, *J* = 8.0 Hz, 1H), 5.82 (t, *J* = 54.8 Hz, 1H), 5.64 (d, *J* = 8.0 Hz, 1H), 3.92 – 3.86 (m, 1H), 2.96 (d, *J* = 11.8 Hz, 1H), 2.43 (s, 3H), 1.08 (d, *J* = 6.0 Hz, 1H), 1.05 – 0.99 (m, 9H), 0.98 (d, *J* = 5.2 Hz, 1H). **^{13}C NMR** (126 MHz, $CDCl_3$) δ 144.02, 134.98, 129.98, 126.94, 123.31, 117.06 (t, *J* = 239.9 Hz), 109.20 (d, *J* = 9.3 Hz), 44.47, 44.33, 44.28, 33.42, 29.26, 21.57, 18.09 (d, *J* = 7.3 Hz). **^{19}F NMR** (471 MHz, Chloroform-*d*) δ -107.05 (d, *J* = 286.1 Hz), -114.16 (d, *J* = 286.2 Hz). **HRMS**: Calculation for $C_{18}H_{23}F_2NO_2S$, $[M+H]^+$, 356.1490 Found: 356.1493. HPLC: INA column, 99:1 hexane: isopropanol, 1.0 mL/min, t_R = major: 19.5 min, minor: 15.7 min. 91% ee. $[\alpha]_D^{29}$ = 23° (c 0.9, CH_2Cl_2).

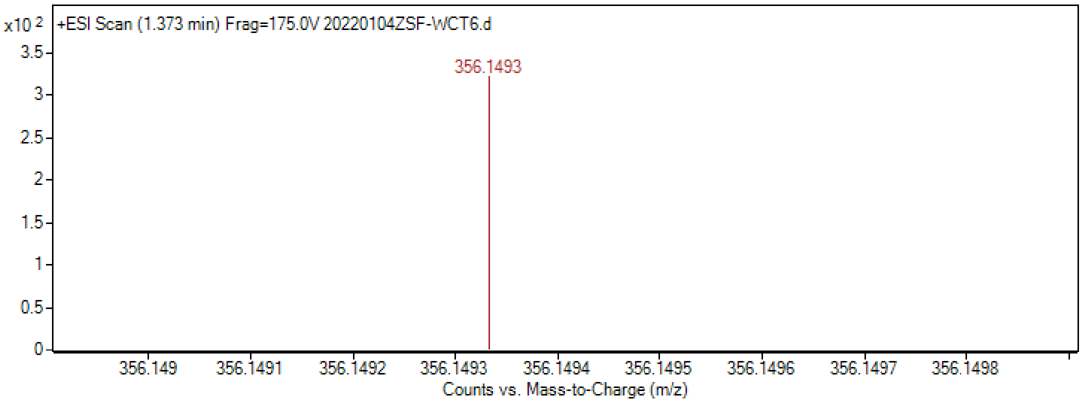

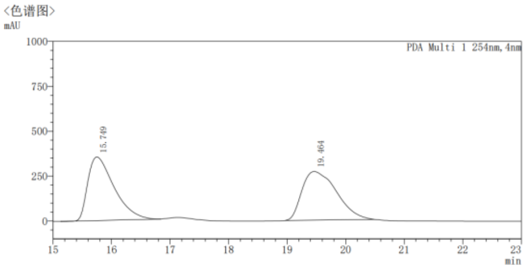

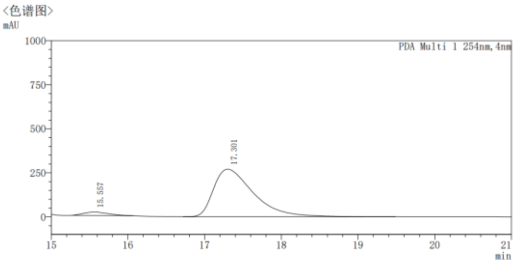

<峰表>
PDA Ch1 254nm

峰号	保留时间	面积	高度	高度%	面积%
1	15.749	11066949	353459	56.581	49.855
2	19.464	11131163	271235	43.419	50.145
总计		22198112	624694	100.000	100.000

<峰表>
PDA Ch1 254nm

峰号	保留时间	面积	高度	高度%	面积%
1	15.557	457329	19525	6.737	4.456
2	17.301	9805204	270269	93.263	95.544
总计		10262533	289794	100.000	100.000

(1*S*,6*R*)-6-(difluoromethyl)-1-hexyl-3-tosyl-3-azabicyclo[4.1.0]hept-4-ene (2d)

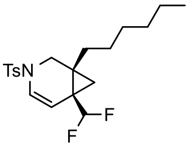

Colorless oil, 18 mg, 93% yield, Rf = 0.2 (PE: EtOAc = 10: 1). **^{1}H NMR** (500 MHz, Chloroform-*d*) δ 7.65 (d, *J* = 7.9 Hz, 2H), 7.33 (d, *J* = 7.9 Hz, 2H), 6.46 (d, *J* = 8.1 Hz, 1H), 5.82 – 5.13 (m, 2H), 3.89 (dd, *J* = 11.7, 3.0 Hz, 1H), 2.76 (d, *J* = 11.6 Hz, 1H), 2.44 (s, 3H), 1.59 (d, *J* = 11.2 Hz, 1H), 1.41 – 1.33 (m, 3H), 1.32 – 1.12 (m, 6H), 0.97 (t, *J* = 4.7 Hz, 1H), 0.90 (dt, *J* = 13.6, 6.2 Hz, 4H). **^{13}C NMR** (126 MHz, $CDCl_3$) δ 144.06, 134.80, 129.96, 127.00, 122.90, 116.89 (t, *J* = 239.8 Hz), 107.67 (t, *J* = 4.3 Hz), 44.11, 35.07 (d, *J* = 6.4 Hz), 31.73, 31.60, 29.41, 26.49, 25.30 (t, *J* = 25.9 Hz), 22.57, 22.04 (d, *J* = 5.9 Hz), 21.58, 14.03. **^{19}F NMR** (471 MHz, Chloroform-*d*) δ -110.12 (d, *J* = 284.4 Hz), -119.73 (d, *J* = 284.4 Hz). **HRMS**: Calculation for $C_{20}H_{27}F_2NO_2S$, $[M+Na]^+$, 406.1623 Found: 406.1619. HPLC: INC column, 99:1 hexane: isopropanol, 0.80 mL/min, t_R = major: 34.5 min, minor: 31.1 min. 95% ee. $[\alpha]_D^{29}$ = 17° (c 0.6, CH_2Cl_2).

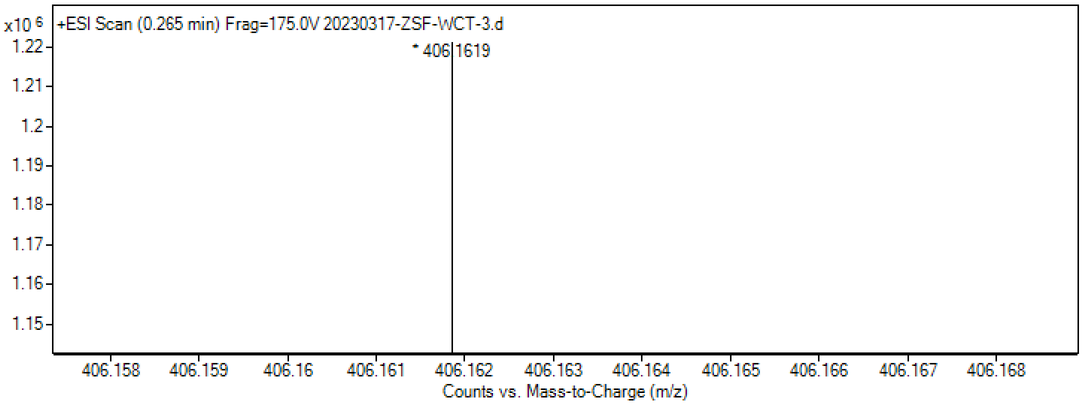

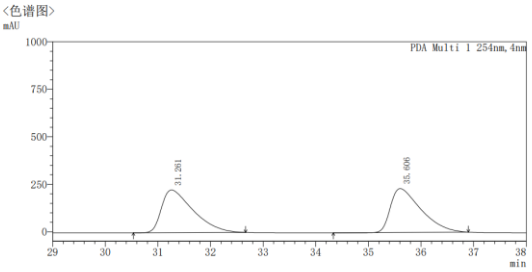

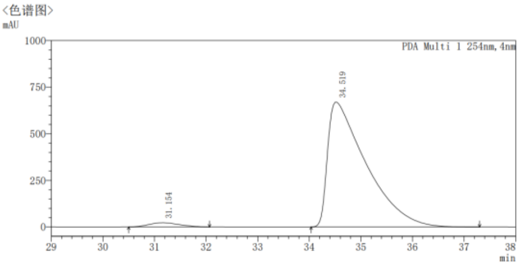

<峰表>
PDA Ch1 254nm

峰号	保留时间	面积	高度%	面积%
1	31.261	9395517	49.327	50.254
2	35.606	9300714	50.673	49.746
总计		18696231	100.000	100.000

<峰表>
PDA Ch1 254nm

峰号	保留时间	面积	高度	高度%	面积%
1	31.154	917442	22329	3.225	2.571
2	34.519	34767594	670160	96.775	97.429
总计		35685036	692489	100.000	100.000

(1*S*,6*S*)-1-cyclopropyl-6-(difluoromethyl)-3-tosyl-3-azabicyclo[4.1.0]hept-4-ene (2e)

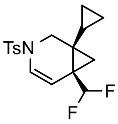

White solid, 15 mg, 89% yield, m.p. = 58.7 - 58.9 ºC, Rf = 0.2 (PE: EtOAc = 10: 1). **^{1}H NMR** (500 MHz, Chloroform-*d*) δ 7.66 (d, *J* = 8.4 Hz, 2H), 7.33 (d, *J* = 8.1 Hz, 2H), 6.48 (d, *J* = 8.2 Hz, 1H), 5.87 – 5.26 (m, 2H), 4.03 – 3.74 (m, 1H), 2.87 (dd, *J* = 11.7, 1.3 Hz, 1H), 2.43 (s, 3H), 1.08 – 0.99 (m, 1H), 0.84 (t, *J* = 5.3 Hz, 1H), 0.78 (d, *J* = 6.0 Hz, 1H), 0.75 – 0.62 (m, 1H), 0.52 (dddd, *J* = 9.5, 8.0, 6.1, 4.7 Hz, 1H), 0.26 (ddd, *J* = 11.4, 5.8, 4.2 Hz, 1H), 0.20 – 0.14 (m, 1H). **^{13}C NMR** (126 MHz, $CDCl_3$) δ 144.11, 134.67, 129.98, 127.01, 123.00, 116.62 (t, *J* = 238.6 Hz), 106.88 (t, *J* = 3.7 Hz), 45.25, 35.45 (d, *J* = 7.3 Hz), 26.14 (t, *J* = 25.2 Hz) 21.56, 17.93 (d, *J* = 6.6 Hz), 10.48, 4.35, 3.44. **^{19}F NMR** (471 MHz, Chloroform-*d*) δ -111.58 (d, *J* = 284.4 Hz), -120.06 (d, *J* = 285.9 Hz). **HRMS**: Calculation for $C_{17}H_{19}F_2NO_2S$, $[M+H]^+$, 340.1178 Found: 340.1176. HPLC: OD-H column, 95:5 hexane: isopropanol, 1.0 mL/min, t_R = major: 9.1 min, minor: 8.1 min. 97% ee. $[\alpha]_D^{29}$ = 24° (c 0.4, CH_2Cl_2).

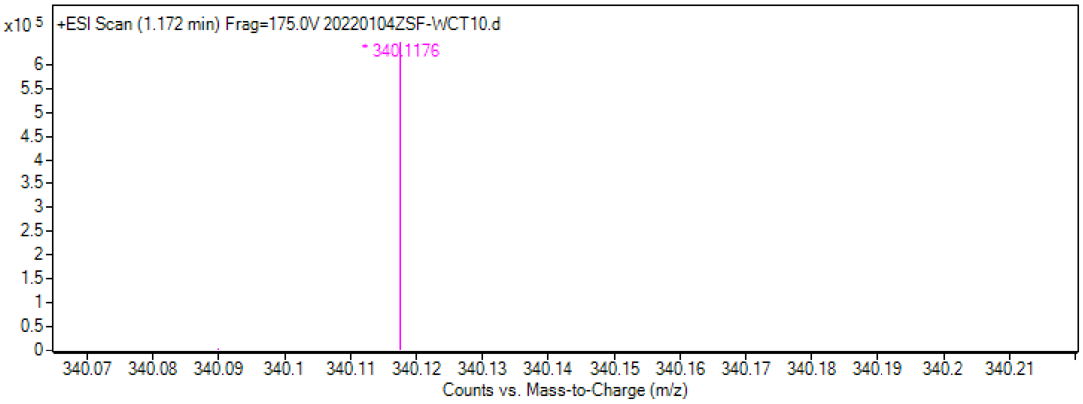

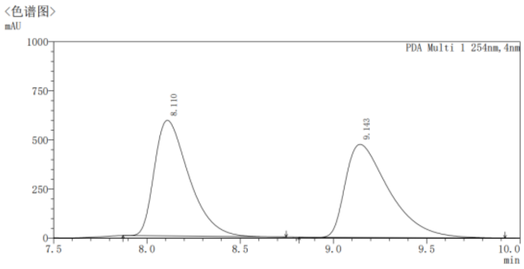

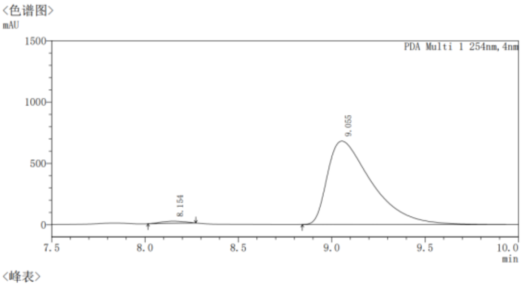

<峰表>

PDA Ch1 254nm

峰号	保留时间	面积	高度	浓度	高度%	面积%
1	8.110	7586835	588661	0.000	55.401	49.530
2	9.143	7730903	473881	0.000	44.599	50.470
总计		15317738	1062543		100.000	100.000

<峰表>

PDA Ch1 254nm

峰号	保留时间	面积	高度	浓度	高度%	面积%
1	8.154	156903	17791	0.000	2.547	1.370
2	9.055	11299893	680793	0.000	97.453	98.630
总计		11456796	698583		100.000	100.000

(1*S*,6*S*)-1-cyclopentyl-6-(difluoromethyl)-3-tosyl-3-azabicyclo[4.1.0]hept-4-ene (2f)

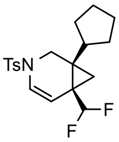

Colorless oil, 17 mg, 90% yield, Rf = 0.2 (PE: EtOAc = 10: 1). **^{1}H NMR** (500 MHz, Chloroform-*d*) δ 7.65 (d, *J* = 8.1 Hz, 2H), 7.33 (d, *J* = 7.8 Hz, 2H), 6.46 (d, *J* = 8.0 Hz, 1H), 5.81 – 5.05 (m, 2H), 3.83 (d, *J* = 11.6 Hz, 1H), 2.88 (d, *J* = 11.6 Hz, 1H), 2.43 (s, 3H), 1.80 – 1.46 (m, 8H), 1.37 (t, *J* = 12.9 Hz, 1H), 0.98 – 0.93 (m, 1H), 0.92 (s, 1H). **^{13}C NMR** (126 MHz,

$CDCl_3$) δ 144.03, 134.85, 129.94, 126.95, 123.02, 116.78 (t, J = 240.3 Hz), 107.94 (t, J = 4.6 Hz), 42.98, 41.07, 36.39 (d, J = 6.4 Hz), 29.28, 27.95, 25.80, 25.49, 22.59 (d, J = 6.5 Hz). **^{19}F NMR** (471 MHz, Chloroform-*d*) δ -109.29 (d, J = 284.7 Hz), -119.43 (d, J = 284.8 Hz). **HRMS**: Calculation for $C_{19}H_{23}F_2NO_2S$, $[M+H]^+$, 390.1310 Found: 390.1300. HPLC: INA column, 95:5 hexane: isopropanol, 1.0 mL/min, t_R = major: 10.5 min, minor: 11.5 min. 96% ee. $[\alpha]_D^{29}$ = 56° (c 1.3, CH_2Cl_2).

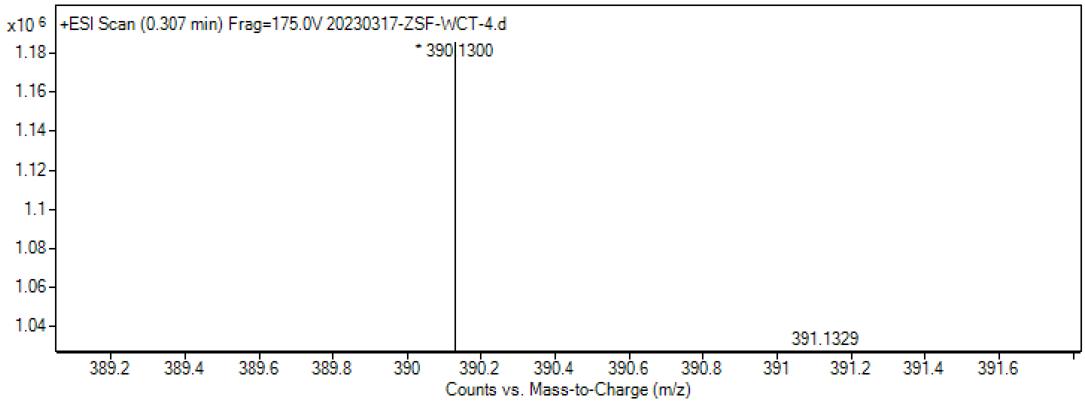

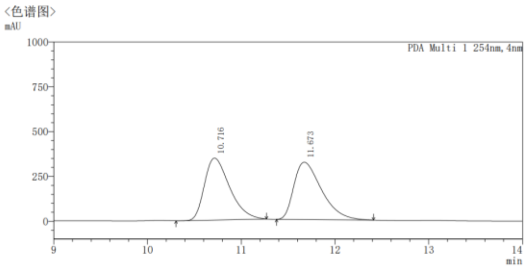

<峰表>

PDA Ch1 254nm

峰号	保留时间	面积	高度	高度%	面积%
1	10.716	6449048	345896	51.914	49.483
2	11.673	6583939	320385	48.086	50.517
总计		13032987	666281	100.000	100.000

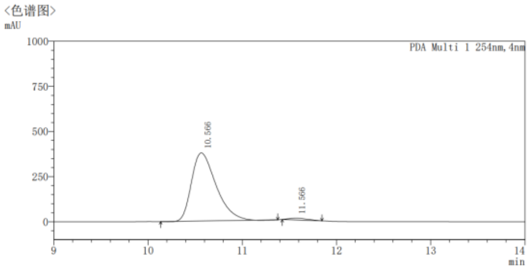

<峰表>

PDA Ch1 254nm

峰号	保留时间	面积	高度	高度%	面积%
1	10.566	6838720	377459	97.567	98.106
2	11.566	132047	9412	2.433	1.894
总计		6970767	386870	100.000	100.000

(1*S*,6*S*)-1-cyclohexyl-6-(difluoromethyl)-3-tosyl-3-azabicyclo[4.1.0]hept-4-ene (2g)

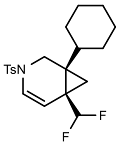

Colorless oil, 18 mg, 95% yield, Rf = 0.2 (PE: EtOAc = 10: 1). **^{1}H NMR** (500 MHz, Chloroform-*d*) δ 7.64 (d, J = 8.2 Hz, 2H), 7.33 (d, J = 8.0 Hz, 2H), 6.44 (d, J = 8.1 Hz, 1H), 5.70 – 5.38 (m, 2H), 3.81 (dd, J = 11.7, 2.6 Hz, 1H), 2.88 (d, J = 11.7 Hz, 1H), 2.43 (s, 3H), 1.83 (d, J = 10.1 Hz, 1H), 1.68 (t, J = 15.5 Hz, 4H), 1.40 – 1.30 (m, 1H), 1.18 (ddd, J = 28.1, 15.7, 6.6 Hz, 4H), 1.05 (d, J = 11.9 Hz, 1H), 0.93 – 0.85 (m, 1H), 0.80 (d, J = 5.0 Hz, 1H). **^{13}C NMR** (126 MHz, $CDCl_3$) δ 144.02, 134.87, 129.94, 126.92, 122.95, 115.62 (t, J = 239.2 Hz), 108.40 (t, J = 4.2 Hz), 42.07, 41.13, 39.68 (d, J = 6.8 Hz), 29.28, 26.87, 26.57, 26.17, 22.01, 21.98 (d, J = 5.5 Hz). **^{19}F NMR** (471 MHz, Chloroform-*d*) δ -107.37 (d, J = 285.1 Hz), -119.32 (d, J = 284.8 Hz). **HRMS**: Calculation for $C_{20}H_{25}F_2NO_2S$, $[M+Na]^+$, 404.1466 Found: 404.1461. HPLC: OD-H column, 97:3 hexane: isopropanol, 0.5 mL/min, t_R = major: 15.4 min, minor: 16.1 min. 97% ee. $[\alpha]_D^{29}$ = 62° (c 0.4, CH_2Cl_2).

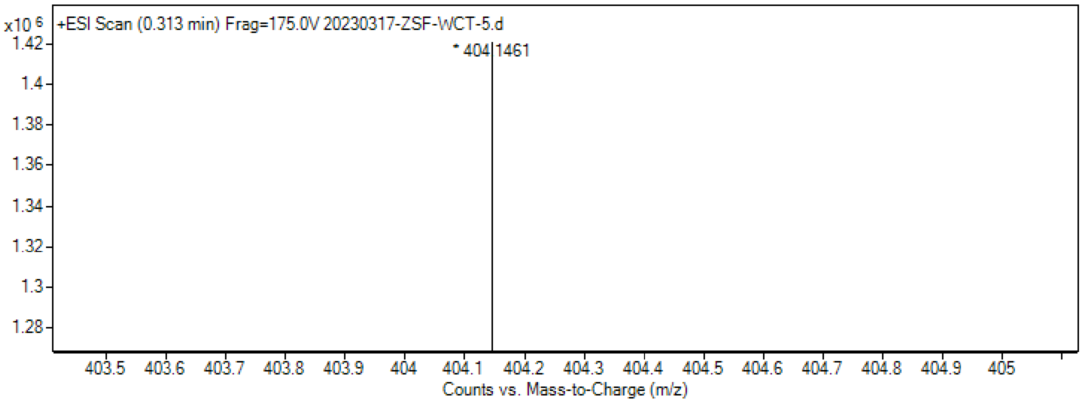

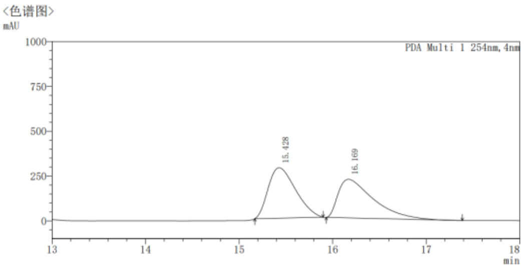

<峰表>
PDA Ch1 254nm

峰号	保留时间	面积	高度	高度%	面积%
1	15.428	5739797	281207	56.621	50.420
2	16.169	5644100	215441	43.379	49.580
总计		11383897	496648	100.000	100.000

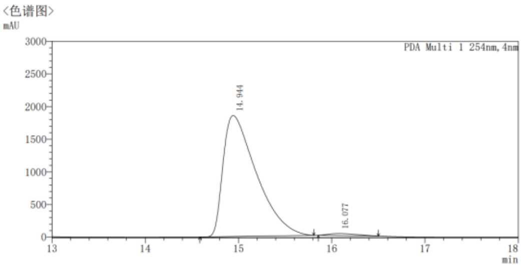

<峰表>
PDA Ch1 254nm

峰号	保留时间	面积	高度	高度%	面积%
1	14.944	45994428	1853326	98.433	98.625
2	16.077	641367	29501	1.567	1.375
总计		46635795	1882828	100.000	100.000

(1*S*,6*S*)-6-(difluoromethyl)-1-(fluoromethyl)-3-tosyl-3-azabicyclo[4.1.0]hept-4-ene (2h)

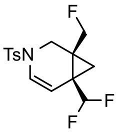

Colorless oil, 12 mg, 72% yield, Rf = 0.2 (PE: EtOAc = 15: 1). **^{1}H NMR** (500 MHz, Chloroform-*d*) δ 7.67 (d, *J* = 7.8 Hz, 2H), 7.34 (d, *J* = 7.9 Hz, 2H), 6.54 (d, *J* = 8.2 Hz, 1H), 5.72 (t, *J* = 56.0 Hz, 1H), 5.44 (d, *J* = 8.2 Hz, 1H), 4.52 (ddd, *J* = 72.1, 47.7, 10.6 Hz, 2H), 4.00 (d, *J* = 11.5 Hz, 1H), 3.01 (d, *J* = 11.5 Hz, 1H), 2.44 (s, 3H), 1.28 (d, *J* = 5.7 Hz, 1H), 1.17 (d, *J* = 4.4 Hz, 1H). **^{13}C NMR** (126 MHz, $CDCl_3$) δ 144.41, 134.35, 130.08, 127.09, 123.83, 115.58 (t, *J* = 241.3 Hz), 106.40(t, *J* = 5.04 Hz), 83.52 (d, *J* = 171.7 Hz), 42.33 (d, *J* = 2.5 Hz), 34.48 (d, *J* = 3.7 Hz), 34.30 (d, *J* = 4.4 Hz), 21.59, 20.02. **^{19}F NMR** (471 MHz, $CDCl_3$) δ -112.76 (d, *J* = 285.5 Hz), -118.09 (d, *J* = 285.5 Hz), -218.02. **HRMS**: Calculation for $C_{15}H_{16}F_3NO_2S$, $[M+H]^+$, 332.0927 Found: 332.0927. HPLC: INA column, 95:5 hexane: isopropanol, 0.50 mL/min, t_R = major: 20.4 min, minor: 22.9 min. 96% ee. $[\alpha]_D^{29}$ = 28° (c 0.8, CH_2Cl_2).

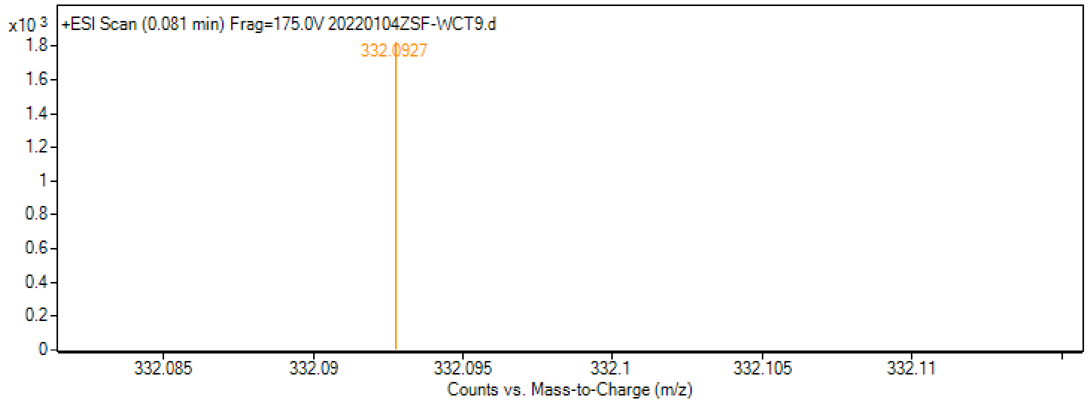

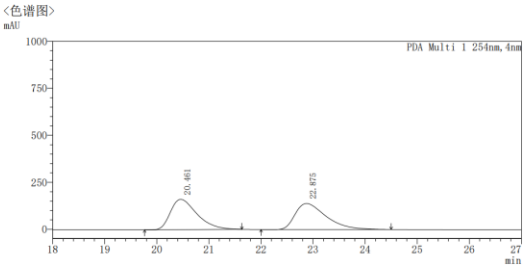

<峰表>
PDA Ch1 254nm

峰号	保留时间	面积	高度%	面积%
1	20.461	5743106	53.899	50.012
2	22.875	5740382	46.101	49.988
总计		11483488	100.000	100.000

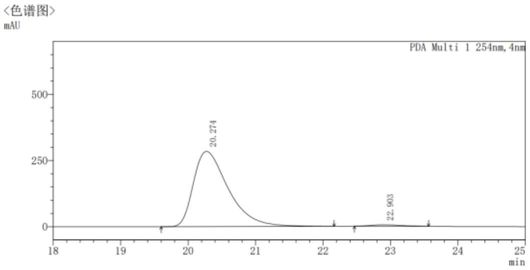

<峰表>
PDA Ch1 254nm

峰号	保留时间	面积	高度	高度%	面积%
1	20.274	10253702	284840	98.207	98.312
2	22.903	176044	5201	1.793	1.688
总计		10429745	290042	100.000	100.000

(1*R*,6*S*)-1-(3-chloropropyl)-6-(difluoromethyl)-3-tosyl-3-azabicyclo[4.1.0]hept-4-ene (2i)

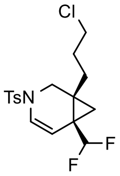

Colorless oil, 17 mg, 90% yield, Rf = 0.2 (PE: EtOAc = 10: 1). ^{1}**H NMR** (500 MHz, Chloroform-*d*) δ 7.65 (d, *J* = 8.1 Hz, 2H), 7.34 (d, *J* = 7.9 Hz, 2H), 6.47 (d, *J* = 8.1 Hz, 1H), 5.73 – 5.38 (m, 2H), 3.86 (dd, *J* = 11.6, 2.8 Hz, 1H), 3.53 (t, *J* = 6.2 Hz, 2H), 2.76 (d, *J* = 11.6 Hz, 1H), 2.44 (s, 3H), 1.91 – 1.83 (m, 2H), 1.74 (ddd, *J* = 15.1, 10.2, 5.0 Hz, 1H), 1.65 (ddd, *J* = 14.5, 10.7, 6.0 Hz, 1H), 1.01 (dd, *J* = 16.4, 4.9 Hz, 2H). ^{13}**C NMR** (126 MHz, $CDCl_3$) δ 144.24, 134.65, 130.03, 126.99, 123.13, 116.73 (t, *J* = 240.1 Hz), 107.49 (t, *J* = 4.4 Hz), 44.46, 44.05, 34.23 (d, *J* = 6.3 Hz), 29.33, 28.82, 25.50 (t, *J* = 25.9 Hz), 21.85 (d, *J* = 5.8 Hz), 21.58. ^{19}**F NMR** (471 MHz, Chloroform-*d*) δ -110.23 (d, *J* = 284.6 Hz), -119.63 (d, *J* = 284.7 Hz). **HRMS**: Calculation for $C_{17}H_{20}ClF_2NO_2S$, $[M+H]^+$, 376.0944 Found: 376.0937. HPLC: OD-H column, 99:1 hexane: isopropanol, 1.0 mL/min, t_R = major: 35.3 min, minor: 41.1 min. 96% ee. $[\alpha]_D^{29}$ = -32° (c 0.5, CH_2Cl_2).

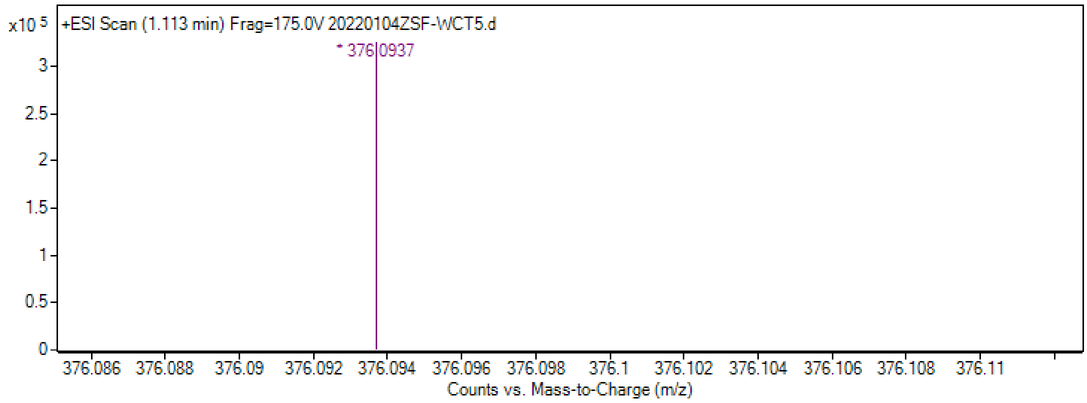

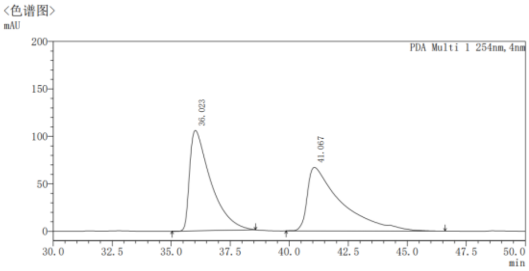

<峰表>

PDA Ch1 254nm

峰号	保留时间	面积	高度	高度%	面积%
1	36.023	6588748	105786	61.246	49.550
2	41.067	6708418	66937	38.754	50.450
总计		13297166	172723	100.000	100.000

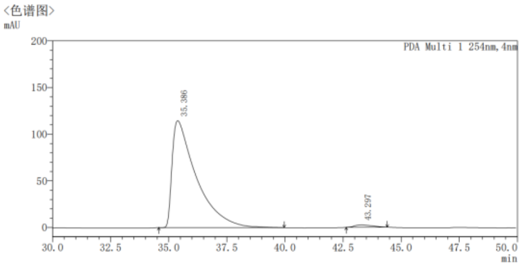

<峰表>

PDA Ch1 254nm

峰号	保留时间	面积	高度%	面积%
1	35.386	8348513	97.848	98.396
2	43.297	136114	2.152	1.604
总计		8484628	100.000	100.000

(1*R*,6*S*)-6-(difluoromethyl)-1-(non-8-en-1-yl)-3-tosyl-3-azabicyclo[4.1.0]hept-4-ene (2j)

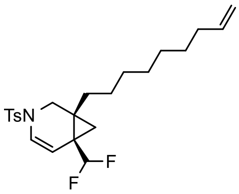

Colorless oil, 19 mg, 89% yield, Rf = 0.2 (PE: EtOAc = 10: 1). **^{1}H NMR** (500 MHz, Chloroform-*d*) δ 7.65 (d, *J* = 8.0 Hz, 2H), 7.33 (d, *J* = 7.9 Hz, 2H), 6.46 (d, *J* = 8.1 Hz, 1H), 5.81 (ddt, *J* = 16.9, 10.2, 6.7 Hz, 1H), 5.66 – 5.27 (m, 2H), 5.11 – 4.78 (m, 2H), 3.89 (dd, *J* = 11.6, 2.9 Hz, 1H), 2.76 (d, *J* = 11.6 Hz, 1H), 2.44 (s, 3H), 2.04 (t, *J* = 7.2 Hz, 2H), 1.41 – 1.29 (m, 5H), 1.27 (q, *J* = 6.8, 6.0 Hz, 7H), 0.97 (t, *J* = 4.7 Hz, 1H), 0.91 (d, *J* = 5.5 Hz, 1H). **^{13}C NMR** (126 MHz, $CDCl_3$) δ 144.05, 139.08, 134.81, 129.95, 127.00, 122.91, 116.88 (t, *J* = 239.6 Hz), 114.28, 107.67, 44.11, 35.06 (d, *J* = 5.8 Hz), 33.74, 31.72, 29.70, 29.24, 29.00, 28.83, 26.50, 25.95 – 24.46 (m), 22.04 (d, *J* = 5.6 Hz), 21.58. **^{19}F NMR** (471 MHz, Chloroform-*d*) δ -110.16 (d, *J* = 284.3 Hz), -119.71 (d, *J* = 284.5 Hz). **HRMS**: Calculation for $C_{23}H_{31}F_2NO_2S$, $[M+Na]^+$, 446.1936 Found: 446.1933. HPLC: INA column, 95:5 hexane: isopropanol, 1.0 mL/min, t_R = major: 11.4 min, minor: 10.4 min. 94% ee. $[\alpha]_D^{29}$ = 38° (c 0.3, CH_2Cl_2).

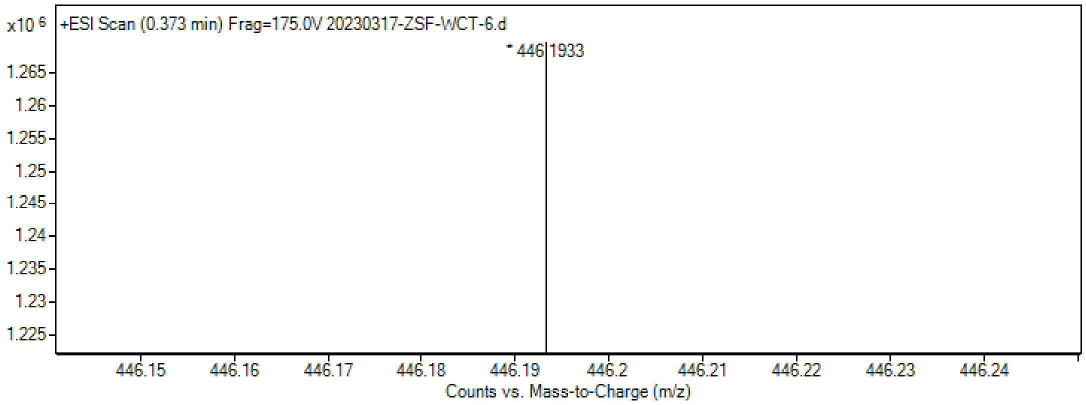

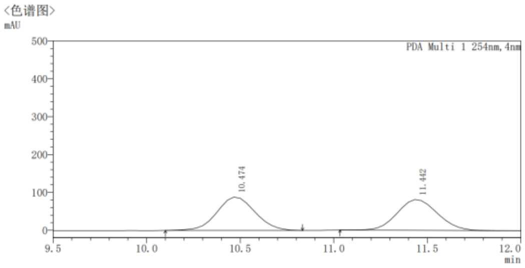

<峰表>
PDA Ch1 254nm

峰号	保留时间	面积	高度	高度%	面积%
1	10.474	1250118	88222	52.133	50.886
2	11.442	1206592	81003	47.867	49.114
总计		2456710	169225	100.000	100.000

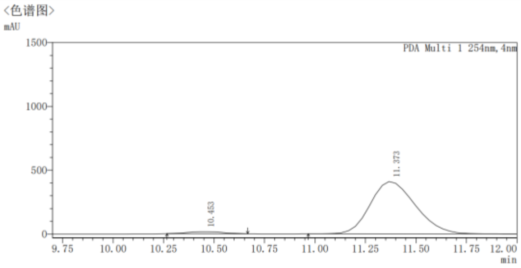

<峰表>
PDA Ch1 254nm

峰号	保留时间	面积	高度	高度%	面积%
1	10.453	193080	15631	3.673	2.939
2	11.373	6376621	409964	96.327	97.061
总计		6569701	425594	100.000	100.000

(1*S*,6*S*)-6-(difluoromethyl)-1-((*S*)-6-methylhept-5-en-2-yl)-3-tosyl-3-azabicyclo[4.1.0]hept-4-ene (2k)

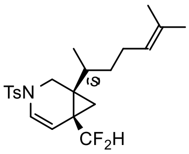

Colorless oil, 18 mg, 89% yield, Rf = 0.2 (PE: EtOAc = 10: 1). **^{1}H NMR** (500 MHz, Chloroform-*d*) δ 7.64 (d, *J* = 8.2 Hz, 2H), 7.33 (d, *J* = 7.9 Hz, 2H), 6.44 (d, *J* = 8.0 Hz, 1H), 5.82 – 5.30 (m, 2H), 5.01 (t, *J* = 7.3 Hz, 1H), 3.83 (dd, *J* = 11.7, 3.1 Hz, 1H), 2.81 (d, *J* = 11.6 Hz, 1H), 2.43 (s, 3H), 1.97 (t, *J* = 11.3 Hz, 1H), 1.85 (dq, *J* = 15.4, 7.9 Hz, 1H), 1.65 (s, 3H), 1.54 (s, 3H), 1.45 (q, *J* = 9.5, 9.1 Hz, 1H), 1.26 (ddd, *J* = 18.4, 8.3, 4.3 Hz, 2H), 1.08 (d, *J* = 6.3 Hz, 3H), 0.93 (dd, *J* = 5.3, 3.6 Hz, 1H), 0.80 (d, *J* = 5.4 Hz, 1H). **^{13}C NMR** (126 MHz, $CDCl_3$) δ 144.04, 134.85, 132.63, 129.97, 126.94, 123.45, 122.97, 116.52 (t, *J* = 239.3 Hz), 108.33 (t, *J* = 4.0 Hz), 40.85 (d, *J* = 7.0 Hz), 40.41, 35.44, 33.47, 27.64 – 26.52 (m), 25.99, 25.66, 22.09 (d, *J* = 5.1 Hz), 21.58, 17.59, 16.68. **^{19}F NMR** (471 MHz, Chloroform-*d*) δ -107.66 (d, *J* = 284.8 Hz), -119.18 (d, *J* = 284.8 Hz). **HRMS**: Calculation for $C_{22}H_{29}F_2NO_2S$, $[M+Na]^+$, 432.1770 Found: 432.1770. HPLC: INC column, 99:1 hexane: isopropanol, 1.0 mL/min, t_R = major: 22.7 min, minor: 20.6 min. 96% ee. $[\alpha]_D^{29}$ = 19° (c 0.5, CH_2Cl_2).

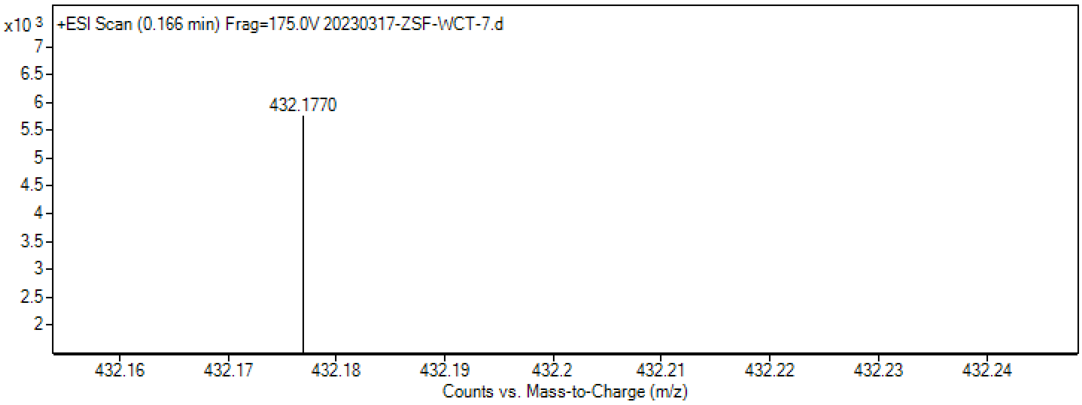

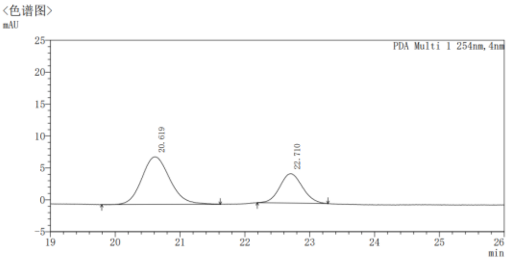

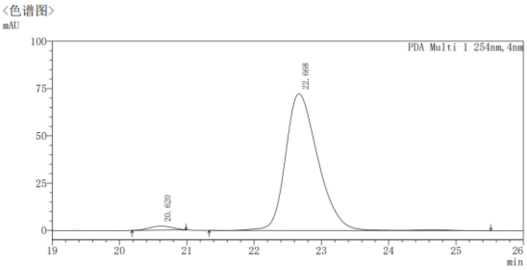

<峰表>
PDA Ch1 254nm

峰号	保留时间	面积	高度	面积%	峰结束
1	20.619	223575	7455	66.597	21.621
2	22.710	112138	4613	33.403	23.280
总计		335713	12069	100.000	

<峰表>
PDA Ch1 254nm

峰号	保留时间	面积	高度	浓度	面积%	高度%
1	20.620	50610	2144	0.000	2.049	2.880
2	22.668	2419270	72290	0.000	97.951	97.120
总计		2469880	74433		100.000	100.000

(1*S*,6*S*)-1-(((tert-butyldimethylsilyl)oxy)methyl)-6-(difluoromethyl)-3-tosyl-3-azabicyclo[4.1.0]hept-4-ene (2l)

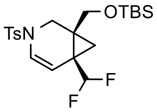

Yellow solid, 19 mg, 83% yield, m.p. = 81.7 - 81.9 ºC, Rf = 0.2 (PE: EtOAc = 10: 1). **^{1}H NMR** (500 MHz, Chloroform-*d*) δ 7.62 (d, *J* = 7.9 Hz, 2H), 7.30 (d, *J* = 7.9 Hz, 2H), 6.47 (d, *J* = 8.2 Hz, 1H), 5.66 (t, *J* = 55.9 Hz, 1H), 5.48 (d, *J* = 8.2 Hz, 1H), 3.85 (dd, *J* = 11.7, 2.5 Hz, 1H), 3.75 (d, *J* = 11.1 Hz, 1H), 3.61 (d, *J* = 11.0 Hz, 1H), 2.86 (d, *J* = 11.5 Hz, 1H), 2.40 (s, 3H), 1.17 (d, *J* = 5.3 Hz, 1H), 0.92 (t, *J* = 4.5 Hz, 1H), 0.83 (s, 9H), -0.00 (d, *J* = 3.1 Hz, 6H). **^{13}C NMR** (126 MHz, $CDCl_3$) δ 144.11, 134.52, 129.92, 127.08, 123.05, 115.91 (t, *J* = 239.8 Hz), 107.12 (t, *J* = 4.8 Hz), 62.76, 42.88, 35.84, 25.71, 24.86 (t, *J* = 25.9 Hz), 21.55, 19.83 (d, *J* = 5.8 Hz), 18.11, -5.64 (d, *J* = 3.6 Hz). **^{19}F NMR** (471 MHz, Chloroform-*d*) δ -111.37 (d, *J* = 284.4 Hz), -118.24 (d, *J* = 284.3 Hz). **HRMS**: Calculation for $C_{21}H_{31}F_2NO_3SSi$, $[M+H]^+$, 444.1835 Found:444.1832. HPLC: INC column, 90:10 hexane: isopropanol, 1.0 mL/min, t_R = major: 21.1 min, minor: 18.0 min. 96% ee. $[\alpha]_D^{29}$ = 36° (c 0.9, CH_2Cl_2).

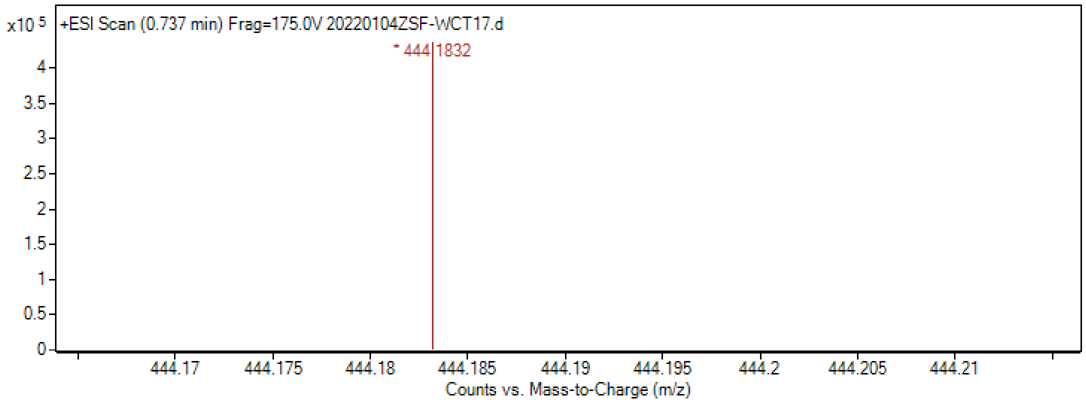

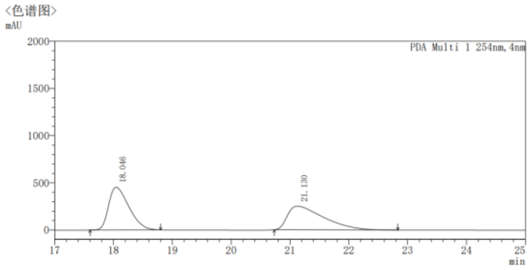

<峰表>
PDA Ch1 254nm

峰号	保留时间	面积	高度	高度%	面积%
1	18.046	10865119	452422	64.270	50.409
2	21.130	10688631	251514	35.730	49.591
总计		21553751	703936	100.000	100.000

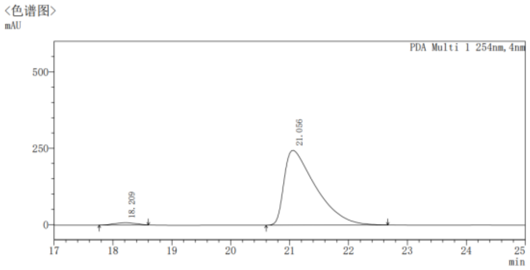

<峰表>
PDA Ch1 254nm

峰号	保留时间	面积	高度	高度%	面积%
1	18.209	197124	7519	2.976	2.108
2	21.056	9156271	245145	97.024	97.892
总计		9353395	252664	100.000	100.000

(1*R*,6*S*)-1-benzyl-6-(difluoromethyl)-3-tosyl-3-azabicyclo[4.1.0]hept-4-ene (2m)

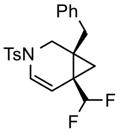

Colorless oil, 12 mg, 62% yield, Rf = 0.2 (PE: EtOAc = 10: 1). **^{1}H NMR** (500 MHz, Chloroform-*d*) δ 7.44 (d, *J* = 8.2 Hz, 2H), 7.31 – 7.22 (m, 2H), 7.22 – 7.17 (m, 3H), 7.10 – 7.05 (m, 2H), 6.40 (d, *J* = 8.1 Hz, 1H), 5.60 (t, *J* = 55.4 Hz, 1H), 5.46 (d, *J* = 8.1 Hz, 1H), 3.60 (dd, *J* = 12.1, 2.7 Hz, 1H), 2.88 (d, *J* = 14.9 Hz, 1H), 2.67 (dd, *J* = 13.6, 4.5 Hz, 2H), 2.35 (s, 3H), 1.16 (d, *J* = 5.5 Hz, 1H), 0.87 (t, *J* = 4.7 Hz, 1H). **^{13}C NMR** (126 MHz, $CDCl_3$) δ 144.06, 137.25, 134.68, 129.89, 128.79, 128.58, 127.09, 126.96, 123.34 115.80 (t, *J* = 240.3 Hz), 107.69 (t, *J* = 4.7 Hz), 44.28, 37.11, 35.34 (d, *J* = 5.4 Hz), 25.11 (t, *J* = 25.7 Hz), 22.34 (d, *J* = 6.2 Hz), 21.56. **^{19}F NMR** (471 MHz, Chloroform-*d*) δ -110.86 (d, *J* = 284.5 Hz), -119.12 (d, *J* = 285.5 Hz). **HRMS**: Calculation for $C_{21}H_{21}F_2NO_2S$, $[M+H]^+$, 390.1334 Found: 390.1332. HPLC: whelk column, 95:5 hexane: isopropanol, 1.0 mL/min, t_R = major: 25.9 min, minor: 30.3 min. 92% ee. $[\alpha]_D^{29}$ = 56° (c 0.7, CH_2Cl_2).

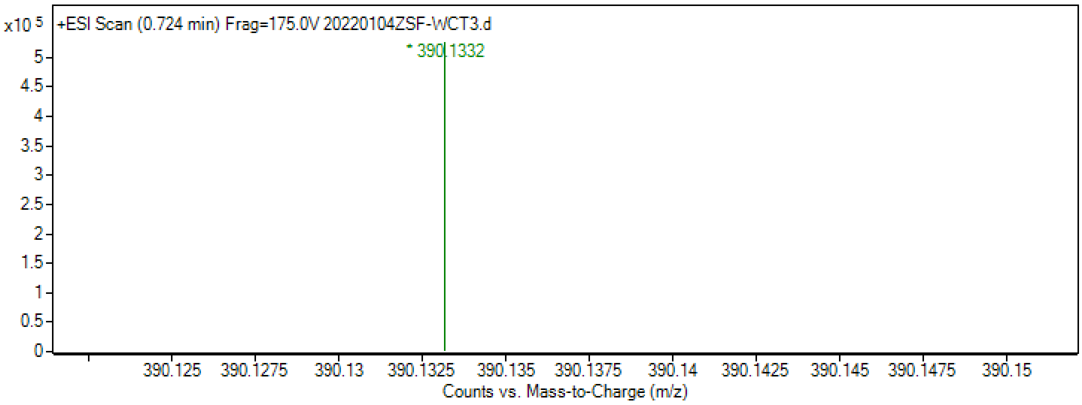

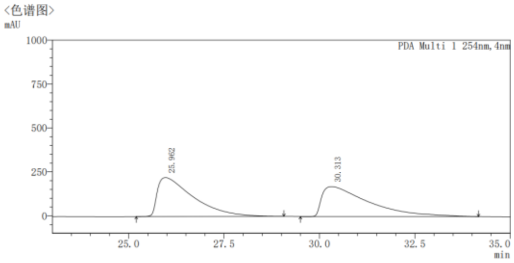

<峰表>
PDA Ch1 254nm

峰号	保留时间	面积	高度	高度%	面积%
1	25.962	14550611	221925	56.581	50.014
2	30.313	14542274	170301	43.419	49.986
总计		29092885	392226	100.000	100.000

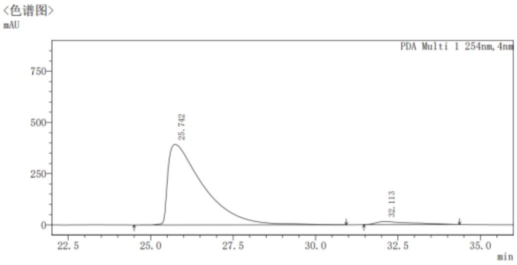

<峰表>
PDA Ch1 254nm

峰号	保留时间	面积	高度	高度%	面积%
1	25.742	29276596	393112	96.596	96.386
2	32.113	1097774	13851	3.404	3.614
总计		30374370	406963	100.000	100.000

(1*S*,6*S*)-6-(difluoromethyl)-1-((5-methylfuran-2-yl)methyl)-3-tosyl-3-azabicyclo[4.1.0]hept-4-ene (2n)

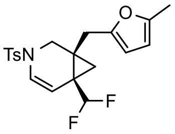

Yellow solid, 18 mg, 86% yield, m.p. = 76.5 - 76.7 ºC, Rf = 0.2 (PE: EtOAc = 10: 1). **^{1}H NMR** (500 MHz, Chloroform-*d*) δ 7.61 (d, *J* = 8.2 Hz, 2H), 7.31 (d, *J* = 8.0 Hz, 2H), 6.47 (d, *J* = 8.1 Hz, 1H), 5.92 (d, *J* = 3.0 Hz, 1H), 5.87 (s, 1H), 5.67 (t, *J* = 55.7 Hz, 1H), 5.49 (d, *J* = 8.1 Hz, 1H), 3.86 (d, *J* = 11.9 Hz, 1H), 2.91 – 2.81 (m, 2H), 2.74 (d, *J* = 15.9 Hz, 1H), 2.43 (s, 3H), 2.25 (s, 3H), 1.15 (d, *J* = 5.5 Hz, 1H), 0.94 (t, *J* = 4.6 Hz, 1H). **^{13}C NMR** (126 MHz, $CDCl_3$) δ 151.35, 149.33, 144.06, 134.68, 129.90, 127.00, 123.18, 116.44 (t, *J* = 240.6 Hz), 107.98, 107.63 (t, *J* = 4.7 Hz), 106.16, 44.39, 34.14 (d, *J* = 4.9 Hz), 30.06, 25.26 (t, *J* = 25.8 Hz), 21.67 (d, *J* = 5.4 Hz), 21.57, 13.54. **^{19}F NMR** (471 MHz, Chloroform-*d*) δ -111.49 (d, *J* = 284.4 Hz), -119.03 (d, *J* = 284.4 Hz). **HRMS**: Calculation for $C_{20}H_{21}F_2NO_3S$, $[M+H]^+$, 416.1102 Found:416.1092. HPLC: OD-H column, 95:5 hexane: isopropanol, 1.0 mL/min, t_R = major: 16.6 min, minor: 14.9 min. 95% ee. $[\alpha]_D^{29}$ = 27° (c 0.6, CH_2Cl_2).

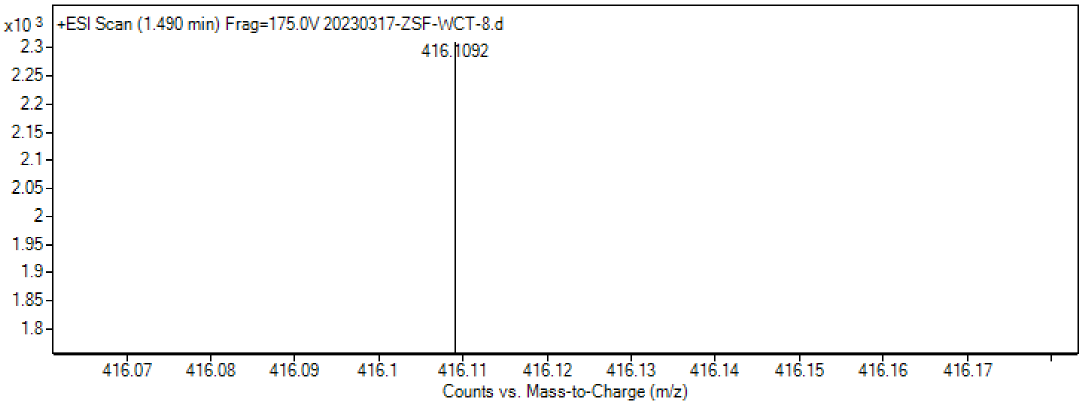

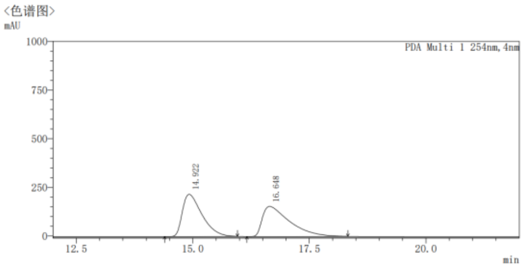

<峰表>
PDA Ch1 254nm

峰号	保留时间	面积	高度	高度%	面积%
1	14.922	6321687	216715	58.412	50.302
2	16.648	6245680	154297	41.588	49.698
总计		12567367	371012	100.000	100.000

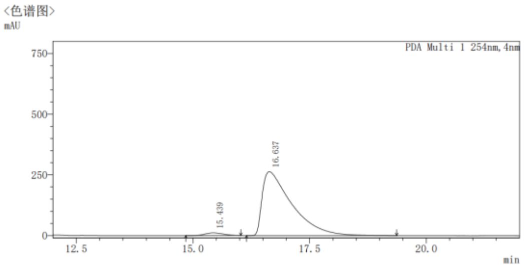

<峰表>
PDA Ch1 254nm

峰号	保留时间	面积	高度	高度%	面积%
1	15.439	297177	11067	4.037	2.492
2	16.637	11627383	263085	95.963	97.508
总计		11924560	274152	100.000	100.000

(1*S*,6*S*)-6-(difluoromethyl)-1-phenyl-3-tosyl-3-azabicyclo[4.1.0]hept-4-ene (2o)

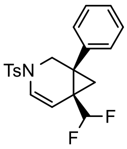

Yellow solid, 19 mg, 98% yield, m.p.. = 85.0 - 85.2 ºC, Rf = 0.2 (PE: EtOAc = 10: 1). **^{1}H NMR** (500 MHz, $CDCl_3$) δ 7.65 (d, J = 8.4 Hz, 2H), 7.31 (qd, J = 7.7, 3.7 Hz, 7H), 6.58 (d, J = 8.2 Hz, 1H), 5.69 (d, J = 8.2 Hz, 1H), 4.82 (t, J = 53.7 Hz, 1H), 3.99 (dd, J = 11.8, 3.4 Hz, 1H), 3.01 (d, J = 11.8 Hz, 1H), 2.44 (s, 3H), 1.48 (d, J = 3.2 Hz, 2H). **^{13}C NMR** (126 MHz, $CDCl_3$) δ 144.19, 135.99, 134.71, 130.02, 129.66, 129.05, 128.33, 127.08, 123.66, 117.00 (d, J = 239.9 Hz), 107.35 (t, J = 3.6, 0.6 Hz), 47.23, 40.33 (d, J = 8.1 Hz), 26.24 (t, J = 1.3, 0.4 Hz), 21.59, 19.52 (d, J = 5.9 Hz). **^{19}F NMR** (471 MHz, $CDCl_3$) δ -113.65 (d, J = 284.2 Hz), -120.81 (d, J = 282.7 Hz). **HRMS**: Calculation for $C_{20}H_{19}F_2NO_2S$, $[M+H]^+$, 376.1177 Found: 376.1166. HPLC: INA column, 99:1 hexane: isopropanol, 1.0 mL/min, t_R = major: 11.5 min, minor: 10.2 min. 93% ee. $[\alpha]_D^{29}$ = 90° (c 0.3, CH_2Cl_2).

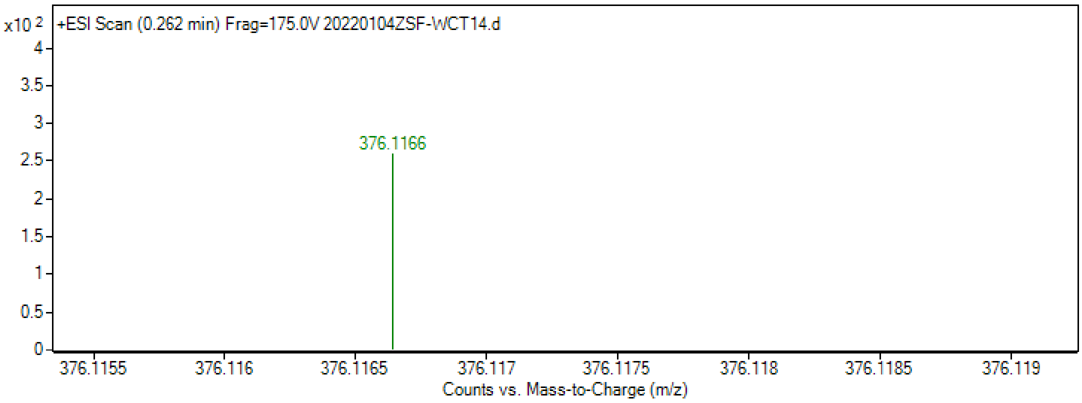

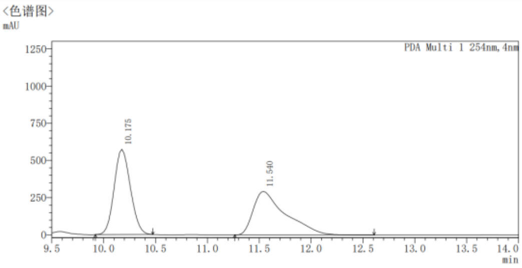

<峰表>
PDA Ch1 254nm

峰号	保留时间	面积	高度	高度%	面积%
1	10.175	6242785	572938	66.228	49.866
2	11.540	6276319	292155	33.772	50.134
总计		12519105	865093	100.000	100.000

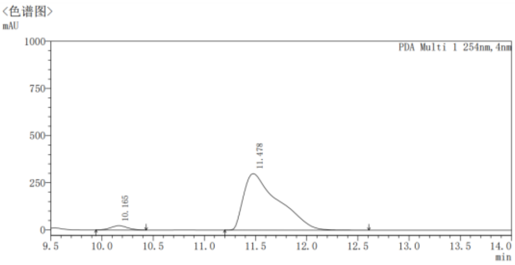

<峰表>
PDA Ch1 254nm

峰号	保留时间	面积	高度	高度%	面积%
1	10.165	250918	22321	6.918	3.428
2	11.478	7068174	300334	93.082	96.572
总计		7319092	322655	100.000	100.000

(1*S*,6*S*)-1-([1,1'-biphenyl]-4-yl)-6-(difluoromethyl)-3-tosyl-3-azabicyclo[4.1.0]hept-4-ene (2p)

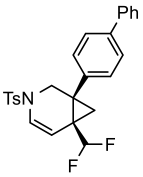

Yellow solid, 21 mg, 94% yield, m.p. = 130.0 - 130.3 °C, Rf = 0.2 (PE: EtOAc = 10: 1). **^{1}H NMR** (500 MHz, Chloroform-*d*) δ 7.66 (d, *J* = 8.2 Hz, 2H), 7.55 (d, *J* = 8.1 Hz, 4H), 7.44 (t, *J* = 7.6 Hz, 2H), 7.35 (dd, *J* = 15.5, 8.1 Hz, 5H), 6.60 (d, *J* = 8.2 Hz, 1H), 5.71 (d, *J* = 8.2 Hz, 1H), 4.90 (t, *J* = 54.4 Hz, 1H), 4.02 (dd, *J* = 11.7, 2.9 Hz, 1H), 3.04 (d, *J* = 11.8 Hz, 1H), 2.44 (s, 3H), 1.52 (dd, *J* = 8.3, 4.6 Hz, 2H). **^{13}C NMR** (126 MHz, $CDCl_3$) δ 144.20, 141.31, 140.23, 134.91, 134.72, 130.07, 130.02, 128.88, 127.73, 127.65, 127.10, 127.07, 123.71, 116.09 (t, *J* = 239.7 Hz), 107.30, 47.19, 40.00 (d, *J* = 8.0 Hz), 26.35 (t, *J* = 54.2 Hz), 21.60, 19.60 (d, *J* = 5.6 Hz). **^{19}F NMR** (471 MHz, Chloroform-*d*) δ -113.52 (d, *J* = 283.7 Hz), -120.80 (d, *J* = 283.7 Hz). **HRMS**: Calculation for $C_{26}H_{23}F_2NO_2S$, $[M+H]^+$, 452.1490 Found: 452.1482. HPLC: OD-H column, 95:5 hexane: isopropanol, 1.0 mL/min, t_R = major: 23.6 min, minor: 20.3 min. 91% ee. $[\alpha]_D^{29}$ = 88° (c 0.4, CH_2Cl_2).

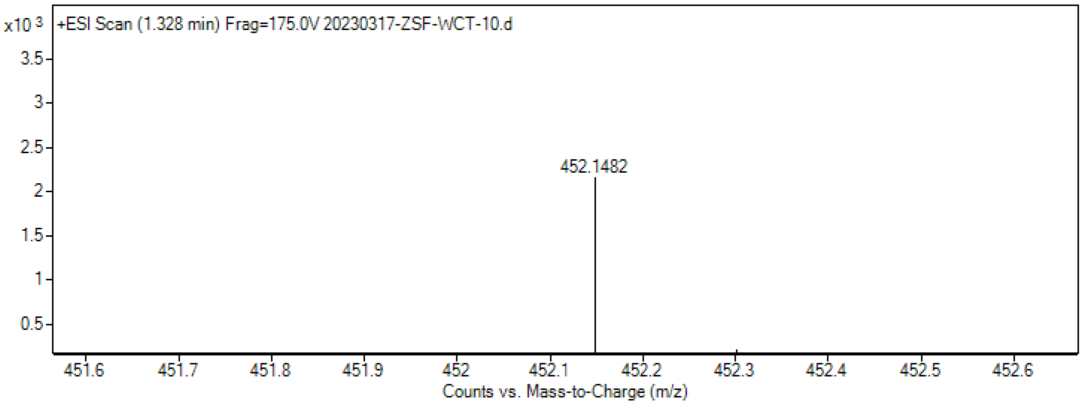

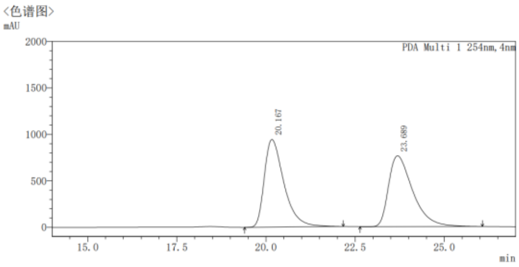

<峰表>
PDA Ch1 254nm

峰号	保留时间	面积	高度	高度%	面积%
1	20.167	36226844	944589	55.321	50.164
2	23.689	35990339	762882	44.679	49.836
总计		72217183	1707471	100.000	100.000

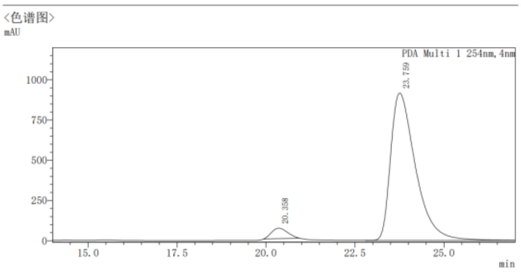

<峰表>
PDA Ch1 254nm

峰号	保留时间	面积	高度	高度%	面积%
1	20.358	2033502	64997	6.618	4.349
2	23.759	44722508	917131	93.382	95.651
总计		46756009	982129	100.000	100.000

(1*S*,6*S*)-6-(difluoromethyl)-1-(p-tolyl)-3-tosyl-3-azabicyclo[4.1.0]hept-4-ene (2q)

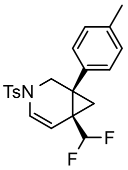

Yellow solid, 19 mg, 96% yield, m.p. = 95.3 - 95.5 ºC, Rf = 0.2 (PE: EtOAc = 10: 1). ^{1}**H NMR** (500 MHz, Chloroform-*d*) δ 7.66 – 7.61 (m, 2H), 7.32 (d, *J* = 8.0 Hz, 2H), 7.18 (d, *J* = 7.9 Hz, 2H), 7.13 (d, *J* = 7.9 Hz, 2H), 6.56 (d, *J* = 8.2 Hz, 1H), 5.68 (d, *J* = 8.2 Hz, 1H), 4.82 (t, *J* = 54.5, 54.0 Hz, 1H), 3.96 (dd, *J* = 11.8, 3.4 Hz, 1H), 2.98 (d, *J* = 11.8 Hz, 1H), 2.44 (s, 3H), 2.32 (s, 3H), 1.49 – 1.40 (m, 2H). ^{13}**C NMR** (126 MHz, $CDCl_3$) δ 144.14, 138.20, 134.72, 132.94, 129.99, 129.70, 129.50, 127.08, 123.55, 117.10 (t, *J* = 239.1 Hz), 107.38 (t, *J* = 3.2 Hz), 47.23, 39.99 (d, *J* = 8.2 Hz), 26.20 (t, *J* = 27.5, 24.8 Hz), 21.59, 21.11, 19.53 (d, *J* = 6.1 Hz). ^{19}**F NMR** (471 MHz, $CDCl_3$) δ -113.58 (d, *J* = 282.8 Hz), -120.94 (d, *J* = 282.9 Hz). **HRMS**: Calculation for $C_{21}H_{21}F_2NO_2S$ $[M+H]^+$, 390.1334 Found: 390.1324. HPLC: INA column, 99:1 hexane: isopropanol, 1.0 mL/min, t_R = major: 21.6 min, minor: 19.3 min. 93% ee. $[\alpha]_D^{29}$ = 19° (c 1.3, CH_2Cl_2).

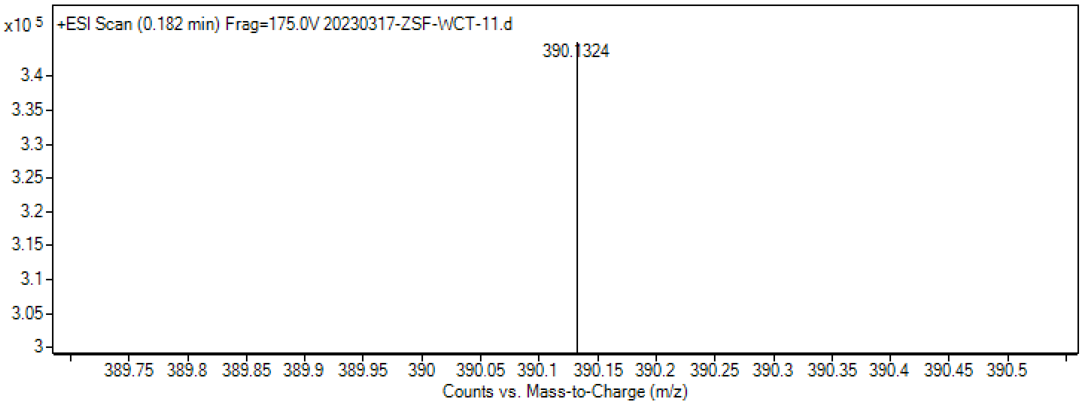

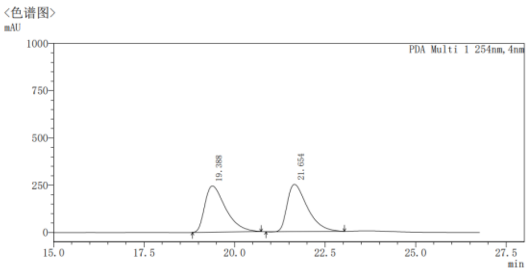

<峰表>
PDA Ch1 254nm

峰号	保留时间	面积	高度	高度%	面积%
1	19.388	9771829	244481	49.454	50.275
2	21.654	9664996	249883	50.546	49.725
总计		19436825	494364	100.000	100.000

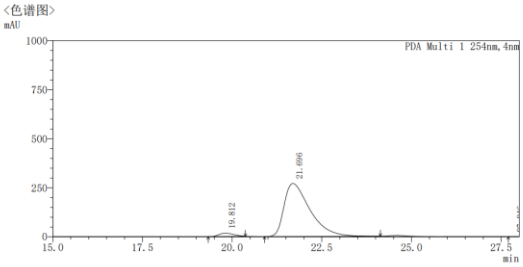

<峰表>
PDA Ch1 254nm

峰号	保留时间	面积	高度	面积%	高度%
1	19.812	498403	17321	3.545	5.998
2	21.696	13558685	271207	96.431	93.916
3	27.846	3463	248	0.025	0.086
总计		14060551	288776	100.000	100.000

(1*S*,6*S*)-6-(difluoromethyl)-1-(4-methoxyphenyl)-3-tosyl-3-azabicyclo[4.1.0]hept-4-ene (2r)

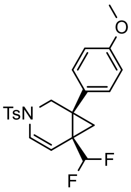

Yellow solid, 19 mg, 92% yield, m.p. = 80.0 - 80.3 ºC, Rf = 0.2 (PE: EtOAc = 10: 1). ^{1}**H NMR** (500 MHz, Chloroform-*d*) δ 7.64 (d, *J* = 8.1 Hz, 2H), 7.33 (d, *J* = 8.0 Hz, 2H), 7.21 (d, *J* = 8.4 Hz, 2H), 6.85 (d, *J* = 8.6 Hz, 2H), 6.56 (d, *J* = 8.1 Hz, 1H), 5.68 (d, *J* = 8.2 Hz, 1H), 4.83 (t, *J* = 54.5 Hz, 1H), 3.96 (dd, *J* = 11.8, 3.3 Hz, 1H), 3.79 (s, 3H), 2.96 (d, *J* = 11.7 Hz, 1H), 2.44 (s, 3H), 1.45 (d, *J* = 4.5 Hz, 1H), 1.42 (d, *J* = 5.8 Hz, 1H). ^{13}**C NMR** (126 MHz,

CDCl3) δ 159.44, 144.14, 134.74, 130.79, 129.99, 127.89, 127.07, 123.50, 117.16 (t, J = 239.1 Hz), 114.38, 107.35, 55.32, 47.25, 39.64 (d, J = 8.0 Hz), 26.15 (d, J = 27.6 Hz), 21.58, 19.69 (d, J = 5.8 Hz). **^{19}F NMR** (471 MHz, Chloroform-*d*) δ -113.47 (d, J = 283.2 Hz), -121.10 (d, J = 283.5 Hz). **HRMS**: Calculation for $C_{21}H_{21}F_2NO_3S$ $[M+H]^+$, 406.1283 Found: 406.1276. HPLC: OD-H column, 95:5 hexane: isopropanol, 0.80 mL/min, t_R = major: 11.7 min, minor: 12.5 min. 96% ee. $[\alpha]_D^{29}$ = 71° (c 0.4, CH_2Cl_2).

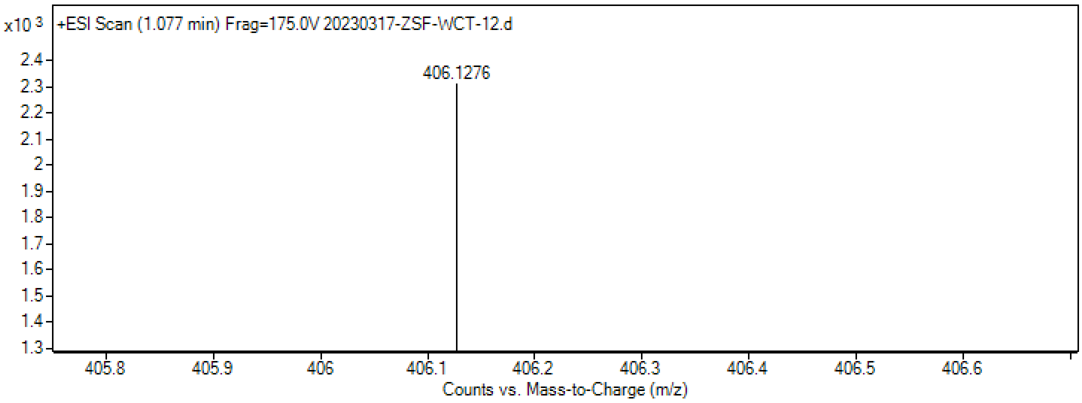

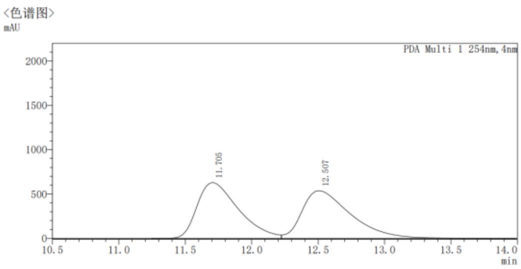

<峰表>
PDA Ch1 254nm

峰号	保留时间	面积	高度	高度%	面积%
1	11.705	13637587	628724	53.890	49.962
2	12.507	13658364	537954	46.110	50.038
总计		27295952	1166677	100.000	100.000

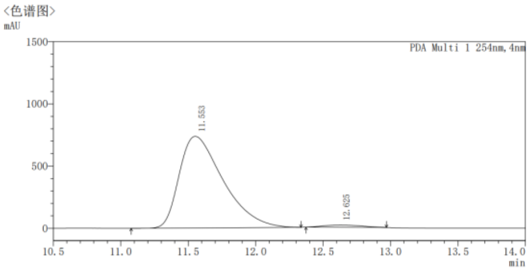

<峰表>
PDA Ch1 254nm

峰号	保留时间	面积	高度	高度%	面积%
1	11.553	16613980	736102	97.562	97.881
2	12.625	359634	18393	2.438	2.119
总计		16973614	754495	100.000	100.000

(1*S*,6*S*)-6-(difluoromethyl)-1-(4-fluorophenyl)-3-tosyl-3-azabicyclo[4.1.0]hept-4-ene (2s)

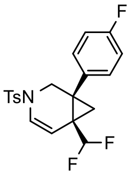

Yellow solid, 17 mg, 85% yield, m.p. = 45.3 - 45.5 ºC, Rf = 0.2 (PE: EtOAc = 10: 1). **^{1}H NMR** (500 MHz, Chloroform-*d*) δ 7.65 (d, J = 8.2 Hz, 2H), 7.34 (d, J = 8.0 Hz, 2H), 7.28 (dd, J = 8.3, 5.5 Hz, 2H), 7.02 (t, J = 8.6 Hz, 2H), 6.58 (d, J = 8.2 Hz, 1H), 5.67 (d, J = 8.2 Hz, 1H), 4.84 (t, J = 54.4 Hz, 1H), 3.96 (dd, J = 11.8, 3.0 Hz, 1H), 2.96 (d, J = 11.8 Hz, 1H), 2.44 (s, 3H), 1.51 – 1.46 (m, 1H), 1.44 (d, J = 5.7 Hz, 1H). **^{13}C NMR** (126 MHz, CDCl3) δ 162.42 (d, J = 248.2 Hz), 144.26, 134.66, 131.83 (d, J = 3.0 Hz), 131.42 (d, J = 8.2 Hz), 127.05, 123.75, 116.90 (t, J = 239.4 Hz), 116.04 (d, J = 21.8 Hz), 107.15, 47.23, 39.52 (d, J = 7.7 Hz), 39.52 (d, J = 7.7 Hz), 21.58, 19.67 (d, J = 5.8 Hz). **^{19}F NMR** (471 MHz, CDCl3) δ -112.90, 113.69 (d, J = 284.3 Hz), -120.91 (d, J = 284.1 Hz). **HRMS**: Calculation for $C_{20}H_{18}F_3NO_2S$ $[M+H]^+$, 394.1083 Found: 394.1076. HPLC: OD-H column, 99.5:0.5 hexane: isopropanol, 1.0 mL/min, t_R = major: 54.2 min, minor: 47.6 min. 94% ee. $[\alpha]_D^{29}$ = 14° (c 0.7, CH_2Cl_2).

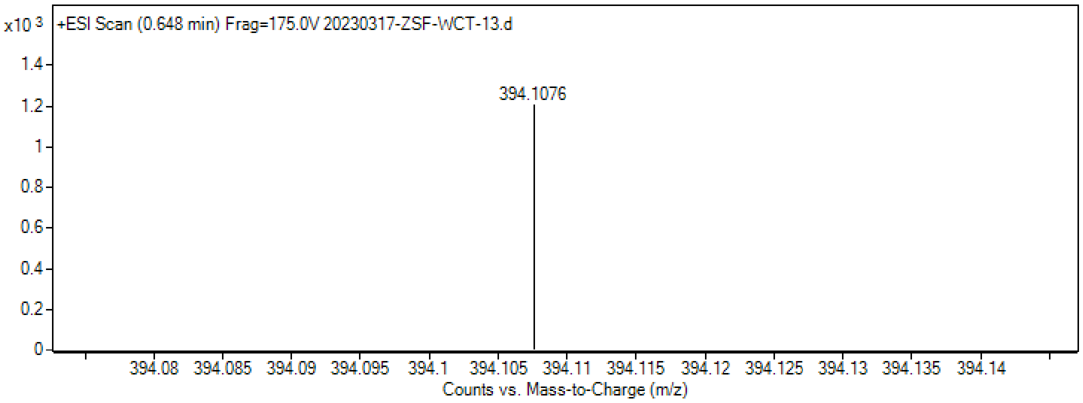

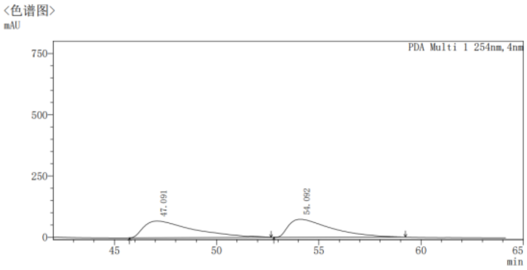

<峰表>
PDA Ch1 254nm

峰号	保留时间	面积	高度	高度%	面积%
1	47.091	11462013	68185	48.190	51.851
2	54.092	10643619	73309	51.810	48.149
总计		22105632	141494	100.000	100.000

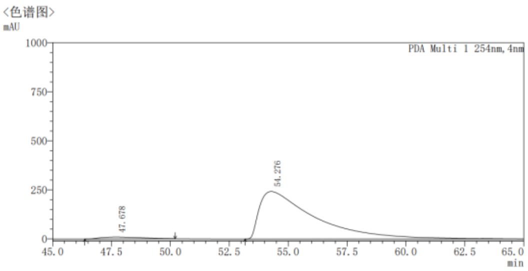

<峰表>
PDA Ch1 254nm

峰号	保留时间	面积	高度	高度%	面积%
1	47.678	1136532	9780	3.880	2.780
2	54.276	39751603	242276	96.120	97.220
总计		40888135	252057	100.000	100.000

(1*S*,6*S*)-1-(4-chlorophenyl)-6-(difluoromethyl)-3-tosyl-3-azabicyclo[4.1.0]hept-4-ene (2t)

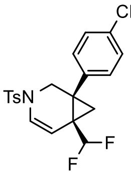

Yellow solid, 18 mg, 88% yield, m.p. = 95.0 - 95.5 ºC, Rf = 0.2 (PE: EtOAc = 10: 1). **^{1}H NMR** (500 MHz, Chloroform-*d*) δ 7.57 (d, *J* = 8.3 Hz, 2H), 7.29 – 7.23 (m, 4H), 7.20 – 7.09 (m, 2H), 6.50 (d, *J* = 8.2 Hz, 1H), 5.59 (d, *J* = 8.2 Hz, 1H), 4.76 (t, *J* = 54.4 Hz, 1H), 3.88 (dd, *J* = 11.8, 2.8 Hz, 1H), 2.88 (d, *J* = 11.8 Hz, 1H), 2.37 (s, 3H), 1.44 – 1.39 (m, 1H), 1.37 (d, *J* = 5.6 Hz, 1H). **^{13}C NMR** (126 MHz, $CDCl_3$) δ 144.29, 134.56 (d, *J* = 11.7 Hz), 134.32, 131.04, 130.05, 129.29, 127.06, 123.84, δ 116.81 (t, *J* = 239.6 Hz), 107.07, 47.10, 39.54 (d, *J* = 7.6 Hz), 26.33 (t, *J* = 27.1 Hz), 21.59, 19.54 (d, *J* = 5.9 Hz). **^{19}F NMR** (471 MHz, Chloroform-*d*) δ -113.73 (d, *J* = 284.3 Hz), -120.76 (d, *J* = 284.3 Hz). **HRMS**: Calculation for $C_{20}H_{18}ClF_2NO_2S$ $[M+H]^+$, 410.0788 Found: 410.0782. HPLC: INA column, 99:1 hexane: isopropanol, 1.0 mL/min, t_R = major: 28.4 min, minor: 26.1 min. 95% ee. $[\alpha]_D^{29}$ = 33° (c 0.6, CH_2Cl_2).

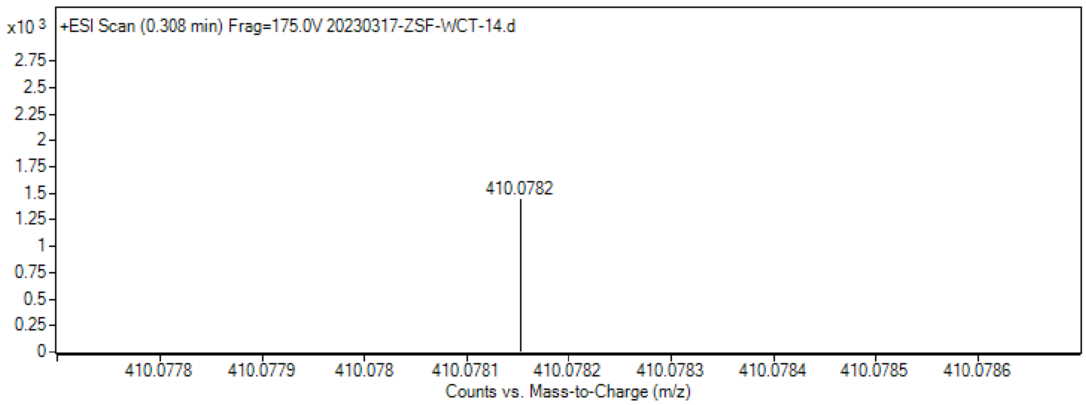

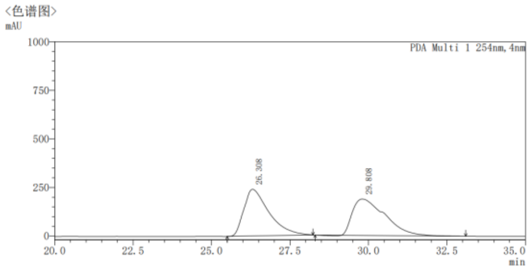

<峰表>
PDA Ch1 254nm

峰号	保留时间	面积	高度	面积%	高度%
1	26.308	13399031	239963	49.680	56.147
2	29.808	13571874	187424	50.320	43.853
总计		26970905	427388	100.000	100.000

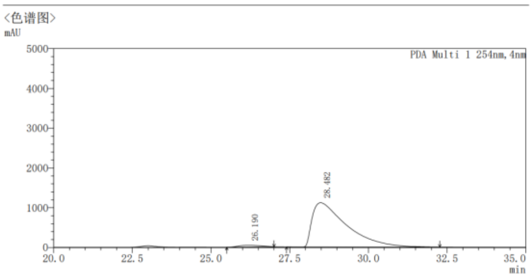

<峰表>
PDA Ch1 254nm

峰号	保留时间	面积	高度	高度%	面积%
1	26.190	2161333	44058	3.810	2.487
2	28.482	84728367	1112200	96.190	97.513
总计		86889700	1156258	100.000	100.000

(1*S*,6*S*)-1-(4-bromophenyl)-6-(difluoromethyl)-3-tosyl-3-azabicyclo[4.1.0]hept-4-ene (2u)

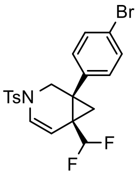

Yellow solid, 22 mg, 94% yield, m.p. = 74.3 – 74.5 °C, Rf = 0.2 (PE: EtOAc = 10: 1). **^{1}H NMR** (500 MHz, Chloroform-d) δ 7.64 (d, J = 8.0 Hz, 2H), 7.46 (d, *J* = 8.1 Hz, 2H), 7.33 (d, *J* = 8.0 Hz, 2H), 7.18 (d, *J* = 8.1 Hz, 2H), 6.58 (d, *J* = 8.2 Hz, 1H), 5.66 (d, *J* = 8.2 Hz, 1H), 4.84 (t, *J* = 54.4 Hz, 1H), 3.95 (dd, *J* = 11.7, 3.1 Hz, 1H), 2.95 (d, *J* = 11.8 Hz, 1H), 2.44 (s, 3H), 1.50 (t, *J* = 5.0 Hz, 1H), 1.44 (d, *J* = 5.8 Hz, 1H). **^{13}C NMR** (126 MHz, $CDCl_3$) δ 144.30, 135.04, 134.58, 132.26, 131.36, 130.06, 127.06, 123.86, 122.45, 116.79 (t, *J* = 239.6 Hz), 107.03, 47.04, 39.60 (d, *J* = 7.8 Hz), 26.29 (t, *J* = 26.9 Hz), 21.60, 19.49 (d, *J* = 5.7 Hz). **^{19}F NMR** (471 MHz, Chloroform-*d*) δ -113.73 (d, *J* = 284.4 Hz), -120.74 (d, *J* = 284.4 Hz). **HRMS**: Calculation for $C_{20}H_{18}BrF_2NO_2S$, $[M+H]^+$, 476.0102 Found: 476.0093. HPLC: OD-H column, 98:2 hexane: isopropanol, 1.0 mL/min, t_R = major: 18.4 min, minor: 19.5 min. 96% ee. $[\alpha]_D^{29}$ = 24° (c 0.5, CH_2Cl_2).

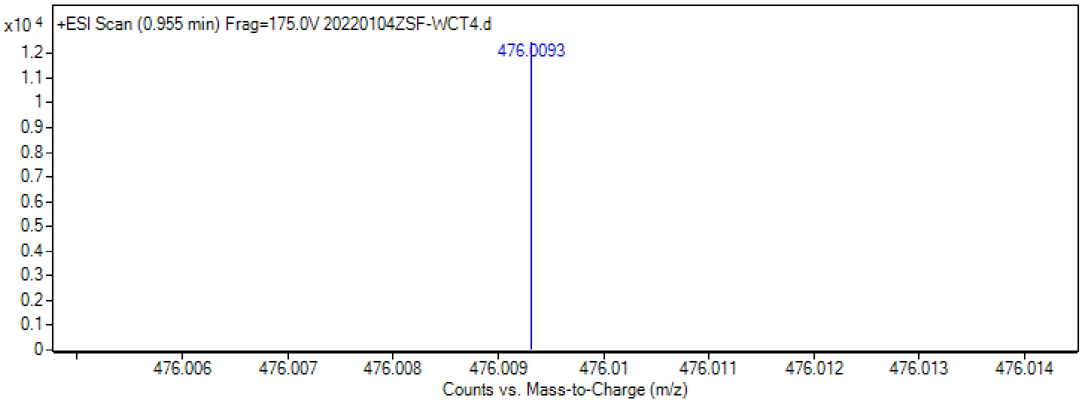

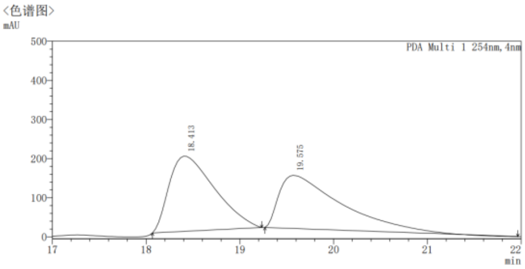

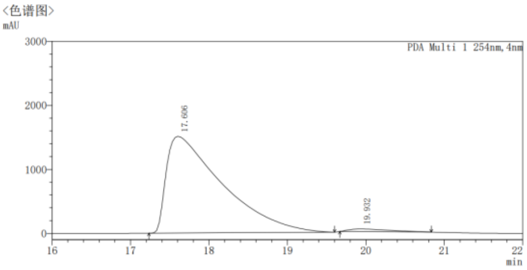

<峰表>

PDA Ch1 254nm

峰号	保留时间	面积	高度	高度%	面积%
1	18.413	6517688	192054	58.605	50.957
2	19.575	6272836	135656	41.395	49.043
总计		12790524	327710	100.000	100.000

<峰表>

PDA Ch1 254nm

峰号	保留时间	面积	高度	高度%	面积%
1	17.606	74807204	1510062	97.330	97.992
2	19.932	1533221	41419	2.670	2.008
总计		76340425	1551480	100.000	100.000

(1*S*,6*S*)-6-(difluoromethyl)-3-tosyl-1-(4-(trifluoromethyl)phenyl)-3-azabicyclo[4.1.0]hept-4-ene (2v)

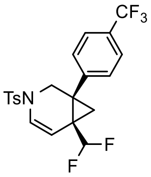

Yellow solid, 19 mg, 96% yield, m.p. = 45.3 -45.5 °C, Rf = 0.2 (PE: EtOAc = 10: 1). **^{1}H NMR** (500 MHz, Chloroform-d) δ 7.58 (d, *J* = 8.0 Hz, 2H), 7.53 (d, *J* = 8.0 Hz, 2H), 7.37 (d, *J* = 8.0 Hz, 2H), 7.32 – 7.11 (m, 2H), 6.54 (d, *J* = 8.2 Hz, 1H), 5.61 (d, *J* = 8.2 Hz, 1H), 4.78 (t, *J* = 54.4 Hz, 1H), 3.90 (dd, *J* = 11.8, 3.0 Hz, 1H), 2.92 (d, *J* = 11.8 Hz, 1H), 2.38 (s, 3H), 1.49 (d, *J* = 7.8 Hz, 1H), 1.43 (d, *J* = 5.8 Hz, 1H). **^{13}C NMR** (126 MHz, CDCl3) δ 144.36, 140.09, 134.56, 130.65 (d, *J* = 32.5 Hz), 130.15, 130.08, 127.07, 126.06 (q, *J* = 3.8 Hz), 124.07, 123.78 (q, *J* = 272.1 Hz), 116.61 (t, *J* = 239.8 Hz), 106.90 (d, *J* = 2.9 Hz), 47.06, 39.77 (d, *J* = 7.5 Hz), 26.43 (t, *J* = 27.0 Hz), 21.58, 19.46 (d, *J* = 5.8 Hz). **^{19}F NMR** (471 MHz, $CDCl_3$) δ -62.78, -113.98 (d, *J* = 286.5 Hz), -120.58 (d, *J* = 284.9 Hz). **HRMS**: Calculation for $C_{21}H_{18}F_5NO_2S$, $[M+Na]^+$, 446.0870 Found: 446.0858. HPLC: INA column, 98:2 hexane: isopropanol, 0.80 mL/min, t_R = major: 21.3 min, minor: 23 min. 94% ee. $[\alpha]_D^{29}$ = 33° (c 0.6, CH_2Cl_2).

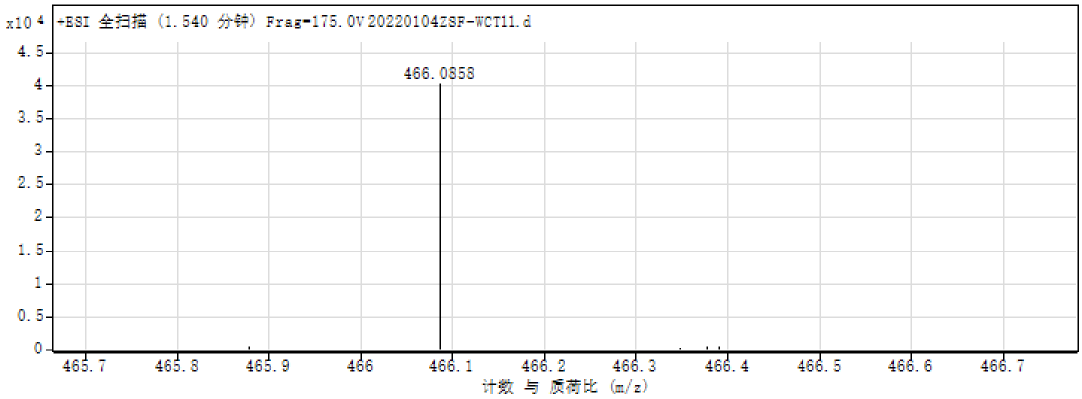

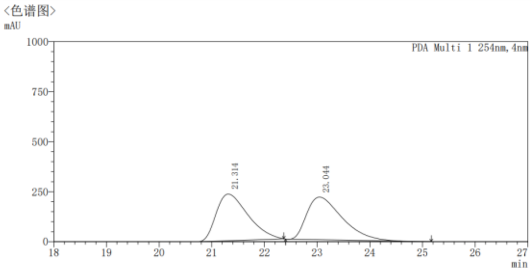

<峰表>
PDA Ch1 254nm

峰号	保留时间	面积	高度	高度%	面积%
1	21.314	9819303	233818	52.339	49.627
2	23.044	9966805	212916	47.661	50.373
总计		19786109	446734	100.000	100.000

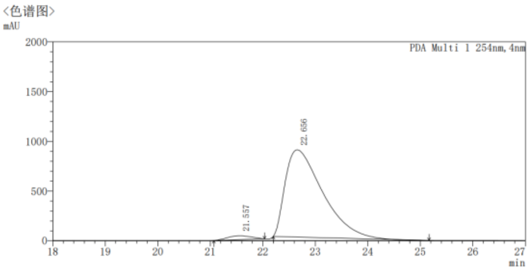

<峰表>
PDA Ch1 254nm

峰号	保留时间	面积	高度	高度%	面积%
1	21.557	1253691	38914	4.249	2.799
2	22.656	43535313	877024	95.751	97.201
总计		44789004	915938	100.000	100.000

(1*S*,6*S*)-1-(3-((4l2-buta-1,2,3-trien-1-yl)oxy)phenyl)-6-(difluoromethyl)-3-tosyl-3-azabicyclo[4.1.0]hept-4-ene (2w)

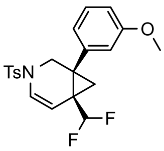

Yellow solid, 18 mg, 90% yield, m.p. = 134.1 – 134.3 ºC, Rf = 0.2 (PE: EtOAc = 10: 1. **^{1}H NMR** (500 MHz, Chloroform-*d*) δ 7.64 (d, *J* = 7.9 Hz, 2H), 7.33 (d, *J* = 8.0 Hz, 2H), 7.24 (t, *J* = 8.5 Hz, 1H), 6.88 (d, *J* = 7.6 Hz, 1H), 6.83 (d, *J* = 6.8 Hz, 2H), 6.57 (d, *J* = 8.2 Hz, 1H), 5.68 (d, *J* = 8.2 Hz, 1H), 4.85 (t, *J* = 54.4 Hz, 1H), 3.98 (dd, *J* = 11.9, 3.3 Hz, 1H), 3.77 (s, 3H), 3.01 (d, *J* = 11.8 Hz, 1H), 2.44 (s, 3H), 1.45 (s, 2H). **^{13}C NMR** (126 MHz, $CDCl_3$) δ 159.96, 144.21, 137.50, 134.74, 130.12, 130.03, 127.08, 123.68, 121.83, 115.36, 117.01 (t, *J* = 239.2 Hz), 113.73, 107.30 (t, *J* = 3.4 Hz), 55.29, 47.14, 40.36 (d, *J* = 8.2 Hz), 26.30 (t, *J* = 27.1 Hz,) 21.57, 19.64 (d, *J* = 5.6 Hz). **^{19}F NMR** (471 MHz, Chloroform-*d*) δ -113.41 (d, *J* = 284.1 Hz), -120.74 (d, *J* = 282.6 Hz). **HRMS**: Calculation for $C_{21}H_{21}F_2NO_3S$, $[M+H]^+$, 406.1283 Found: 406.1281. HPLC: INA column, 95:5 hexane: isopropanol, 0.80 mL/min, t_R = major: 22.8 min, minor: 19.8 min. 92% ee. $[\alpha]_D^{29}$ = 58° (c 0.6, CH_2Cl_2).

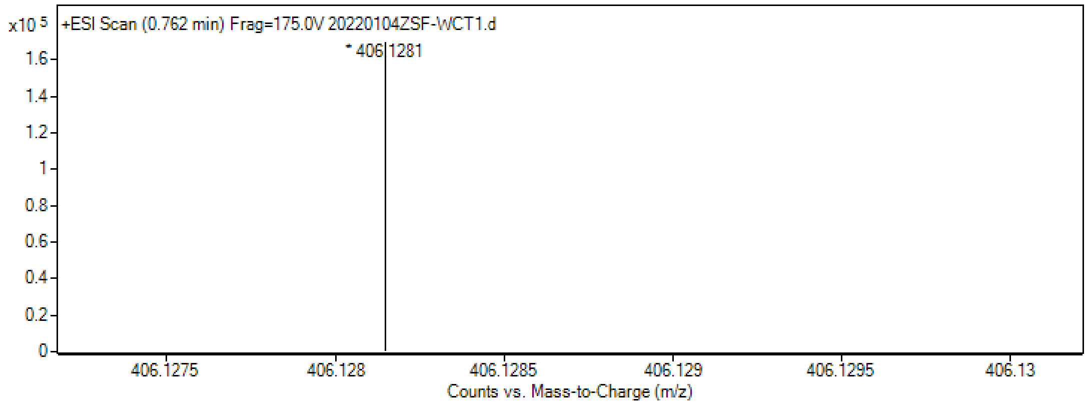

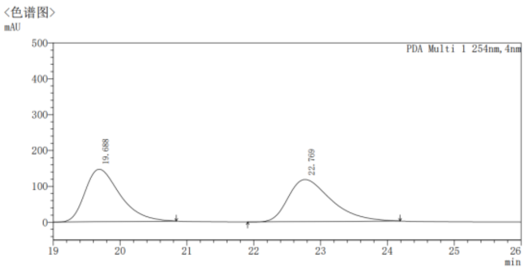

<峰表>

PDA Ch1 254nm

峰号	保留时间	面积	高度	高度%	面积%
1	19.688	5324129	146446	55.508	50.314
2	22.769	5257744	117382	44.492	49.686
总计		10581873	263827	100.000	100.000

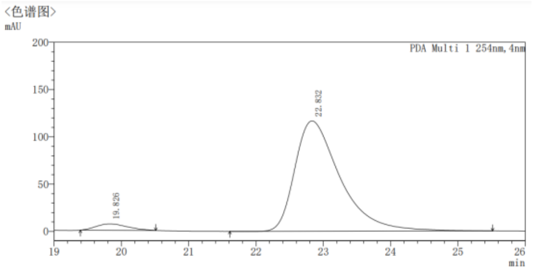

<峰表>

PDA Ch1 254nm

峰号	保留时间	面积	高度	高度%	面积%
1	19.826	212589	6663	5.404	3.753
2	22.832	5451209	116623	94.596	96.247
总计		5663798	123286	100.000	100.000

(1*S*,6*S*)-6-(difluoromethyl)-1-(m-tolyl)-3-tosyl-3-azabicyclo[4.1.0]hept-4-ene (2x)

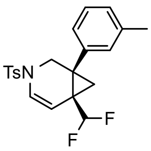

Yellow solid, 17 mg, 89% yield, m.p. = 95.9 -96.2 °C, Rf = 0.2 (PE: EtOAc = 10: 1). **^{1}H NMR** (500 MHz, Chloroform-d) δ 7.64 (d, *J* = 8.0 Hz, 2H), 7.33 (d, *J* = 7.9 Hz, 2H), 7.21 (t, *J* = 7.7 Hz, 1H), 7.10 (d, *J* = 7.6 Hz, 3H), 6.57 (d, *J* = 8.1 Hz, 1H), 5.69 (d, *J* = 8.2 Hz, 1H), 4.83 (t, *J* = 54.5 Hz, 1H), 3.97 (dd, *J* = 11.7, 3.3 Hz, 1H), 3.00 (d, *J* = 11.8 Hz, 1H), 2.44 (s, 3H), 2.32 (s, 3H), 1.46 (d, *J* = 4.7 Hz, 2H). **^{13}C NMR** (126 MHz, CDCl3) δ 144.17, 138.84, 135.91, 134.76, 130.33, 130.02, 129.08, 128.91, 127.09, 126.68, 123.60, 117.06 (t, *J* = 239.0 Hz), 107.41, 47.25, 40.36 (d, *J* = 8.1 Hz), 26.09 (t, *J* = 26.8 Hz), 21.60, 21.34 19.50 (d, *J* = 5.7 Hz). **^{19}F NMR** (471 MHz, Chloroform-*d*) δ -113.58 (d, *J* = 282.8 Hz), -120.77 (d, *J* = 282.8 Hz). **HRMS**: Calculation for $C_{21}H_{21}F_2NO_2S$, $[M+H^+]^+$, 390.1334 Found: 390.1332. HPLC: OJH column, 95:5 hexane: isopropanol, 0.40 mL/min, t_R = major: 38.2 min, minor: 24.5 min. 91% ee. $[\alpha]_D^{29}$ = 54° (c 1.8, CH_2Cl_2).

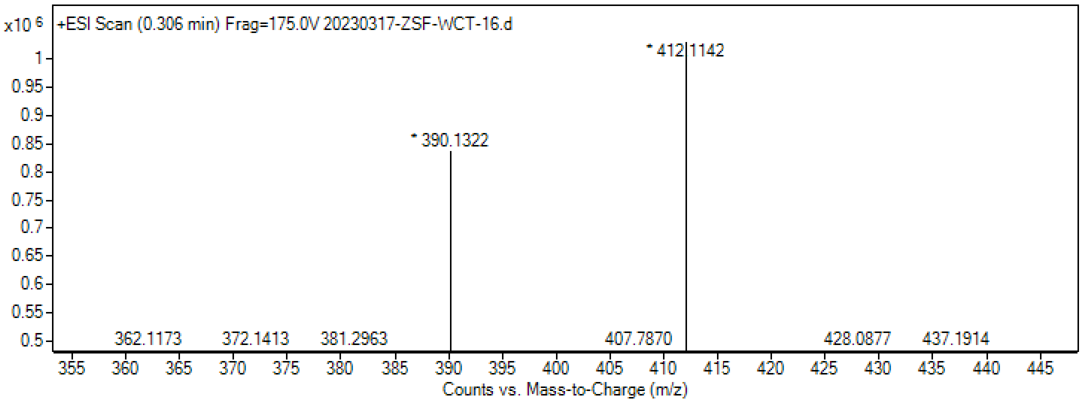

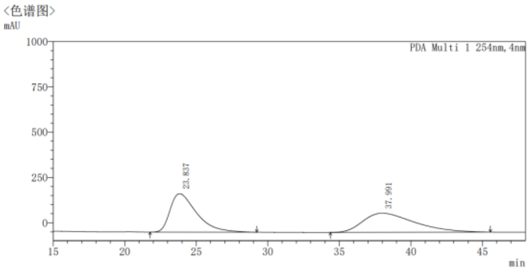

<峰表>
PDA Ch1 254nm

峰号	保留时间	面积	高度	高度%	面积%
1	23.837	27376750	212411	66.775	50.682
2	37.991	26640117	105689	33.225	49.318
总计		54016867	318100	100.000	100.000

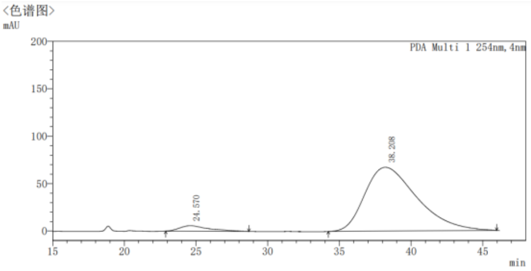

<峰表>
PDA Ch1 254nm

峰号	保留时间	面积	高度	高度%	面积%
1	24.570	812068	5751	7.879	4.545
2	38.208	17056020	67247	92.121	95.455
总计		17868088	72999	100.000	100.000

(1*S*,6*S*)-1-(3-chlorophenyl)-6-(difluoromethyl)-3-tosyl-3-azabicyclo[4.1.0]hept-4-ene (2y)

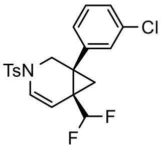

Yellow solid, 17 mg, 84% yield, m.p. = 55.3 - 55.5 °C, Rf = 0.2 (PE: EtOAc = 10: 1). **^{1}H NMR** (500 MHz, Chloroform-*d*) δ 7.65 (d, *J* = 8.1 Hz, 2H), 7.34 (d, *J* = 8.0 Hz, 2H), 7.31 – 7.23 (m, 3H), 7.22 – 7.18 (m, 1H), 6.59 (d, *J* = 8.2 Hz, 1H), 5.67 (d, *J* = 8.2 Hz, 1H), 4.86 (t, *J* = 54.4 Hz, 1H), 3.96 (dd, *J* = 11.8, 3.2 Hz, 1H), 2.99 (d, *J* = 11.9 Hz, 1H), 2.44 (s, 3H), 1.50 – 1.44 (m, 2H). **^{13}C NMR** (126 MHz, $CDCl_3$) δ 144.34, 138.07, 134.86, 134.66, 130.38, 130.30, 129.79, 128.65, 127.89, 127.06, 123.92, 116.71 (t, *J* = 239.8 Hz), 107.07 (t, *J* = 3.6 Hz), 47.05, 39.83 (d, *J* = 7.7 Hz), 26.41 (t, *J* = 27.0 Hz), 21.59, 19.49 (d, *J* = 5.8 Hz). **^{19}F NMR** (471 MHz, Chloroform-*d*) δ -113.75 (d, *J* = 284.4 Hz), -120.64 (d, *J* = 284.4 Hz). **HRMS**: Calculation for $C_{20}H_{18}ClF_2NO_2S$, $[M+H^+]^+$, 410.0788 Found: 410.0779. HPLC: INA column, 99:1 hexane: isopropanol, 1.0 mL/min, t_R = major: 22.7 min, minor: 21.1 min. 93% ee. $[\alpha]_D^{29}$ = 82° (c 0.6, CH_2Cl_2).

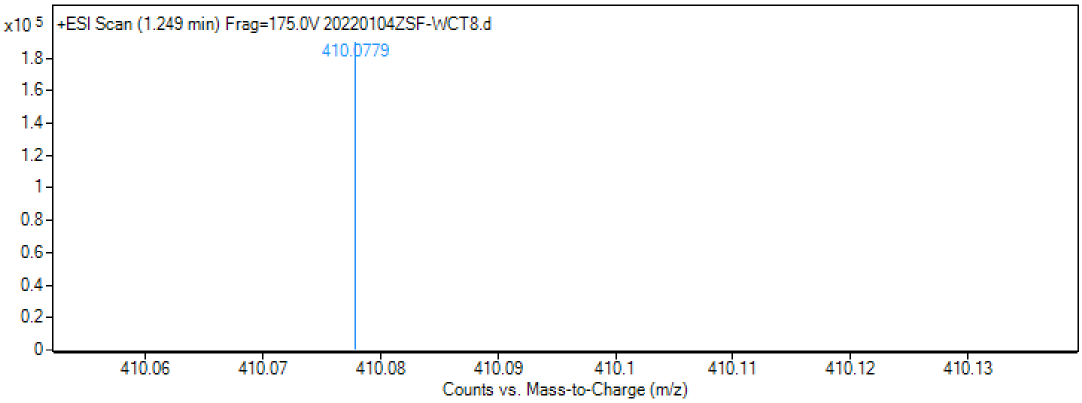

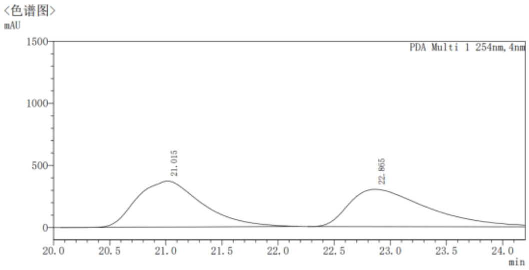

<峰表>
PDA Ch1 254nm

峰号	保留时间	面积	高度	高度%	面积%
1	21.015	14996912	370839	55.205	50.253
2	22.865	14846074	300913	44.795	49.747
总计		29842986	671752	100.000	100.000

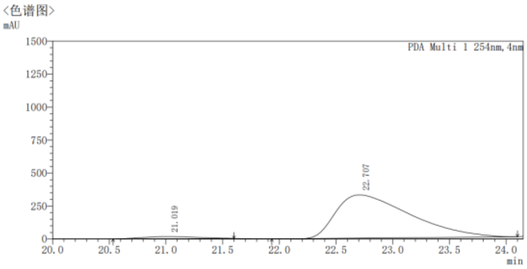

<峰表>
PDA Ch1 254nm

峰号	保留时间	面积	高度	高度%	面积%
1	21.019	536920	15536	4.527	3.481
2	22.707	14886847	327658	95.473	96.519
总计		15423766	343195	100.000	100.000

(1*S*,6*S*)-6-(difluoromethyl)-1-(2-methoxyphenyl)-3-tosyl-3-azabicyclo[4.1.0]hept-4-ene (2z)

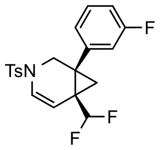

Yellow oil, 15 mg, 79% yield, m.p. = 55.3 - 55.5 ºC, Rf = 0.2 (PE: EtOAc = 10: 1). ^{1}**H NMR** (500 MHz, Chloroform-*d*) δ 7.65 (d, *J* = 8.1 Hz, 2H), 7.34 (d, *J* = 8.1 Hz, 2H), 7.33 – 7.27 (m, 1H), 7.09 (d, *J* = 7.7 Hz, 1H), 7.00 (d, *J* = 8.7 Hz, 2H), 6.59 (d, *J* = 8.1 Hz, 1H), 5.67 (d, *J* = 8.2 Hz, 1H), 4.85 (t, *J* = 54.4 Hz, 1H), 3.98 (dd, *J* = 11.8, 3.1 Hz, 1H), 2.99 (d, *J* = 11.8 Hz, 1H), 2.45 (s, 3H), 1.47 (s, 2H). ^{13}**C NMR** (126 MHz, $CDCl_3$) δ 162.85 (d, *J* = 247.9 Hz), 144.33, 138.42 (d, *J* = 7.3 Hz), 134.58, 130.71 (d, *J* = 8.4 Hz), 130.08, 127.05, 125.31, 123.88, 117.70 (t, *J* = 239.3 Hz), 116.58, 115.48 (d, *J* = 20.9 Hz), 107.10, 47.01, 39.79 (d, *J* = 9.4 Hz), 26.44 (t, *J* = 27.1 Hz), 21.59, 19.56 (d, *J* = 5.7 Hz). ^{19}**F NMR** (471 MHz, Chloroform-*d*) δ -113.73 (d, *J* = 284.2 Hz), -120.69 (d, *J* = 284.2 Hz). **HRMS**: Calculation for $C_{20}H_{18}F_3NO_2S$, $[M+Na^+]^+$, 416.0903 Found: 416.0907. HPLC: OJH column, 95:5 hexane: isopropanol, 1.0 mL/min, t_R = major: 74.7 min, minor: 46.8 min. 93% ee. $[\alpha]_D^{29}$ = 14° (c 0.6, CH_2Cl_2).

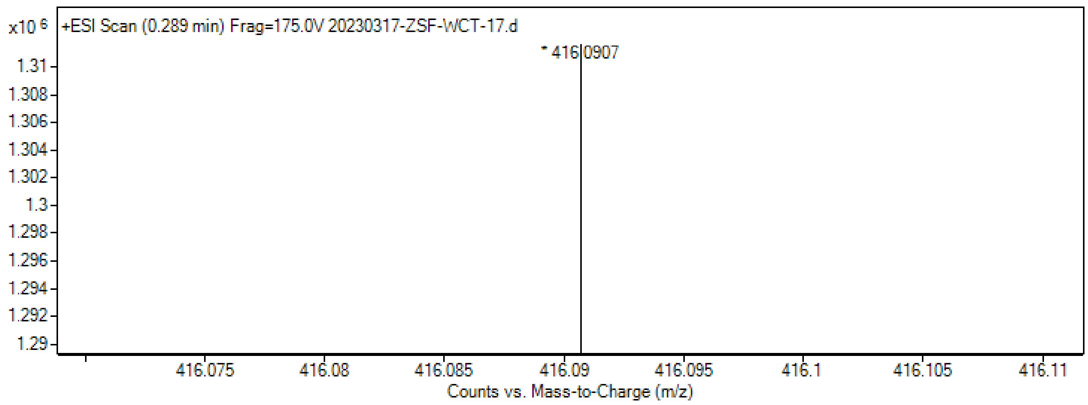

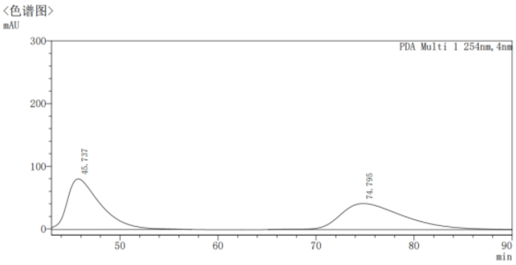

<峰表>
PDA Ch1 254nm

峰号	保留时间	面积	高度	高度%	面积%
1	45.737	19227428	80851	66.055	50.592
2	74.795	18777212	41549	33.945	49.408
总计		38004639	122400	100.000	100.000

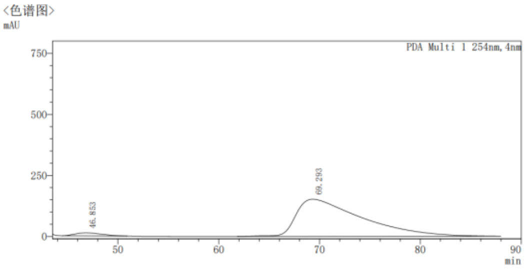

<峰表>
PDA Ch1 254nm

峰号	保留时间	面积	高度	高度%	面积%
1	46.853	2282156	12301	7.479	3.220
2	69.293	68583460	152181	92.521	96.780
总计		70865616	164483	100.000	100.000

(1*S*,6*S*)-6-(difluoromethyl)-1-mesityl-3-tosyl-3-azabicyclo[4.1.0]hept-4-ene (2aa)

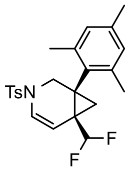

Yellow solid, 16 mg, 75% yield, m.p. = 86.1 - 86.4 °C, Rf = 0.2 (PE: EtOAc = 10: 1). **^{1}H NMR** (500 MHz, Chloroform-*d*) δ 7.72 – 7.59 (m, 2H), 7.32 (d, *J* = 8.0 Hz, 2H), 6.83 (s, 1H), 6.80 (s, 1H), 6.58 (d, *J* = 8.1 Hz, 1H), 5.68 (d, *J* = 8.2 Hz, 1H), 4.91 (t, *J* = 55.1 Hz, 1H), 3.84 (dd, *J* = 12.5, 2.8 Hz, 1H), 2.94 (d, *J* = 12.4 Hz, 1H), 2.43 (s, 3H), 2.28 (s, 3H), 2.22 (s, 3H), 2.12 (s, 3H), 1.46 (dd, *J* = 5.7, 2.5 Hz, 1H), 1.23 (d, *J* = 5.8 Hz, 1H). **^{13}C NMR** (126 MHz, CDCl3) δ 144.20, 138.64, 137.99, 137.71, 134.83, 130.11, 129.98, 129.62, 129.39, 127.06, 123.08, 115.48 (t, *J* = 238.1 Hz), 107.61 (d, *J* = 5.0 Hz), 43.84, 38.18 (d, *J* = 8.3 Hz), 27.32 (t, *J* = 26.1 Hz), 22.08 (t, *J* = 3.6 Hz), 21.60, 20.79, 20.48, 19.88 (d, *J* = 6.4 Hz). **^{19}F NMR** (471 MHz, Chloroform-*d*) δ -111.33 (d, *J* = 281.7 Hz), -116.21 (d, *J* = 281.7 Hz). **HRMS**: Calculation for $C_{23}H_{25}F_2NO_2S$, $[M+Na]^+$, 440.1467 Found: 440.1468. HPLC: whelk column, 95:5 hexane: isopropanol, 1.0 mL/min, t_R = major: 22.5 min, minor: 18.4 min. 95% ee. $[\alpha]_D^{29}$ = 46° (c 0.6, CH_2Cl_2).

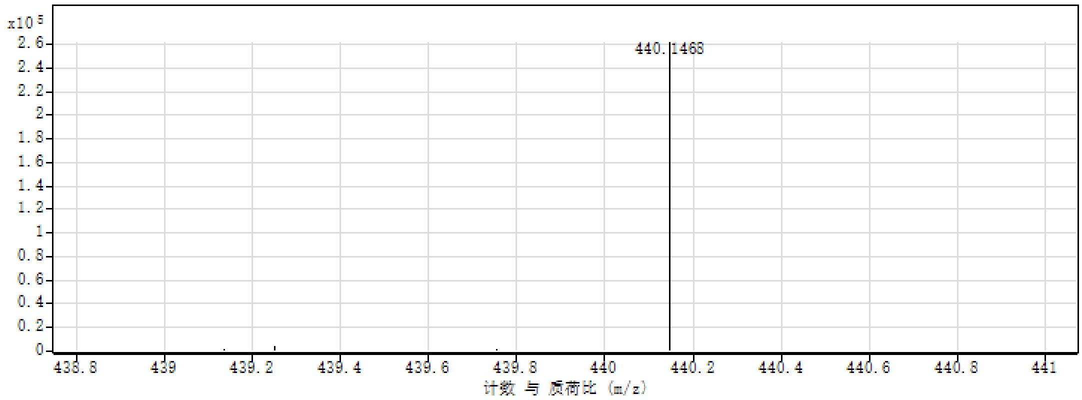

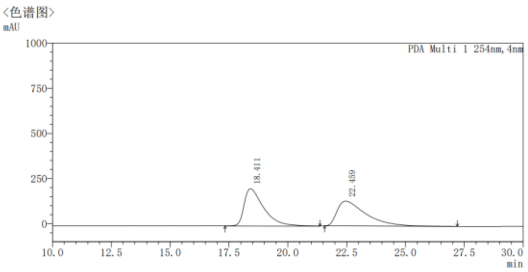

<峰表>
PDA Ch1 254nm

峰号	保留时间	面积	高度	高度%	面积%
1	18.411	12112249	207030	60.051	50.747
2	22.459	11755691	137728	39.949	49.253
总计		23867939	344758	100.000	100.000

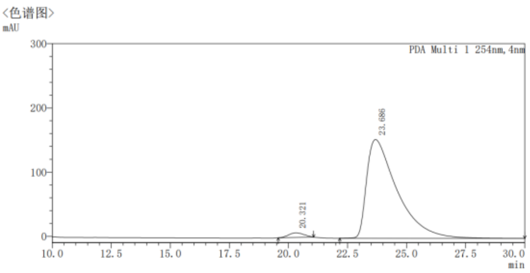

<峰表>
PDA Ch1 254nm

峰号	保留时间	面积	高度	高度%	面积%
1	20.321	325784	7206	4.477	2.299
2	23.686	13843291	153755	95.523	97.701
总计		14169076	160961	100.000	100.000

(1*S*,6*S*)-6-(difluoromethyl)-1-(thiophen-2-yl)-3-tosyl-3-azabicyclo[4.1.0]hept-4-ene (2ab)

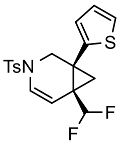

Yellow solid, 18 mg, 95% yield, m.p. = 85.6 - 86.7 °C, Rf = 0.2 (PE: EtOAc = 10: 1). **^{1}H NMR** (500 MHz, Chloroform-*d*) δ 7.66 (d, *J* = 8.1 Hz, 2H), 7.34 (d, *J* = 8.0 Hz, 2H), 7.23 (dd, *J* = 4.6, 1.8 Hz, 1H), 6.95 (d, *J* = 4.5 Hz, 2H), 6.57 (d, *J* = 8.2 Hz, 1H), 5.65 (d, *J* = 8.2 Hz, 1H), 4.98 (t, *J* = 54.5 Hz, 1H), 4.09 (dd, *J* = 11.8, 3.4 Hz, 1H), 3.08 (d, *J* = 11.7 Hz, 1H), 2.44 (s, 3H), 1.58 (d, *J* = 5.4 Hz, 1H), 1.54 (t, *J* = 5.1 Hz, 1H). **^{13}C NMR** (126 MHz, $CDCl_3$) δ 144.31, 139.16, 134.70, 130.07, 127.69, 127.22, 127.08, 125.84, 123.86, 115.71 (t, *J* = 239.6 Hz), 106.75 (t, *J* = 3.6 Hz), 46.97, 35.08 (d, *J* = 8.1 Hz), 27.55 (t, *J* = 27.4 Hz), 21.58, 21.12 (d, *J* = 5.7 Hz). **^{19}F NMR** (471 MHz, Chloroform-*d*) δ -113.74 (d, *J* = 284.5 Hz), -121.65 (d, *J* = 284.7 Hz). **HRMS**: Calculation for $C_{18}H_{17}F_2NO_2S_2$, $[M+H]^+$, 382.0742 Found: 382.0737. HPLC: INA column, 95:5 hexane: isopropanol, 0.80 mL/min, t_R = major: 16.9 min, minor: 15.5 min. 90% ee. $[\alpha]_D^{29}$ = 73° (c 1.4, CH_2Cl_2).

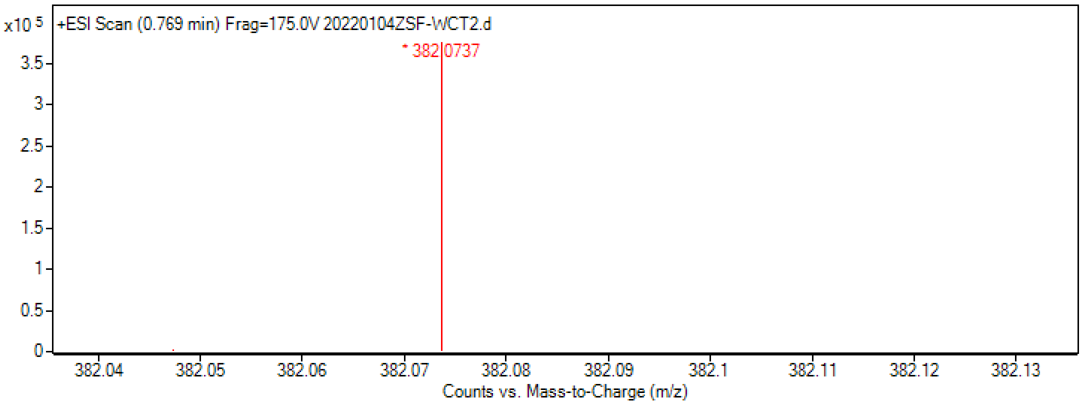

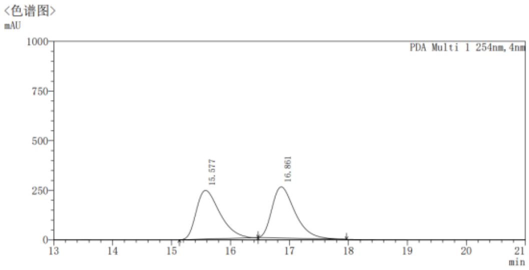

<峰表>

PDA Ch1 254nm

峰号	保留时间	面积	高度	高度%	面积%
1	15.577	6860596	245677	48.758	49.515
2	16.861	6994888	258189	51.242	50.485
总计		13855484	503866	100.000	100.000

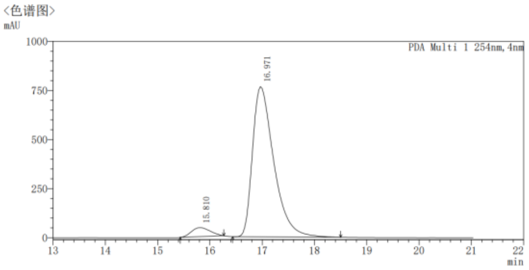

<峰表>

PDA Ch1 254nm

峰号	保留时间	面积	高度	高度%	面积%
1	15.810	1145663	45981	5.672	5.039
2	16.971	21591799	764623	94.328	94.961
总计		22737462	810603	100.000	100.000

7

(1*S*,6*S*)-6-(difluoromethyl)-1-(furan-2-yl)-3-tosyl-3-azabicyclo[4.1.0]hept-4-ene (2ac)

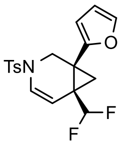

Yellow solid, 17 mg, 92% yield, m.p. = 85.6 - 86.7 °C, Rf = 0.2 (PE: EtOAc = 10: 1). **^{1}H NMR** (500 MHz, Chloroform-*d*) δ 7.67 (d, *J* = 7.9 Hz, 2H), 7.37 – 7.30 (m, 3H), 6.57 (d, *J* = 8.2 Hz, 1H), 6.32 (t, *J* = 2.6 Hz, 1H), 6.21 (d, *J* = 3.3 Hz, 1H), 5.62 (d, *J* = 8.2 Hz, 1H), 5.02 (t, *J* = 54.7 Hz, 1H), 4.05 (dd, *J* = 11.7, 3.3 Hz, 1H), 3.22 (d, *J* = 11.6 Hz, 1H), 2.44 (s, 3H), 1.61 (d, *J* = 5.6 Hz, 1H), 1.42 (t, *J* = 5.3 Hz, 1H). **^{13}C NMR** (126 MHz, $CDCl_3$) δ 149.93, 144.32, 142.56, 134.61, 130.07, 127.09, 123.80, 116.50 (t, *J* = 239.2 Hz), 110.57, 108.73, 106.40 (t, *J* = 3.5 Hz), 43.60, 33.13 (d, *J* = 7.3 Hz), 27.26 (t, *J* = 27.3 Hz), 21.60, 19.58 (d, *J* = 6.2 Hz). **^{19}F NMR** (471 MHz, Chloroform-*d*) δ -113.28 (d, *J* = 285.3 Hz), -120.93 (d, *J* = 285.9 Hz). **HRMS**: Calculation for $C_{18}H_{17}F_2NO_3S$, $[M+Na]^+$, 388.0789 Found: 388.0781. HPLC: INA column, 95:5 hexane: isopropanol, 0.80 mL/min, t_R = major:17.1 min, minor: 15.8 min. 91% ee. $[\alpha]_D^{29}$ = 15° (c 0.3, CH_2Cl_2).

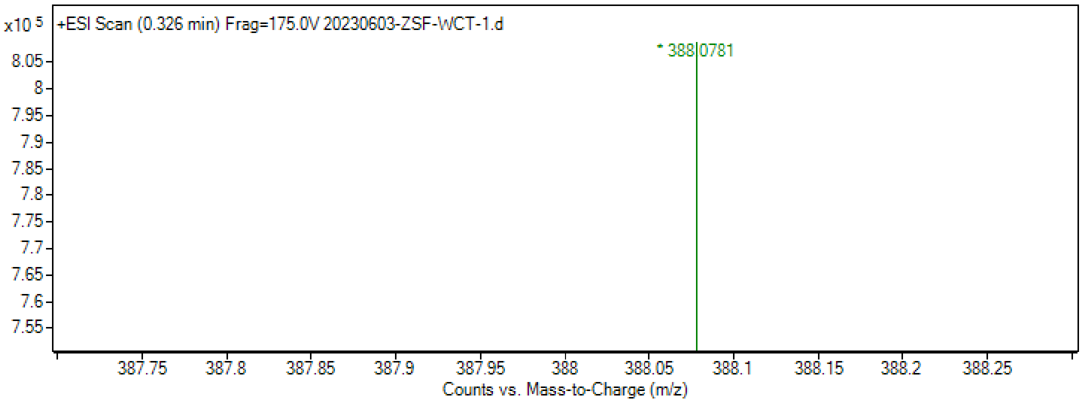

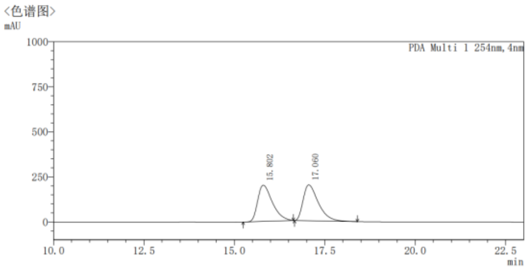

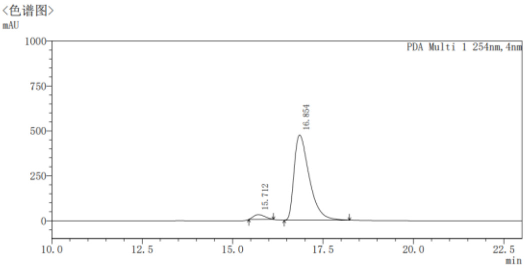

<峰表>
PDA Ch1 254nm

峰号	保留时间	面积	高度	高度%	面积%
1	15.802	5664191	201118	50.251	49.348
2	17.060	5813980	199112	49.749	50.652
总计		11478171	400230	100.000	100.000

<峰表>
PDA Ch1 254nm

峰号	保留时间	面积	高度	高度%	面积%
1	15.712	593468	26876	5.364	4.117
2	16.854	13822181	474152	94.636	95.883
总计		14415649	501029	100.000	100.000

(1*S*,6*S*)-6-(difluoromethyl)-3-tosyl-3-azabicyclo[4.1.0]hept-4-ene (2ad)

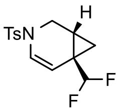

Colorless oil, 14 mg, 95% yield, Rf = 0.2 (PE: EtOAc = 10: 1). **^{1}H NMR** (500 MHz, Chloroform-*d*) δ 7.58 (d, *J* = 8.3 Hz, 2H), 7.25 (d, *J* = 8.1 Hz, 2H), 6.45 (d, *J* = 8.3 Hz, 1H), 5.46 (t, *J* = 57.3 Hz, 1H), 5.32 (d, *J* = 8.3 Hz, 1H), 3.87 (d, *J* = 11.8 Hz, 1H), 2.93 (dd, *J* = 11.8, 2.6 Hz, 1H), 2.36 (s, 3H), 1.71 (t, *J* = 7.6 Hz, 1H), 1.05 (dd, *J* = 9.1, 5.1 Hz, 1H), 0.64 (s, 1H). **^{13}C NMR** (126 MHz, $CDCl_3$) δ 144.11, 134.61, 129.91, 127.05, 116.23 (t, *J* = 240.6 Hz), 106.35, 39.90, 21.70 (t, *J* = 4.1 Hz), 21.55, 20.21 (t, *J* = 25.7 Hz), 16.01 (t, *J* = 4.1 Hz). **^{19}F NMR** (471 MHz, Chloroform-*d*) δ -120.14 (d, *J* = 280.4 Hz), -120.96 (d, *J* = 280.4 Hz). **HRMS**: Calculation for $C_{14}H_{15}F_2NO_2S$, $[M+H]^+$, 300.0864 Found: 300.0853. HPLC: INA column, 95:5 hexane: isopropanol, 0.70 mL/min, t_R = major: 23.9 min, minor: 22.8 min. 88% ee. $[\alpha]_D^{29}$ = 16° (c 0.4, CH_2Cl_2).

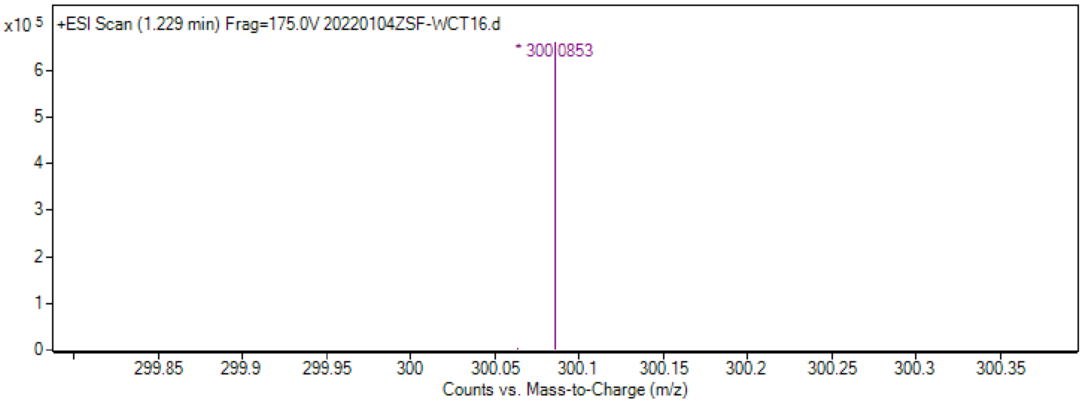

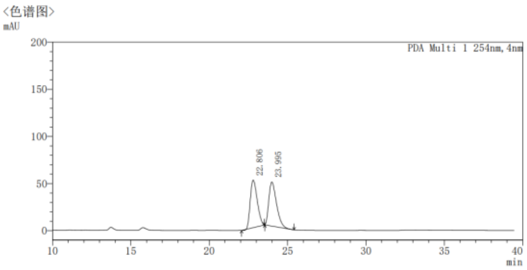

〈峰表〉
PDA Ch1 254nm

峰号	保留时间	面积	高度	高度%	面积%
1	22.806	1558334	50426	51.886	49.416
2	23.995	1595148	46760	48.114	50.584
总计		3153482	97186	100.000	100.000

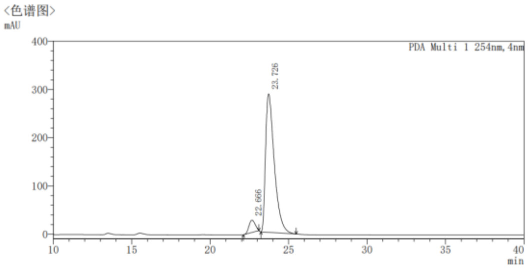

〈峰表〉
PDA Ch1 254nm

峰号	保留时间	面积	高度	高度%	面积%
1	22.666	697999	25523	8.168	5.903
2	23.726	11127389	286940	91.832	94.097
总计		11825388	312463	100.000	100.000

(1*R*,6*R*,7*S*)-6-(difluoromethyl)-7-phenyl-3-tosyl-3-azabicyclo[4.1.0]hept-4-ene (2ae)

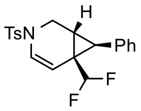

Yellow solid, 18 mg, 92% yield, m.p. = 134.9 - 135.1 ºC, Rf = 0.2 (PE: EtOAc = 10: 1). **^{1}H NMR** (500 MHz, Chloroform-*d*) δ 7.68 (d, *J* = 8.3 Hz, 2H), 7.31 (d, *J* = 8.2 Hz, 2H), 7.26 (d, *J* = 2.8 Hz, 2H), 7.23 (d, *J* = 7.2 Hz, 1H), 7.11 (d, *J* = 7.3 Hz, 2H), 6.62 (d, *J* = 8.3 Hz, 1H), 5.66 (d, *J* = 8.3 Hz, 1H), 4.94 (t, *J* = 54.2 Hz, 1H), 4.17 (d, *J* = 11.7 Hz, 1H), 3.08 (dd, *J* = 11.7, 2.6 Hz, 1H), 2.39 (s, 4H), 2.33 (d, *J* = 6.2 Hz, 1H). **^{13}C NMR** (126 MHz, $CDCl_3$) δ 144.25, 134.48, 134.28, 130.02, 128.74, 128.65, 127.40, 127.06, 123.84, 116.27 (t, *J* = 239.0 Hz), 106.12, 39.52, 34.55 (d, *J* = 5.4 Hz), 26.51, 26.26 (d, *J* = 5.6 Hz), 21.51. **^{19}F NMR** (471 MHz, Chloroform-*d*) δ -117.65 (d, *J* = 285.8 Hz), -118.48 (d, *J* = 284.7 Hz). **HRMS**: Calculation for $C_{20}H_{19}F_2NO_2S$, $[M+H]^+$, 376.1177 Found: 376.1169. HPLC: OD-H column, 99:1 hexane: isopropanol, 1.0 mL/min, t_R = major: 52.9 min, minor: 37.9 min. 69% ee. $[\alpha]_D^{29}$ = -13° (c 0.6, CH_2Cl_2).

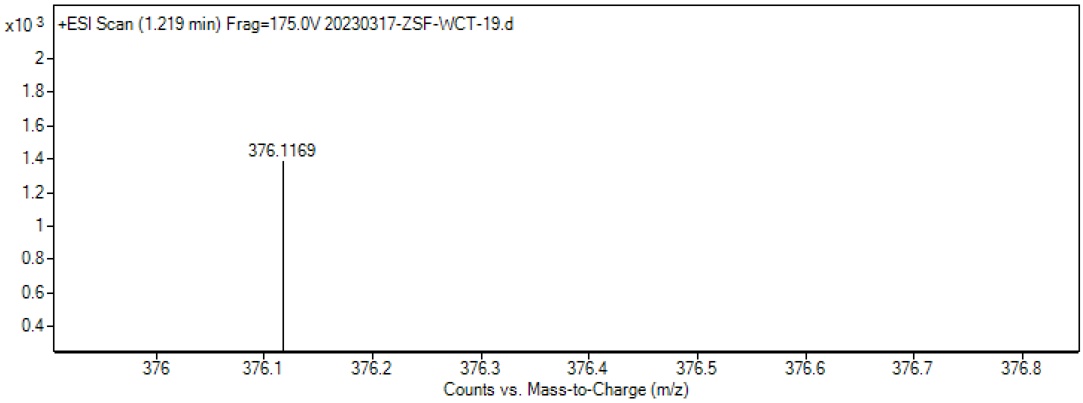

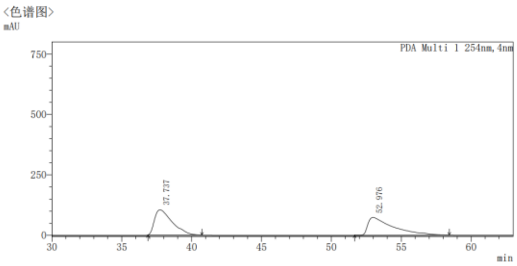

〈峰表〉
PDA Ch1 254nm

峰号	保留时间	面积	高度	面积%	高度%
1	37.737	9105307	104006	49.823	58.561
2	52.976	9165427	73404	50.152	41.330
3	69.259	4738	193	0.026	0.109
总计		18275472	177603	100.000	100.000

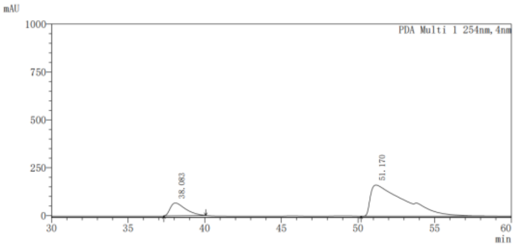

〈峰表〉
PDA Ch1 254nm

峰号	保留时间	面积	高度	高度%	面积%
1	38.083	4659123	66912	29.194	15.449
2	51.170	25498358	162283	70.806	84.551
总计		30157481	229195	100.000	100.000

(1*S*,6*S*)-6-(difluoromethyl)-1-(p-tolyl)-3-oxabicyclo[4.1.0]hept-4-ene (2af)

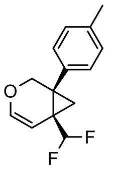

Colorless oil, 10 mg, 86% yield, Rf = 0.2 (PE: EtOAc = 30: 1). **^{1}H NMR** (500 MHz, Chloroform-*d*) δ 7.27 (d, *J* = 7.9 Hz, 2H), 7.15 (d, *J* = 7.8 Hz, 2H), 6.37 (d, *J* = 5.9 Hz, 1H), 5.56 (d, *J* = 5.9 Hz, 1H), 4.91 (t, *J* = 54.7 Hz, 1H), 4.04 (dd, *J* = 10.7, 3.6 Hz, 1H), 3.80 (d, *J* = 10.7 Hz, 1H), 2.34 (s, 3H), 1.73 (t, *J* = 4.7 Hz, 1H), 1.57 (d, *J* = 5.0 Hz, 1H). **^{13}C NMR** (126 MHz, $CDCl_3$) δ 143.48, 137.87, 132.48, 129.68, 129.59, 117.29 (t, *J* = 239.3 Hz), 102.42, 67.35, 38.16 (d, *J* = 7.3 Hz), 25.23, (t, *J* = 26.7 Hz), 21.11, 19.38 (d, *J* = 6.4 Hz). **^{19}F NMR** (471 MHz, Chloroform-*d*) δ -113.75 (d, *J* = 282.6 Hz), -121.20 (d, *J* = 282.6 Hz). **HRMS**: Calculation for $C_{14}H_{14}F_2O$, $[M+Na]^+$, 259.0905 Found: 259.0897. HPLC: INC column, 99:1 hexane: isopropanol, 0.80 mL/min, t_R = major: 7.8 min, minor: 10.5 min. 86% ee. $[\alpha]_D^{29}$ = 12° (c 0.6, CH_2Cl_2).

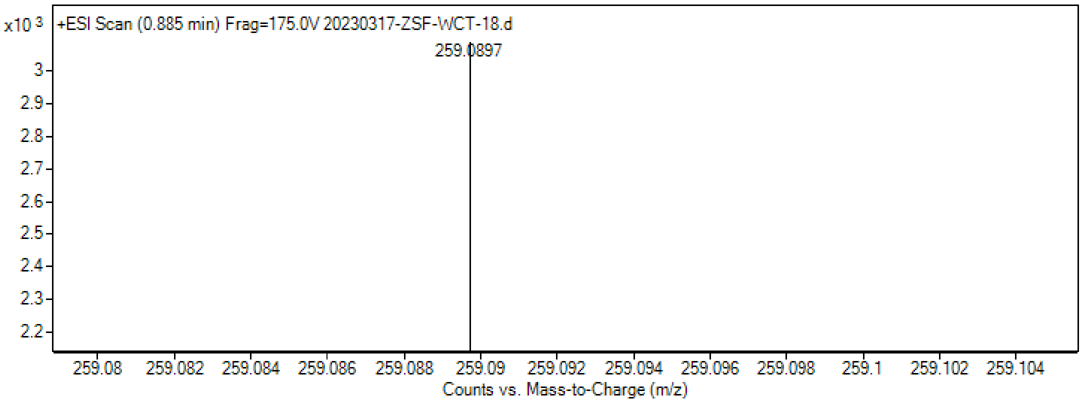

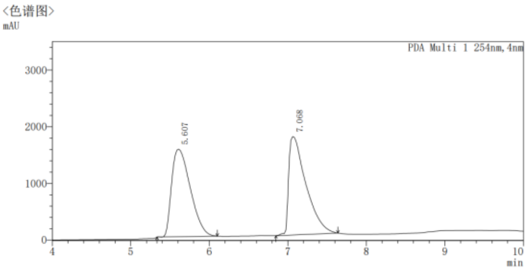

<峰表>

PDA Ch1 254nm

峰号	保留时间	面积	高度	高度%	面积%
1	5.607	25556948	1546416	47.111	49.027
2	7.068	26571209	1736075	52.889	50.973
总计		52128157	3282490	100.000	100.000

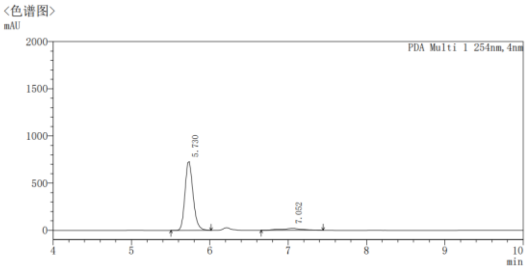

<峰表>

PDA Ch1 254nm

峰号	保留时间	面积	高度	高度%	面积%
1	5.730	5212611	728746	97.397	93.188
2	7.052	381017	19474	2.603	6.812
总计		5593628	748220	100.000	100.000

(1*S*,7*S*)-1-(difluoromethyl)-7-methyl-4-tosyl-4-azabicyclo[5.1.0]oct-2-ene (2ag)

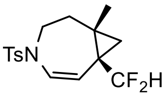

Colorless oil, 14 mg, 88% yield, Rf = 0.2 (PE: EtOAc = 30: 1). **^{1}H NMR** (400 MHz, $CDCl_3$) δ 7.68 (d, *J* = 8.4 Hz, 2H), 7.33 (d, *J* = 8.0 Hz, 2H), 6.54 (dd, *J* = 10.0, 1.1 Hz, 1H), 5.60 – 5.01 (m, 1H), 4.94 (d, *J* = 10.0 Hz, 1H), 3.80 (d, *J* = 7.6 Hz, 1H), 3.51 – 3.04 (m, 1H), 2.44 (s, 3H), 2.16 – 1.88 (m, 1H), 1.50 (dd, *J* = 15.4, 9.9 Hz, 1H), 1.17 (s, 3H), 0.89 (dd, *J* = 5.1, 1.5 Hz, 1H), 0.72 (s, 1H). **^{13}C NMR** (101 MHz, Chloroform-*d*) δ 143.88, 135.38, 129.80, 126.96, 126.25, 116.78 (t, *J* = 238.4 Hz), 104.41, 44.66, 35.86, 28.02 (t, *J* = 22.5 Hz), 26.67 (d, *J* = 4.8 Hz), 25.67 (d, *J* = 5.9 Hz). 21.58, 19.62. **^{19}F NMR** (376 MHz, $CDCl_3$) δ -109.55 (d, *J* = 278.9 Hz), -120.50 (d, *J* = 279.1 Hz). **HRMS**: Calculation for $C_{16}H_{19}F_2NO_2S$, $[M+Na]^+$, 350.0997 Found: 350.1003. HPLC: ODH

column, 99:1 hexane: isopropanol, 1.0 mL/min, t_R = major: 17.7 min, minor: 10.3 min. 50% ee. $[\alpha]_D^{26}$= 117° (c 0.2, CH_2Cl_2).

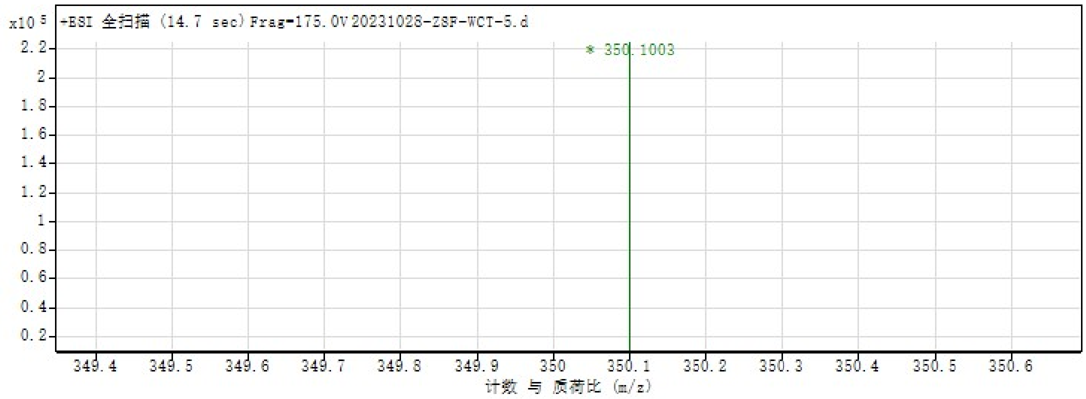

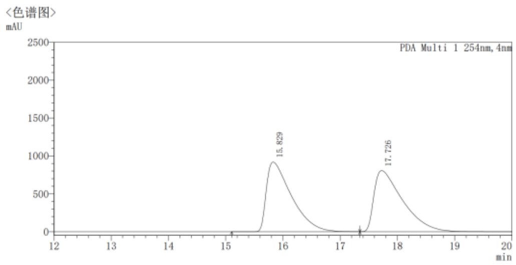

<峰表>
PDA Ch1 254nm

峰号	保留时间	面积	高度	高度%	面积%
1	15.829	28097811	920976	53.253	49.900
2	17.726	28210351	808453	46.747	50.100
总计		56308162	1729429	100.000	100.000

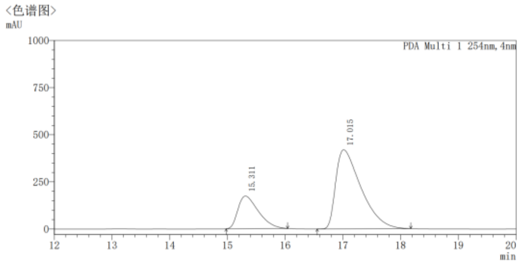

<峰表>
PDA Ch1 254nm

峰号	保留时间	面积	高度	高度%	面积%
1	15.311	4274000	173191	29.216	25.013
2	17.015	12812844	419614	70.784	74.987
总计		17086845	592806	100.000	100.000

(1*R*,6*S*)-6-(fluoromethyl)-1-methyl-3-tosyl-3-azabicyclo[4.1.0]hept-4-ene (2aj)

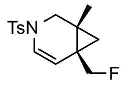

Colorless oil, 13 mg, 93% yield, Rf = 0.2 (PE: EtOAc = 10: 1). **^{1}H NMR** (400 MHz, Chloroform-*d*) δ 7.67 (d, *J* = 8.3 Hz, 2H), 7.34 (d, *J* = 8.0 Hz, 2H), 6.41 (d, *J* = 8.0 Hz, 1H), 5.39 (d, *J* = 8.0 Hz, 1H), 4.60 (dd, *J* = 49.3, 10.1 Hz, 1H), 4.22 (dd, *J* = 47.9, 10.1 Hz, 1H), 3.85 (d, *J* = 11.4 Hz, 1H), 2.73 (d, *J* = 11.3 Hz, 1H), 2.44 (s, 3H), 1.25 (s, 3H), 0.97 (t, *J* = 4.8 Hz, 1H), 0.70 (d, *J* = 4.9 Hz, 1H). **^{13}C NMR** (101 MHz, $CDCl_3$) δ 143.93, 134.74, 129.88, 127.04, 122.20, 113.14, 86.20 (d, *J* = 169.5 Hz), 46.35, 31.16 (d, *J* = 3.6 Hz), 23.76 (d, *J* = 8.6 Hz), 23.22 (d, *J* = 23.7 Hz), 21.56, 17.34. **^{19}F NMR** (376 MHz, $CDCl_3$) δ -213.49. **HRMS**: Calculation for $C_{14}H_{14}F_2O$, $[M+H]^+$, 296.1115 Found: 296.1105. HPLC: INC column, 95:5 hexane: isopropanol, 1.0 mL/min, t_R = major: 22.9 min, minor: 21.5 min. 90% ee. $[\alpha]_D^{29}$ = 34° (c 0.6, CH_2Cl_2).

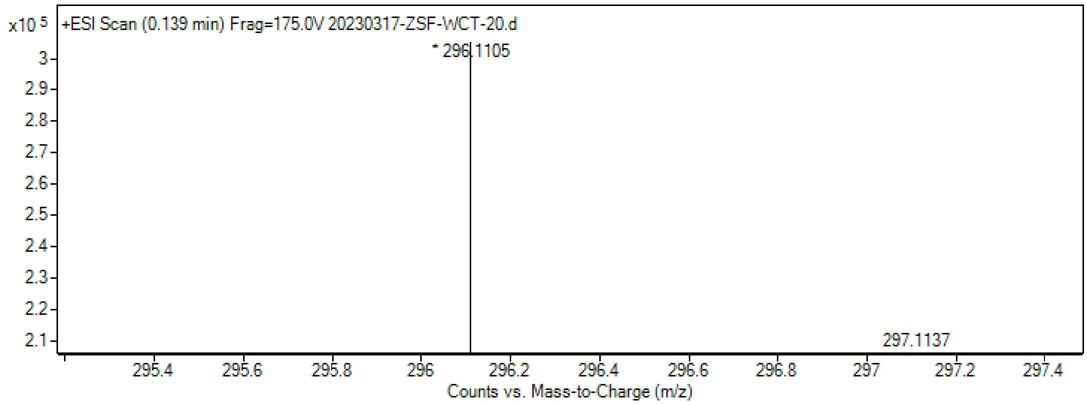

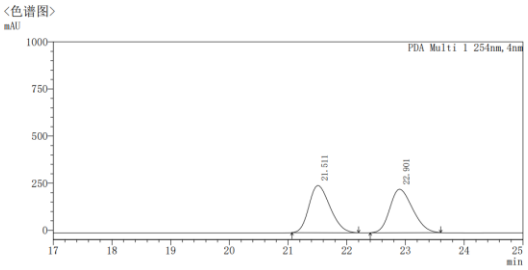

<峰表>
PDA Ch1 254nm

峰号	保留时间	面积	高度	高度%	面积%
1	21.511	6252450	250350	52.037	50.125
2	22.901	6221382	230751	47.963	49.875
总计		12473831	481101	100.000	100.000

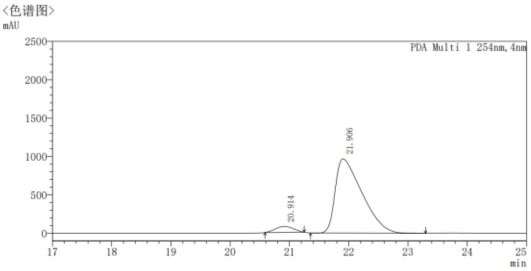

<峰表>
PDA Ch1 254nm

峰号	保留时间	面积	高度	高度%	面积%
1	20.914	1610235	75929	7.280	5.048
2	21.906	30289447	967038	92.720	94.952
总计		31899681	1042967	100.000	100.000

(1*R*,6*R*)-1-methyl-3-tosyl-3-azabicyclo[4.1.0]hept-4-ene (2ah)

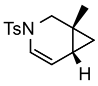

Colorless oil, 11 mg, 84% yield, Rf = 0.2 (PE: EtOAc = 20: 1). **^{1}H NMR** (500 MHz, Chloroform-*d*) δ 7.65 (d, *J* = 8.1 Hz, 2H), 7.31 (d, *J* = 8.1 Hz, 2H), 6.28 (d, *J* = 8.0 Hz, 1H), 5.40 (dd, *J* = 7.9, 5.6 Hz, 1H), 3.84 (d, *J* = 11.4 Hz, 1H), 2.73 (d, *J* = 11.3 Hz, 1H), 2.42 (s, 3H), 1.11 (s, 3H), 0.92 (dt, *J* = 9.2, 5.0 Hz, 1H), 0.68 – 0.59 (m, 1H), 0.56 (t, *J* = 4.3 Hz, 1H). **^{13}C NMR** (126 MHz, $CDCl_3$) δ 143.60, 135.05, 129.74, 127.01, 120.32, 112.67, 45.98, 25.73, 21.94, 21.53, 20.25, 15.92. HPLC: INA column, 97:3 hexane: isopropanol, 1.0 mL/min, t_R = major: 10.5 min, minor: 23min. 43% ee. $[\alpha]_D^{29}$ = 34° (c 0.6, CH_2Cl_2).

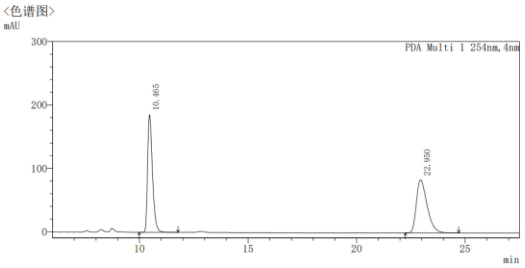

<峰表>
PDA Ch1 254nm

峰号	保留时间	面积	高度	面积%	高度%
1	10.465	2893688	185512	50.140	68.887
2	22.950	2877545	83789	49.860	31.113
总计		5771234	269301	100.000	100.000

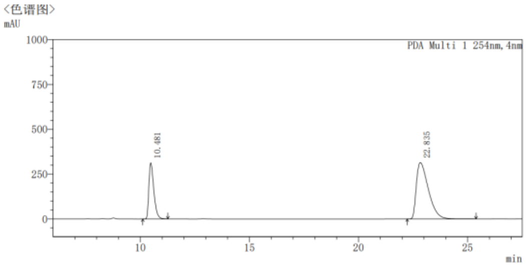

<峰表>
PDA Ch1 254nm

峰号	保留时间	面积	高度	高度%	面积%
1	10.481	4928153	313253	49.859	28.988
2	22.835	12072486	315026	50.141	71.012
总计		17000640	628279	100.000	100.000

(1R,6S)-6-(difluoromethyl-d)-1-methyl-3-tosyl-3-azabicyclo[4.1.0]hept-4-ene (2aq)

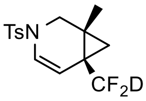

Colorless oil, 15 mg, 93% yield, Rf = 0.2 (PE: EtOAc = 20: 1). **^{1}H NMR** (400 MHz, CDCl3) δ 7.67 (d, J = 8.4 Hz, 2H), 7.59 – 7.25 (m, 2H), 6.48 (dd, J = 8.2, 1.1 Hz, 1H), 5.50 (d, J = 8.2 Hz, 1H), 4.08 – 3.66 (m, 1H), 2.72 (dd, J = 11.5, 1.3 Hz, 1H), 2.45 (s, 3H),

1.27 (s, 3H), 1.06 (d, J = 4.6 Hz, 1H), 0.97 (d, J = 5.4 Hz, 1H). **^{13}C NMR** (101 MHz, Chloroform-*d*) δ 144.12, 134.64, 129.96, 127.03, 122.95, 107.11 (t, *J* = 4.1 Hz), 45.96, 30.46 (d, *J* = 6.1 Hz), 25.02 (t, *J* = 25.6 Hz), 22.84 (d, *J* = 6.4 Hz), 21.57, 17.12. **^{19}F NMR** (376 MHz, $CDCl_3$) δ -112.03 (dt, *J* = 284.7, 8.2Hz)

, -120.43 (dt, *J* = 276.6, 8.9 Hz). **HRMS**: Calculation for $C_{15}H_{16}DF_2NO_2S$, $[M+H]^+$, 315.1084 Found: 315.1088. HPLC: INC column, 99:9 hexane: isopropanol, 1.0 mL/min, t_R = major: 40.9 min, minor: 38.2 min. 97% ee. $[\alpha]_D^{26}$ = -141° (c 0.2, CH_2Cl_2).

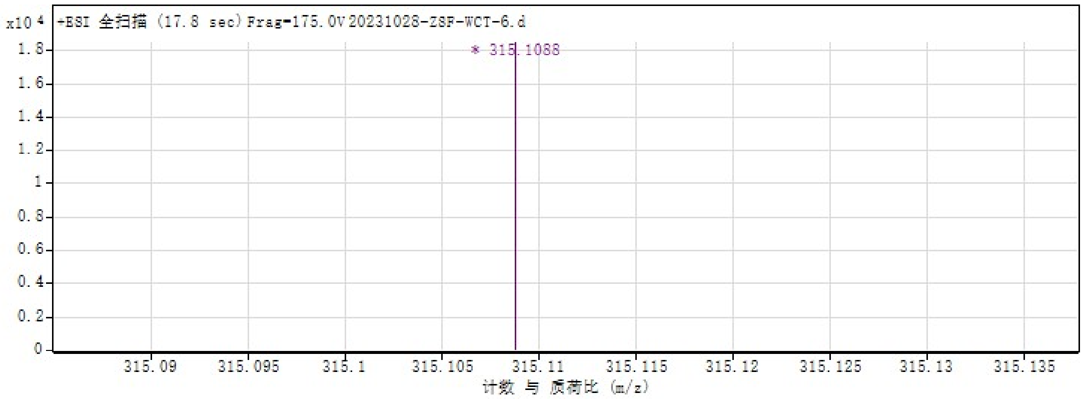

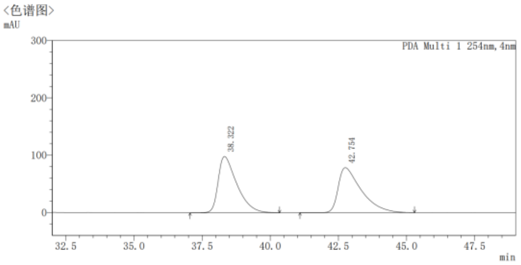

<峰表>
PDA Ch1 254nm

峰号	保留时间	面积	高度	高度%	面积%
1	38.322	4692880	98254	55.546	50.177
2	42.754	4659710	78634	44.454	49.823
总计		9352590	176888	100.000	100.000

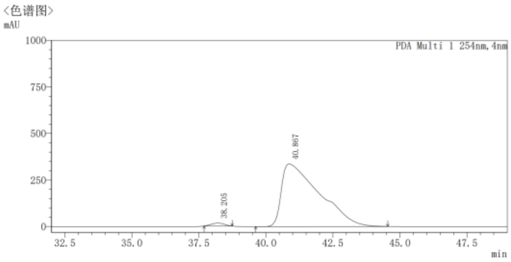

<峰表>
PDA Ch1 254nm

峰号	保留时间	面积	高度	高度%	面积%
1	38.205	539874	16568	4.692	1.609
2	40.867	33008132	336528	95.308	98.391
总计		33548006	353097	100.000	100.000

1ah and **1ai** were adapted to this asymmetric reaction even at elevated temperature.

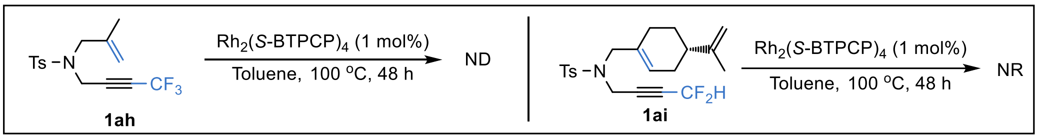

4. The derivatizations of difluoromethylated cyclopropane compounds[5]

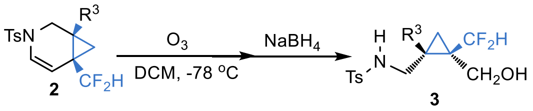

A solution of **2** (0.1 mmol) in CH_2Cl_2 (3 mL) was cooled to -78 °C and ozone was bubbled into the reaction mixture until a pale blue color was observed (ca. 5 min). The excess ozone was discharged by nitrogen stream for 5 min, and then $NaBH_4$ (1.5 equiv.) were added. The mixture was stirred at room temperature for 30 min at room temperature, and then saturated aqueous NH_4Cl was added. The mixture was extracted with CH_2Cl_2 and the organic layer was dried over $MgSO_4$, filtered, and concentrated on a rotary evaporator. The residue was subjected to preparative TLC on silica gel with PE: EtOAc (3/1-2/1)

to give **3**.

N-(((1*R*,2*R*)-2-(difluoromethyl)-2-(hydroxymethyl)cyclopropyl)methyl)-4-methylbenzenesulfonamide (3a)

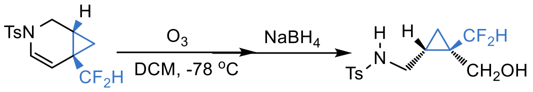

2ae, 88% ee **3**, 87%, 88% ee

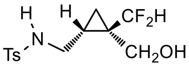

Colorless oil, 27 mg, 87% yield, Rf = 0.2 (PE: EtOAc = 2: 1). **^{1}H NMR** (400 MHz, Chloroform-*d*) δ 7.77 (d, *J* = 8.2 Hz, 2H), 7.33 (d, *J* = 8.0 Hz, 2H), 5.93 (d, *J* = 9.4 Hz, 1H), 5.69 (t, *J* = 57.4 Hz, 1H), 4.30 (d, *J* = 12.4 Hz, 1H), 3.63 – 3.52 (m, 1H), 3.20 (d, *J* = 12.9 Hz, 1H), 2.65 (s, 1H), 2.56 (ddd, *J* = 13.7, 10.8, 3.0 Hz, 1H), 2.44 (s, 3H), 1.36 (dt, *J* = 9.8, 5.4 Hz, 1H), 0.99 (dd, *J* = 9.0, 5.8 Hz, 1H), 0.42 (t, *J* = 5.7 Hz, 1H). **^{13}C NMR** (101 MHz, $CDCl_3$) δ 143.56, 136.89, 129.80, 127.05, 118.19 (t, *J* = 241.8 Hz), 60.55, 43.11, 28.75 (t, *J* = 21.9 Hz), 21.53, 18.94, 10.60. **^{19}F NMR** (376 MHz, Chloroform-*d*) δ -118.33 (d, *J* = 280.3 Hz), -125.25 (d, *J* = 280.2 Hz). **HRMS**: Calculation for $C_{13}H_{17}F_2NO3S$, $[M+Na^+]^+$, 328.0789 Found: 328.0793. HPLC: INC column, 85:15 hexane: isopropanol, 0.85 mL/min, t_R = major: 34.5 min, minor: 37.2 min. 88% ee. $[\alpha]_D^{29}$ = 3° (c 0.6, CH_2Cl_2).

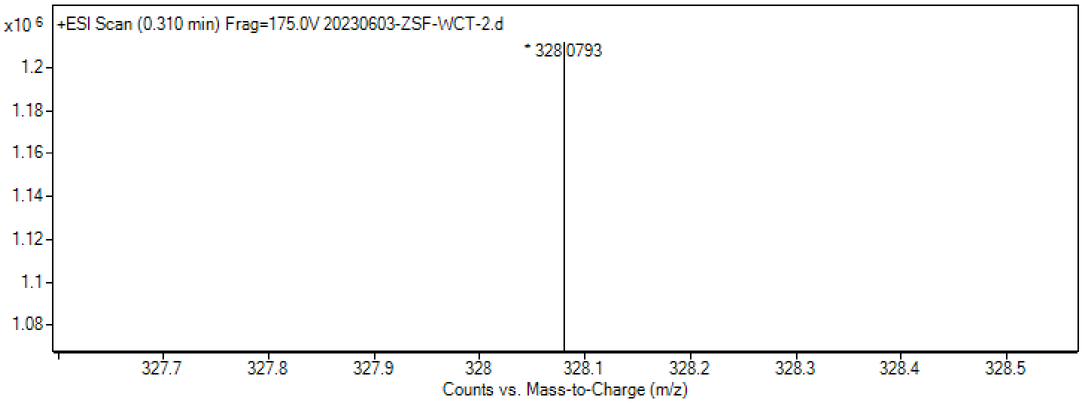

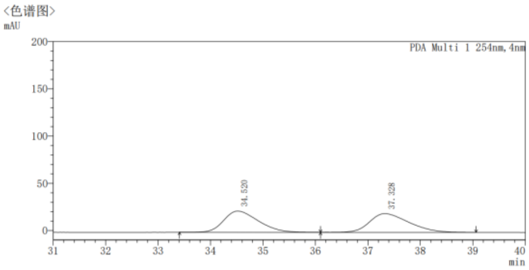

<峰表>
PDA Ch1 254nm

峰号	保留时间	面积	高度	高度%	面积%
1	34.520	1030938	22485	53.191	50.068
2	37.328	1028132	19788	46.809	49.932
总计		2059070	42273	100.000	100.000

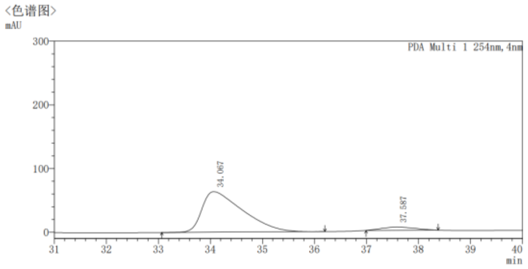

<峰表>
PDA Ch1 254nm

峰号	保留时间	面积	高度	高度%	面积%
1	34.067	3454389	63692	92.800	93.957
2	37.587	222171	4942	7.200	6.043
总计		3676560	68634	100.000	100.000

N-(((1*R*,2*R*)-2-(difluoromethyl)-1-hexyl-2-(hydroxymethyl)cyclopropyl)methyl)-4-methylbenzenesulfonamide (3b)

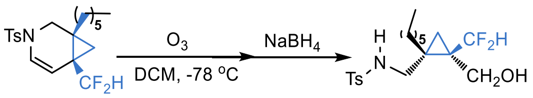

2d, 95% ee **3**, 95%, 97% ee

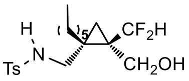

Colorless oil, 37 mg, 95% yield, Rf = 0.2 (PE: EtOAc = 2: 1). ^{1}H NMR (500 MHz, Chloroform-*d*) δ 7.75 (d, *J* = 8.0 Hz, 2H), 7.30 (d, *J* = 7.9 Hz, 2H), 6.01 (d, *J* = 10.2 Hz, 1H), 5.65 (t, *J* = 55.2 Hz, 1H), 4.47 (dd, *J* = 13.3, 3.1 Hz, 1H), 3.73 – 3.46 (m, 1H), 3.21 – 2.82 (m, 1H), 2.42 (s, 3H), 2.38 (s, 1H), 2.36 – 2.20 (m, 1H), 2.08 – 1.87 (m, 1H), 1.67 (s, 1H), 1.44 (s, 1H), 1.31 – 1.17 (m, 6H), 1.11 – 1.02 (m, 1H), 0.88 (t, *J* = 7.0 Hz, 3H), 0.78 (d, *J* = 6.1 Hz, 1H), 0.59 (d, *J* = 5.3 Hz, 1H). ^{13}C NMR (126 MHz, $CDCl_3$) δ 143.24, 136.70, 129.68, 127.09, 118.88 (t, *J* = 239.8 Hz), 59.24, 46.16, 32.96 – 32.49 (m), 31.71, 29.96, 29.39, 29.28 (d, *J* = 5.5 Hz), 26.39, 22.63, 21.51, 19.48 (d, *J* = 6.4 Hz), 14.07. ^{19}F NMR (471 MHz, Chloroform-*d*) δ -110.24 (d, *J* = 283.1 Hz), -117.88 (d, *J* = 283.1 Hz). **HRMS**: Calculation for $C_{19}H_{29}F_2NO_3S$, $[M+Na]^+$, 412.1728 Found: 412.1713. HPLC: INA column, 85:15 hexane: isopropanol, 0.80 mL/min, t_R = major: 8.9 min, minor: 9.8 min. 97% ee. $[\alpha]_D^{29}$ = 82° (c 1.2, CH_2Cl_2).

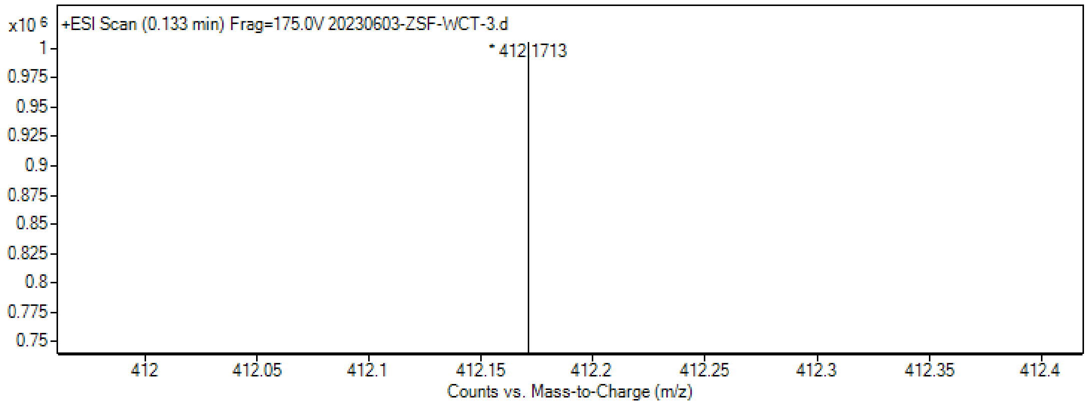

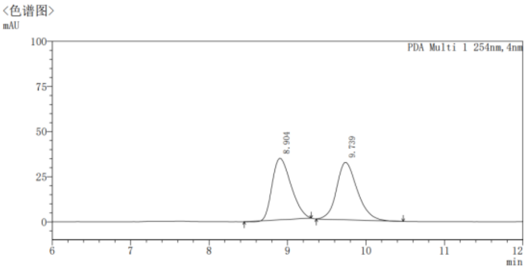

<峰表>

PDA Ch1 254nm

峰号	保留时间	面积	高度	高度%	面积%
1	8.904	570883	34133	51.775	49.412
2	9.739	584466	31792	48.225	50.588
总计		1155348	65925	100.000	100.000

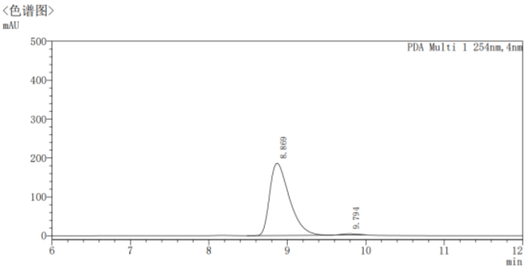

<峰表>

PDA Ch1 254nm

峰号	保留时间	面积	高度	高度%	面积%
1	8.869	3240943	186089	98.264	98.666
2	9.794	43825	3287	1.736	1.334
总计		3284768	189376	100.000	100.000

N-(((1*S*,2*R*)-2-(difluoromethyl)-2-(hydroxymethyl)-[1,1'-bi(cyclopropan)]-1-yl)methyl)-4-methylbenzenesulfonamide (3c)

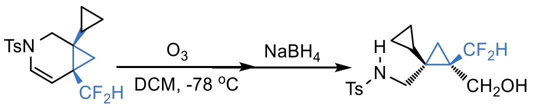

2e, 97% ee **3c**, 77%, 94% ee

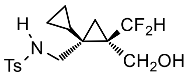

Colorless oil, 24 mg, 77% yield, Rf = 0.2 (PE: EtOAc = 2: 1). **^{1}H NMR** (500 MHz, Chloroform-*d*) δ 7.57 (d, *J* = 8.0 Hz, 2H), 7.11 (d, *J* = 8.0 Hz, 2H), 5.98 (d, *J* = 10.7 Hz, 1H), 5.70 (d, *J* = 55.1 Hz, 1H), 4.35 (d, *J* = 13.4 Hz, 1H), 3.55 – 3.22 (m, 1H), 2.97 – 2.64 (m, 1H), 2.51 – 2.30 (m, 1H), 2.24 (s, 3H), 2.18 (s, 1H), 1.23 (p, *J* = 7.5 Hz, 1H), 0.46 – 0.35 (m, 3H), 0.20 (t, *J* = 5.7 Hz, 1H), -0.00 (d, *J* = 5.3 Hz, 2H). **^{13}C NMR** (126 MHz, $CDCl_3$) δ 143.16, 136.94, 129.64, 127.09, 119.64 (t, *J* = 238.3 Hz), 61.57, 49.02, 33.60 (t, *J* = 24.5 Hz), 30.35 (d, *J* = 6.5 Hz), 21.50, 16.24, 11.47, 6.18, 4.06. **^{19}F NMR** (471 MHz, Chloroform-*d*) δ -112.39 (d, *J* = 284.2 Hz), -118.28 (d, *J* = 284.1 Hz). **HRMS**: Calculation for $C_{16}H_{21}F_2NO_3S$, $[M+Na^+]^+$, 368.1102 Found: 368.1093. HPLC: INA column, 85:15 hexane: isopropanol, 0.80 mL/min, t_R = major: 12.3 min, minor: 17.5 min. 94% ee. $[\alpha]_D^{29}$ = 12° (c 0.6, CH_2Cl_2).

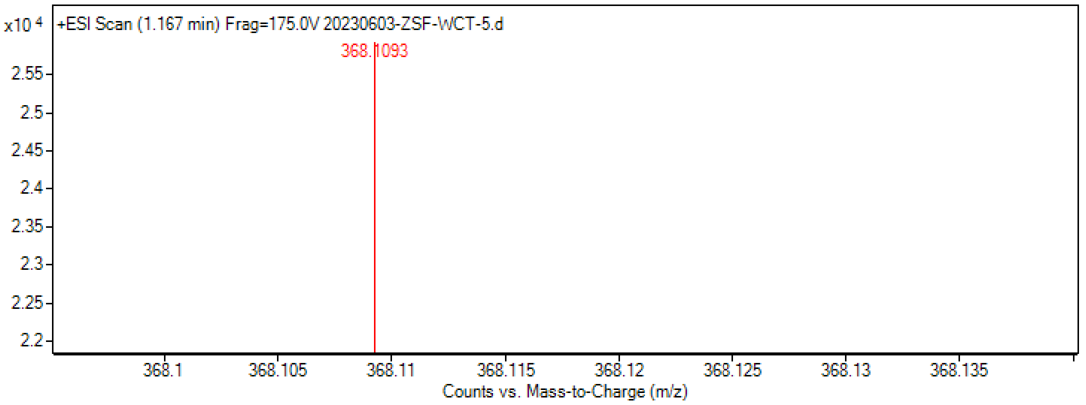

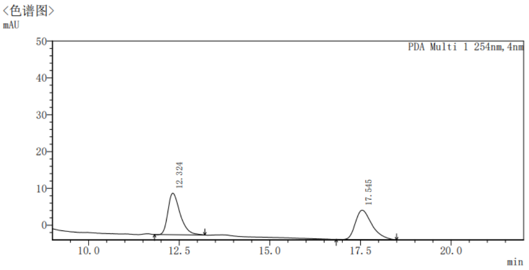

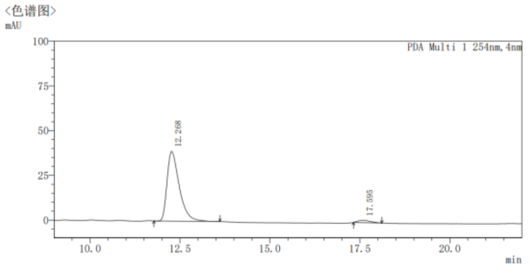

<峰表>

PDA Ch1 254nm

峰号	保留时间	面积	高度	高度%	面积%
1	12.324	265040	11282	58.486	50.832
2	17.545	256359	8008	41.514	49.168
总计		521400	19290	100.000	100.000

<峰表>

PDA Ch1 254nm

峰号	保留时间	面积	高度	高度%	面积%
1	12.268	919460	39030	96.897	96.830
2	17.595	30100	1250	3.103	3.170
总计		949560	40280	100.000	100.000

N-(((1*S*,2*R*)-1-(((tert-butyldimethylsilyl)oxy)methyl)-2-(difluoromethyl)-2-(hydroxymethyl)cyclopropyl)methyl)-4-methylbenzenesulfonamide (3d)

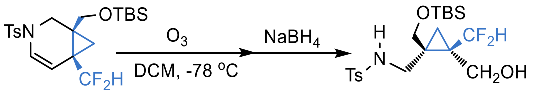

2l, 96% ee **3d**, 88%, 95% ee

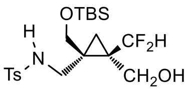

Colorless oil, 40 mg, 88% yield, Rf = 0.2 (PE: EtOAc = 2: 1). **^{1}H NMR** (500 MHz, Chloroform-*d*) δ 7.69 (d, *J* = 8.1 Hz, 2H), 7.27 (d, *J* = 8.0 Hz, 2H), 6.38 – 5.64 (m, 2H), 4.34 (d, *J* = 13.0 Hz, 1H), 3.88 – 3.54 (m, 2H), 3.43 (dd, *J* = 12.4, 8.5 Hz, 1H), 3.20 – 2.96 (m, 1H), 2.66 (dd, *J* = 12.9, 4.5 Hz, 1H), 2.39 (s, 4H), 1.08 (d, *J* = 5.9 Hz, 1H), 0.83 (s, 9H), 0.58 (t, *J* = 5.1 Hz, 1H), 0.00 (d, *J* = 2.7 Hz, 6H). **^{13}C NMR** (126 MHz, $CDCl_3$) δ 143.48, 136.28, 129.75, 127.14, 117.68 (t, *J* = 239.8 Hz), 63.88, 61.72, 47.52, 32.84 (t, *J* = 23.7 Hz), 25.80, 21.52, 18.08, 16.57, -5.66, -5.70. ^{13}C NMR (126 MHz, Chloroform-*d*) δ 117.68 (t, *J* = 239.8 Hz), 32.84 (t, *J* = 23.7 Hz). **^{19}F NMR** (471 MHz, Chloroform-*d*) δ -111.86 (d, *J* = 284.4 Hz), -117.29 (d, *J* = 284.4 Hz). **HRMS**: Calculation for $C_{20}H_{33}F_2NO_4SSi$, $[M+Na]^+$, 472.1760 Found: 472.1747. HPLC: INC column, 85:15 hexane: isopropanol, 0.80 mL/min, t_R = major: 14.1 min, minor: 23.4 min. 95% ee. $[\alpha]_D^{29}$ = 13° (c 0.3, CH_2Cl_2).

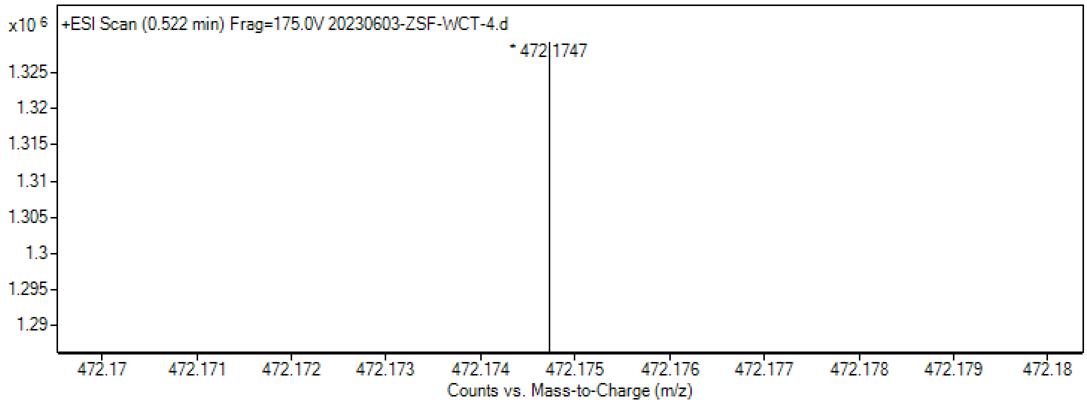

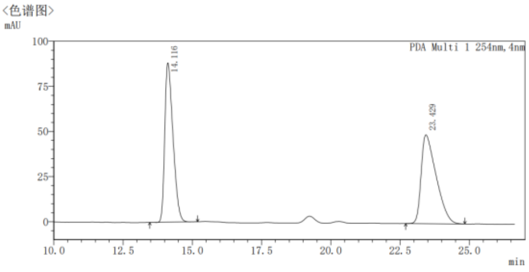

<峰表>

PDA Ch1 254nm

峰号	保留时间	面积	高度	高度%	面积%
1	14.116	1826698	88310	64.187	49.651
2	23.429	1852368	49271	35.813	50.349
总计		3679066	137581	100.000	100.000

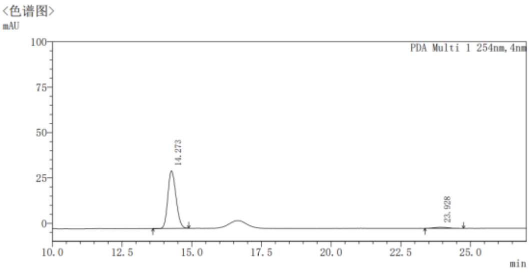

<峰表>

PDA Ch1 254nm

峰号	保留时间	面积	高度	高度%	面积%
1	14.273	643733	31823	98.377	97.488
2	23.928	16591	525	1.623	2.512
总计		660323	32348	100.000	100.000

N-(((2*R*)-2-(difluoromethyl)-2-(hydroxymethyl)-1-(3-methoxyphenyl)cyclopropyl)methyl)-4-methylbenzenesulfonamide (3e)

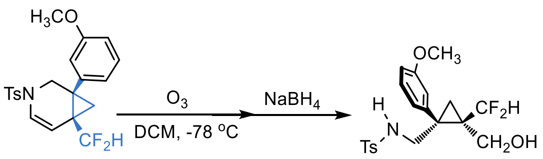

2w, 93% ee

3e, 63%, 94% ee

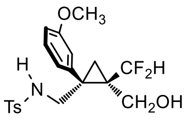

White solid, 26 mg, 63% yield, m.p. = 64.9 - 65.1 °C, Rf = 0.2 (PE: EtOAc = 2: 1). **^{1}H NMR** (500 MHz, Chloroform-*d*) δ 7.50 (d, *J* = 8.0 Hz, 2H), 7.17 – 7.10 (m, 2H), 6.81 (d, *J* = 7.6 Hz, 1H), 6.77 – 6.71 (m, 2H), 5.55 (d, *J* = 9.5 Hz, 1H), 4.84 (t, *J* = 54.4 Hz, 1H), 4.48 (d, *J* = 13.3 Hz, 1H), 3.70 (s, 3H), 3.35 (d, *J* = 11.5 Hz, 1H), 3.28 (dd, *J* = 13.4, 6.5 Hz, 1H), 3.03 (dd, *J* = 13.3, 3.2 Hz, 1H), 2.39 – 2.34 (m, 1H), 2.32 (s, 3H), 1.23 – 1.17 (m, 2H), 0.99 (t, *J* = 5.4 Hz, 1H). **^{13}C NMR** (126 MHz, $CDCl_3$) δ 159.72, 143.18, 138.60, 136.78, 129.85, 129.14, 126.90, 122.33, 119.12 (t, *J* = 239.2 Hz), 115.75, 113.53, 59.90, 56.14, 49.78, 35.99, 33.94 (t, *J* = 24.9 Hz), 21.48, 18.61. **^{13}C NMR** (126 MHz, Chloroform-*d*) δ 119.12 (t, *J* = 239.2 Hz), 33.94 (t, *J* = 24.9 Hz). ^{19}F NMR (471 MHz, Chloroform-*d*) δ -115.19 (d, *J* = 281.5 Hz), -119.46 (d, *J* = 281.5 Hz). **HRMS**: Calculation for $C_{20}H_{23}F_2NO_4S$, $[M+H]^+$, 412.1389 Found: 412.1384. HPLC: OD-H column, 85:15 hexane: isopropanol, 0.80 mL/min, t_R = major: 16.4 min, minor: 14.7 min. 94% ee. $[\alpha]_D^{29}$ = 7° (c 0.3, CH_2Cl_2).

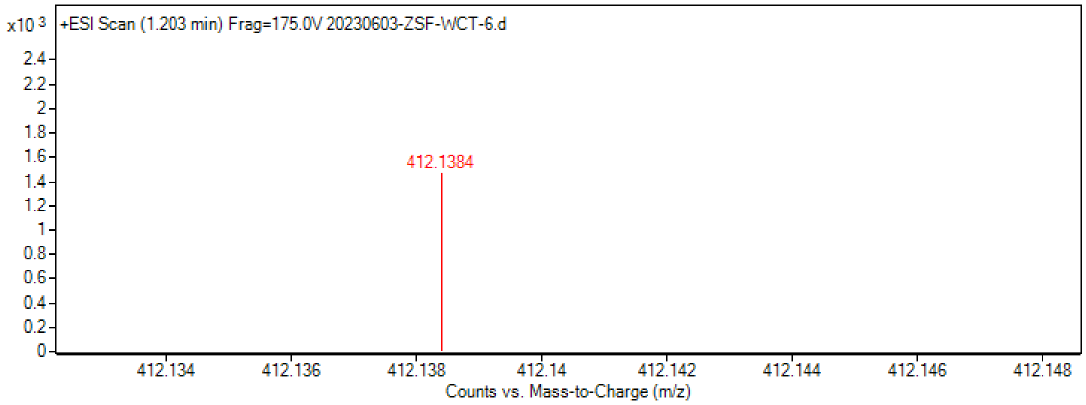

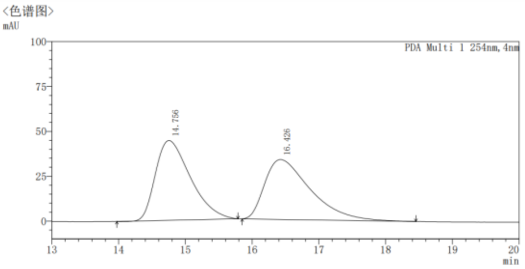

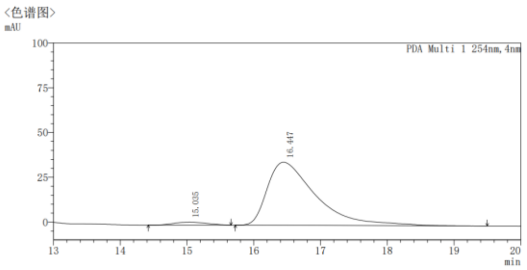

<峰表>
PDA Ch1 254nm

峰号	保留时间	面积	高度	高度%	面积%
1	14.756	1624205	44480	57.103	50.529
2	16.426	1590169	33413	42.897	49.471
总计		3214374	77893	100.000	100.000

<峰表>
PDA Ch1 254nm

峰号	保留时间	面积	高度	高度%	面积%
1	15.035	55577	1641	4.450	3.031
2	16.447	1778308	35240	95.550	96.969
总计		1833885	36881	100.000	100.000

N-(((2*R*)-1-(4-chlorophenyl)-2-(difluoromethyl)-2-(hydroxymethyl)cyclopropyl)methyl)-4-methylbenzenesulfonamide (3f)

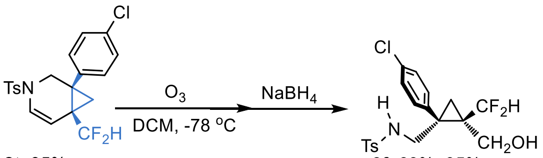

2t, 95% ee **3f**, 63%, 95% ee

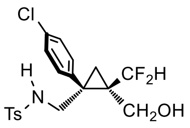

White solid, 26 mg, 63% yield, m.p. = 84.8 - 85.1 °C, Rf = 0.2 (PE: EtOAc = 2: 1). **^{1}H NMR** (400 MHz, Chloroform-*d*) δ 7.55 (d, *J* = 8.2 Hz, 2H), 7.22 (t, *J* = 6.4 Hz, 6H), 5.96 (dd, *J* = 9.8, 3.2 Hz, 1H), 5.17 – 4.74 (m, 1H), 4.59 (d, *J* = 13.2 Hz, 1H), 3.44 (ddd, *J* = 12.7, 9.9, 2.4 Hz, 1H), 3.33 (dd, *J* = 13.4, 6.9 Hz, 1H), 3.06 (dd, *J* = 13.5, 3.2 Hz, 1H), 2.60 (s, 1H), 2.43 (s, 3H), 1.26 (d, *J* = 7.2 Hz, 1H), 1.17 – 0.94 (m, 1H). **^{13}C NMR** (101 MHz, $CDCl_3$) δ 143.34, 136.73, 135.71, 133.91, 131.63, 129.58, 128.83, 126.83, 118.83 (t, *J* = 239.0 Hz), 60.52, 49.89, 34.46 (d, *J* = 7.1 Hz), 34.34 – 33.24 (m), 22.66, 18.53 (d, *J* = 6.6 Hz). **^{19}F NMR** (376 MHz, Chloroform-*d*) δ -115.11 (d, *J* = 282.0 Hz), -119.31 (d, *J* = 281.9 Hz). **HRMS**: Calculation for $C_{19}H_{20}ClF_2NO_3S$, $[M+H]^+$, 416.0893 Found: 416.0885. HPLC: INA column, 85:15 hexane: isopropanol, 0.80 mL/min, t_R = major: 16.3 min, minor: 15 min. 91% ee. $[\alpha]_D^{29}$ = 38° (c 0.3, CH_2Cl_2).

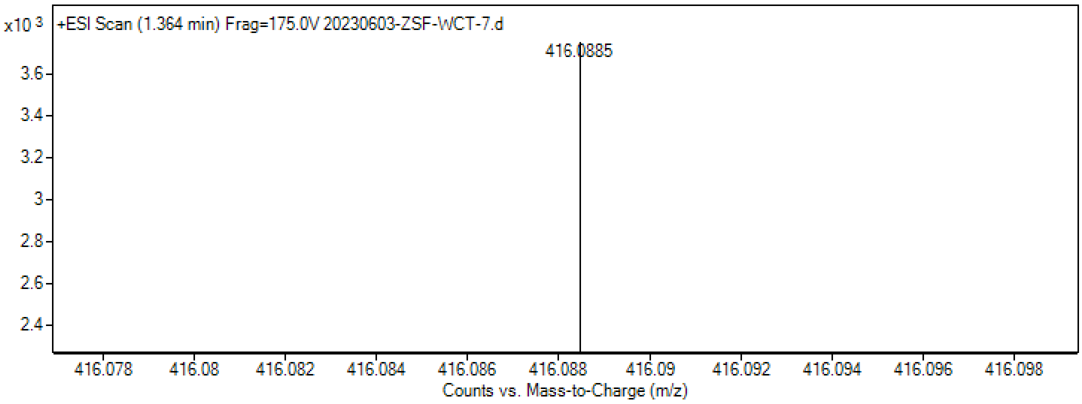

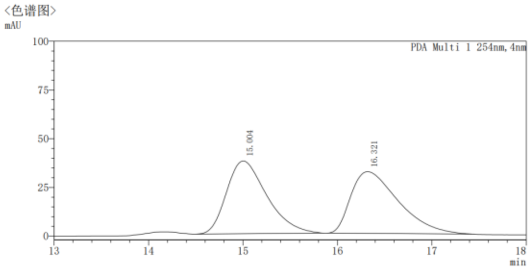

〈峰表〉
PDA Ch1 254nm

峰号	保留时间	面积	高度	高度%	面积%
1	15.004	1106703	37379	54.129	49.915
2	16.321	1110478	31676	45.871	50.085
总计		2217181	69055	100.000	100.000

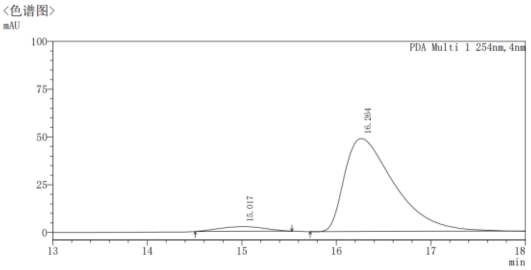

〈峰表〉
PDA Ch1 254nm

峰号	保留时间	面积	高度	高度%	面积%
1	15.017	82293	2491	4.872	4.389
2	16.264	1792803	48632	95.128	95.611
总计		1875096	51123	100.000	100.000

Procedure for gram-scale reaction: To a toluene solution of **1l** (2.5 mmol, 15 mL) in Schlenk tube with a magnetic bar was added Rh_2(*S*-BTPCP)$_4$ (0.2 mol%, 8.9 mg) at 0 °C under N_2. The sealed tube was then stirred at room temperature under nitrogen atmosphere for 60 h. The mixture was then concentrated and the residue was purified by chromatography on silica gel (eluent: PE: EtOAc) to afford the desired product **2l** (92%, 96% ee).

The derivatizations of 2l:

A solution of **2l** (96% ee, 44 mg, 0.10 mmol) in CH_2Cl_2 (1 mL) was cooled to –50 °C, and

allyltrimethylsilane (95 μL, 0.60 mmol) and trifluoroacetic acid (31 μL, 0.40 mmol) were added to the solution. The reaction mixture was warmed to -30 °C and stirred at the same temperature for 2 h. The mixture was allowed to warm to room temperature and stirred overnight. Saturated aqueous $NaHCO_3$ was added, and the mixture was extracted with DCM and organic layer was washed with water and brine, dried over $MgSO_4$, filtered, and concentrated on a rotary evaporator. The residue was subjected to preparative TLC on silica gel with hexane/ethyl acetate (10/1) to give **4** as a mixture of two diastereomers.

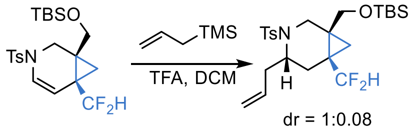

2l, 96% ee **4**, 89%, 96 % ee

(1*S*,4*S*,6*S*)-4-allyl-1-(((tert-butyldimethylsilyl)oxy)methyl)-6-(difluoromethyl)-3-tosyl-3-azabicyclo[4.1.0]heptane (4)

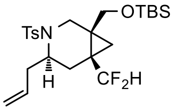

Colorless oil, 45 mg, 89% yield, Rf = 0.2 (PE: EtOAc = 10: 1). **^{1}H NMR** (500 MHz, Chloroform-*d*) δ 7.63 (d, *J* = 8.3 Hz, 2H), 7.26 (d, *J* = 8.0 Hz, 2H), 5.71 – 5.34 (m, 2H), 5.02 – 4.87 (m, 2H), 3.95 – 3.84 (m, 1H), 3.75 (d, *J* = 11.6 Hz, 2H), 3.43 (d, *J* = 11.2 Hz, 1H), 3.11 (d, *J* = 12.8 Hz, 1H), 2.40 (s, 3H), 2.16 (dt, *J* = 15.7, 8.2 Hz, 1H), 2.07 (d, *J* = 14.4 Hz, 1H), 1.93 – 1.85 (m, 1H), 1.68 (dd, *J* = 14.5, 6.2 Hz, 1H), 0.96 – 0.89 (m, 1H), 0.83 (s, 9H), 0.62 (s, 1H), 0.00 (d, *J* = 1.8 Hz, 6H). **^{13}C NMR** (126 MHz, $CDCl_3$) δ 143.33, 137.56, 134.41, 129.70, 126.97, 117.90, δ 116.70 (t, *J* = 240.66 Hz), 63.80, 49.79, 39.69, 33.36, 25.69, 25.15 (d, *J* = 7.3 Hz), 22.53 (t, *J* = 26.7 Hz), 21.53, 18.08, 17.07 (d, *J* = 6.9 Hz), -5.71, -5.74. **^{19}F NMR** (471 MHz, Chloroform-*d*) δ -111.31 (d, *J* = 282.9 Hz), -120.15 (d, *J* = 282.9 Hz). **HRMS**: Calculation for $C_{24}H_{37}F_2NO_3SSi$, $[M+H]^+$, 508.2124 Found: 508.2126. HPLC: INA column, 95:5 hexane: isopropanol, 1.0 mL/min, t_R = major: 5.9 min, minor: 6.6 min. 96% ee. $[\alpha]_D^{29}$ = 15° (c 0.5, CH_2Cl_2).

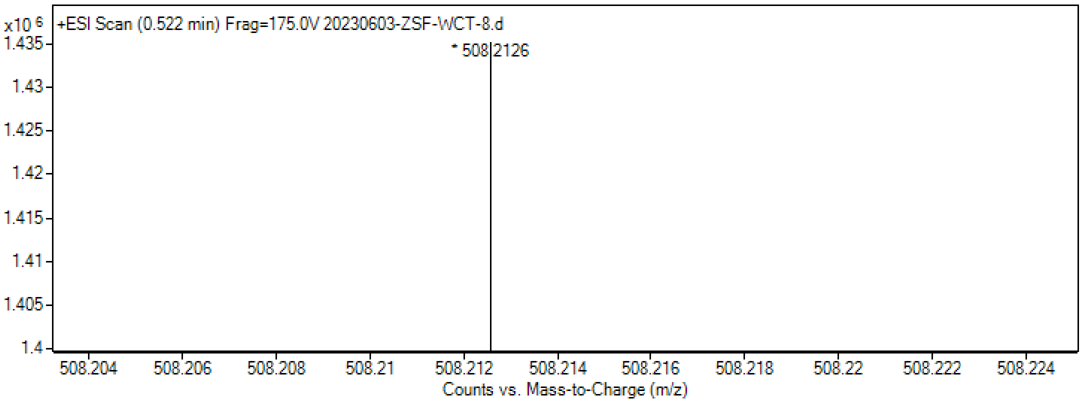

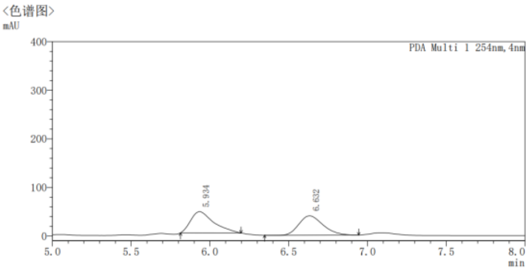

<峰表>
PDA Ch1 254nm

峰号	保留时间	面积	高度	高度%	面积%
1	5.934	445225	43761	52.309	51.326
2	6.632	422213	39898	47.691	48.674
总计		867438	83659	100.000	100.000

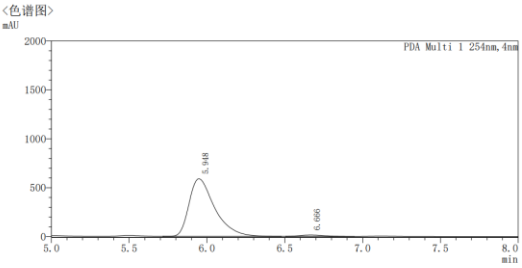

<峰表>
PDA Ch1 254nm

峰号	保留时间	面积	高度	高度%	面积%
1	5.948	6744580	589251	97.793	97.946
2	6.666	141469	13296	2.207	2.054
总计		6886049	602547	100.000	100.000

To a solution **2l** (42 mg, 0.1 mmol) in DCM (2 mL) and NIS (34 mg, 1.5 equiv.) was added at room temperature while stirring. Then $TMSN_3$ (17 mg, 1.5 equiv.) was slowly added. After 12 h, the reaction mixture was quenched with saturated aqueous $Na_2S_2O_3$ and extracted with DCM. Then concentrated in vacuo, and purified by flash column chromatography (silica gel, PE: EtOAc = 15:1) to afford product **5**.

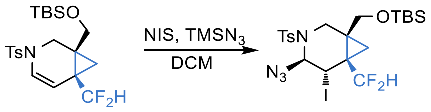

2l, 96% ee **5**, 62%, 94% ee, dr > 20:1

(1*S*,6*S*)-4-azido-1-(((tert-butyldimethylsilyl)oxy)methyl)-6-(difluoromethyl)-5-iodo-3-tosyl-3-azabicyclo[4.1.0]heptane (5)

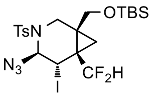

White solid,, 38 mg, 62% yield, m.p. = 44.8 -45.1 °C Rf = 0.2 (PE: EtOAc = 10: 1). **^{1}H NMR** (500 MHz, Chloroform-*d*) δ 7.78 (d, *J* = 8.2 Hz, 2H), 7.35 (d, *J* = 8.1 Hz, 2H), 6.05 (t, *J* = 58.1 Hz, 1H), 5.75 (d, *J* = 2.2 Hz, 1H), 4.53 (d, *J* = 2.2 Hz, 1H), 3.71 (dd, *J* = 11.3, 3.1 Hz, 1H), 3.65 (s, 2H), 3.44 (dd, *J* = 11.2, 2.6 Hz, 1H), 2.44 (s, 3H), 2.02 (s, 1H), 1.19 (d, *J* = 6.7 Hz, 1H), 0.83 (s, 9H), -0.01 (d, *J* = 6.8 Hz, 6H). **^{13}C NMR** (126 MHz, $CDCl_3$) δ 144.67, 133.26, 129.78, 128.21, 117.36 (d, *J* = 243.8 Hz), 63.37 (d, *J* = 7.4 Hz), 42.57, 28.39 (d, *J* = 4.9 Hz), 27.71 – 27.00 (m), 25.70, 24.74 (d, *J* = 5.1 Hz), 21.65, 18.03, 15.04 (d, *J* = 6.3 Hz), -5.70. **^{19}F NMR** (376 MHz, Chloroform-*d*) δ -117.39 (d, *J* = 287.3 Hz), -120.57 (d, *J* = 287.3 Hz). **HRMS**: Calculation for $C_{21}H_{31}F_2IN_4O_3SSi$, $[M+Na]^+$, 635.0797 Found: 635.0775. HPLC: AD-H column, 99.5:0.5 hexane: isopropanol, 0.80 mL/min, t_R = major: 7.8 min, minor: 10.5 min. 94% ee. $[\alpha]_D^{29}$ = 15° (c 0.3, CH_2Cl_2).

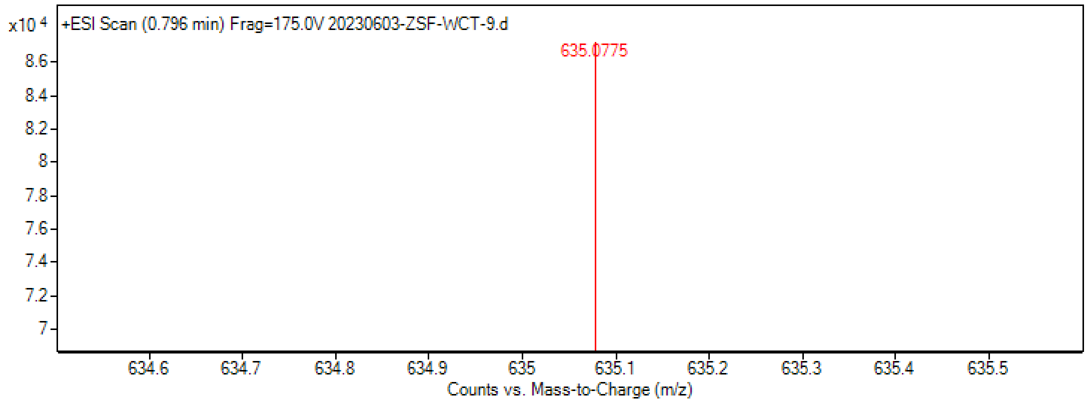

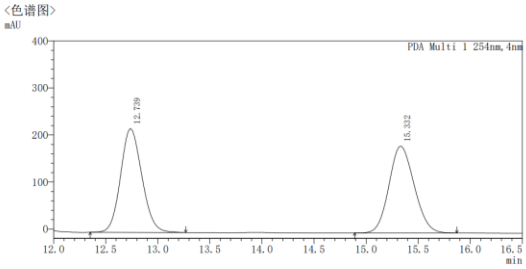

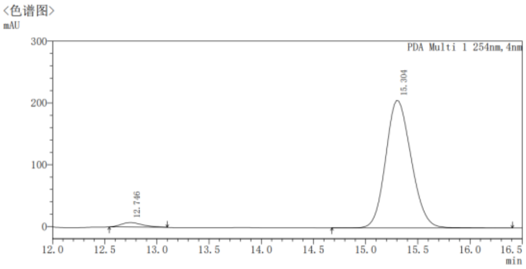

<峰表>
PDA Ch1 254nm

峰号	保留时间	面积	高度	高度%	面积%
1	12.739	3144259	221024	54.490	50.288
2	15.332	3108250	184599	45.510	49.712
总计		6252509	405623	100.000	100.000

<峰表>
PDA Ch1 254nm

峰号	保留时间	面积	高度	高度%	面积%
1	12.746	105063	7324	3.426	2.910
2	15.304	3505113	206472	96.574	97.090
总计		3610175	213796	100.000	100.000

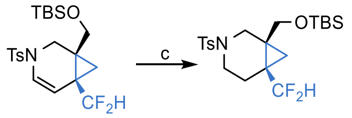

2l, 96% ee　　**6**, 92 %, 95% ee

The substrates **2l** (42 mg, 96% ee, 0.1 mmol) was dissolved in DCM (2 mL) at room temperature, Et_3SiH (2.0 equiv., 0.2 mmol) and TFA (2.0 equiv., 0.2 mmol) were subsequently added into the solution. The reaction was stirred at room temperature overnight. The mixture was purified by silica gel column chromatography to obtain the product **6** (Colorless oil, 41 mg, 92%, 95 % ee , PE: EtOAc = 10: 1).

(1*S*,6*S*)-1-(((tert-butyldimethylsilyl)oxy)methyl)-6-(difluoromethyl)-3-tosyl-3-azabicyclo[4.1.0]heptane (6)

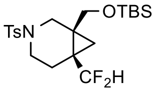

Colorless oil, 41 mg, 95% yield, Rf = 0.2 (PE: EtOAc = 10:1). ^{1}H NMR (500 MHz, Chloroform-*d*) δ 7.62 (d, *J* = 8.2 Hz, 2H), 7.32 (d, *J* = 8.0 Hz, 2H), 5.60 (t, *J* = 56.1 Hz, 1H), 3.68 (t, *J* = 10.8 Hz, 2H), 3.52 (dd, *J* = 18.1, 8.8 Hz, 2H), 2.57 (d, *J* = 11.4 Hz, 1H), 2.43 (s, 3H), 2.22 (dq, *J* = 11.4, 4.8 Hz, 2H), 2.10 – 1.75 (m, 1H), 1.05 (d, *J* = 5.6 Hz, 1H), 1.00 – 0.93 (m, 1H), 0.83 (s, 9H), 0.00 (d, *J* = 4.7 Hz, 6H). **^{13}C NMR** (126 MHz, $CDCl_3$) δ 143.74, 133.03, 129.73, 127.63, 118.56 (t, *J* = 238.6 Hz), 63.42, 46.41, 43.58, 26.94 (d, *J* = 6.9 Hz), 25.70, 24.34 (t, *J* = 26.4 Hz), 21.89, 21.53, 16.92 (d, *J* = 7.4 Hz), -5.60, -5.65. **^{19}F NMR** (471 MHz, Chloroform-*d*) δ -113.22 (d, *J* = 283.9 Hz), -120.33 (d, *J* = 283.9 Hz). **HRMS**: Calculation for $C_{21}H_{33}F_2NO_3SSi$, $[M+Na]^+$, 468.1816 Found:

468.1812. HPLC: INA column, 98:2 hexane: isopropanol, 0.80 mL/min, t_R = major: 21.4 min, minor: 22.9 min. 95% ee. $[\alpha]_D^{29}$ = 13° (c 0.3, CH_2Cl_2).

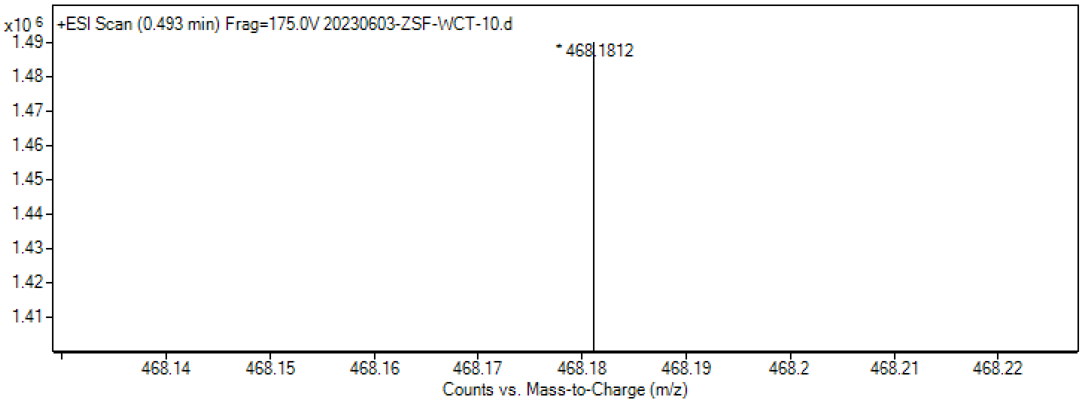

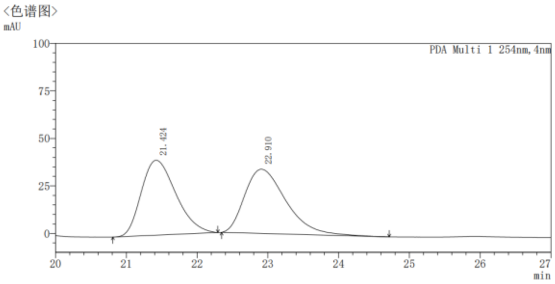

<峰表>

PDA Ch1 254nm

峰号	保留时间	面积	高度	高度%	面积%
1	21.424	1343452	39451	53.871	49.502
2	22.910	1370476	33780	46.129	50.498
总计		2713928	73231	100.000	100.000

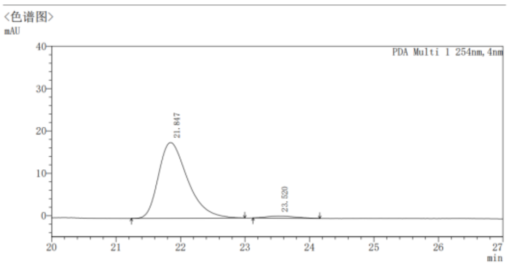

<峰表>

PDA Ch1 254nm

峰号	保留时间	面积	高度	高度%	面积%
1	21.847	562035	17895	97.395	97.412
2	23.520	14933	479	2.605	2.588
总计		576967	18374	100.000	100.000

Competitive KIE experiments

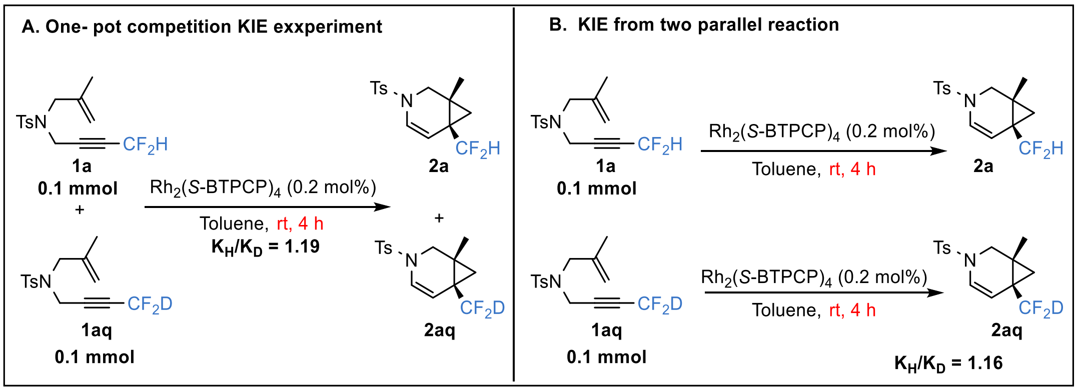

One- pot competition KIE experiment: under N_2, to an over-dried 25 mL Schlenk tube equipped with a magnetic stir bar was added **1a** (0.1 mmol) ,**1aq** (0.1 mmol) and Rh_2(*S*-BTPCP) (0.72 mg), toluene (2 mL). The reaction was stirred at room temperature for 4 hours, The reaction mixture was concentrated under reduced pressure. After that, the yields of product were determined by NMR.

KIE from two parallel reaction:

To a toluene solution of **1a** or **1aq** (0.1 mmol, 0.4 ml) in Schlenk tube with a magnetic bar was added 0.2

mol% (0.36 mg) Rh_2(*S*-BTPCP)$_4$ under N_2. The sealed tube was then stirred at room temperature under nitrogen atmosphere for 4 h. he reaction mixture was concentrated under reduced pressure. After that, the yield of product **2a** or **1aq** was determined by NMR.

5. The NOE of compound 4

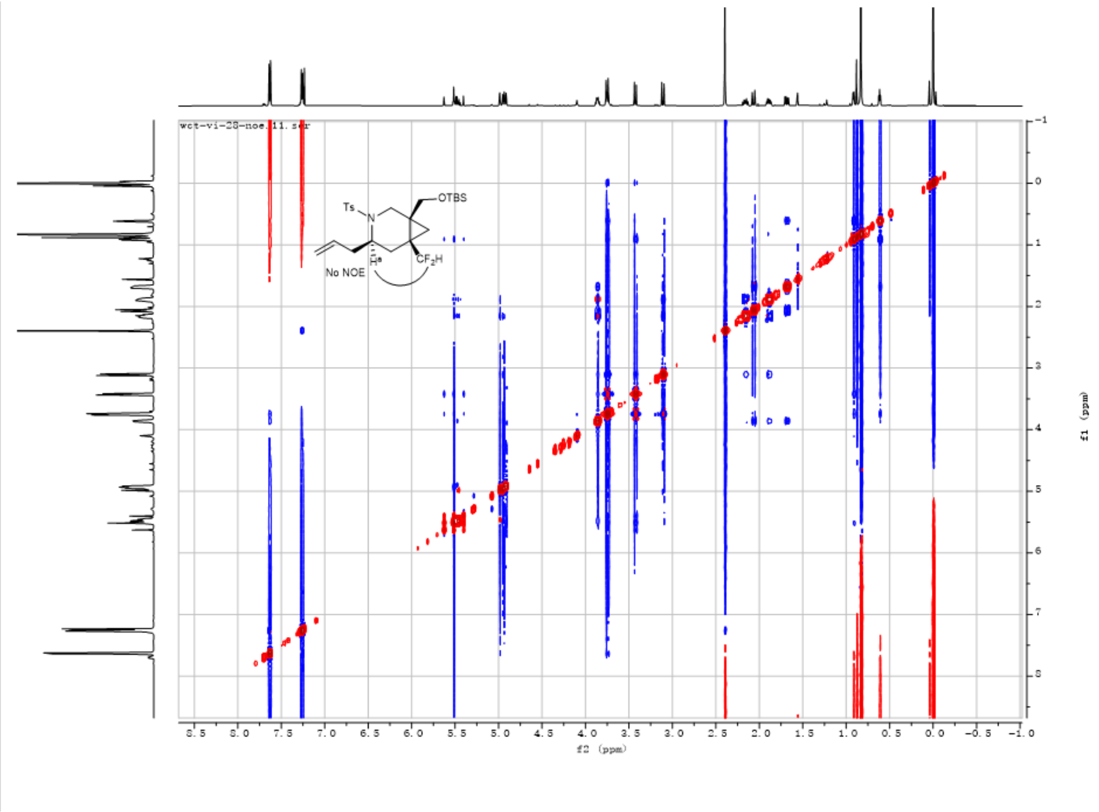

6. The X-ray diffraction analysis

Crystal data and structure refinement for **2e** (CCDC 2271390)

Single crystal of **2e** was grown from slow evaporation of DCM/PE solvent. A suitable crystal was selected and measured on an Agilent SuperNova, Dual, Cu at zero, AtlasS2 diffractometer. The crystal was kept at 293(2) K during data collection.

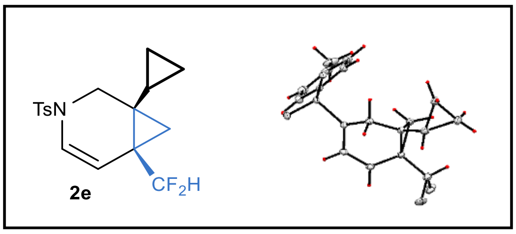

Table 1 Crystal data and structure refinement for 2e.

Identification code	2e
Empirical formula	$C_{17}H_{19}F_2NO_2S$
Formula weight	339.39
Temperature/K	170.00(10)
Crystal system	monoclinic
Space group	P2
a/Å	14.9687(2)
b/Å	11.94911(17)
c/Å	19.7926(3)
α/°	90
β/°	109.8820(19)
γ/°	90
Volume/Å^3	3329.15(10)
Z	8
ρ_{calc}g/cm^3	1.354
μ/mm^{-1}	1.992
F(000)	1424.0
Crystal size/mm^3	0.15 × 0.13 × 0.1
Radiation	Cu Kα (λ = 1.54184)
2Θ range for data collection/	4.748 to 147.622
Index ranges	$-18 \leq h \leq 12$, $-14 \leq k \leq 14$, $-24 \leq l \leq 24$
Reflections collected	27106
Independent reflections	13049 [R_{int} = 0.0401, R_{sigma} = 0.0461]
Data/restraints/parameters	13049/1/833

Goodness-of-fit on F^2	1.018
Final R indexes [I>=2σ (I)]	R_1 = 0.0426, wR_2 = 0.1086
Final R indexes [all data]	R_1 = 0.0452, wR_2 = 0.1119
Largest diff. peak/hole / e Å^{-3}	0.16/-0.41
Flack/Hooft parameter	0.063(11)/0.081(8)

Crystal data and structure refinement for **3f** (CCDC 2304712)

Single crystal of **3f** was grown from slow evaporation of DCM/PE solvent. A suitable crystal was selected and measured on an Agilent SuperNova, Dual, Cu at zero, AtlasS2 diffractometer. The crystal was kept at 293(2) K during data collection.

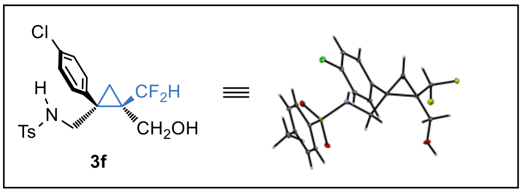

Table 1 Crystal data and structure refinement for 3f.

Identification code	3f
Empirical formula	$C_{19}H_{20}ClF_2NO_3S$
Formula weight	415.87
Temperature/K	149.99(10)
Crystal system	monoclinic
Space group	C2
a/Å	23.0015(11)
b/Å	6.2636(3)
c/Å	13.1008(6)
α/°	90
β/°	92.850(5)
γ/°	90
Volume/Å^3	1885.14(16)
Z	4
ρ_{calc}g/cm^3	1.465
μ/mm^{-1}	3.185
F(000)	864.0
Crystal size/mm^3	0.14 × 0.13 × 0.1
Radiation	Cu Kα (λ = 1.54184)
2Θ range for data collection/	6.756 to 147.814
Index ranges	$-26 \le h \le 28$, $-6 \le k \le 7$, $-16 \le l \le 15$
Reflections collected	7665
Independent reflections	3377 [R_{int} = 0.0511, R_{sigma} = 0.0557]
Data/restraints/parameters	3377/1/250
Goodness-of-fit on F^2	1.063
Final R indexes [I>=2σ (I)]	R_1 = 0.0619, wR_2 = 0.1550
Final R indexes [all data]	R_1 = 0.0632, wR_2 = 0.1581
Largest diff. peak/hole / e Å^{-3}	0.81/-0.42
Flack/Hooft parameter	0.018(18)/0.015(13)

Crystal data and structure refinement for **5** (CCDC 2305253)

Single crystal of **5** was grown from slow evaporation of DCM/PE solvent at 0 °C. A suitable crystal was selected and measured on an Agilent SuperNova, Dual, Cu at zero, AtlasS2 diffractometer. The crystal was kept at 293(2) K during data collection.

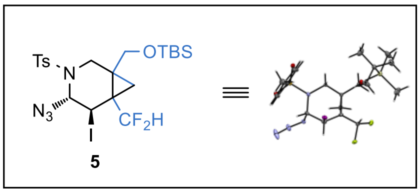

Table 1 Crystal data and structure refinement for 5.

Identification code	5
Empirical formula	$C_{21}H_{31}F_2IN_4O_3SSi$
Formula weight	612.55
Temperature/K	293.00(10)
Crystal system	orthorhombic
Space group	$P2_12_12_1$
a/Å	7.5604(5)
b/Å	14.2693(10)
c/Å	25.599(2)
α/°	90
β/°	90
γ/°	90
Volume/Å^3	2761.7(3)
Z	4
ρ_{calc}g/cm^3	1.473
μ/mm^{-1}	10.595
F(000)	1240.0
Crystal size/mm^3	0.14 × 0.12 × 0.1
Radiation	Cu Kα (λ = 1.54184)
2Θ range for data collection/°	6.906 to 147.136
Index ranges	$-5 \leq h \leq 9$, $-17 \leq k \leq 17$, $-23 \leq l \leq 31$
Reflections collected	10141
Independent reflections	5377 [R_{int} = 0.0707, R_{sigma} = 0.0896]
Data/restraints/parameters	5377/303/312
Goodness-of-fit on F^2	1.045
Final R indexes [I>=2σ (I)]	R_1 = 0.0786, wR_2 = 0.1920
Final R indexes [all data]	R_1 = 0.1040, wR_2 = 0.2342
Largest diff. peak/hole / e Å^{-3}	1.21/-1.25
Flack/Hooft parameter	-0.013(11)/0.026(8)

Crystal data and structure refinement for **complex B** (CCDC 2281599)

Single crystal of **complex B** was grown from slow evaporation of DCM/PE solvent. A suitable crystal was selected and measured on an Agilent SuperNova, Dual, Cu at zero, AtlasS2 diffractometer. The crystal was kept at 293(2) K during data collection.

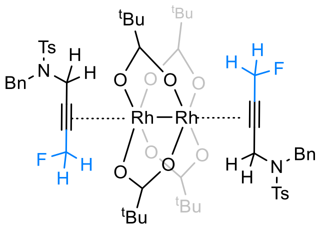

complex B

|||

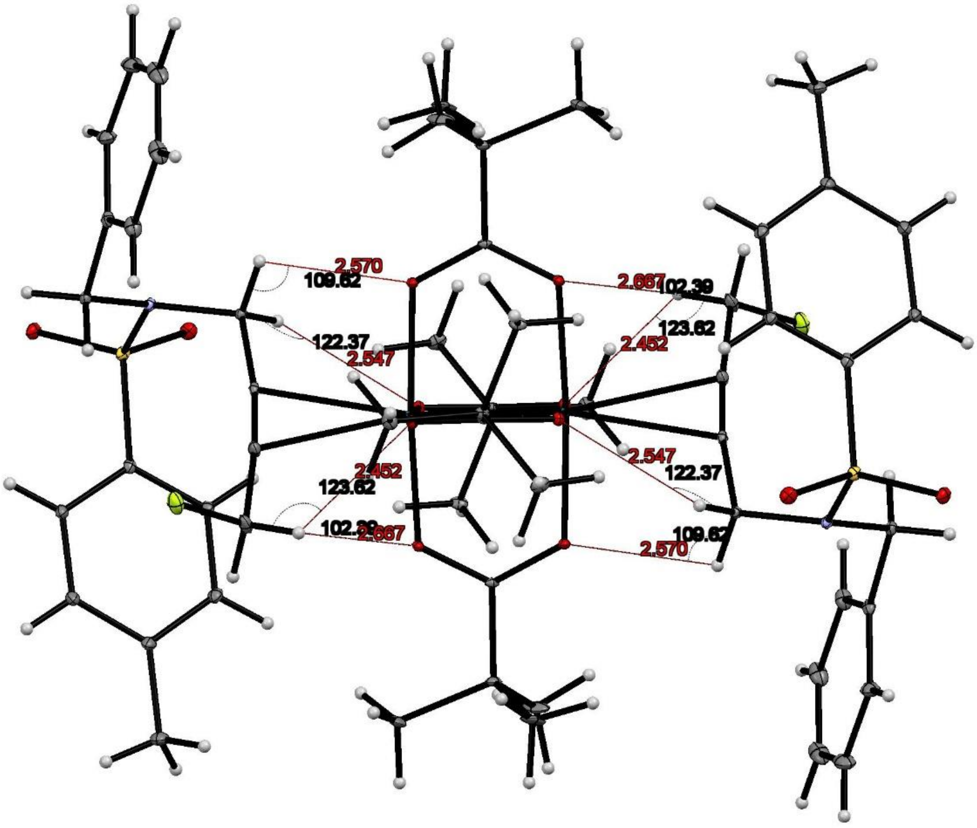

Table 2 Crystal data and structure refinement for complex B.

Identification code	**complex B**
Empirical formula	$C_{56}H_{72}F_2N_2O_{12}Rh_2S_2$
Formula weight	1273.09
Temperature/K	200.00(10)
Crystal system	triclinic
Space group	P-1
a/Å	10.9854(6)
b/Å	11.9706(7)
c/Å	12.8092(8)
α/°	75.767(5)

β/°	83.728(5)
γ/°	70.207(5)
Volume/Å^3	1535.65(17)
Z	1
ρ_{calc}g/cm^3	1.377
μ/mm^{-1}	0.668
F(000)	658.0
Crystal size/mm^3	0.14 × 0.11 × 0.09
Radiation	Mo Kα (λ = 0.71073)
2Θ range for data collection/°	4.37 to 49.996
Index ranges	-12 ≤ h ≤ 13, -13 ≤ k ≤ 14, -15 ≤ l ≤ 14
Reflections collected	10400
Independent reflections	5399 [R_{int} = 0.0337, R_{sigma} = 0.0533]
Data/restraints/parameters	5399/78/390
Goodness-of-fit on F^2	1.033
Final R indexes [I>=2σ (I)]	R_1 = 0.0429, wR_2 = 0.1048
Final R indexes [all data]	R_1 = 0.0512, wR_2 = 0.1112
Largest diff. peak/hole / e Å^{-3}	0.88/-1.39

Crystal data and structure refinement for **complex C** (CCDC 2281598)

Single crystal of **complex C** was grown from slow evaporation of DCM/PE solvent. A suitable crystal was selected and measured on an Agilent SuperNova, Dual, Cu at zero, AtlasS2 diffractometer. The crystal was kept at 293(2) K during data collection.

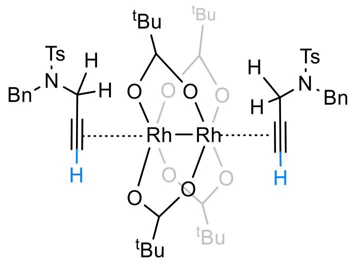

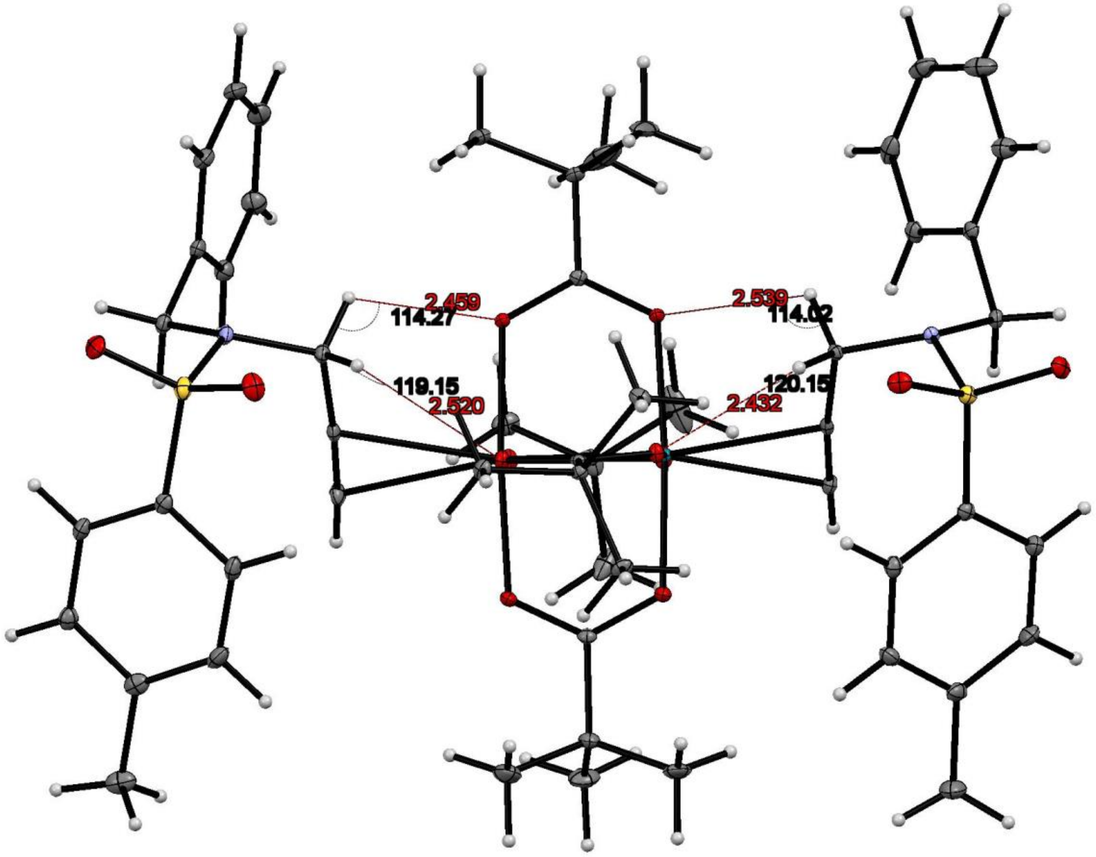

Table 3 Crystal data and structure refinement for complex C.

Identification code	**complex C**
Empirical formula	$C_{54}H_{70}N_2O_{12}Rh_2S_2$
Formula weight	1209.06
Temperature/K	150.00(10)
Crystal system	triclinic
Space group	P-1
a/Å	11.8923(4)
b/Å	12.9555(5)
c/Å	19.3309(7)
α/°	83.007(3)
β/°	87.278(3)
γ/°	85.019(3)
Volume/Å^3	2942.95(19)
Z	2

ρ_{calc}g/cm^3	1.364
μ/mm^{-1}	0.689
F(000)	1252.0
Crystal size/mm^3	0.12 × 0.11 × 0.09
Radiation	Mo Kα (λ = 0.71073)
2Θ range for data collection/°	4.026 to 49.998
Index ranges	-14 ≤ h ≤ 14, -15 ≤ k ≤ 14, -20 ≤ l ≤ 22
Reflections collected	23989
Independent reflections	10358 [R_{int} = 0.0318, R_{sigma} = 0.0514]
Data/restraints/parameters	10358/0/671
Goodness-of-fit on F^2	1.038
Final R indexes [I>=2σ (I)]	R_1 = 0.0350, wR_2 = 0.0683
Final R indexes [all data]	R_1 = 0.0476, wR_2 = 0.0747
Largest diff. peak/hole / e Å^{-3}	0.47/-0.58

7. The NMR spectra of compounds 1aj-al and complexes A-C.

complex A:

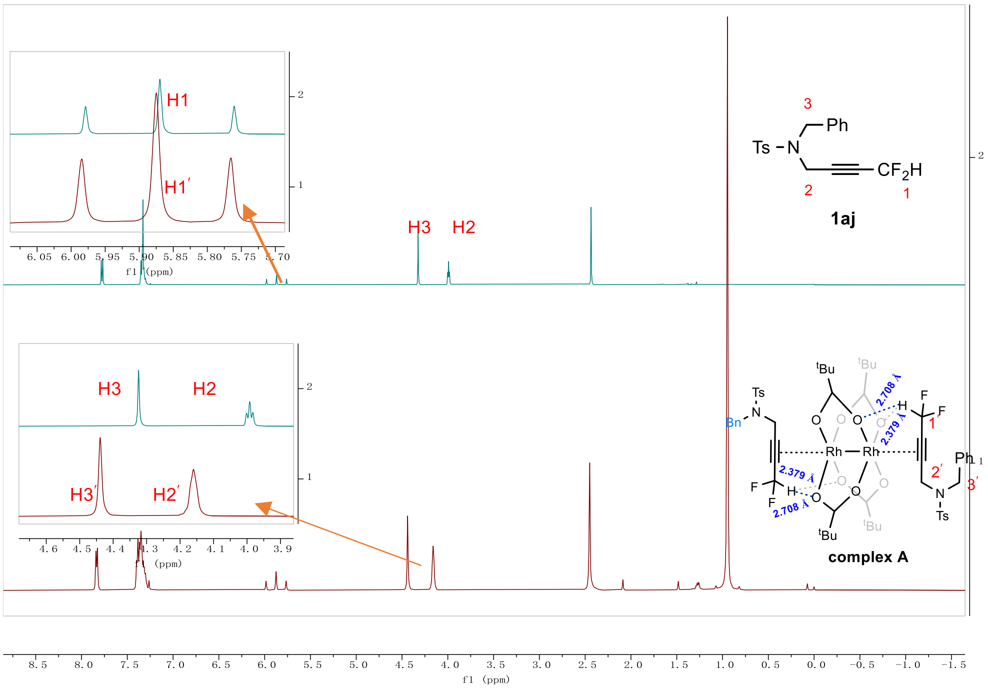

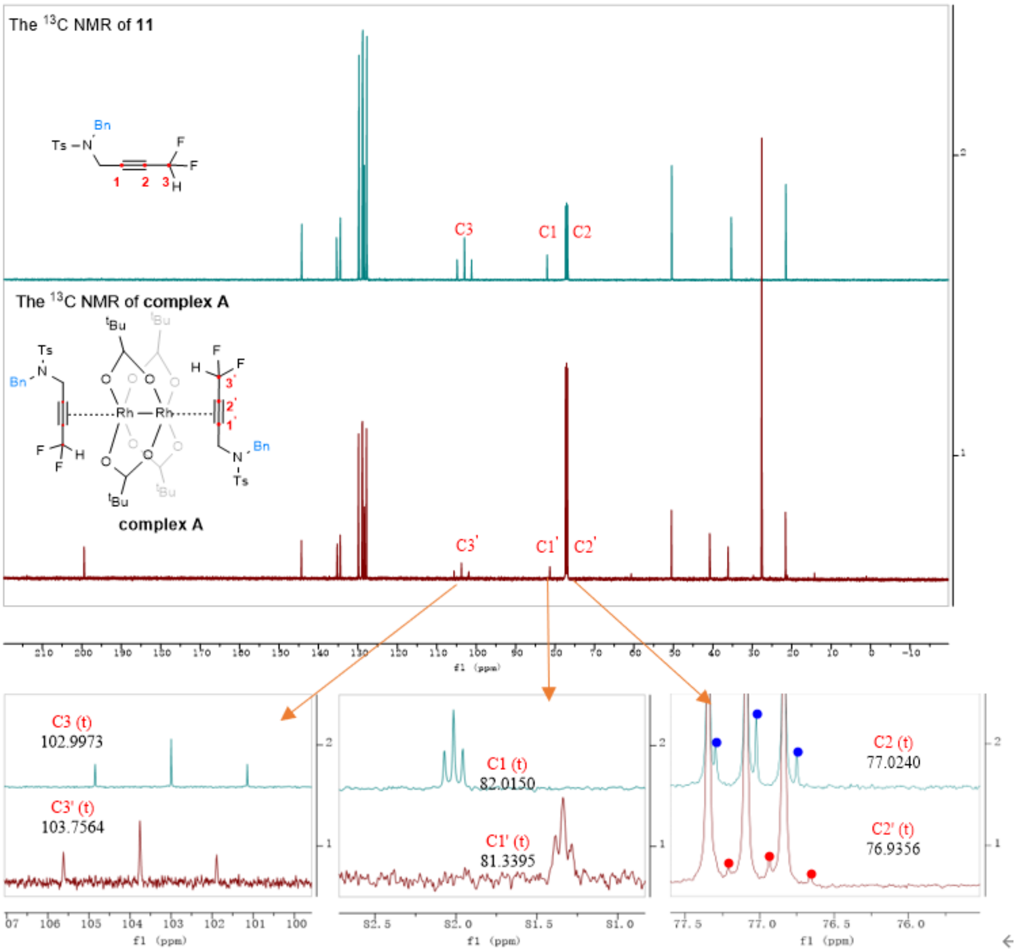
The ^{13}C NMR of 11
The ^{13}C NMR of complex A
complex A
C3 (t)
102.9973
C3' (t)
103.7564
C1 (t)
82.0150
C1' (t)
81.3395
C2 (t)
77.0240
C2' (t)
76.9356
f1 (ppm)

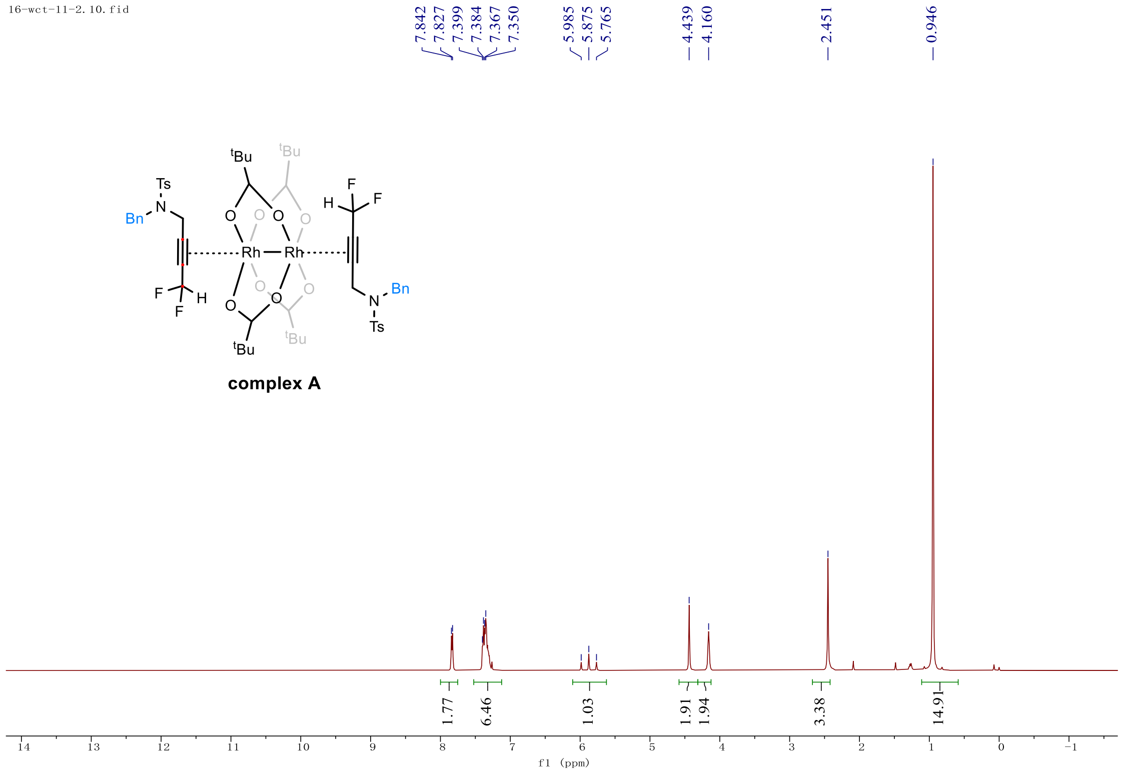
16-wct-11-2.10.fid
7.842
7.827
7.399
7.384
7.367
7.350
5.985
5.875
5.765
4.439
4.160
2.451
0.946
complex A
1.77
6.46
1.03
1.91
1.94
3.38
14.91
f1 (ppm)

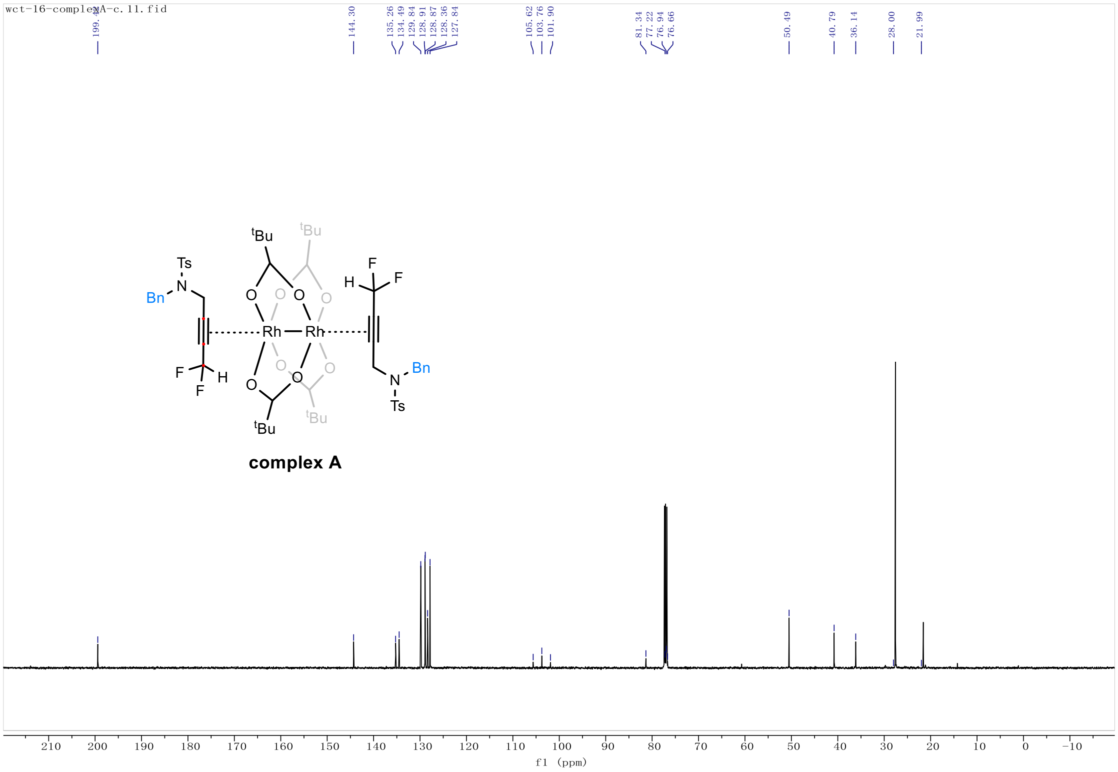
wct-16-complexA-c.11.fid
199.44
144.30
135.26
134.49
129.84
128.91
128.87
128.36
127.84
105.62
103.76
101.90
81.34
77.22
76.94
76.66
50.49
40.79
36.14
28.00
21.99
complex A
f1 (ppm)

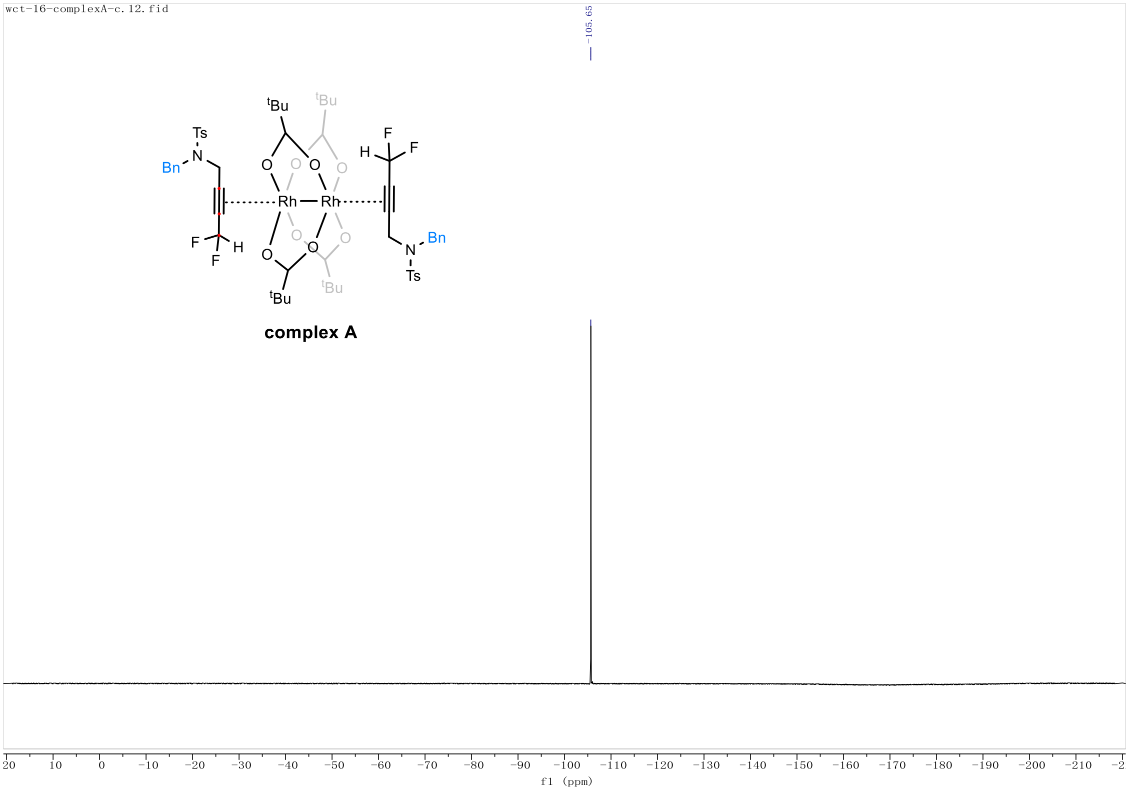
wct-16-complexA-c.12.fid
-105.65
complex A
f1 (ppm)

Complex A:

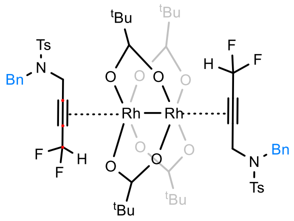

green solid. [1]**H NMR** (500 MHz, Chloroform-*d*) δ 7.83 (d, *J* = 7.9 Hz, 2H), 7.37 (dd, *J* = 16.4, 7.8 Hz, 7H), 5.88 (t, *J* = 54.8 Hz, 1H), 4.44 (s, 2H), 4.16 (s, 2H), 2.45 (s, 3H), 0.95 (s, 18H). [13]**C NMR** (126 MHz, $CDCl_3$) δ 199.42, 144.30, 135.26, 134.49, 129.84, 128.91, 128.87, 128.36, 127.84, 103.76 (t, *J* = 232.9 Hz), 81.34(t, *J* = 5.5 Hz), 76.94(t, *J* = 34.3 Hz),50.49, 40.79, 36.14, 28.00, 21.99.[19]**F NMR** (471 MHz, CDCl3) δ -105.65.

complex B:

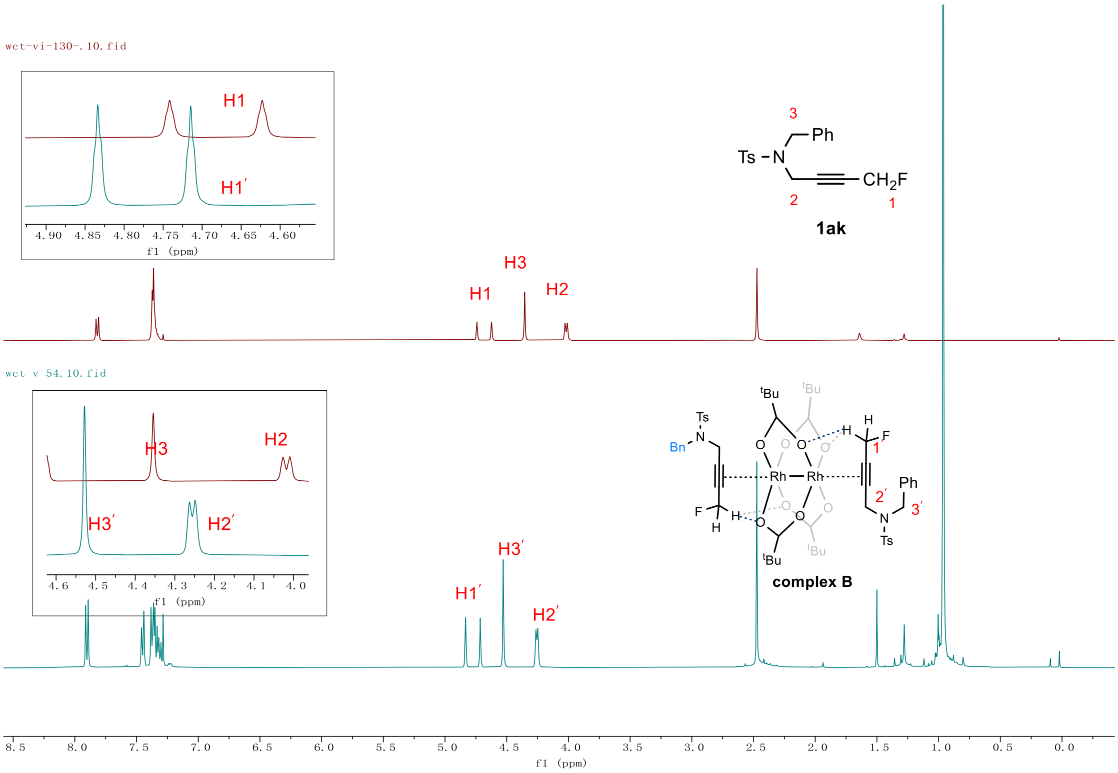

wct-vi-130-.10.fid
wct-v-54.10.fid
H1
H1′
H2
H3
H1′
H2′
H3′
1ak
complex B
f1 (ppm)

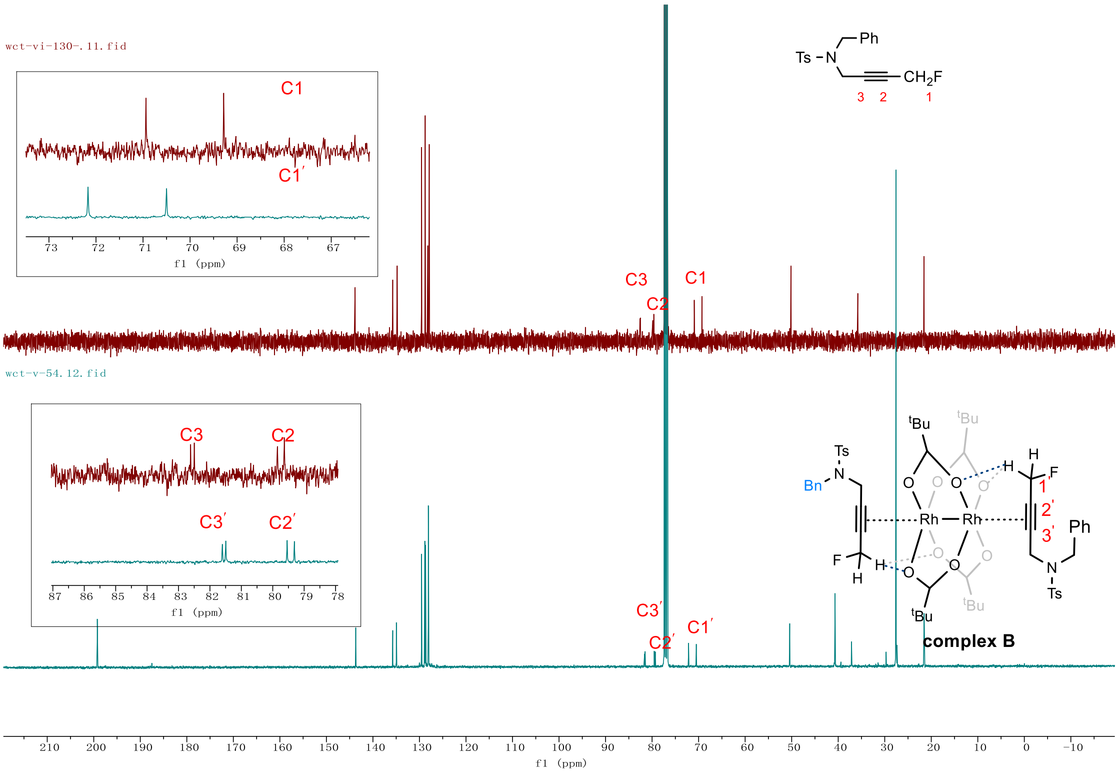

wct-vi-130-.11.fid
wct-v-54.12.fid
C1
C1′
C2
C3
C2′
C3′
complex B
f1 (ppm)

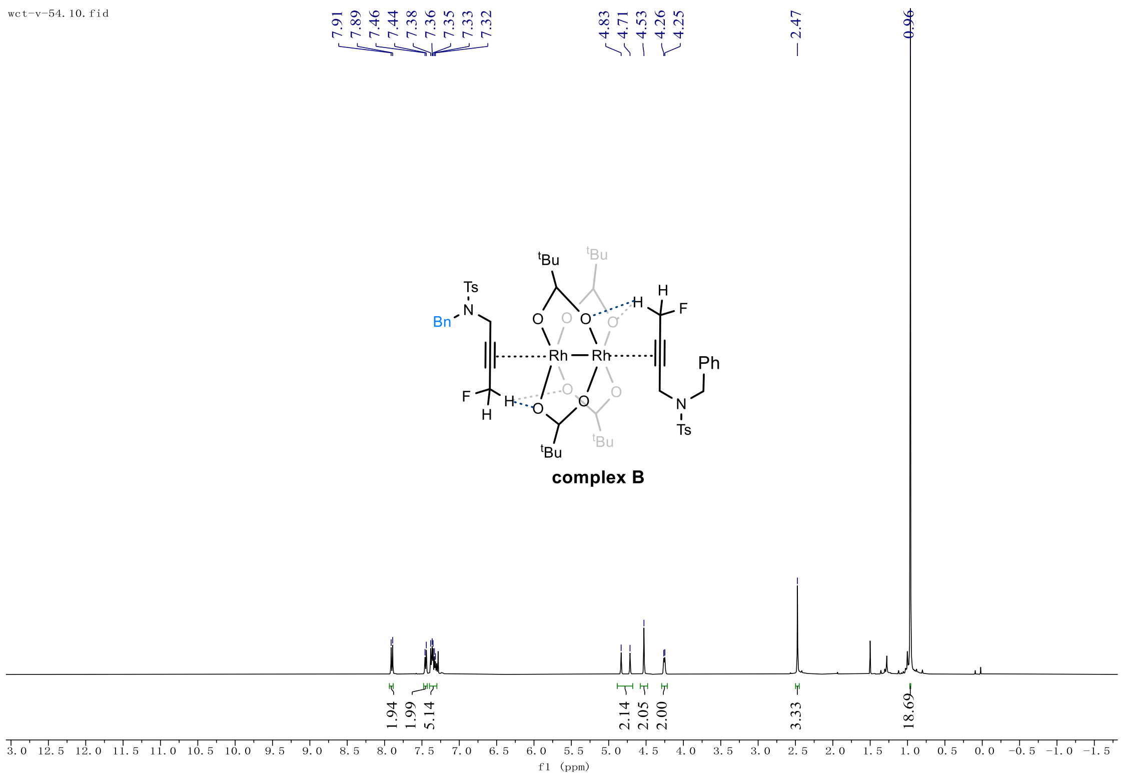
wct-v-54.10.fid
7.91
7.89
7.46
7.44
7.38
7.36
7.35
7.33
7.32
4.83
4.71
4.53
4.26
4.25
2.47
0.96
Ts
N
Bn
F
H
tBu
O
Rh
Ph
complex B
1.94
1.99
5.14
2.14
2.05
2.00
3.33
18.69
f1 (ppm)

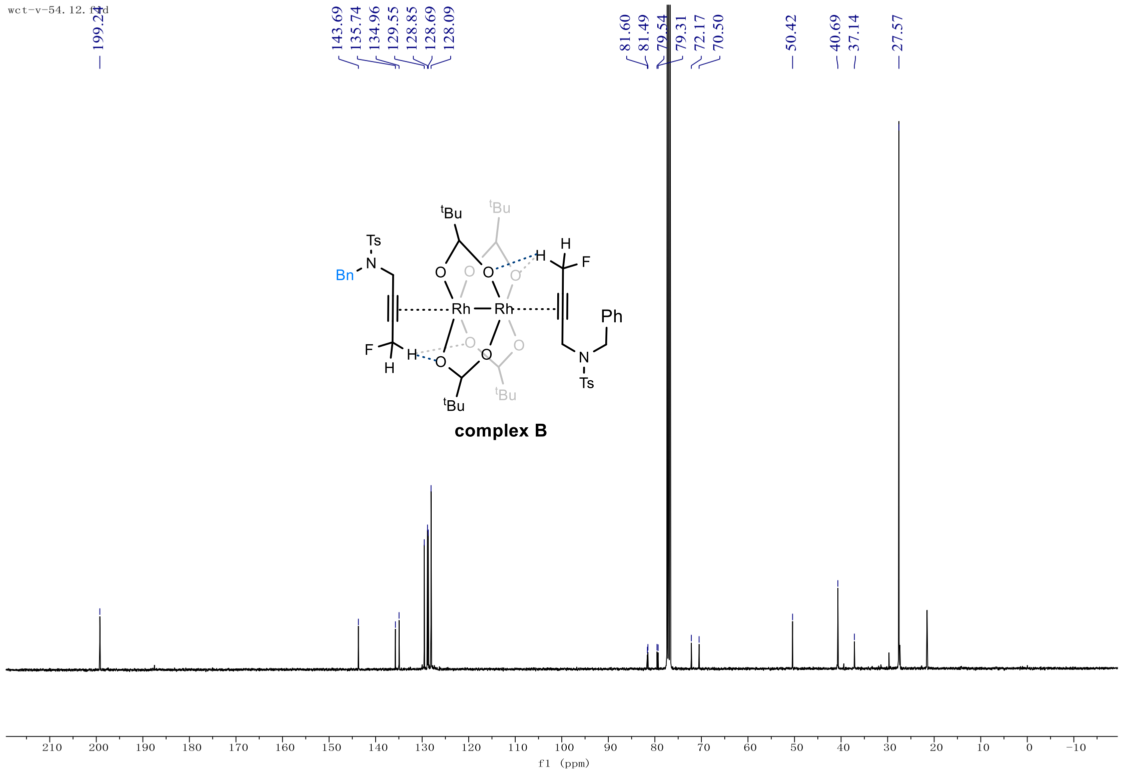
wct-v-54.12.fid
199.24
143.69
135.74
134.96
129.55
128.85
128.69
128.09
81.60
81.49
79.54
79.31
72.17
70.50
50.42
40.69
37.14
27.57
complex B
f1 (ppm)

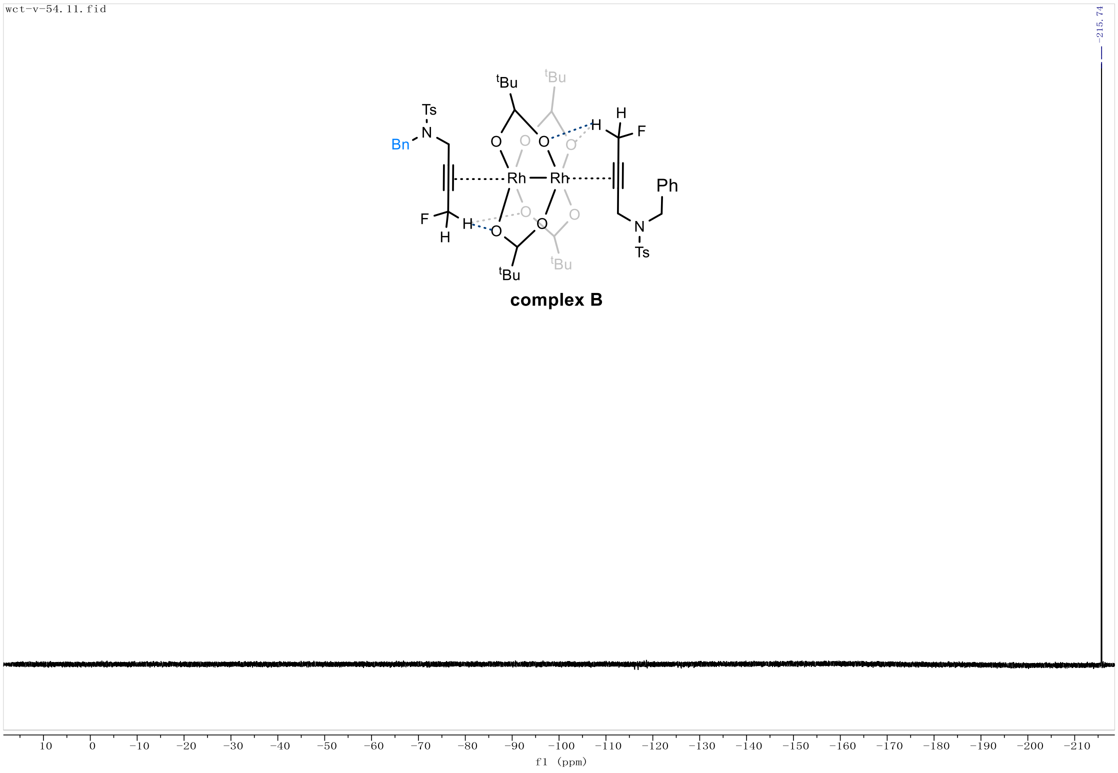

Complex B:

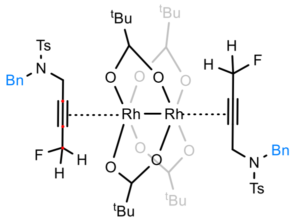

green solid. **^{1}H NMR** (400 MHz, Chloroform-*d*) δ 7.90 (d, *J* = 8.2 Hz, 2H), 7.45 (d, *J* = 7.1 Hz, 2H), 7.42 – 7.31 (m, 5H), 4.77 (d, *J* = 47.7 Hz, 2H), 4.53 (s, 2H), 4.26 (d, *J* = 5.9 Hz, 2H), 2.47 (s, 3H), 0.96 (s, 18H). **^{13}C NMR** (101 MHz, $CDCl_3$) δ 199.24, 143.69, 135.74, 134.96, 129.55, 128.85, 128.69, 128.09, 81.55 (d, *J* = 11.0 Hz), 79.43 (d, *J* = 23.0 Hz), 71.34 (d, *J* = 167.8 Hz), 50.42, 40.69, 37.14, 27.57. **^{19}F NMR** (376 MHz, $CDCl_3$) δ -215.74.

complex C:

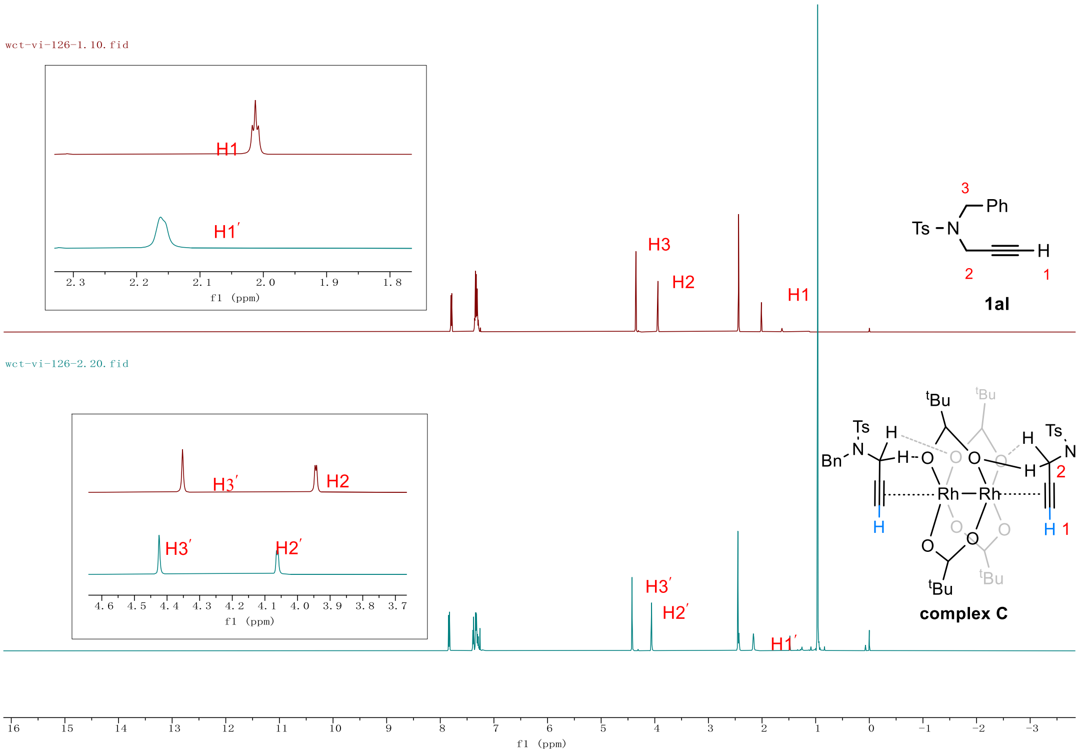

wct-vi-126-1.10.fid
wct-vi-126-2.20.fid
H1
H1′
H3
H2
H3′
H2′
Ts
N
Ph
H
1al
complex C
Rh
tBu
Bn
f1 (ppm)

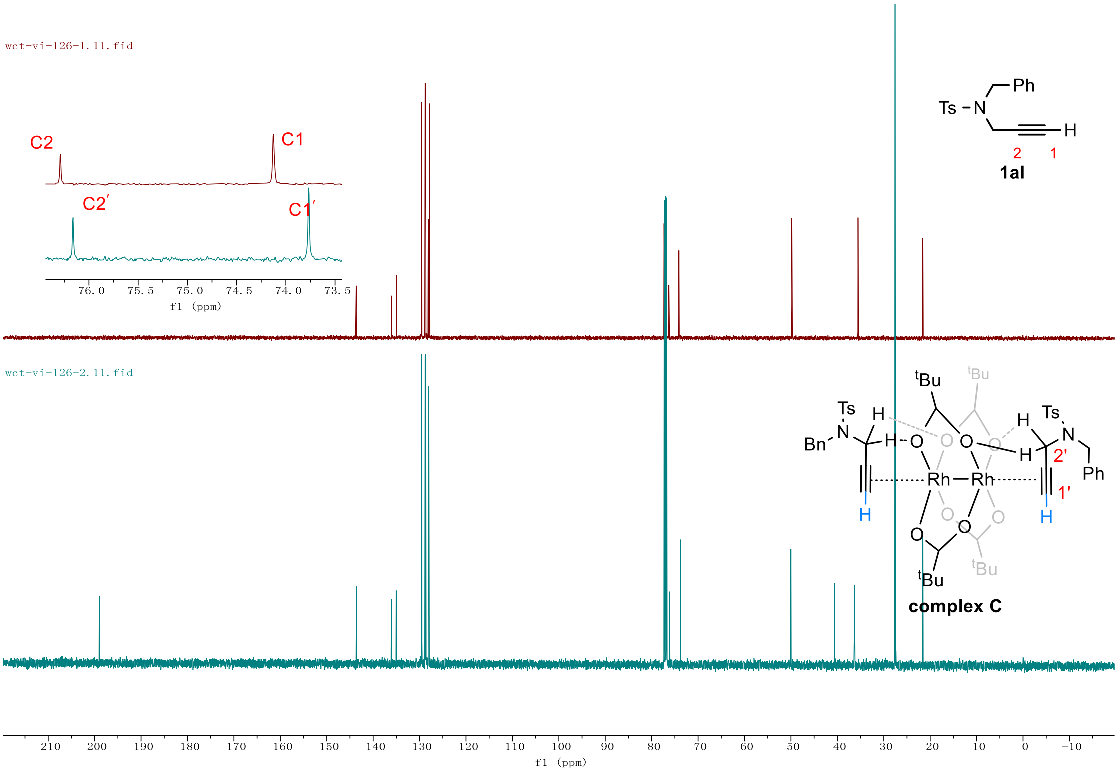

wct-vi-126-1.11.fid
wct-vi-126-2.11.fid
C2
C1
C2′
C1′
Ts
N
Ph
H
1al
complex C
Rh
tBu
Bn
f1 (ppm)

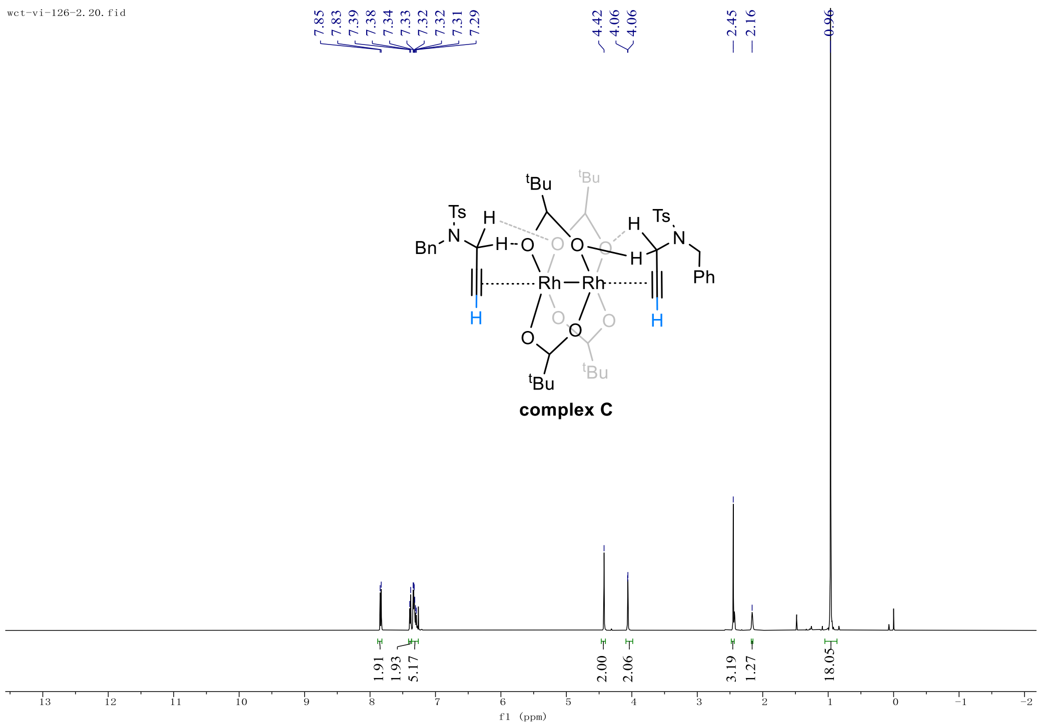

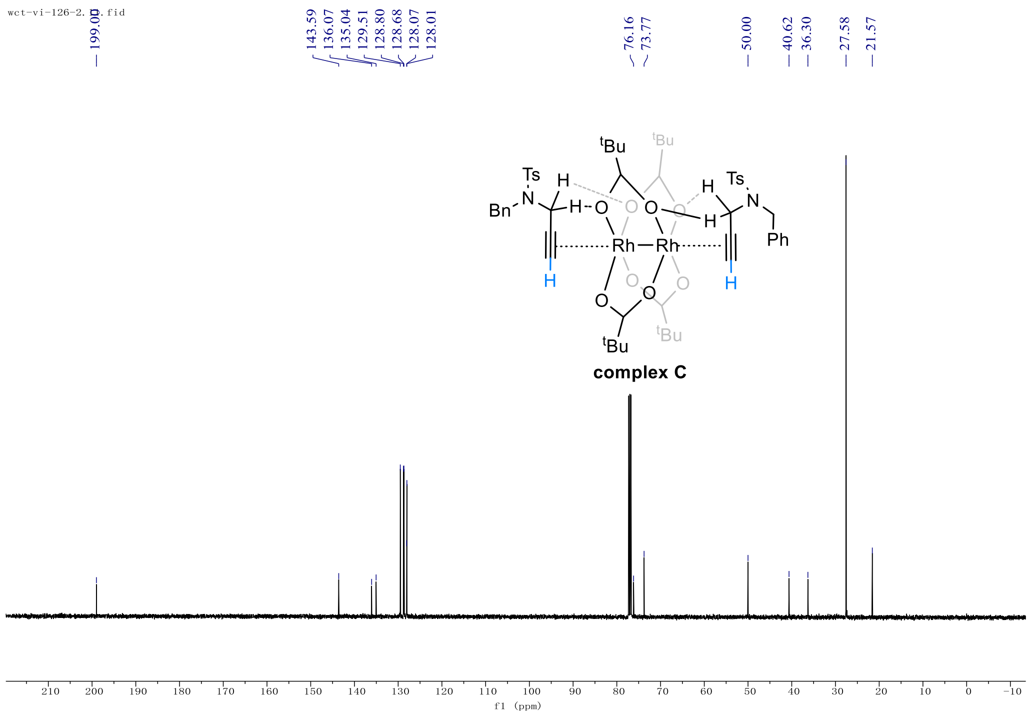

Complex C:

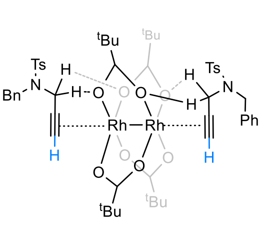
complex C

green solid. **^{1}H NMR** (500 MHz, Chloroform-*d*) δ 7.84 (d, *J* = 8.2 Hz,2H), 7.39 (d, *J* = 7.2 Hz, 2H), 7.36 – 7.28 (m, 5H), 4.42 (s, 2H), 4.06 (d, *J* = 2.3 Hz, 2H), 2.45 (s, 2H), 2.16 (s, 1H), 0.96 (s, 18H). **^{13}C NMR** (126 MHz, $CDCl_3$) δ 199.00, 143.59, 136.07, 135.04, 129.51, 128.80, 128.68, 128.07, 128.01, 76.16, 73.77, 50.00, 40.62, 36.30, 27.58, 21.57.

8. The NMR spectra of compounds 1-6

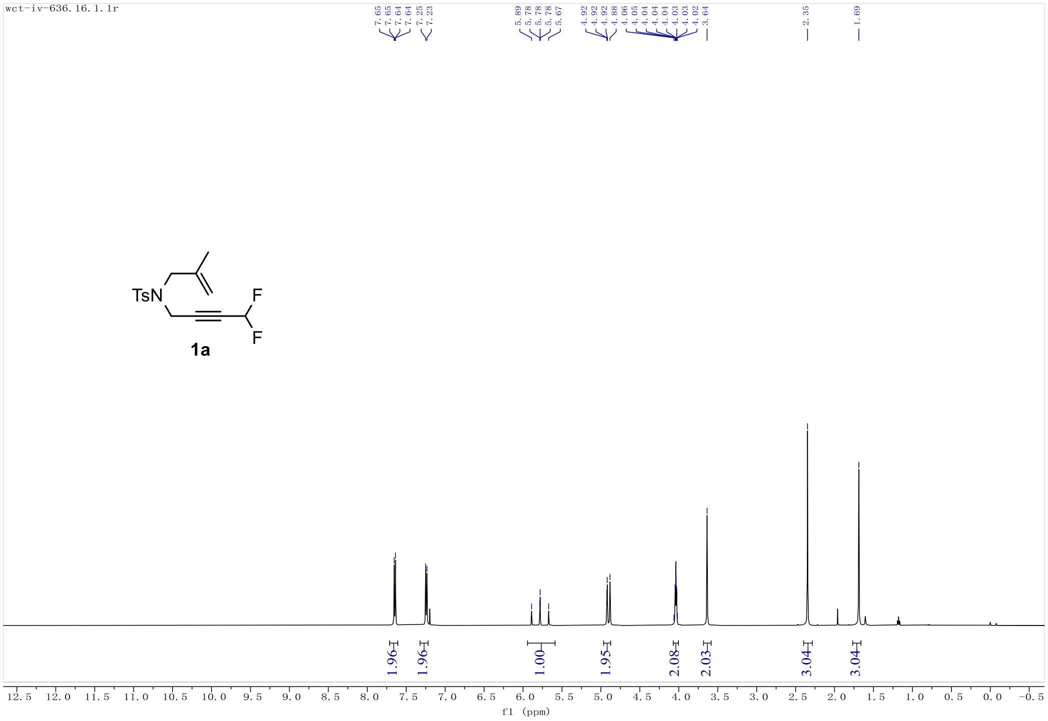

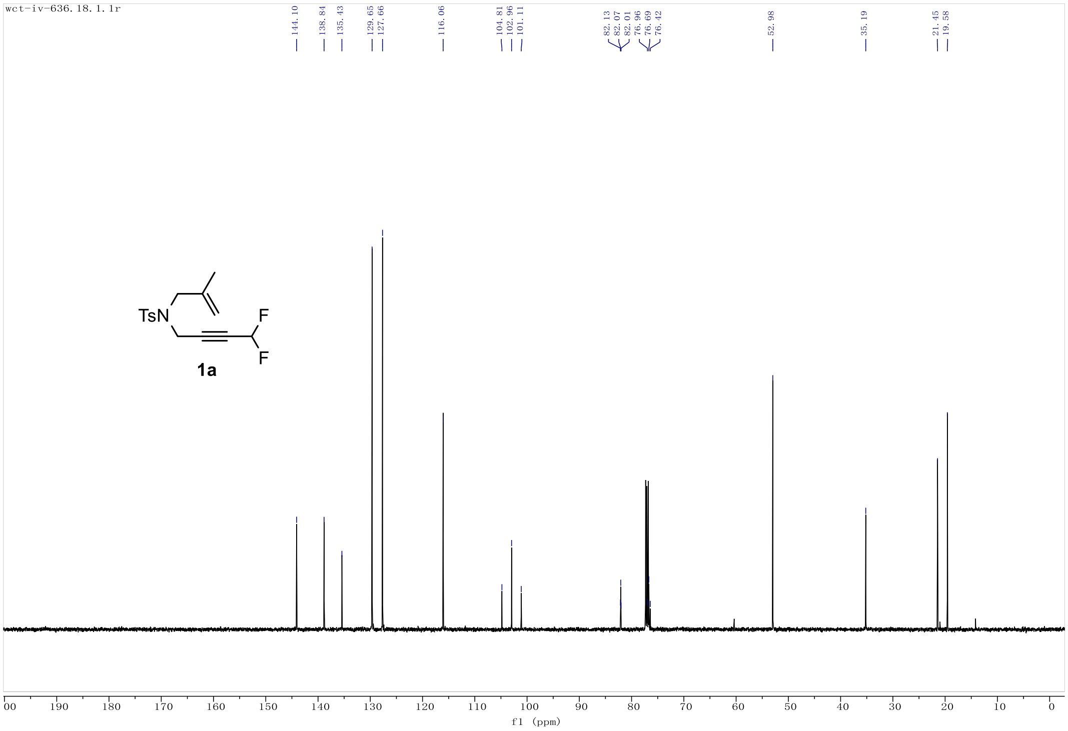

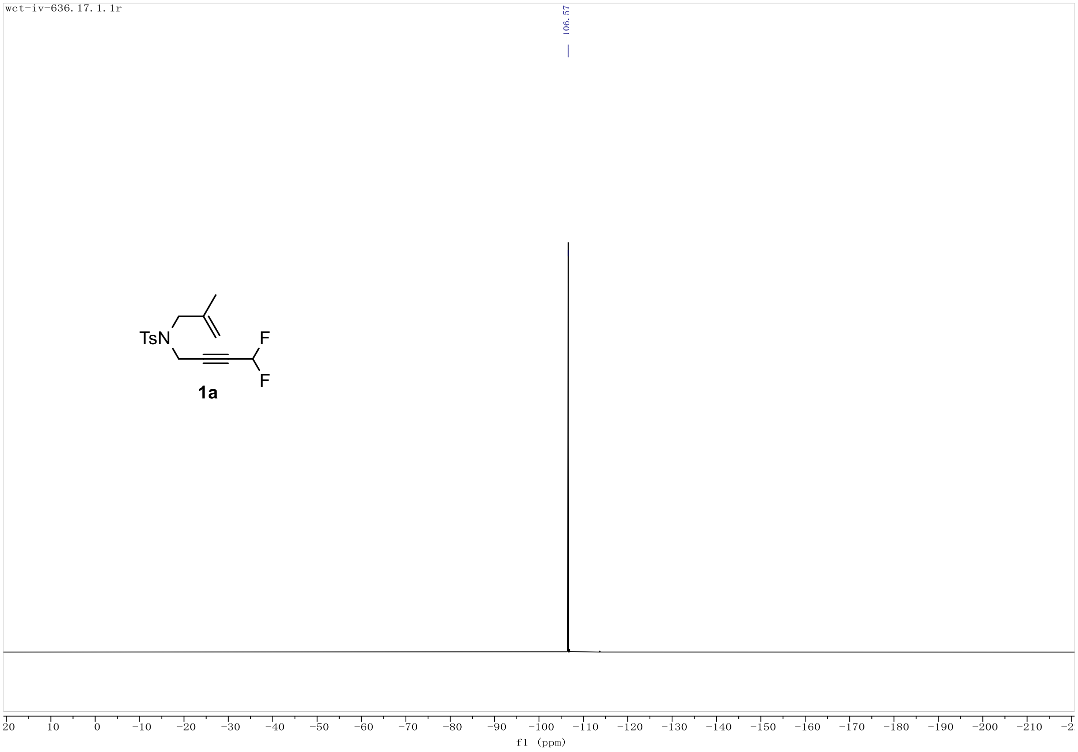
wct-iv-636.17.1.1r
-106.57
TsN
F
F
1a
f1 (ppm)

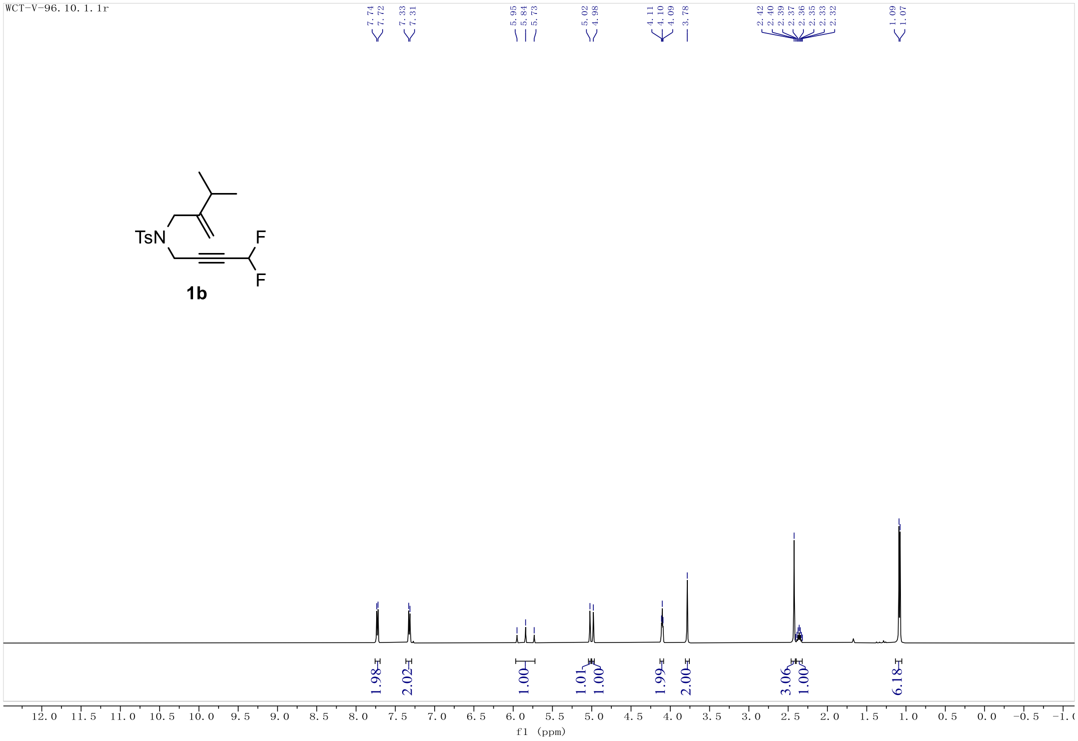
WCT-V-96.10.1.1r
7.74
7.72
7.33
7.31
5.95
5.84
5.73
5.02
4.98
4.11
4.10
4.09
3.78
2.42
2.40
2.39
2.37
2.36
2.35
2.33
2.32
1.09
1.07
TsN
F
F
1b
1.98
2.02
1.00
1.01
1.00
1.99
2.00
3.06
1.00
6.18
f1 (ppm)

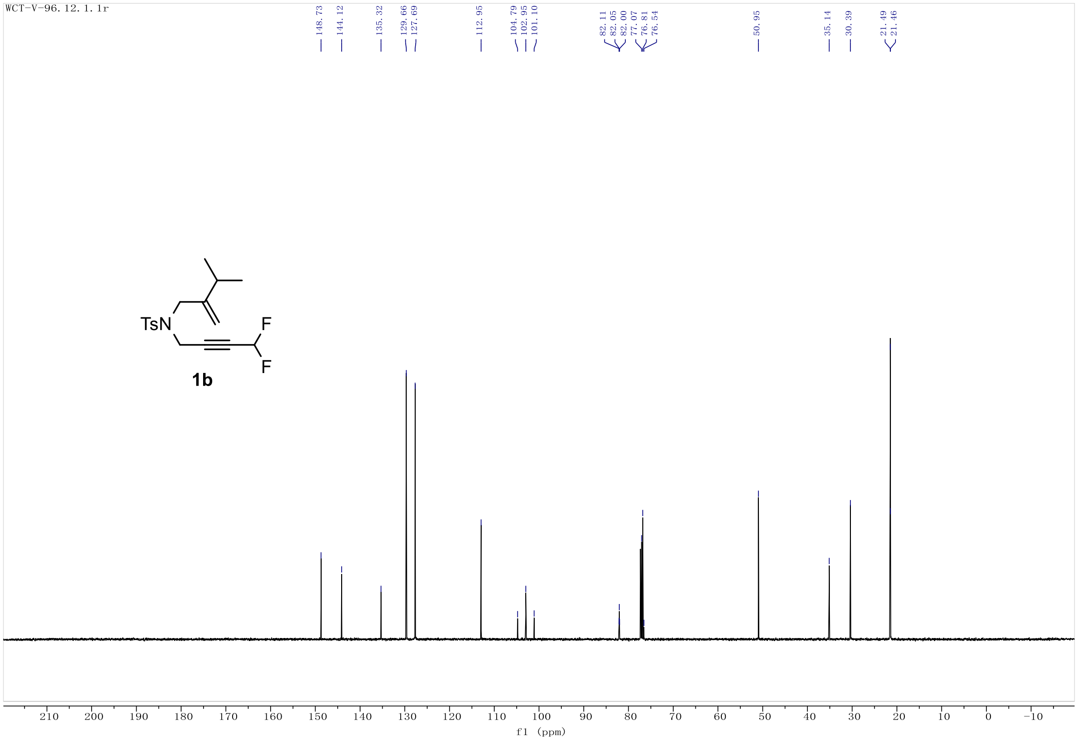
WCT-V-96.12.1.1r
148.73
144.12
135.32
129.66
127.69
112.95
104.79
102.95
101.10
82.11
82.05
82.00
77.07
76.81
76.54
50.95
35.14
30.39
21.49
21.46
TsN
F
F
1b
f1 (ppm)

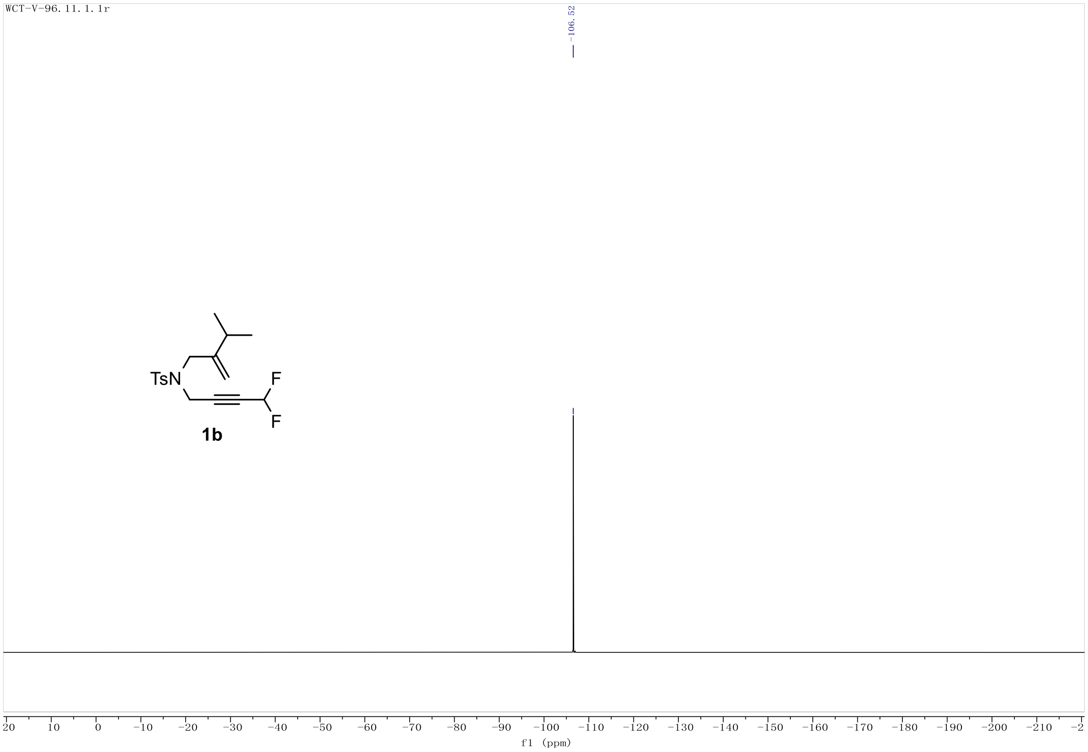
WCT-V-96.11.1.1r
-106.52
TsN
F
F
1b
f1 (ppm)

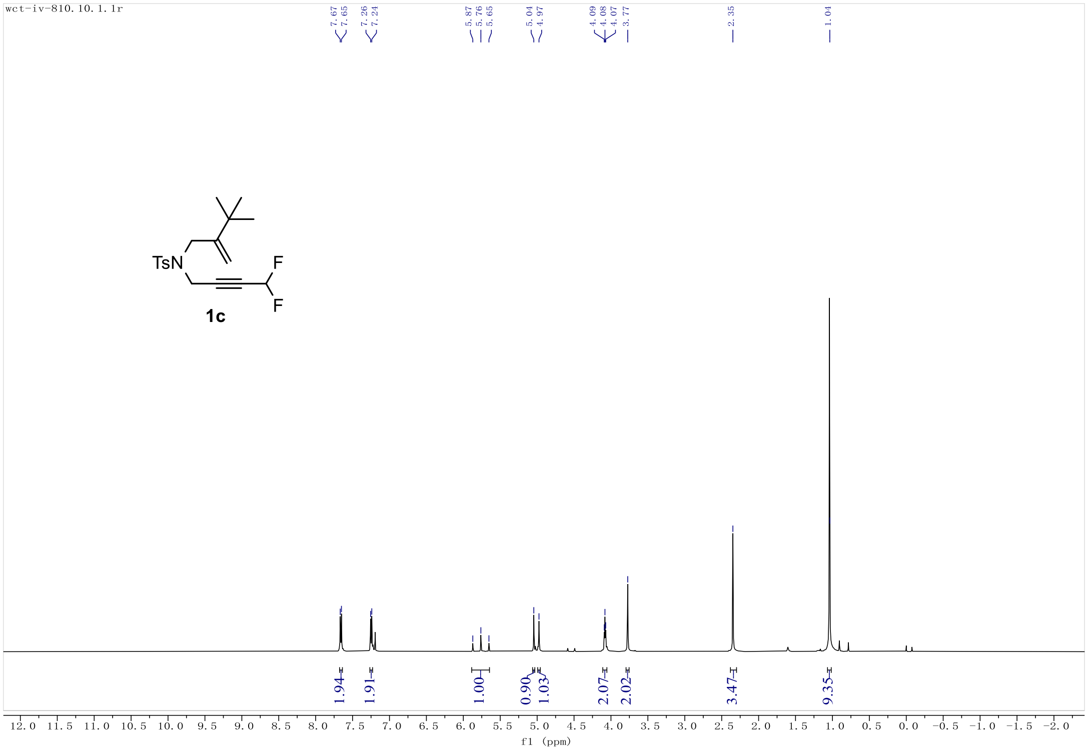
wct-iv-810.10.1.1r
7.67
7.65
7.26
7.24
5.87
5.76
5.65
5.04
4.97
4.09
4.08
4.07
3.77
2.35
1.04
TsN
F
F
1c
1.94
1.91
1.00
0.90
1.03
2.07
2.02
3.47
9.35
12.0 11.5 11.0 10.5 10.0 9.5 9.0 8.5 8.0 7.5 7.0 6.5 6.0 5.5 5.0 4.5 4.0 3.5 3.0 2.5 2.0 1.5 1.0 0.5 0.0 −0.5 −1.0 −1.5 −2.0
f1 (ppm)

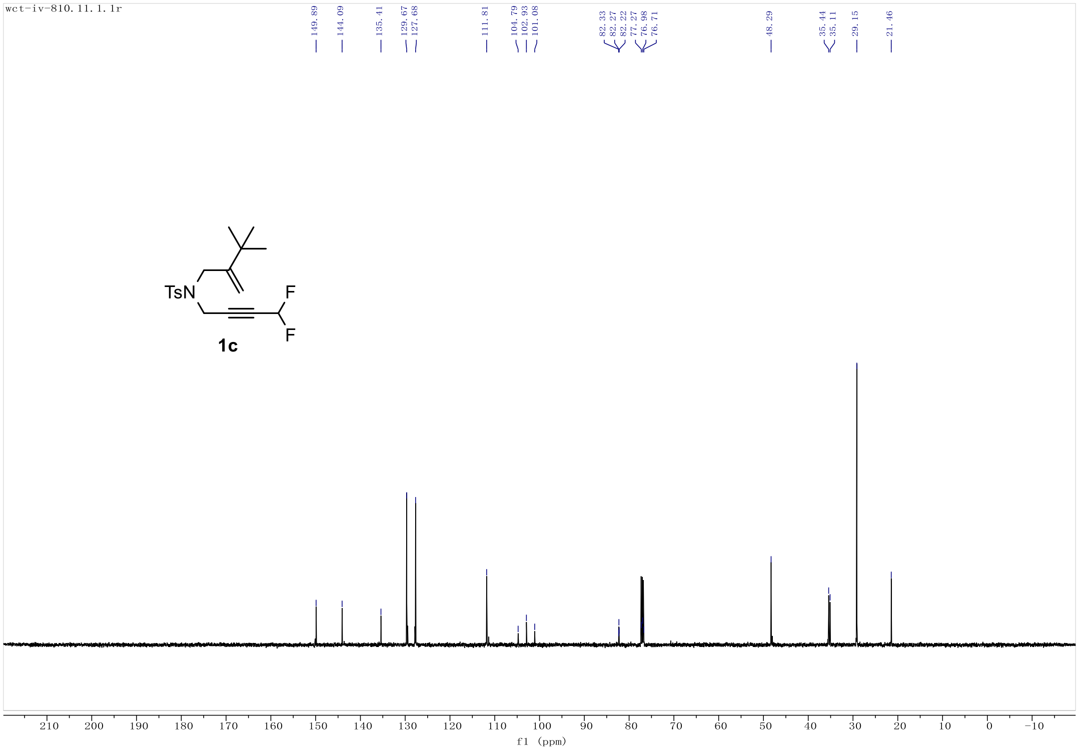
wct-iv-810.11.1.1r
149.89
144.09
135.41
129.67
127.68
111.81
104.79
102.93
101.08
82.33
82.27
82.22
77.27
76.98
76.71
48.29
35.44
35.11
29.15
21.46
TsN
F
F
1c
210 200 190 180 170 160 150 140 130 120 110 100 90 80 70 60 50 40 30 20 10 0 −10
f1 (ppm)

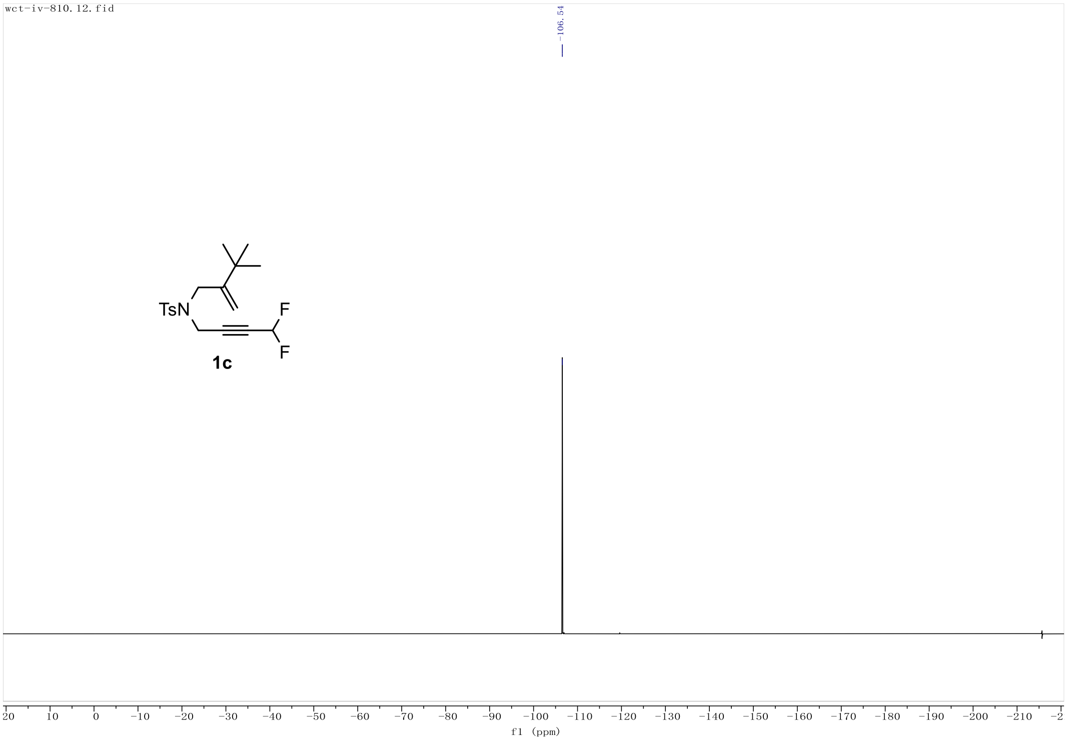
wct-iv-810.12.fid
-106.54
TsN
F
F
1c
20 10 0 −10 −20 −30 −40 −50 −60 −70 −80 −90 −100 −110 −120 −130 −140 −150 −160 −170 −180 −190 −200 −210
f1 (ppm)

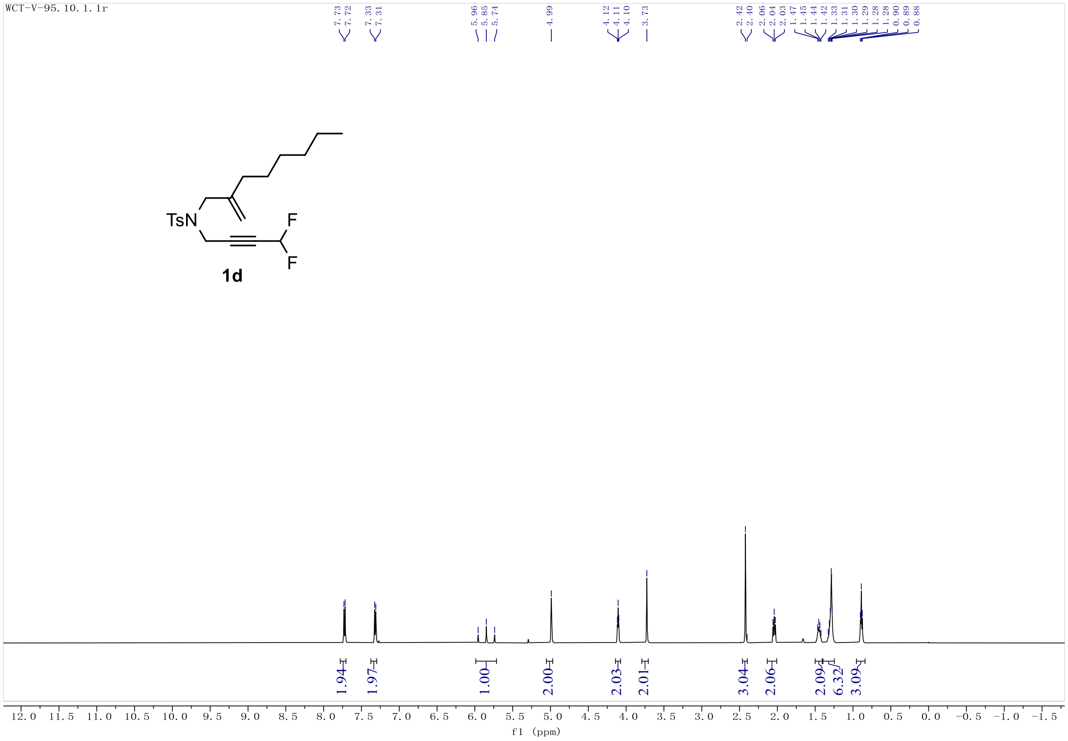
WCT-V-95.10.1.1r
7.73 7.72 7.33 7.31 5.96 5.85 5.74 4.99 4.12 4.11 4.10 3.73 2.42 2.40 2.06 2.04 2.03 1.47 1.45 1.44 1.42 1.33 1.31 1.30 1.29 1.28 0.90 0.89 0.88
TsN
F
F
1d
1.94 1.97 1.00 2.00 2.03 2.01 3.04 2.06 2.09 6.32 3.09
12.0 11.5 11.0 10.5 10.0 9.5 9.0 8.5 8.0 7.5 7.0 6.5 6.0 5.5 5.0 4.5 4.0 3.5 3.0 2.5 2.0 1.5 1.0 0.5 0.0 −0.5 −1.0 −1.5
f1 (ppm)

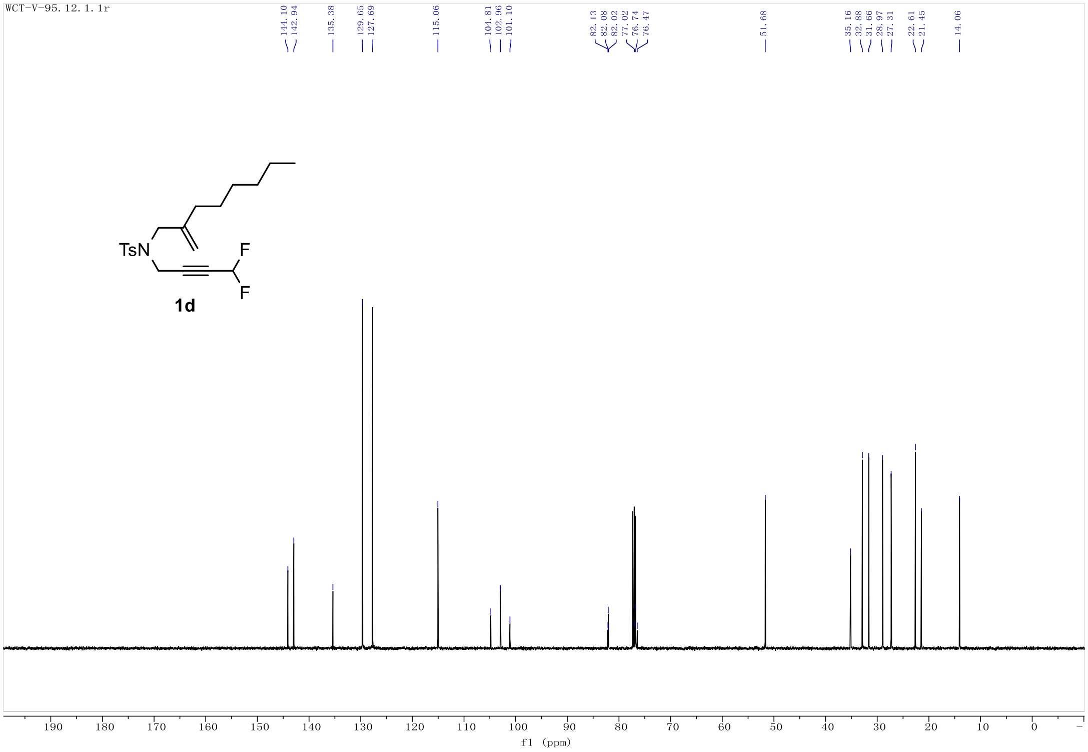
WCT-V-95.12.1.1r
144.10
142.94
135.38
129.65
127.69
115.06
104.81
102.96
101.10
82.13
82.08
82.02
77.02
76.74
76.47
51.68
35.16
32.88
31.66
28.97
27.31
22.61
21.45
14.06
TsN
F
F
1d
f1 (ppm)

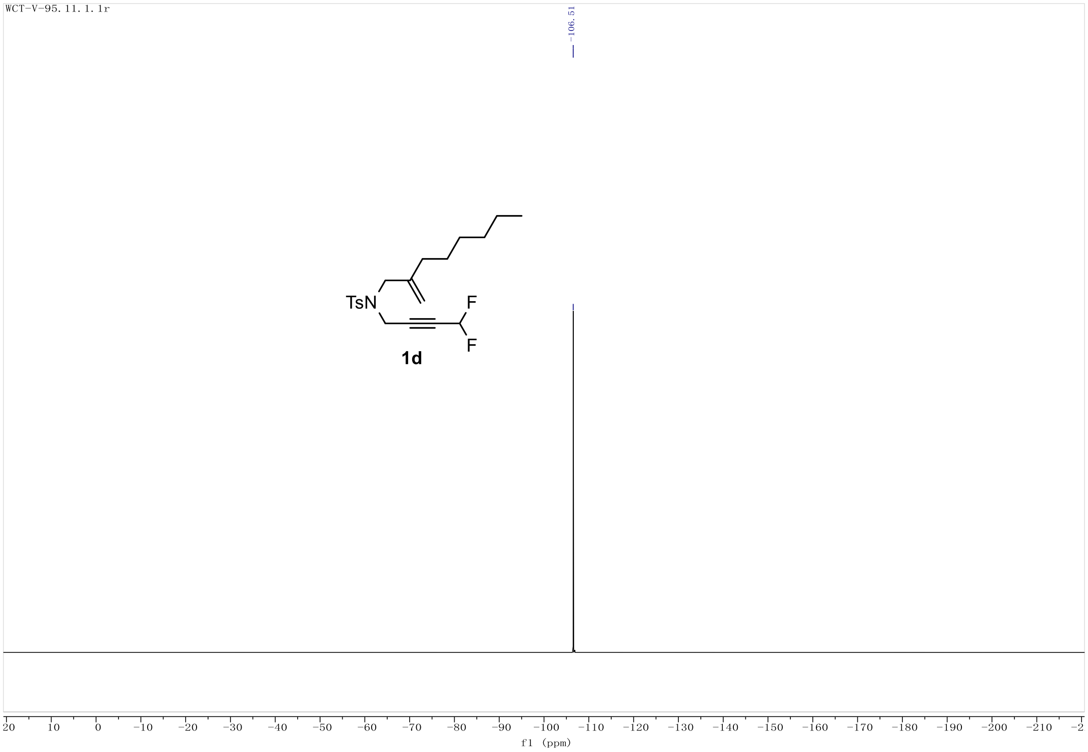
WCT-V-95.11.1.1r
-106.51
TsN
F
F
1d
f1 (ppm)

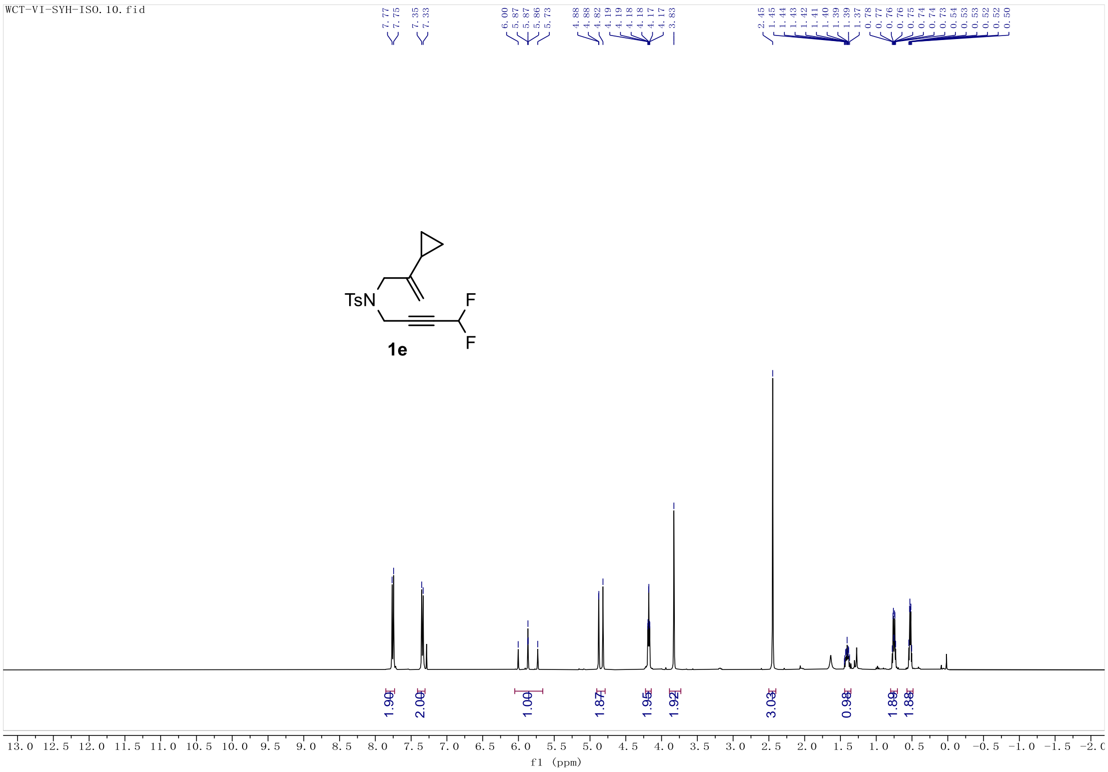
WCT-VI-SYH-ISO.10.fid
7.77
7.75
7.35
7.33
6.00
5.87
5.87
5.86
5.73
4.88
4.88
4.82
4.19
4.19
4.18
4.18
4.17
4.17
3.83
2.45
1.45
1.44
1.43
1.42
1.41
1.40
1.39
1.39
1.37
0.78
0.77
0.76
0.76
0.75
0.74
0.74
0.73
0.54
0.53
0.53
0.52
0.52
0.50
TsN
F
F
1e
1.90
2.00
1.00
1.87
1.95
1.92
3.03
0.98
1.89
1.88
f1 (ppm)

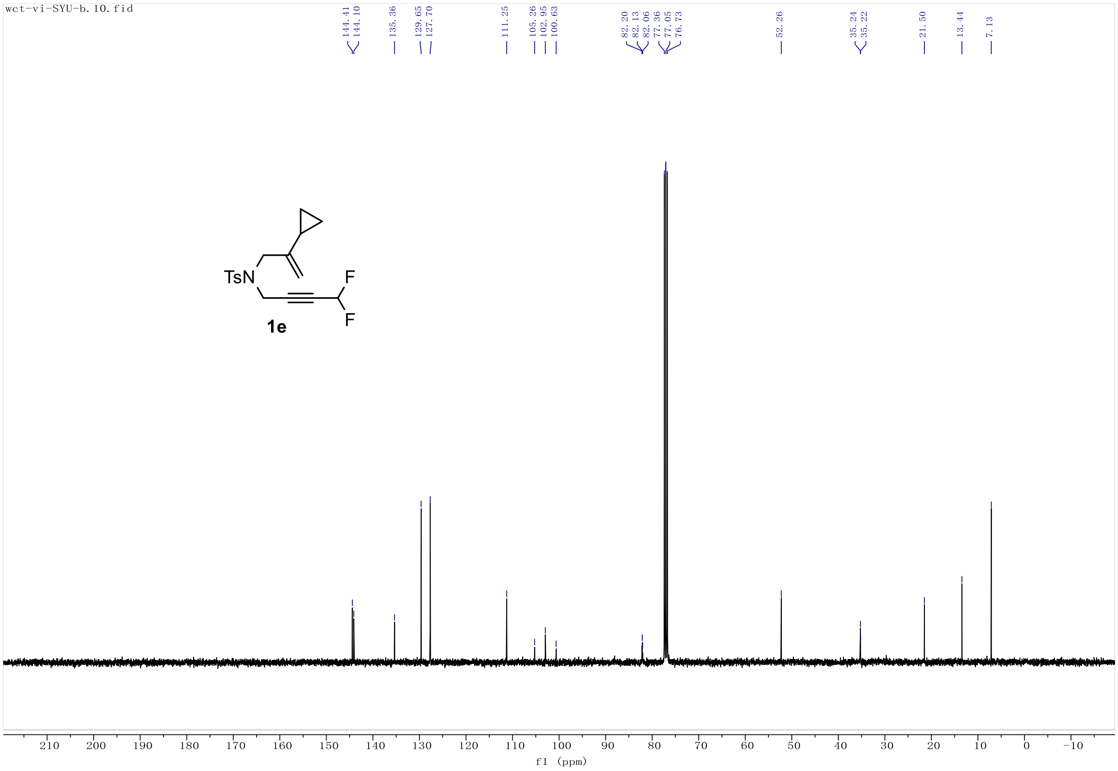
wct-vi-SYU-b.10.fid
144.41
144.10
135.36
129.65
127.70
111.25
105.26
102.95
100.63
82.20
82.13
82.06
77.36
77.05
76.73
52.26
35.24
35.22
21.50
13.44
7.13
TsN
F
F
1e
f1 (ppm)

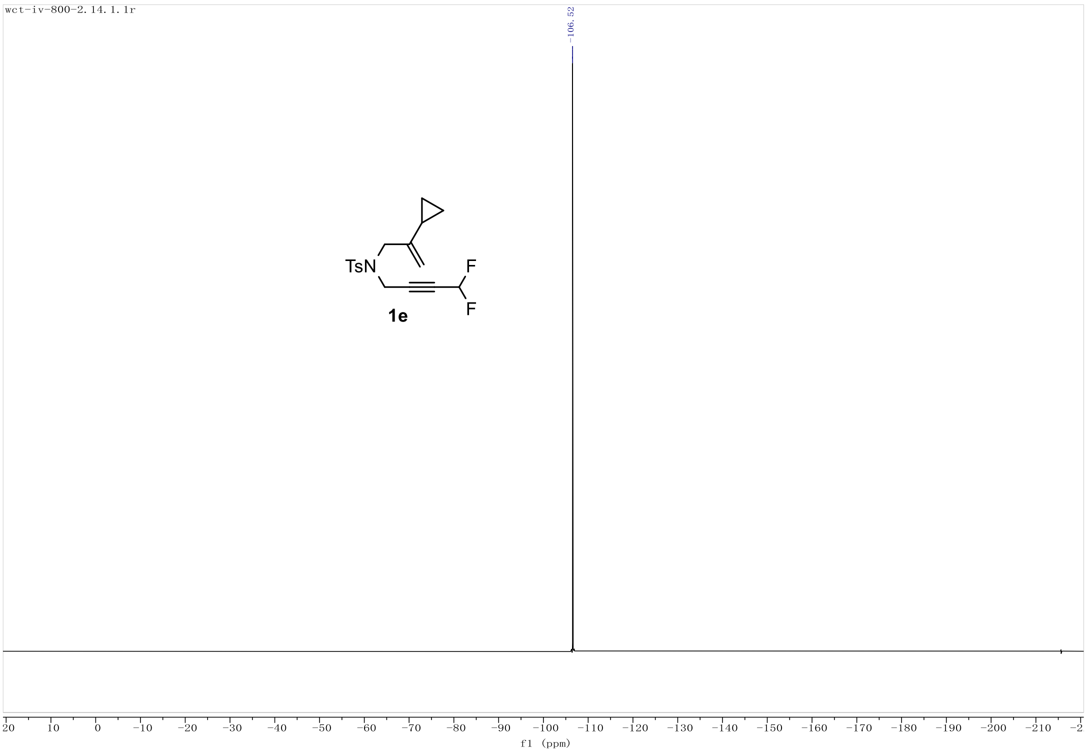
wct-iv-800-2.14.1.1r
-106.52
TsN
F
F
1e
20 10 0 −10 −20 −30 −40 −50 −60 −70 −80 −90 −100 −110 −120 −130 −140 −150 −160 −170 −180 −190 −200 −210 −2
f1 (ppm)

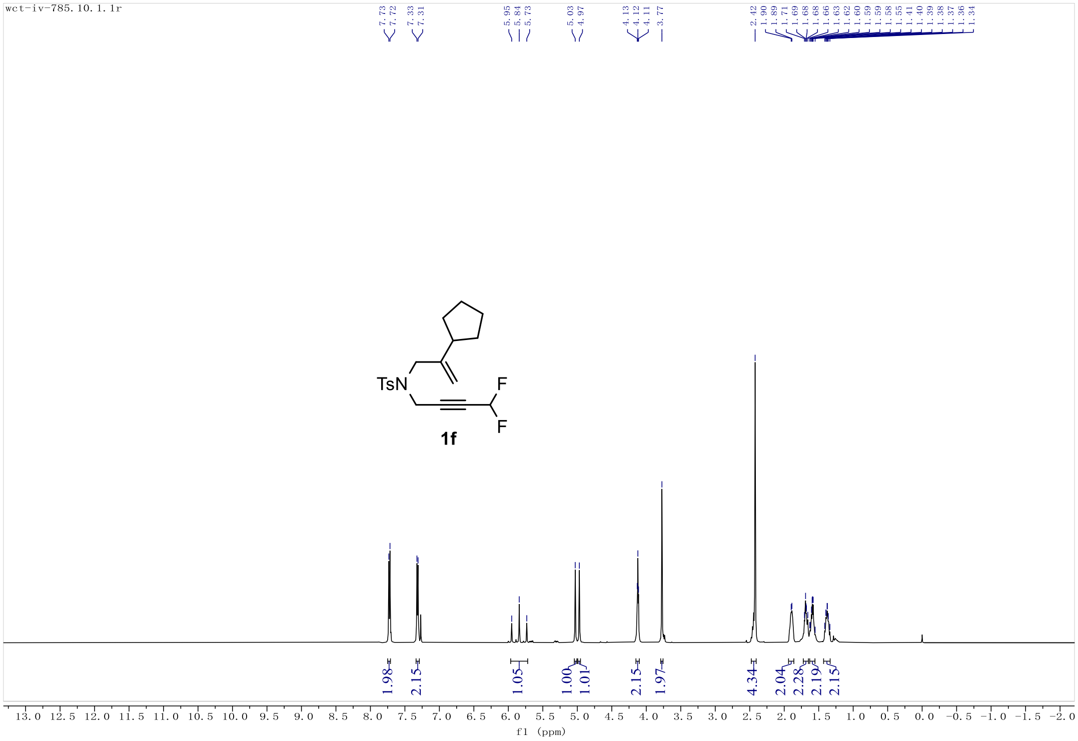
wct-iv-785.10.1.1r
7.73 7.72 7.33 7.31 5.95 5.84 5.73 5.03 4.97 4.13 4.12 4.11 3.77 2.42 1.90 1.89 1.71 1.69 1.68 1.68 1.66 1.63 1.62 1.60 1.59 1.59 1.58 1.55 1.41 1.40 1.39 1.38 1.37 1.36 1.34
TsN
F
F
1f
1.98 2.15 1.05 1.00 1.01 2.15 1.97 4.34 2.04 2.28 2.19 2.15
13.0 12.5 12.0 11.5 11.0 10.5 10.0 9.5 9.0 8.5 8.0 7.5 7.0 6.5 6.0 5.5 5.0 4.5 4.0 3.5 3.0 2.5 2.0 1.5 1.0 0.5 0.0 −0.5 −1.0 −1.5 −2.0
f1 (ppm)

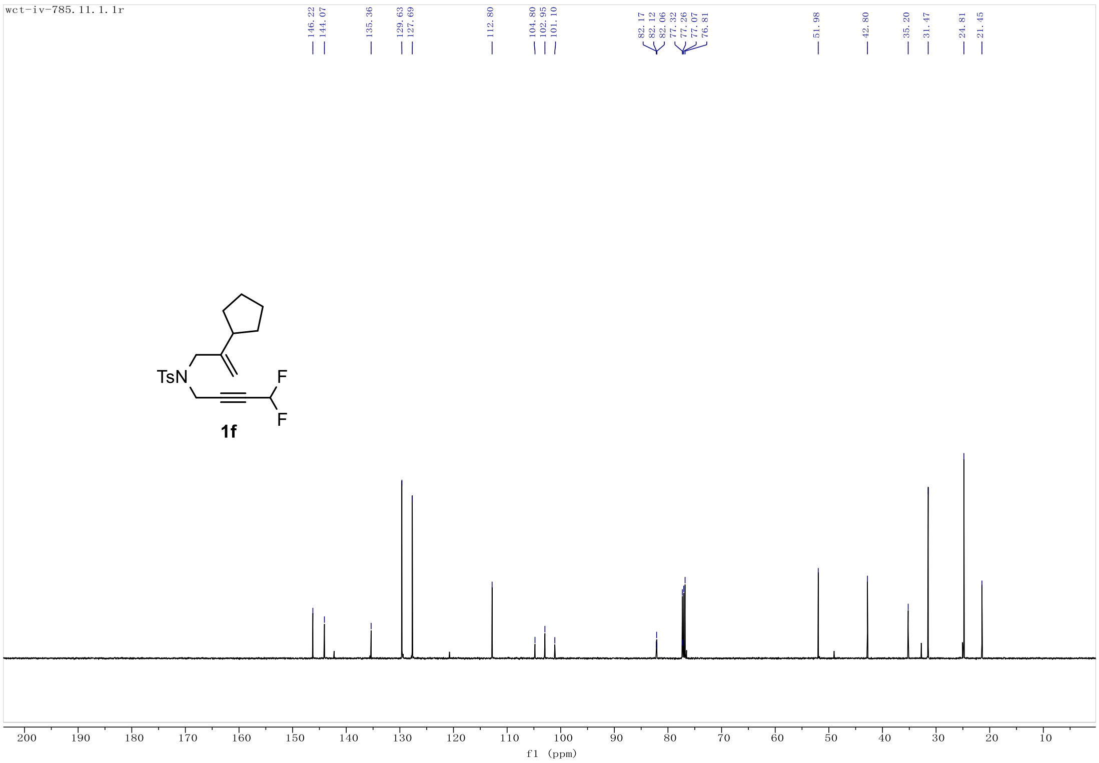
wct-iv-785.11.1.1r
146.22
144.07
135.36
129.63
127.69
112.80
104.80
102.95
101.10
82.17
82.12
82.06
77.32
77.26
77.07
76.81
51.98
42.80
35.20
31.47
24.81
21.45
TsN
F
F
1f
f1 (ppm)

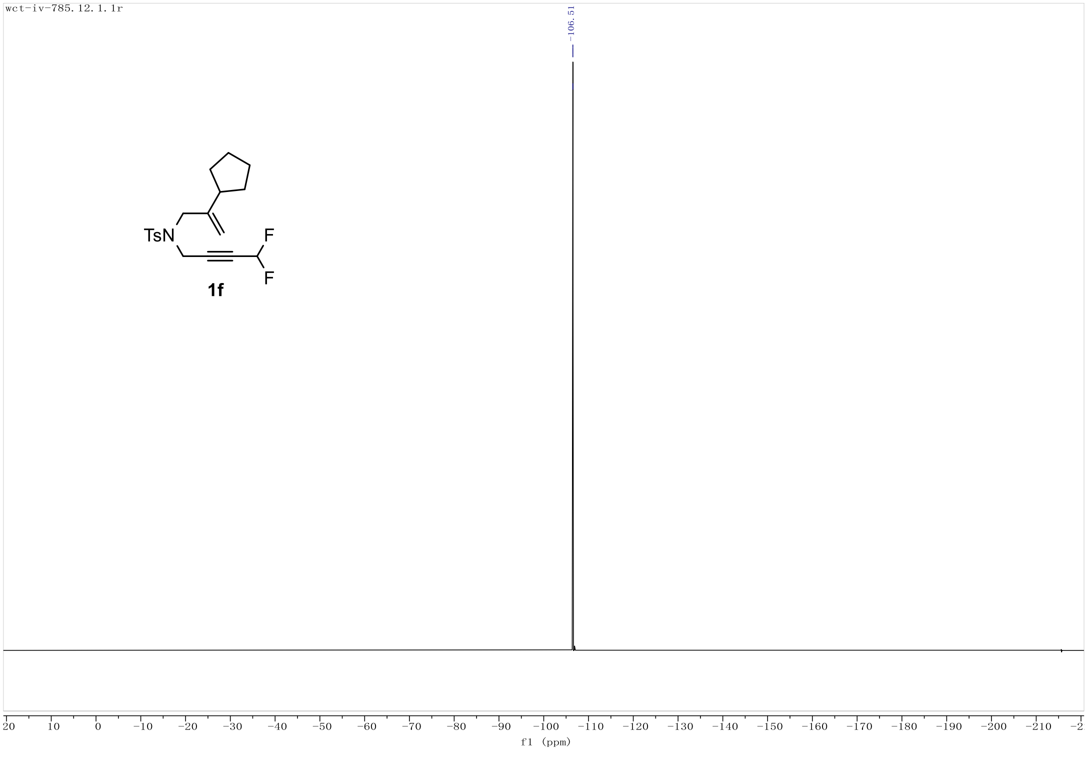
wct-iv-785.12.1.1r
-106.51
TsN
F
F
1f
f1 (ppm)

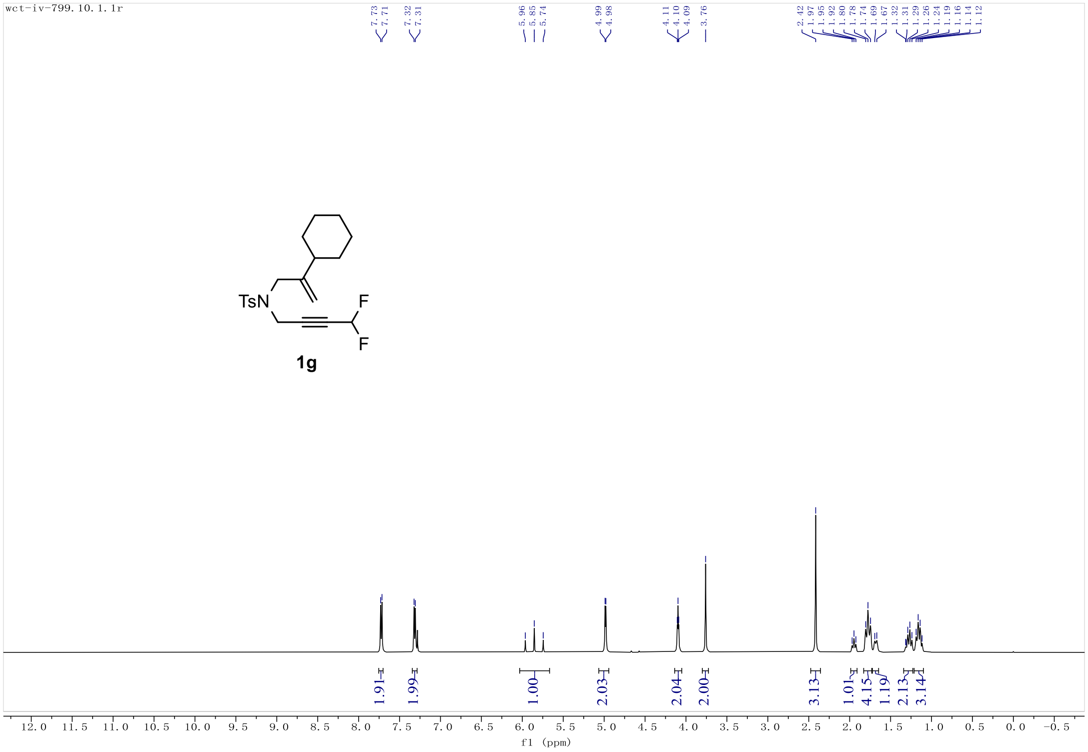
wct-iv-799.10.1.1r
7.73
7.71
7.32
7.31
5.96
5.85
5.74
4.99
4.98
4.11
4.10
4.09
3.76
2.42
1.97
1.95
1.92
1.80
1.78
1.74
1.69
1.67
1.32
1.31
1.29
1.26
1.24
1.19
1.16
1.14
1.12
TsN
F
F
1g
1.91
1.99
1.00
2.03
2.04
2.00
3.13
1.01
4.15
1.19
2.13
3.14
f1 (ppm)

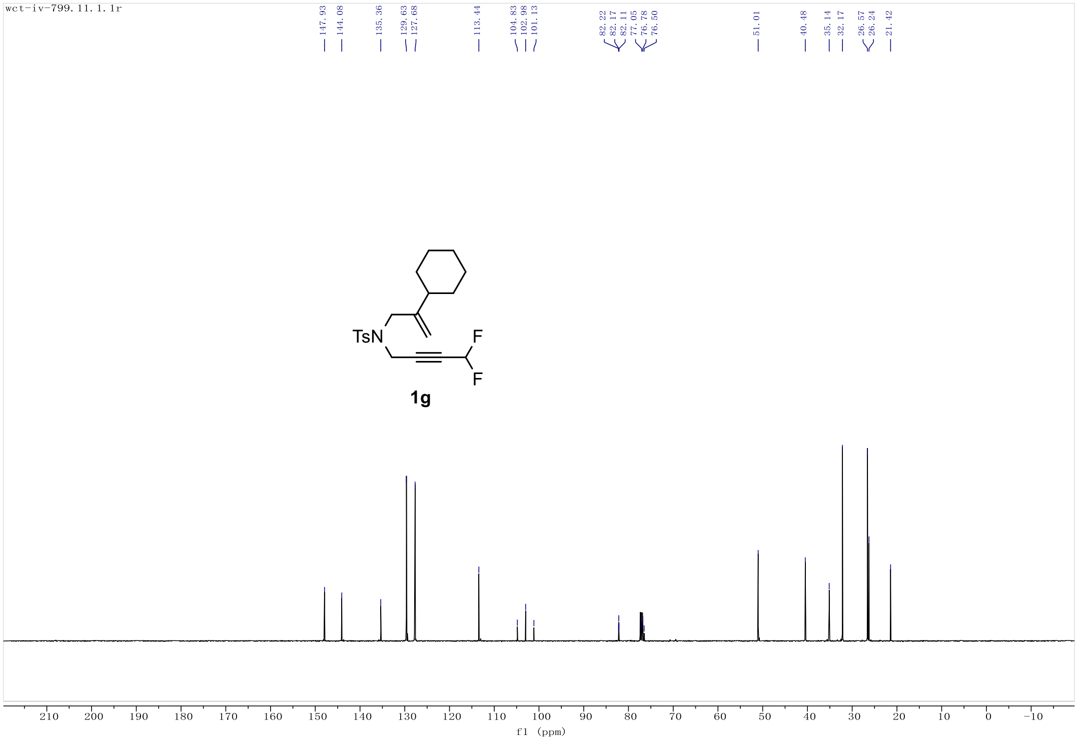
wct-iv-799.11.1.1r
147.93
144.08
135.36
129.63
127.68
113.44
104.83
102.98
101.13
82.22
82.17
82.11
77.05
76.78
76.50
51.01
40.48
35.14
32.17
26.57
26.24
21.42
TsN
F
F
1g
f1 (ppm)

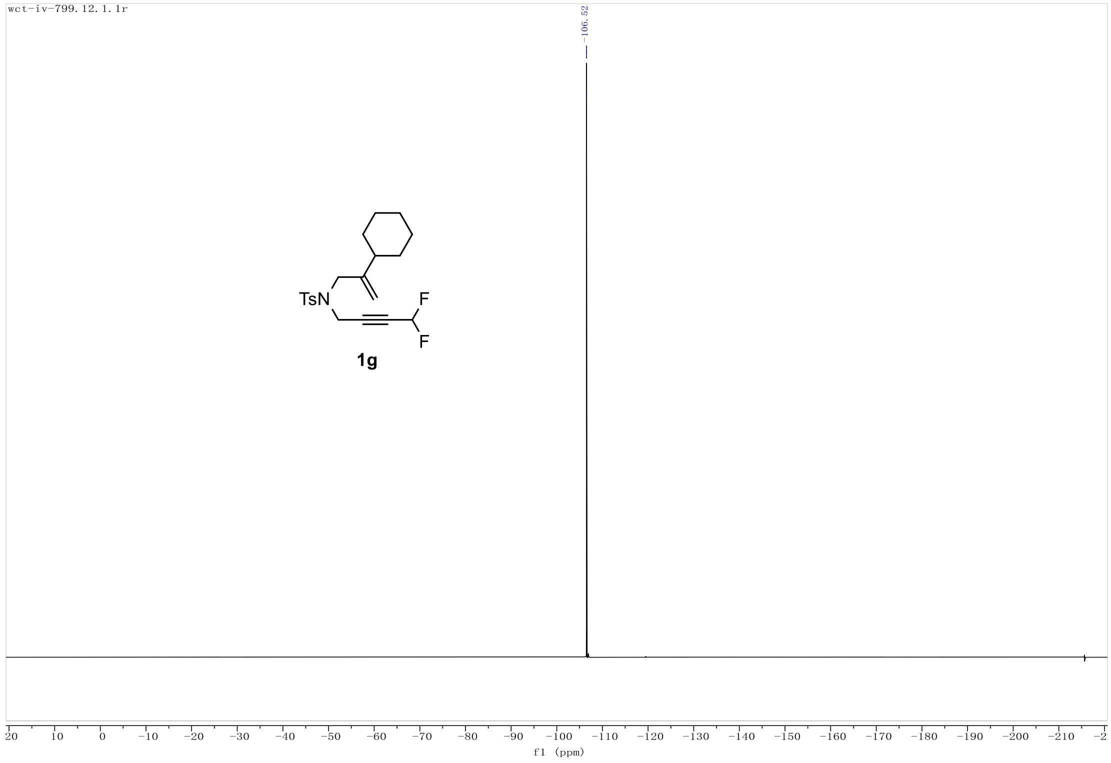
wct-iv-799.12.1.1r
-106.52
TsN
F
F
1g
f1 (ppm)

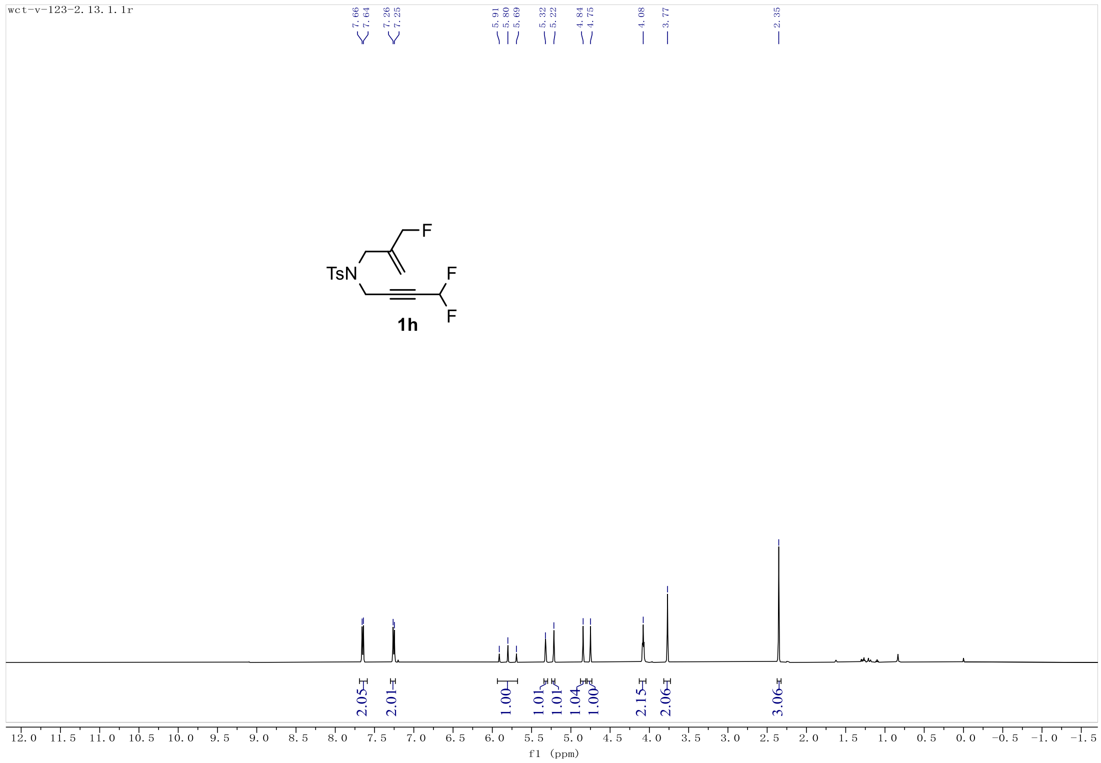
wct-v-123-2.13.1.1r
7.66
7.64
7.26
7.25
5.91
5.80
5.69
5.32
5.22
4.84
4.75
4.08
3.77
2.35
F
TsN
F
F
1h
2.05
2.01
1.00
1.01
1.01
1.04
1.00
2.15
2.06
3.06
f1 (ppm)

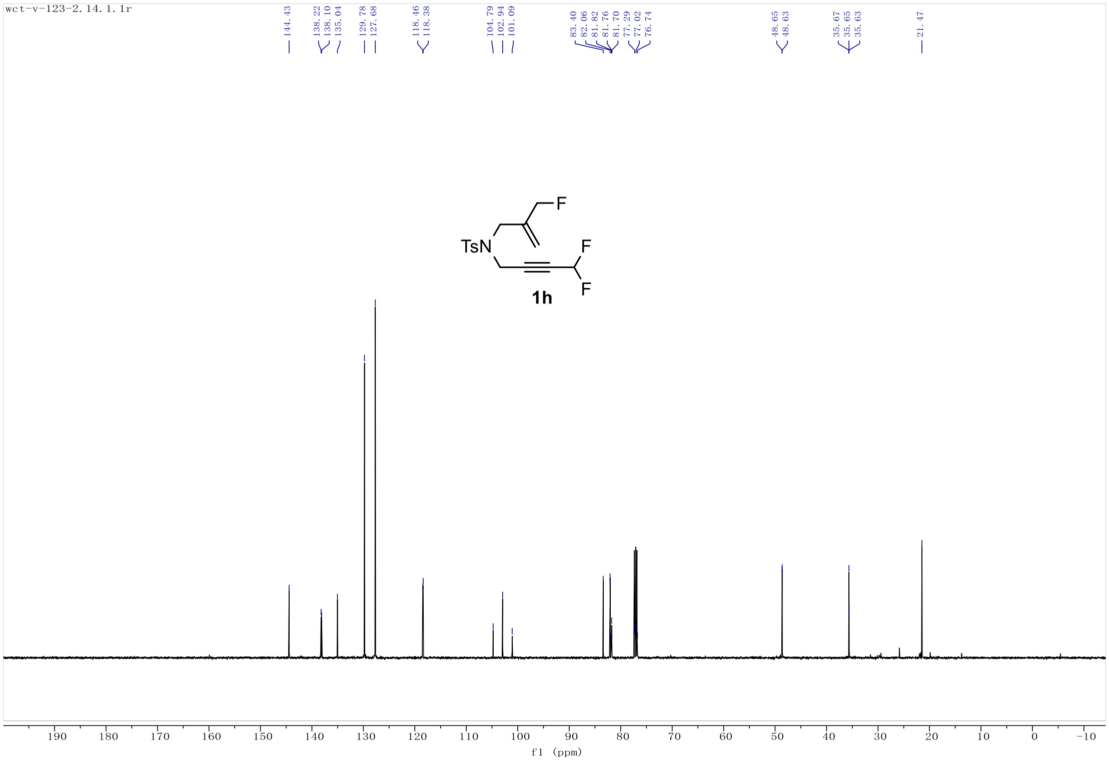
wct-v-123-2.14.1.1r
144.43
138.22
138.10
135.04
129.78
127.68
118.46
118.38
104.79
102.94
101.09
83.40
82.06
81.82
81.76
81.70
77.29
77.02
76.74
48.65
48.63
35.67
35.65
35.63
21.47
TsN
F
F
F
1h
f1 (ppm)

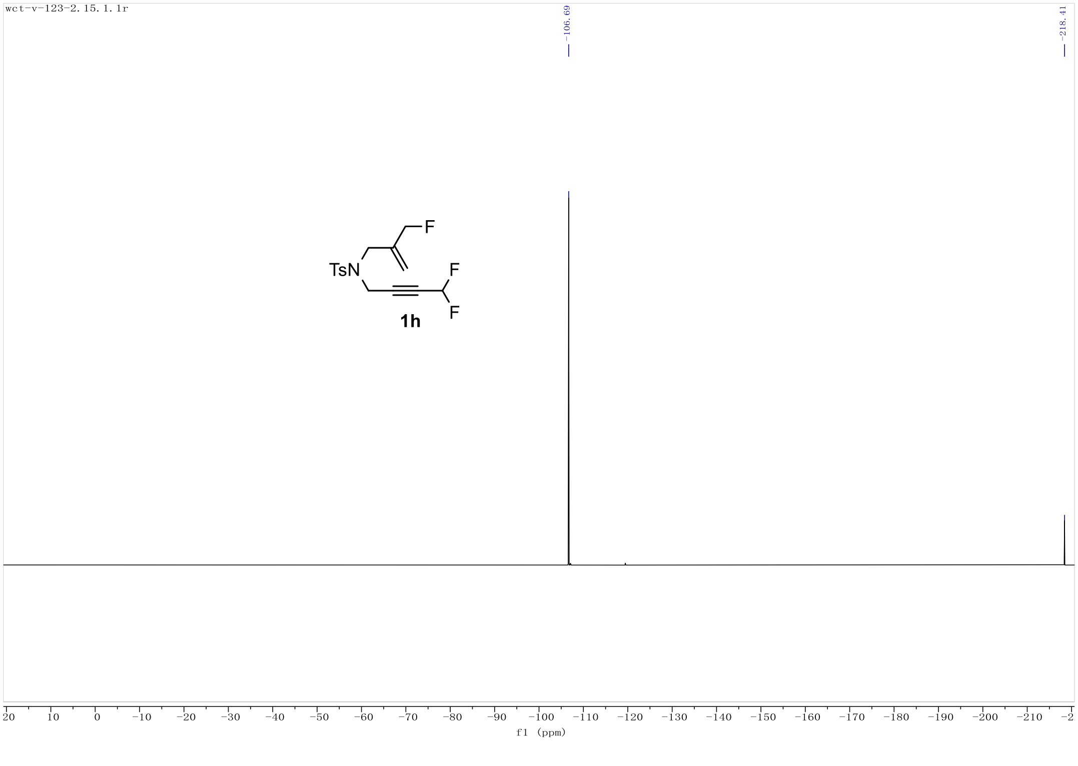
wct-v-123-2.15.1.1r
-106.69
-218.41
TsN
F
F
F
1h
f1 (ppm)

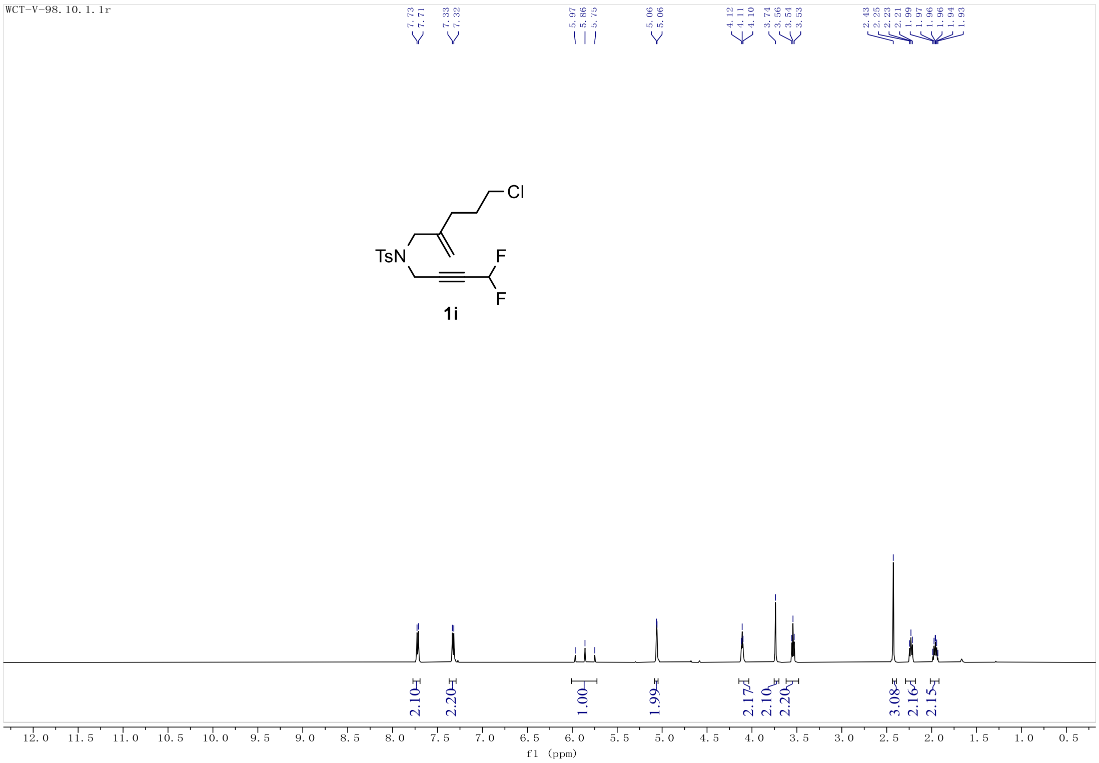
WCT-V-98.10.1.1r
7.73 7.71 7.33 7.32 5.97 5.86 5.75 5.06 5.06 4.12 4.11 4.10 3.74 3.56 3.54 3.53 2.43 2.25 2.23 2.21 1.99 1.97 1.96 1.96 1.94 1.93
Cl
TsN
F
F
1i
2.10 2.20 1.00 1.99 2.17 2.10 2.20 3.08 2.16 2.15
12.0 11.5 11.0 10.5 10.0 9.5 9.0 8.5 8.0 7.5 7.0 6.5 6.0 5.5 5.0 4.5 4.0 3.5 3.0 2.5 2.0 1.5 1.0 0.5
f1 (ppm)

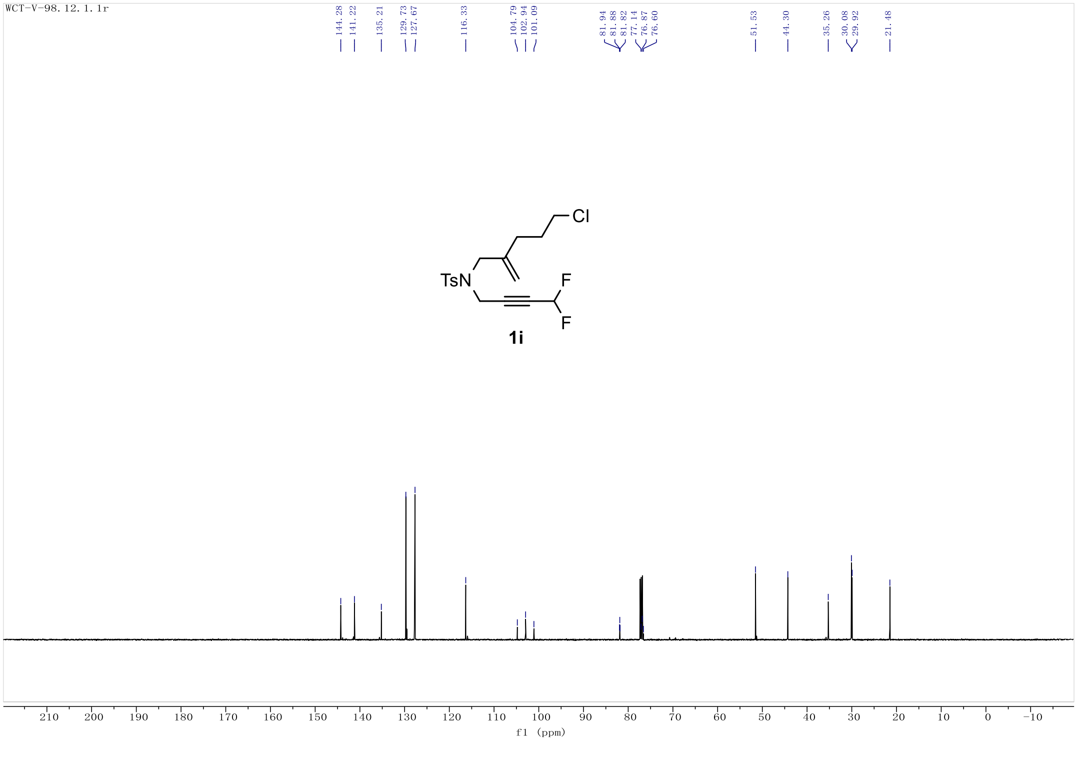
WCT-V-98.12.1.1r
144.28 141.22 135.21 129.73 127.67 116.33 104.79 102.94 101.09 81.94 81.88 81.82 77.14 76.87 76.60 51.53 44.30 35.26 30.08 29.92 21.48
Cl
TsN
F
F
1i
210 200 190 180 170 160 150 140 130 120 110 100 90 80 70 60 50 40 30 20 10 0 -10
f1 (ppm)

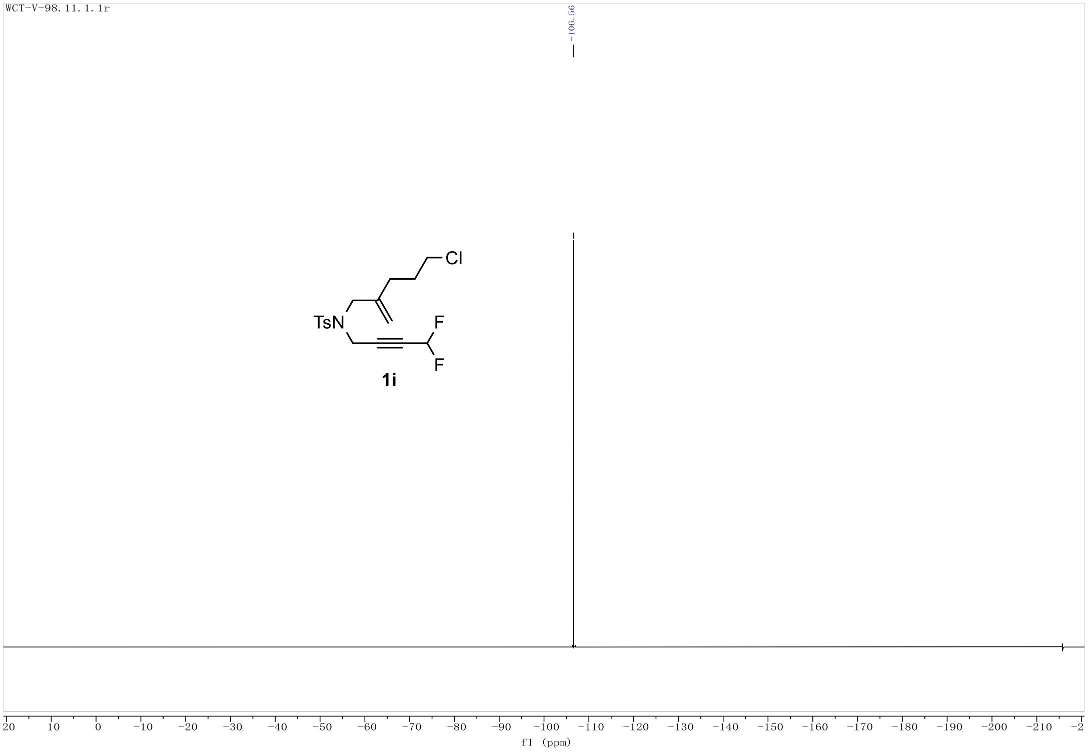

WCT-V-98.11.1.1r
-106.56
Cl
TsN
F
F
1i
f1 (ppm)

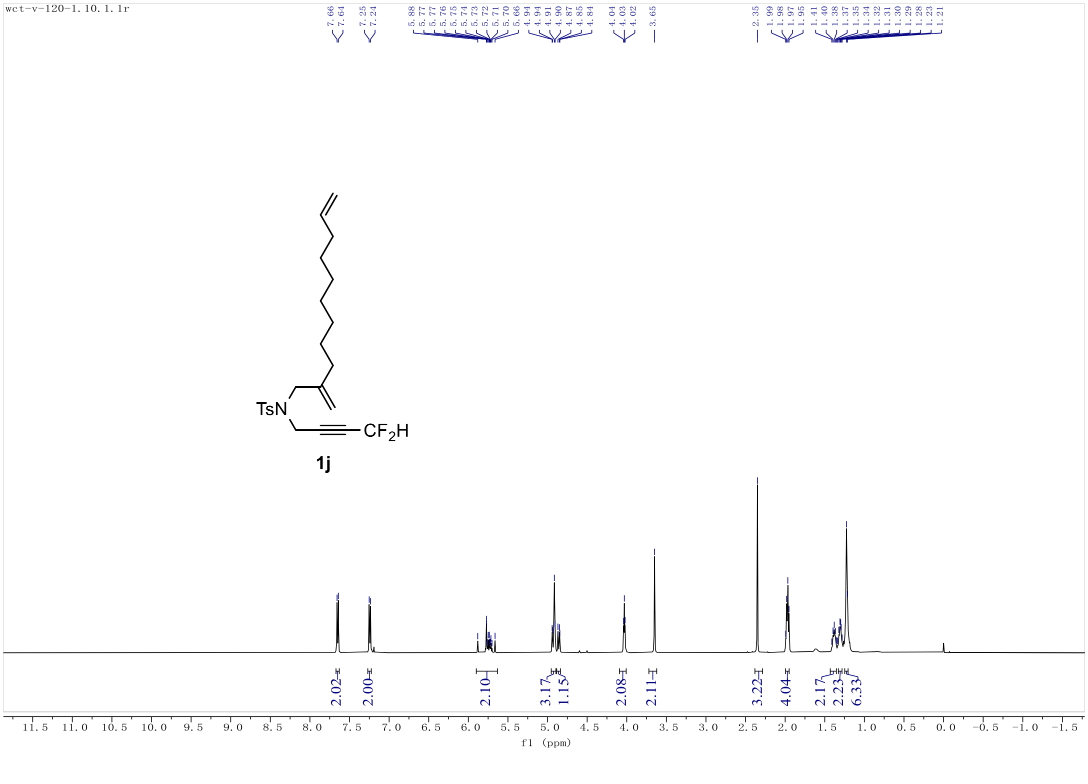

wct-v-120-1.10.1.1r
TsN
CF2H
1j
f1 (ppm)

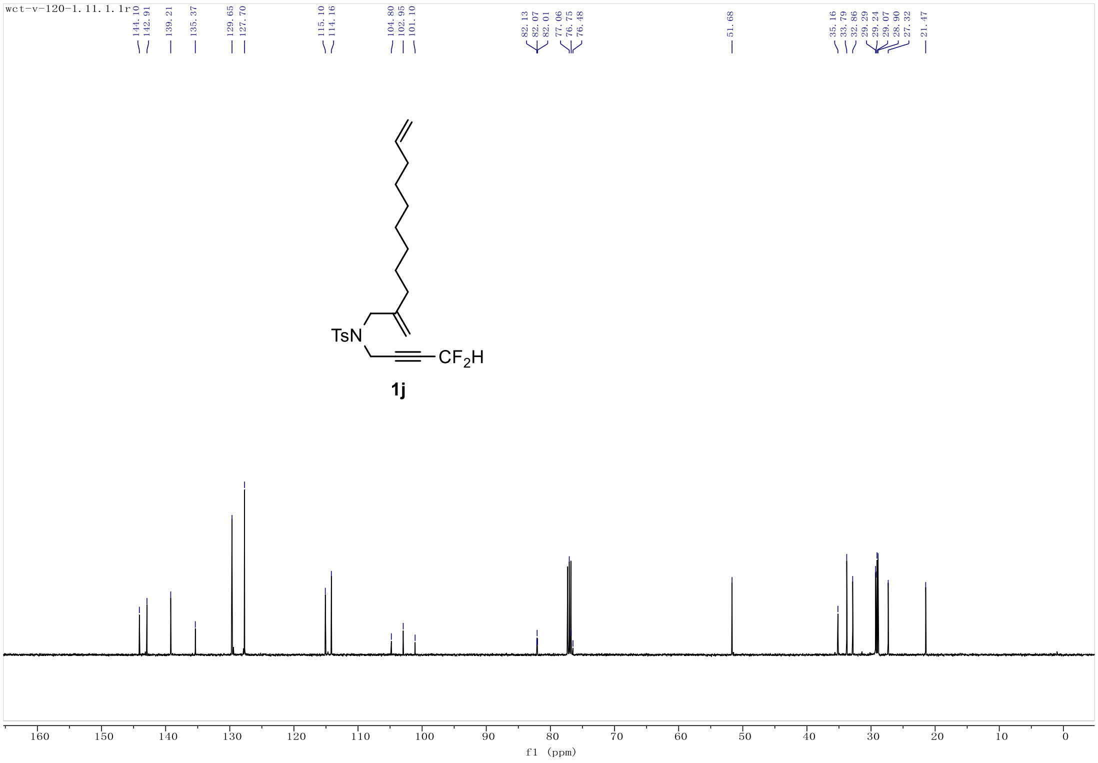
wct-v-120-1.11.1.1r
144.10
142.91
139.21
135.37
129.65
127.70
115.10
114.16
104.80
102.95
101.10
82.13
82.07
82.01
77.06
76.75
76.48
51.68
35.16
33.79
32.86
29.29
29.24
29.07
28.90
27.32
21.47
TsN
CF2H
1j
f1 (ppm)

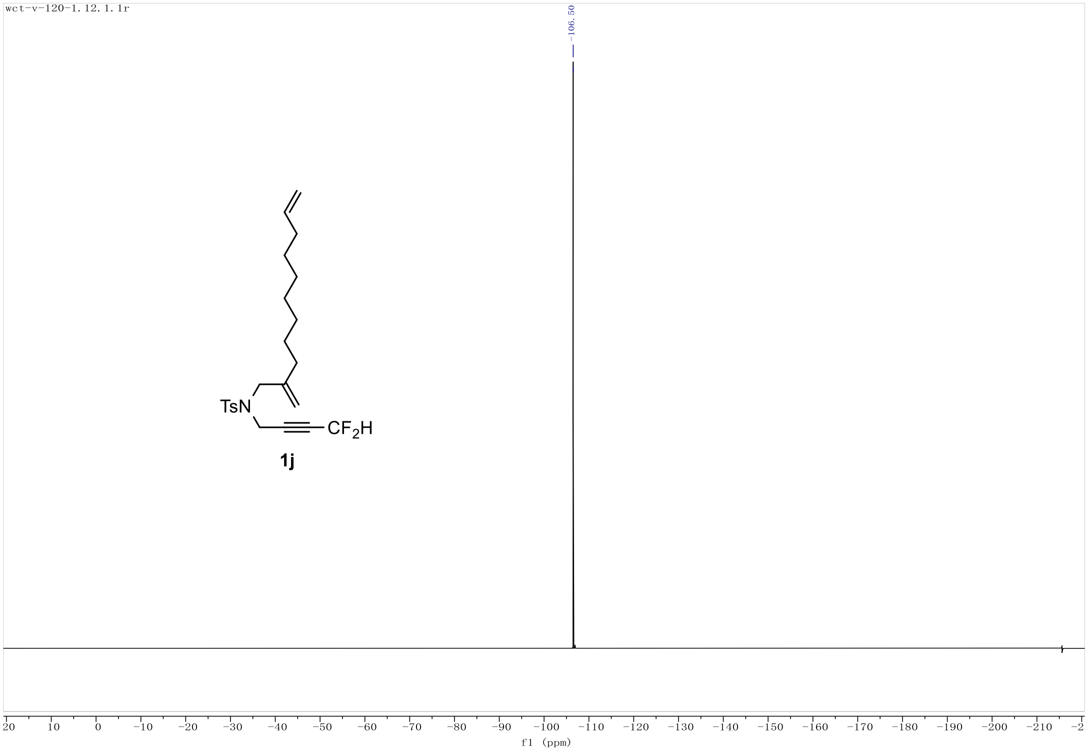
wct-v-120-1.12.1.1r
-106.50
TsN
CF2H
1j
f1 (ppm)

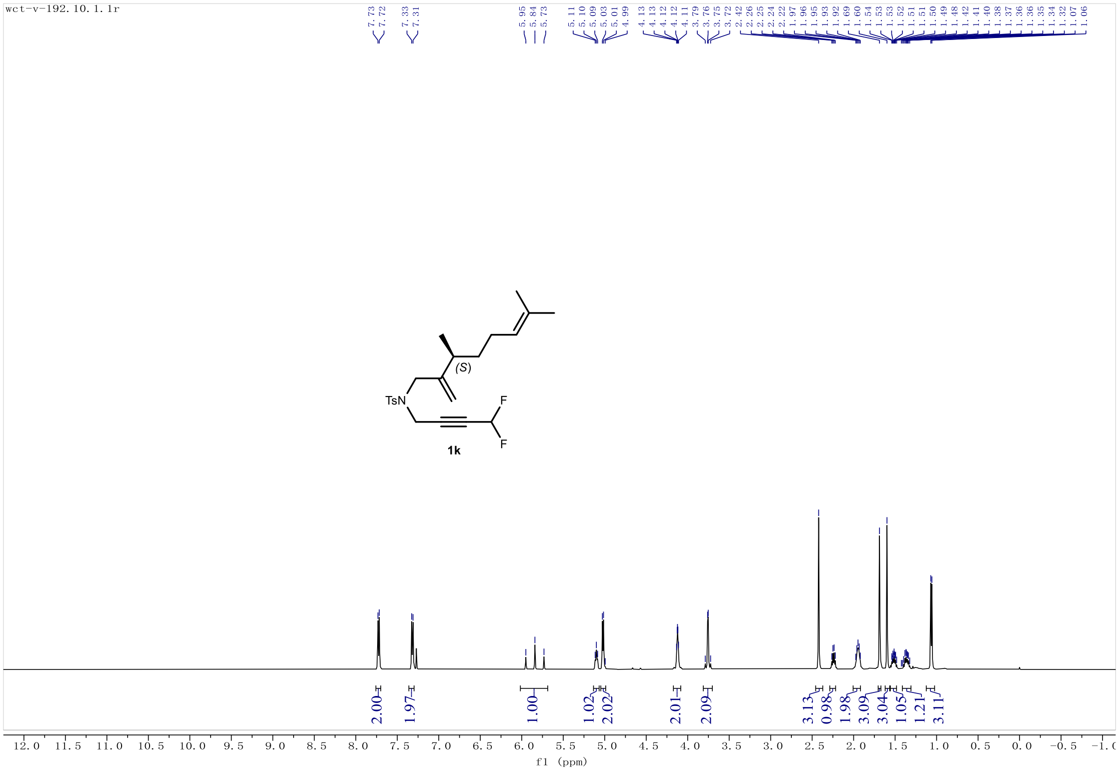
wct-v-192.10.1.1r
7.73 7.72 7.33 7.31 5.95 5.84 5.73 5.11 5.10 5.09 5.03 5.01 4.99 4.13 4.13 4.12 4.12 4.11 3.79 3.76 3.75 3.72 2.42 2.26 2.25 2.24 2.22 1.97 1.96 1.95 1.93 1.92 1.69 1.60 1.54 1.53 1.53 1.52 1.51 1.51 1.50 1.49 1.48 1.42 1.41 1.40 1.38 1.37 1.36 1.36 1.35 1.34 1.32 1.07 1.06
TsN
(S)
F
F
1k
2.00 1.97 1.00 1.02 2.02 2.01 2.09 3.13 0.98 1.98 3.09 3.04 1.05 1.21 3.11
f1 (ppm)

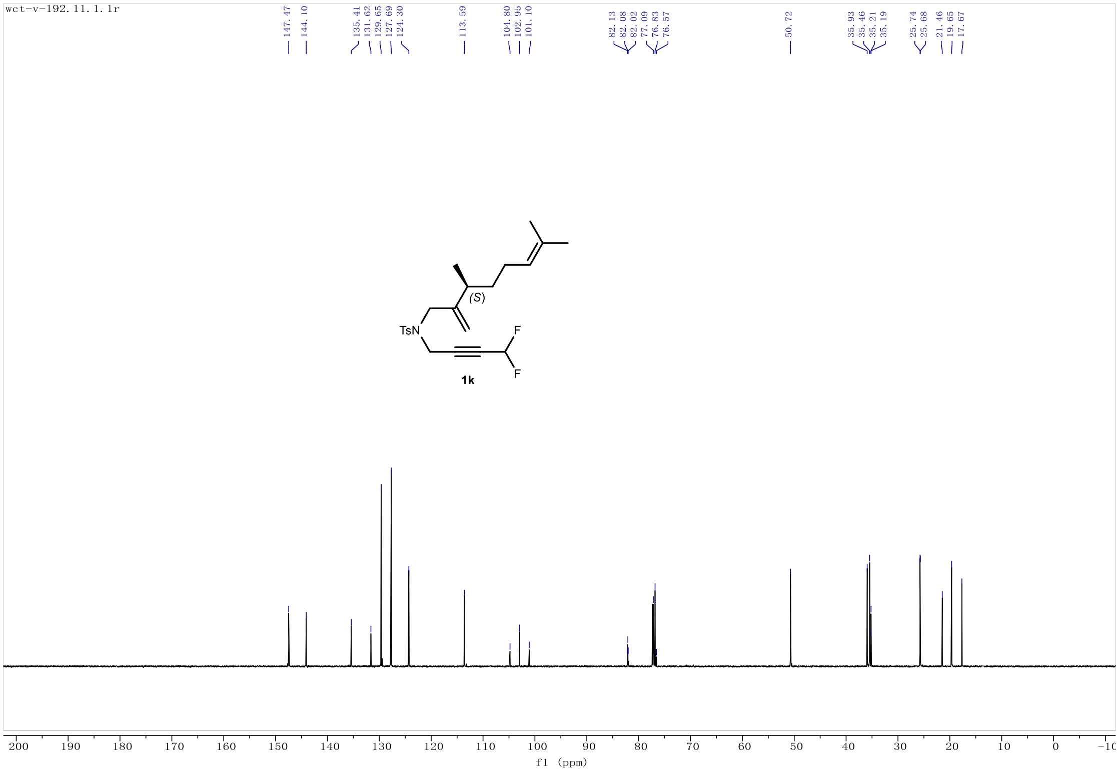
wct-v-192.11.1.1r
147.47 144.10 135.41 131.62 129.65 127.69 124.30 113.59 104.80 102.95 101.10 82.13 82.08 82.02 77.09 76.83 76.57 50.72 35.93 35.46 35.21 35.19 25.74 25.68 21.46 19.65 17.67
TsN
(S)
F
F
1k
f1 (ppm)

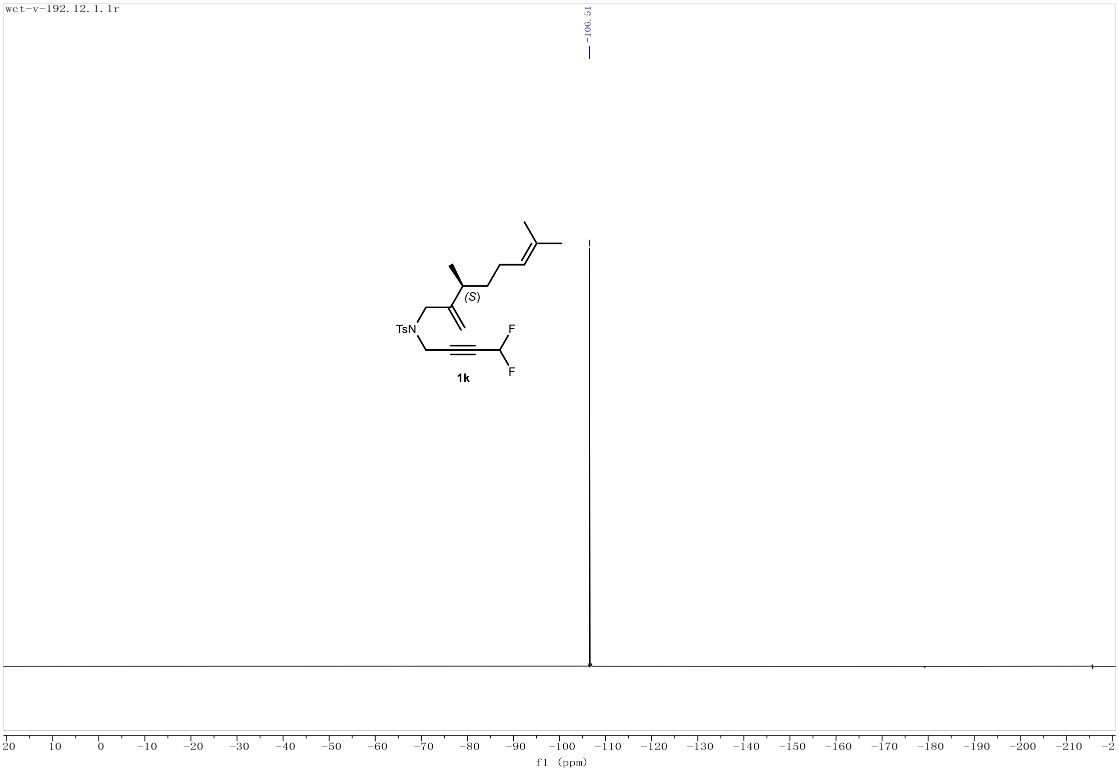
wct-v-192.12.1.1r
-106.51
(S)
TsN
F
F
1k
f1 (ppm)

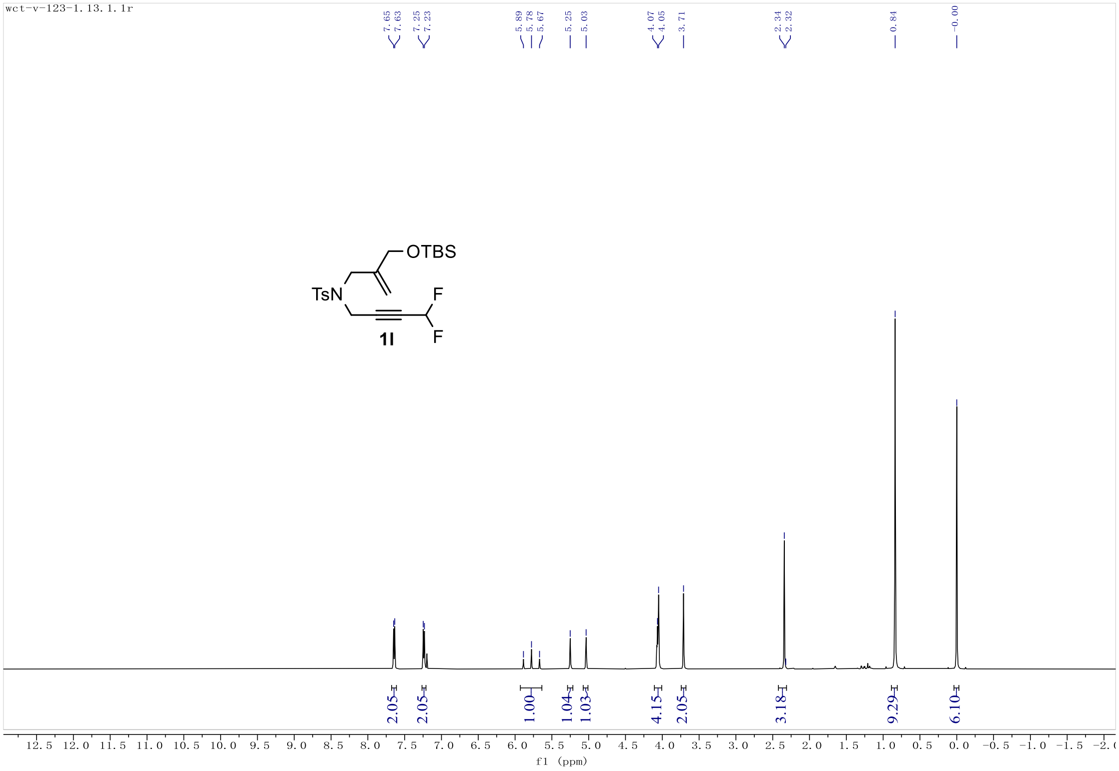
wct-v-123-1.13.1.1r
7.65
7.63
7.25
7.23
5.89
5.78
5.67
5.25
5.03
4.07
4.05
3.71
2.34
2.32
0.84
0.00
OTBS
TsN
F
F
1l
2.05
2.05
1.00
1.04
1.03
4.15
2.05
3.18
9.29
6.10
f1 (ppm)

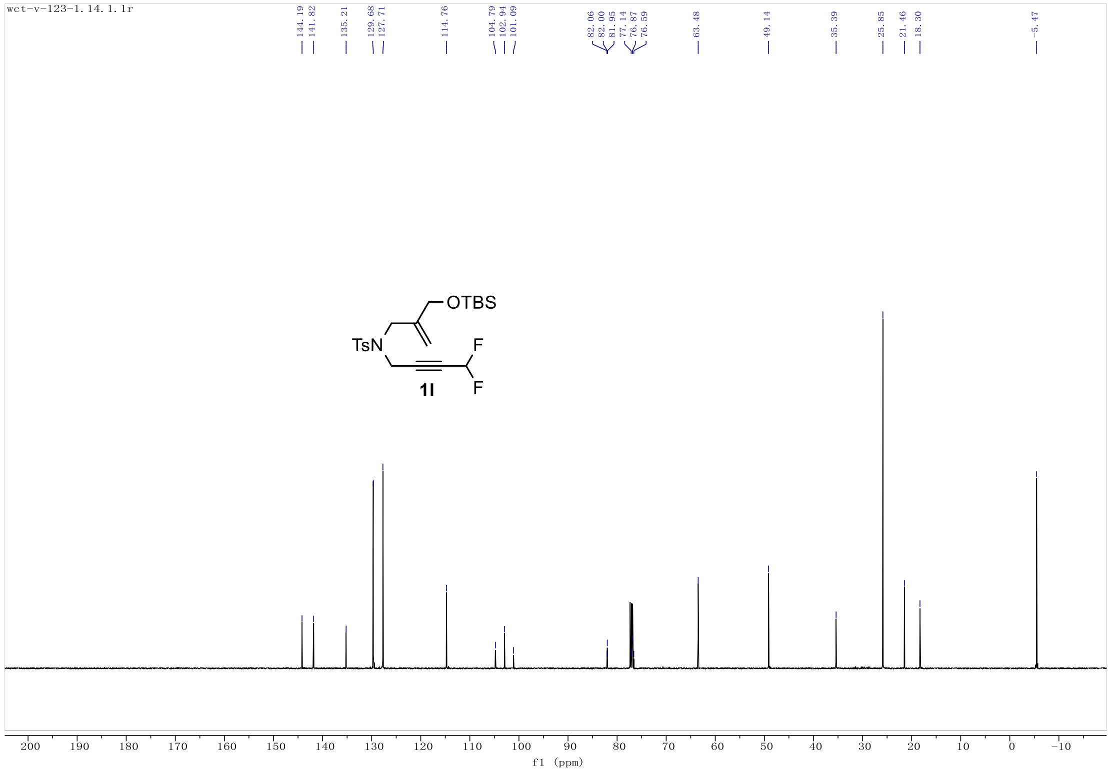
wct-v-123-1.14.1.1r
144.19
141.82
135.21
129.68
127.71
114.76
104.79
102.94
101.09
82.06
82.00
81.95
77.14
76.87
76.59
63.48
49.14
35.39
25.85
21.46
18.30
-5.47
OTBS
TsN
F
F
1l
f1 (ppm)

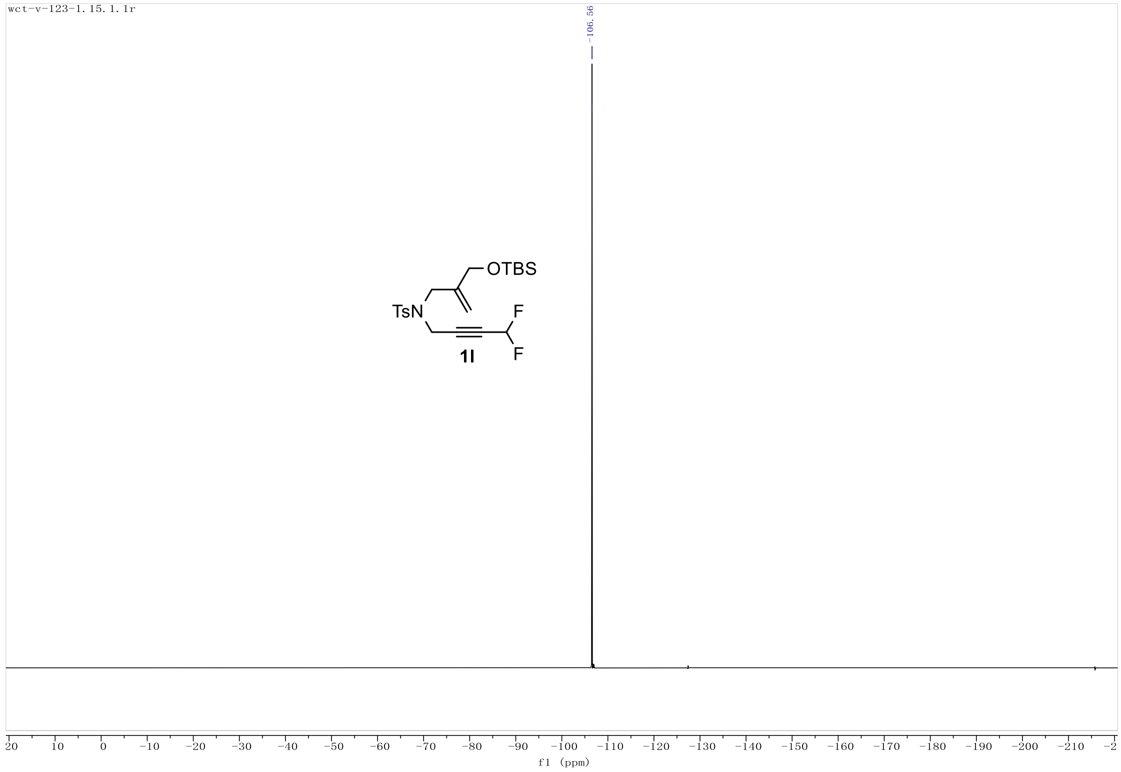
wct-v-123-1.15.1.1r
-106.56
OTBS
TsN
F
F
1l
f1 (ppm)

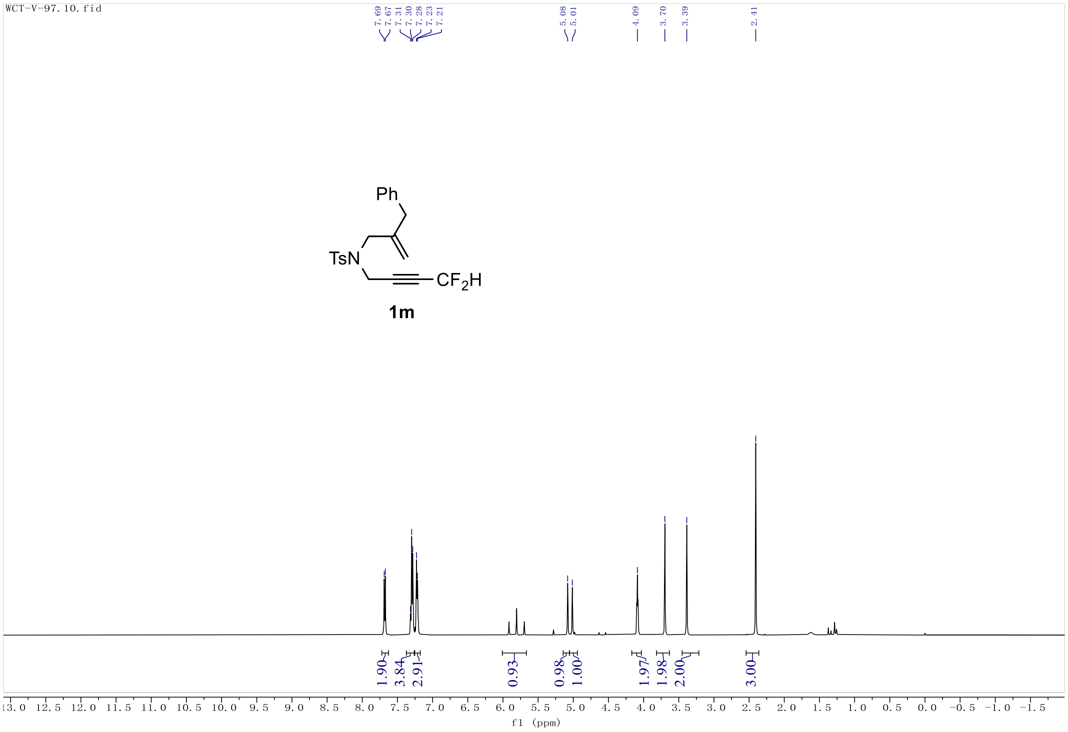
WCT-V-97.10.fid
7.69
7.67
7.31
7.30
7.28
7.23
7.21
5.08
5.01
4.09
3.70
3.39
2.41
Ph
TsN
CF2H
1m
1.90
3.84
2.91
0.93
0.98
1.00
1.97
1.98
2.00
3.00
f1 (ppm)

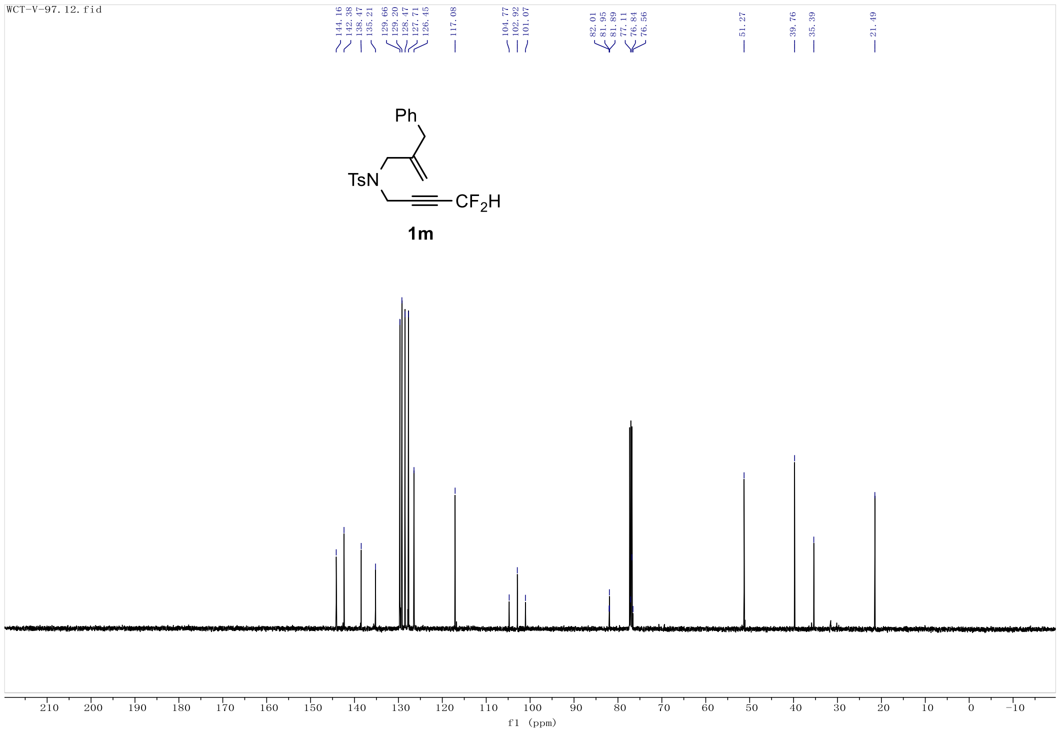
WCT-V-97.12.fid
144.16
142.38
138.47
135.21
129.66
129.20
128.47
127.71
126.45
117.08
104.77
102.92
101.07
82.01
81.95
81.89
77.11
76.84
76.56
51.27
39.76
35.39
21.49
Ph
TsN
CF2H
1m
f1 (ppm)

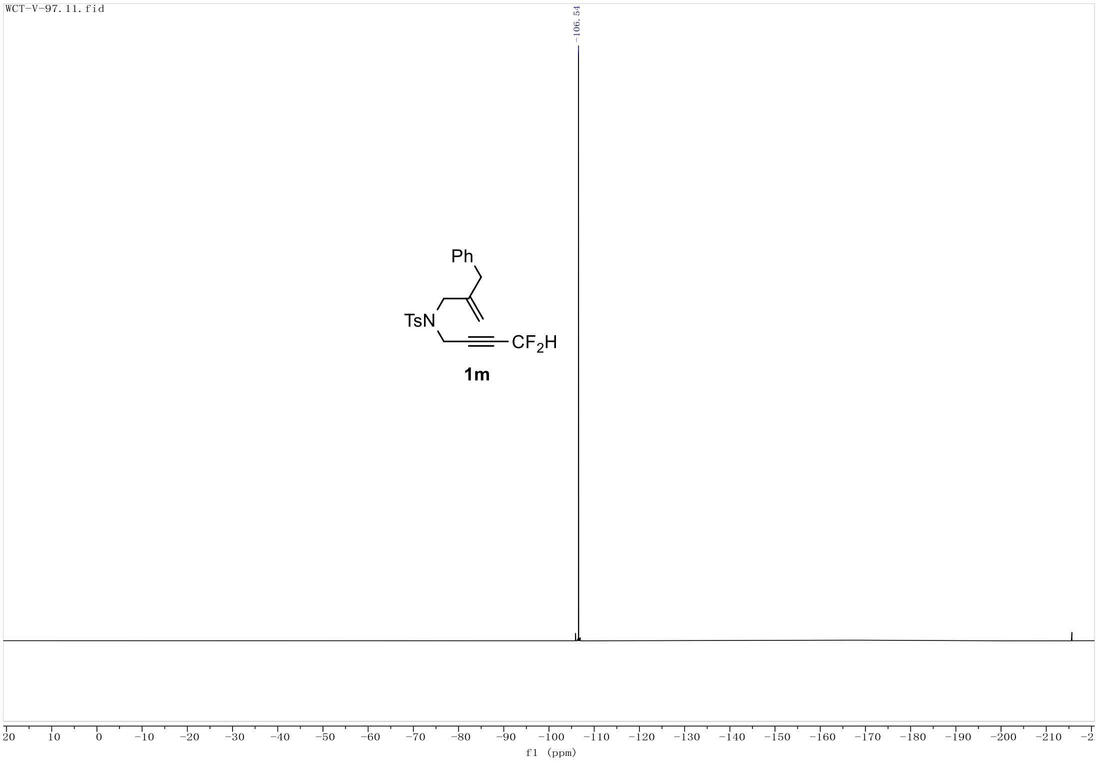
WCT-V-97.11.fid
-106.54
Ph
TsN
CF2H
1m
f1 (ppm)

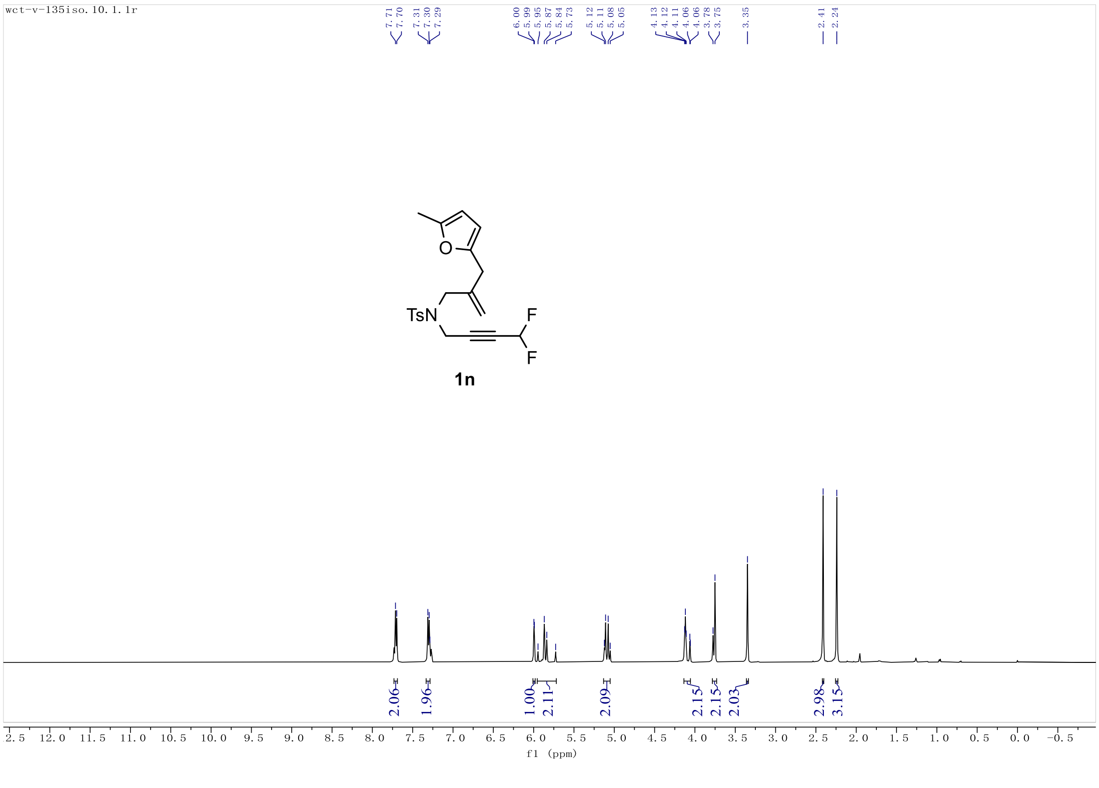
wct-v-135iso.10.1.1r
7.71
7.70
7.31
7.30
7.29
6.00
5.99
5.95
5.87
5.84
5.73
5.12
5.11
5.08
5.05
4.13
4.12
4.11
4.06
4.06
3.78
3.75
3.35
2.41
2.24
O
TsN
F
F
1n
2.06
1.96
1.00
2.11
2.09
2.15
2.15
2.03
2.98
3.15
f1 (ppm)

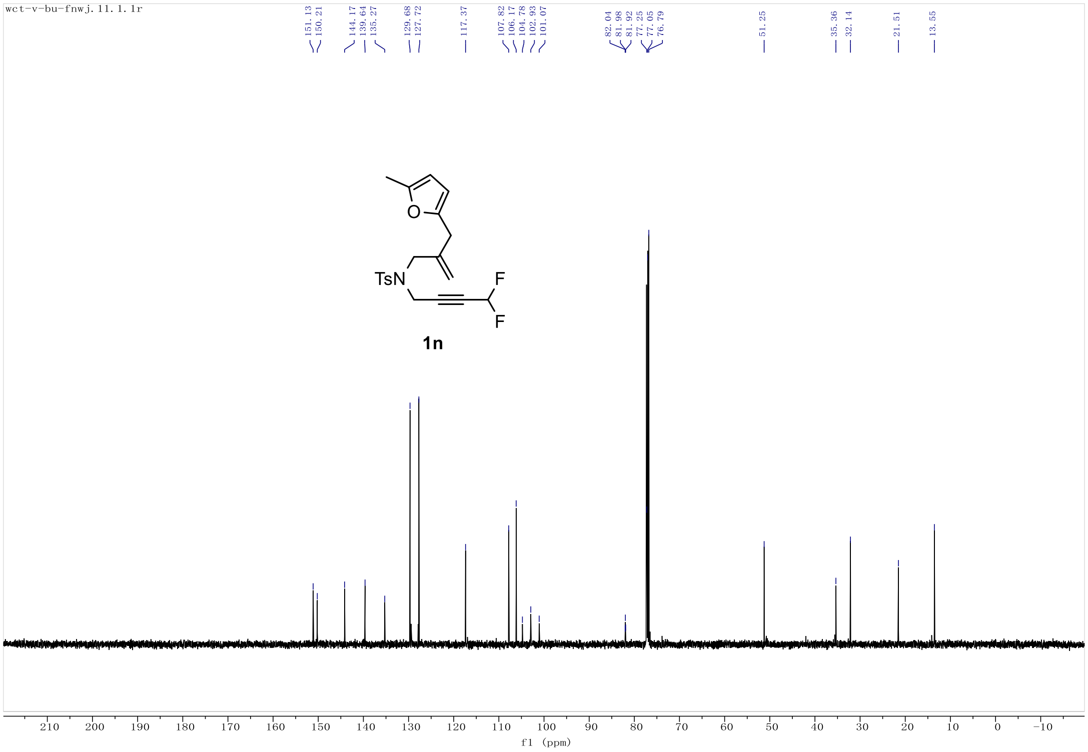
wct-v-bu-fnwj.11.1.1r
151.13
150.21
144.17
139.64
135.27
129.68
127.72
117.37
107.82
106.17
104.78
102.93
101.07
82.04
81.98
81.92
77.25
77.05
76.79
51.25
35.36
32.14
21.51
13.55
TsN
F
F
1n
f1 (ppm)

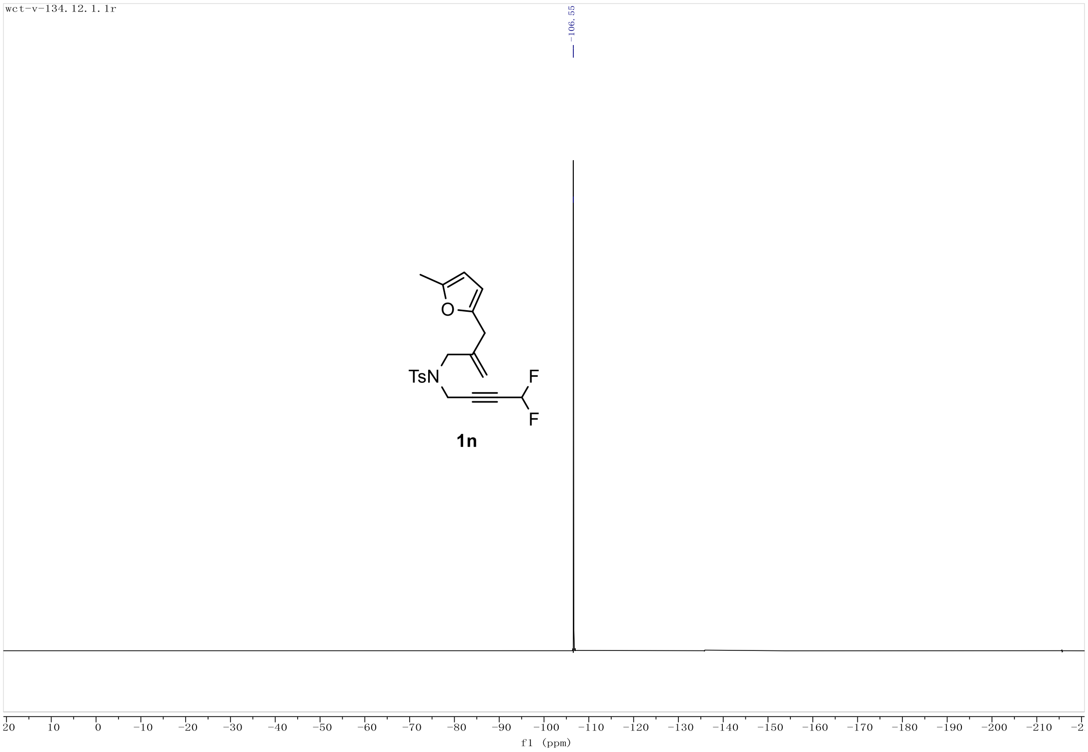
wct-v-134.12.1.1r
-106.55
TsN
F
F
1n
f1 (ppm)

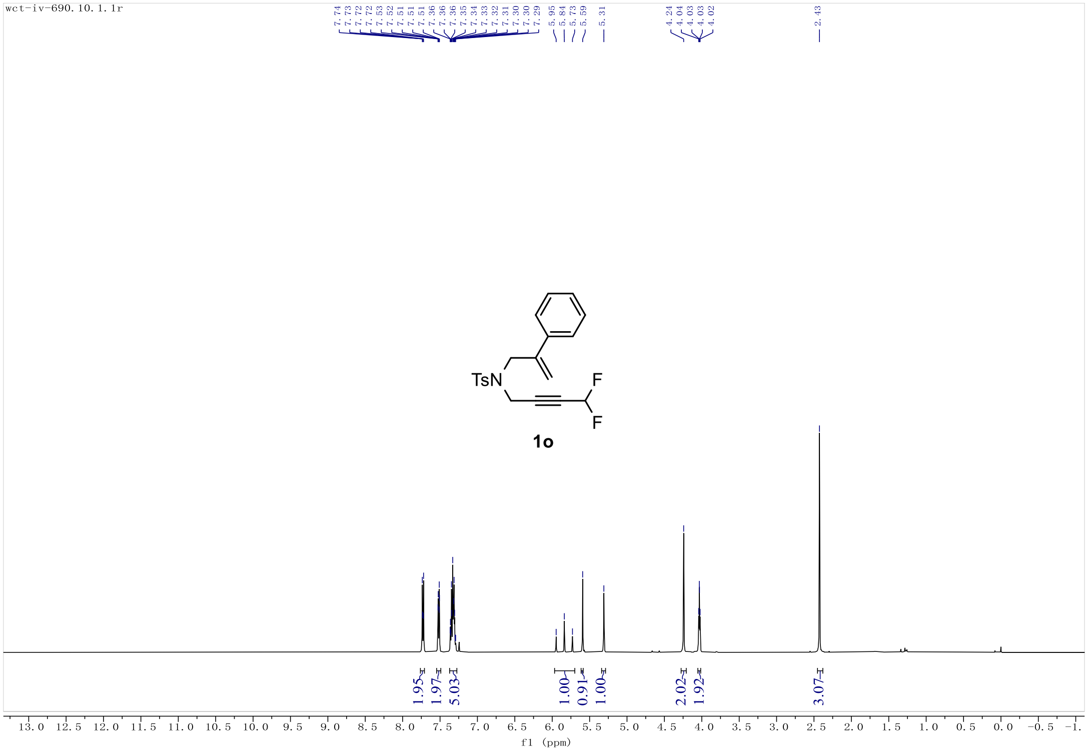
wct-iv-690.10.1.1r
7.74 7.73 7.72 7.72 7.53 7.52 7.51 7.51 7.51 7.51 7.36 7.36 7.36 7.35 7.34 7.33 7.32 7.31 7.30 7.29
5.95 5.84 5.73 5.59
5.31
4.24 4.04 4.03 4.03 4.02
2.43
TsN
F
F
1o
1.95 1.97 5.03 1.00 0.91 1.00 2.02 1.92 3.07
13.0 12.5 12.0 11.5 11.0 10.5 10.0 9.5 9.0 8.5 8.0 7.5 7.0 6.5 6.0 5.5 5.0 4.5 4.0 3.5 3.0 2.5 2.0 1.5 1.0 0.5 0.0 -0.5 -1.
f1 (ppm)

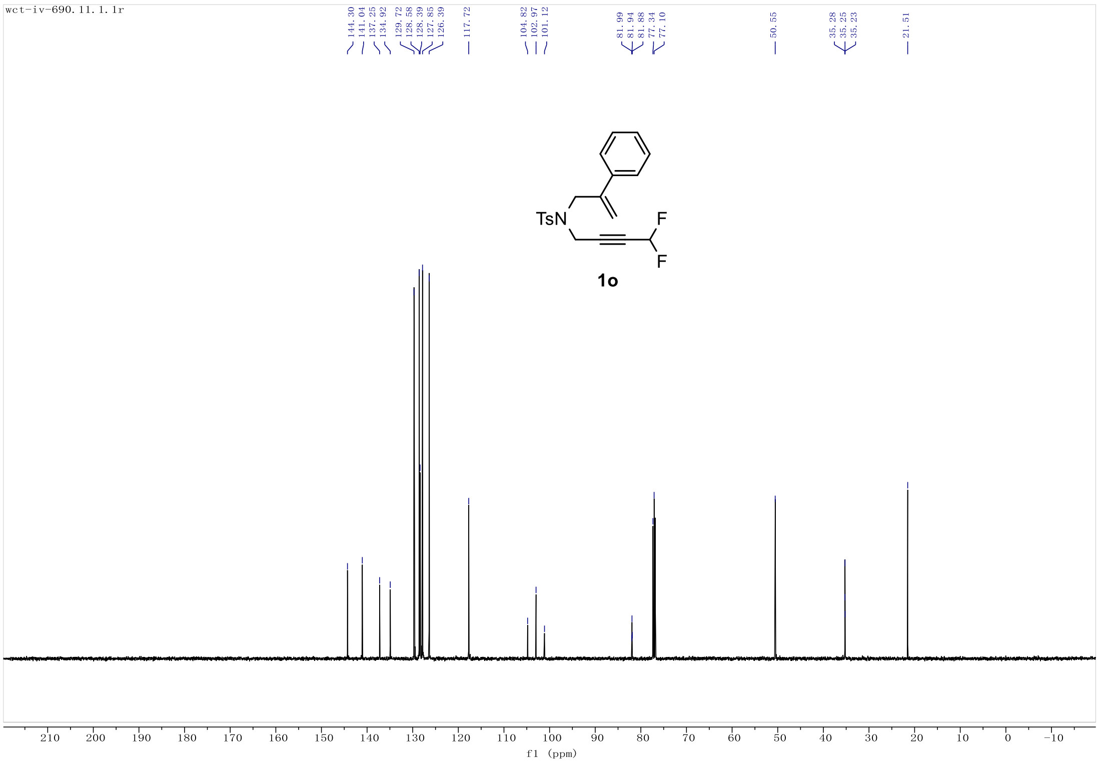
wct-iv-690.11.1.1r
144.30 141.04 137.25 134.92 129.72 128.58 128.39 128.04 127.85 126.39
117.72
104.82 102.97 101.12
81.99 81.94 81.88 77.34 77.10
50.55
35.28 35.25 35.23
21.51
TsN
F
F
1o
210 200 190 180 170 160 150 140 130 120 110 100 90 80 70 60 50 40 30 20 10 0 -10
f1 (ppm)

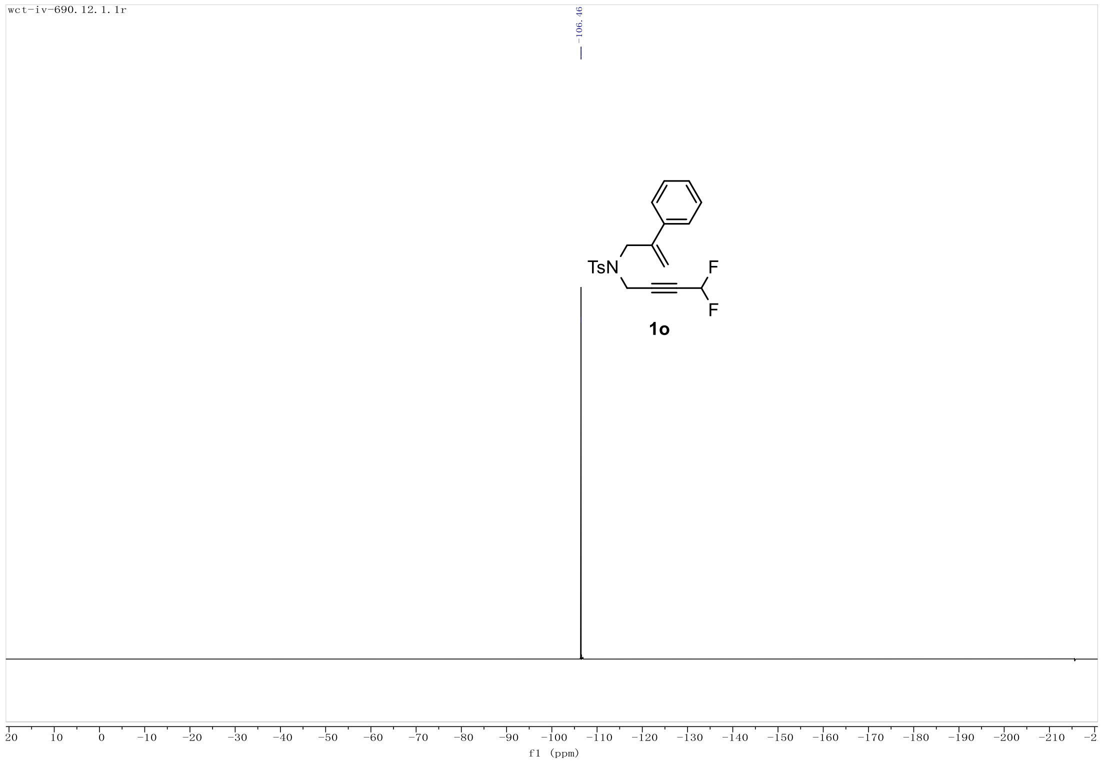
wct-iv-690.12.1.1r
-106.46
TsN
F
F
1o
f1 (ppm)

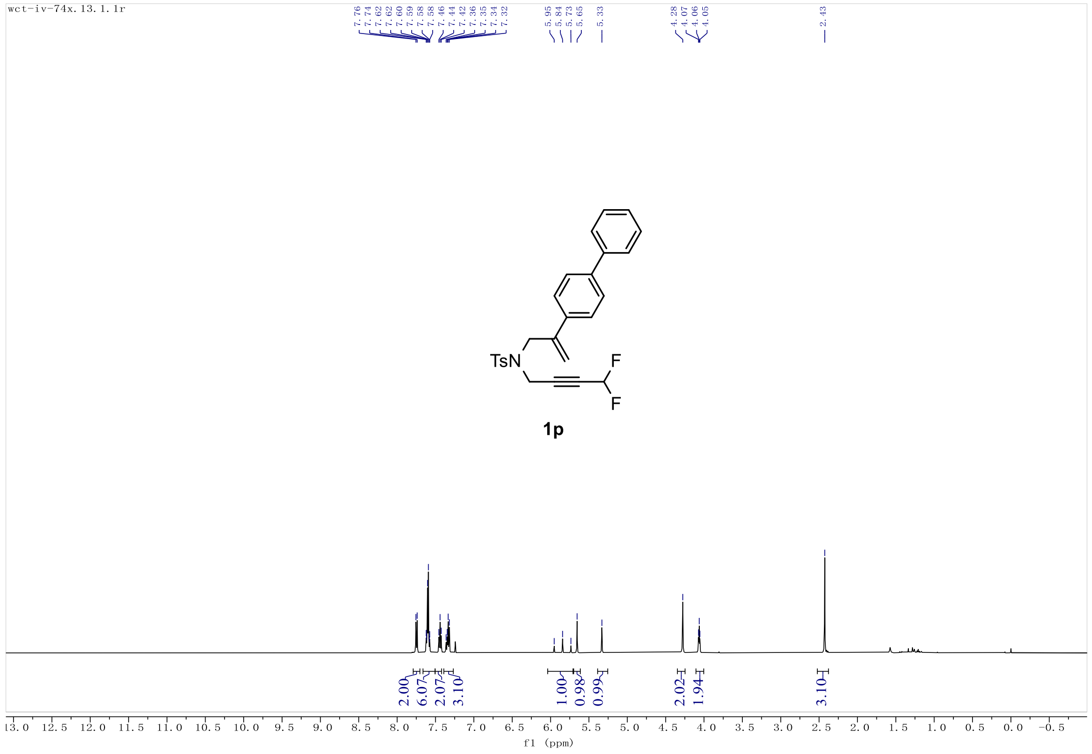
wct-iv-74x.13.1.1r
TsN
F
F
1p
f1 (ppm)

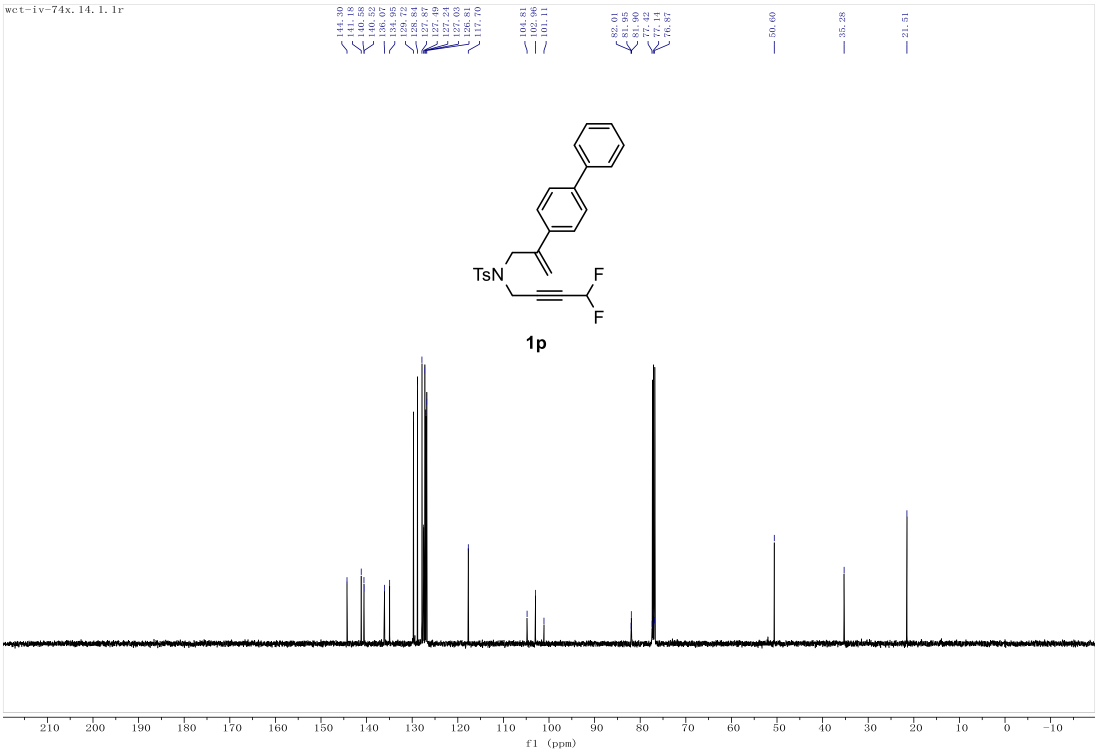
wct-iv-74x.14.1.1r
144.30
141.18
140.58
140.52
136.07
134.95
129.72
128.84
127.87
127.49
127.24
127.03
126.81
117.70
104.81
102.96
101.11
82.01
81.95
81.90
77.42
77.14
76.87
50.60
35.28
21.51
TsN
F
F
1p
f1 (ppm)

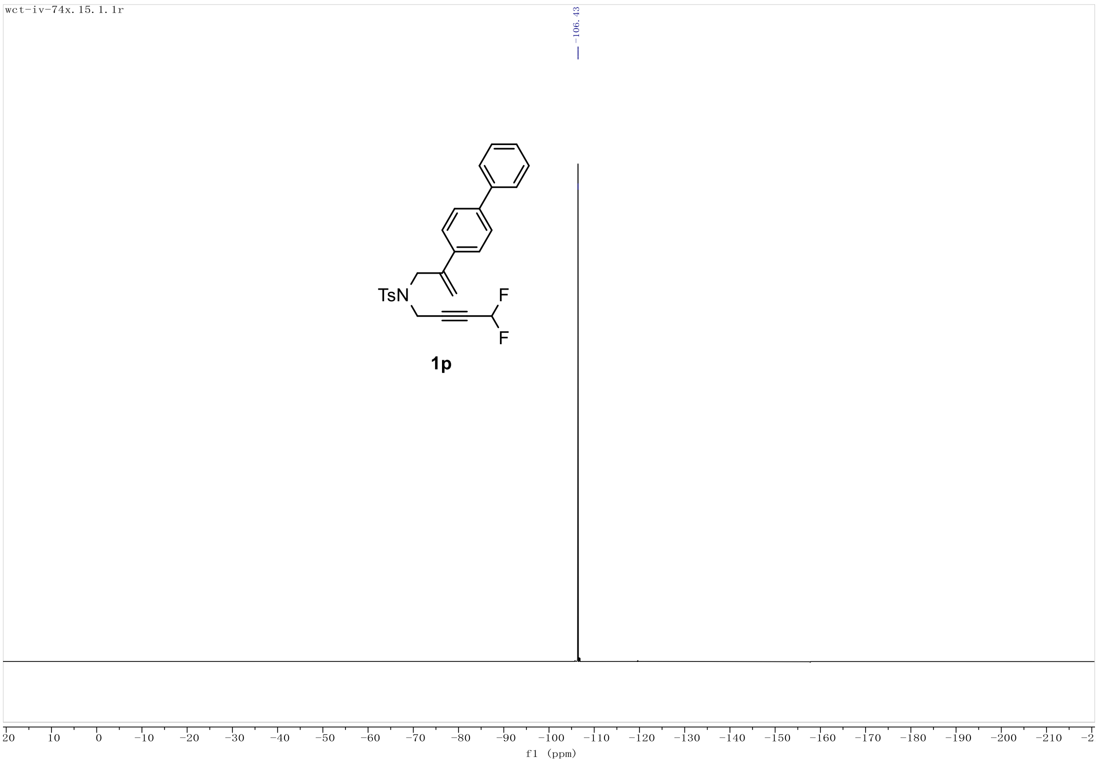
wct-iv-74x.15.1.1r
-106.43
TsN
F
F
1p
f1 (ppm)

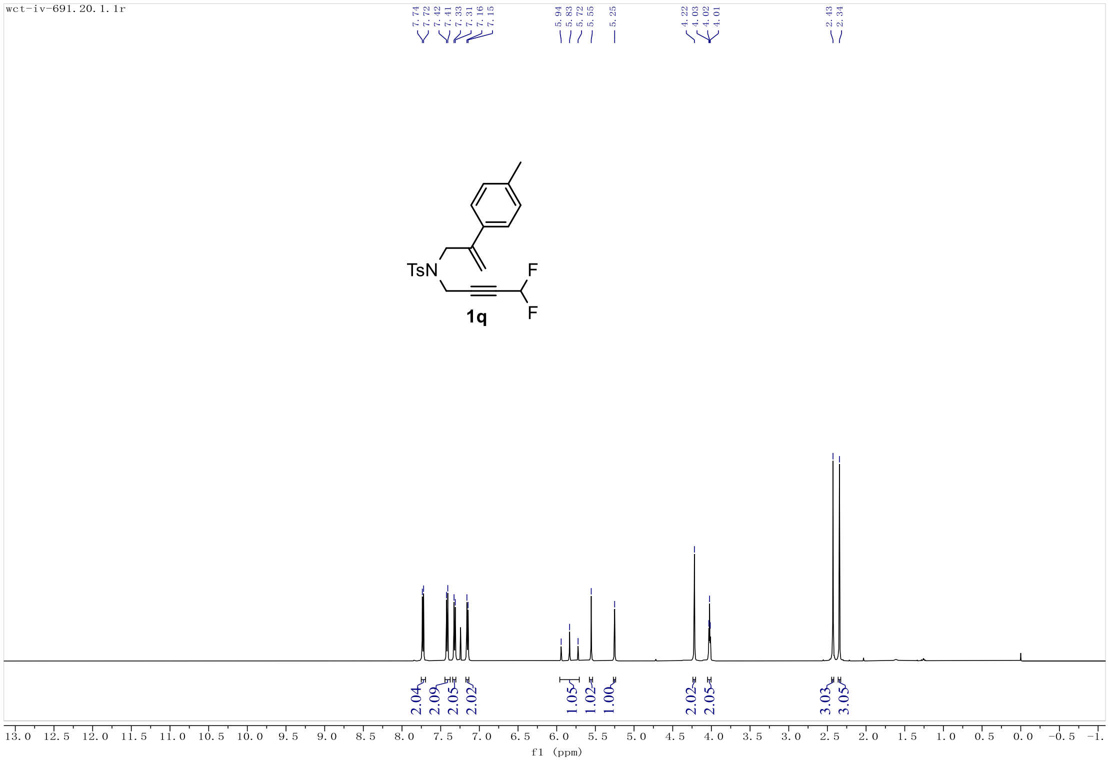
wct-iv-691.20.1.1r
7.74
7.72
7.42
7.41
7.33
7.31
7.16
7.15
5.94
5.83
5.72
5.55
5.25
4.22
4.03
4.02
4.01
2.43
2.34
TsN
F
F
1q
2.04
2.09
2.05
2.02
1.05
1.02
1.00
2.02
2.05
3.03
3.05
f1 (ppm)

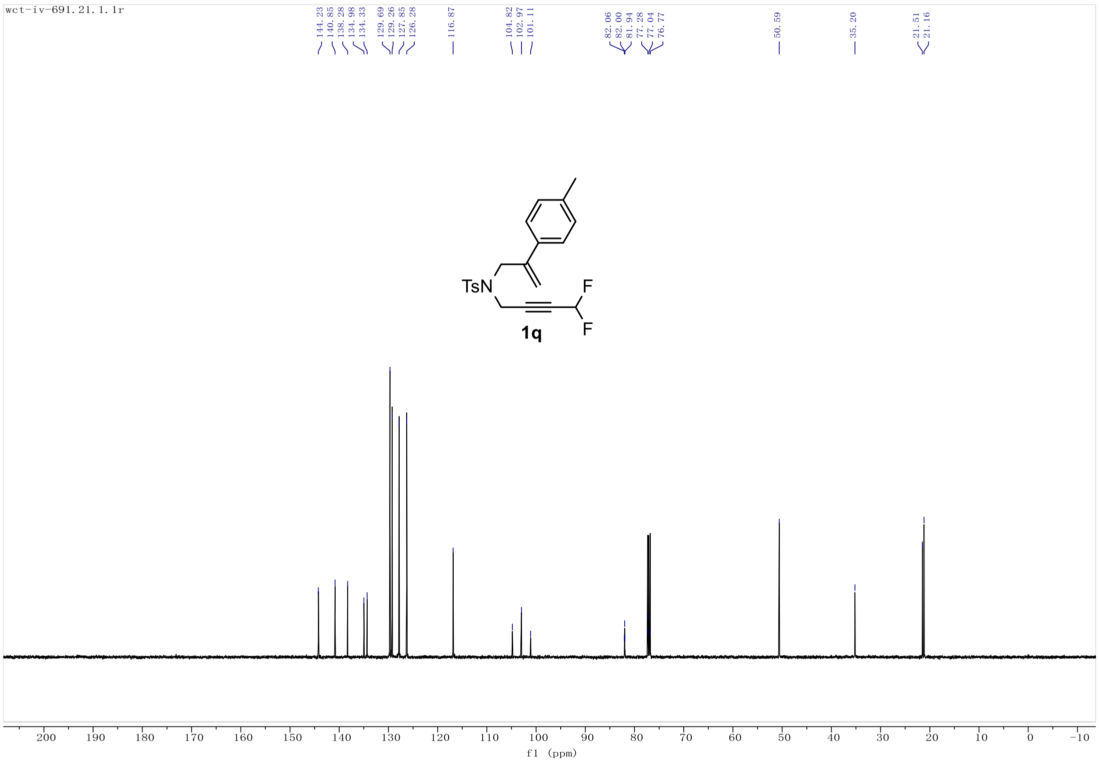
wct-iv-691.21.1.1r
144.23
140.85
138.28
134.98
134.33
129.69
129.26
127.85
126.28
116.87
104.82
102.97
101.11
82.06
82.00
81.94
77.28
77.04
76.77
50.59
35.20
21.51
21.16
TsN
F
F
1q
f1 (ppm)

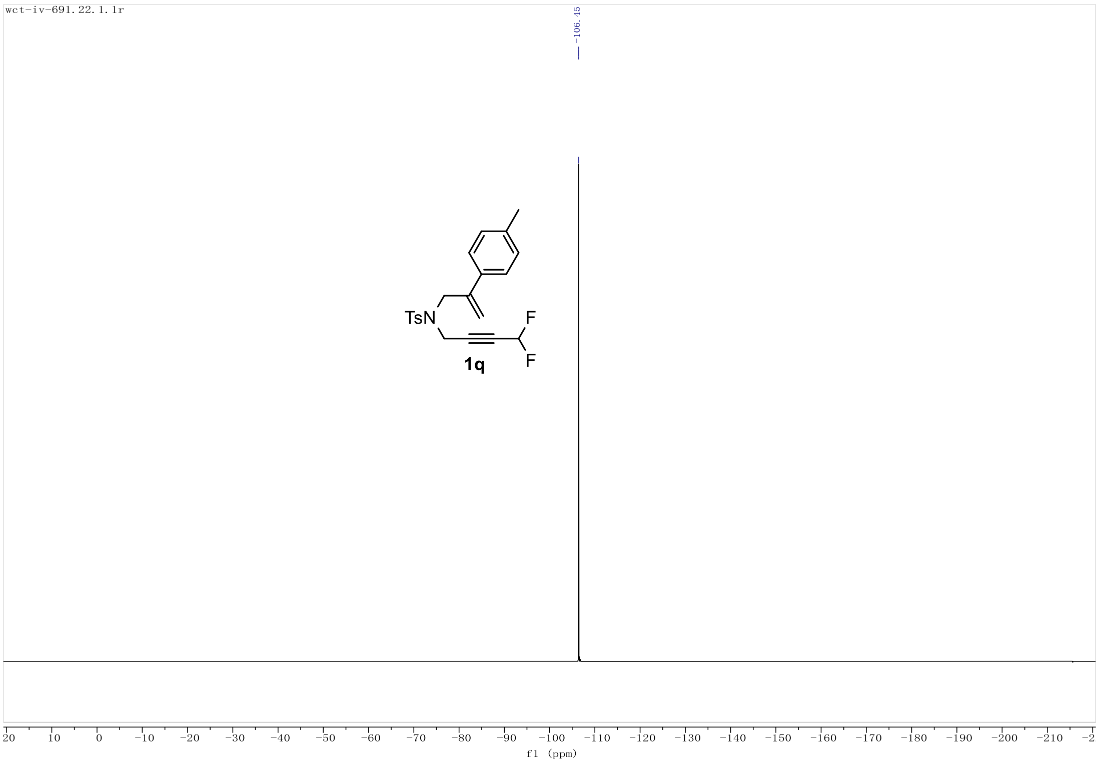
wct-iv-691.22.1.1r
-106.45
TsN
F
F
1q
f1 (ppm)

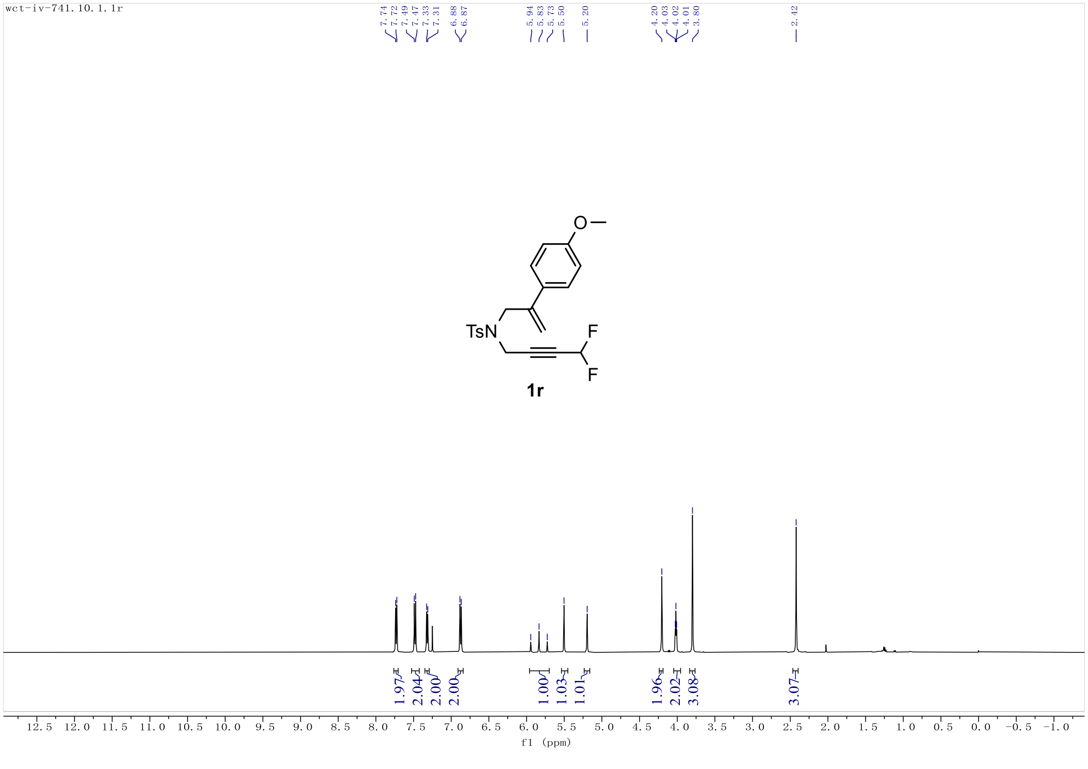
wct-iv-741.10.1.1r
7.74
7.72
7.49
7.47
7.33
7.31
6.88
6.87
5.94
5.83
5.73
5.50
5.20
4.20
4.03
4.02
4.01
3.80
2.42
O
TsN
F
F
1r
1.97
2.04
2.00
2.00
1.00
1.03
1.01
1.96
2.02
3.08
3.07
f1 (ppm)

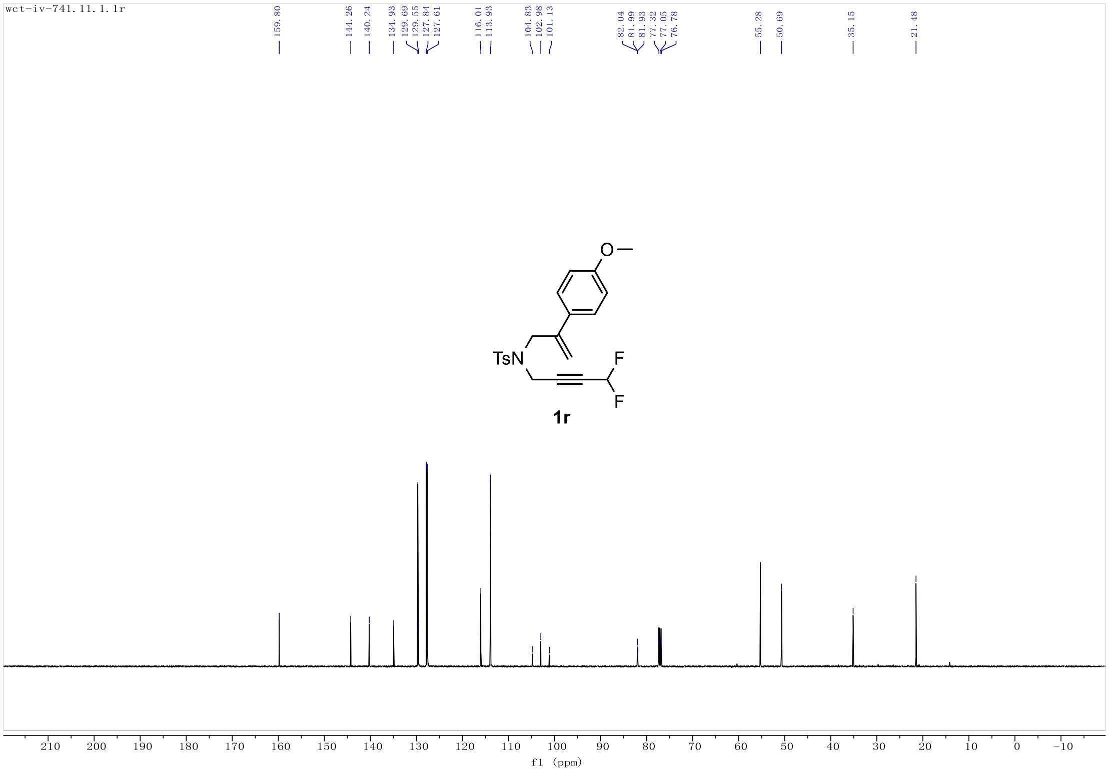
wct-iv-741.11.1.1r
159.80
144.26
140.24
134.93
129.69
129.55
127.84
127.61
116.01
113.93
104.83
102.98
101.13
82.04
81.99
81.93
77.32
77.05
76.78
55.28
50.69
35.15
21.48
TsN
F
F
1r
f1 (ppm)

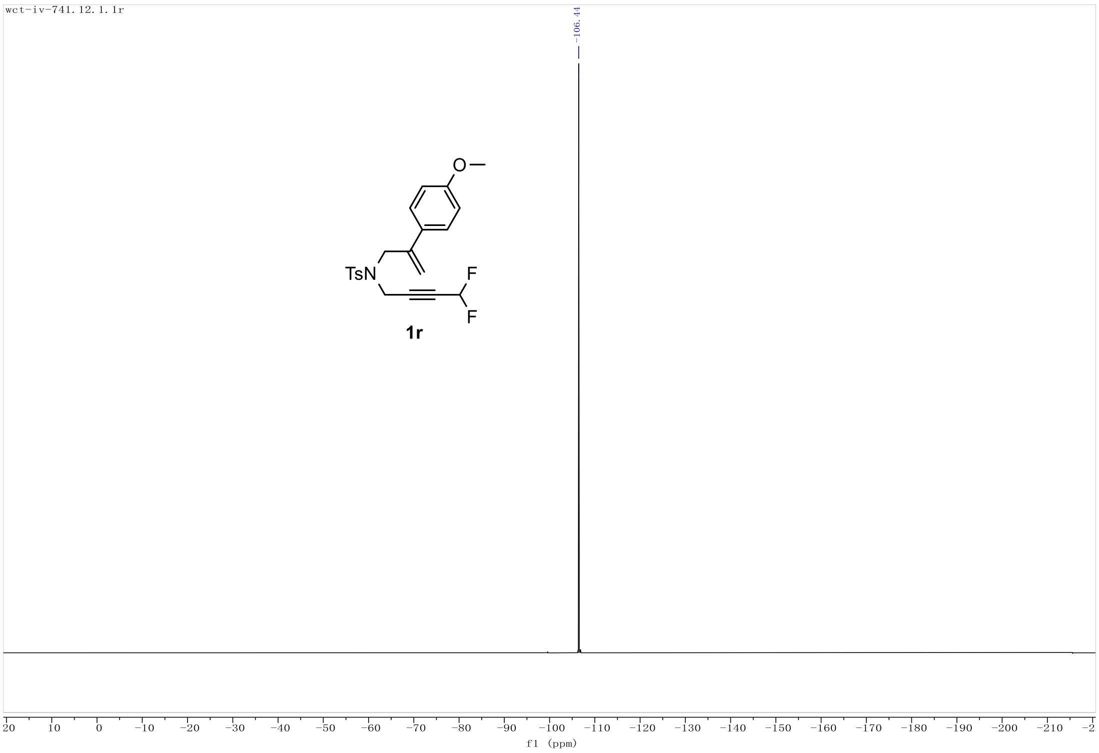
wct-iv-741.12.1.1r
-106.44
TsN
F
F
1r
f1 (ppm)

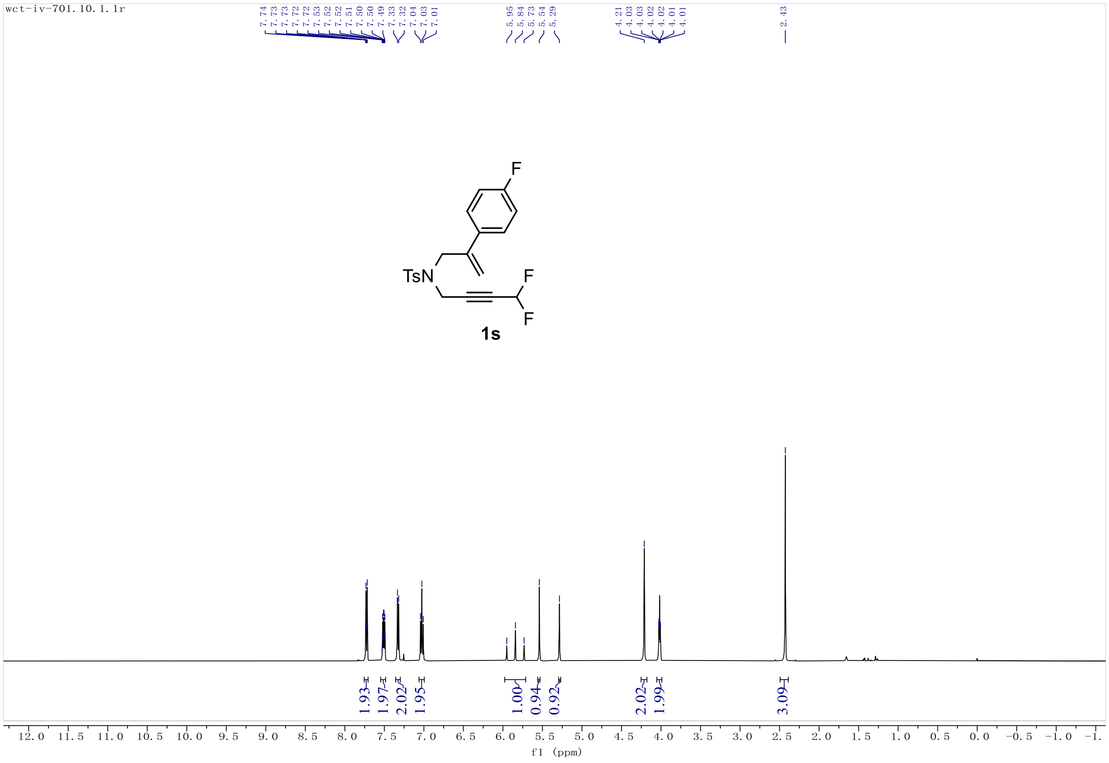
wct-iv-701.10.1.1r
TsN
1s
f1 (ppm)

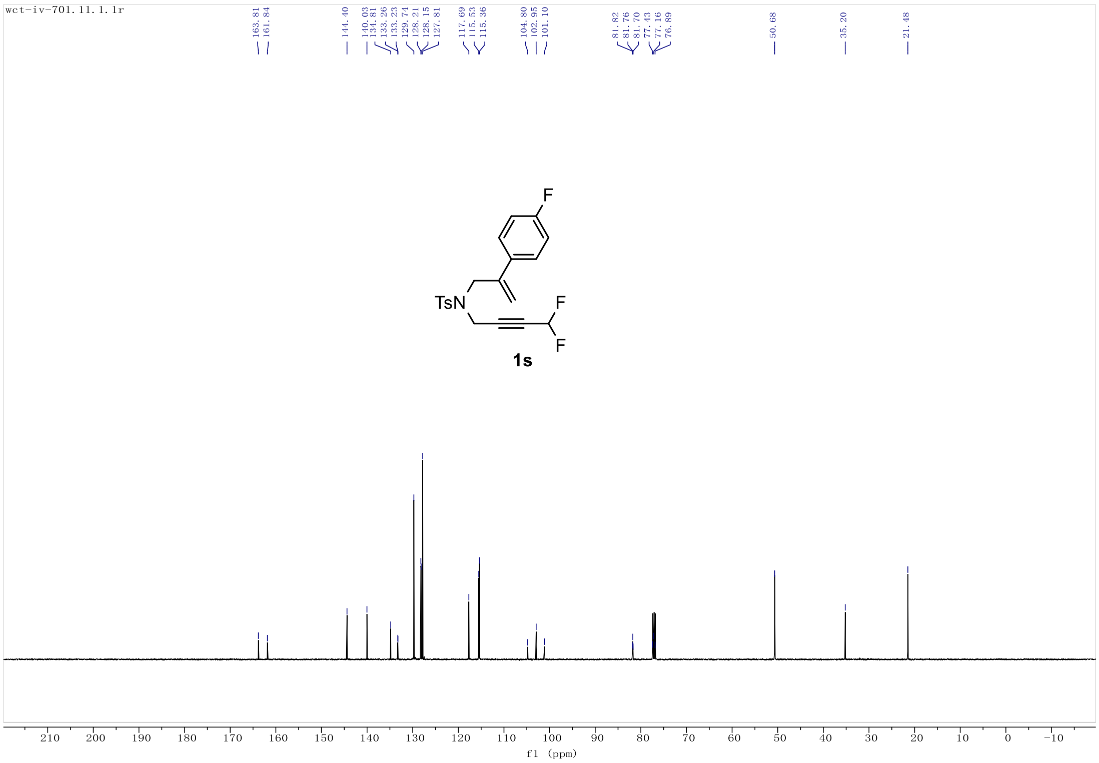
wct-iv-701.11.1.1r
TsN
1s
f1 (ppm)

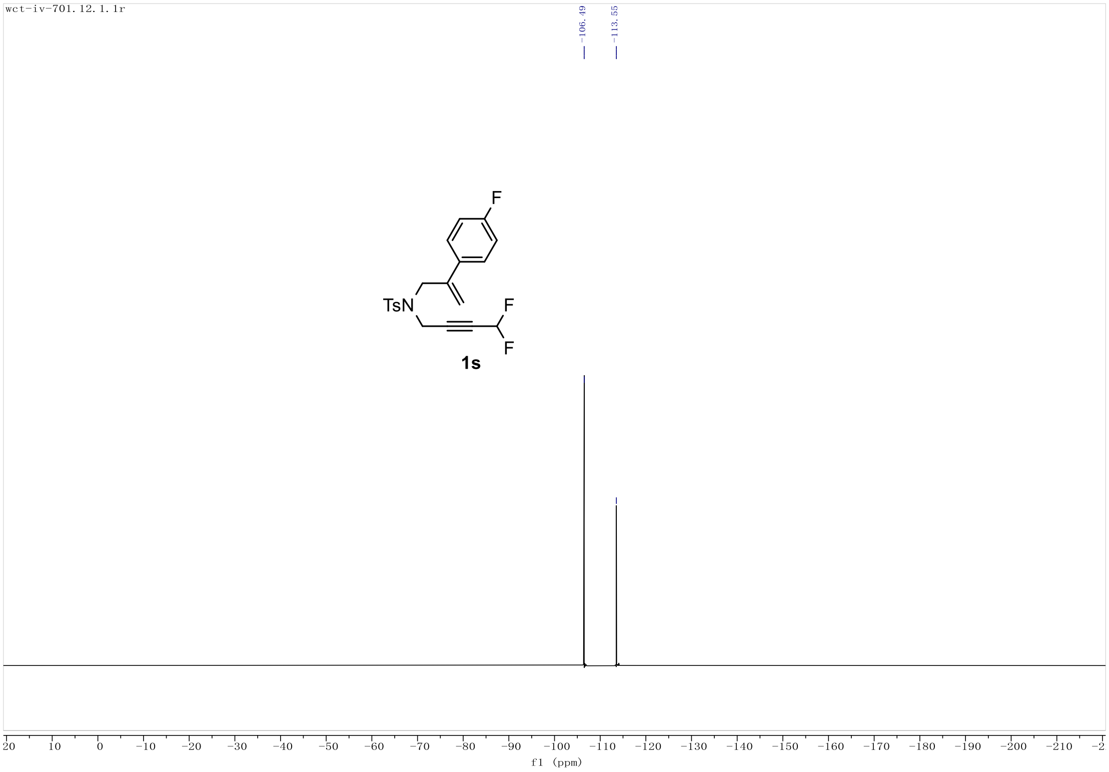
wct-iv-701.12.1.1r
-106.49
-113.55
F
TsN
F
F
1s
f1 (ppm)

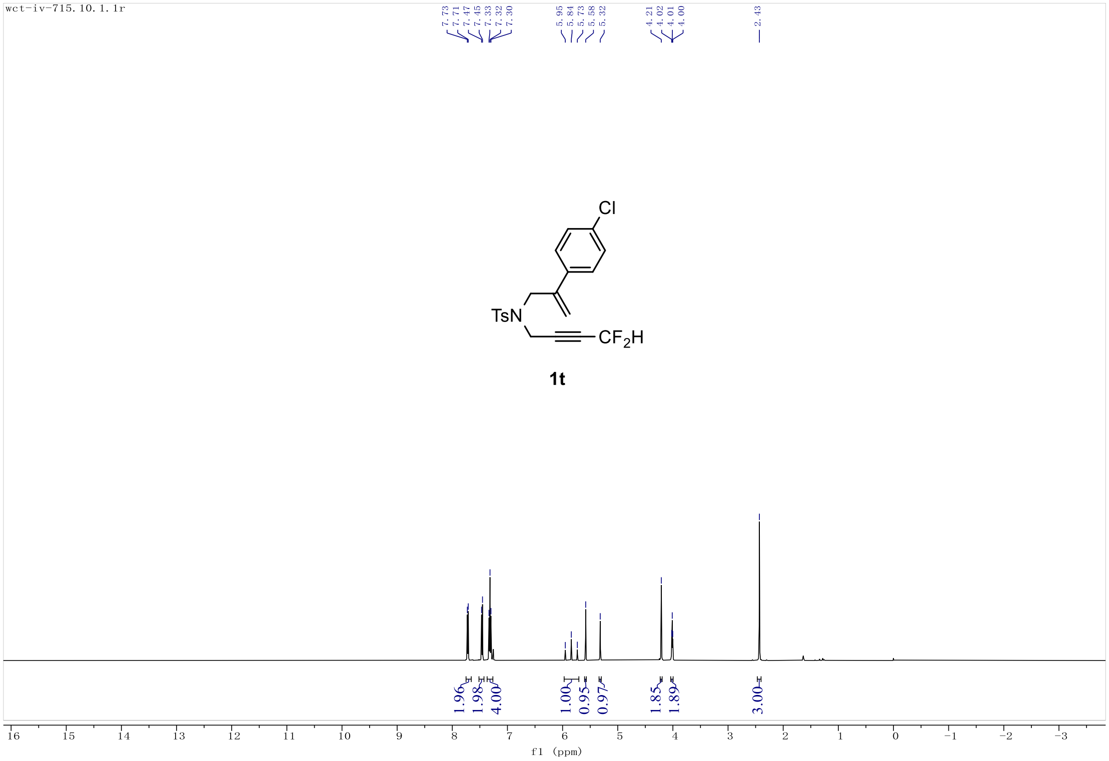
wct-iv-715.10.1.1r
7.73
7.71
7.47
7.45
7.33
7.32
7.30
5.95
5.84
5.73
5.58
5.32
4.21
4.02
4.01
4.00
2.43
Cl
TsN
CF2H
1t
1.96
1.98
4.00
1.00
0.95
0.97
1.85
1.89
3.00
f1 (ppm)

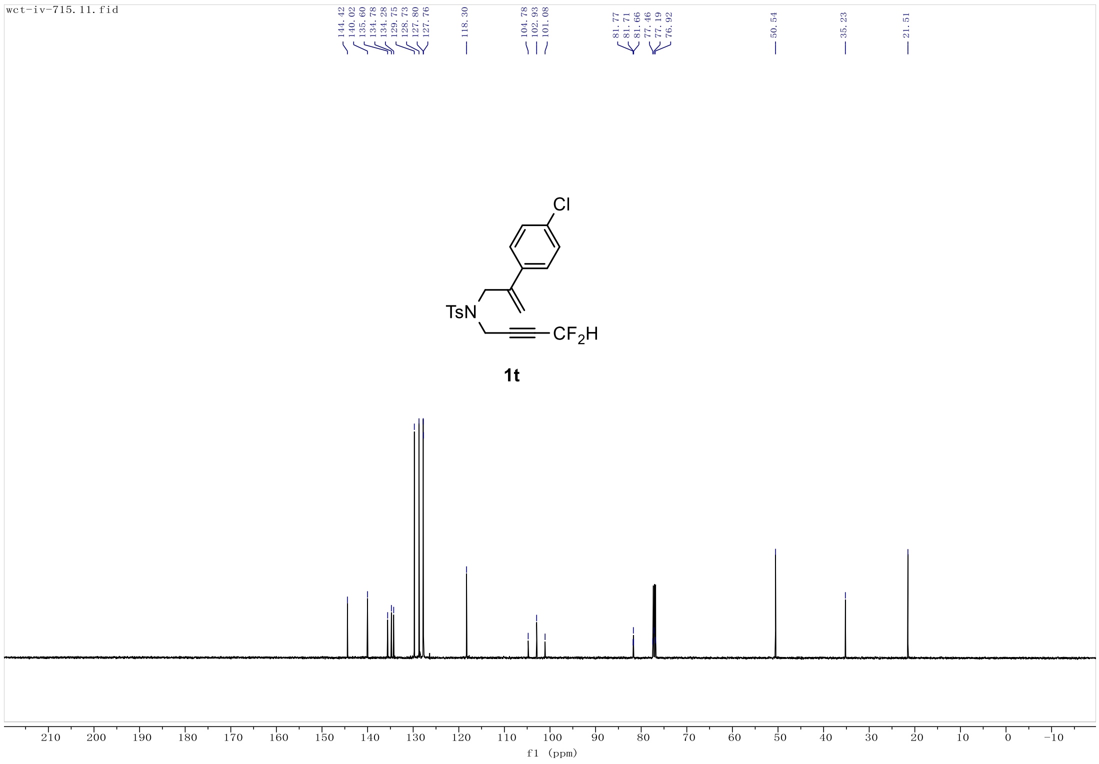
wct-iv-715.11.fid
144.42
140.02
135.60
134.78
134.28
129.75
128.73
127.80
127.76
118.30
104.78
102.93
101.08
81.77
81.71
81.66
77.46
77.19
76.92
50.54
35.23
21.51
Cl
TsN
CF2H
1t
f1 (ppm)

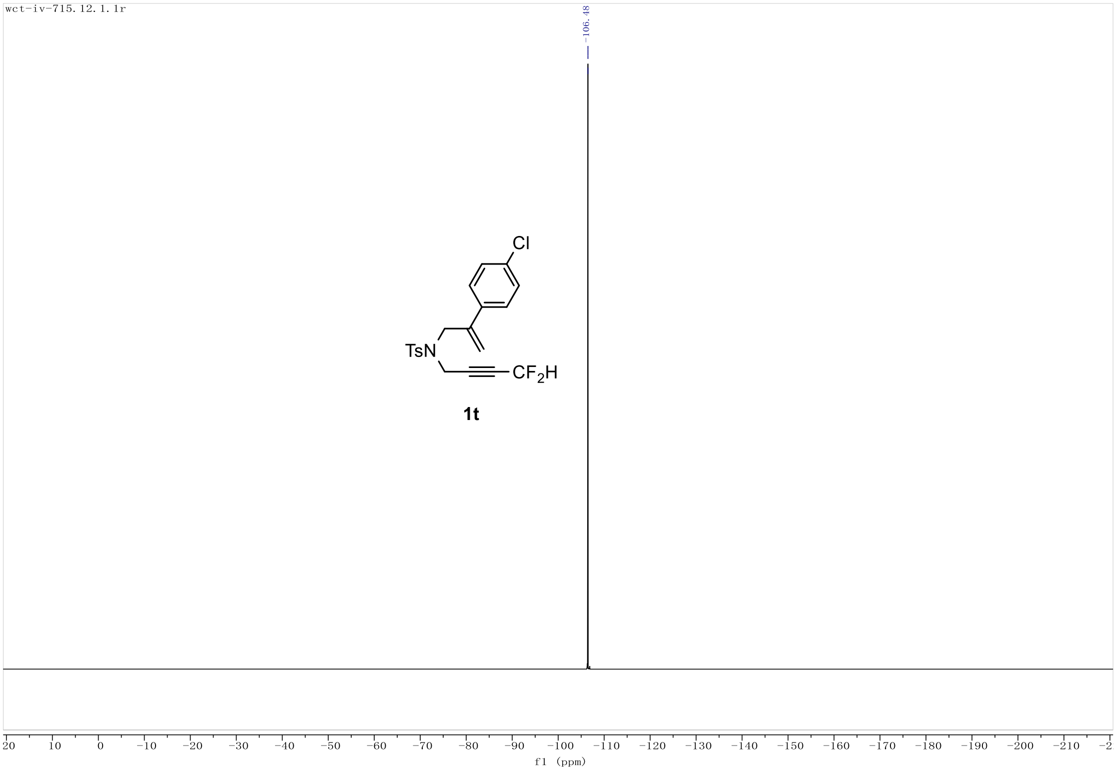
wct-iv-715.12.1.1r
-106.48
Cl
TsN
CF2H
1t
f1 (ppm)

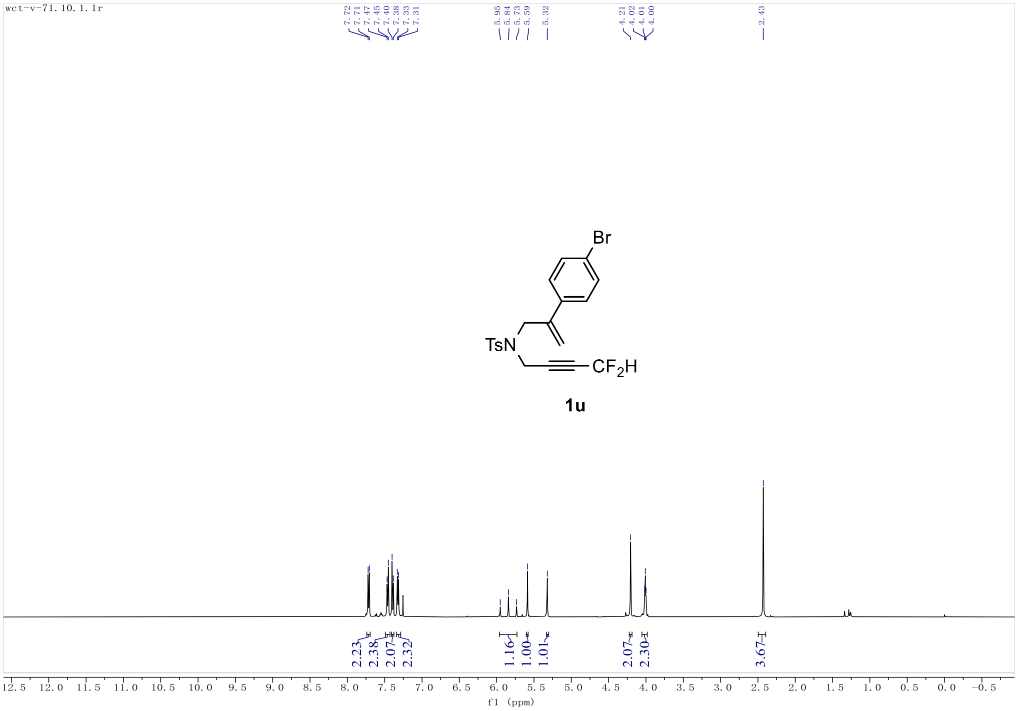
wct-v-71.10.1.1r
7.72 7.71 7.47 7.45 7.40 7.38 7.33 7.31
5.95 5.84 5.73 5.59
5.32
4.21 4.02 4.01 4.00
2.43
Br
TsN
CF2H
1u
2.23 2.38 2.07 2.32
1.16 1.00 1.01
2.07 2.30
3.67
f1 (ppm)

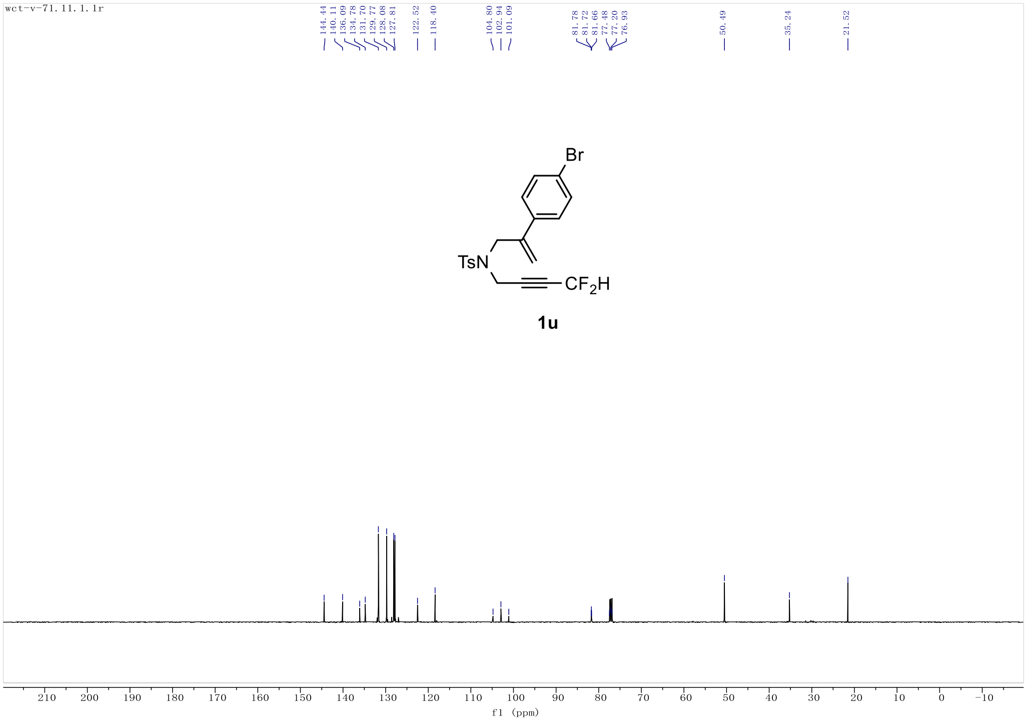
wct-v-71.11.1.1r
144.44 140.11 136.09 134.78 131.70 129.77 128.08 127.81
122.52
118.40
104.80 102.94 101.09
81.78 81.72 81.66 77.48 77.20 76.93
50.49
35.24
21.52
Br
TsN
CF2H
1u
f1 (ppm)

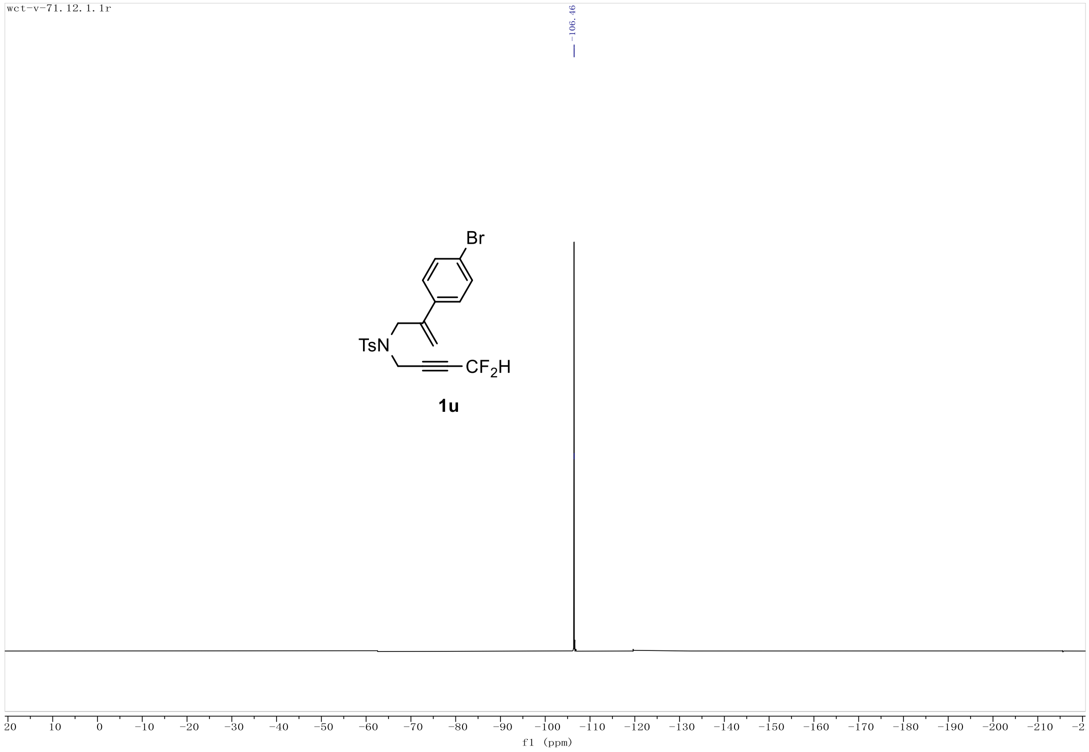
wct-v-71.12.1.1r
−106.46
Br
TsN
CF2H
1u
f1 (ppm)

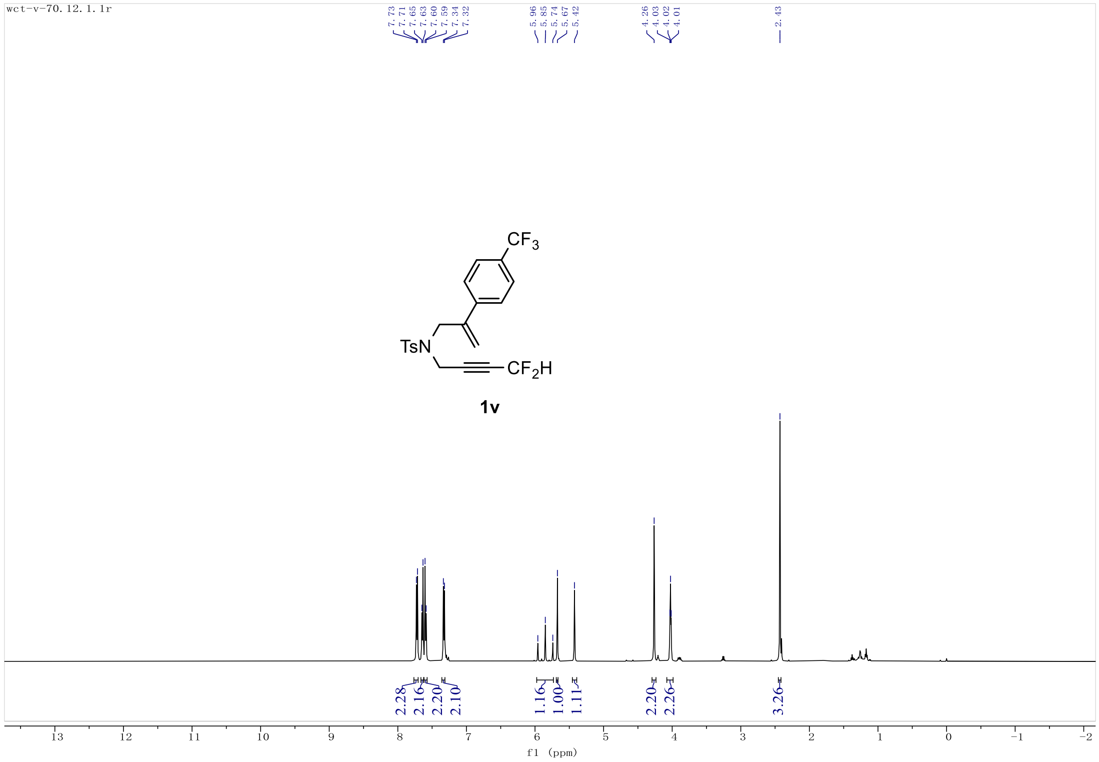
wct-v-70.12.1.1r
7.73
7.71
7.65
7.63
7.60
7.59
7.34
7.32
5.96
5.85
5.74
5.67
5.42
4.26
4.03
4.02
4.01
2.43
CF3
TsN
CF2H
1v
2.28
2.16
2.20
2.10
1.16
1.00
1.11
2.20
2.26
3.26
f1 (ppm)

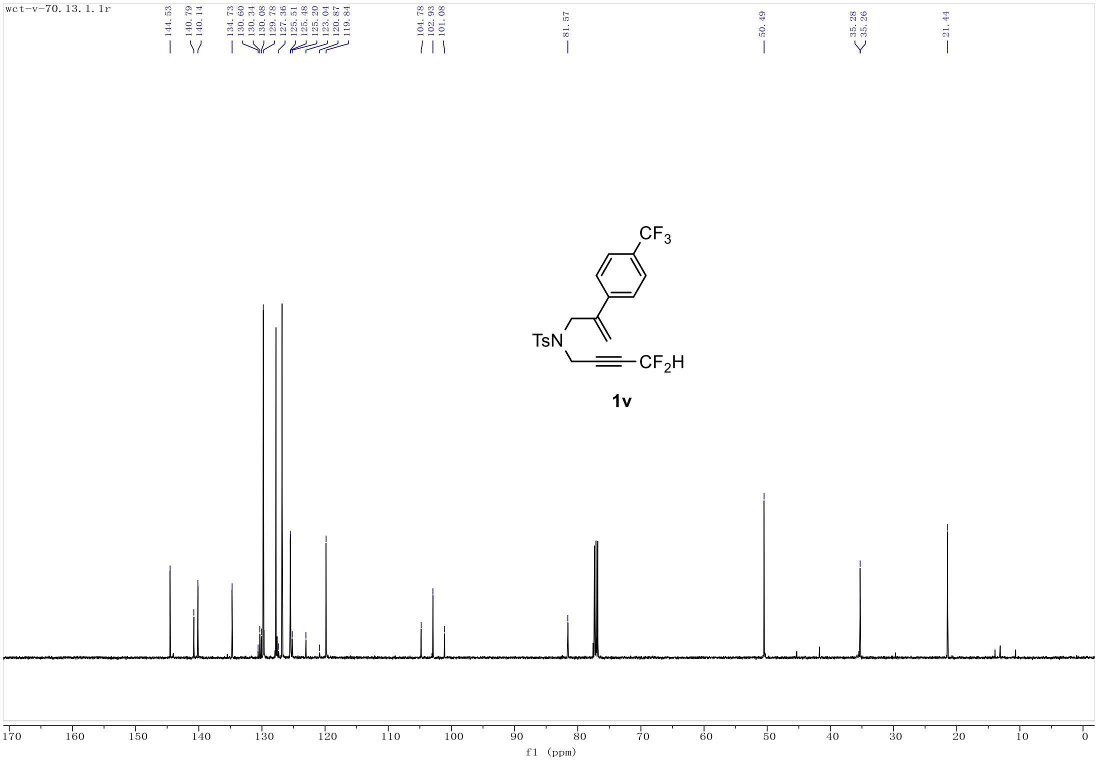
wct-v-70.13.1.1r
144.53
140.79
140.14
134.73
130.60
130.34
130.08
129.78
127.36
125.51
125.48
125.20
123.04
120.87
119.84
104.78
102.93
101.08
81.57
50.49
35.28
35.26
21.44
CF3
TsN
CF2H
1v
f1 (ppm)

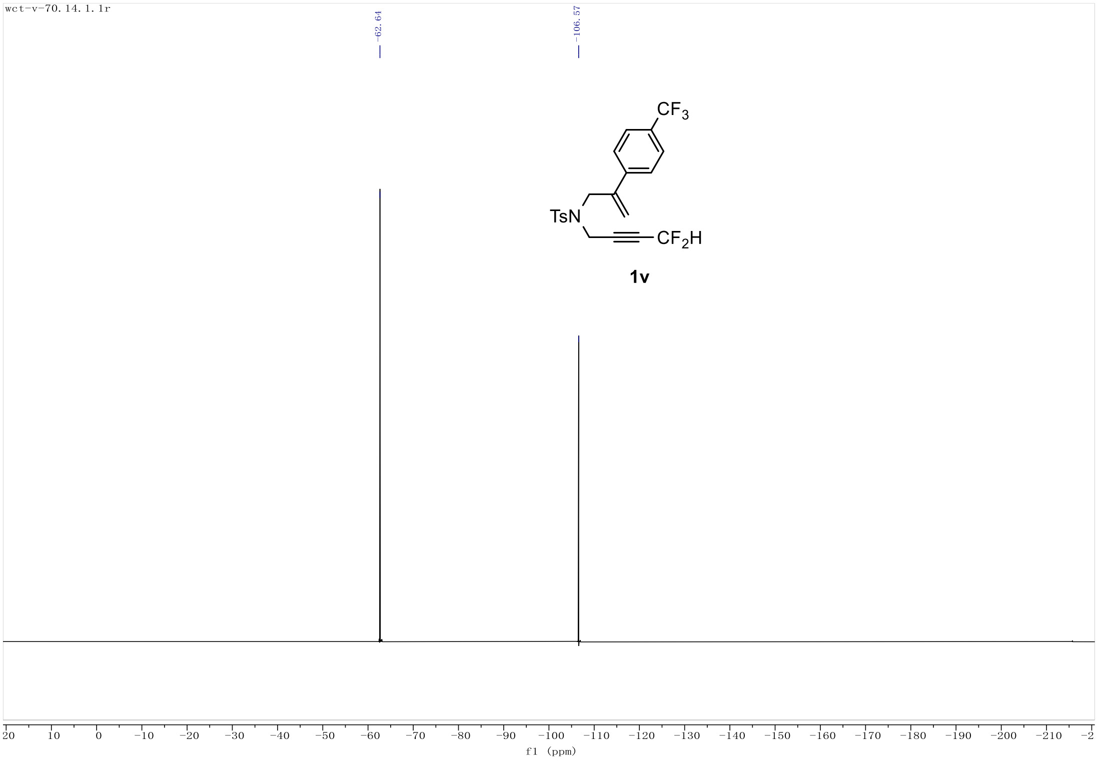
wct-v-70.14.1.1r
-62.64
-106.57
CF3
TsN
CF2H
1v
f1 (ppm)

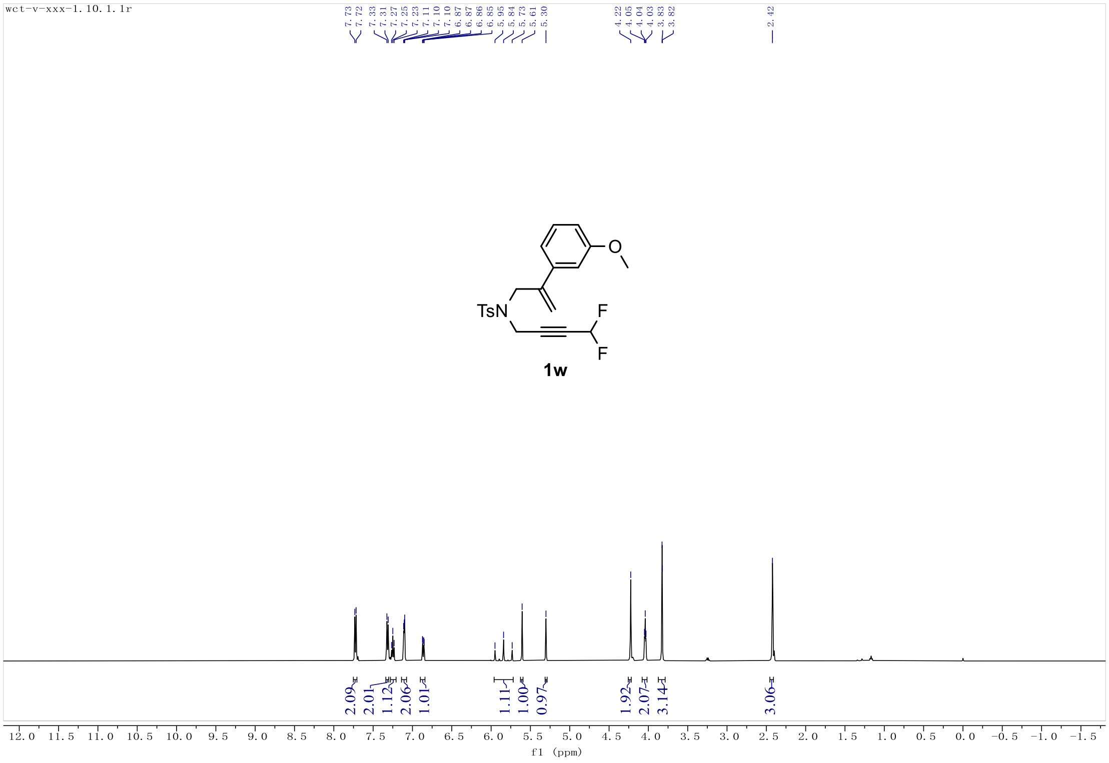
wct-v-xxx-1.10.1.1r
TsN
F
F
1w
f1 (ppm)

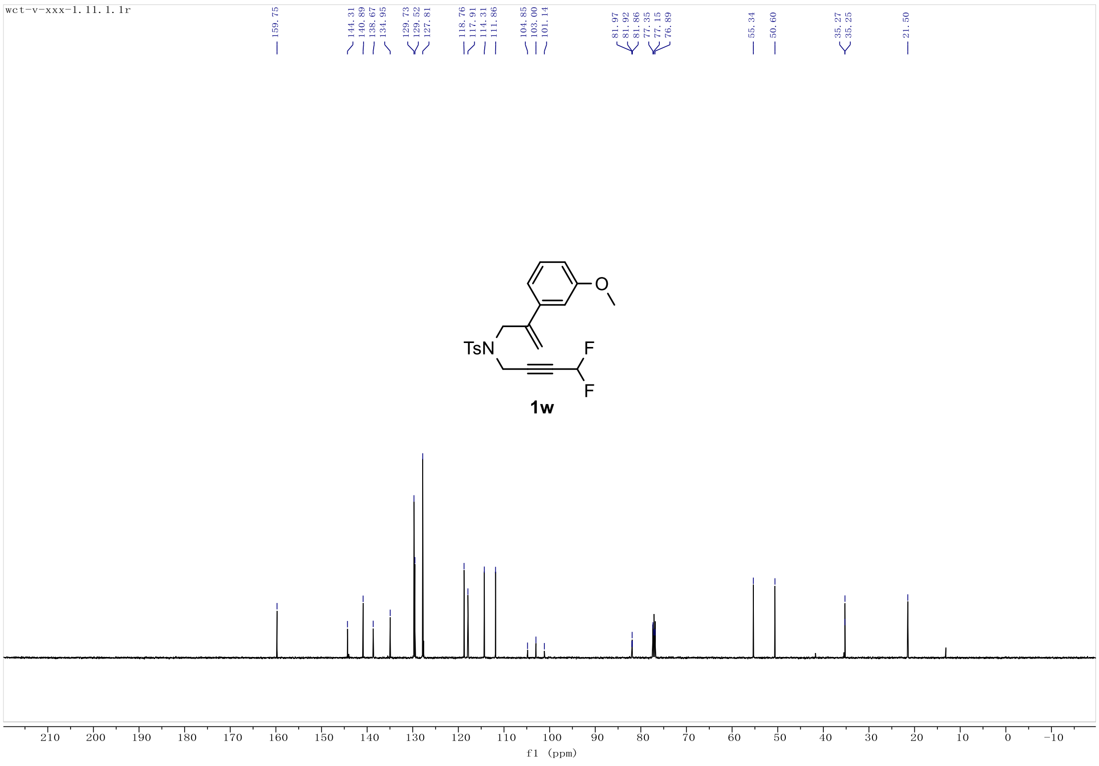
wct-v-xxx-1.11.1.1r
TsN
F
F
1w
f1 (ppm)

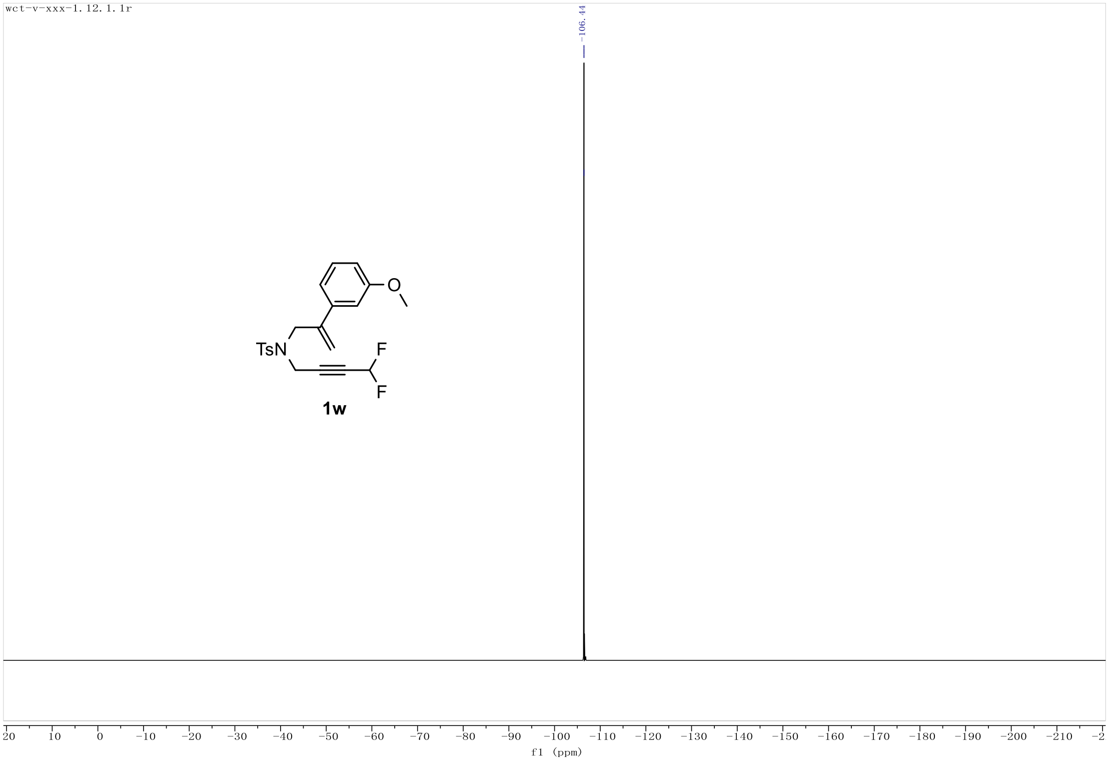
wct-v-xxx-1.12.1.1r
-106.44
TsN
O
F
F
1w
f1 (ppm)

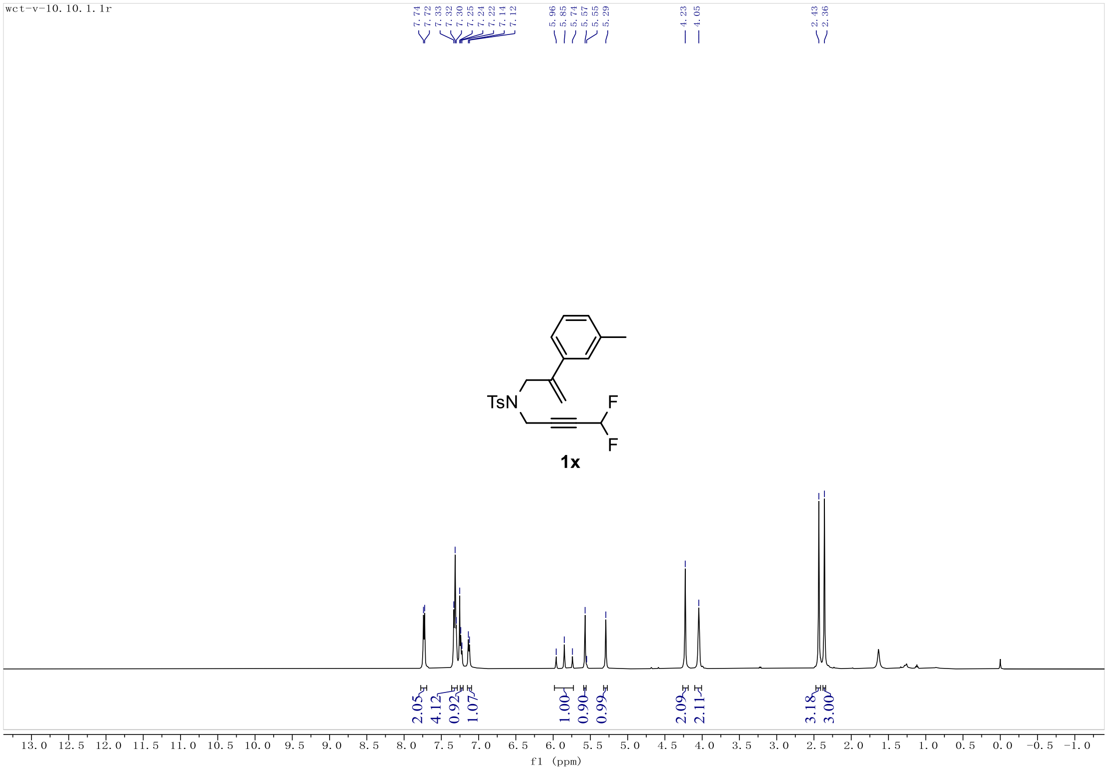
wct-v-10.10.1.1r
7.74
7.72
7.33
7.32
7.30
7.25
7.24
7.22
7.14
7.12
5.96
5.85
5.74
5.57
5.55
5.29
4.23
4.05
2.43
2.36
TsN
F
F
1x
2.05
4.12
0.92
1.07
1.00
0.90
0.99
2.09
2.11
3.18
3.00
f1 (ppm)

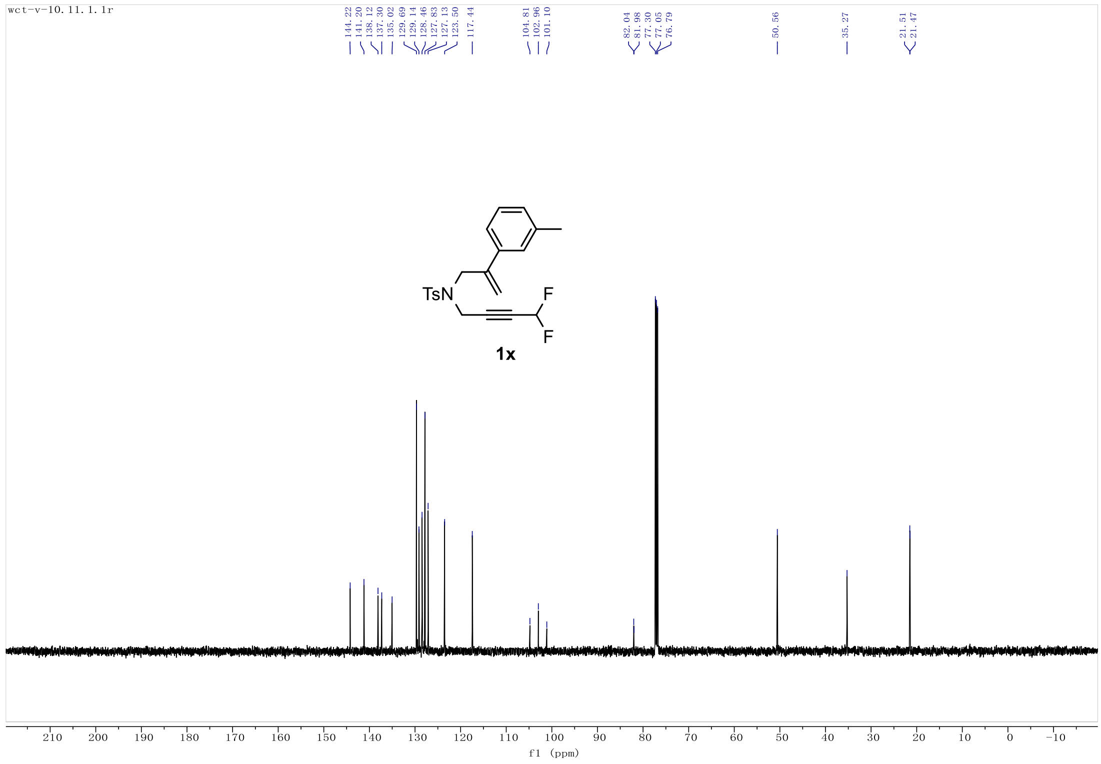
wct-v-10.11.1.1r
144.22
141.20
138.12
137.30
135.02
129.69
129.14
128.46
127.83
127.13
123.50
117.44
104.81
102.96
101.10
82.04
81.98
77.30
77.05
76.79
50.56
35.27
21.51
21.47
TsN
F
F
1x
f1 (ppm)

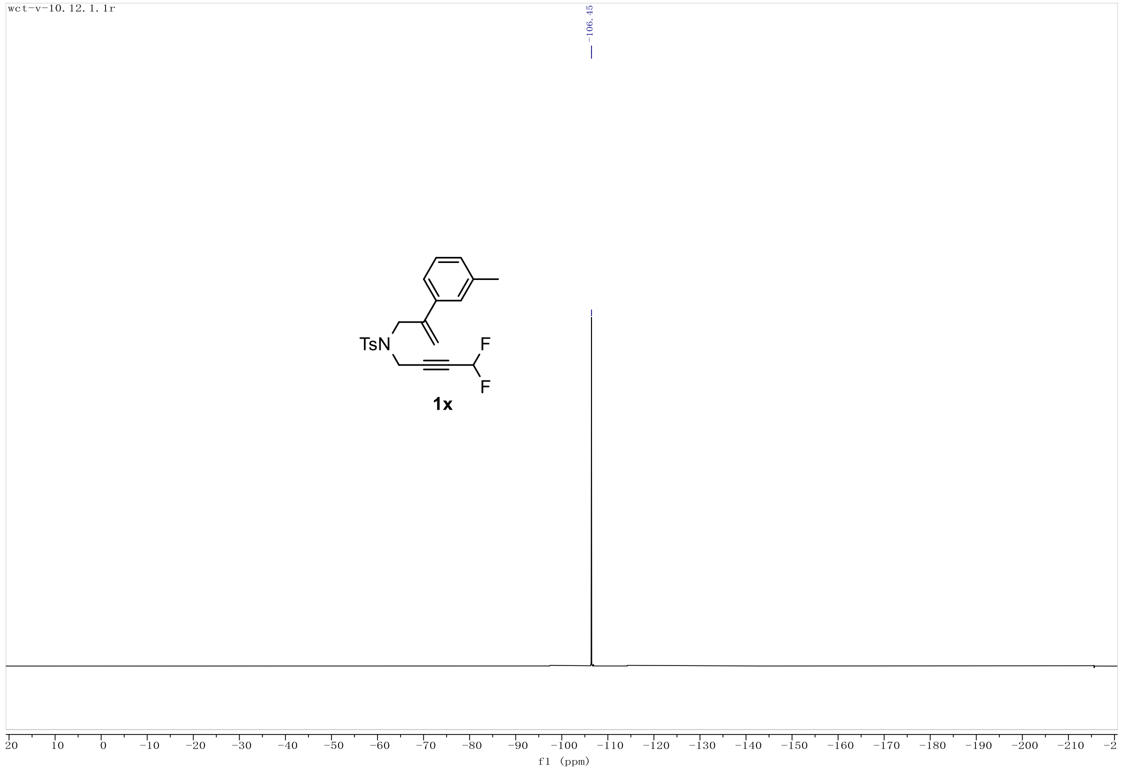
wct-v-10.12.1.1r
-106.45
TsN
F
F
1x
f1 (ppm)

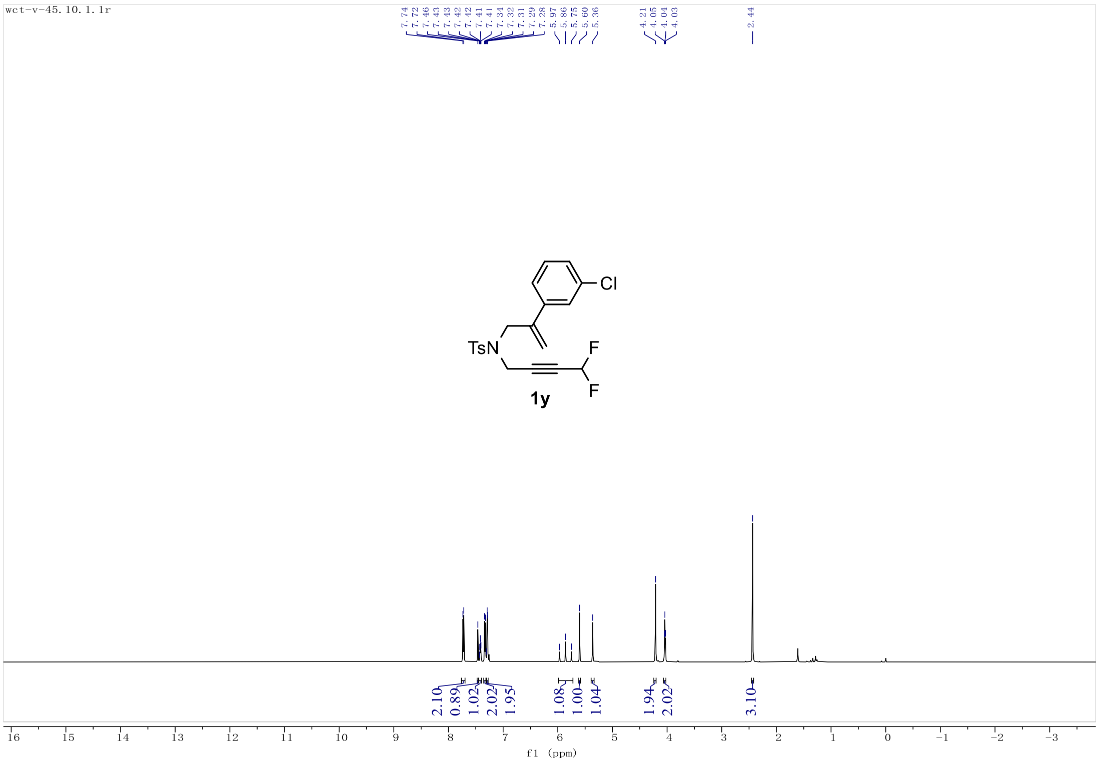

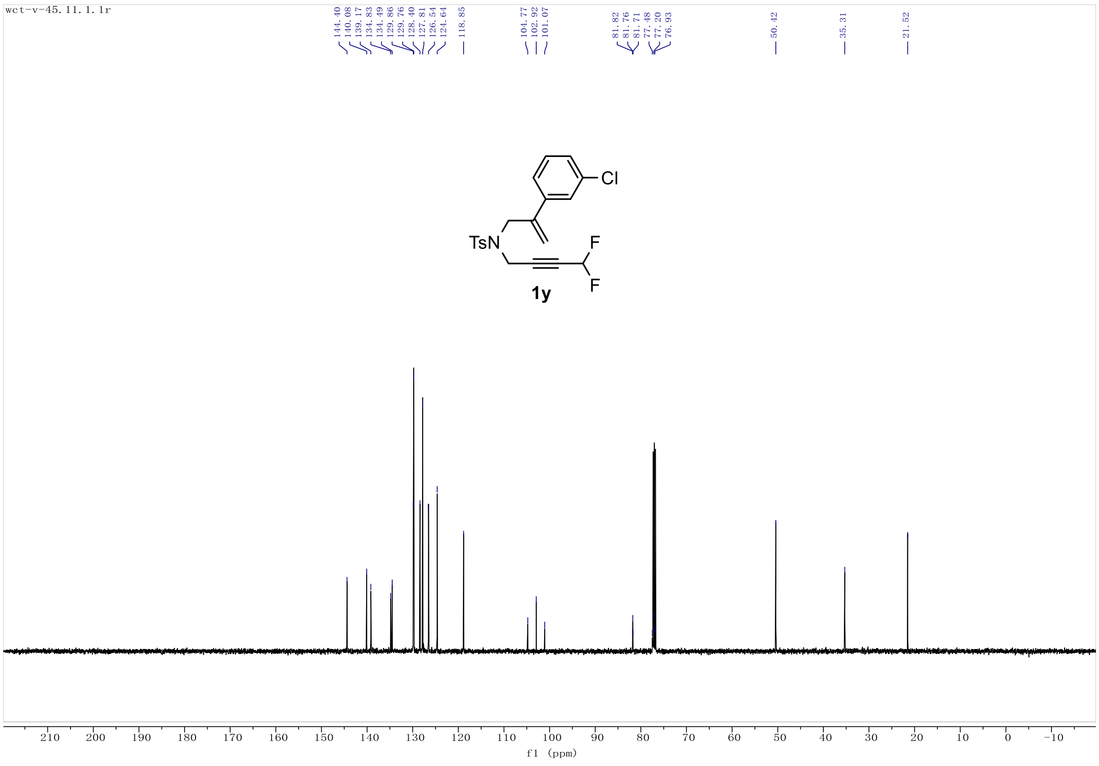

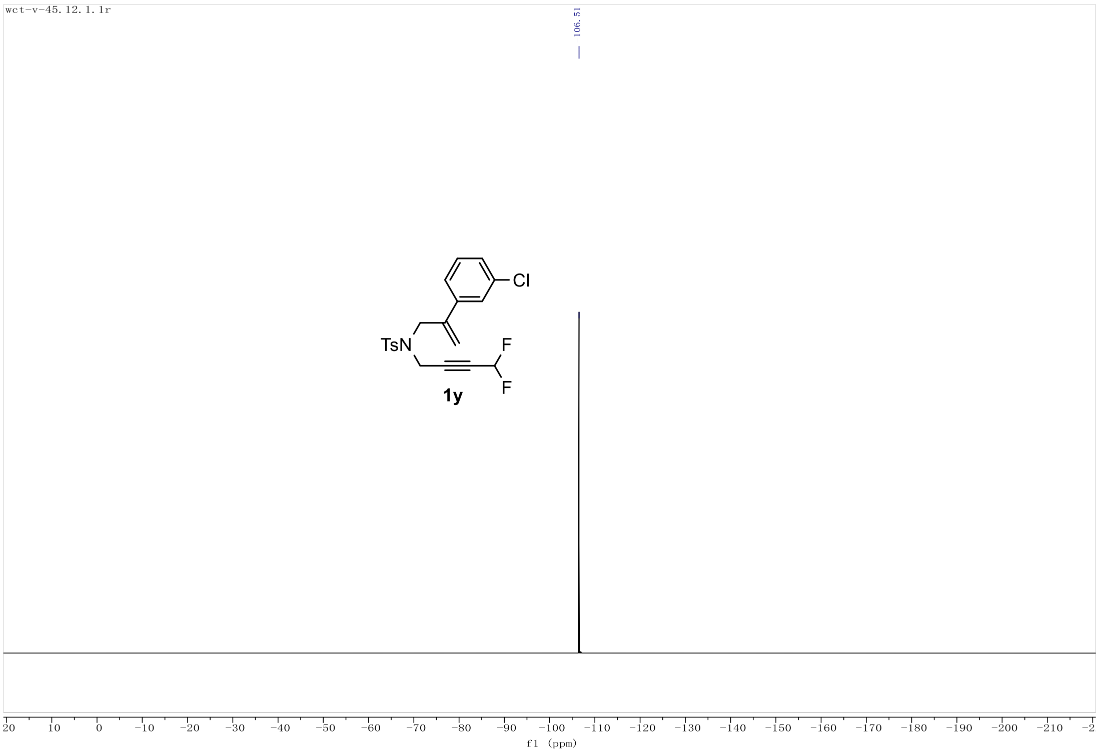
wct-v-45.12.1.1r
-106.51
TsN
Cl
F
F
1y
f1 (ppm)

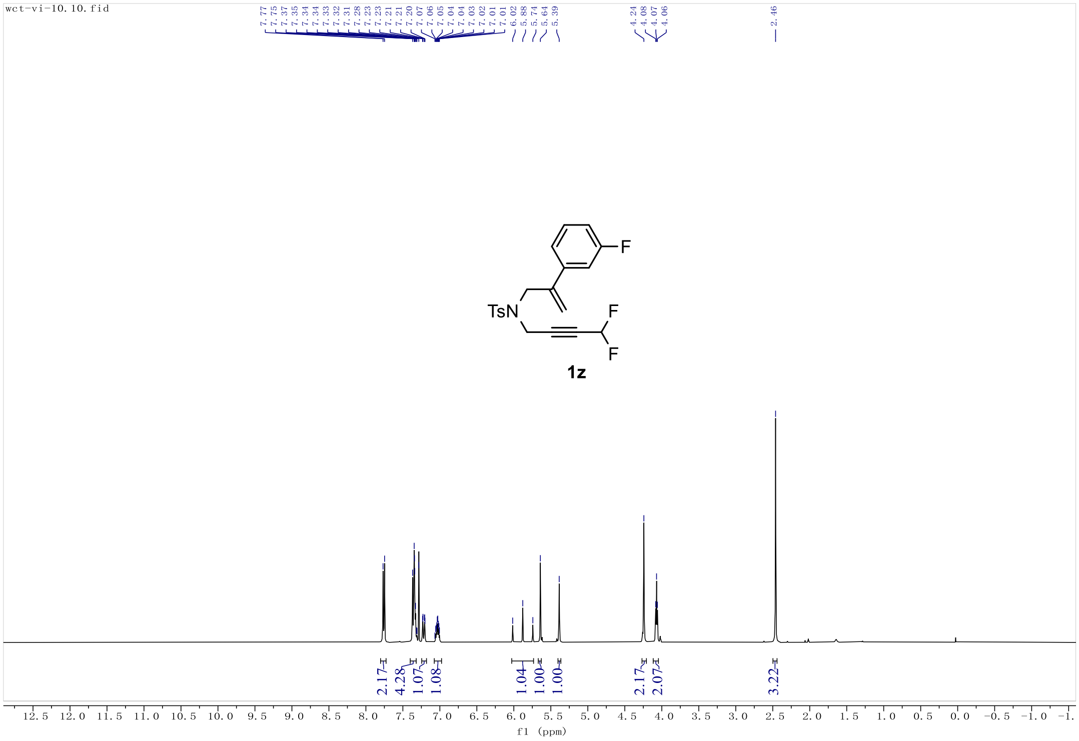
wct-vi-10.10.fid
7.77
7.75
7.37
7.35
7.34
7.34
7.33
7.32
7.31
7.28
7.23
7.23
7.21
7.21
7.20
7.07
7.06
7.05
7.04
7.04
7.03
7.02
7.01
7.01
6.02
5.88
5.74
5.64
5.39
4.24
4.08
4.07
4.06
2.46
TsN
F
F
F
1z
2.17
4.28
1.07
1.08
1.04
1.00
1.00
2.17
2.07
3.22
f1 (ppm)

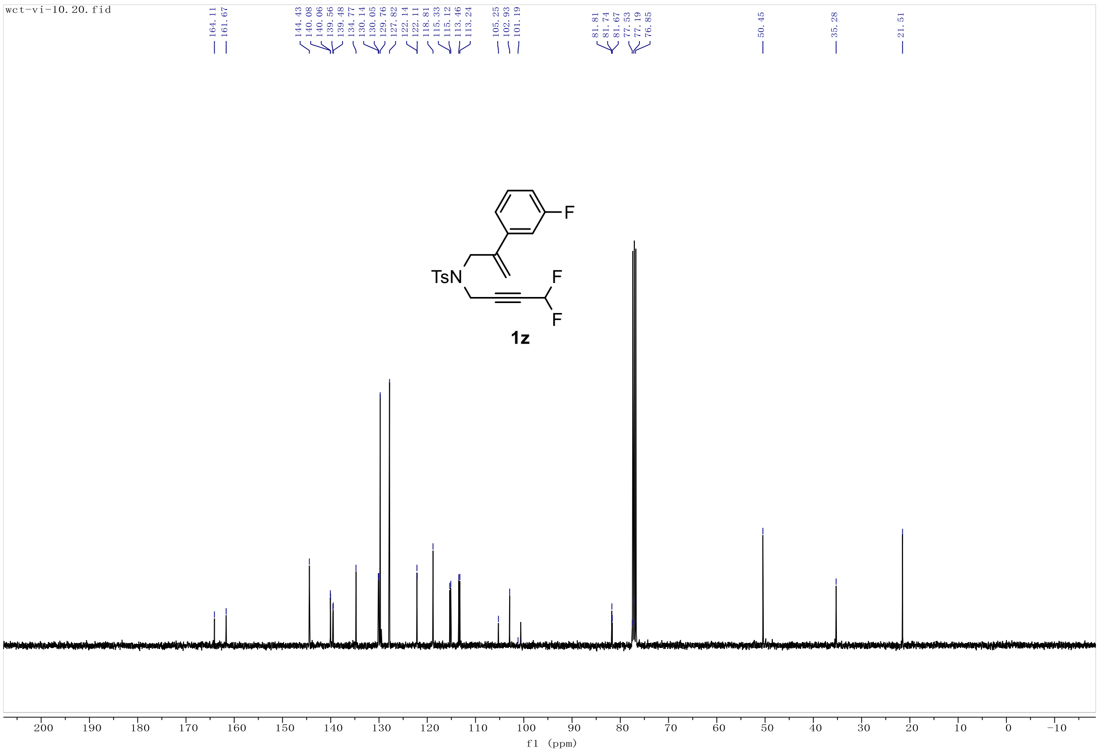
wct-vi-10.20.fid
164.11
161.67
144.43
140.08
140.06
139.56
139.48
134.77
130.14
130.05
129.76
127.82
122.14
122.11
118.81
115.33
115.12
113.46
113.24
105.25
102.93
101.19
81.81
81.74
81.67
77.53
77.19
76.85
50.45
35.28
21.51
TsN
F
F
F
1z
f1 (ppm)

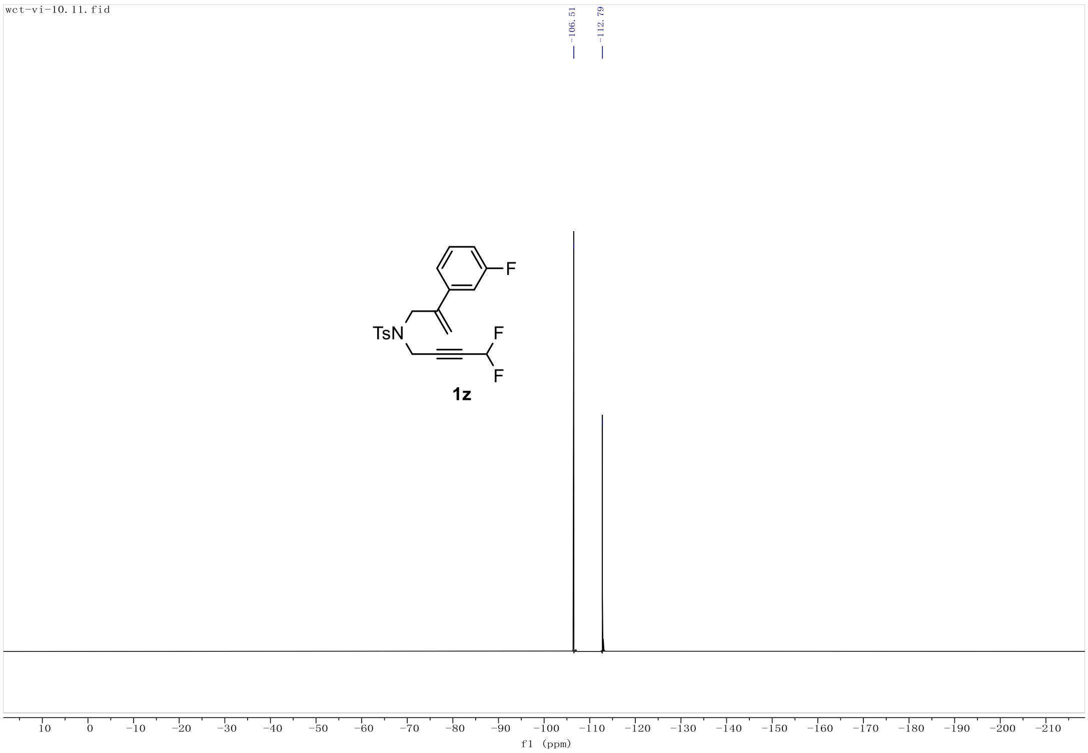
wct-vi-10.11.fid
-106.51
-112.79
TsN
F
F
F
1z
f1 (ppm)

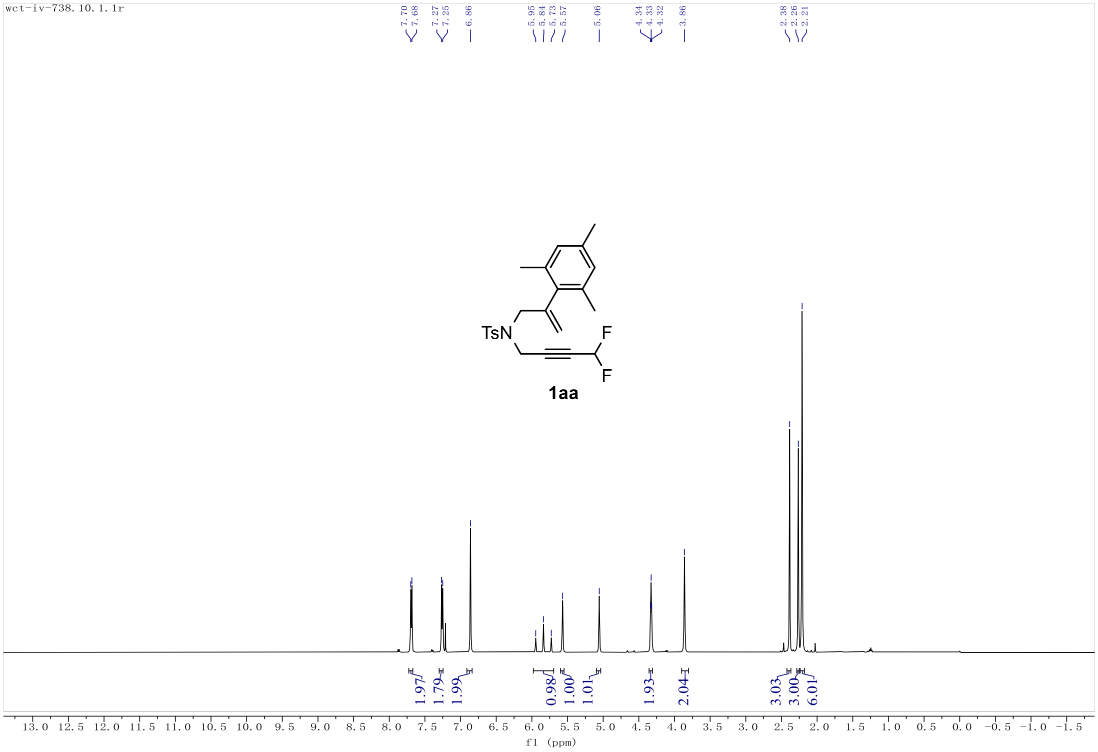

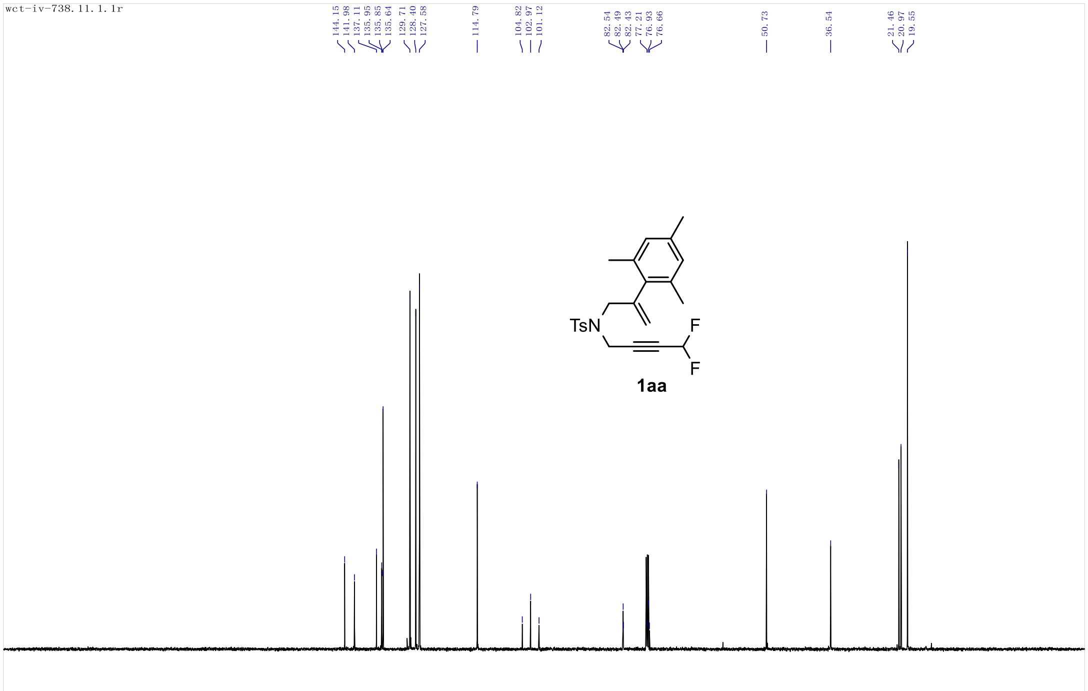

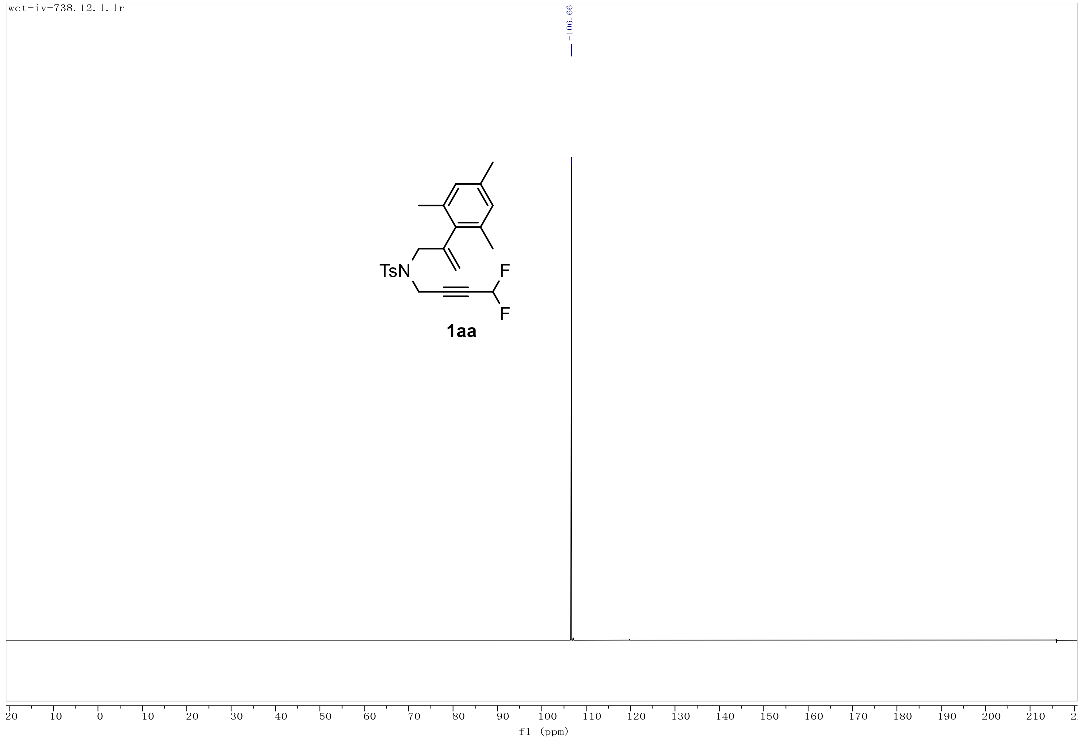
wct-iv-738.12.1.1r
-106.66
TsN
F
F
1aa
f1 (ppm)

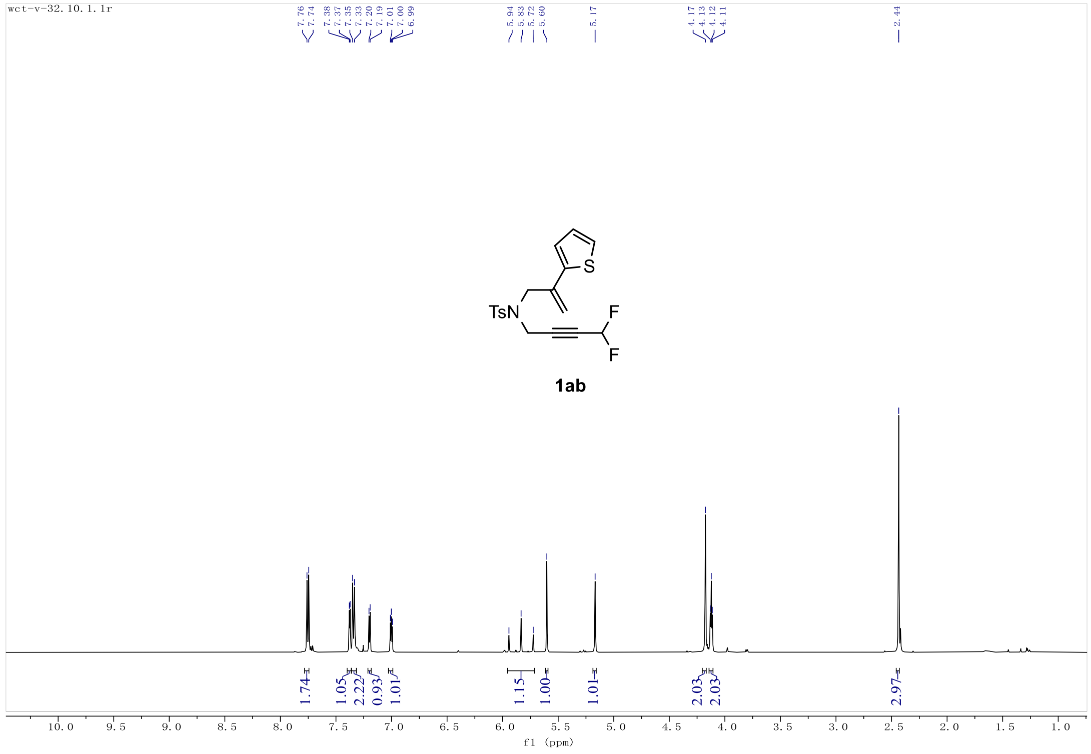
wct-v-32.10.1.1r
TsN
S
F
F
1ab
f1 (ppm)

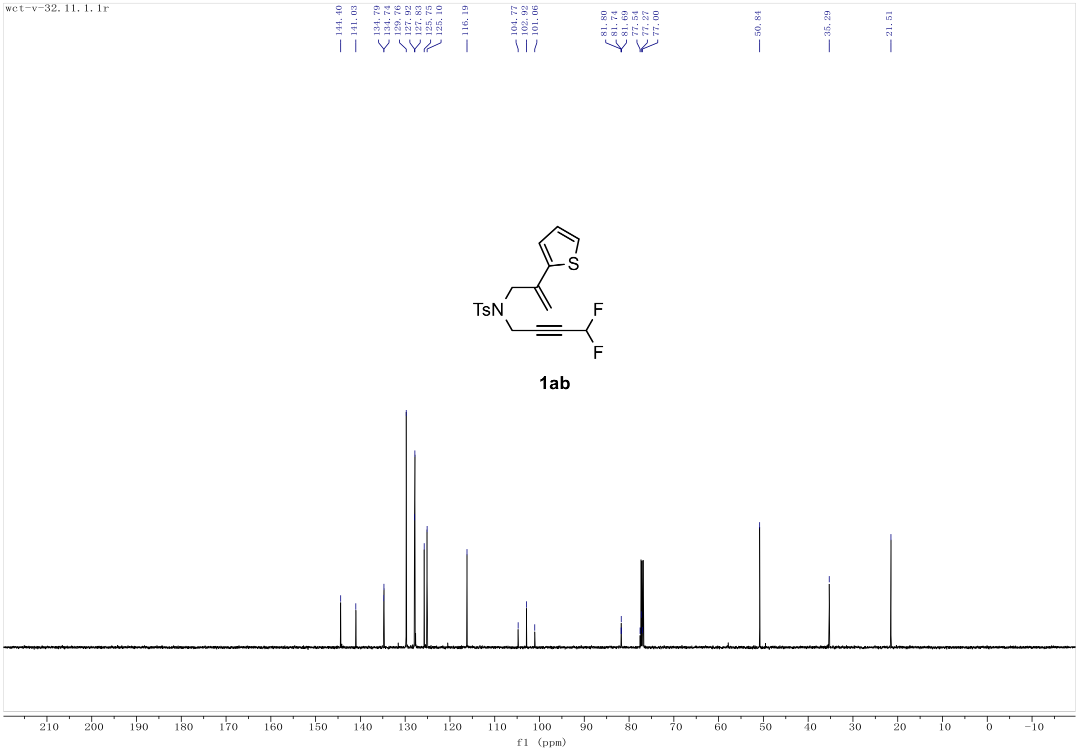
wct-v-32.11.1.1r
144.40
141.03
134.79
134.74
129.76
127.92
127.83
125.75
125.10
116.19
104.77
102.92
101.06
81.80
81.74
81.69
77.54
77.27
77.00
50.84
35.29
21.51
TsN
S
F
F
1ab
f1 (ppm)

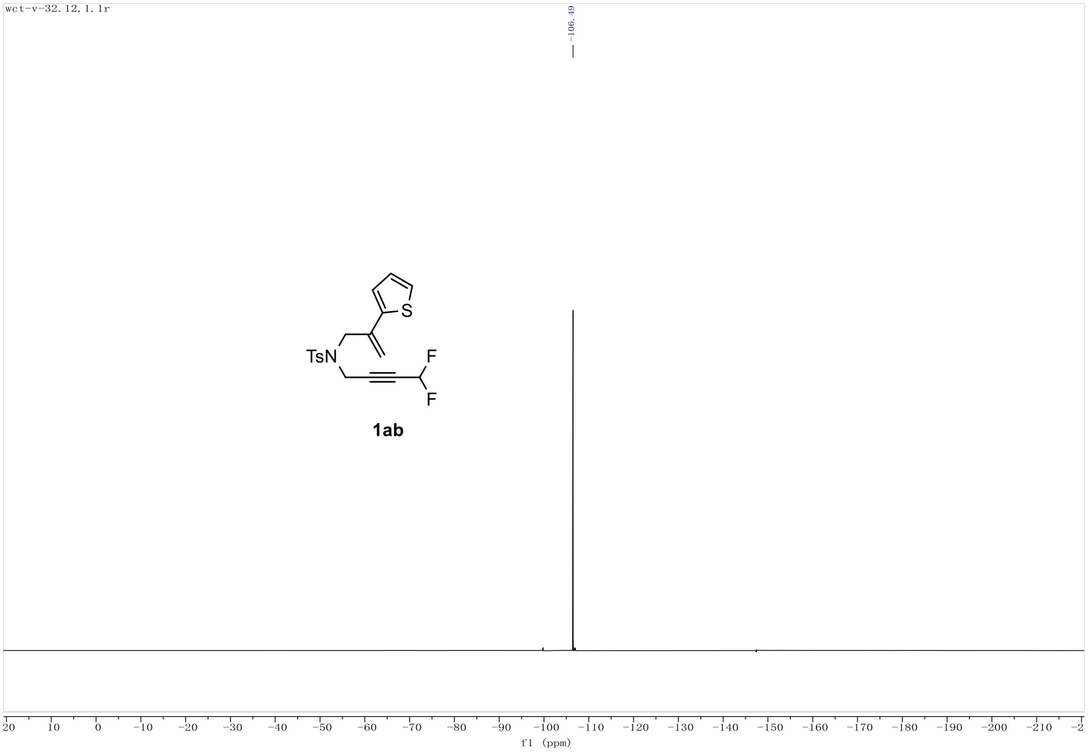
wct-v-32.12.1.1r
-106.49
TsN
S
F
F
1ab
f1 (ppm)

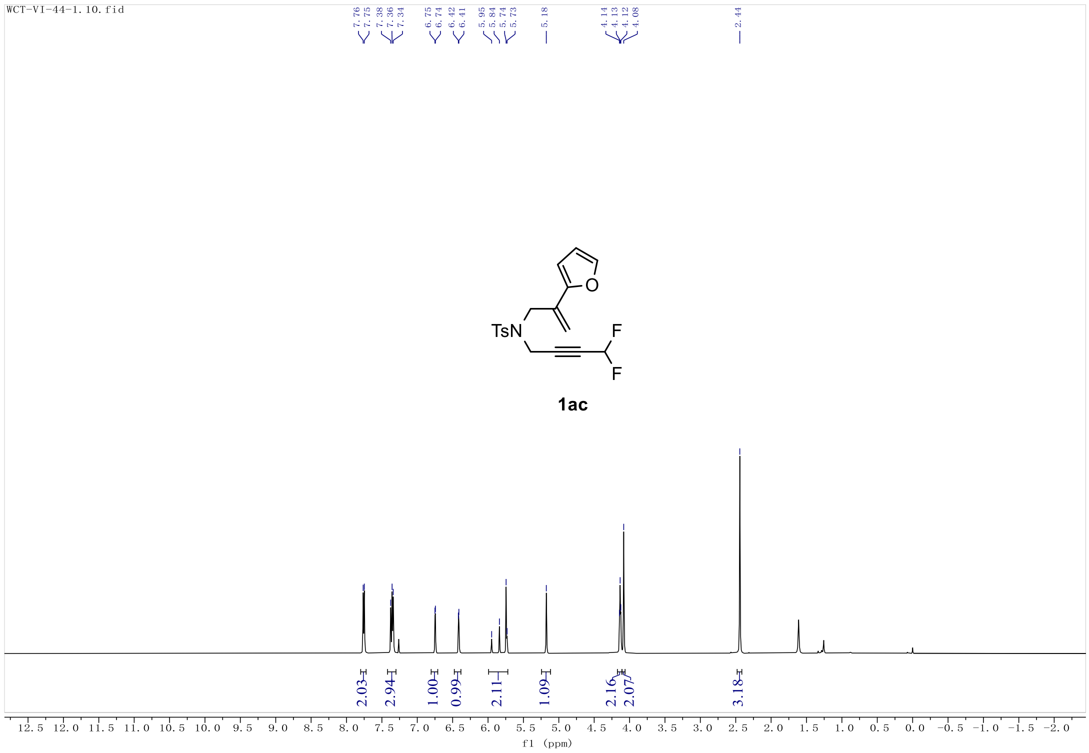
WCT-VI-44-1.10.fid
1ac
TsN
O
F
F
f1 (ppm)

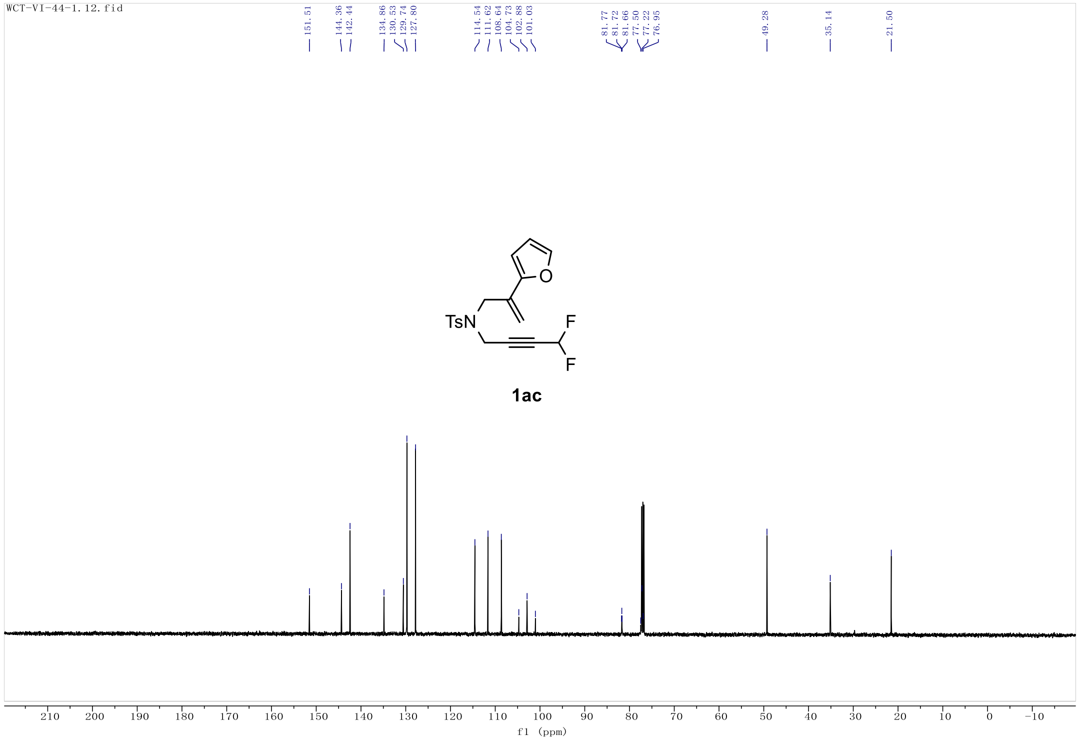
WCT-VI-44-1.12.fid
1ac
TsN
O
F
F
f1 (ppm)

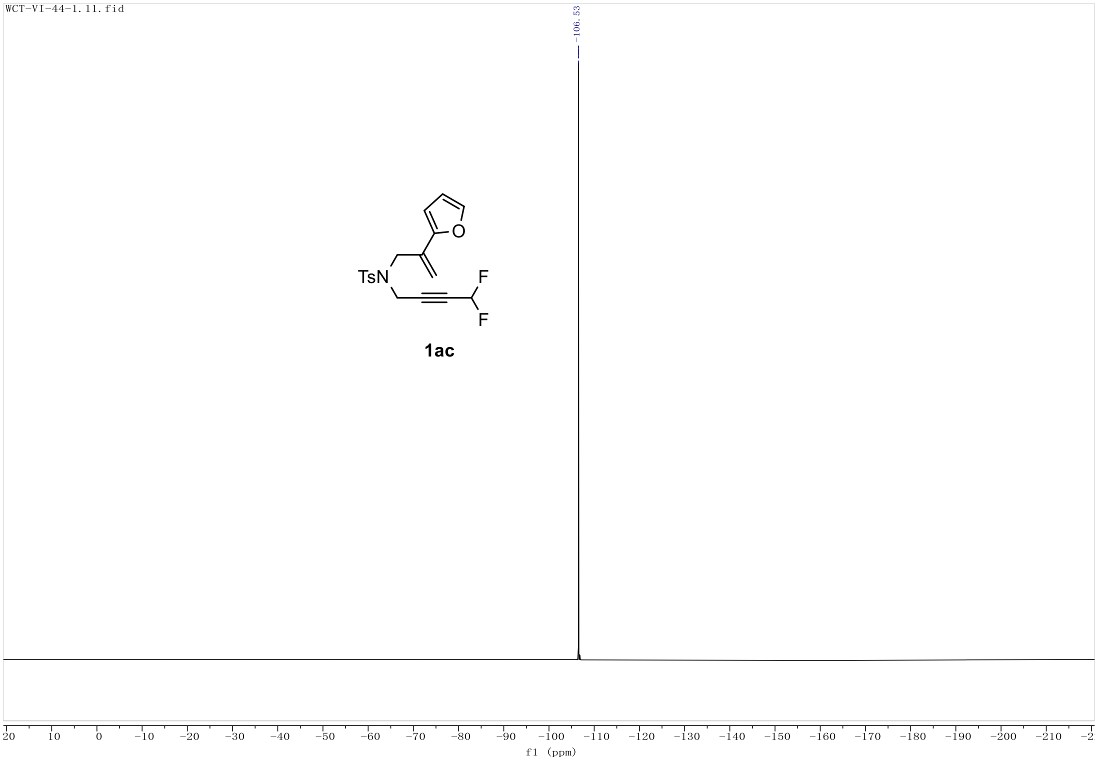
WCT-VI-44-1.11.fid
-106.53
TsN
F
F
O
1ac
f1 (ppm)

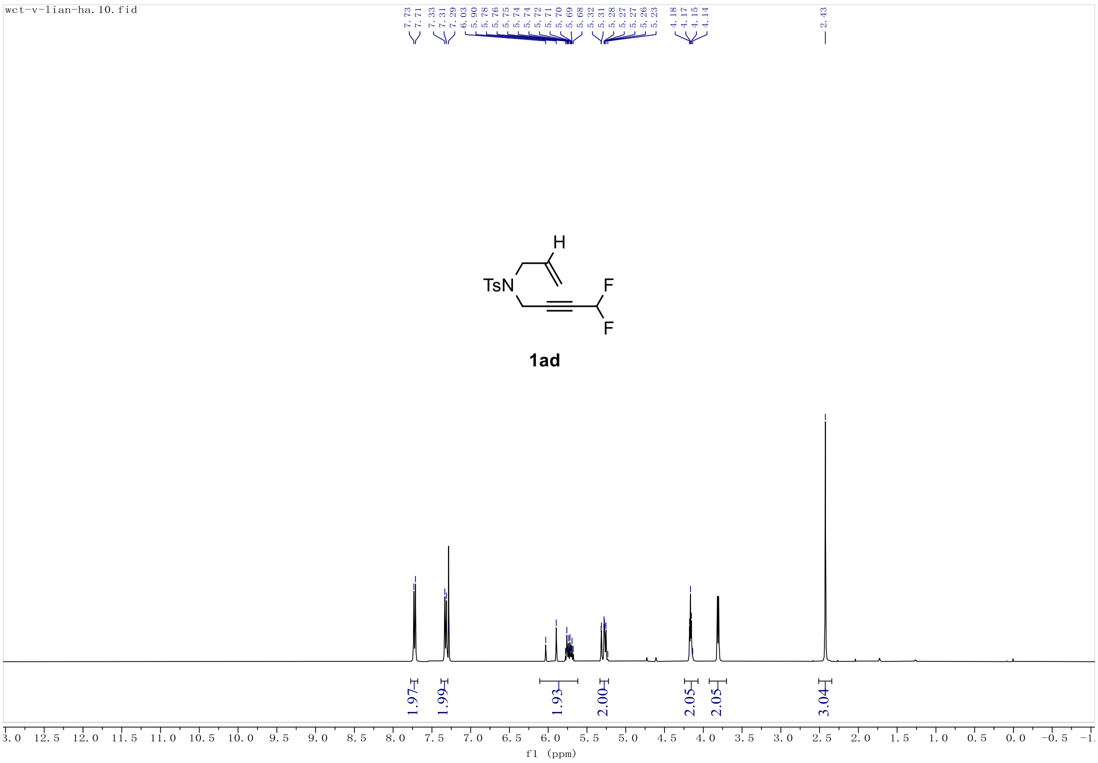
wct-v-lian-ha.10.fid
7.73
7.71
7.33
7.31
7.29
6.03
5.90
5.78
5.76
5.75
5.74
5.72
5.71
5.70
5.69
5.68
5.32
5.31
5.28
5.27
5.26
5.23
4.18
4.17
4.15
4.14
2.43
H
TsN
F
F
1ad
1.97
1.99
1.93
2.00
2.05
2.05
3.04
f1 (ppm)

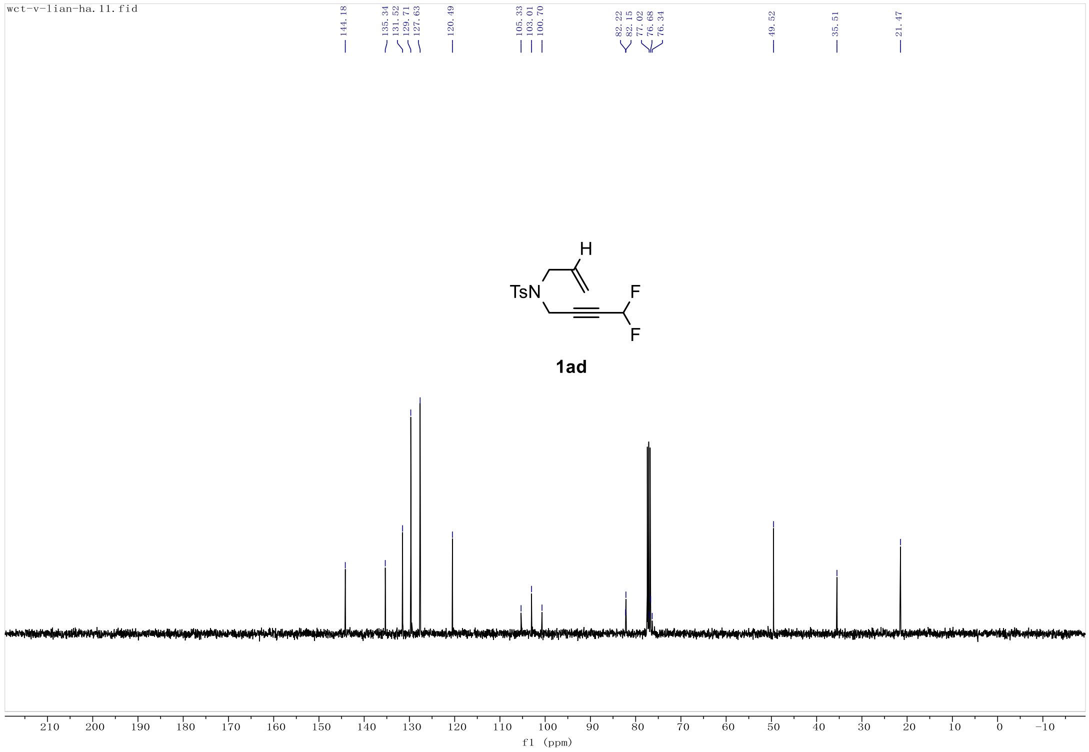
wct-v-lian-ha.11.fid
144.18
135.34
131.52
129.71
127.63
120.49
105.33
103.01
100.70
82.22
82.15
77.02
76.68
76.34
49.52
35.51
21.47
TsN
H
F
F
1ad
f1 (ppm)

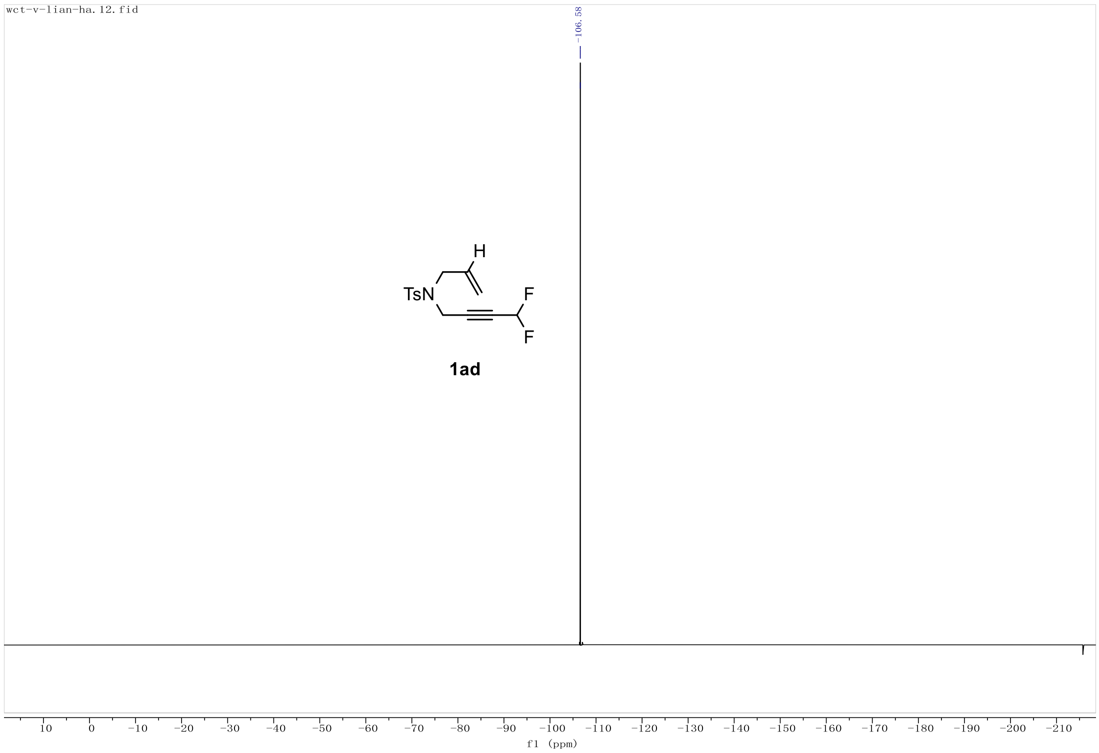
wct-v-lian-ha.12.fid
-106.58
TsN
H
F
F
1ad
f1 (ppm)

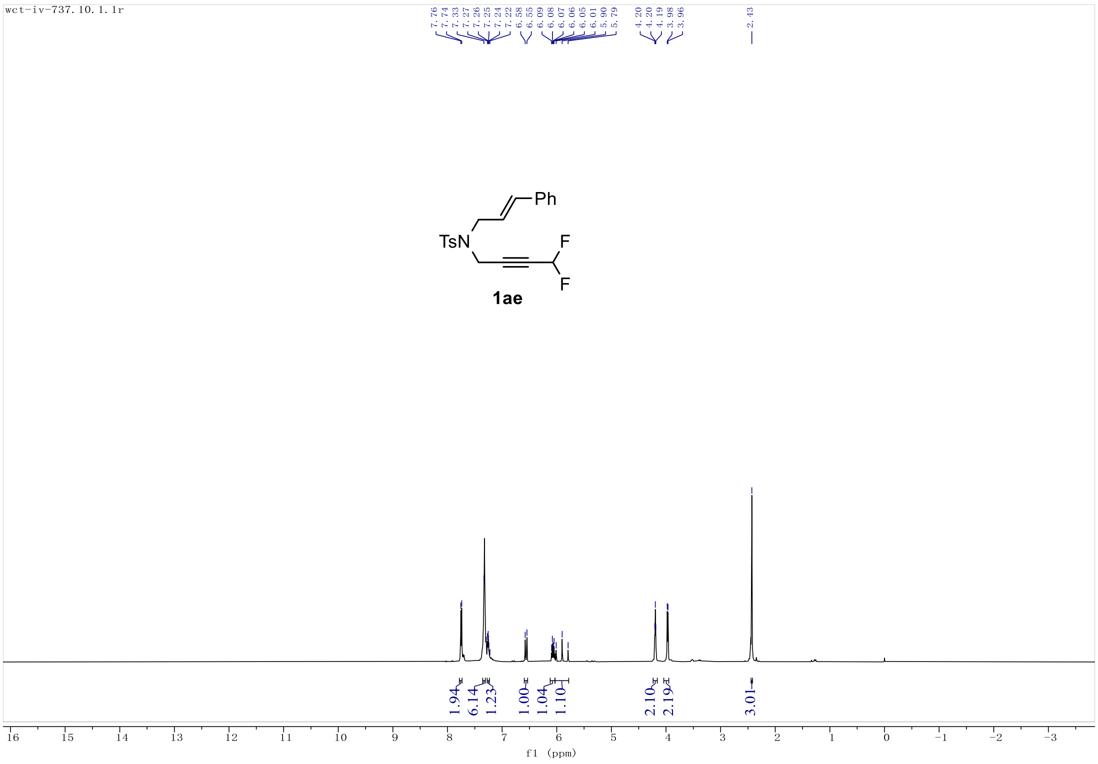
wct-iv-737.10.1.1r
7.76
7.74
7.33
7.27
7.26
7.25
7.24
7.22
6.58
6.55
6.09
6.08
6.07
6.06
6.05
6.01
5.90
5.79
4.20
4.20
4.19
3.98
3.96
2.43
Ph
TsN
F
F
1ae
1.94
6.14
1.23
1.00
1.04
1.10
2.10
2.19
3.01
f1 (ppm)

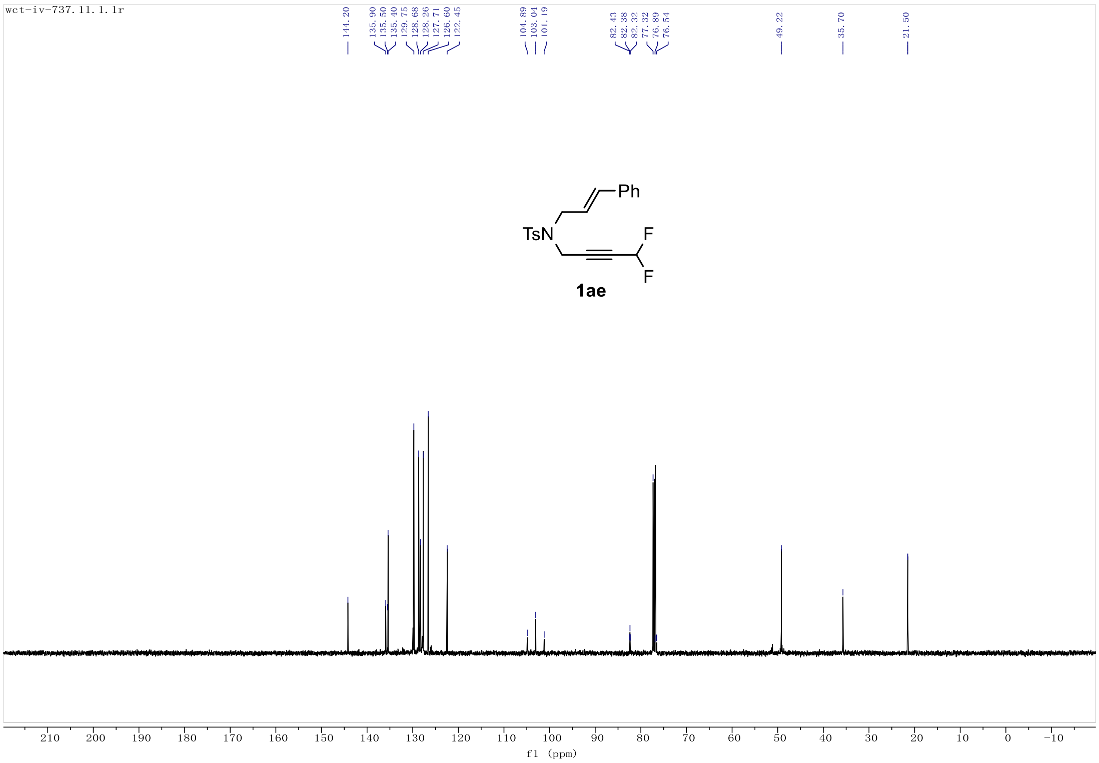
wct-iv-737.11.1.1r
144.20
135.90
135.50
135.40
129.75
128.68
128.26
127.71
126.60
122.45
104.89
103.04
101.19
82.43
82.38
82.32
77.32
76.89
76.54
49.22
35.70
21.50
Ph
TsN
F
F
1ae
f1 (ppm)

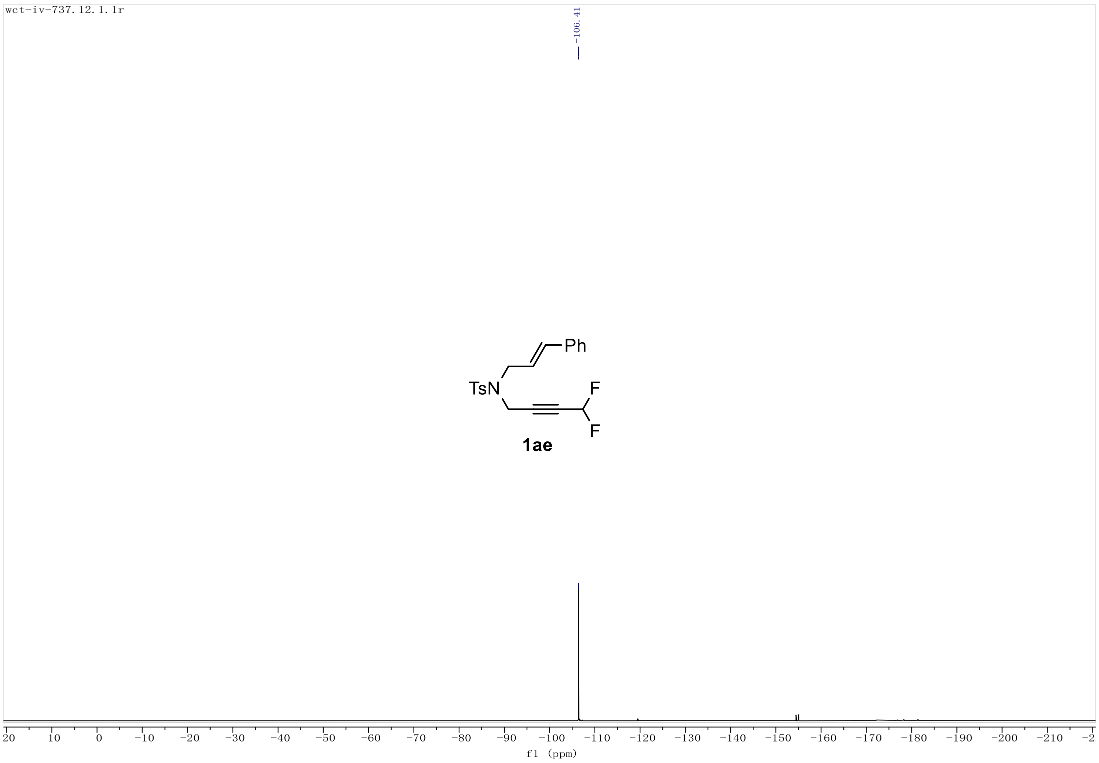
wct-iv-737.12.1.1r
-106.41
TsN
Ph
F
F
f1 (ppm)

1ae

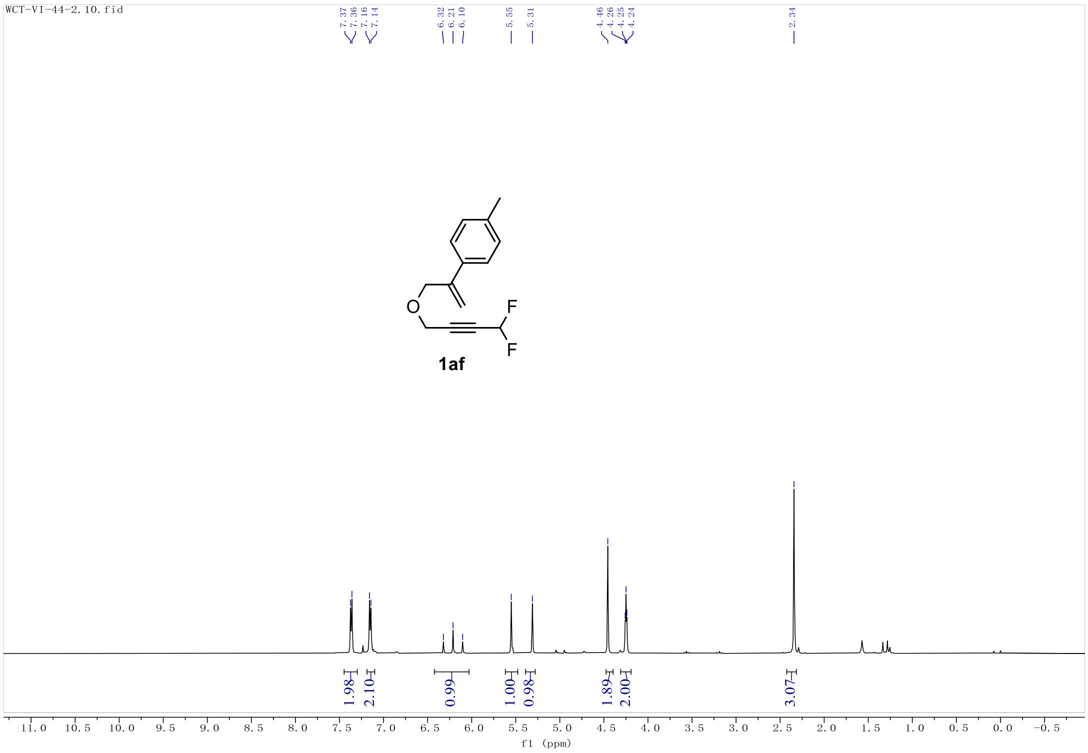
WCT-VI-44-2.10.fid
7.37
7.36
7.16
7.14
6.32
6.21
6.10
5.55
5.31
4.46
4.26
4.25
4.24
2.34
O
F
F
1.98
2.10
0.99
1.00
0.98
1.89
2.00
3.07
f1 (ppm)

1af

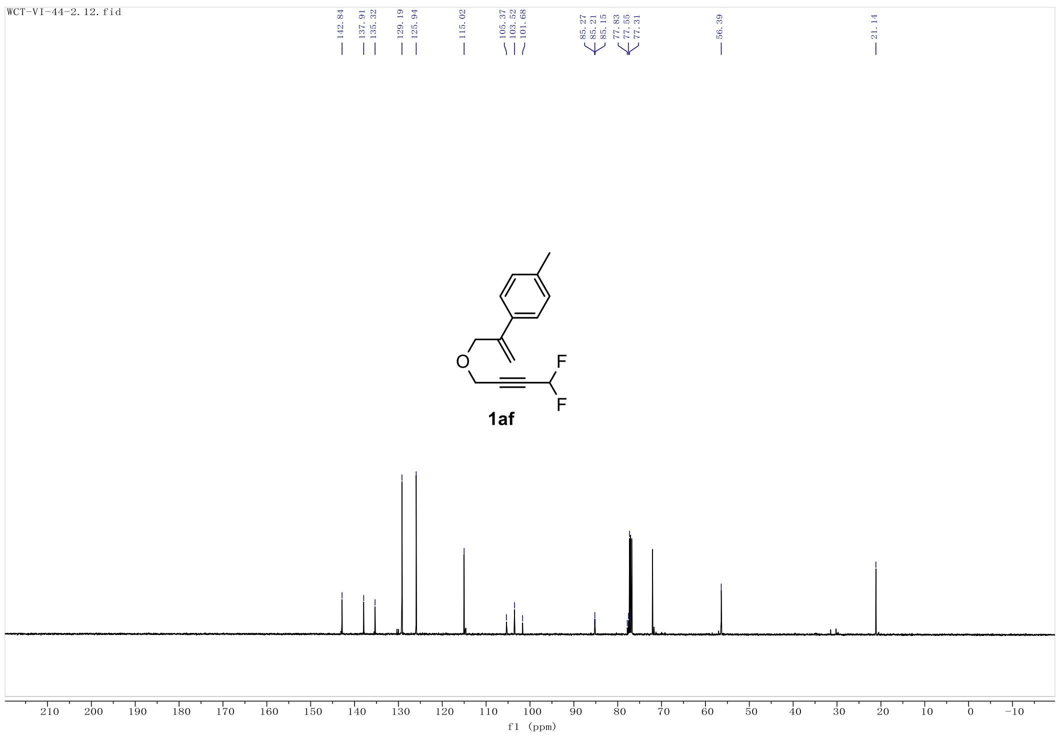
WCT-VI-44-2.12.fid
142.84
137.91
135.32
129.19
125.94
115.02
105.37
103.52
101.68
85.27
85.21
85.15
77.83
77.55
77.31
56.39
21.14
O
F
F
1af
f1 (ppm)

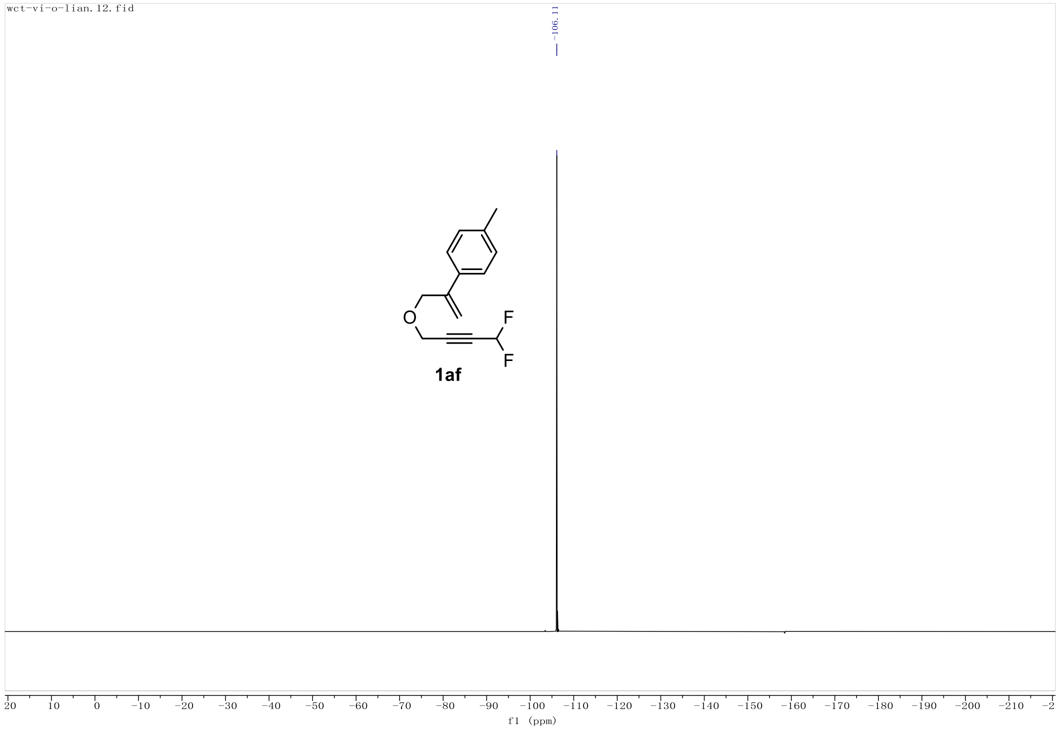
wct-vi-o-lian.12.fid
-106.11
O
F
F
1af
f1 (ppm)

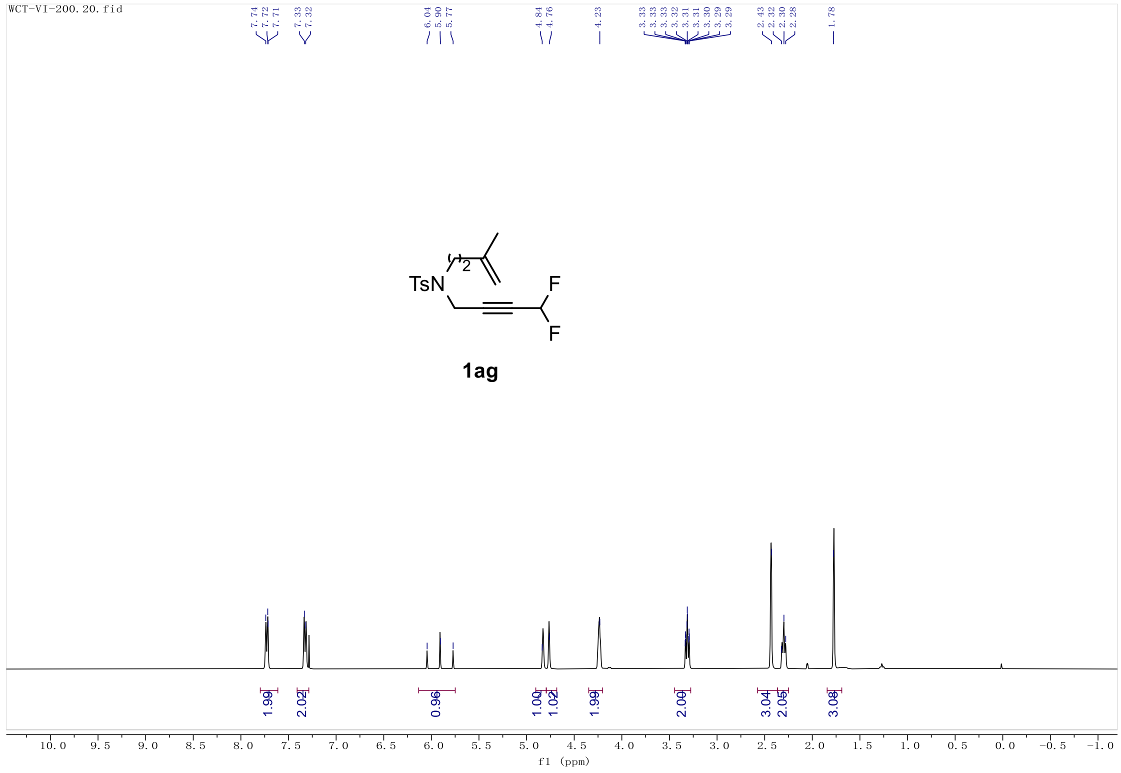
WCT-VI-200.20.fid
7.74
7.72
7.71
7.33
7.32
6.04
5.90
5.77
4.84
4.76
4.23
3.33
3.33
3.33
3.32
3.31
3.30
3.29
2.43
2.32
2.30
2.28
1.78
TsN
2
F
F
1ag
1.99
2.02
0.96
1.00
1.02
1.99
2.00
3.04
2.06
3.08
f1 (ppm)

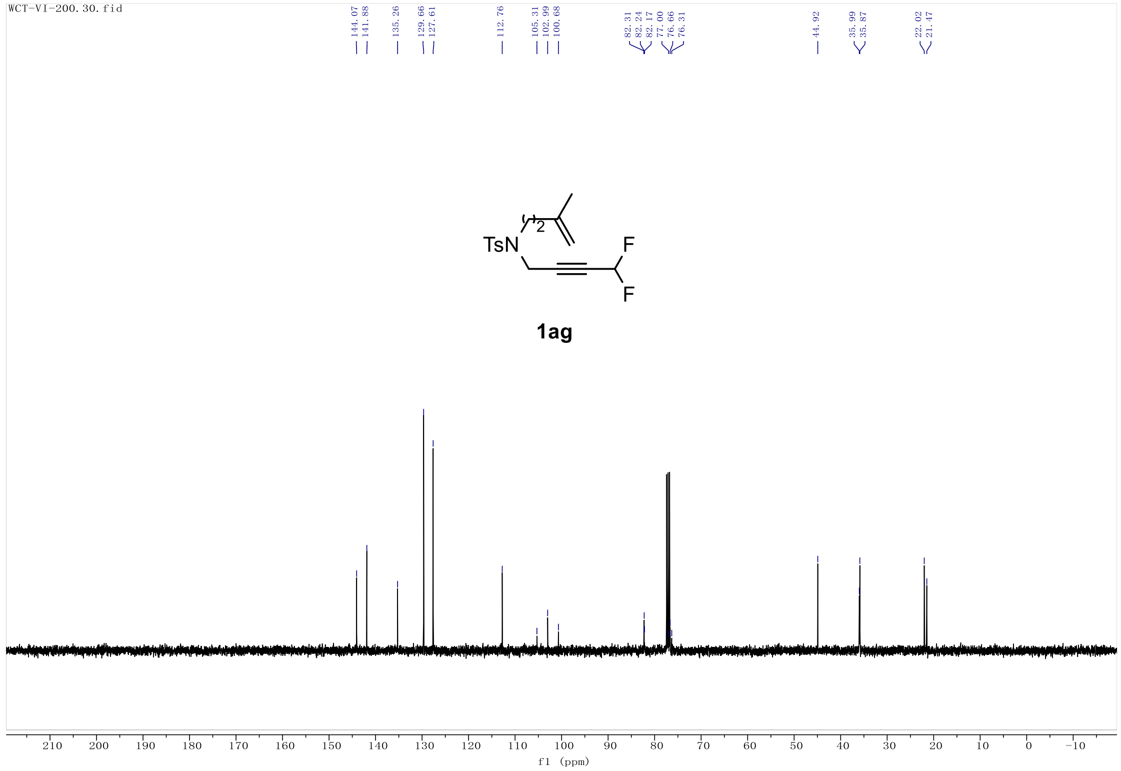
WCT-VI-200.30.fid
144.07
141.88
135.26
129.66
127.61
112.76
105.31
102.99
100.68
82.31
82.24
82.17
77.00
76.66
76.31
44.92
35.99
35.87
22.02
21.47
TsN
2
F
F
1ag
f1 (ppm)

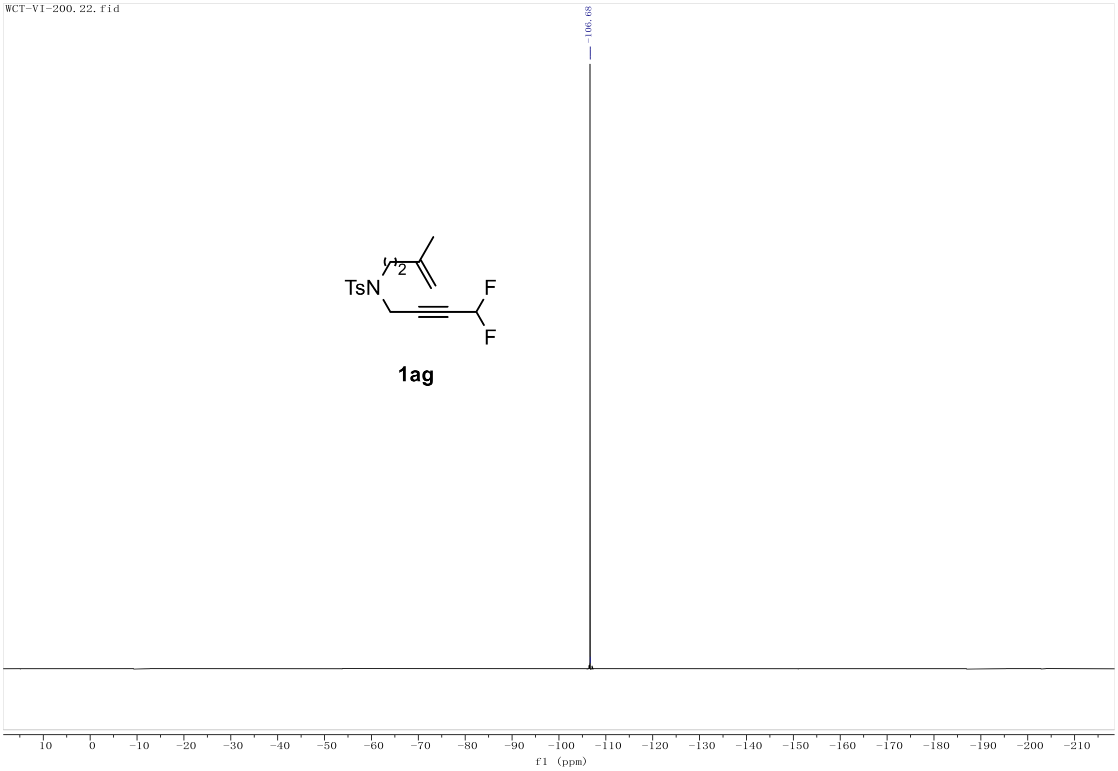
WCT-VI-200.22.fid
-106.68
TsN
2
F
F
f1 (ppm)

1ag

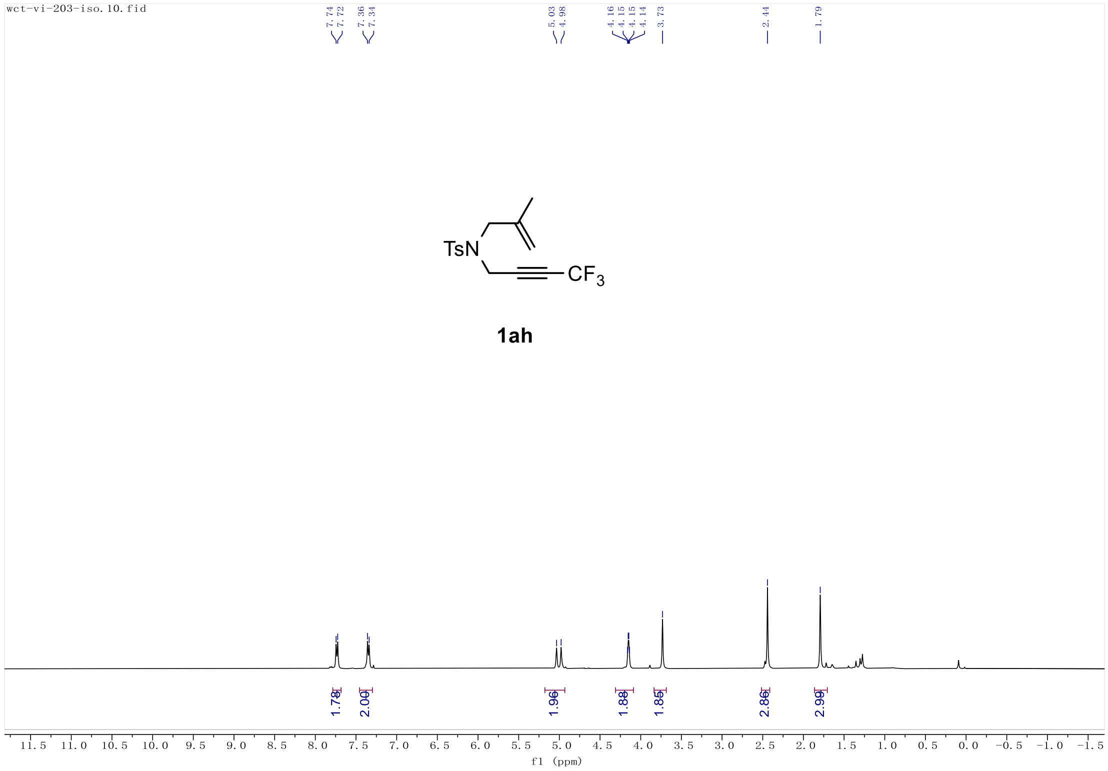
wct-vi-203-iso.10.fid
7.74
7.72
7.36
7.34
5.03
4.98
4.16
4.15
4.15
4.14
3.73
2.44
1.79
TsN
CF3
1.78
2.00
1.96
1.88
1.85
2.86
2.99
f1 (ppm)

1ah

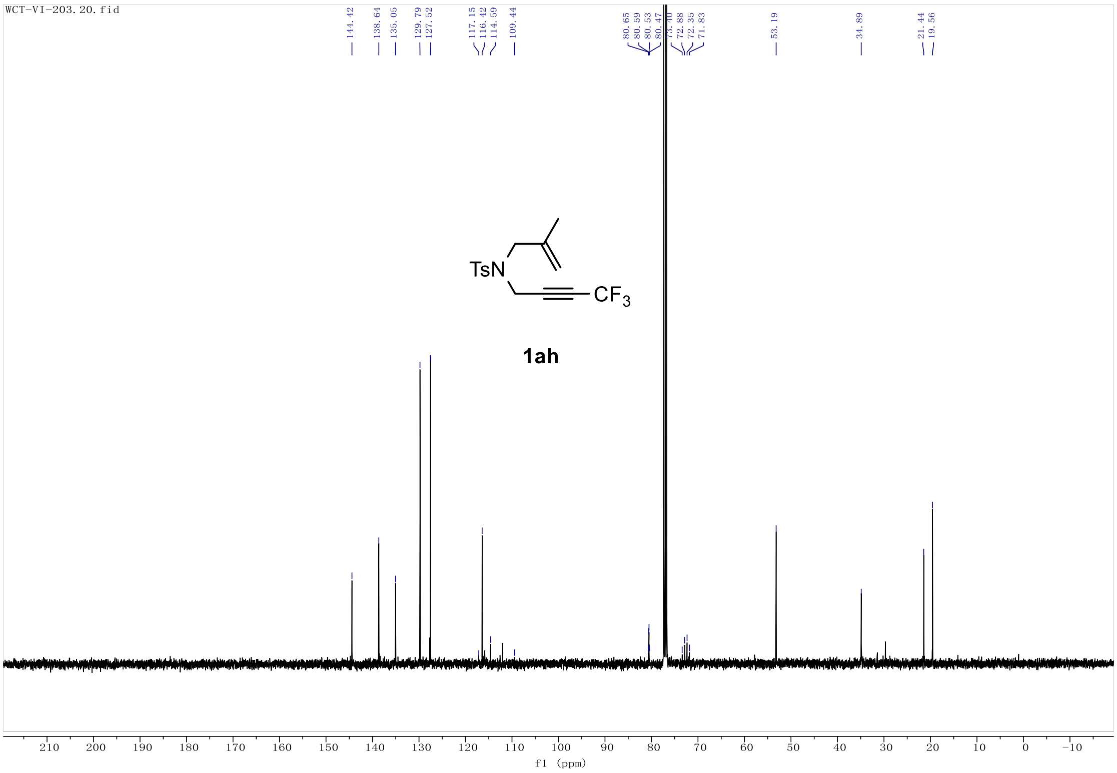
WCT-VI-203.20.fid
144.42
138.64
135.05
129.79
127.52
117.15
116.42
114.59
109.44
80.65
80.59
80.53
80.47
73.40
72.88
72.35
71.83
53.19
34.89
21.44
19.56
TsN
CF3
1ah
210 200 190 180 170 160 150 140 130 120 110 100 90 80 70 60 50 40 30 20 10 0 -10
f1 (ppm)

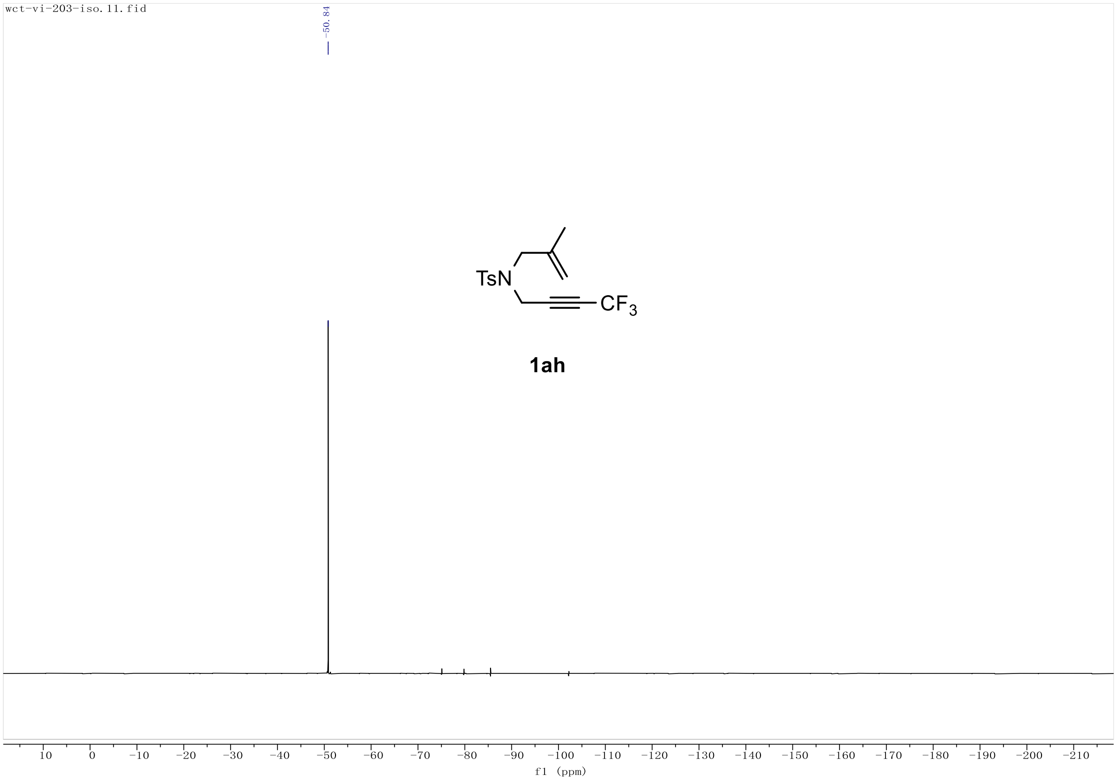
wct-vi-203-iso.11.fid
-50.84
TsN
CF3
1ah
10 0 -10 -20 -30 -40 -50 -60 -70 -80 -90 -100 -110 -120 -130 -140 -150 -160 -170 -180 -190 -200 -210
f1 (ppm)

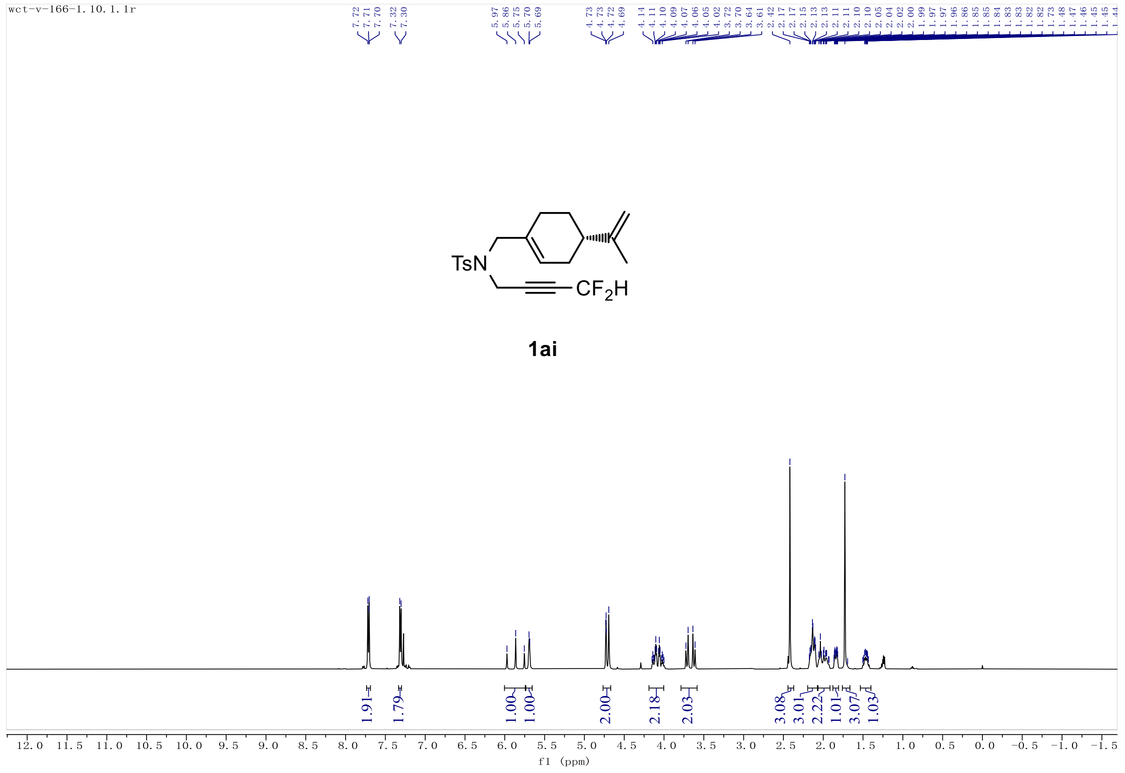
wct-v-166-1.10.1.1r
TsN
CF2H
1ai
f1 (ppm)

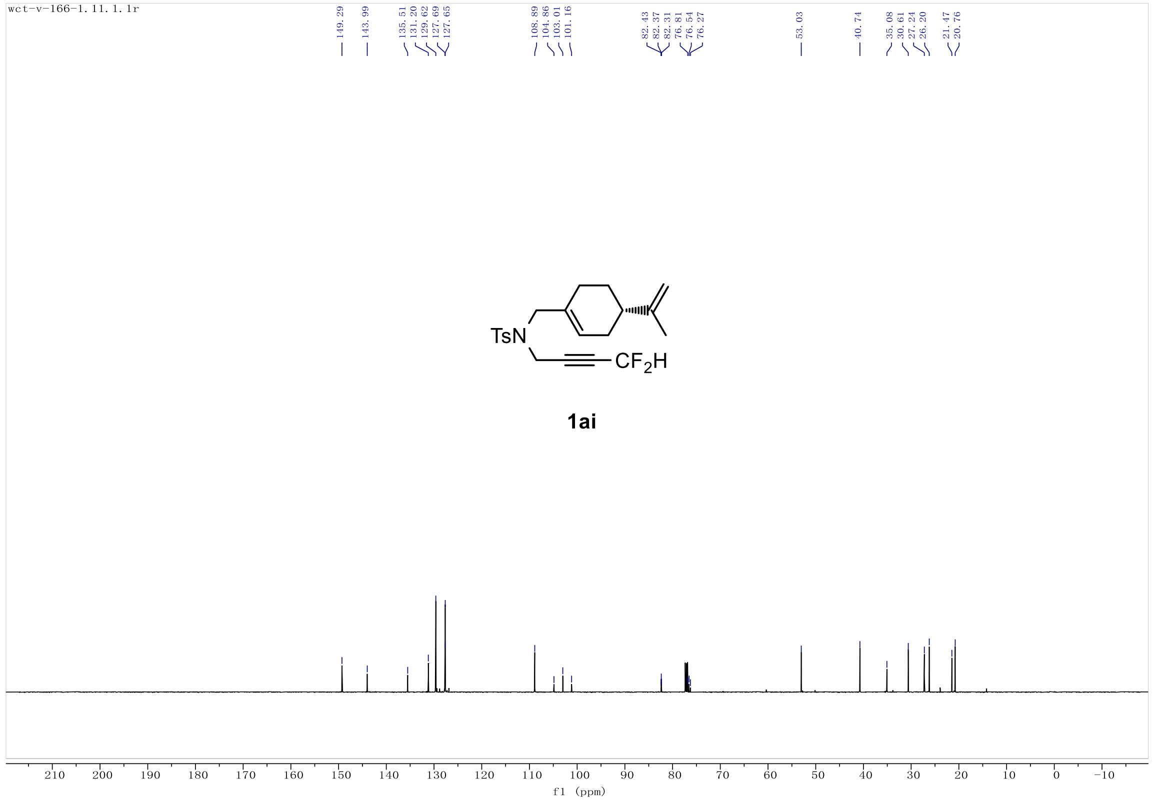
wct-v-166-1.11.1.1r
TsN
CF2H
1ai
f1 (ppm)

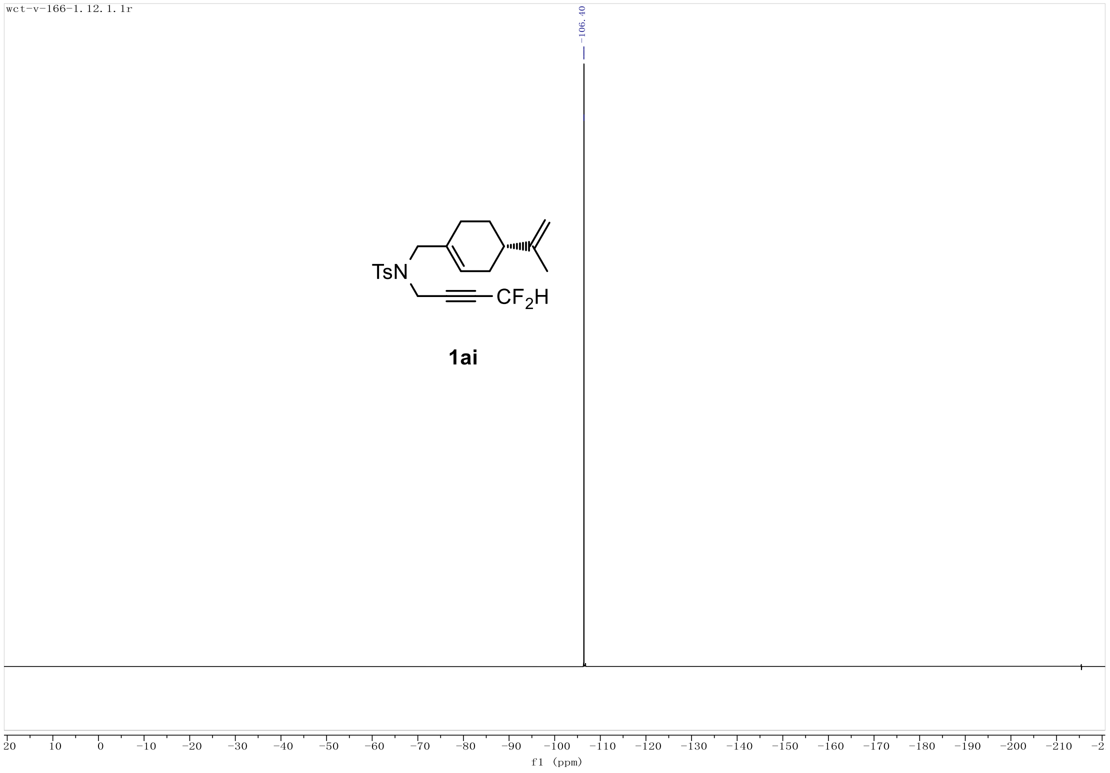
wct-v-166-1.12.1.1r
-106.40
TsN
CF2H
1ai
20 10 0 −10 −20 −30 −40 −50 −60 −70 −80 −90 −100 −110 −120 −130 −140 −150 −160 −170 −180 −190 −200 −210
f1 (ppm)

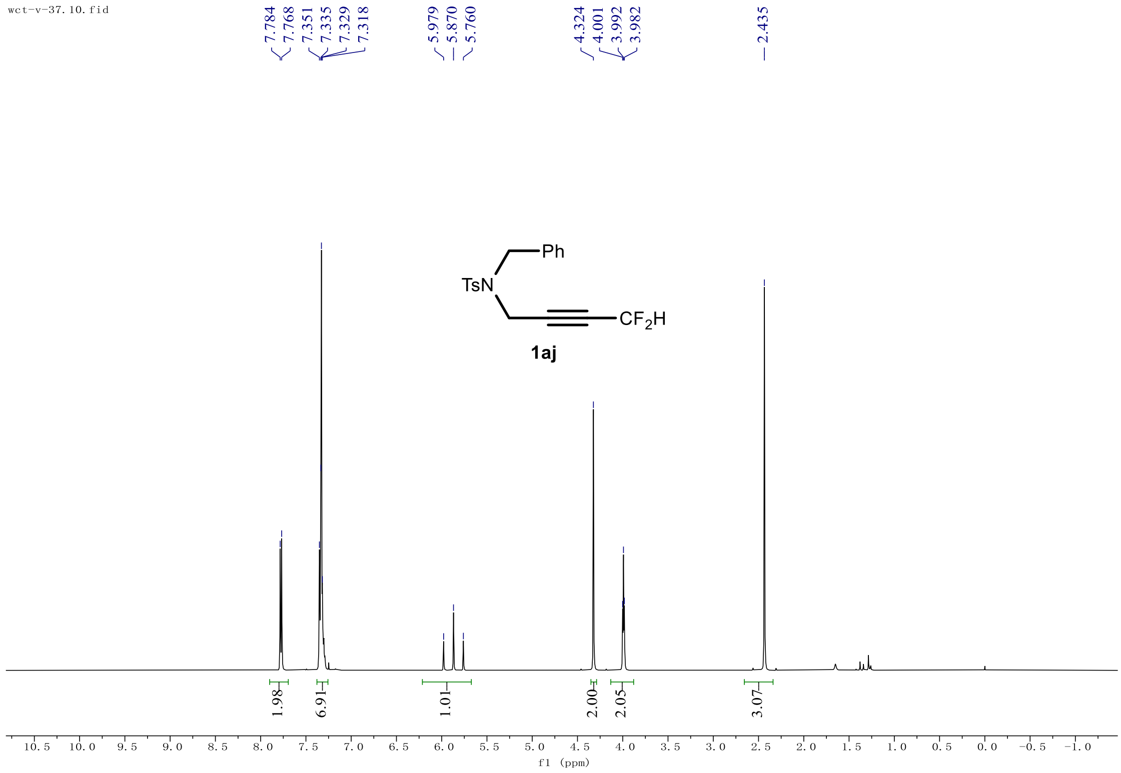
wct-v-37.10.fid
7.784
7.768
7.351
7.335
7.329
7.318
5.979
5.870
5.760
4.324
4.001
3.992
3.982
2.435
Ph
TsN
CF2H
1aj
1.98
6.91
1.01
2.00
2.05
3.07
10.5 10.0 9.5 9.0 8.5 8.0 7.5 7.0 6.5 6.0 5.5 5.0 4.5 4.0 3.5 3.0 2.5 2.0 1.5 1.0 0.5 0.0 −0.5 −1.0
f1 (ppm)

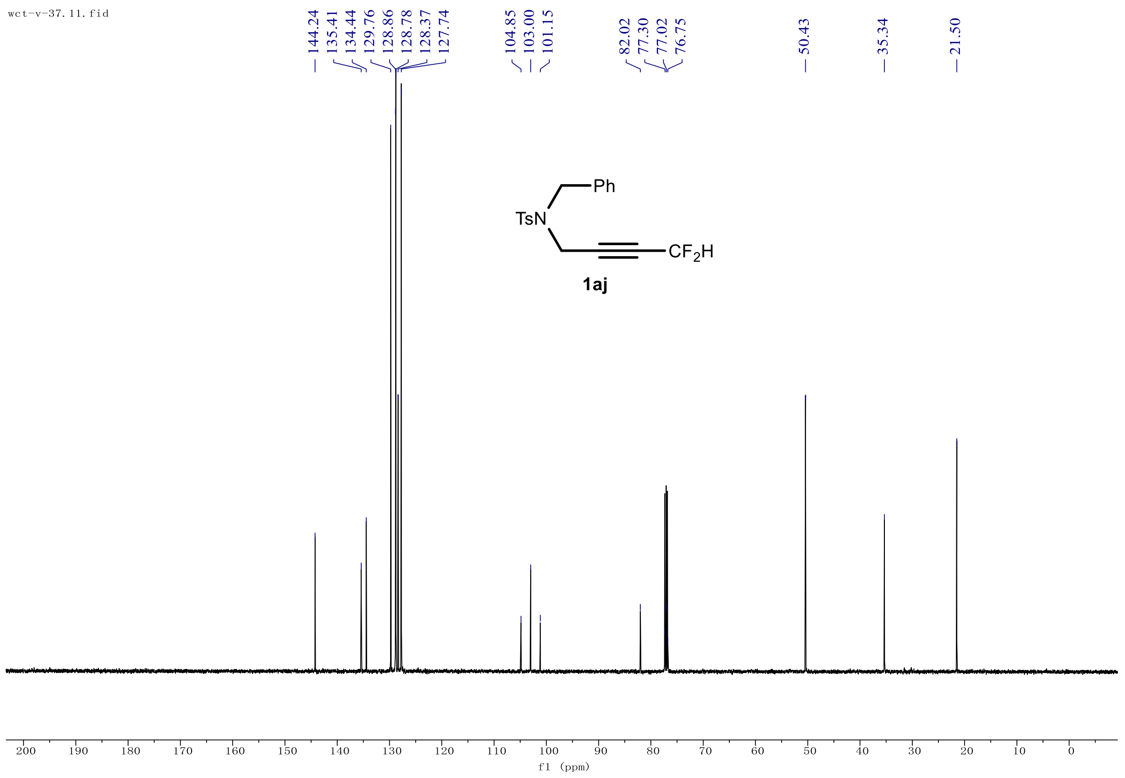

wct-v-37.11.fid
144.24
135.41
134.44
129.76
128.86
128.78
128.37
127.74
104.85
103.00
101.15
82.02
77.30
77.02
76.75
50.43
35.34
21.50
Ph
TsN
CF2H
1aj
200 190 180 170 160 150 140 130 120 110 100 90 80 70 60 50 40 30 20 10 0
f1 (ppm)

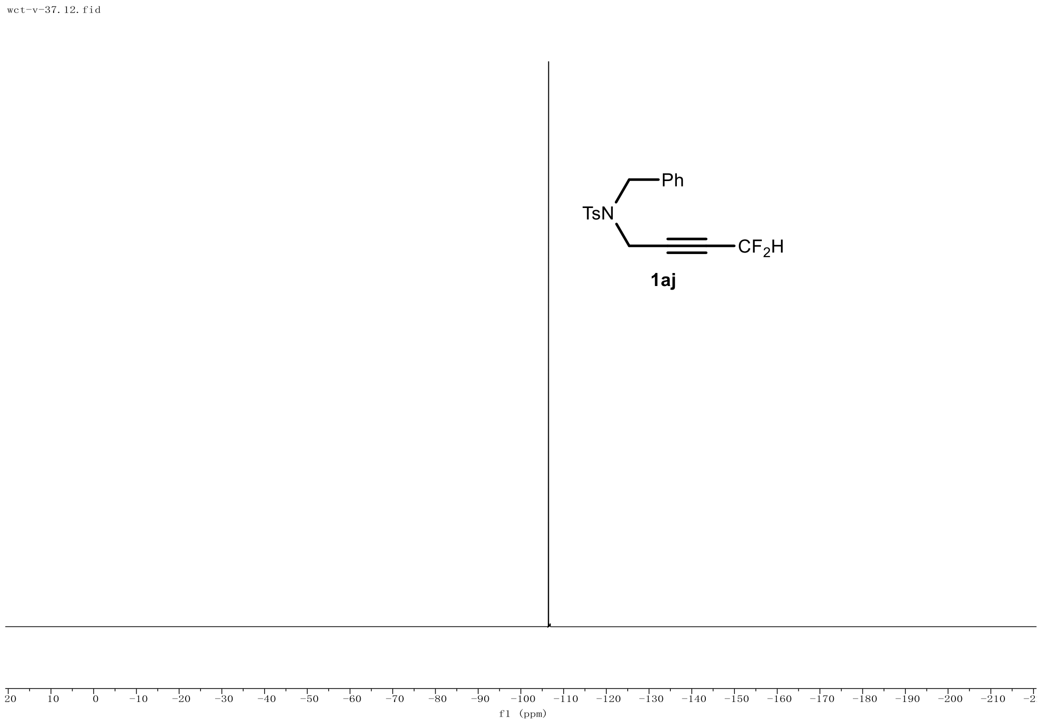

wct-v-37.12.fid
Ph
TsN
CF2H
1aj
20 10 0 -10 -20 -30 -40 -50 -60 -70 -80 -90 -100 -110 -120 -130 -140 -150 -160 -170 -180 -190 -200 -210
f1 (ppm)

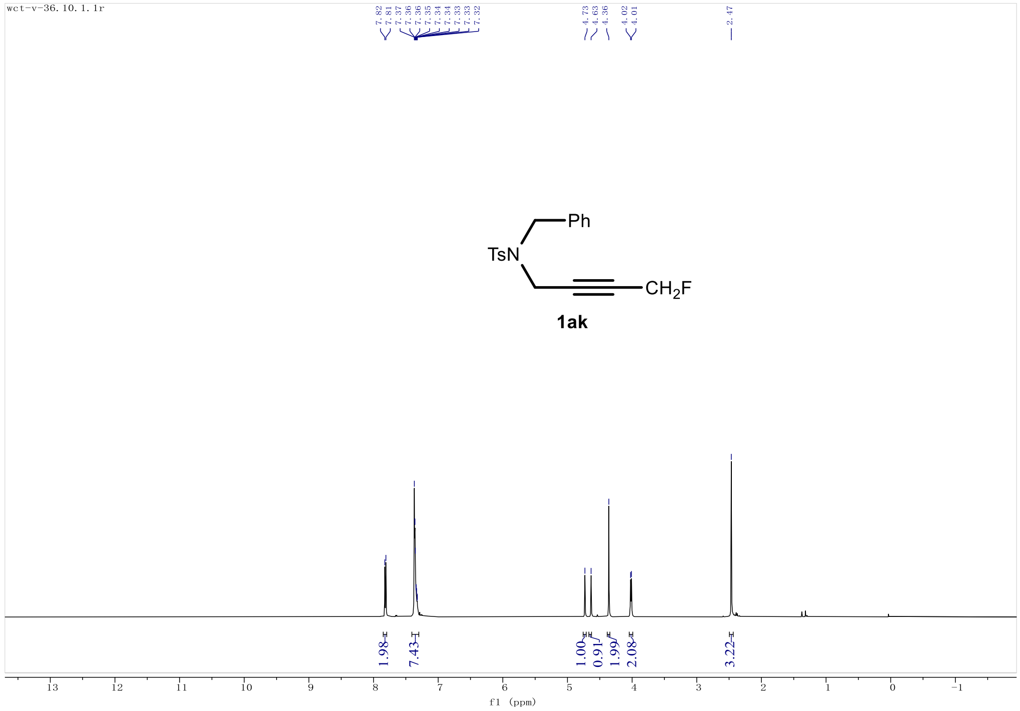
wct-v-36.10.1.1r
7.82
7.81
7.37
7.36
7.36
7.35
7.34
7.34
7.33
7.33
7.32
4.73
4.63
4.36
4.02
4.01
2.47
Ph
TsN
CH2F
1ak
1.98
7.43
1.00
0.91
1.99
2.08
3.22
13
12
11
10
9
8
7
6
5
4
3
2
1
0
-1
f1 (ppm)

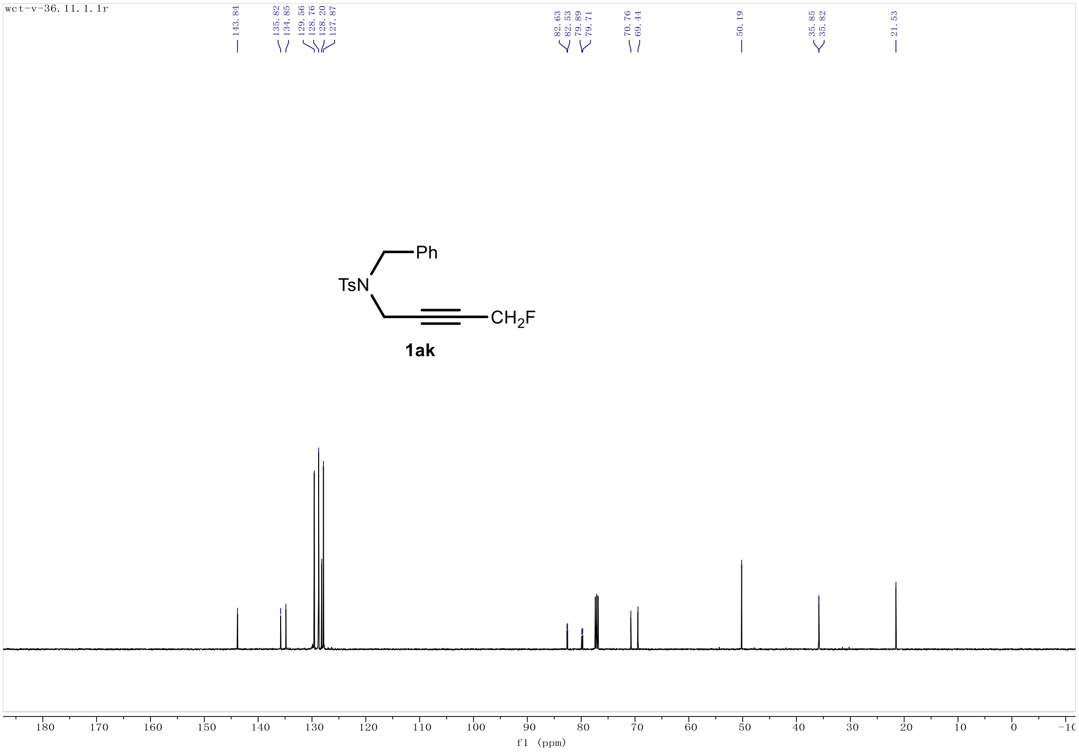
wct-v-36.11.1.1r
143.84
135.82
134.85
129.56
128.76
128.20
127.87
82.63
82.53
79.89
79.71
70.76
69.44
50.19
35.85
35.82
21.53
Ph
TsN
CH2F
1ak
180
170
160
150
140
130
120
110
100
90
80
70
60
50
40
30
20
10
0
-10
f1 (ppm)

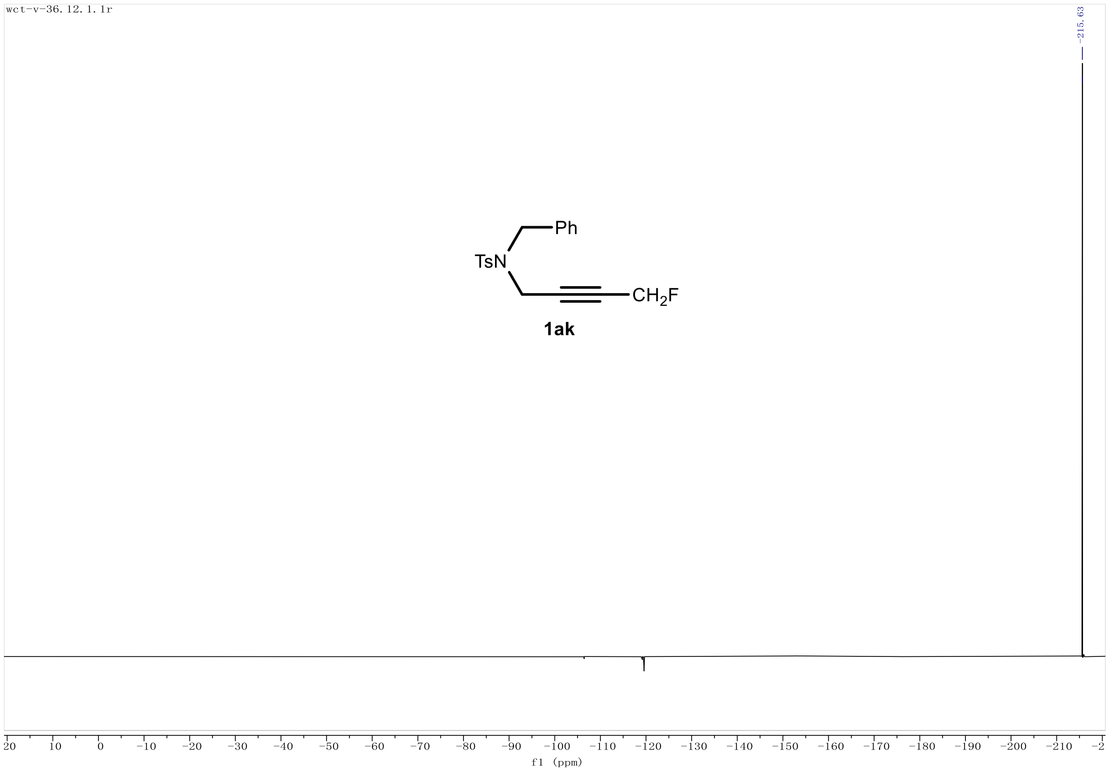
wct-v-36.12.1.1r
-215.63
TsN
Ph
CH2F
1ak
f1 (ppm)

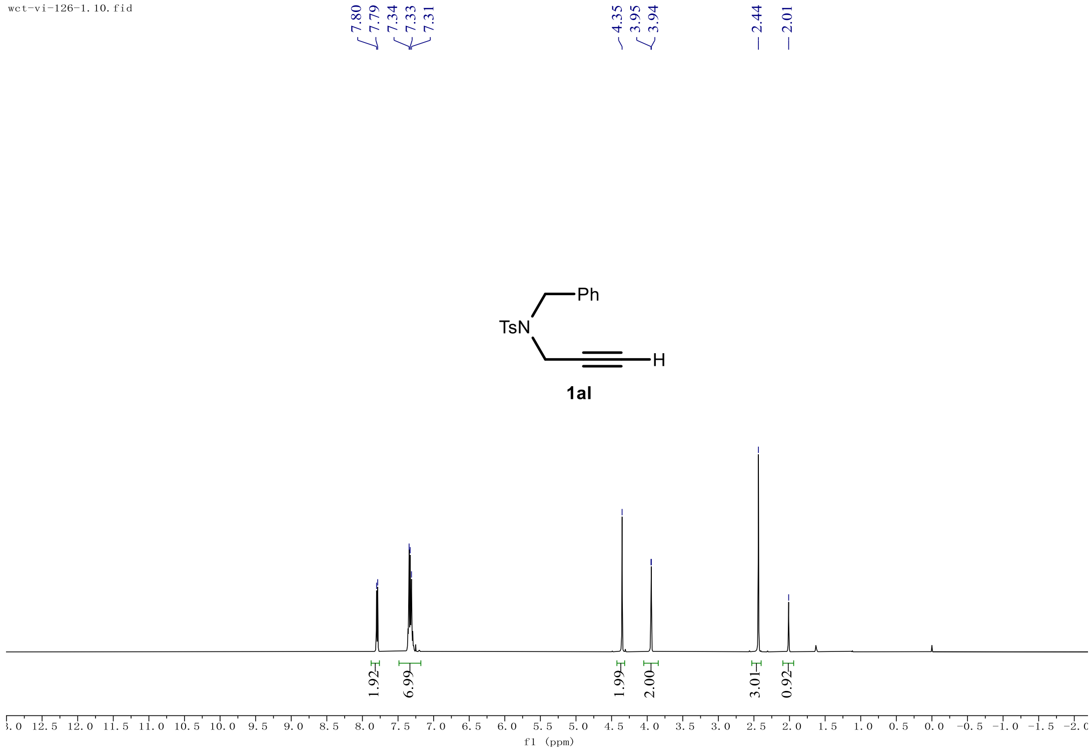
wct-vi-126-1.10.fid
7.80
7.79
7.34
7.33
7.31
4.35
3.95
3.94
2.44
2.01
TsN
Ph
H
1al
1.92
6.99
1.99
2.00
3.01
0.92
f1 (ppm)

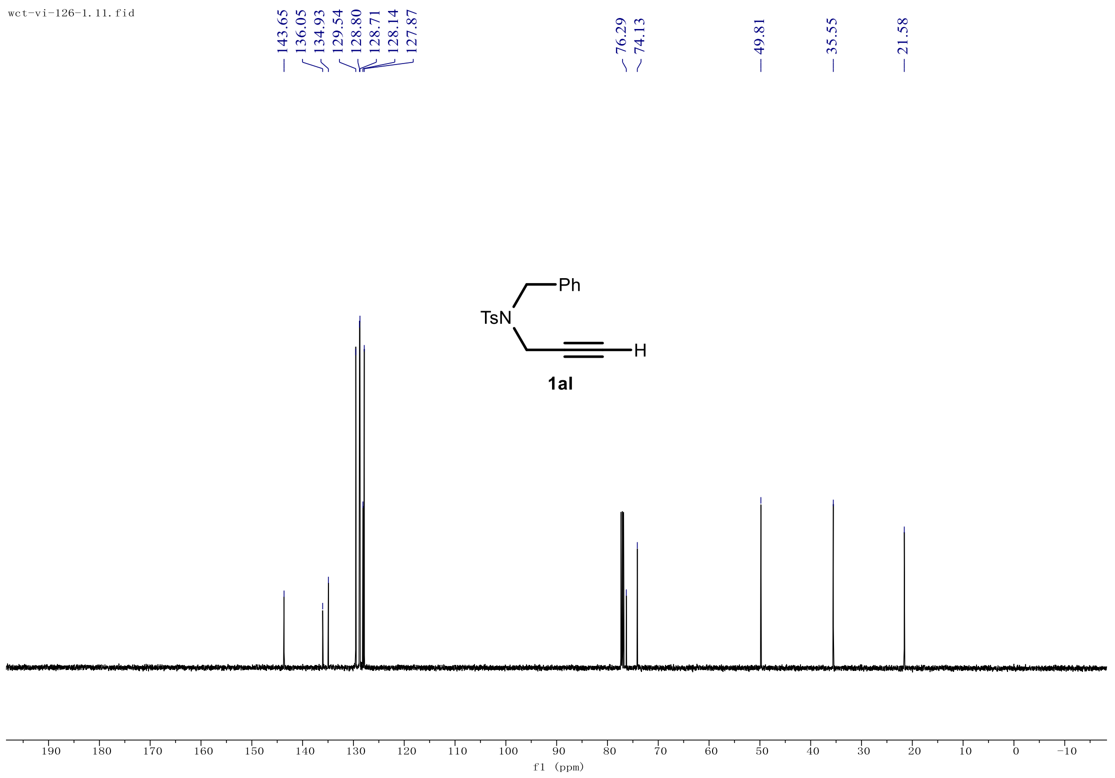

wct-vi-126-1.11.fid
143.65
136.05
134.93
129.54
128.80
128.71
128.14
127.87
76.29
74.13
49.81
35.55
21.58
Ph
TsN
H
1al
190 180 170 160 150 140 130 120 110 100 90 80 70 60 50 40 30 20 10 0 -10
f1 (ppm)

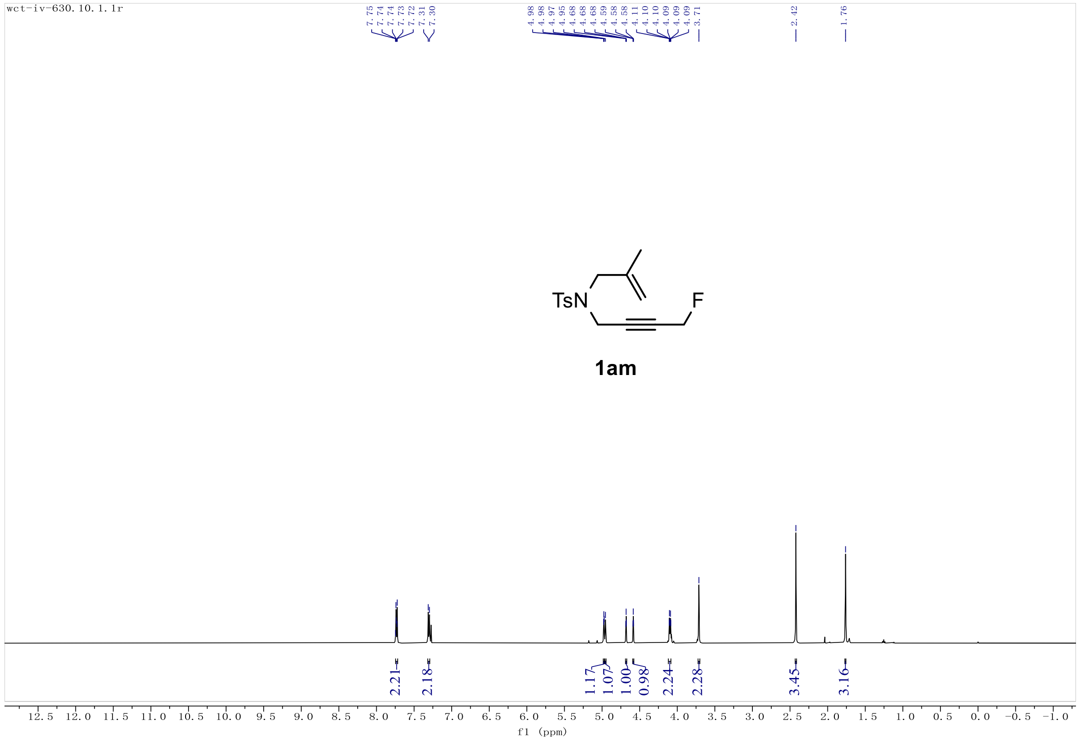

wct-iv-630.10.1.1r
7.75
7.74
7.74
7.73
7.72
7.31
7.30
4.98
4.98
4.97
4.95
4.68
4.68
4.59
4.58
4.58
4.11
4.10
4.10
4.09
4.09
4.09
3.71
2.42
1.76
TsN
F
1am
2.21
2.18
1.17
1.07
1.00
0.98
2.24
2.28
3.45
3.16
12.5 12.0 11.5 11.0 10.5 10.0 9.5 9.0 8.5 8.0 7.5 7.0 6.5 6.0 5.5 5.0 4.5 4.0 3.5 3.0 2.5 2.0 1.5 1.0 0.5 0.0 -0.5 -1.0
f1 (ppm)

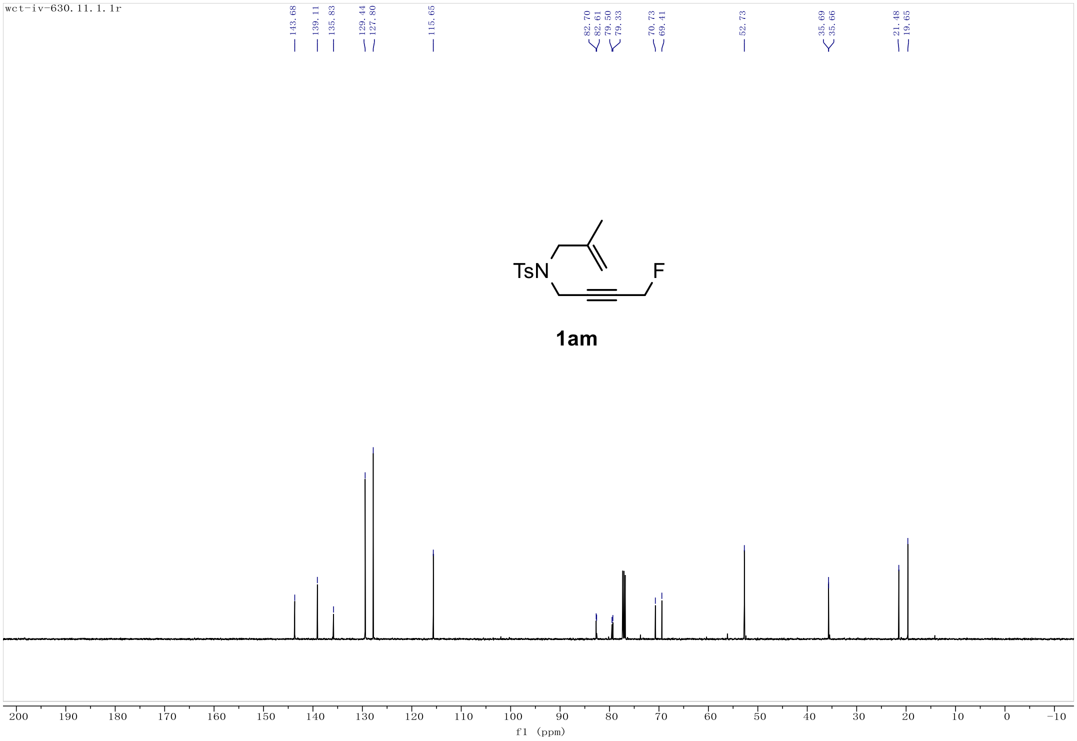
wct-iv-630.11.1.1r
143.68
139.11
135.83
129.44
127.80
115.65
82.70
82.61
79.50
79.33
70.73
69.41
52.73
35.69
35.66
21.48
19.65
TsN
F
1am
f1 (ppm)

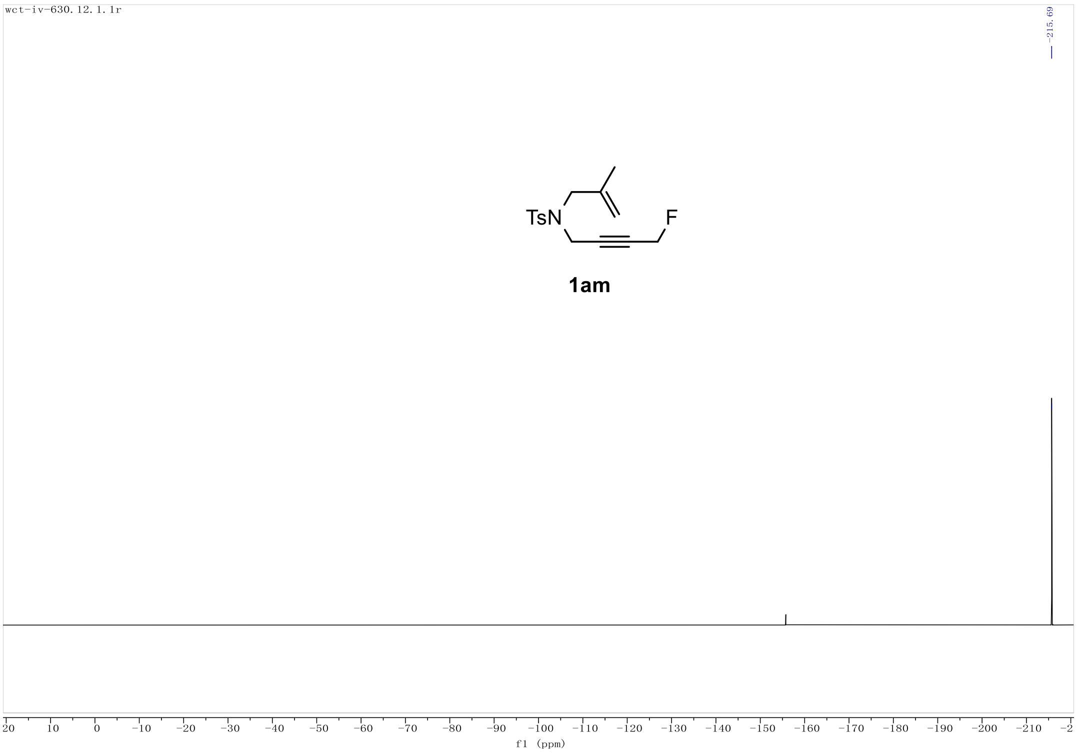
wct-iv-630.12.1.1r
-215.69
TsN
F
1am
f1 (ppm)

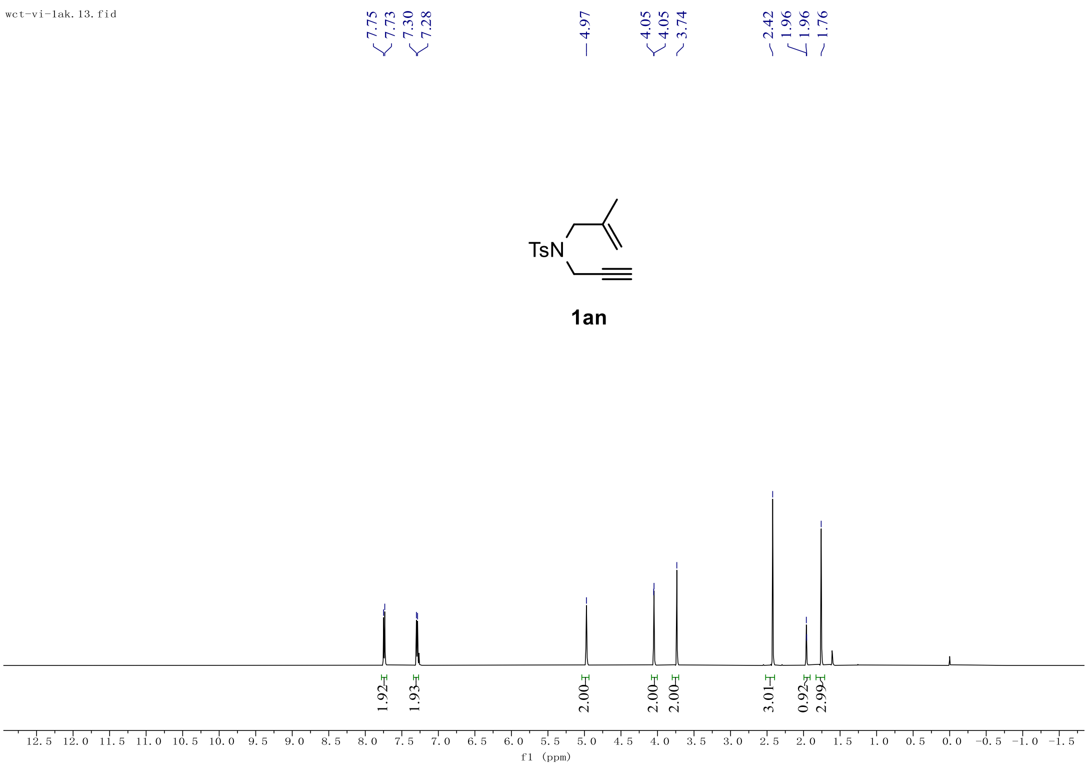
wct-vi-1ak.13.fid
7.75
7.73
7.30
7.28
4.97
4.05
4.05
3.74
2.42
1.96
1.96
1.76
TsN
1an
1.92
1.93
2.00
2.00
2.00
3.01
0.92
2.99
f1 (ppm)

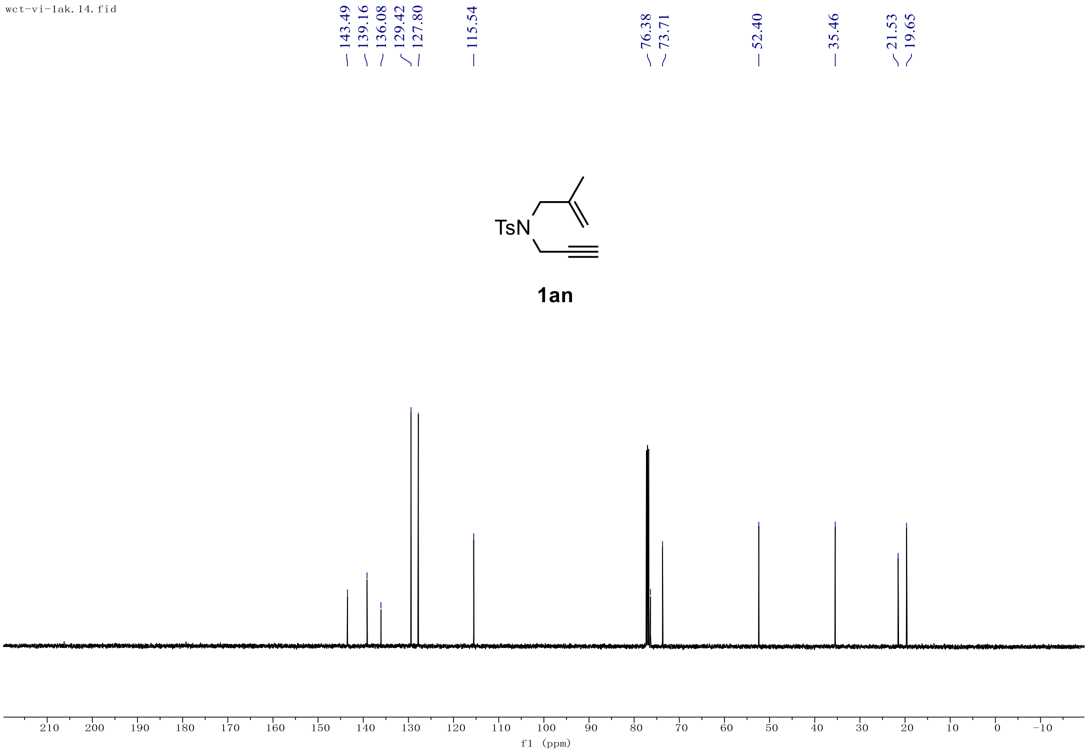
wct-vi-1ak.14.fid
143.49
139.16
136.08
129.42
127.80
115.54
76.38
73.71
52.40
35.46
21.53
19.65
TsN
1an
f1 (ppm)

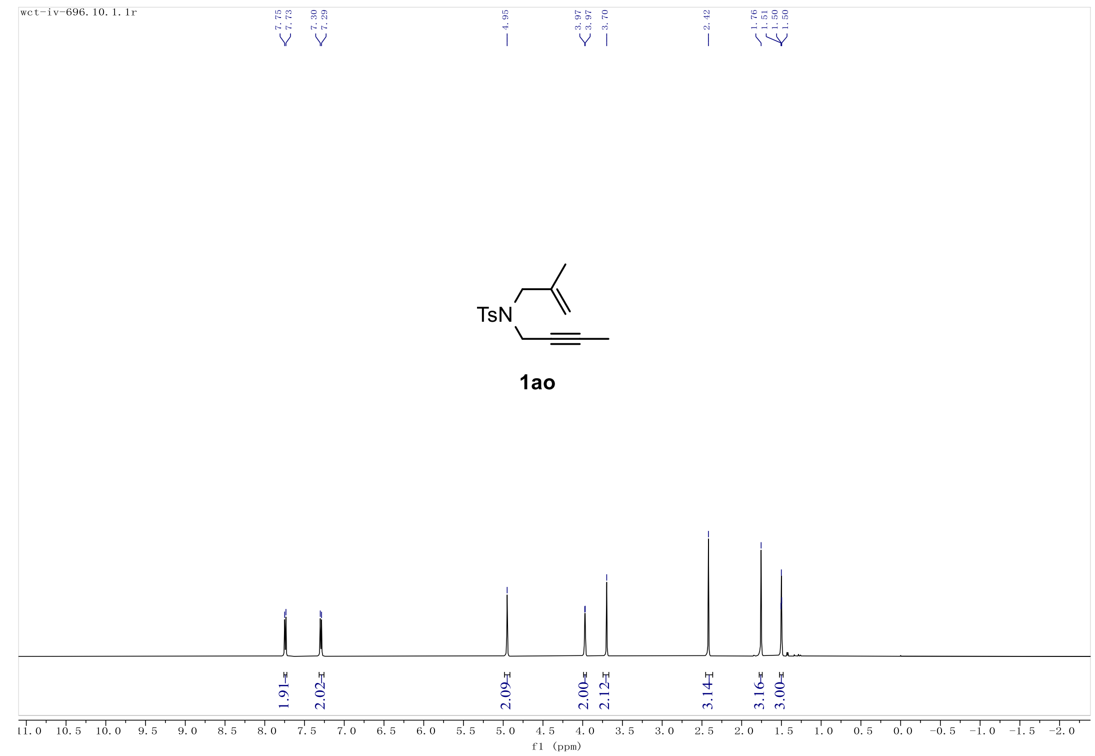
wct-iv-696.10.1.1r
7.75
7.73
7.30
7.29
4.95
3.97
3.97
3.70
2.42
1.76
1.51
1.50
1.50
TsN
1ao
1.91
2.02
2.09
2.00
2.12
3.14
3.16
3.00
f1 (ppm)

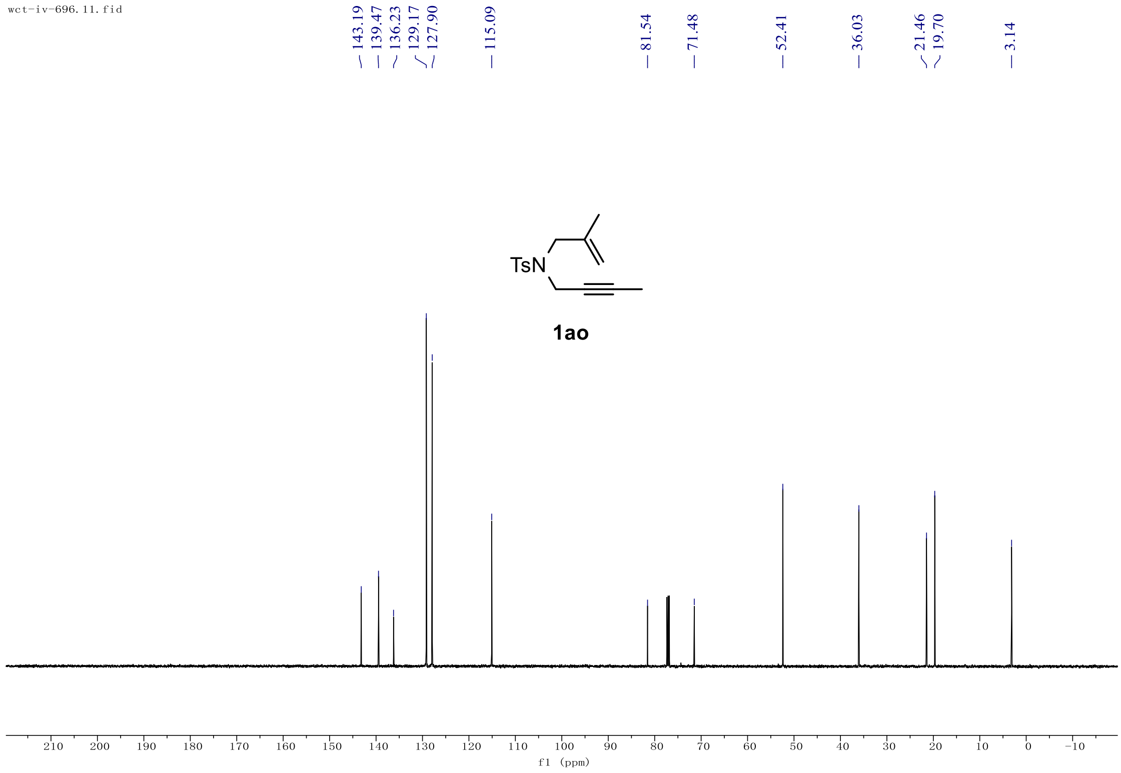
wct-iv-696.11.fid
143.19
139.47
136.23
129.17
127.90
115.09
81.54
71.48
52.41
36.03
21.46
19.70
3.14
TsN
1ao
f1 (ppm)

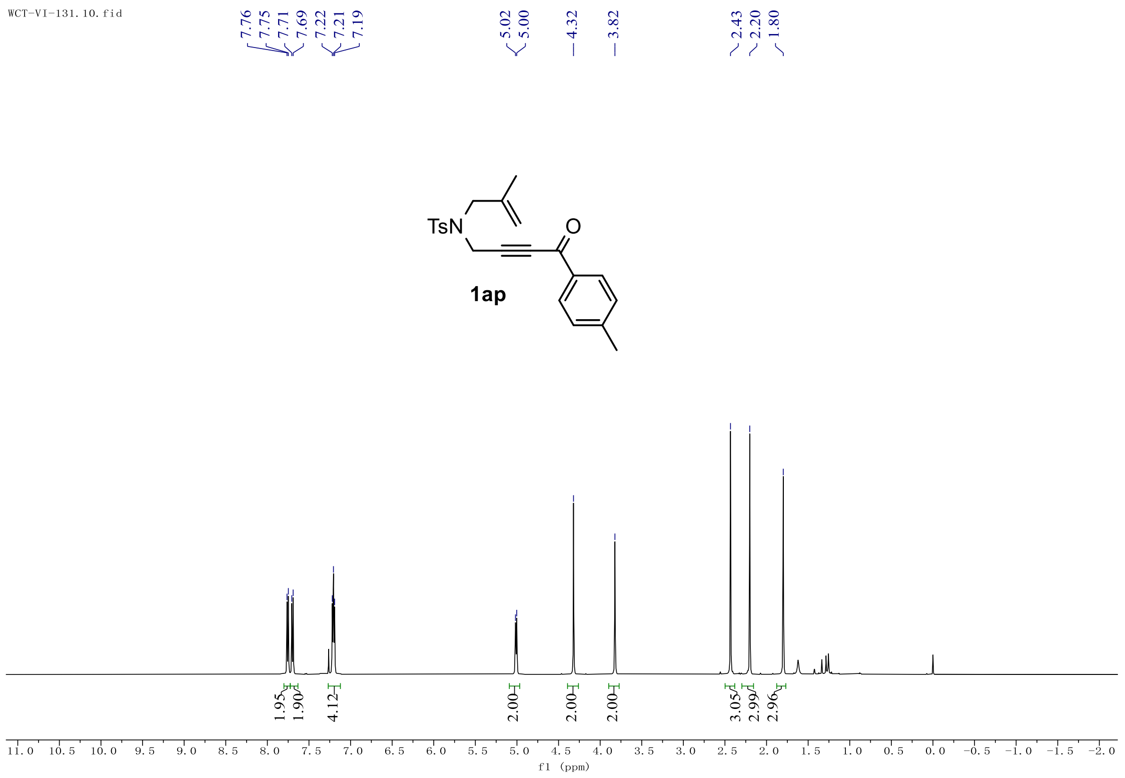
WCT-VI-131.10.fid
7.76
7.75
7.71
7.69
7.22
7.21
7.19
5.02
5.00
4.32
3.82
2.43
2.20
1.80
TsN
O
1ap
1.95
1.90
4.12
2.00
2.00
2.00
3.05
2.99
2.96
f1 (ppm)

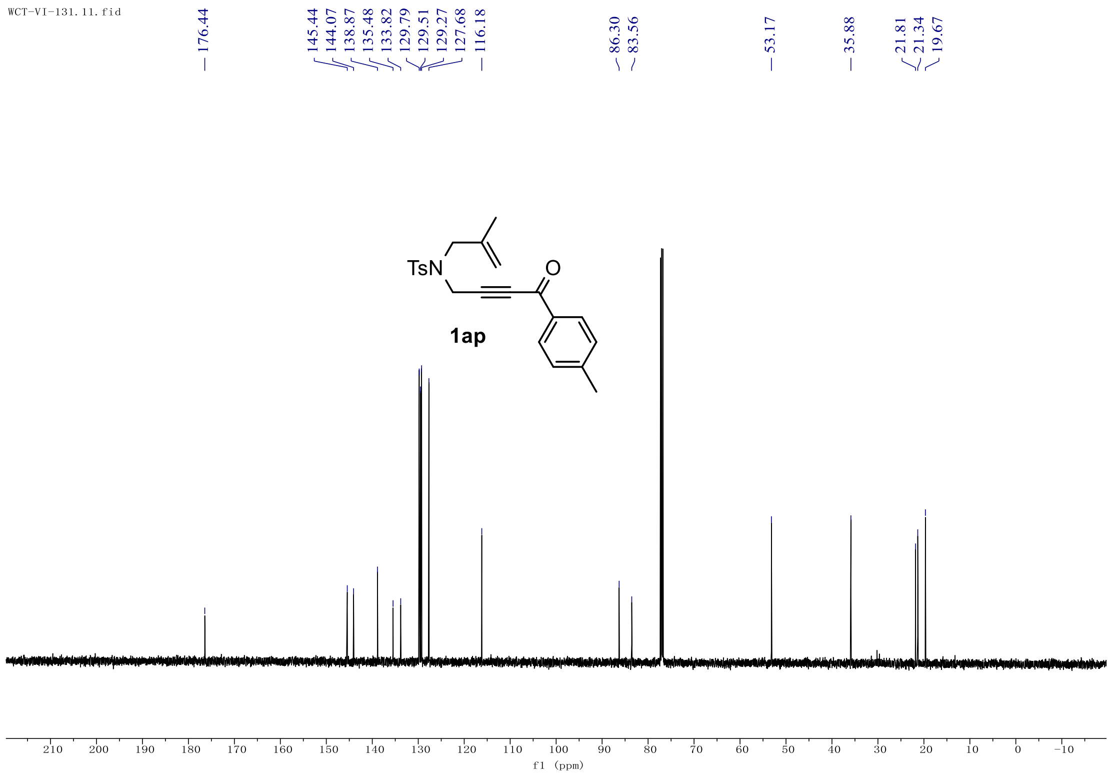
WCT-VI-131.11.fid
176.44
145.44
144.07
138.87
135.48
133.82
129.79
129.51
129.27
127.68
116.18
86.30
83.56
53.17
35.88
21.81
21.34
19.67
TsN
O
1ap
f1 (ppm)

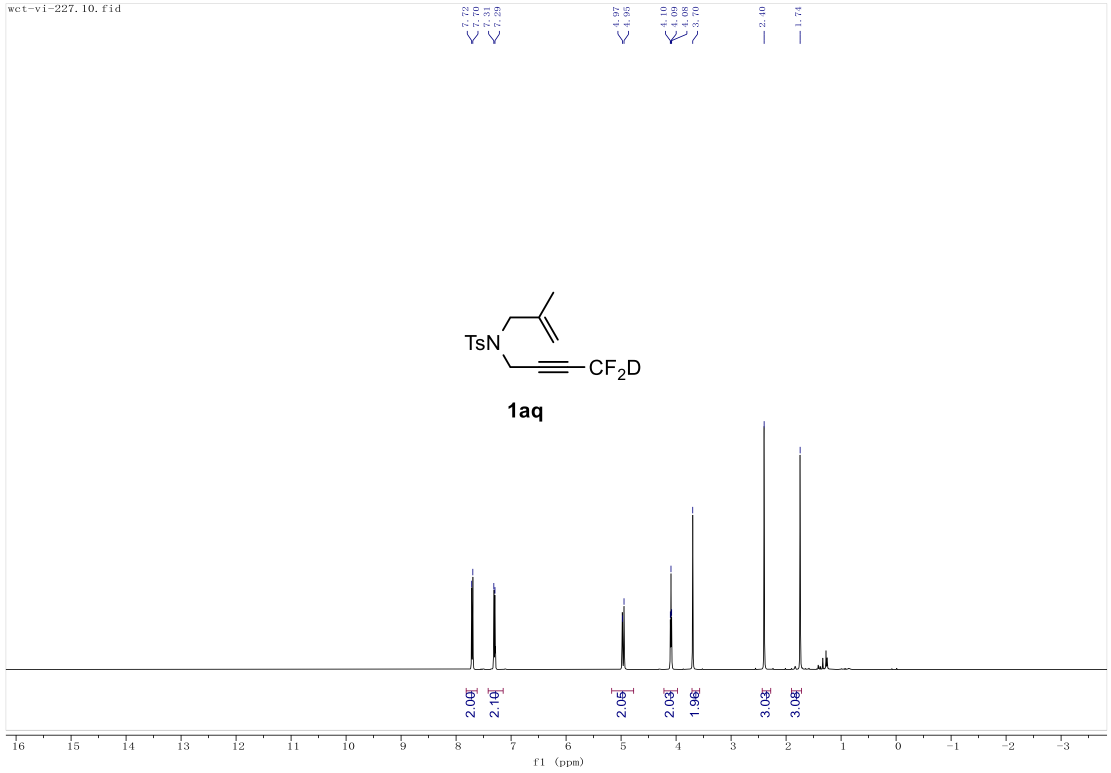
wct-vi-227.10.fid
7.72
7.70
7.31
7.29
4.97
4.95
4.10
4.09
4.08
3.70
2.40
1.74
TsN
CF2D
1aq
2.00
2.10
2.05
2.03
1.96
3.03
3.08
f1 (ppm)

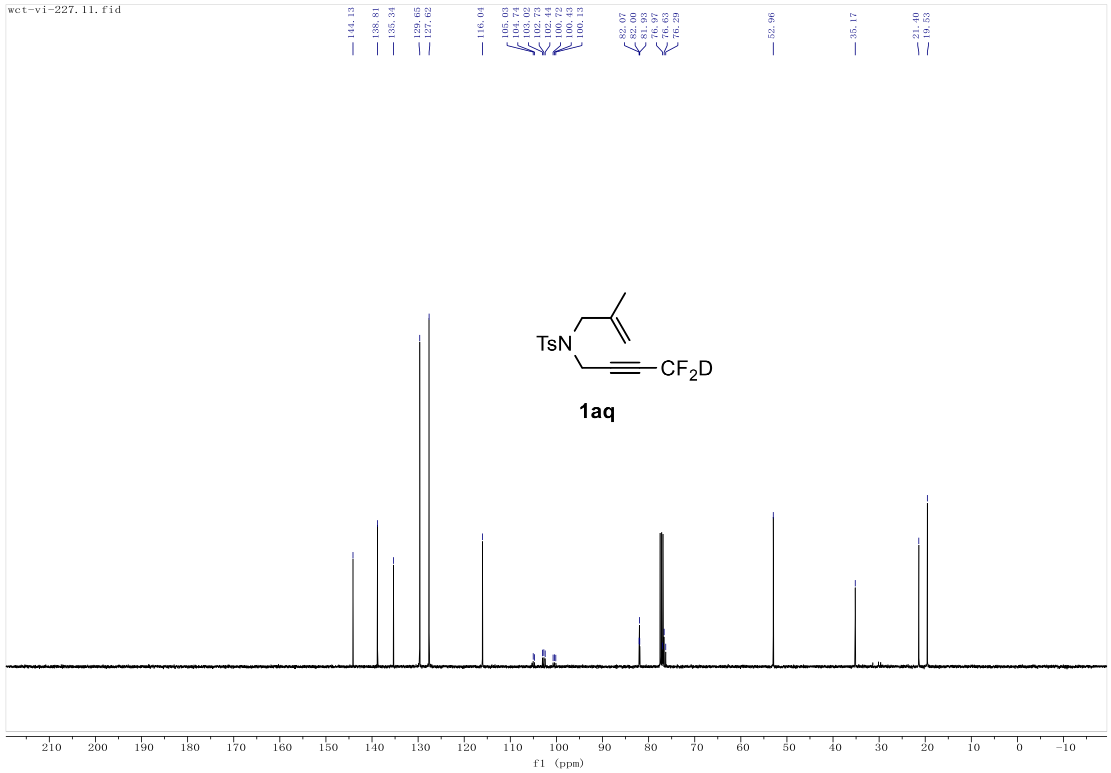
wct-vi-227.11.fid
144.13
138.81
135.34
129.65
127.62
116.04
105.03
104.74
103.02
102.73
102.44
100.72
100.43
100.13
82.07
82.00
81.93
76.97
76.63
76.29
52.96
35.17
21.40
19.53
TsN
CF2D
1aq
f1 (ppm)

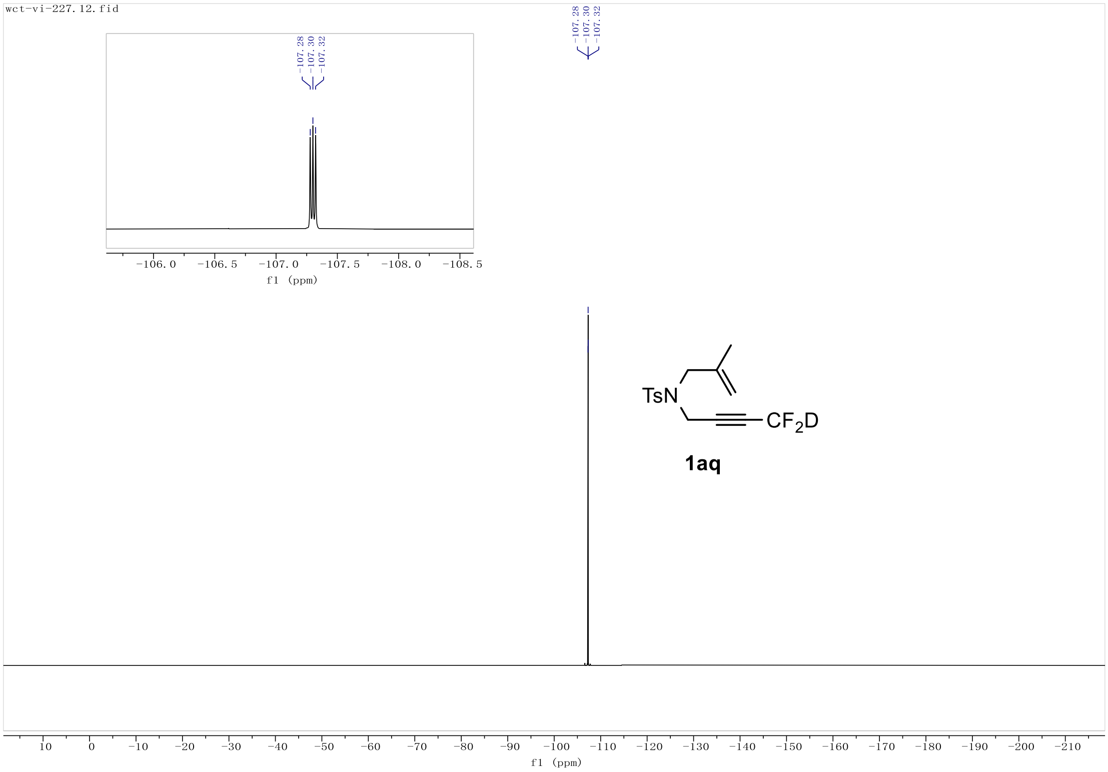

wct-vi-227.12.fid
-107.28
-107.30
-107.32
f1 (ppm)
TsN
CF2D
1aq

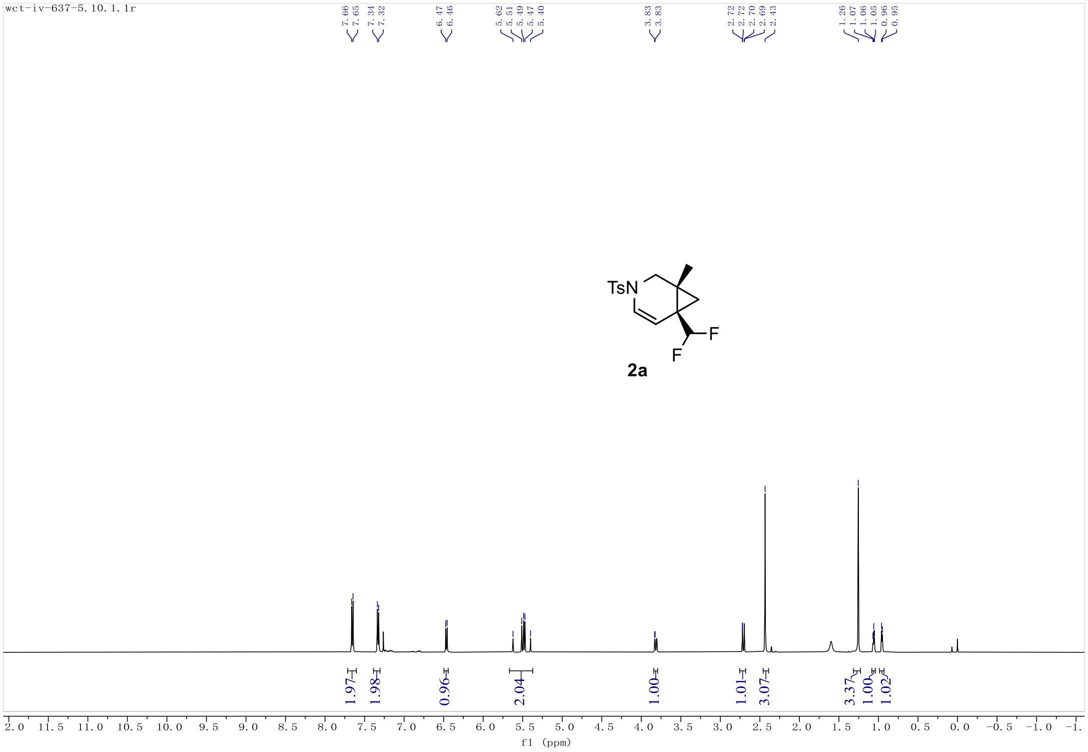

wct-iv-637-5.10.1.1r
7.66
7.65
7.34
7.32
6.47
6.46
5.62
5.51
5.49
5.47
5.40
3.83
3.83
2.72
2.72
2.70
2.69
2.43
1.26
1.07
1.06
1.05
0.96
0.95
TsN
F
F
2a
1.97
1.98
0.96
2.04
1.00
1.01
3.07
3.37
1.00
1.02
f1 (ppm)

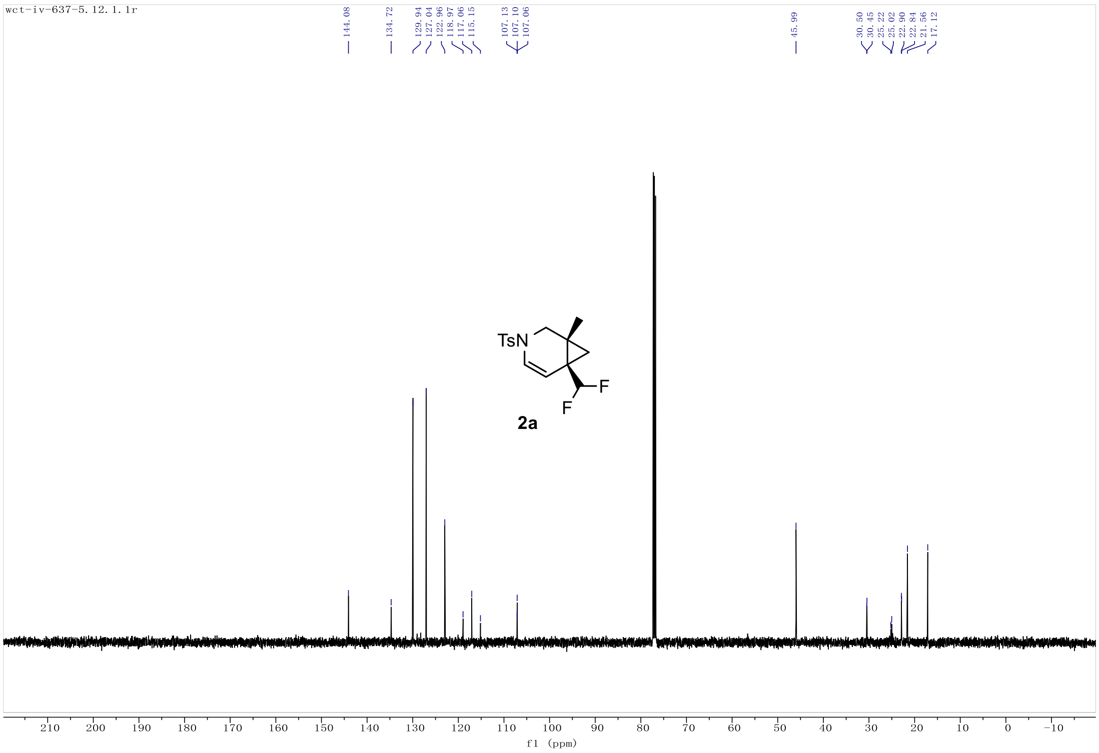
wct-iv-637-5.12.1.1r
144.08
134.72
129.94
127.04
122.96
118.97
117.06
115.15
107.13
107.10
107.06
45.99
30.50
30.45
25.22
25.02
22.90
22.84
21.56
17.12
TsN
F
F
2a
210 200 190 180 170 160 150 140 130 120 110 100 90 80 70 60 50 40 30 20 10 0 −10
f1 (ppm)

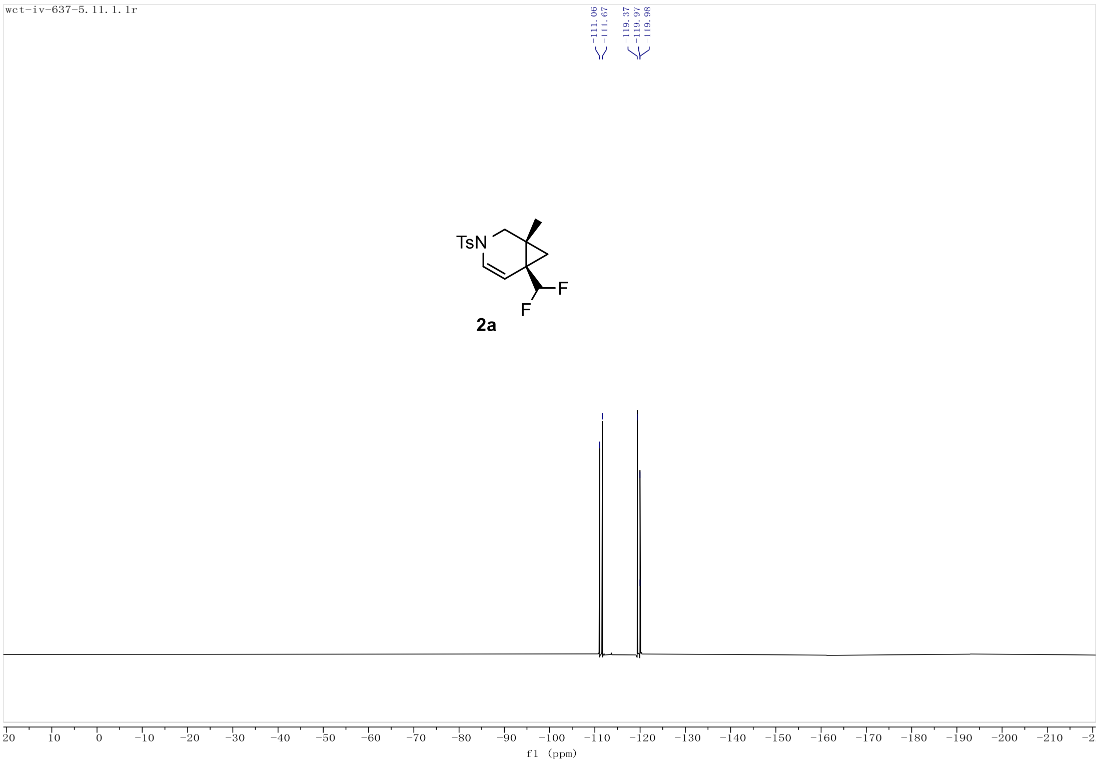
wct-iv-637-5.11.1.1r
-111.06
-111.67
-119.37
-119.97
-119.98
TsN
F
F
2a
20 10 0 −10 −20 −30 −40 −50 −60 −70 −80 −90 −100 −110 −120 −130 −140 −150 −160 −170 −180 −190 −200 −210
f1 (ppm)

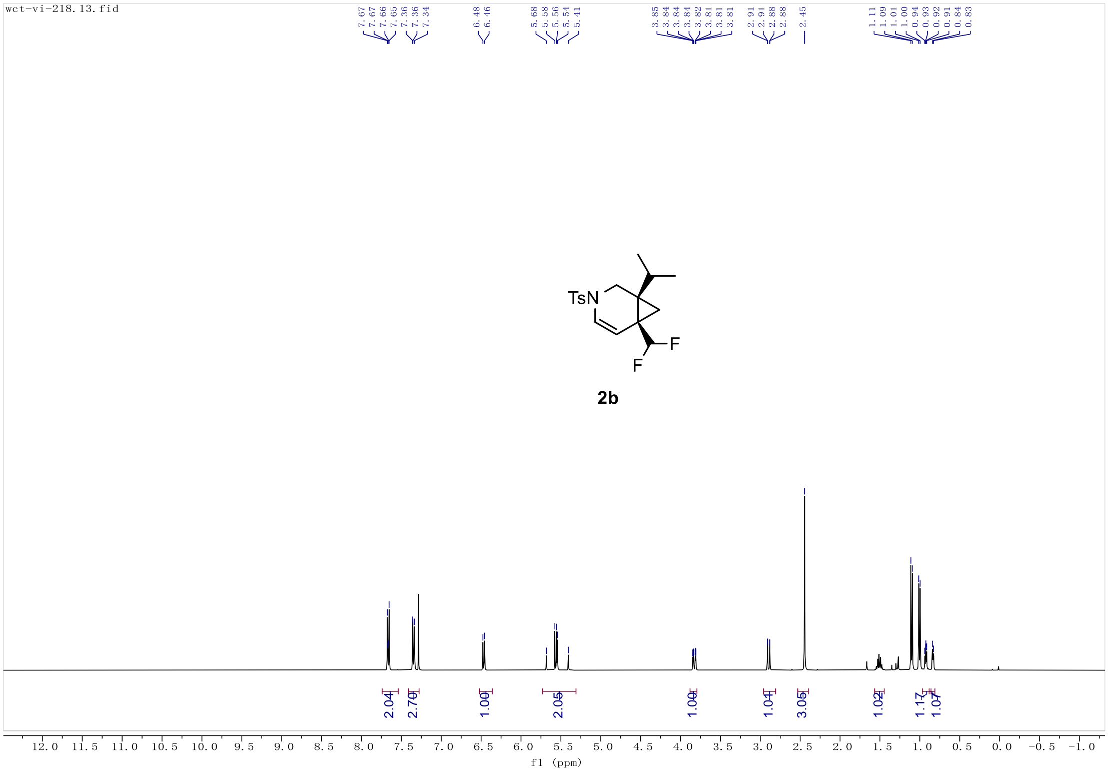

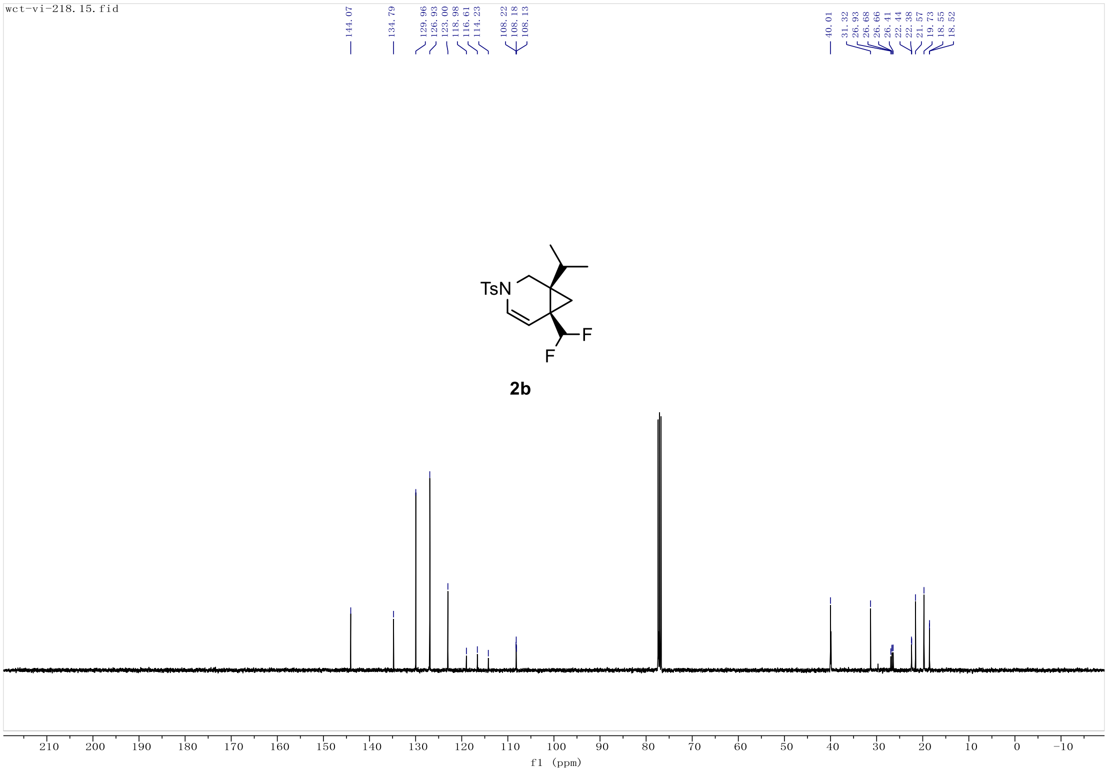

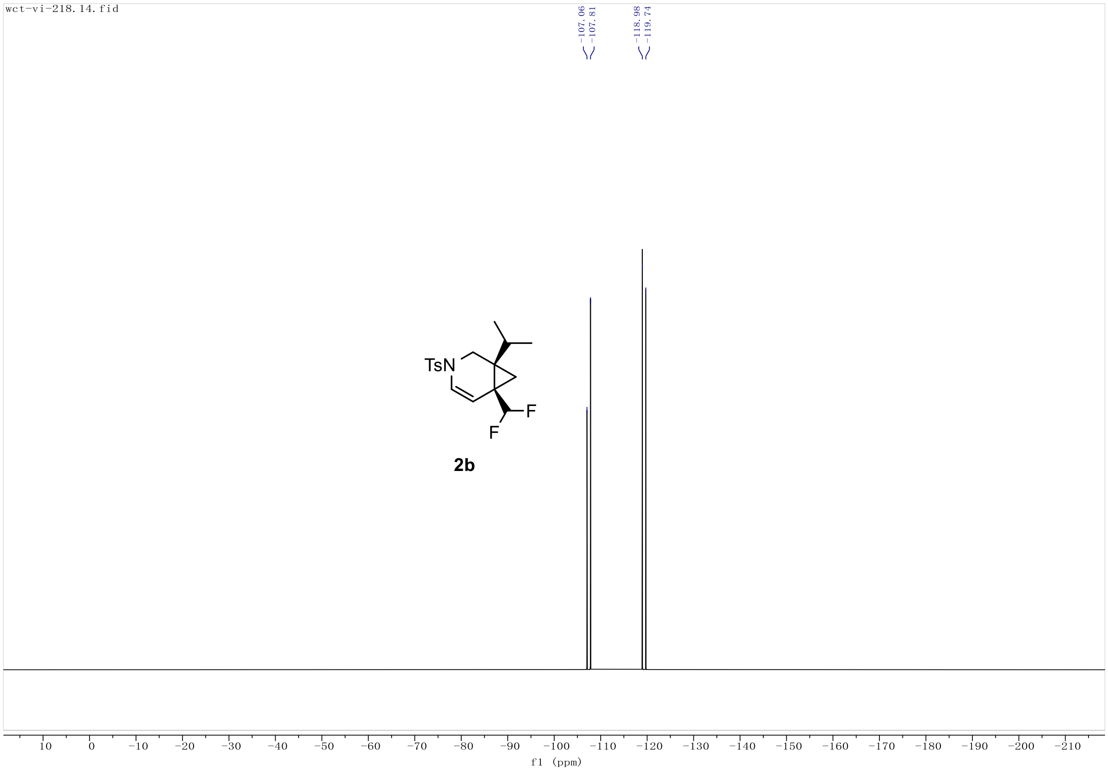
wct-vi-218.14.fid
-107.06
-107.81
-118.98
-119.74
TsN
F
F
2b
f1 (ppm)

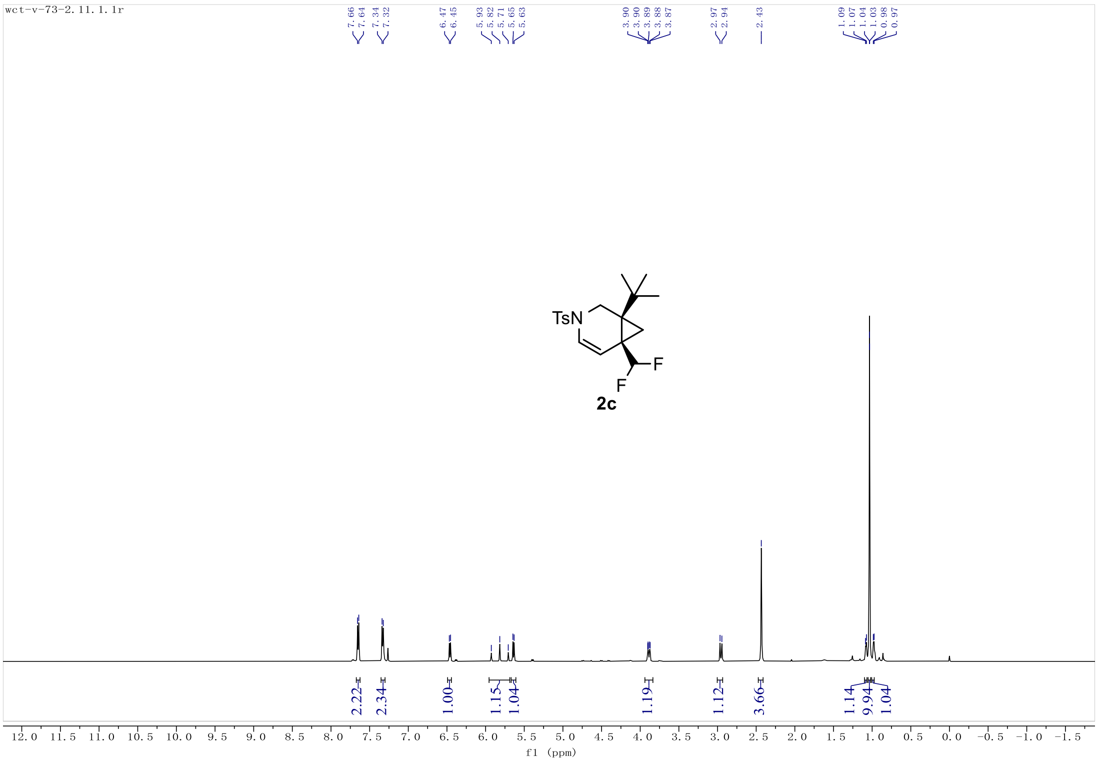
wct-v-73-2.11.1.1r
TsN
F
F
2c
f1 (ppm)

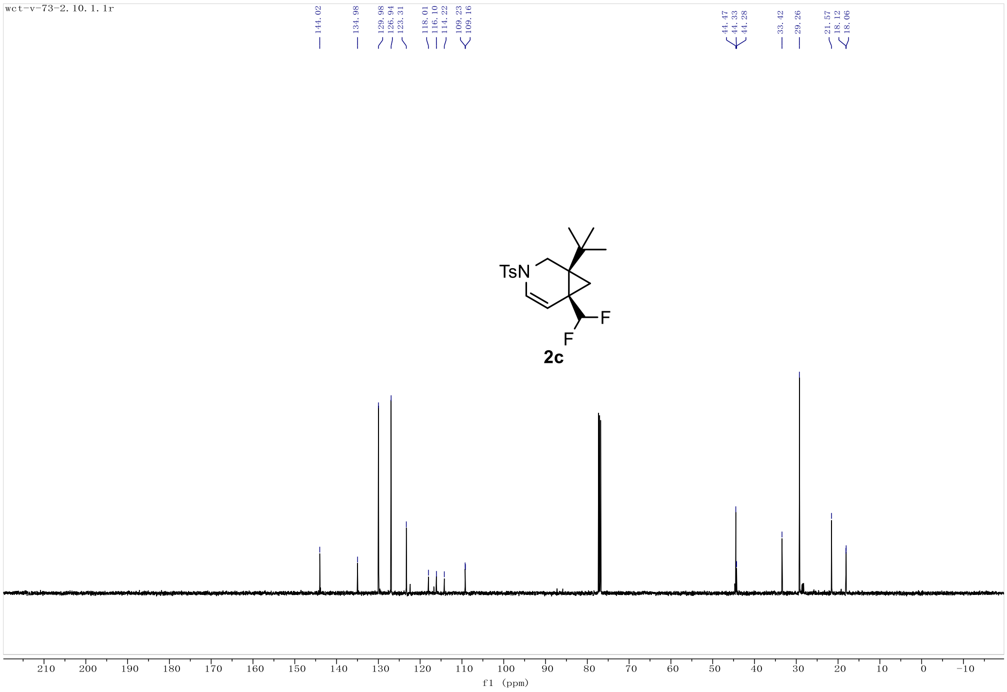
wct-v-73-2.10.1.1r
144.02
134.98
129.98
126.94
123.31
118.01
116.10
114.22
109.23
109.16
44.47
44.33
44.28
33.42
29.26
21.57
18.12
18.06
TsN
F
F
2c
f1 (ppm)

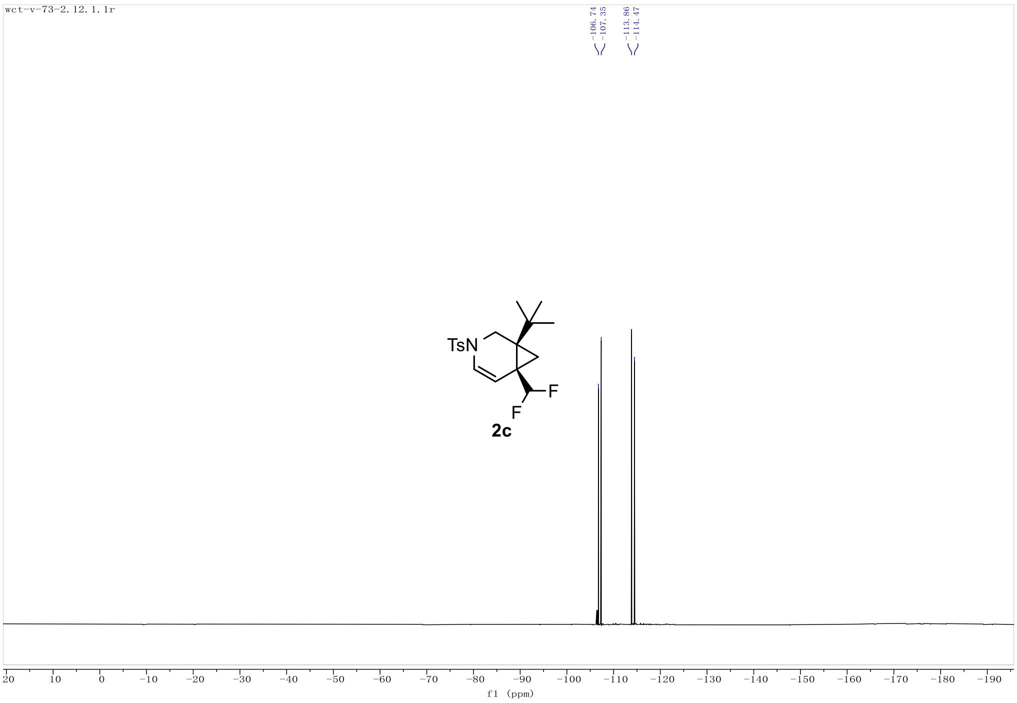
wct-v-73-2.12.1.1r
-106.74
-107.35
-113.86
-114.47
TsN
F
F
2c
f1 (ppm)

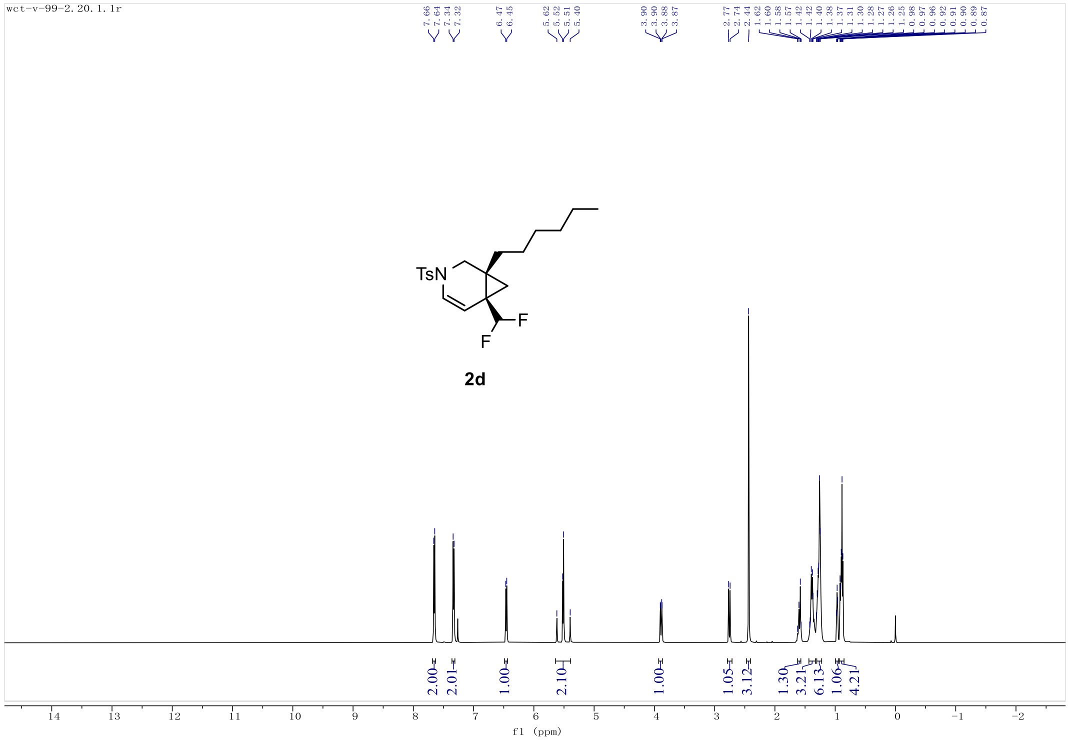
wct-v-99-2.20.1.1r
7.66 7.64 7.34 7.32 6.47 6.45 5.62 5.52 5.51 5.40 3.90 3.90 3.88 3.87 2.77 2.74 2.44 1.62 1.60 1.58 1.57 1.42 1.42 1.40 1.38 1.37 1.31 1.30 1.28 1.27 1.26 1.25 0.98 0.97 0.96 0.92 0.91 0.90 0.89 0.87
TsN
F
F
2d
2.00 2.01 1.00 2.10 1.00 1.05 3.12 1.30 3.21 6.13 1.06 4.21
f1 (ppm)

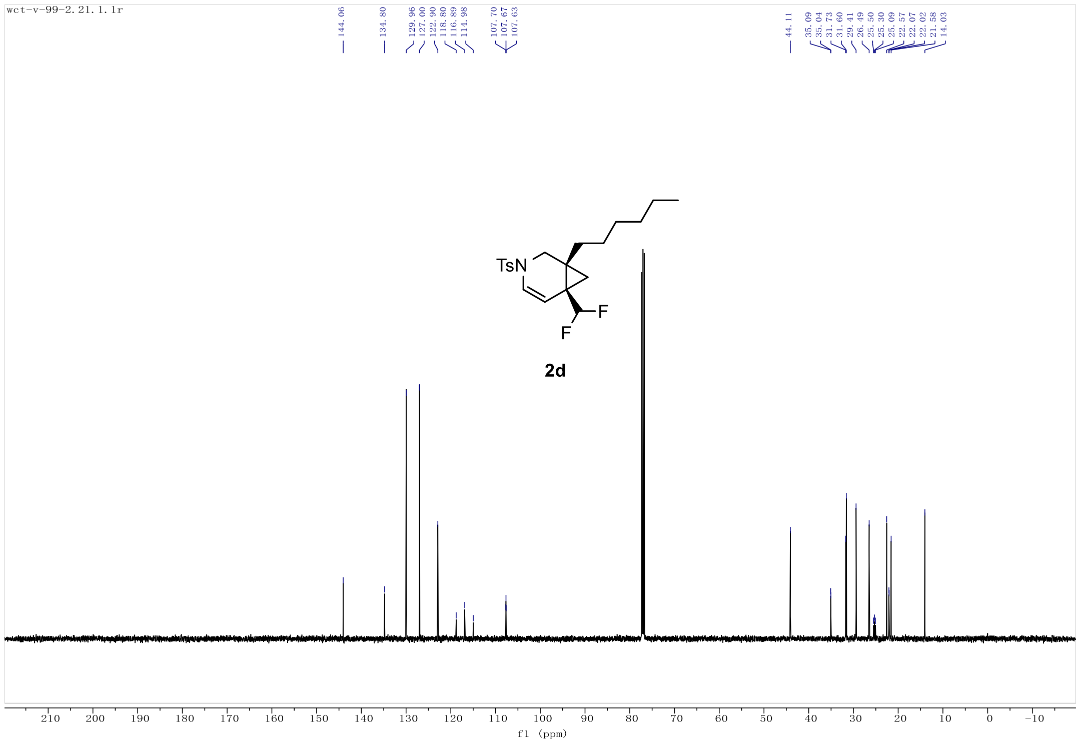
wct-v-99-2.21.1.1r
144.06 134.80 129.96 127.00 122.90 118.80 116.89 114.98 107.70 107.67 107.63
44.11 35.09 35.04 31.73 31.60 29.41 26.49 25.50 25.30 25.09 22.57 22.07 22.02 21.58 14.03
TsN
F
F
2d
f1 (ppm)

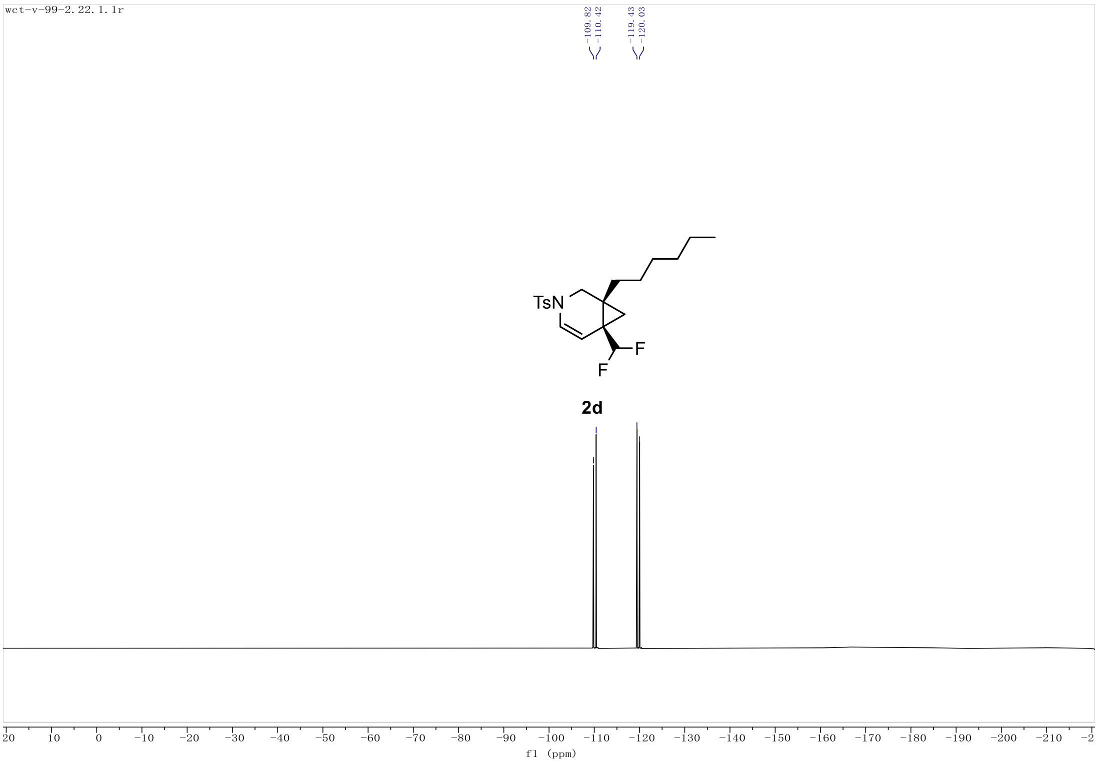
wct-v-99-2.22.1.1r
-109.82
-110.42
-119.43
-120.03
TsN
F
F
2d
f1 (ppm)

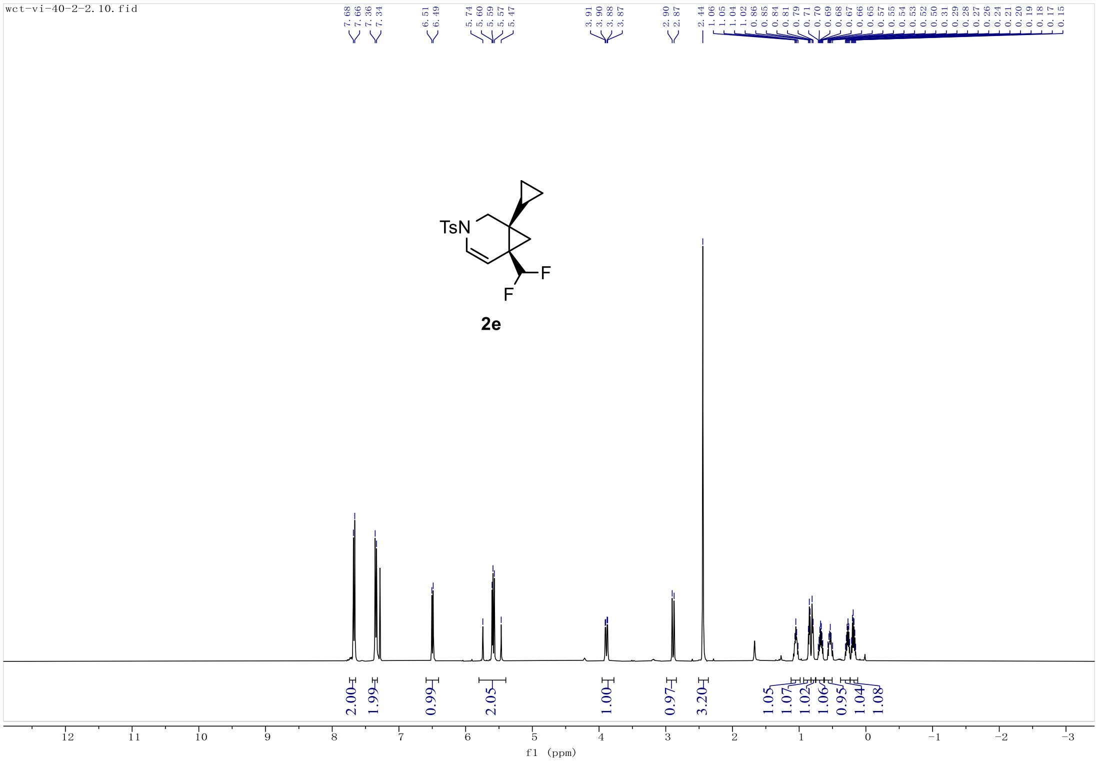
wct-vi-40-2-2.10.fid
TsN
F
F
2e
2.00
1.99
0.99
2.05
1.00
0.97
3.20
1.05
1.07
1.02
1.06
0.95
1.04
1.08
f1 (ppm)

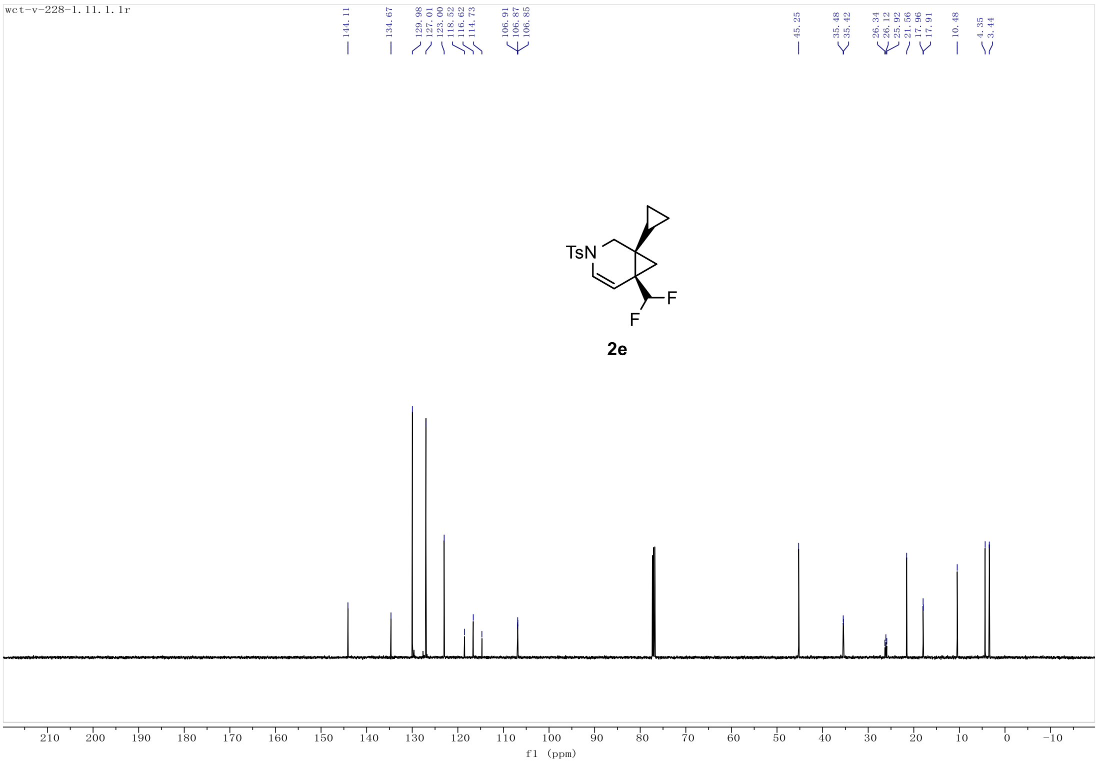
wct-v-228-1.11.1.1r
144.11
134.67
129.98
127.01
123.00
118.52
116.62
114.73
106.91
106.87
106.85
45.25
35.48
35.42
26.34
26.12
25.92
21.56
17.96
17.91
10.48
4.35
3.44
TsN
F
F
2e
f1 (ppm)

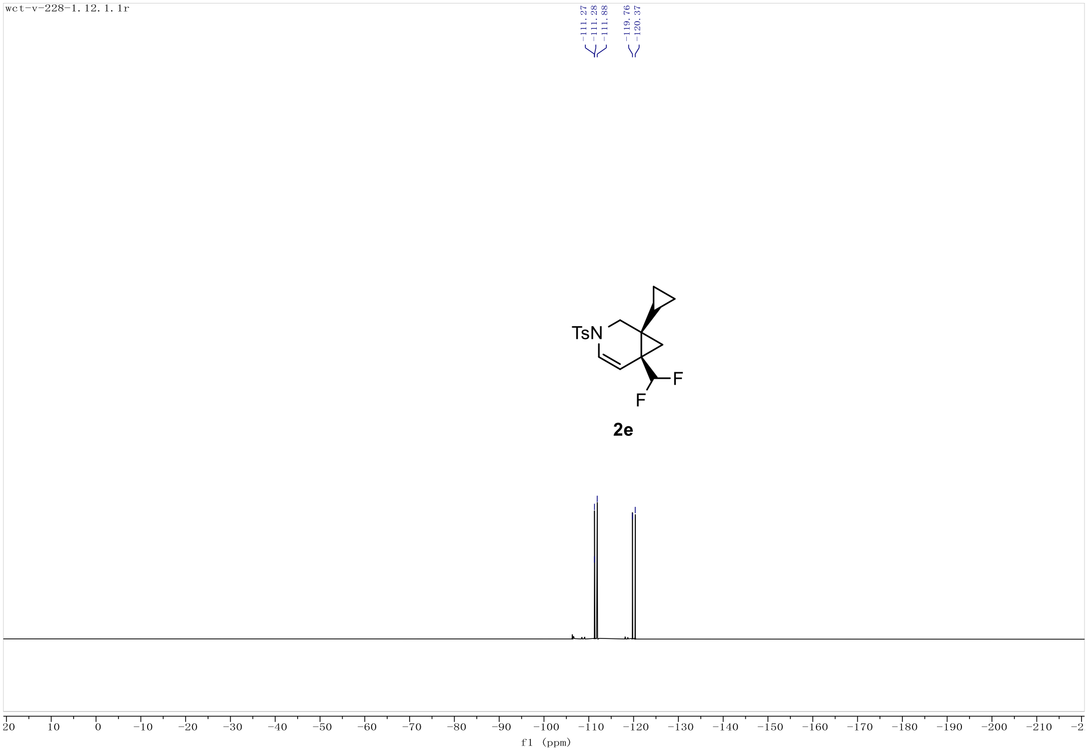
wct-v-228-1.12.1.1r
-111.27
-111.28
-111.88
-119.76
-120.37
TsN
F
F
2e
f1 (ppm)

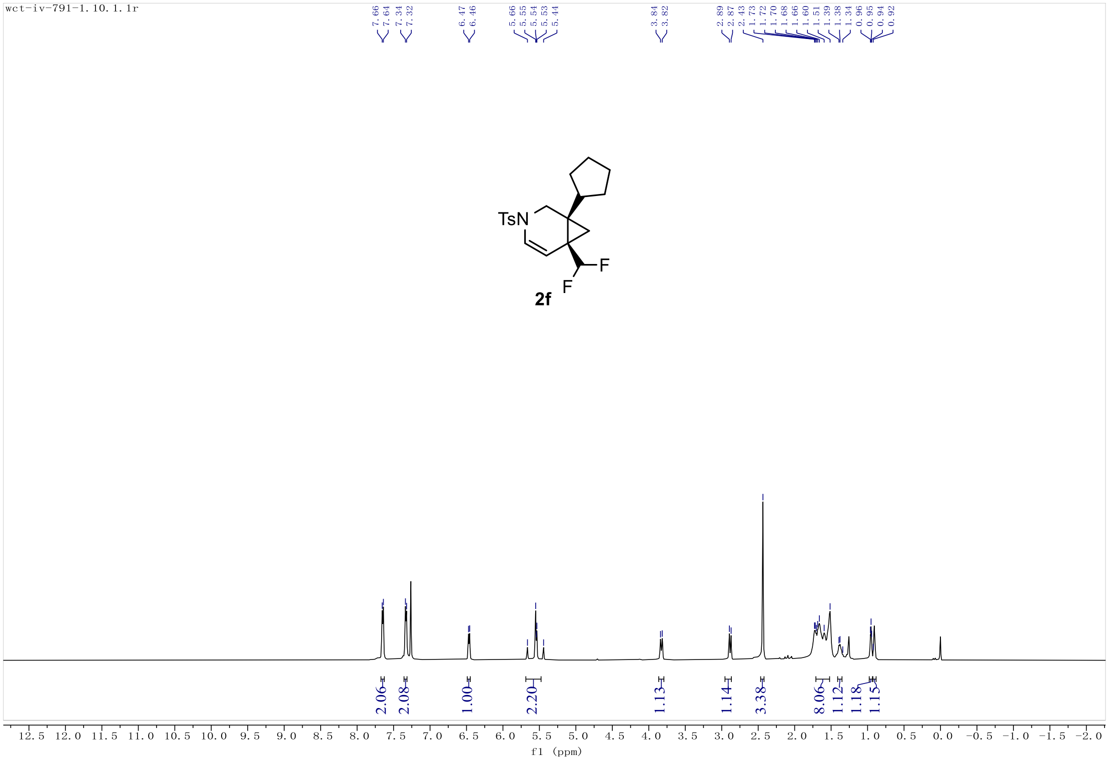
wct-iv-791-1.10.1.1r
7.66 7.64 7.34 7.32
6.47 6.46
5.66 5.55 5.54 5.53 5.44
3.84 3.82
2.89 2.87 2.43 1.73 1.72 1.70 1.68 1.66 1.60 1.51 1.39 1.38 1.34 0.96 0.95 0.94 0.92
TsN
F
F
2f
2.06 2.08 1.00 2.20 1.13 1.14 3.38 8.06 1.12 1.18 1.15
f1 (ppm)

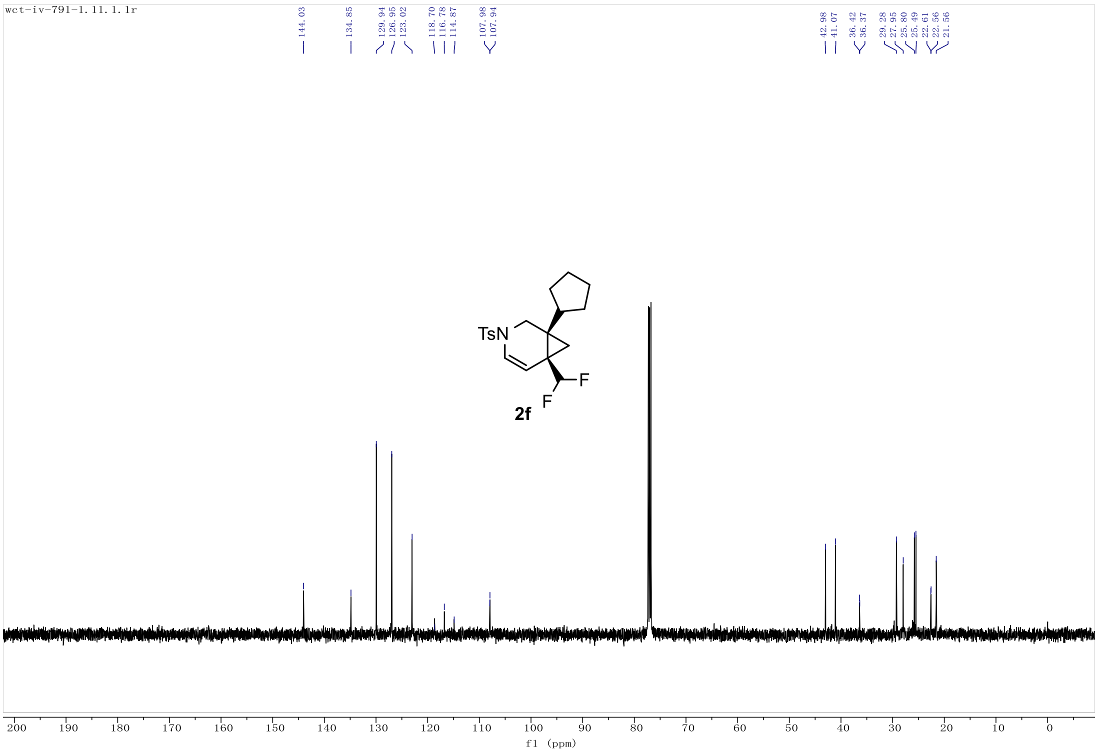
wct-iv-791-1.11.1.1r
144.03
134.85
129.94 126.95 123.02
118.70 116.78 114.87
107.98 107.94
42.98 41.07
36.42 36.37
29.28 27.95 25.80 25.49 22.61 22.56 21.56
TsN
F
F
2f
f1 (ppm)

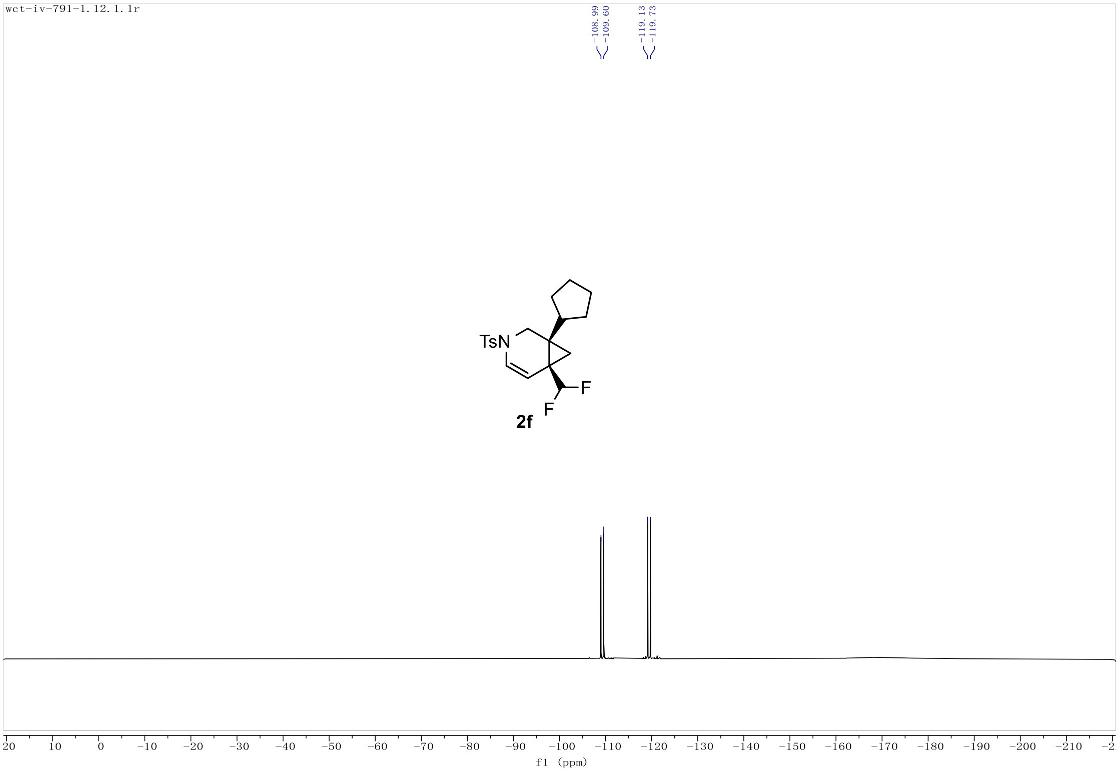
wct-iv-791-1.12.1.1r
-108.99
-109.60
-119.13
-119.73
TsN
F
F
2f
f1 (ppm)

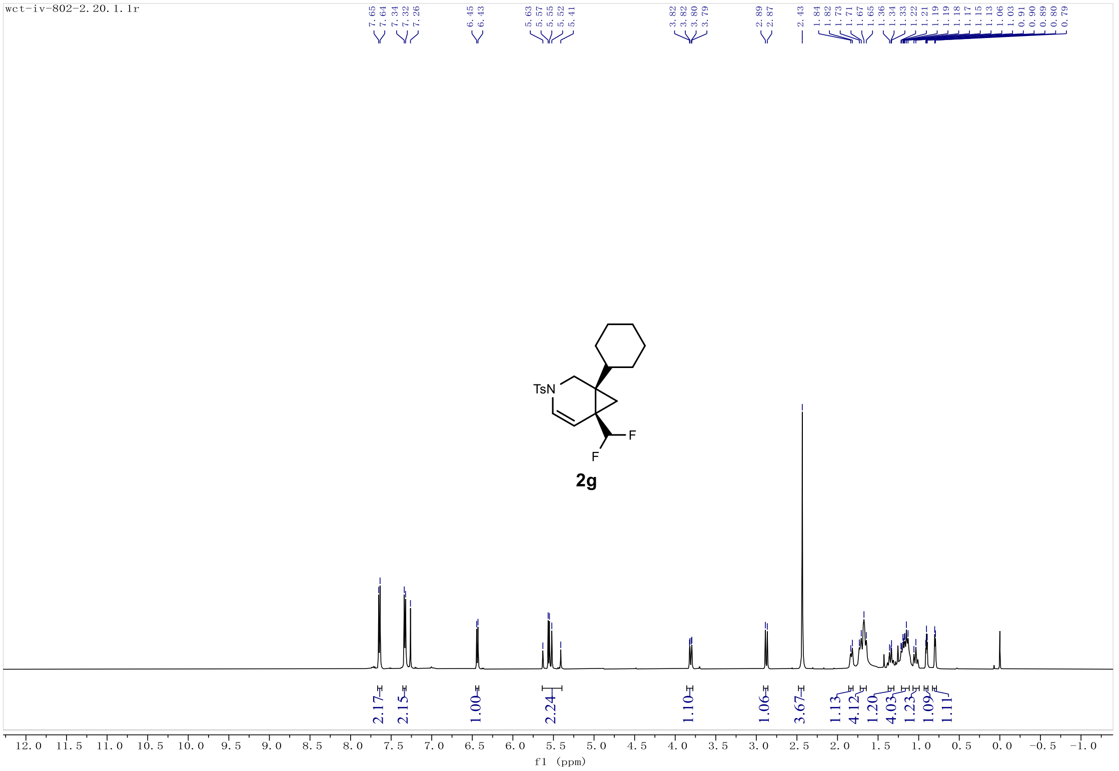
wct-iv-802-2.20.1.1r
TsN
F
F
2g
f1 (ppm)

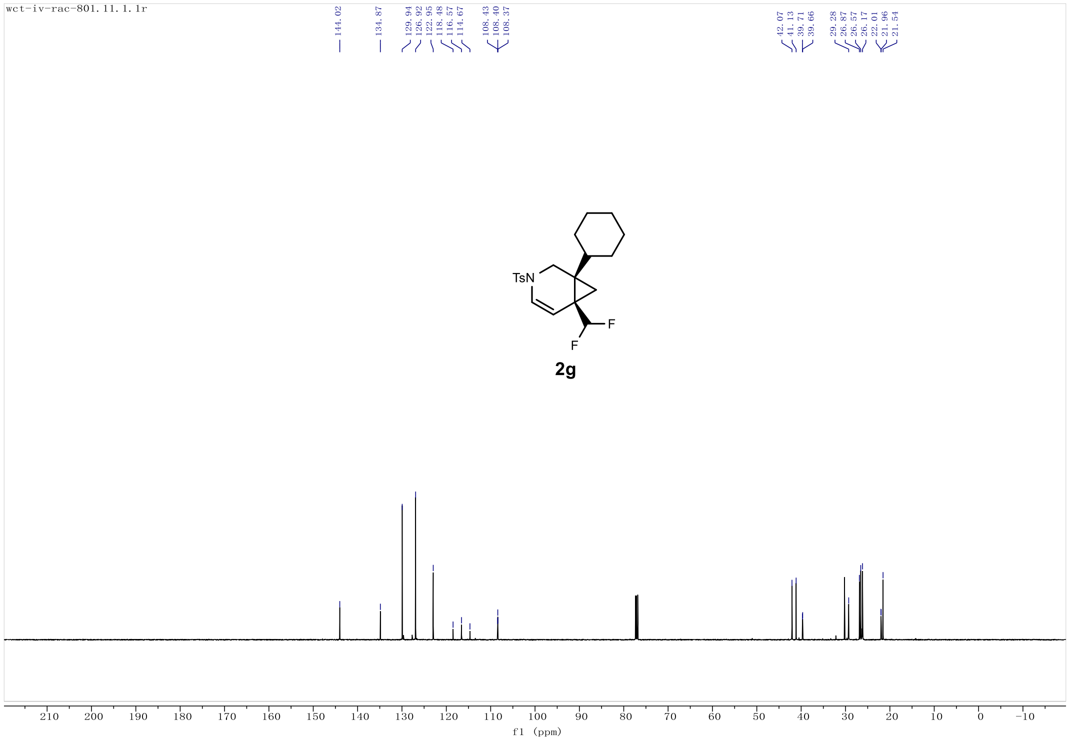
wct-iv-rac-801.11.1.1r
144.02
134.87
129.94
126.92
122.95
118.48
116.57
114.67
108.43
108.40
108.37
42.07
41.13
39.71
39.66
29.28
26.87
26.57
26.17
22.01
21.96
21.54
TsN
F
F
2g
210 200 190 180 170 160 150 140 130 120 110 100 90 80 70 60 50 40 30 20 10 0 −10
f1 (ppm)

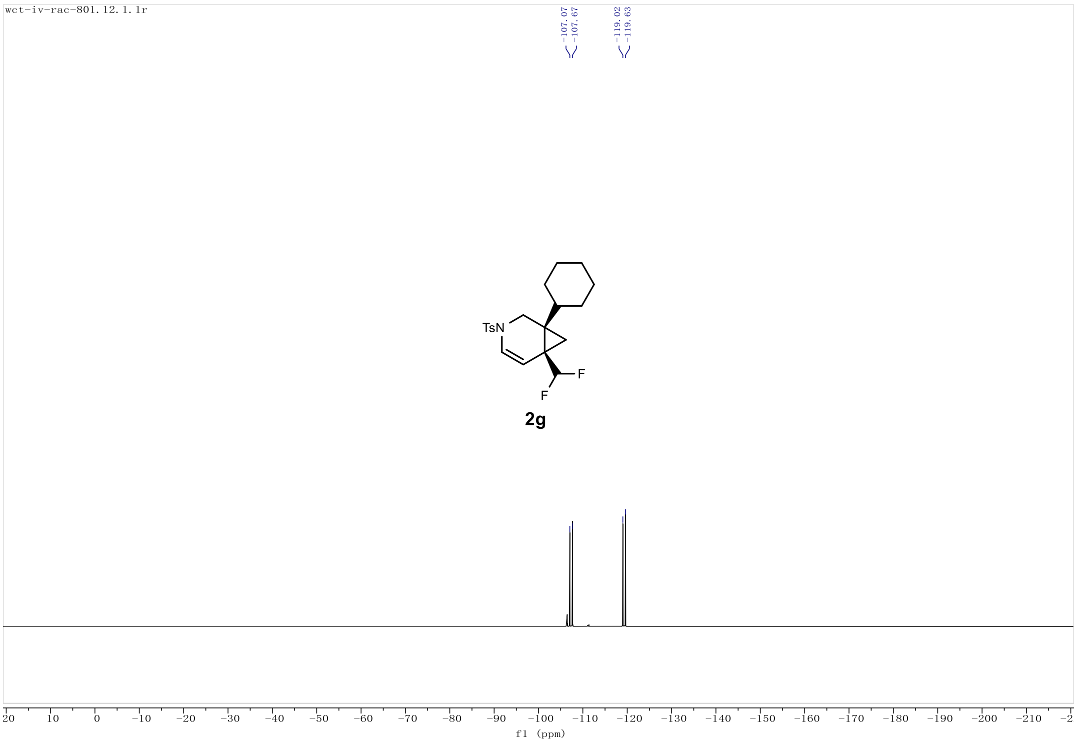
wct-iv-rac-801.12.1.1r
-107.07
-107.67
-119.02
-119.63
TsN
F
F
2g
20 10 0 −10 −20 −30 −40 −50 −60 −70 −80 −90 −100 −110 −120 −130 −140 −150 −160 −170 −180 −190 −200 −210
f1 (ppm)

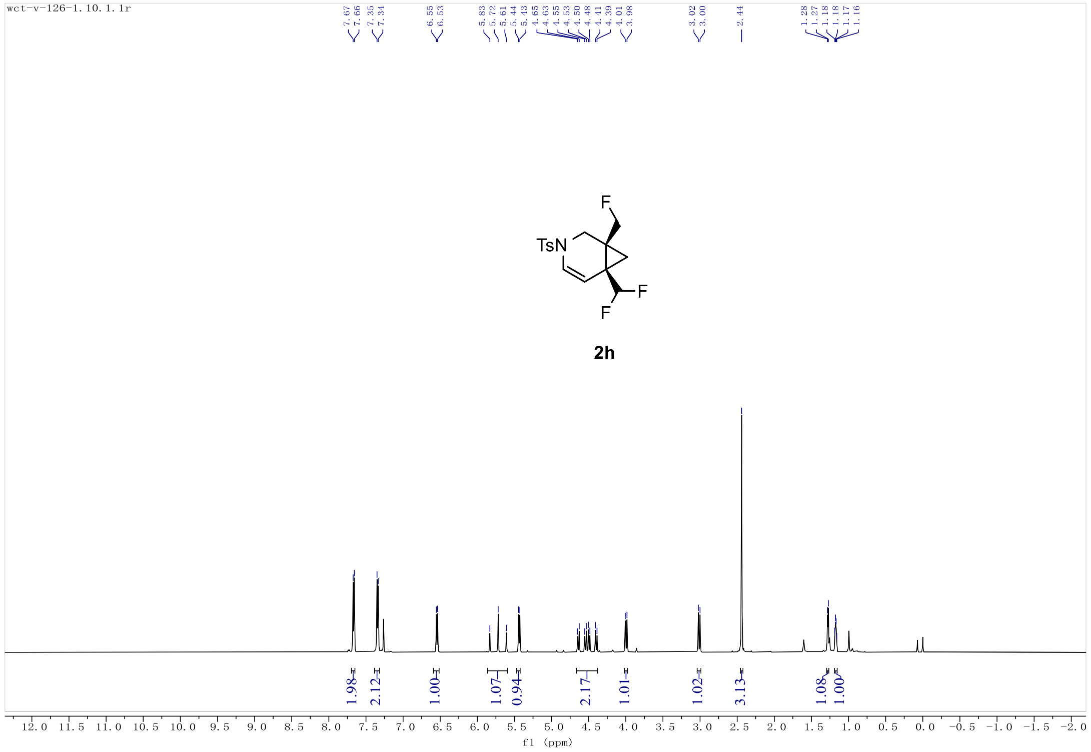
wct-v-126-1.10.1.1r
TsN
F
F
F
2h
f1 (ppm)

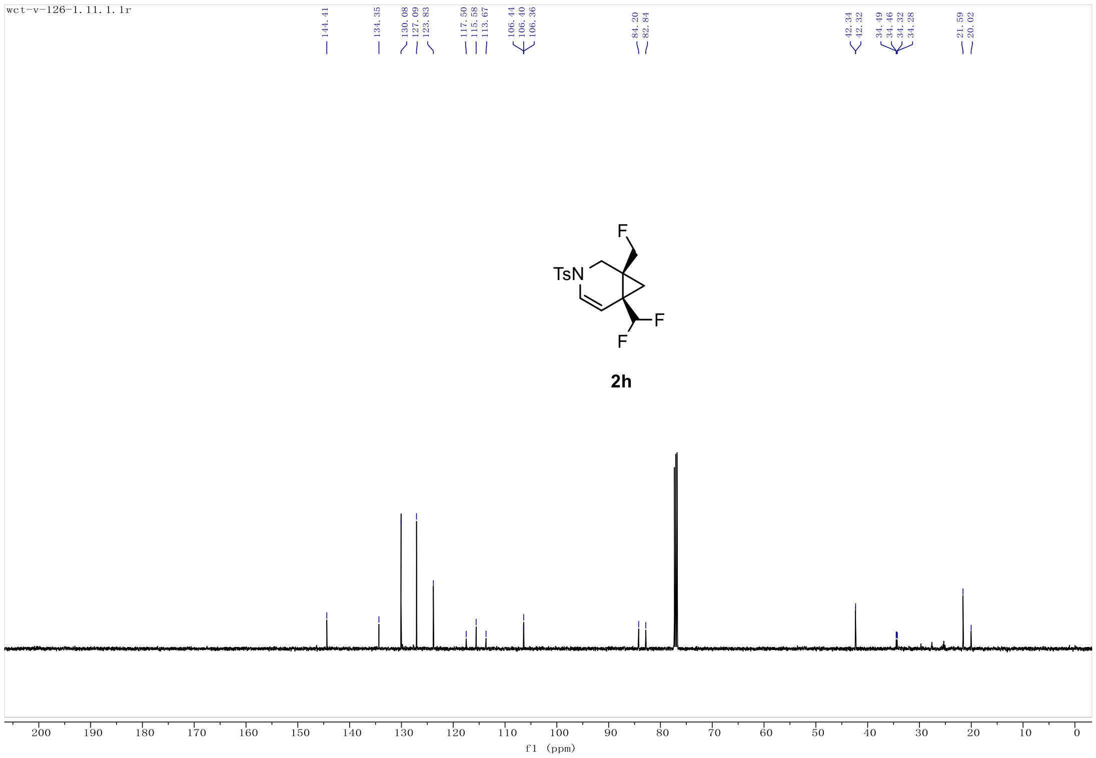
wct-v-126-1.11.1.1r
TsN
F
F
F
2h
f1 (ppm)

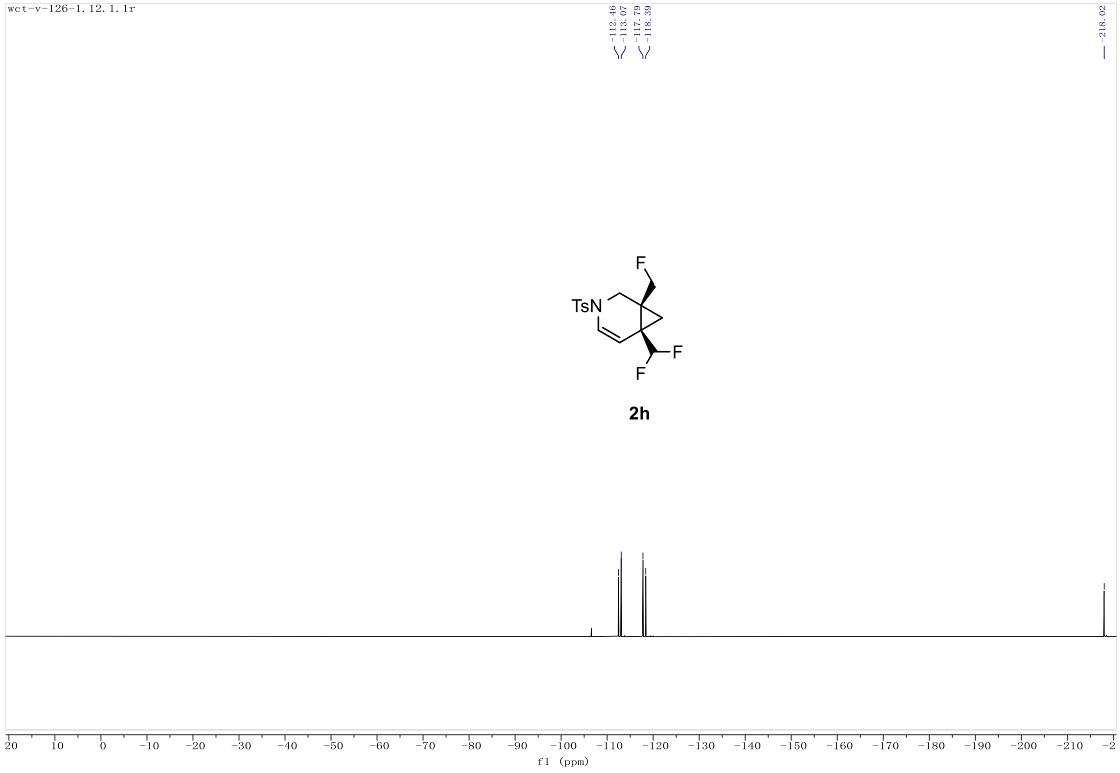
wct-v-126-1.12.1.1r
-112.46
-113.07
-117.79
-118.39
-218.02
TsN
F
F
F
2h
20 10 0 -10 -20 -30 -40 -50 -60 -70 -80 -90 -100 -110 -120 -130 -140 -150 -160 -170 -180 -190 -200 -210
f1 (ppm)

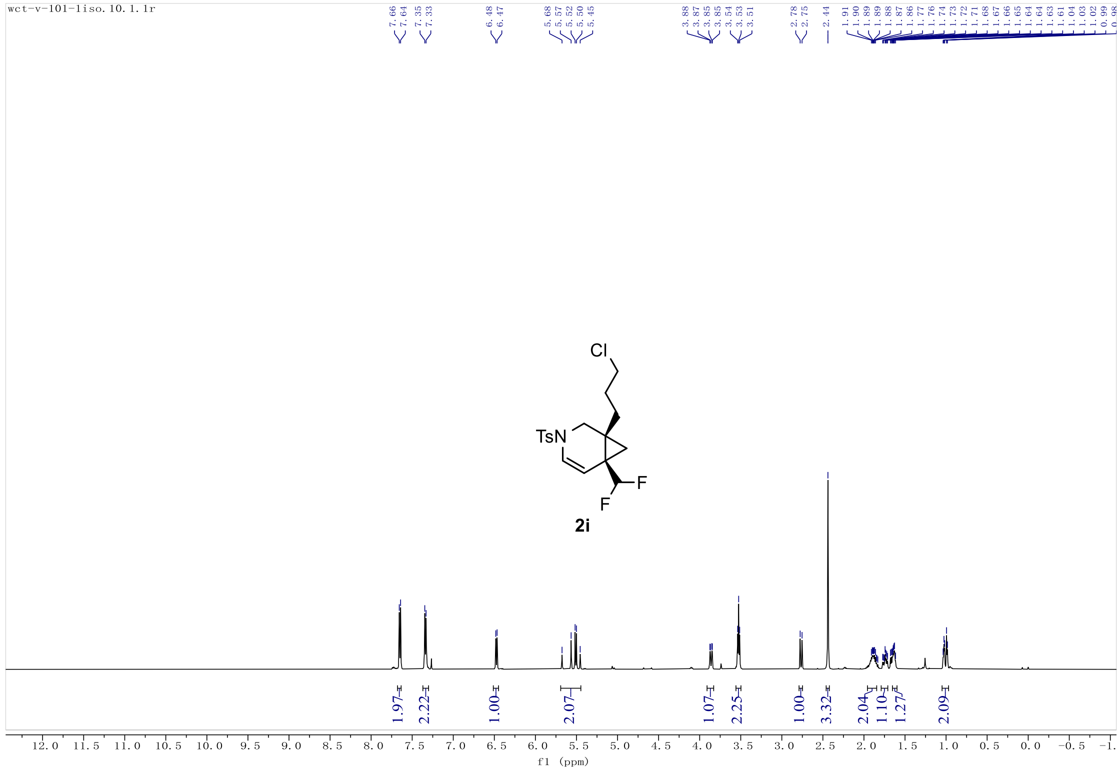
wct-v-101-1iso.10.1.1r
7.66
7.64
7.35
7.33
6.48
6.47
5.68
5.57
5.52
5.50
5.45
3.88
3.87
3.85
3.85
3.54
3.53
3.51
2.78
2.75
2.44
Cl
TsN
F
F
2i
1.97
2.22
1.00
2.07
1.07
2.25
1.00
3.32
2.04
1.10
1.27
2.09
12.0 11.5 11.0 10.5 10.0 9.5 9.0 8.5 8.0 7.5 7.0 6.5 6.0 5.5 5.0 4.5 4.0 3.5 3.0 2.5 2.0 1.5 1.0 0.5 0.0 -0.5
f1 (ppm)

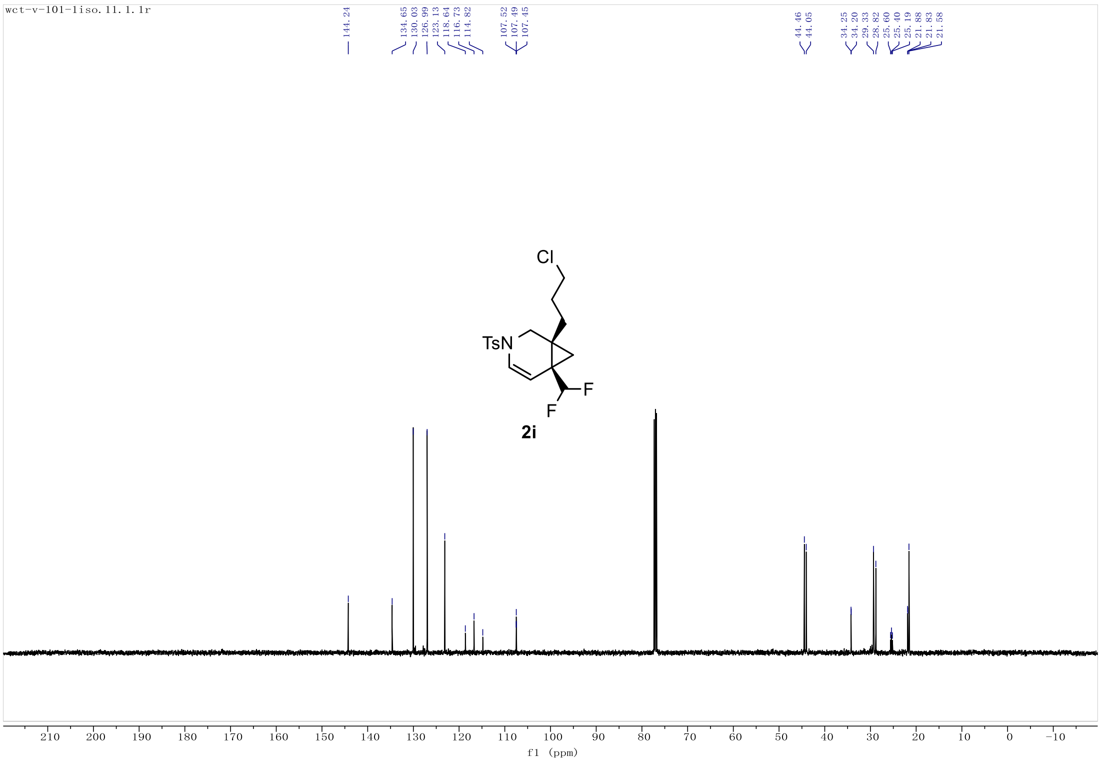
wct-v-101-1iso.11.1.1r
144.24
134.65
130.03
126.99
123.13
118.64
116.73
114.82
107.52
107.49
107.45
44.46
44.05
34.25
34.20
29.33
28.82
25.60
25.40
25.19
21.88
21.83
21.58
Cl
TsN
F
F
2i
f1 (ppm)

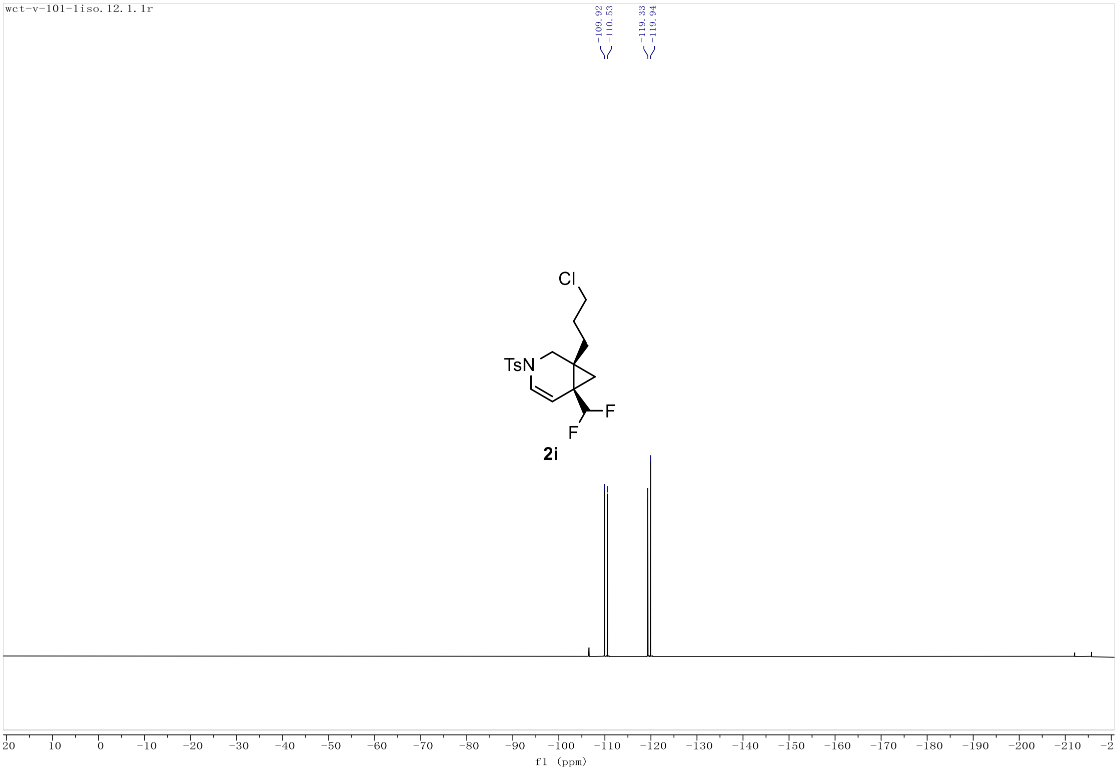
wct-v-101-1iso.12.1.1r
-109.92
-110.53
-119.33
-119.94
Cl
TsN
F
F
2i
f1 (ppm)

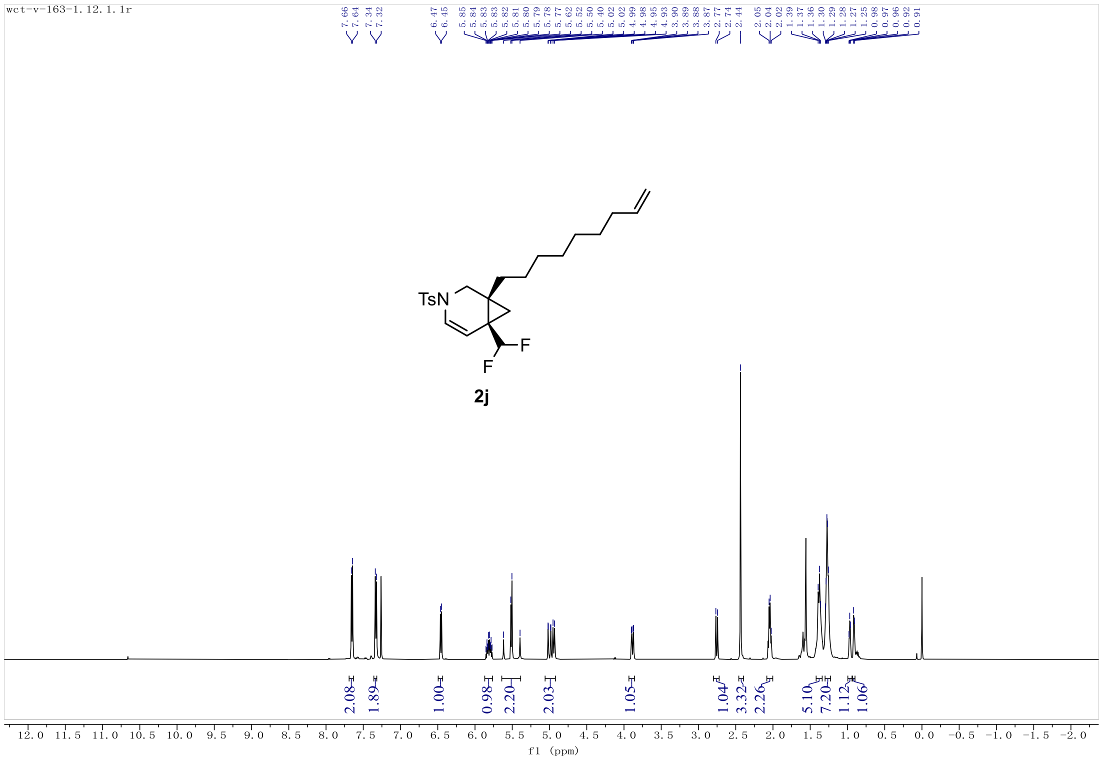

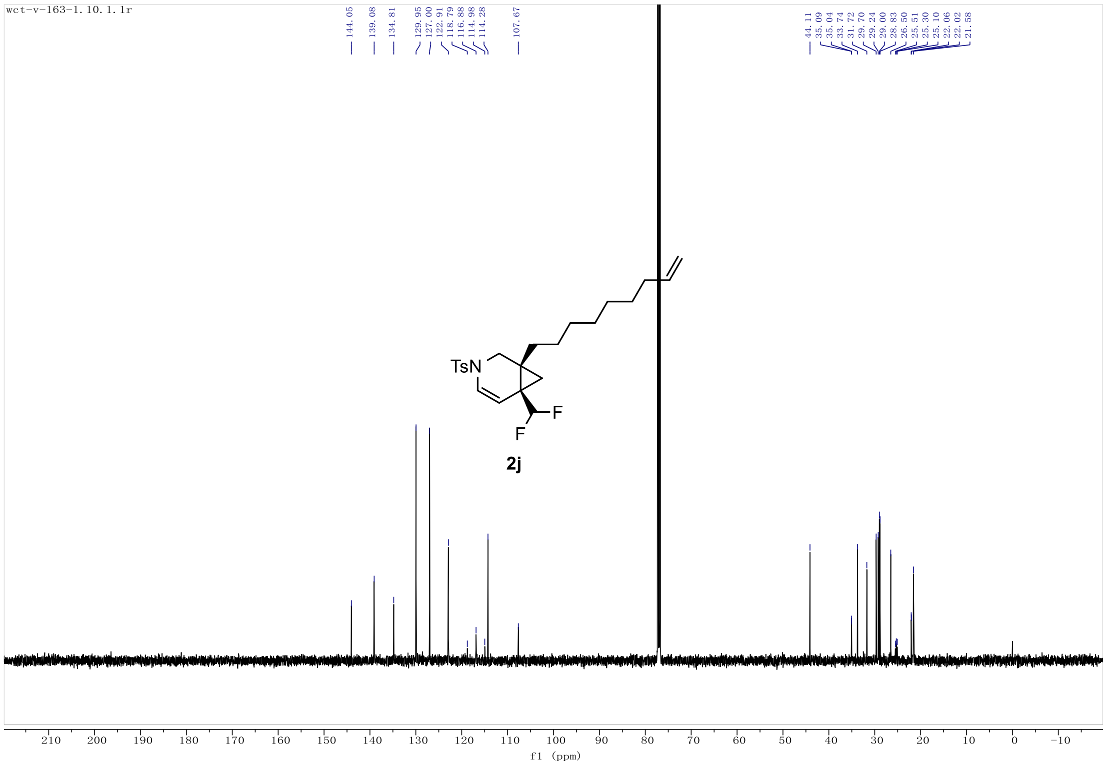

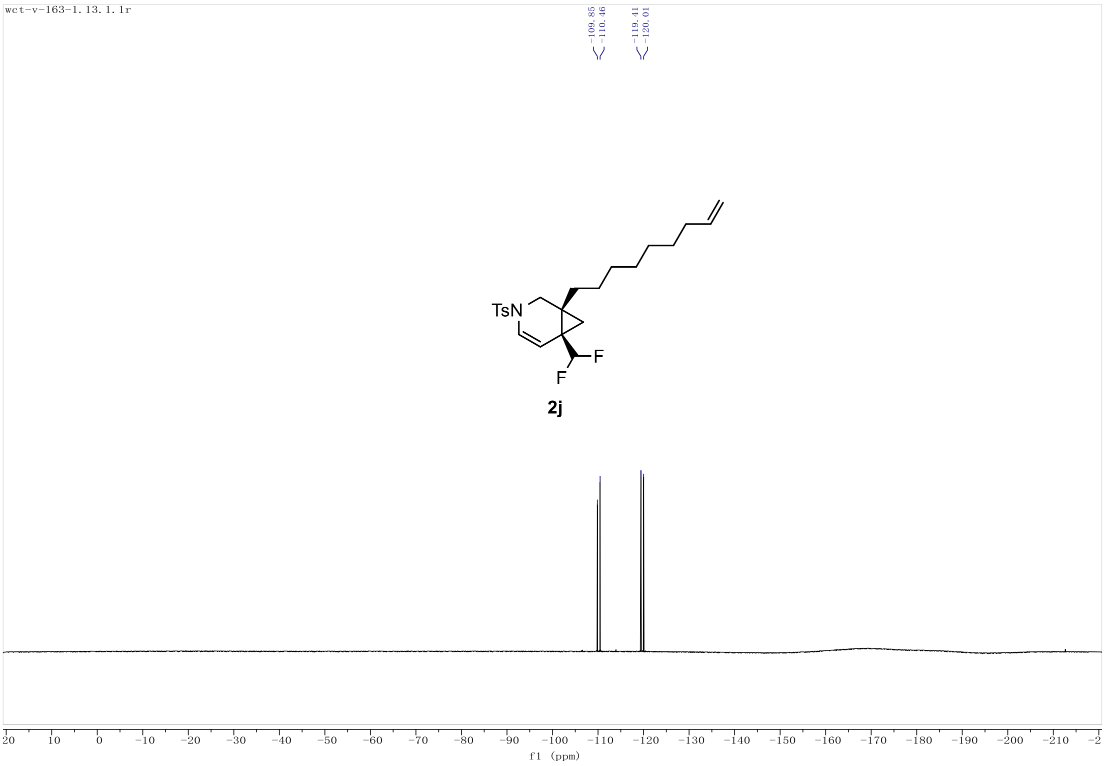
wct-v-163-1.13.1.1r
-109.85
-110.46
-119.41
-120.01
TsN
F
F
2j
f1 (ppm)

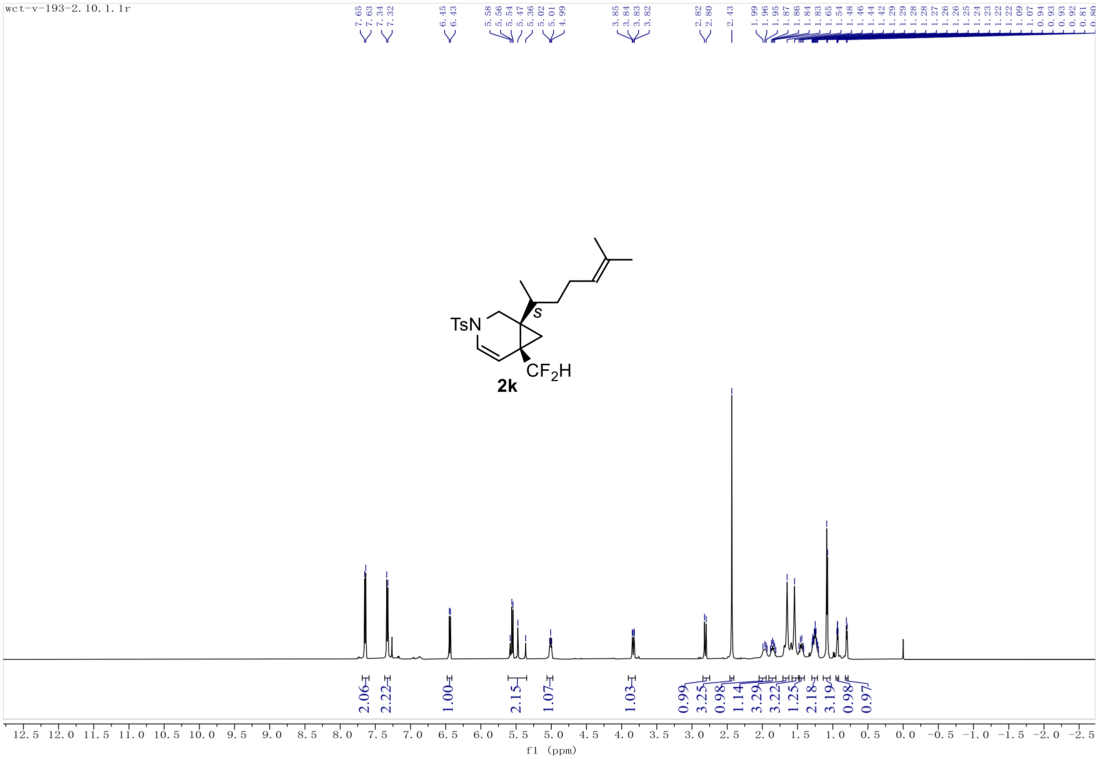
wct-v-193-2.10.1.1r
TsN
S
CF2H
2k
2.06
2.22
1.00
2.15
1.07
1.03
f1 (ppm)

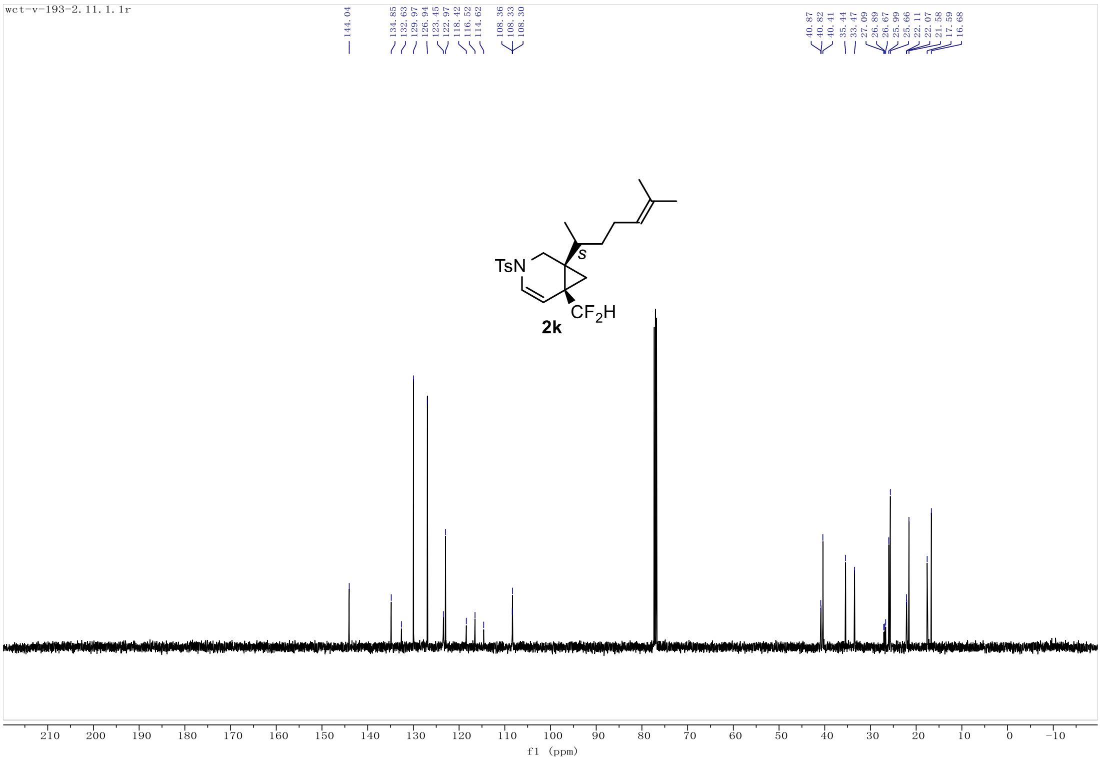
wct-v-193-2.11.1.1r
144.04
134.85
132.63
129.97
126.94
123.45
122.97
118.42
116.52
114.62
108.36
108.33
108.30
40.87
40.82
40.41
35.44
33.47
27.09
26.89
26.67
25.99
25.66
22.11
22.07
21.58
17.59
16.68
TsN
S
CF2H
2k
f1 (ppm)

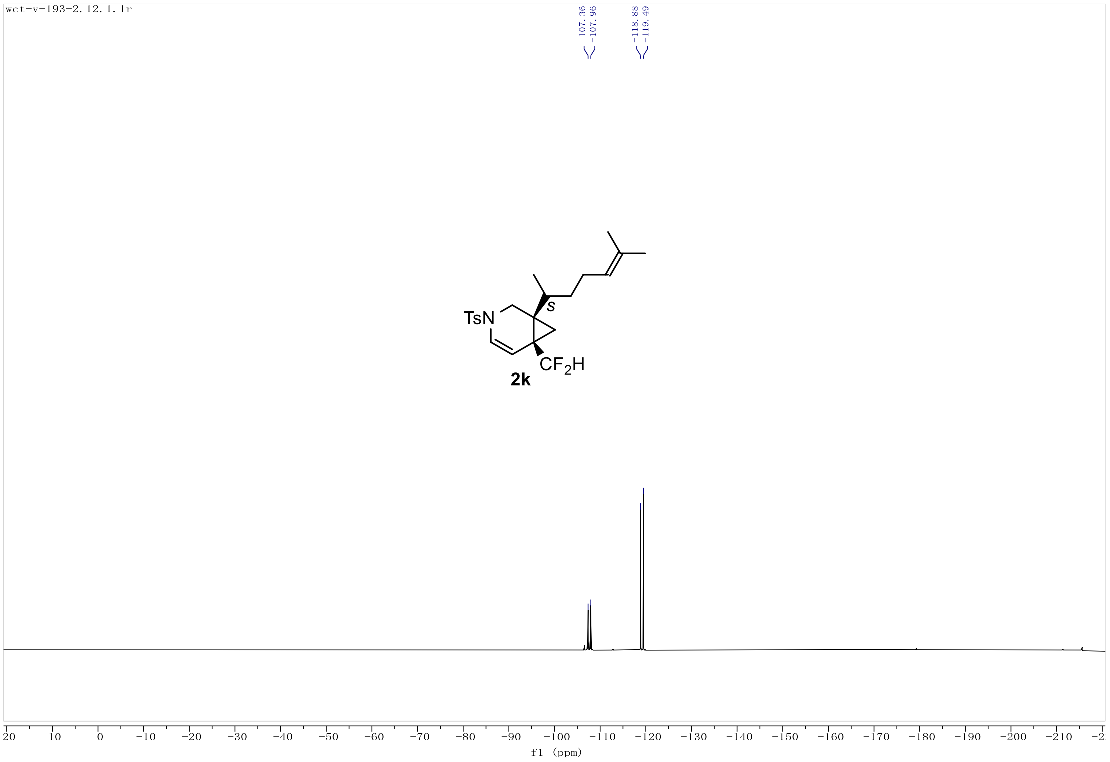
wct-v-193-2.12.1.1r
-107.36
-107.96
-118.88
-119.49
TsN
S
CF2H
2k
f1 (ppm)

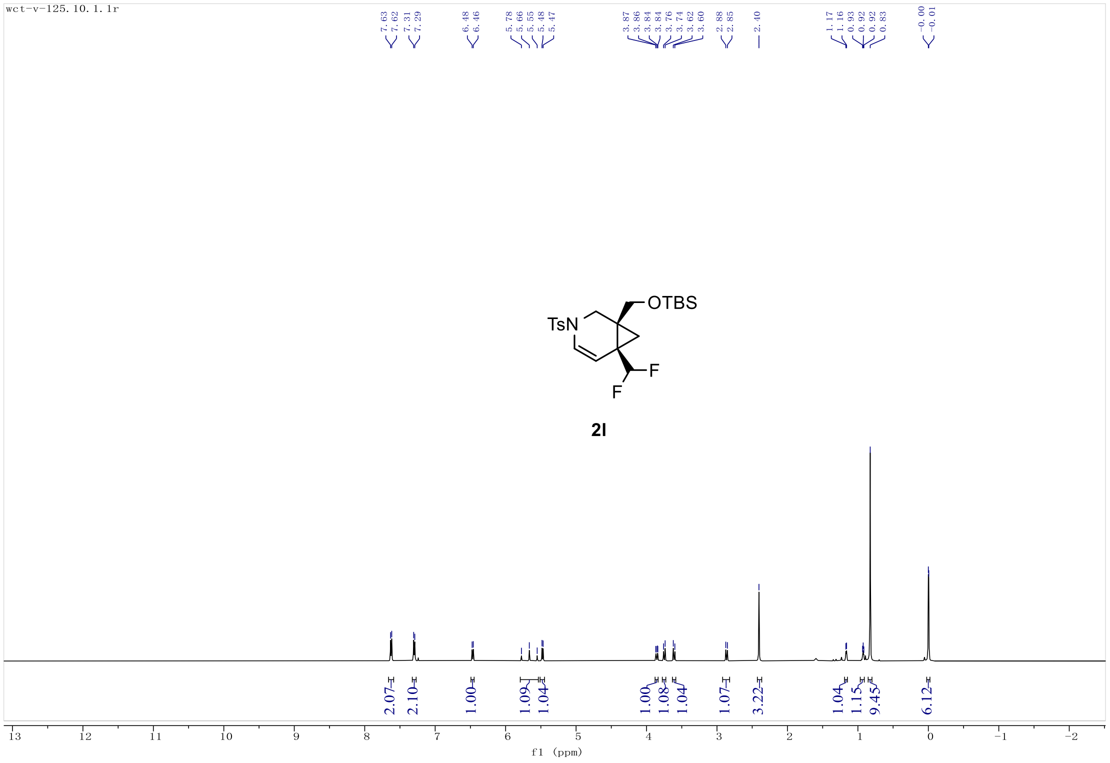
wct-v-125.10.1.1r
7.63
7.62
7.31
7.29
6.48
6.46
5.78
5.66
5.55
5.48
5.47
3.87
3.86
3.84
3.84
3.76
3.74
3.62
3.60
2.88
2.85
2.40
1.17
1.16
0.93
0.92
0.92
0.83
-0.00
-0.01
TsN
OTBS
F
F
2l
2.07
2.10
1.00
1.09
1.04
1.00
1.08
1.04
1.07
3.22
1.04
1.15
9.45
6.12
13 12 11 10 9 8 7 6 5 4 3 2 1 0 -1 -2
f1 (ppm)

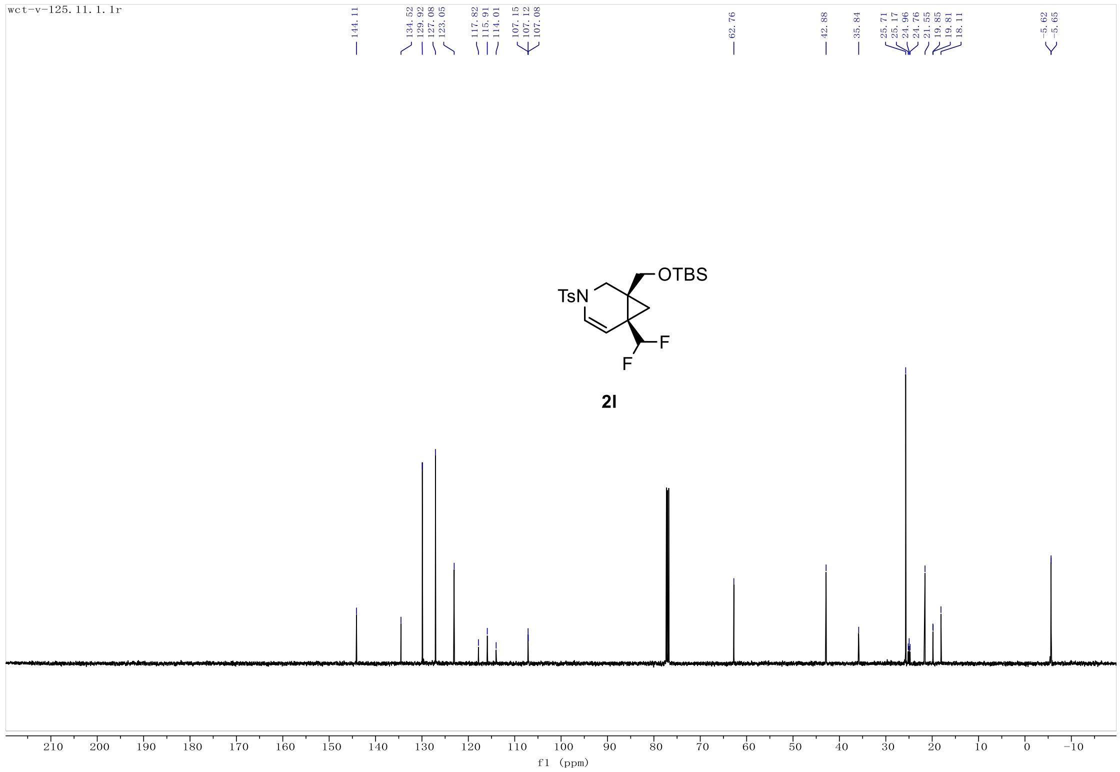
wct-v-125.11.1.1r
144.11
134.52
129.92
127.08
123.05
117.82
115.91
114.01
107.15
107.12
107.08
62.76
42.88
35.84
25.71
25.17
24.96
24.76
21.55
19.85
19.81
18.11
-5.62
-5.65
TsN
OTBS
F
F
2l
210 200 190 180 170 160 150 140 130 120 110 100 90 80 70 60 50 40 30 20 10 0 -10
f1 (ppm)

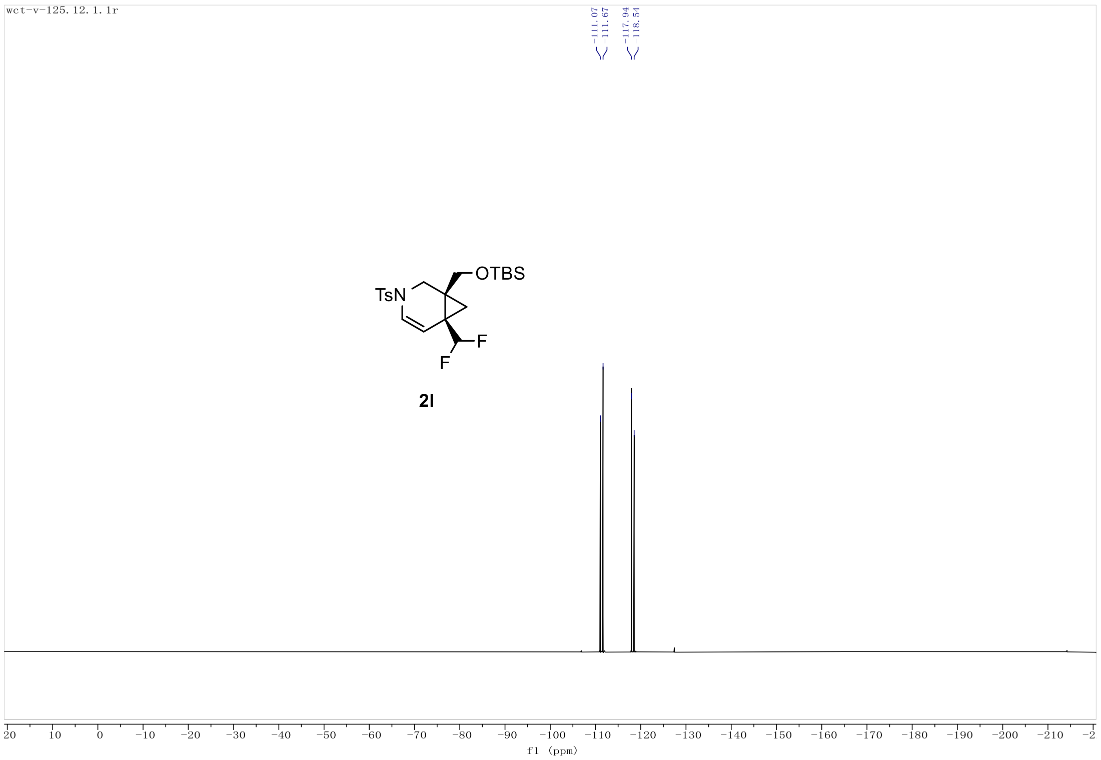
wct-v-125.12.1.1r
-111.07
-111.67
-117.94
-118.54
TsN
OTBS
F
F
2l
f1 (ppm)

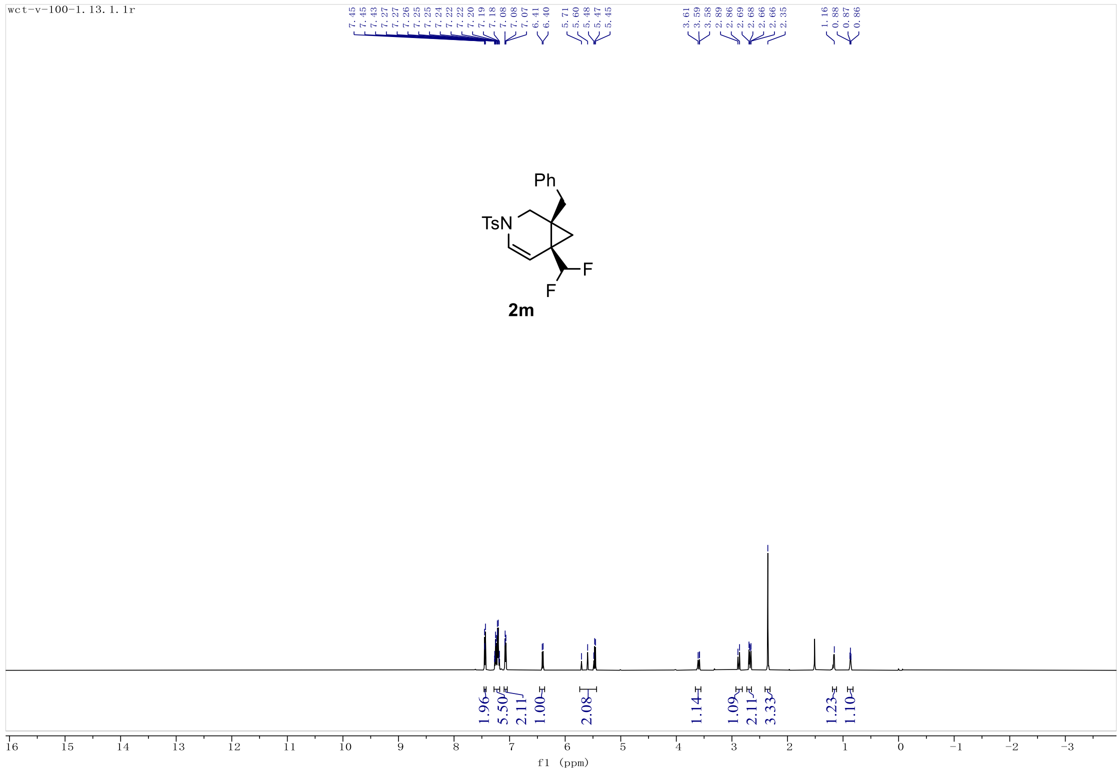
wct-v-100-1.13.1.1r
7.45
7.45
7.43
7.27
7.27
7.26
7.25
7.25
7.24
7.22
7.22
7.20
7.19
7.18
7.08
7.07
6.41
6.40
5.71
5.60
5.48
5.47
5.45
3.61
3.59
3.58
2.89
2.86
2.69
2.68
2.66
2.66
2.35
1.16
0.88
0.87
0.86
Ph
TsN
F
F
2m
1.96
5.50
2.11
1.00
2.08
1.14
1.09
2.11
3.33
1.23
1.10
f1 (ppm)

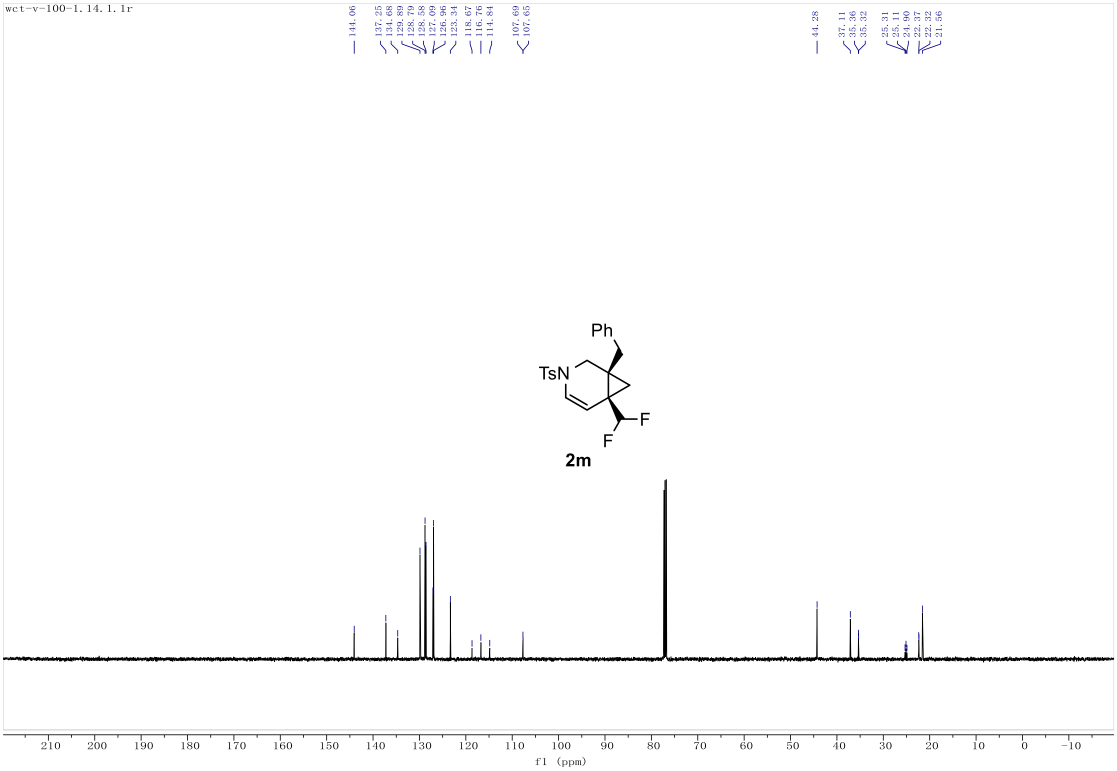
wct-v-100-1.14.1.1r
144.06
137.25
134.68
129.89
128.79
128.58
127.09
126.96
123.34
118.67
116.76
114.84
107.69
107.65
44.28
37.11
35.36
35.32
25.31
25.11
24.90
22.37
22.32
21.56
Ph
TsN
F
F
2m
f1 (ppm)

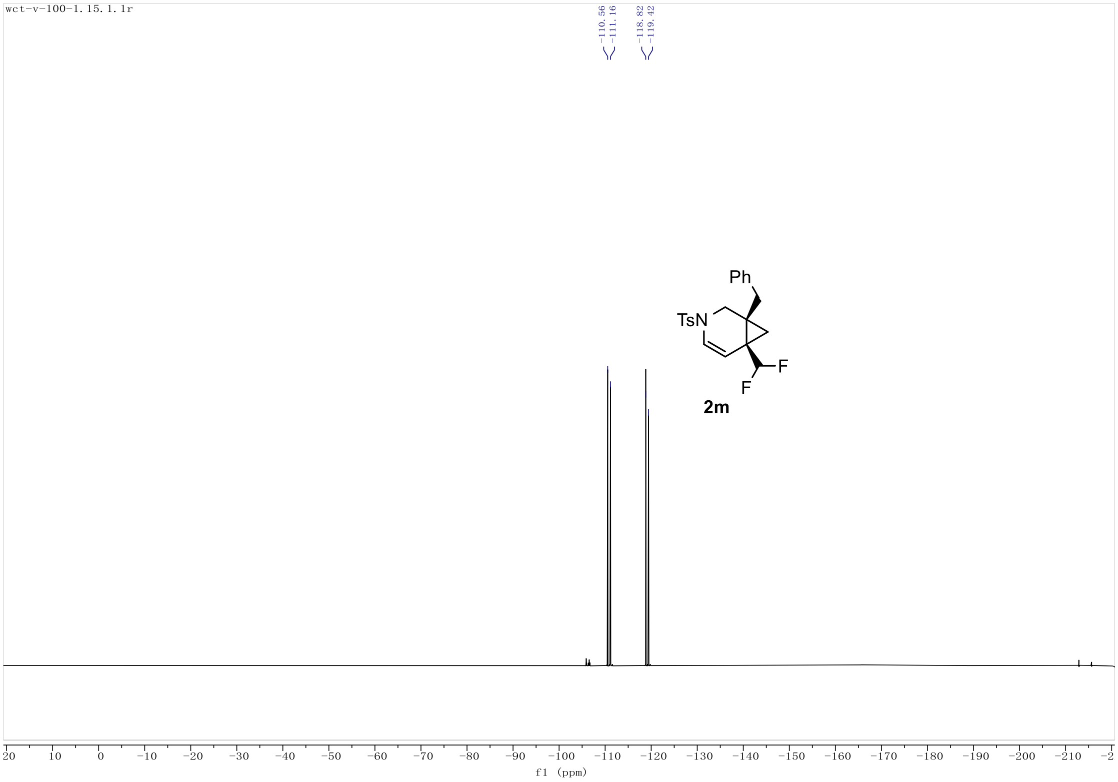
wct-v-100-1.15.1.1r
-110.56
-111.16
-118.82
-119.42
Ph
TsN
F
F
2m
f1 (ppm)

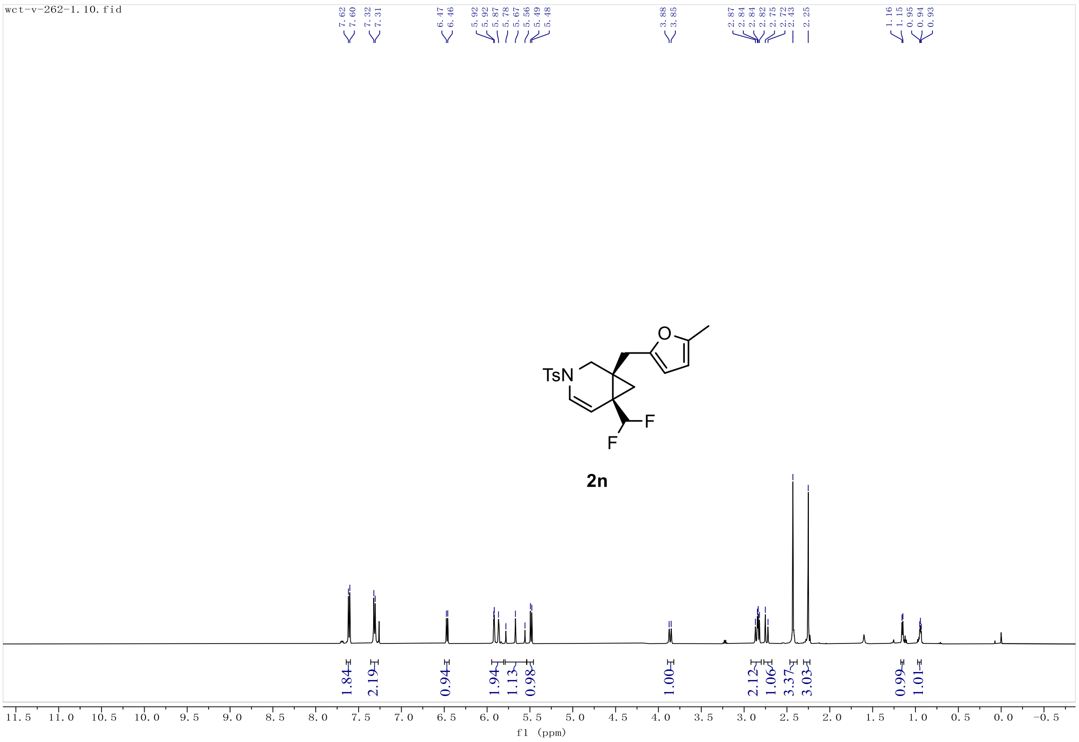
wct-v-262-1.10.fid
f1 (ppm)

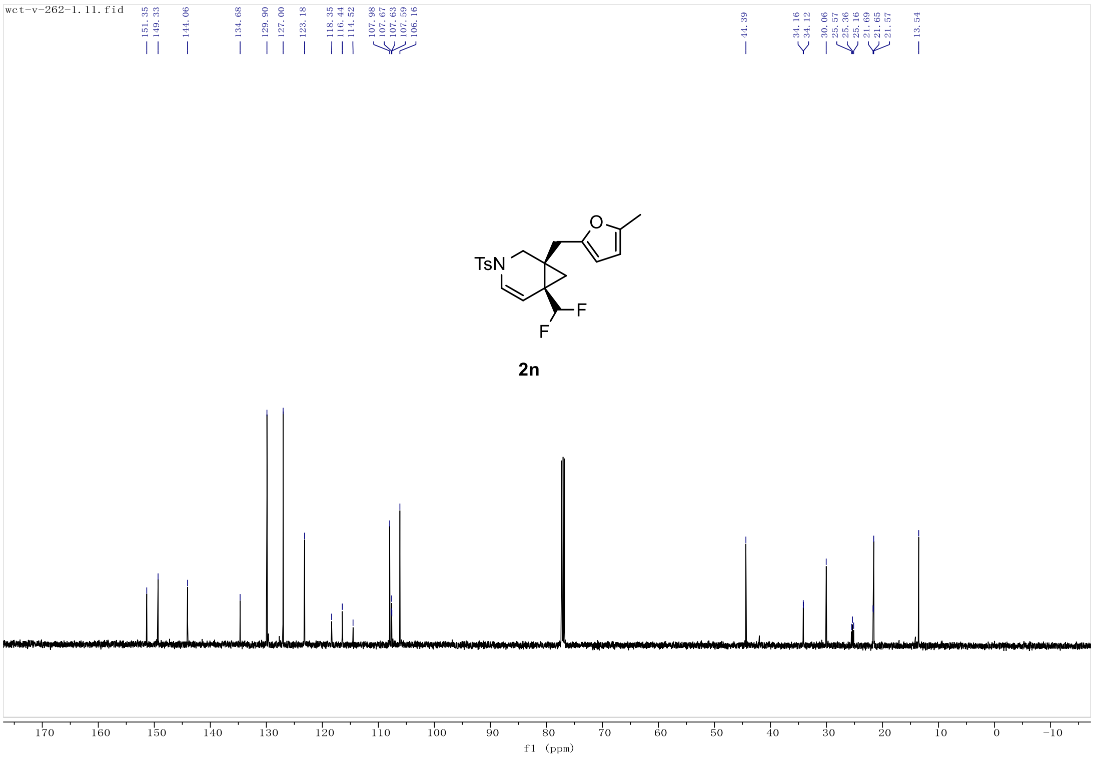
wct-v-262-1.11.fid
f1 (ppm)

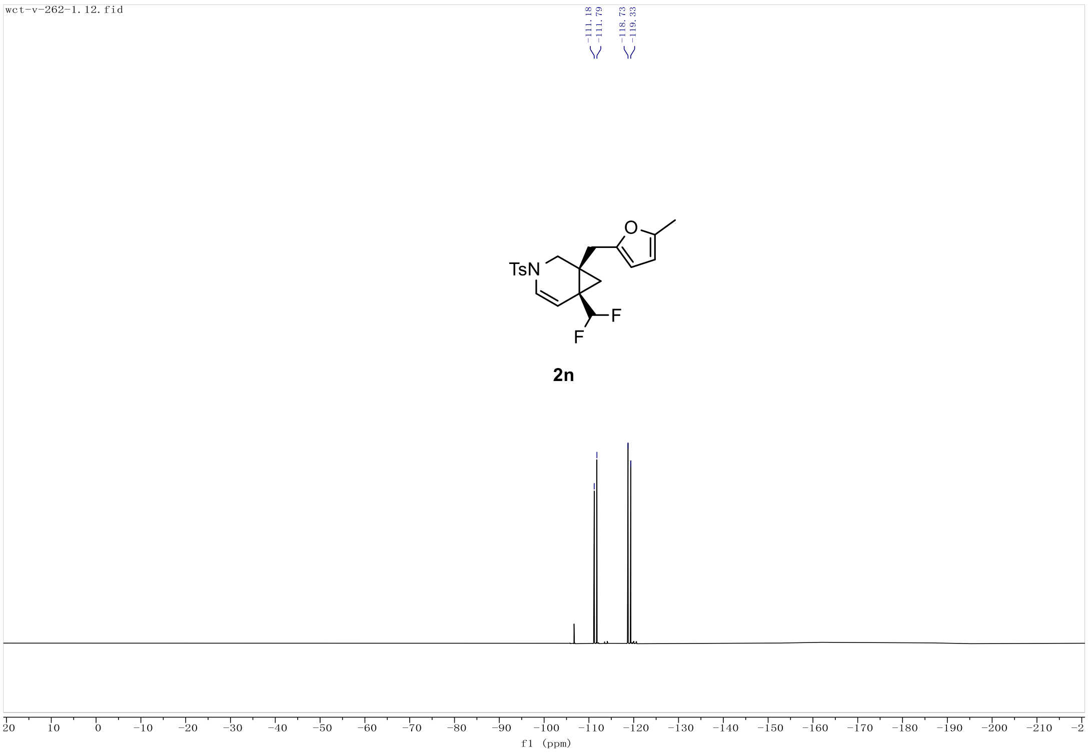
wct-v-262-1.12.fid
-111.18
-111.79
-118.73
-119.33
TsN
O
F
F
2n
f1 (ppm)

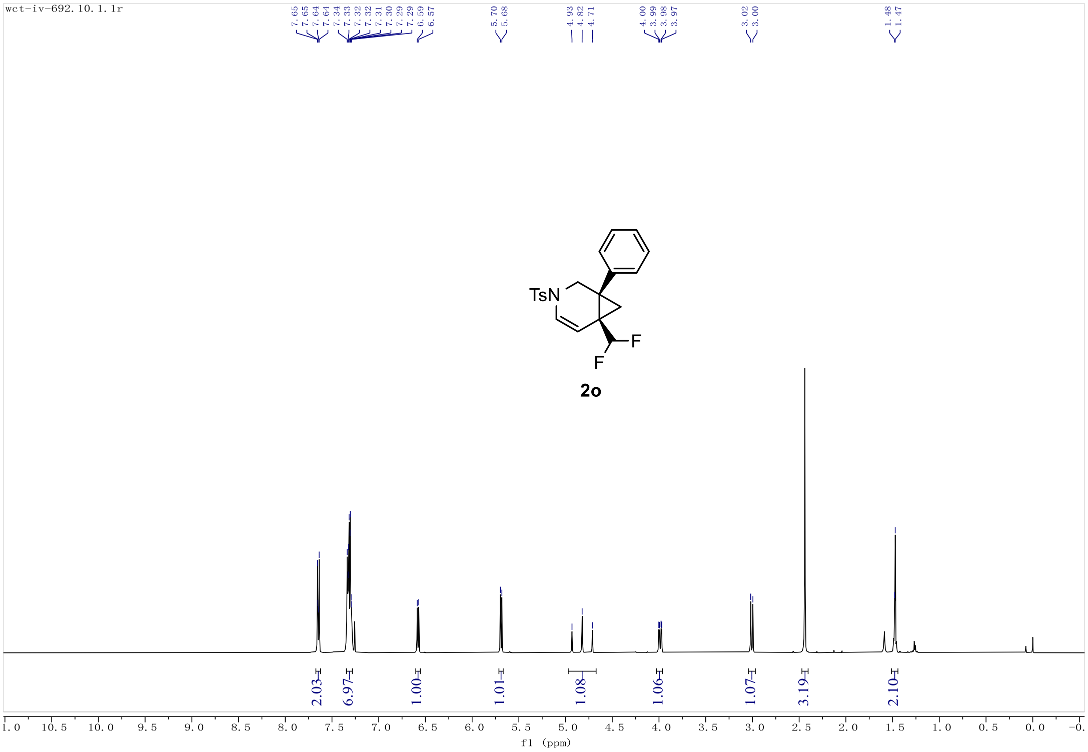
wct-iv-692.10.1.1r
TsN
F
F
2o
2.03
6.97
1.00
1.01
1.08
1.06
1.07
3.19
2.10
f1 (ppm)

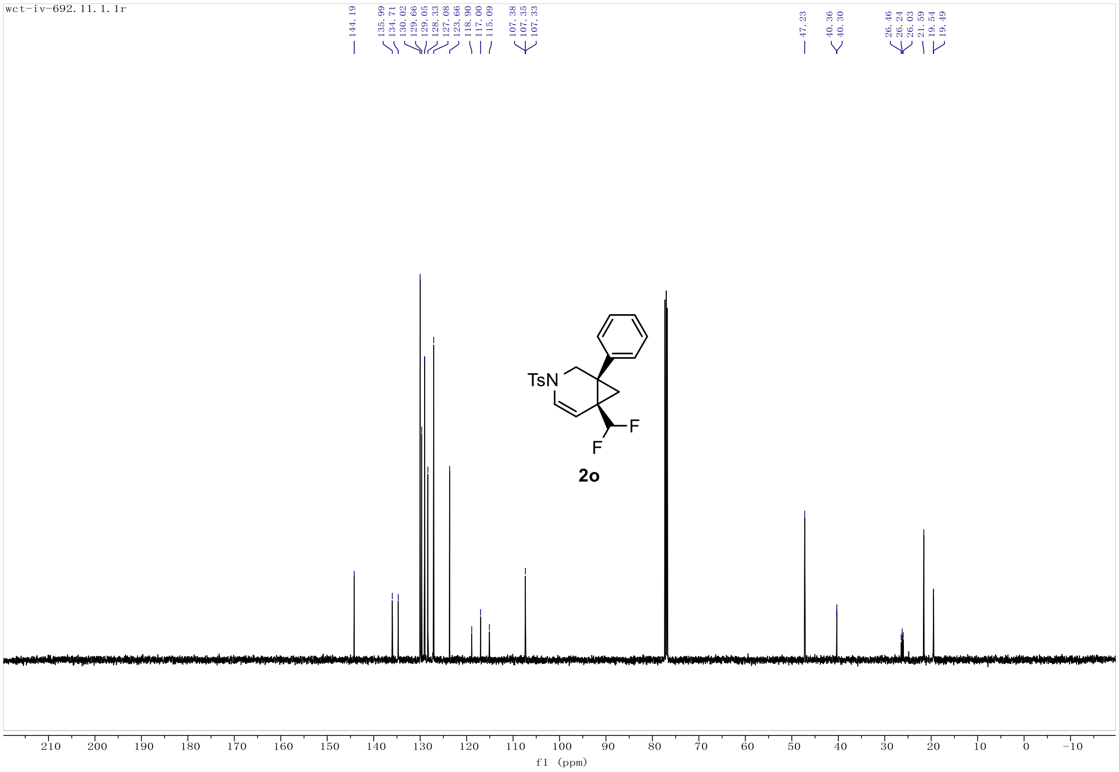

2o

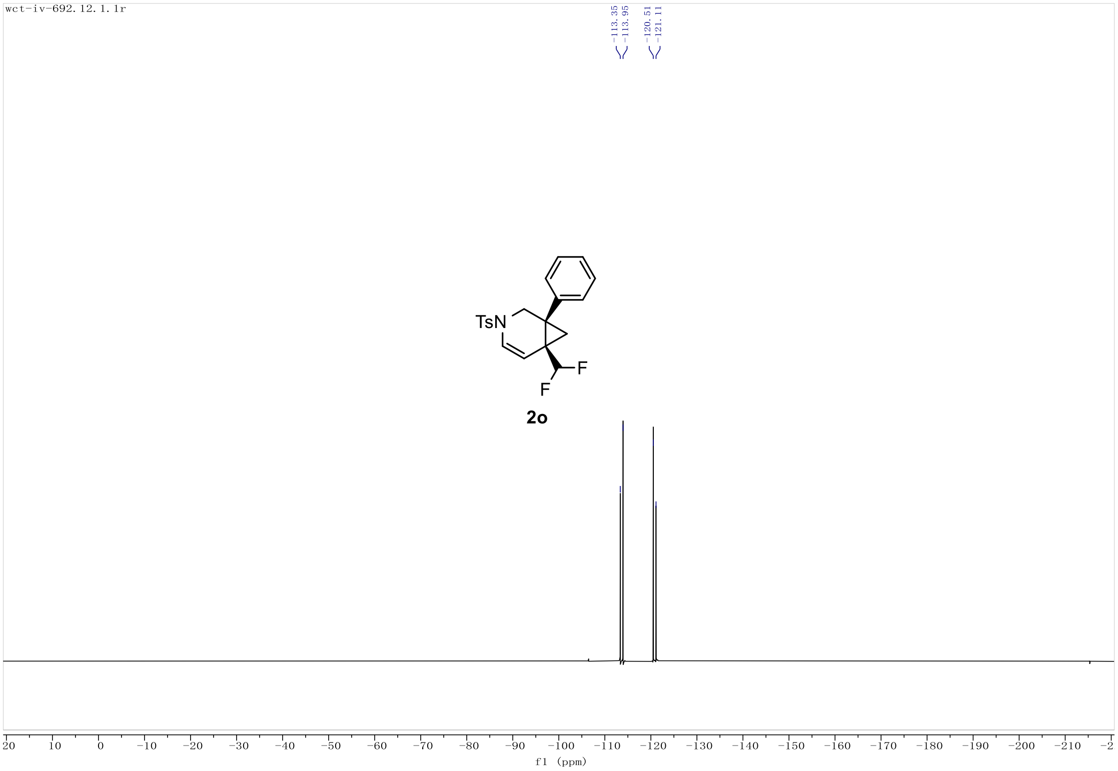

2o

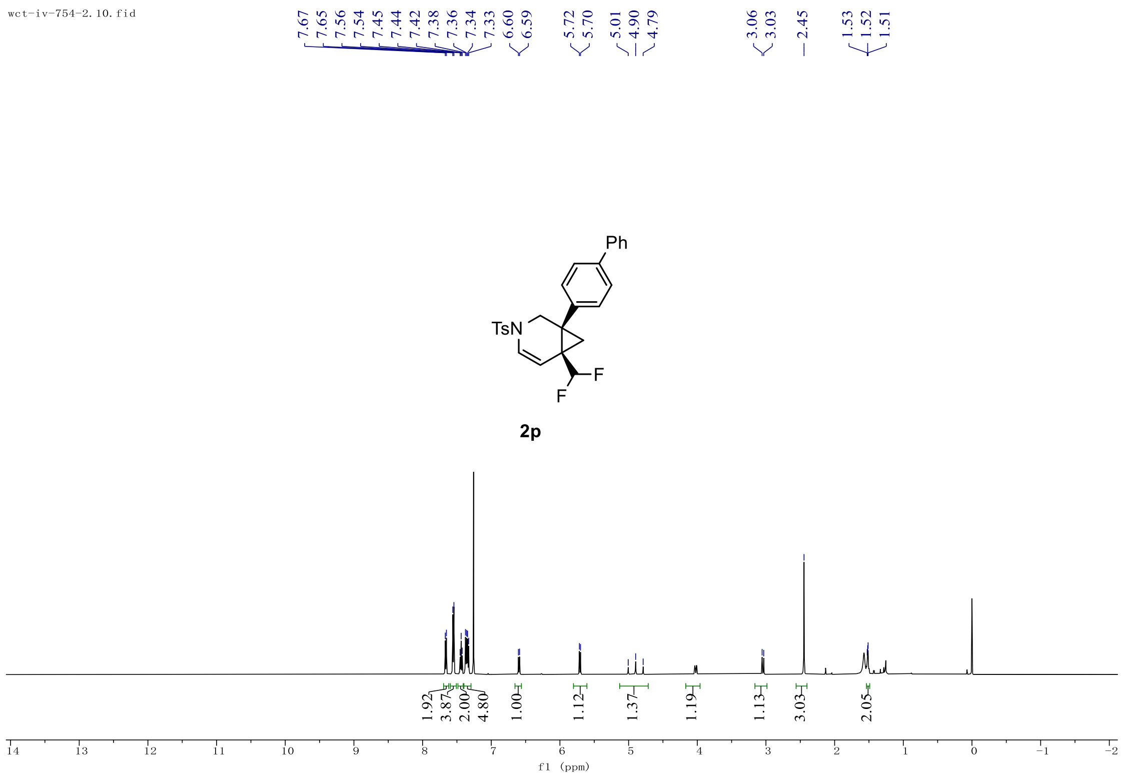
wct-iv-754-2.10.fid
7.67
7.65
7.56
7.54
7.45
7.44
7.42
7.38
7.36
7.34
7.33
6.60
6.59
5.72
5.70
5.01
4.90
4.79
3.06
3.03
2.45
1.53
1.52
1.51
Ph
TsN
F
F
2p
1.92
3.87
2.00
4.80
1.00
1.12
1.37
1.19
1.13
3.03
2.05
f1 (ppm)

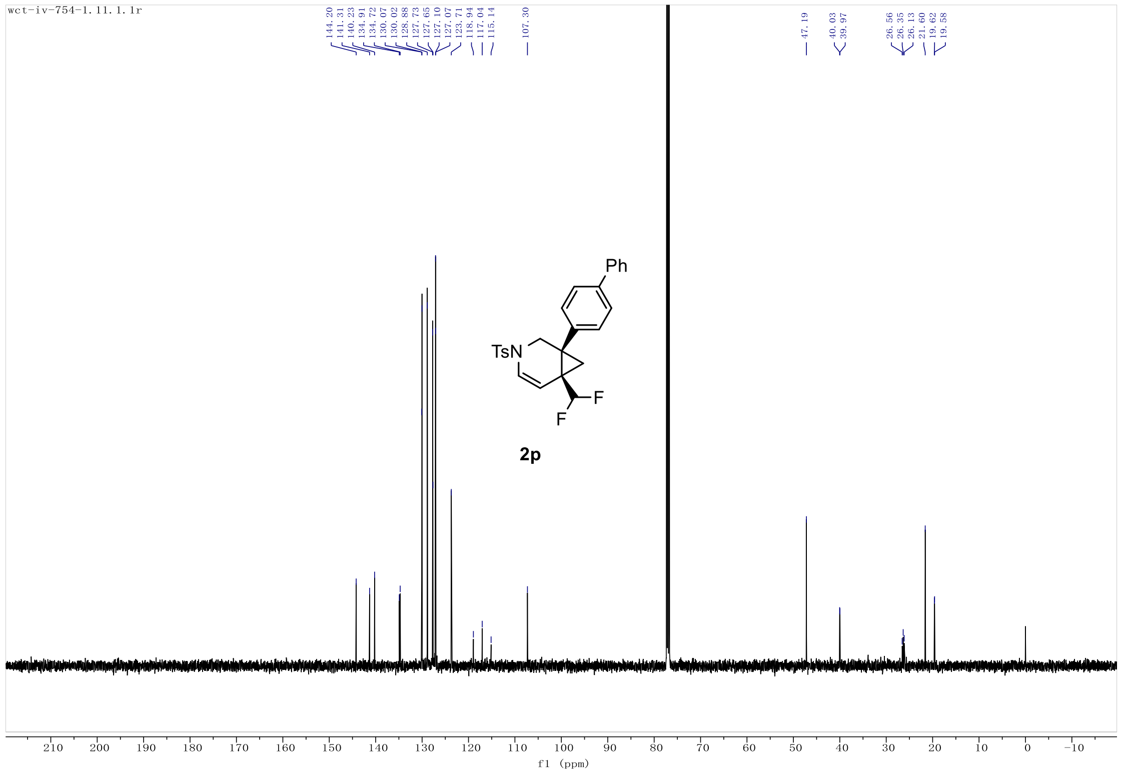
wct-iv-754-1.11.1.1r
144.20
141.31
140.23
134.91
134.72
130.07
130.02
128.88
127.73
127.65
127.10
127.07
123.71
118.94
117.04
115.14
107.30
47.19
40.03
39.97
26.56
26.35
26.13
21.60
19.62
19.58
Ph
TsN
F
F
2p
f1 (ppm)

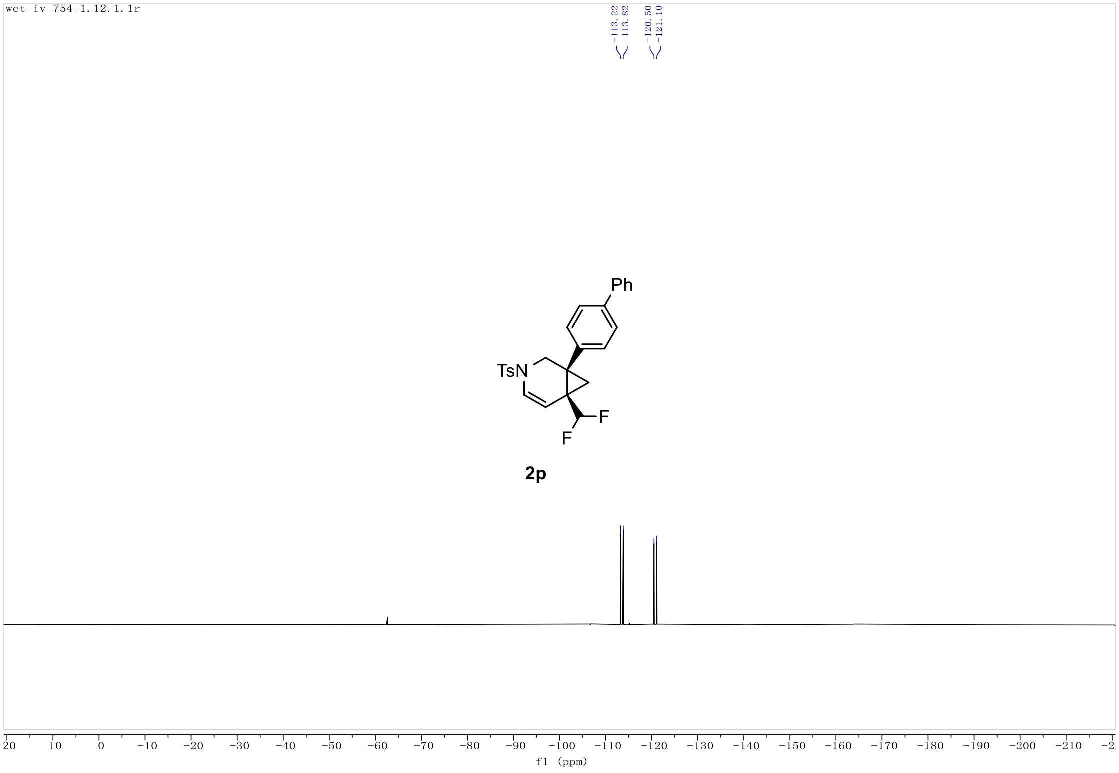
wct-iv-754-1.12.1.1r
-113.22
-113.82
-120.50
-121.10
Ph
TsN
F
F
f1 (ppm)

2p

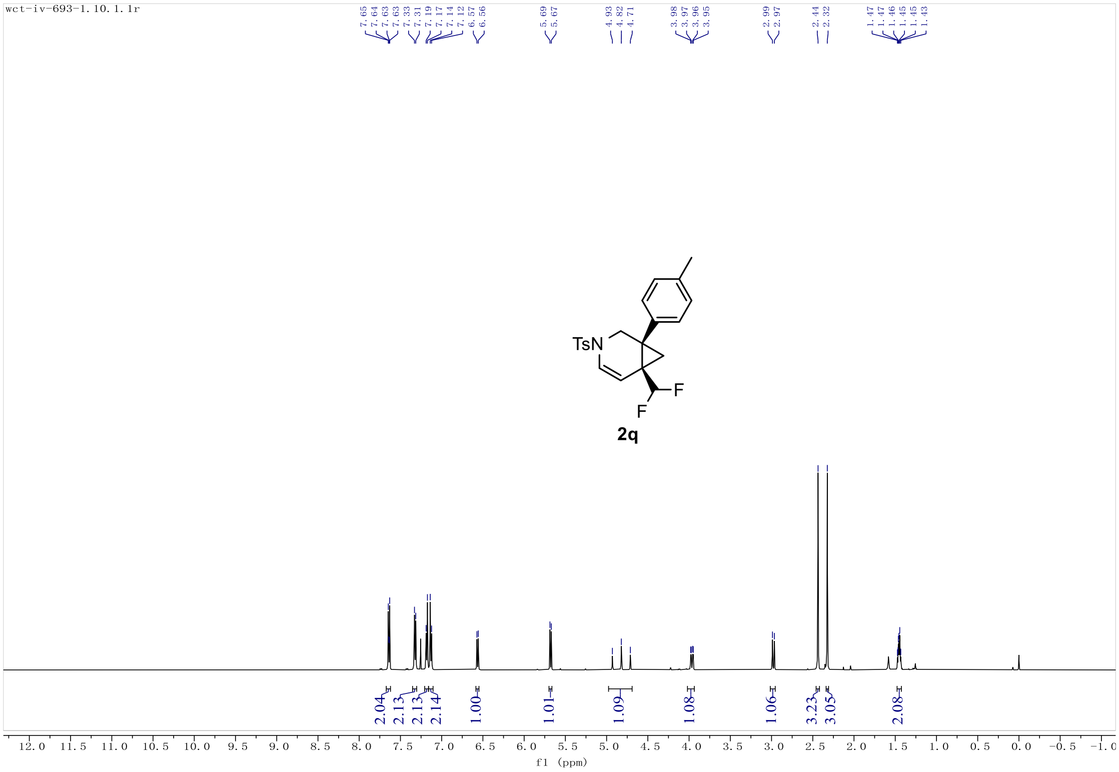
wct-iv-693-1.10.1.1r
TsN
F
F
f1 (ppm)

2q

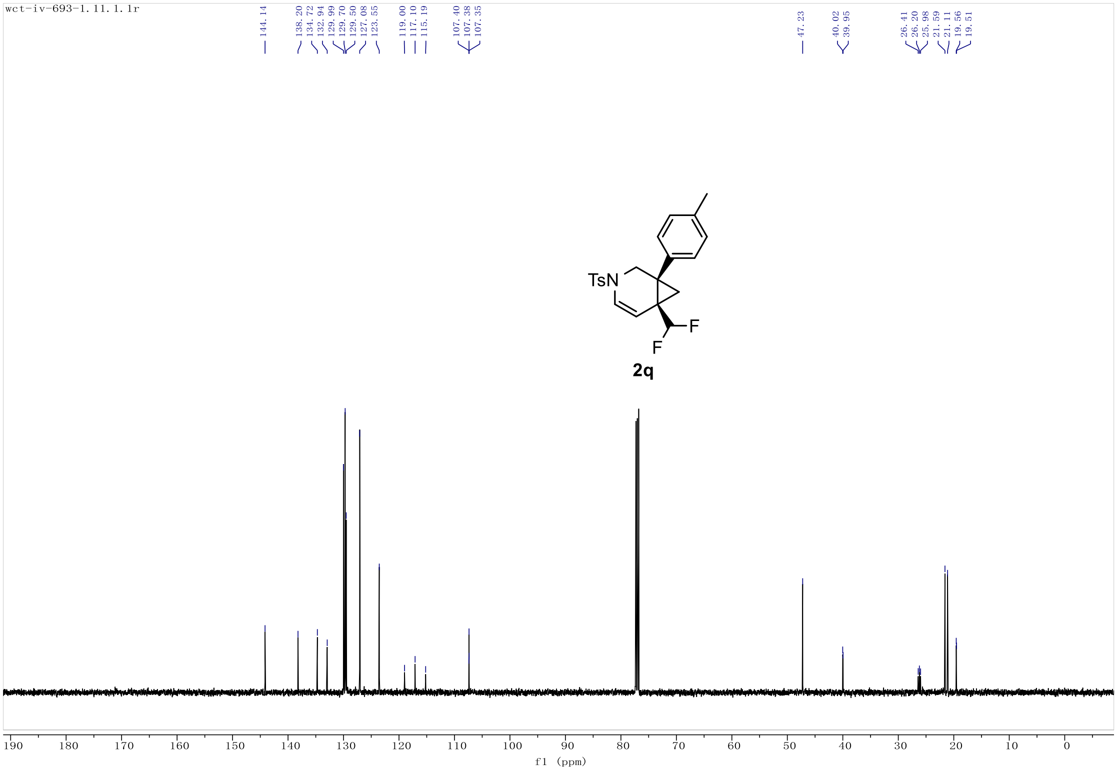
wct-iv-693-1.11.1.1r
144.14
138.20
134.72
132.94
129.99
129.70
129.50
127.08
123.55
119.00
117.10
115.19
107.40
107.38
107.35
47.23
40.02
39.95
26.41
26.20
25.98
21.59
21.11
19.56
19.51
TsN
F
F
2q
190 180 170 160 150 140 130 120 110 100 90 80 70 60 50 40 30 20 10 0
f1 (ppm)

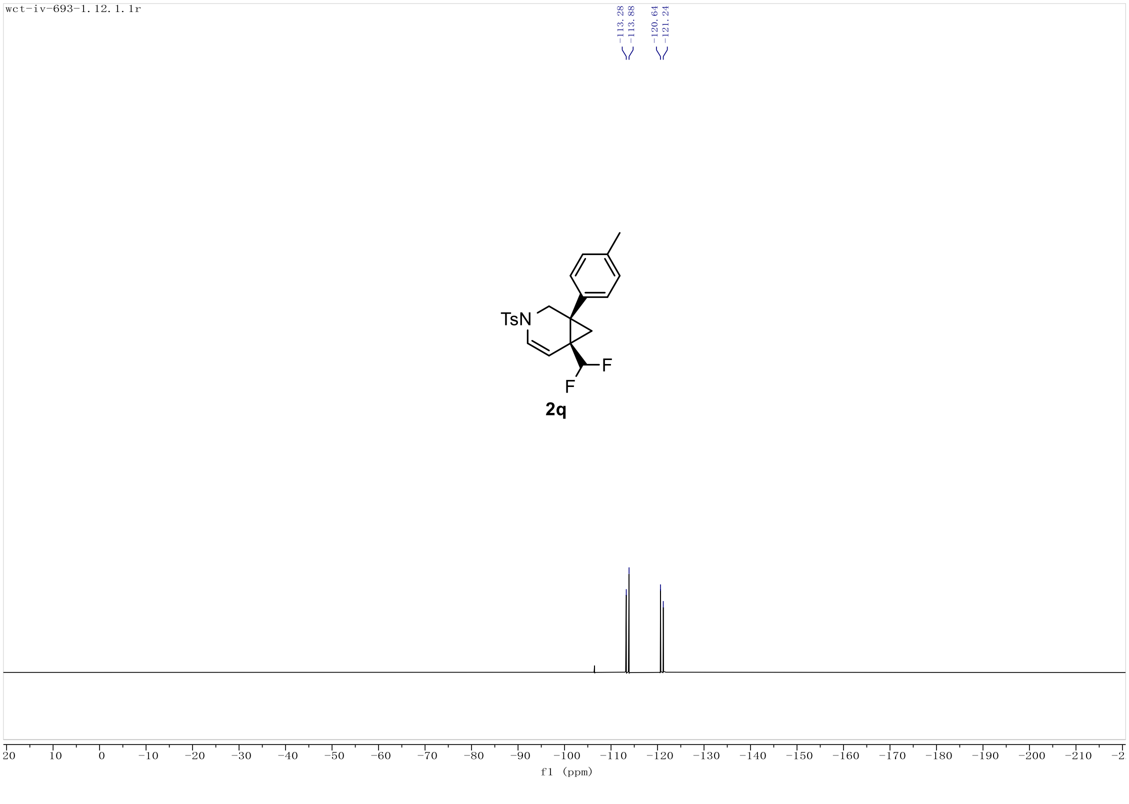
wct-iv-693-1.12.1.1r
-113.28
-113.88
-120.64
-121.24
TsN
F
F
2q
20 10 0 −10 −20 −30 −40 −50 −60 −70 −80 −90 −100 −110 −120 −130 −140 −150 −160 −170 −180 −190 −200 −210 −2
f1 (ppm)

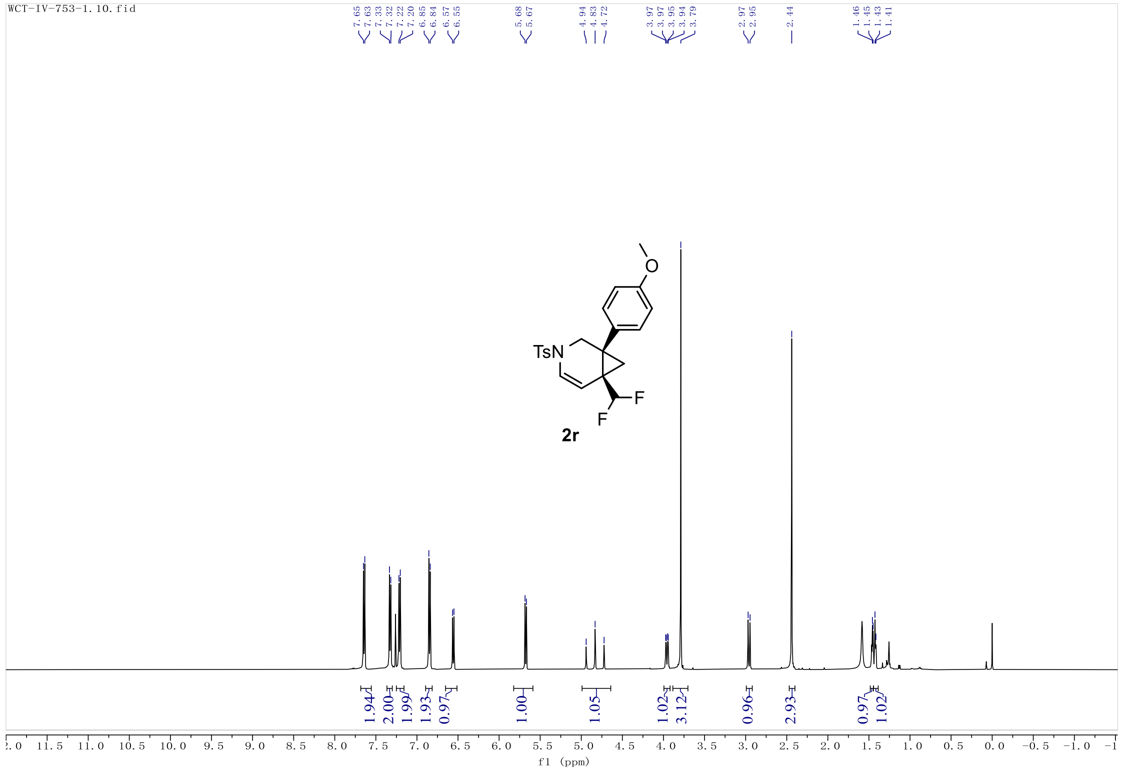
WCT-IV-753-1.10.fid
7.65 7.63 7.33 7.32 7.22 7.20 6.85 6.84 6.57 6.55
5.68 5.67
4.94 4.83 4.72
3.97 3.97 3.95 3.94 3.79
2.97 2.95
2.44
1.46 1.45 1.43 1.41
TsN
O
F
F
2r
1.94 2.00 1.99 1.93 0.97 1.00 1.05 1.02 3.12 0.96 2.93 0.97 1.02
f1 (ppm)

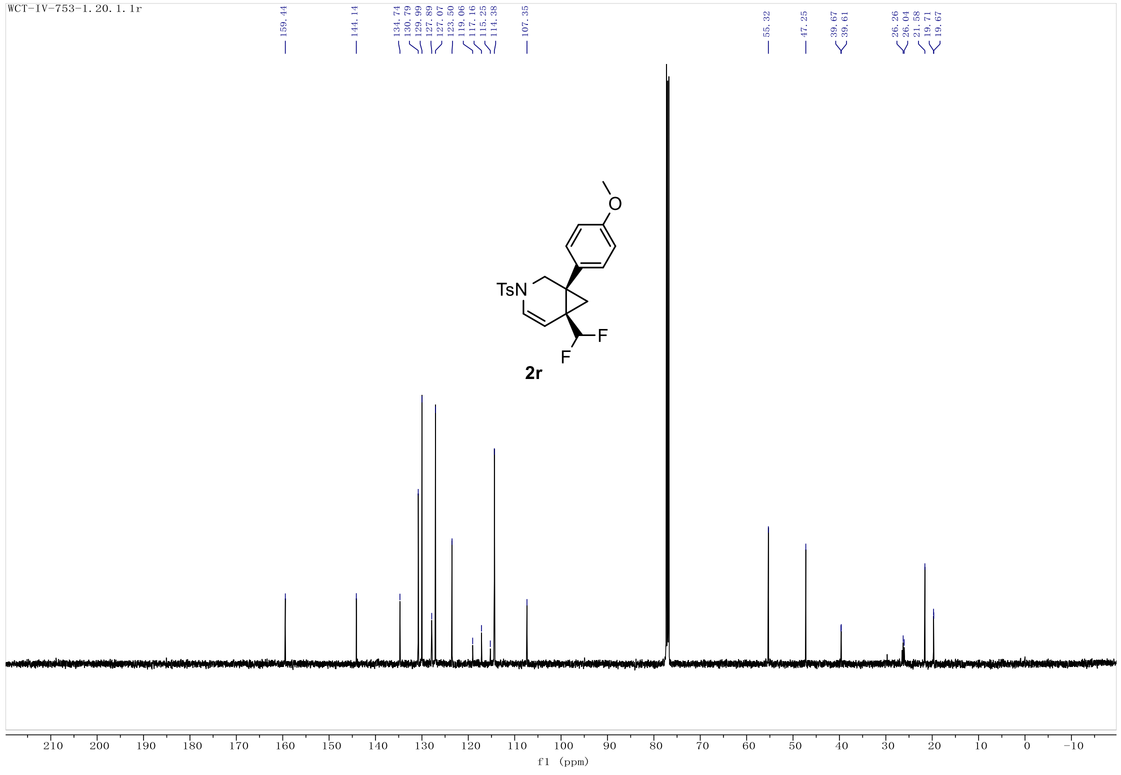
WCT-IV-753-1.20.1.1r
159.44
144.14
134.74 130.79 129.99 127.89 127.07 123.50 119.06 117.16 115.25 114.38
107.35
55.32
47.25
39.67 39.61
26.26 26.04 21.58 19.71 19.67
TsN
O
F
F
2r
f1 (ppm)

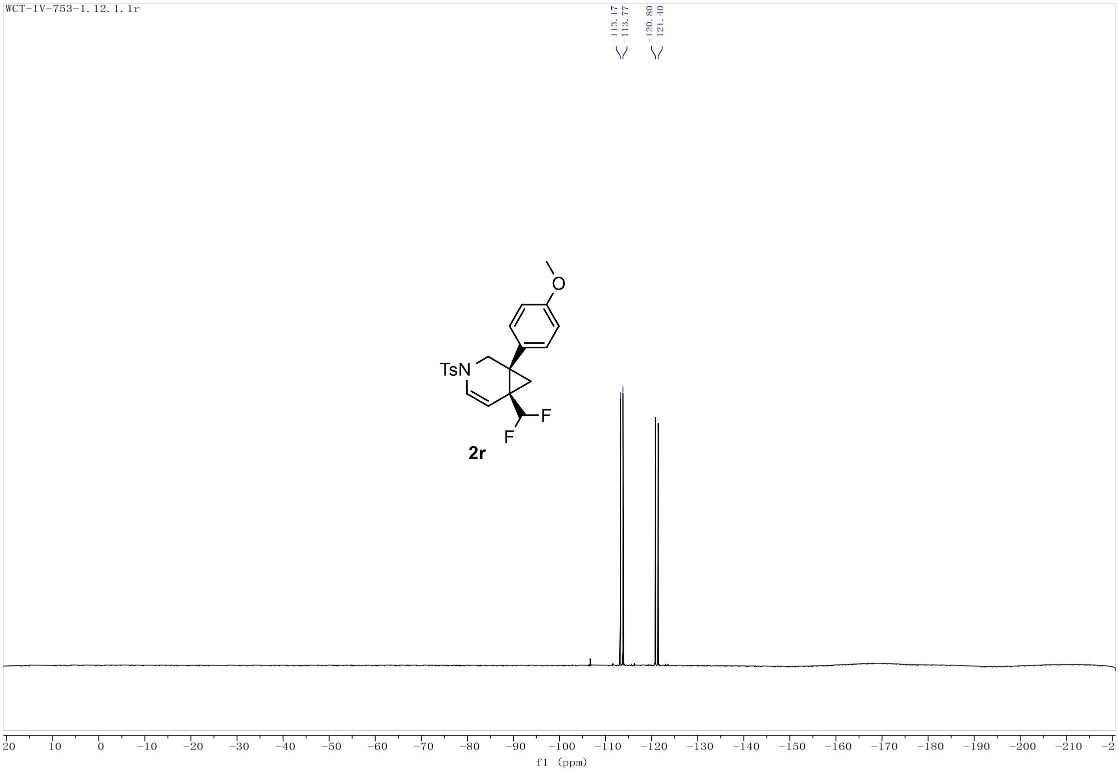
WCT-IV-753-1.12.1.1r
-113.17
-113.77
-120.80
-121.40
TsN
O
F
F
2r
20 10 0 −10 −20 −30 −40 −50 −60 −70 −80 −90 −100 −110 −120 −130 −140 −150 −160 −170 −180 −190 −200 −210
f1 (ppm)

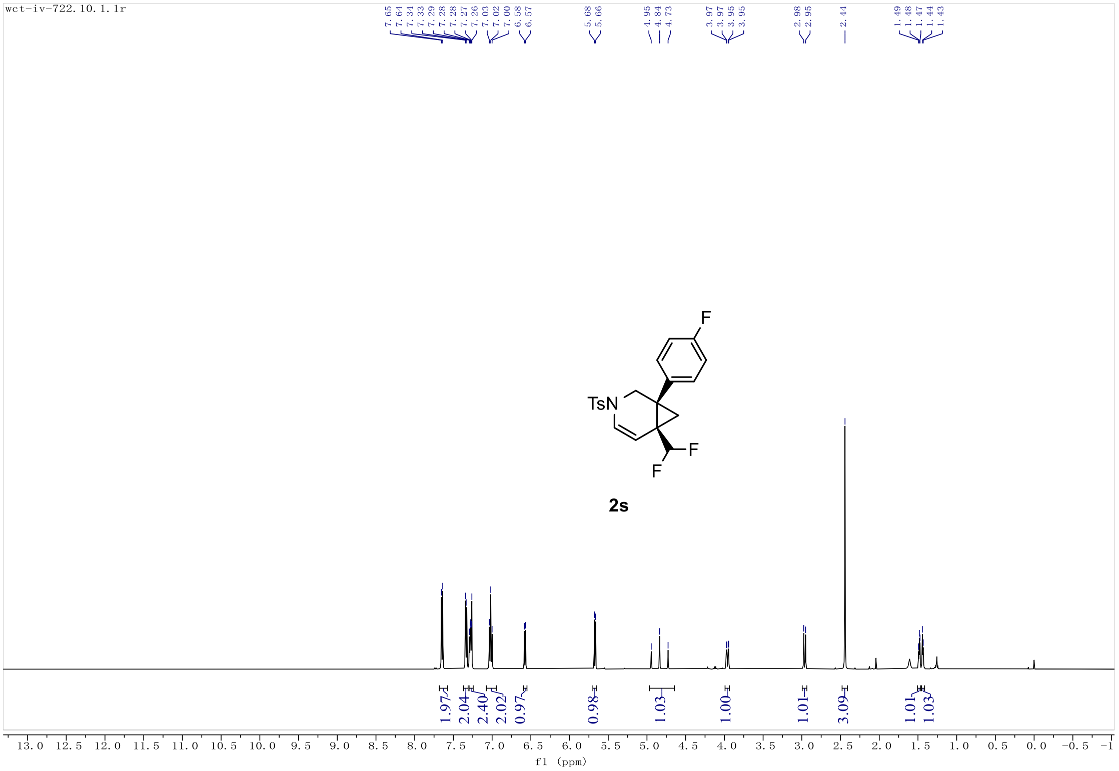
wct-iv-722.10.1.1r
7.65
7.64
7.34
7.33
7.29
7.28
7.27
7.26
7.03
7.02
7.00
6.58
6.57
5.68
5.66
4.95
4.84
4.73
3.97
3.97
3.95
3.95
2.98
2.95
2.44
1.49
1.48
1.47
1.44
1.43
F
TsN
F
F
2s
1.97
2.04
2.40
2.02
0.97
0.98
1.03
1.00
1.01
3.09
1.01
1.03
13.0 12.5 12.0 11.5 11.0 10.5 10.0 9.5 9.0 8.5 8.0 7.5 7.0 6.5 6.0 5.5 5.0 4.5 4.0 3.5 3.0 2.5 2.0 1.5 1.0 0.5 0.0 −0.5
f1 (ppm)

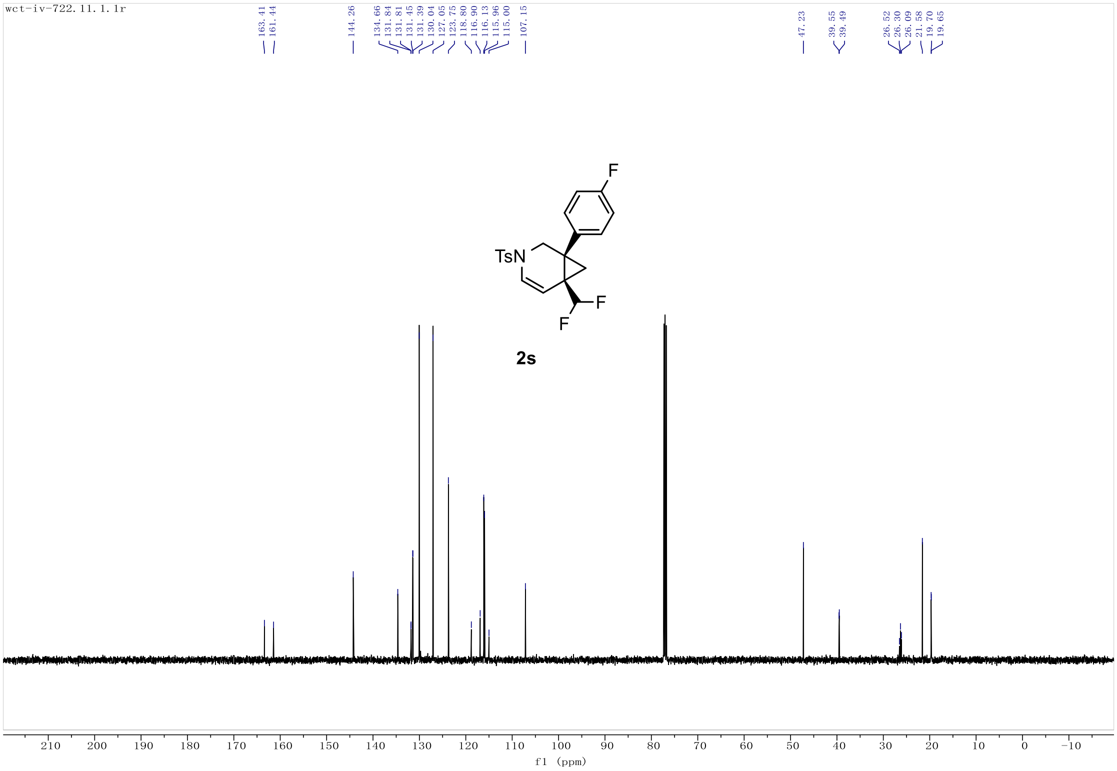
wct-iv-722.11.1.1r
163.41
161.44
144.26
134.66
131.84
131.81
131.45
131.39
130.04
127.05
123.75
118.80
116.90
116.13
115.96
115.00
107.15
47.23
39.55
39.49
26.52
26.30
26.09
21.58
19.70
19.65
TsN
F
F
F
2s
f1 (ppm)

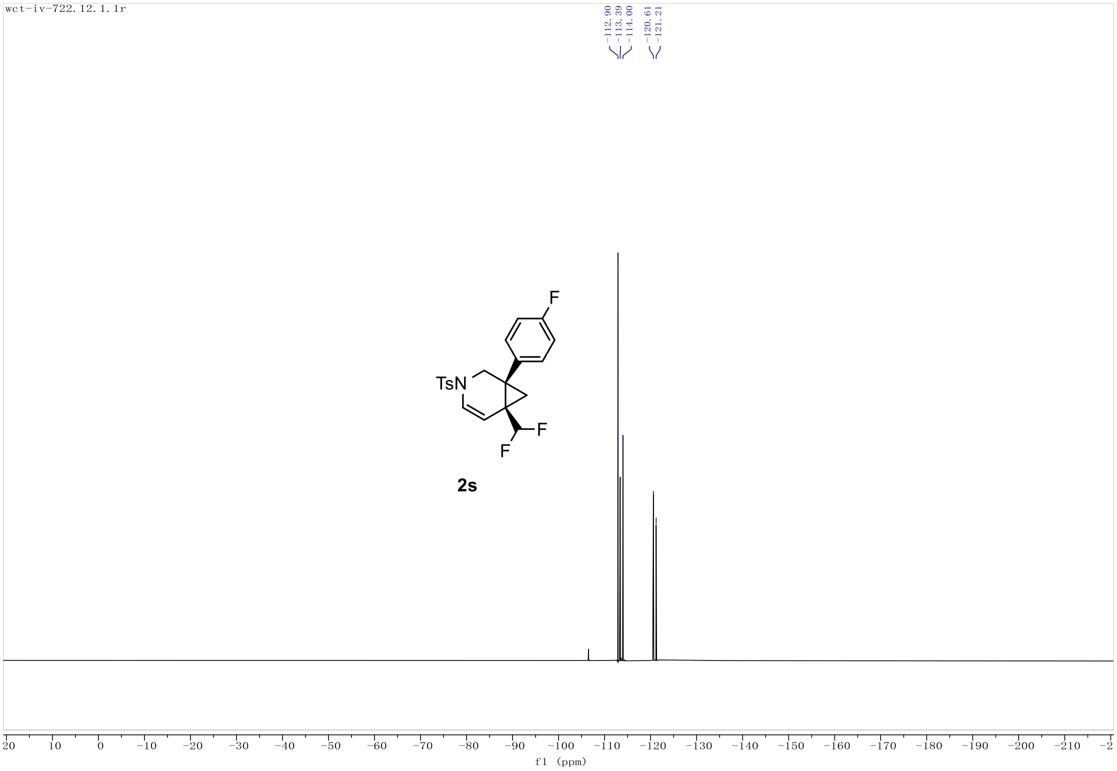
wct-iv-722.12.1.1r
-112.90
-113.39
-114.00
-120.61
-121.21
TsN
F
F
F
2s
f1 (ppm)

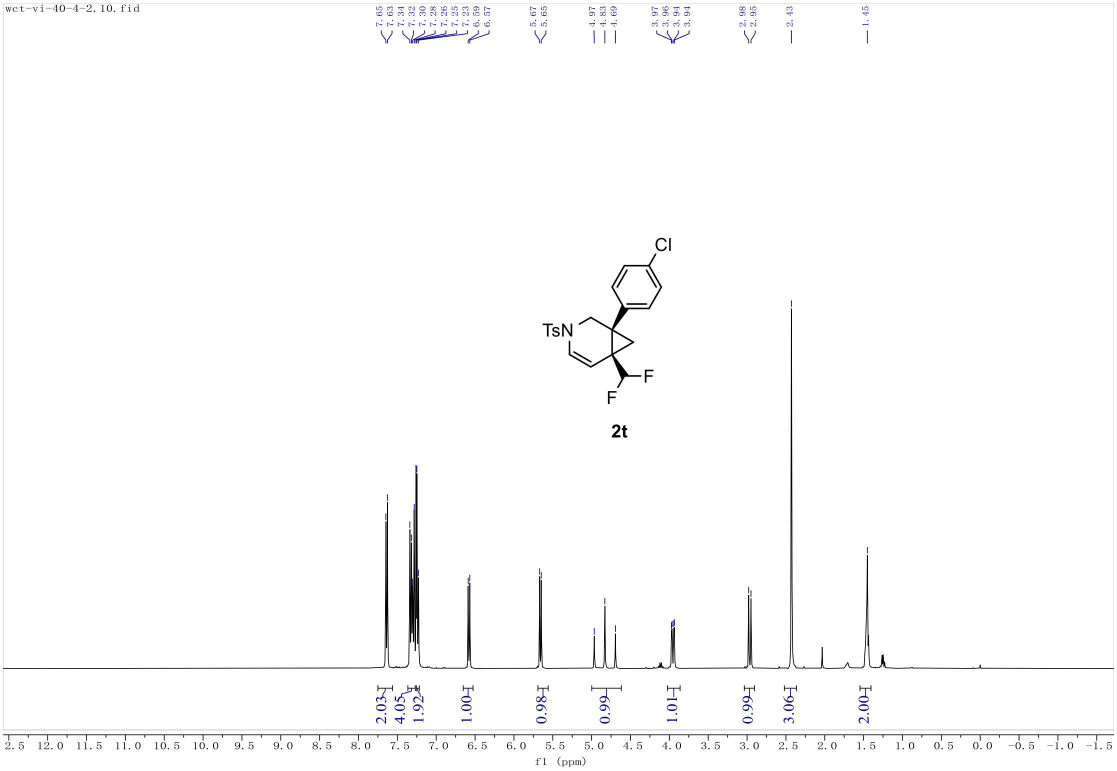
wct-vi-40-4-2.10.fid
2t
f1 (ppm)

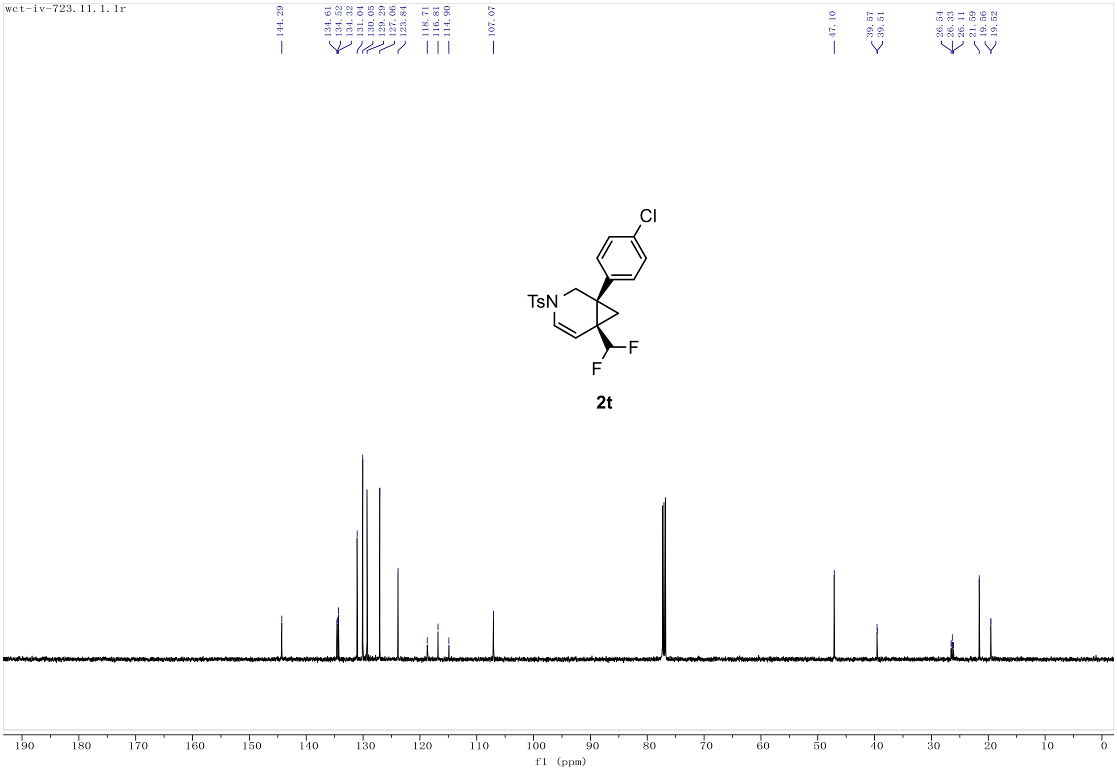
wct-iv-723.11.1.1r
2t
f1 (ppm)

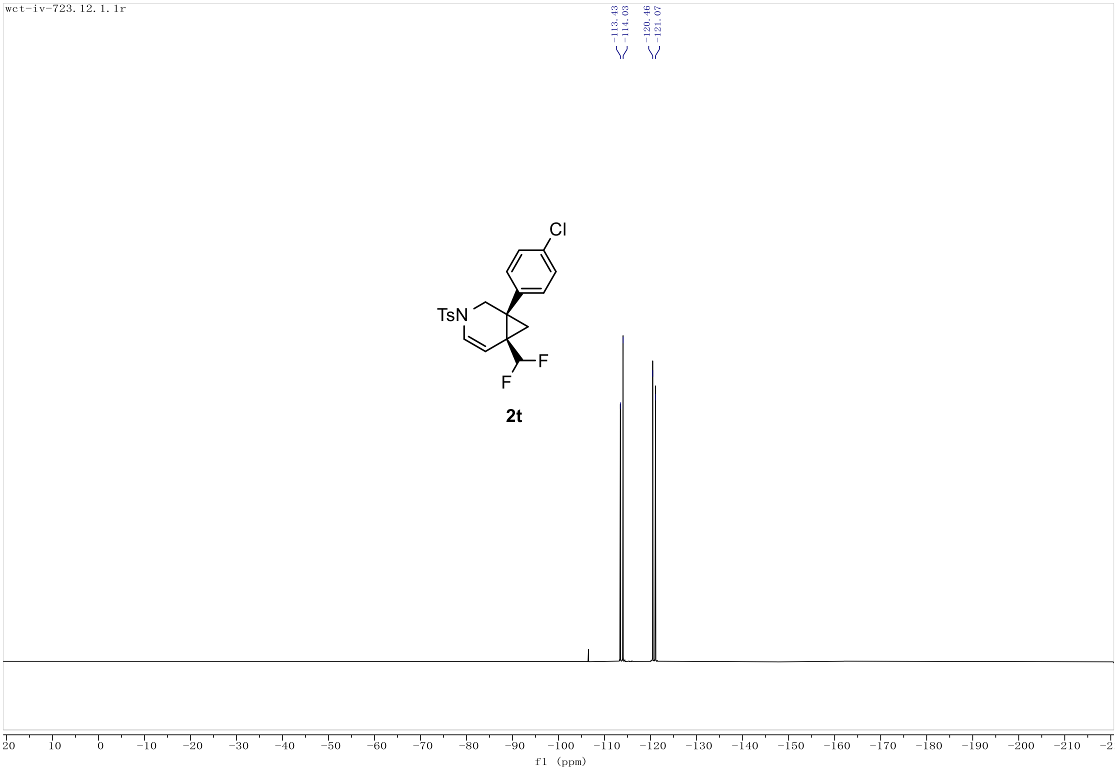
wct-iv-723.12.1.1r
-113.43
-114.03
-120.46
-121.07
Cl
TsN
F
F
2t
f1 (ppm)

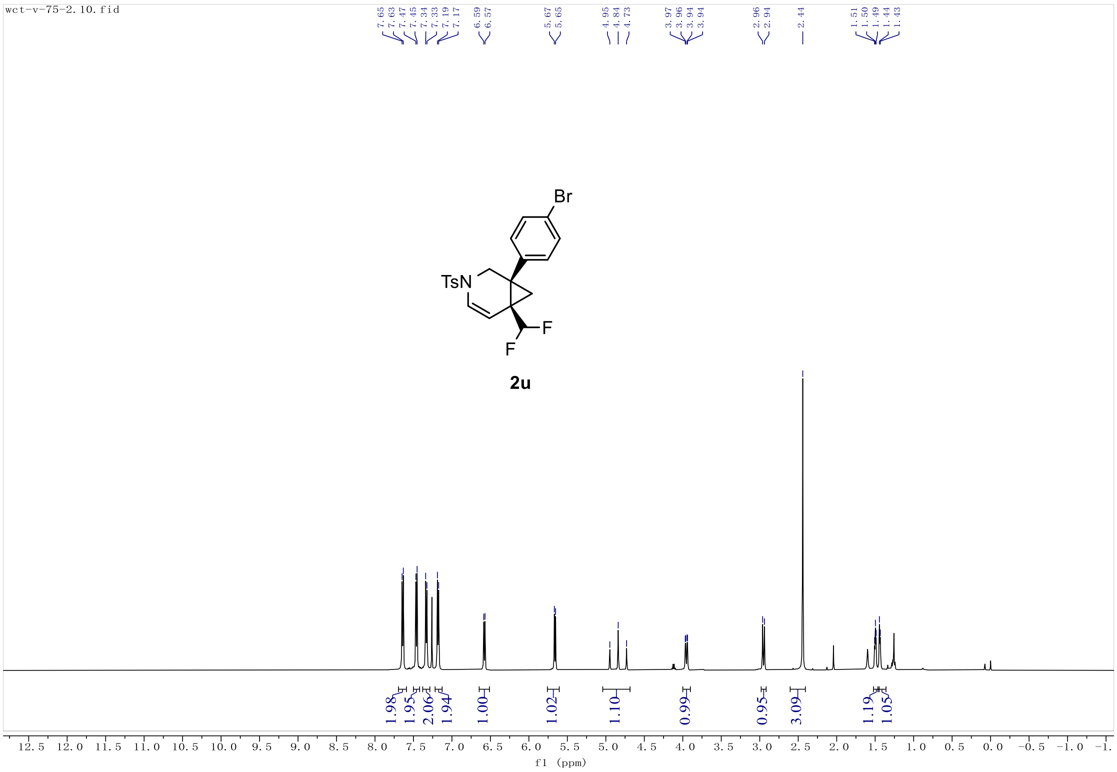
wct-v-75-2.10.fid
7.65
7.63
7.47
7.45
7.34
7.33
7.19
7.17
6.59
6.57
5.67
5.65
4.95
4.84
4.73
3.97
3.96
3.94
3.94
2.96
2.94
2.44
1.51
1.50
1.49
1.44
1.43
Br
TsN
F
F
2u
1.98
1.95
2.06
1.94
1.00
1.02
1.10
0.99
0.95
3.09
1.19
1.05
f1 (ppm)

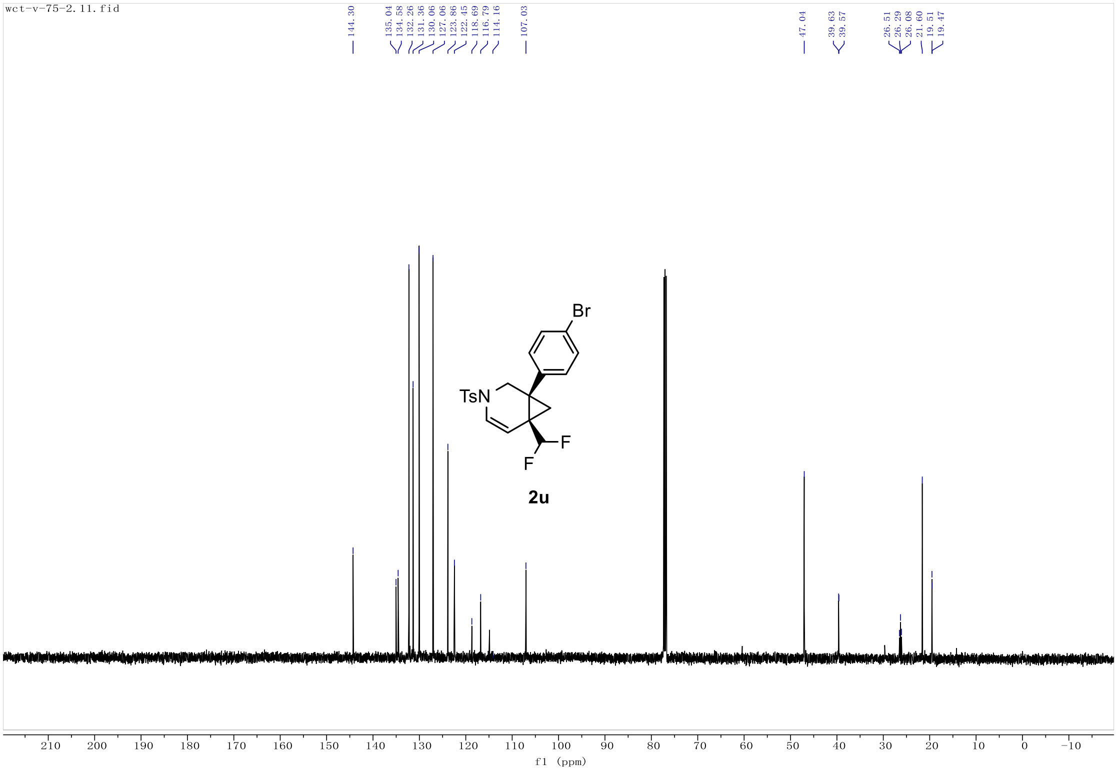
wct-v-75-2.11.fid
144.30
135.04
134.58
132.26
131.36
130.06
127.06
123.86
122.45
118.69
116.79
114.16
107.03
47.04
39.63
39.57
26.51
26.29
26.08
21.60
19.51
19.47
Br
TsN
F
F
2u
f1 (ppm)

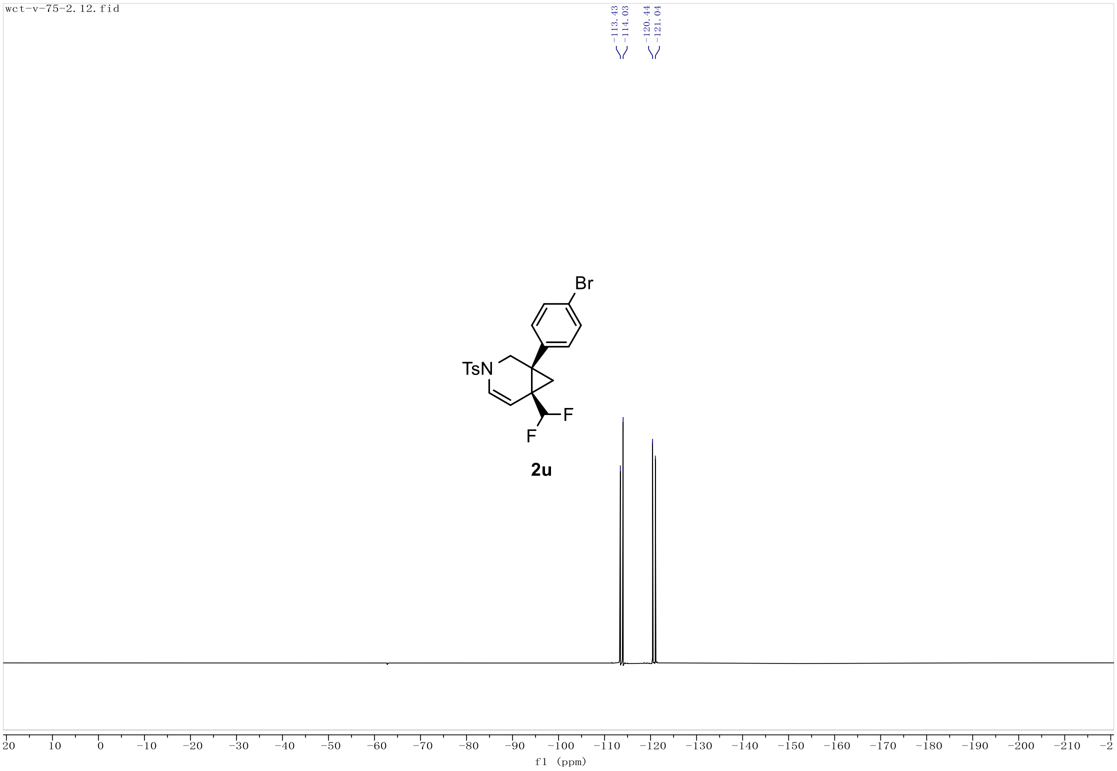
wct-v-75-2.12.fid
-113.43
-114.03
-120.44
-121.04
Br
TsN
F
F
2u
f1 (ppm)

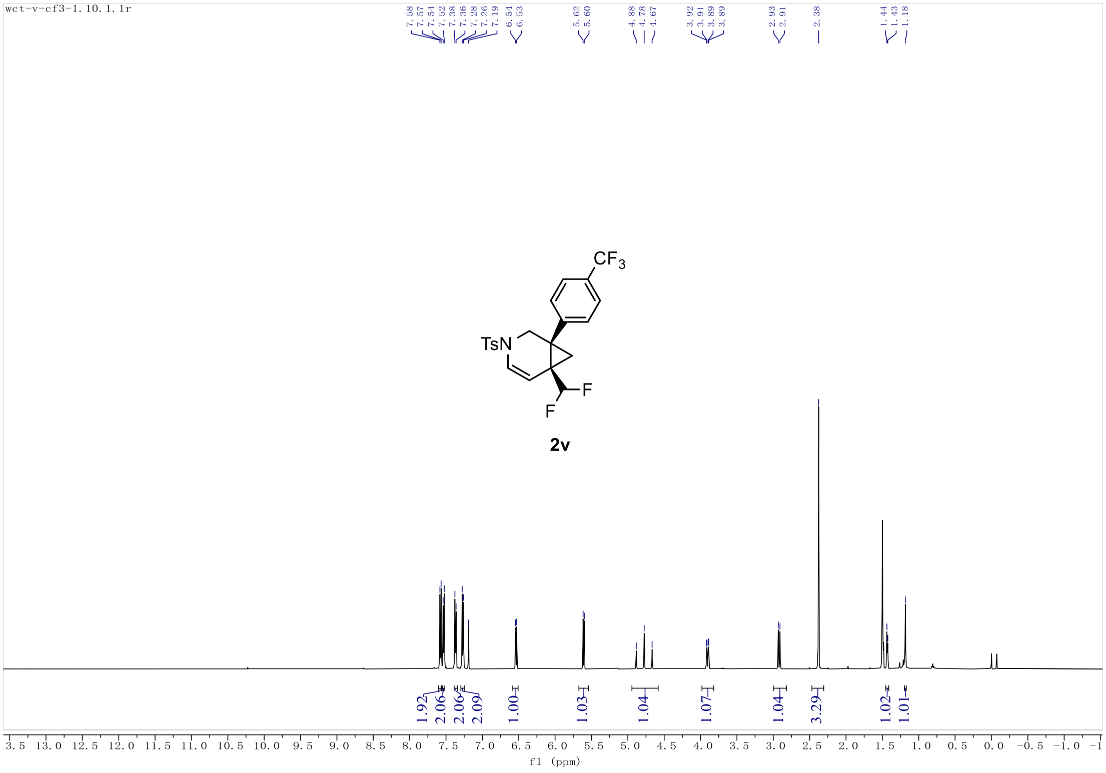
wct-v-cf3-1.10.1.1r
7.58 7.57 7.54 7.52 7.38 7.36 7.28 7.26 7.19 6.54 6.53 5.62 5.60 4.88 4.78 4.67 3.92 3.91 3.89 3.89 2.93 2.91 2.38 1.44 1.43 1.18
CF3
TsN
F
F
2v
1.92 2.06 2.06 2.09 1.00 1.03 1.04 1.07 1.04 3.29 1.02 1.01
f1 (ppm)

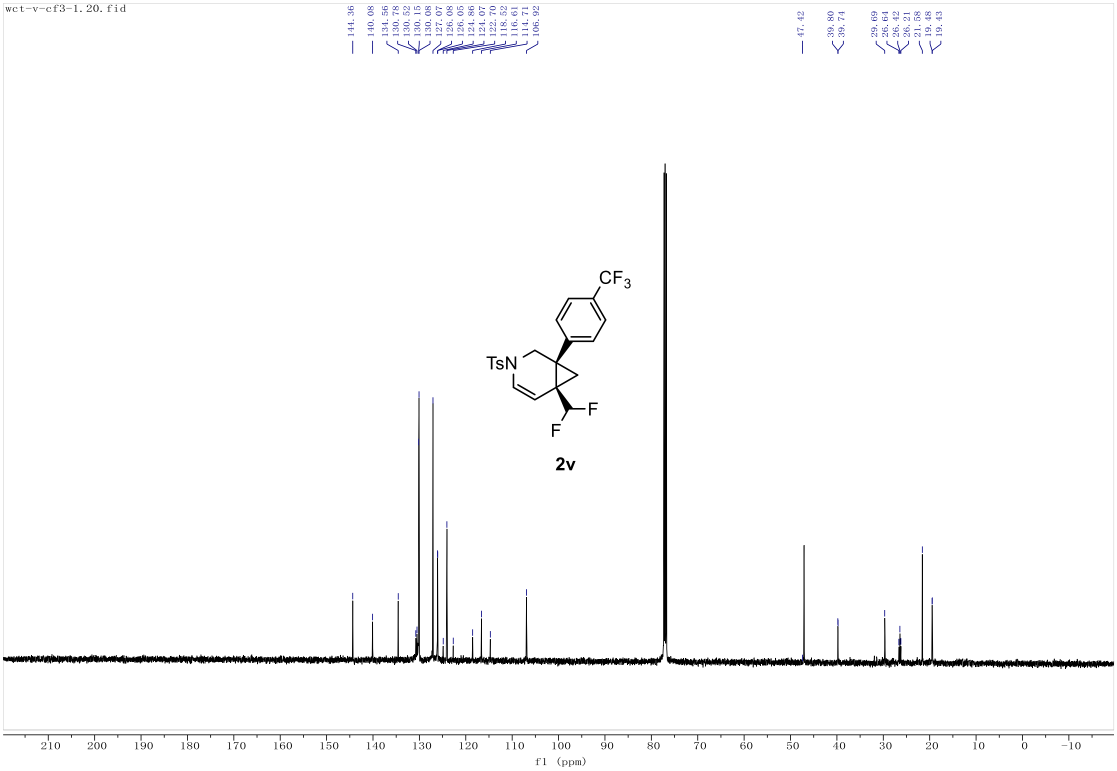
wct-v-cf3-1.20.fid
144.36 140.08 134.56 130.78 130.52 130.15 130.08 127.07 126.08 126.05 124.86 124.07 122.70 118.52 116.61 114.71 106.92 47.42 39.80 39.74 29.69 26.64 26.42 26.21 21.58 19.48 19.43
CF3
TsN
F
F
2v
f1 (ppm)

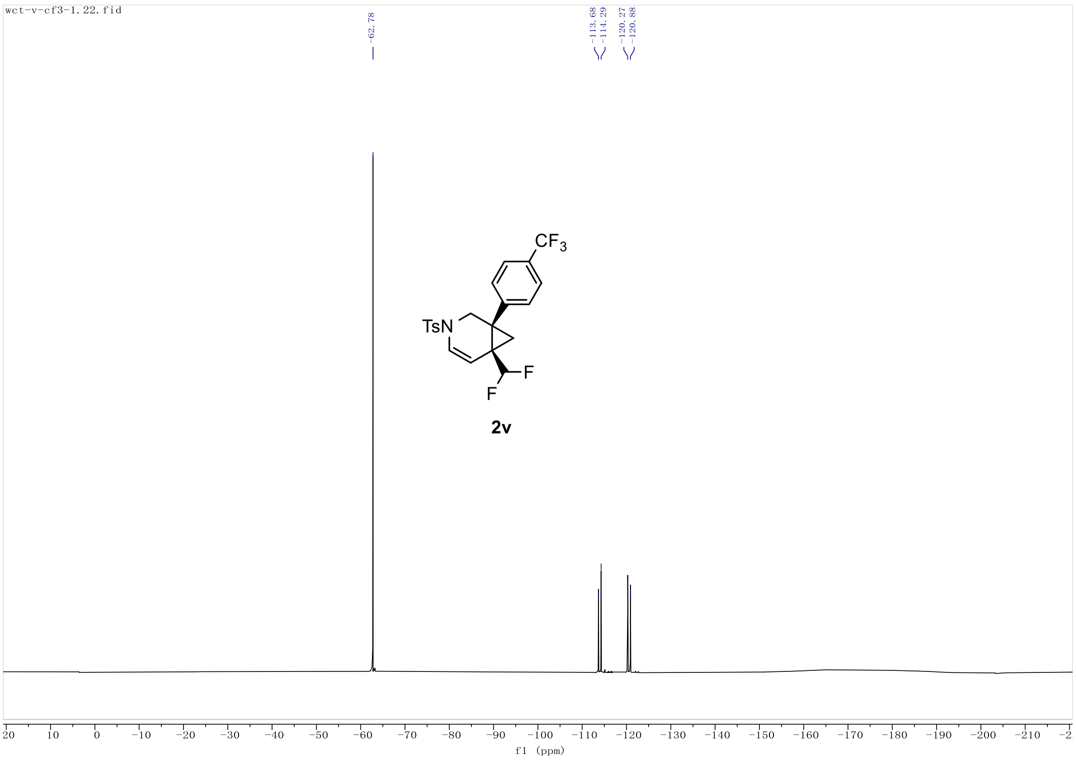
wct-v-cf3-1.22.fid
CF3
TsN
F
F
2v
f1 (ppm)

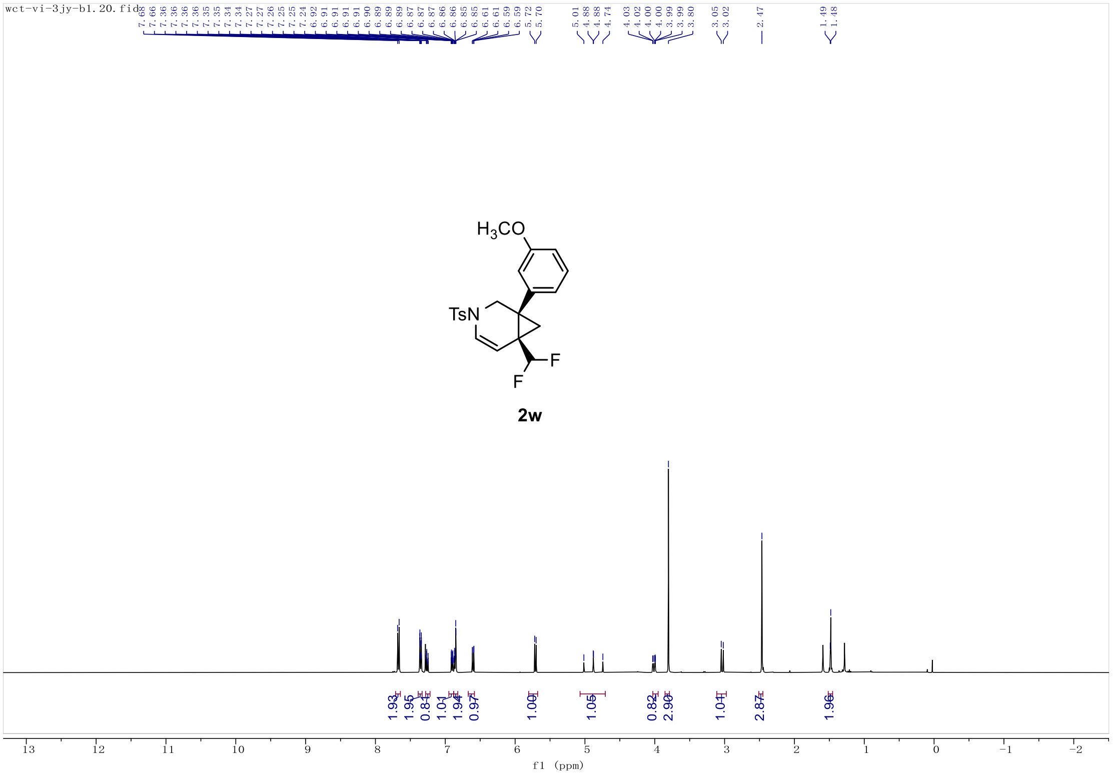
wct-vi-3jy-b1.20.fid
H3CO
TsN
F
F
2w
f1 (ppm)

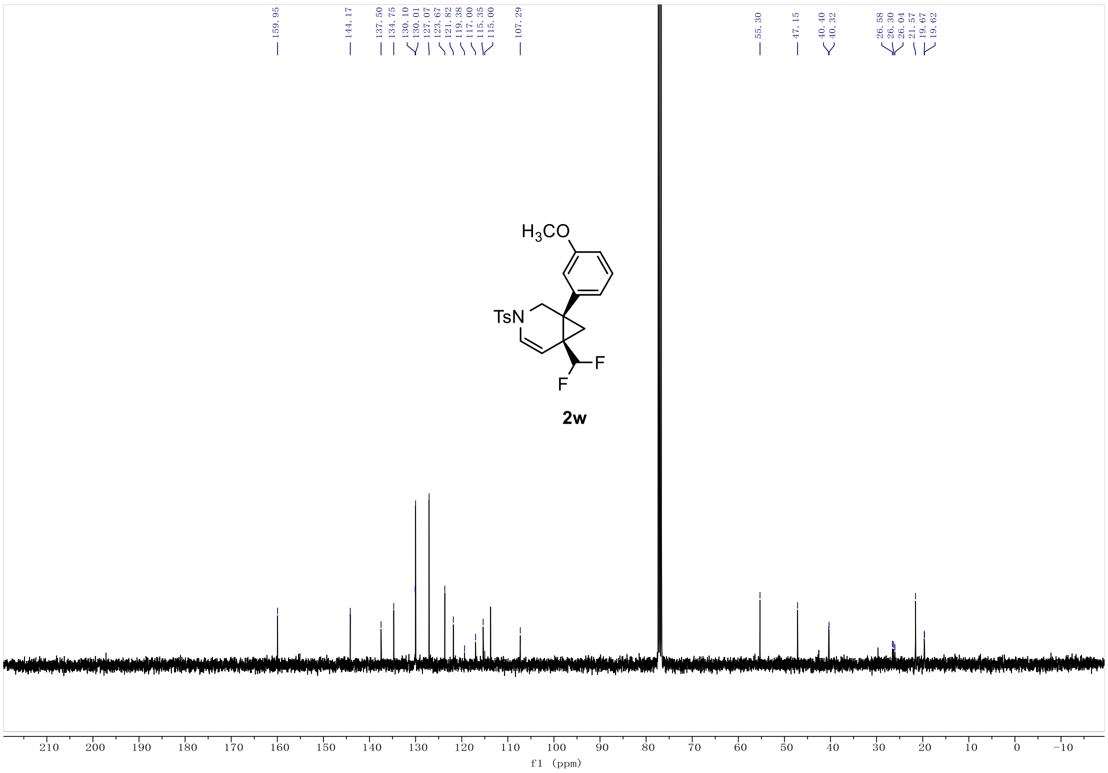
159.95
144.17
137.50
134.75
130.10
130.01
127.07
123.67
121.82
119.38
117.00
115.35
115.00
107.29
55.30
47.15
40.40
40.32
26.58
26.30
26.04
21.57
19.67
19.62
H3CO
TsN
F
F
2w
f1 (ppm)

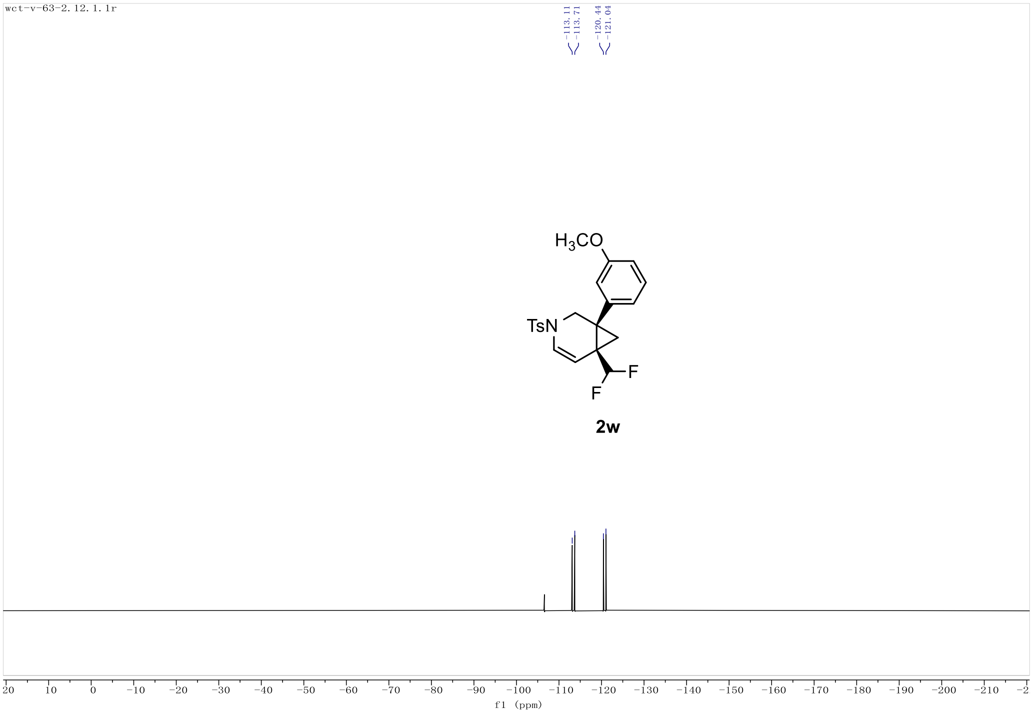
wct-v-63-2.12.1.1r
-113.11
-113.71
-120.44
-121.04
H3CO
TsN
F
F
2w
f1 (ppm)

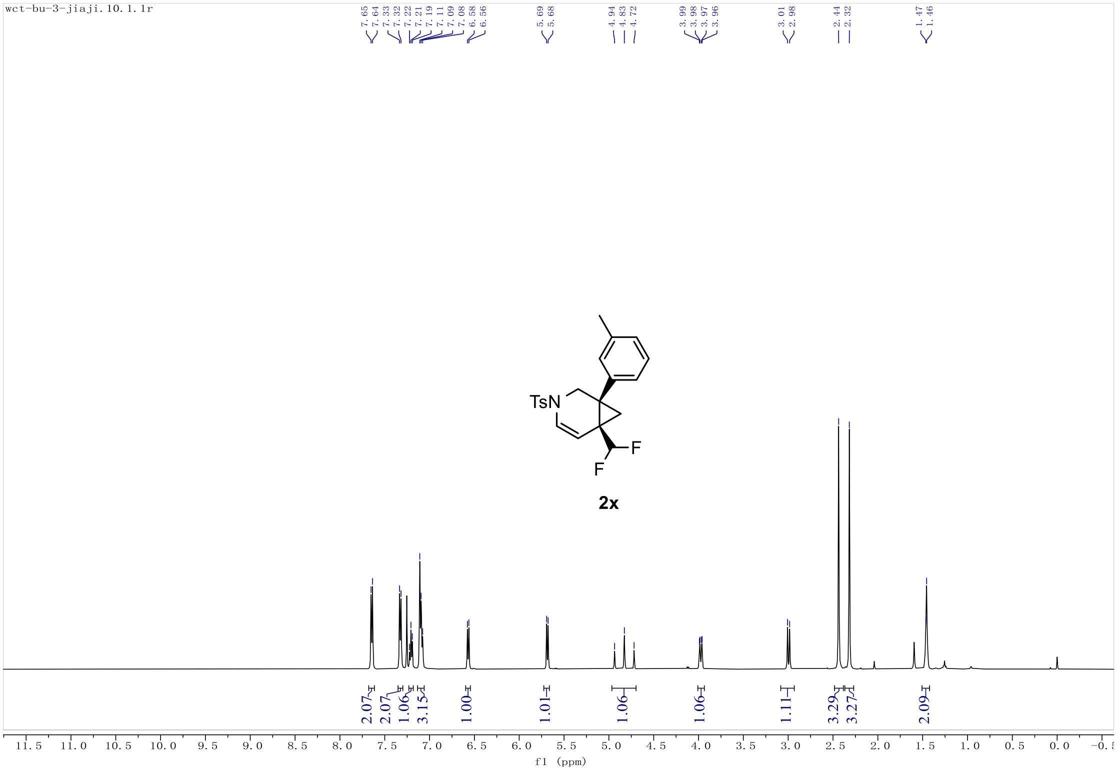
wct-bu-3-jiaji.10.1.1r
7.65
7.64
7.33
7.32
7.22
7.21
7.19
7.11
7.09
7.08
6.58
6.56
5.69
5.68
4.94
4.83
4.72
3.99
3.98
3.97
3.96
3.01
2.98
2.44
2.32
1.47
1.46
TsN
F
F
2x
2.07
2.07
1.06
3.15
1.00
1.01
1.06
1.06
1.11
3.29
3.27
2.09
f1 (ppm)

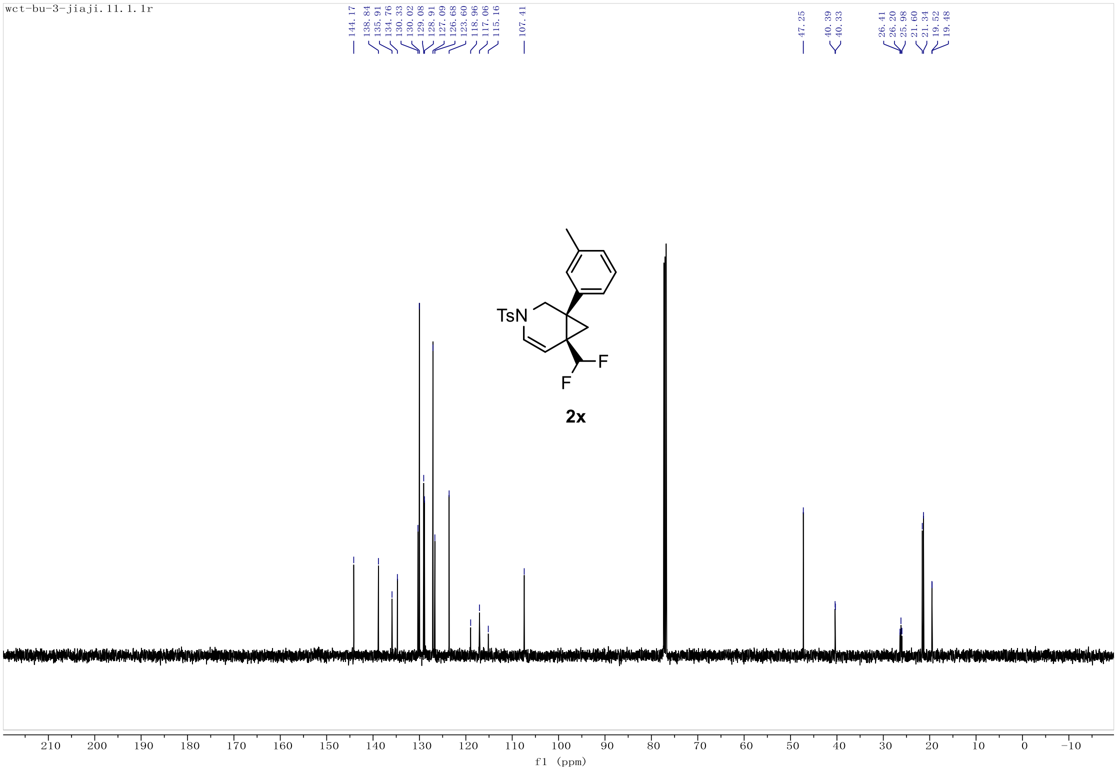
wct-bu-3-jiaji.11.1.1r
144.17
138.84
135.91
134.76
130.33
130.02
129.08
128.91
127.09
126.68
123.60
118.96
117.06
115.16
107.41
47.25
40.39
40.33
26.41
26.20
25.98
21.60
21.34
19.52
19.48
TsN
F
F
2x
f1 (ppm)

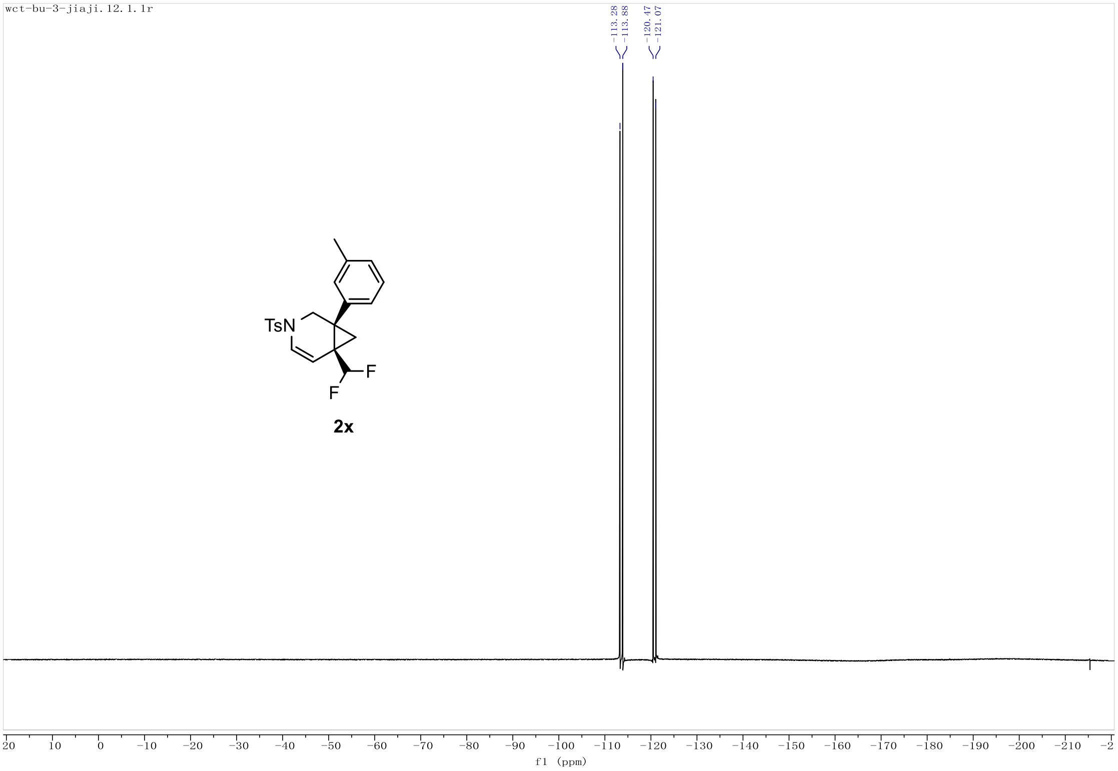
wct-bu-3-jiaji.12.1.1r
-113.28
-113.88
-120.47
-121.07
TsN
F
F
2x
f1 (ppm)

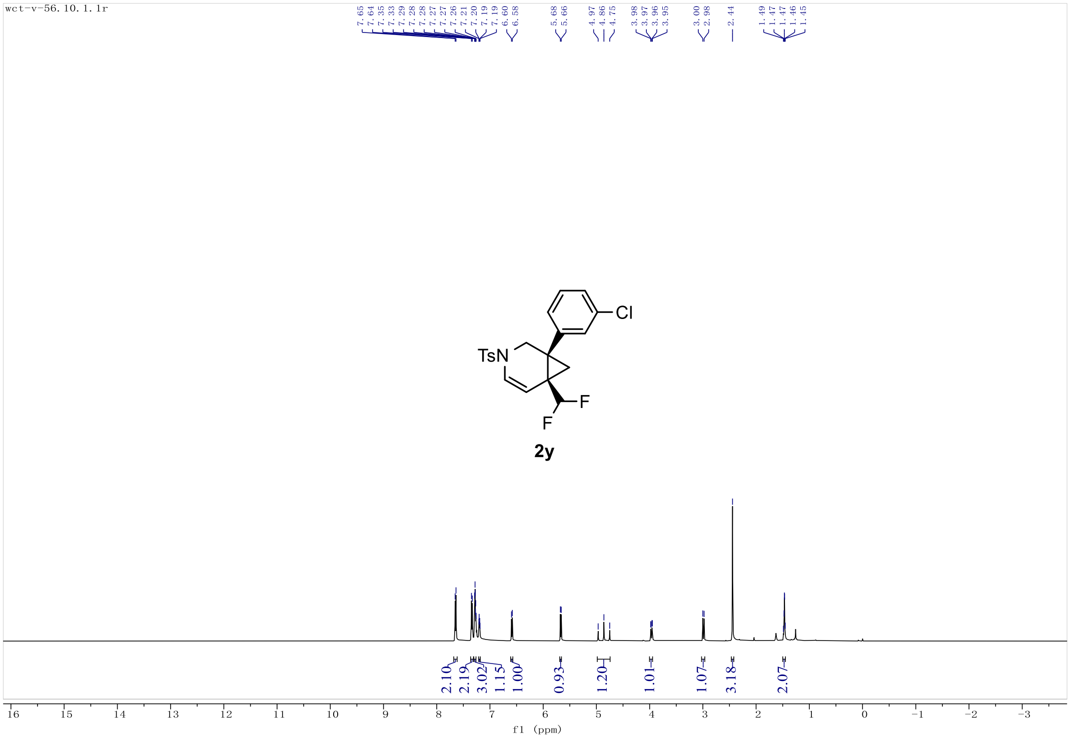
wct-v-56.10.1.1r
7.65 7.64 7.35 7.33 7.29 7.28 7.28 7.27 7.27 7.26 7.21 7.20 7.19 7.19 6.60 6.58
5.68 5.66
4.97 4.86 4.75
3.98 3.97 3.96 3.95
3.00 2.98
2.44
1.49 1.47 1.47 1.46 1.45
Cl
TsN
F
F
2y
2.10 2.19 3.02 1.15 1.00 0.93 1.20 1.01 1.07 3.18 2.07
f1 (ppm)

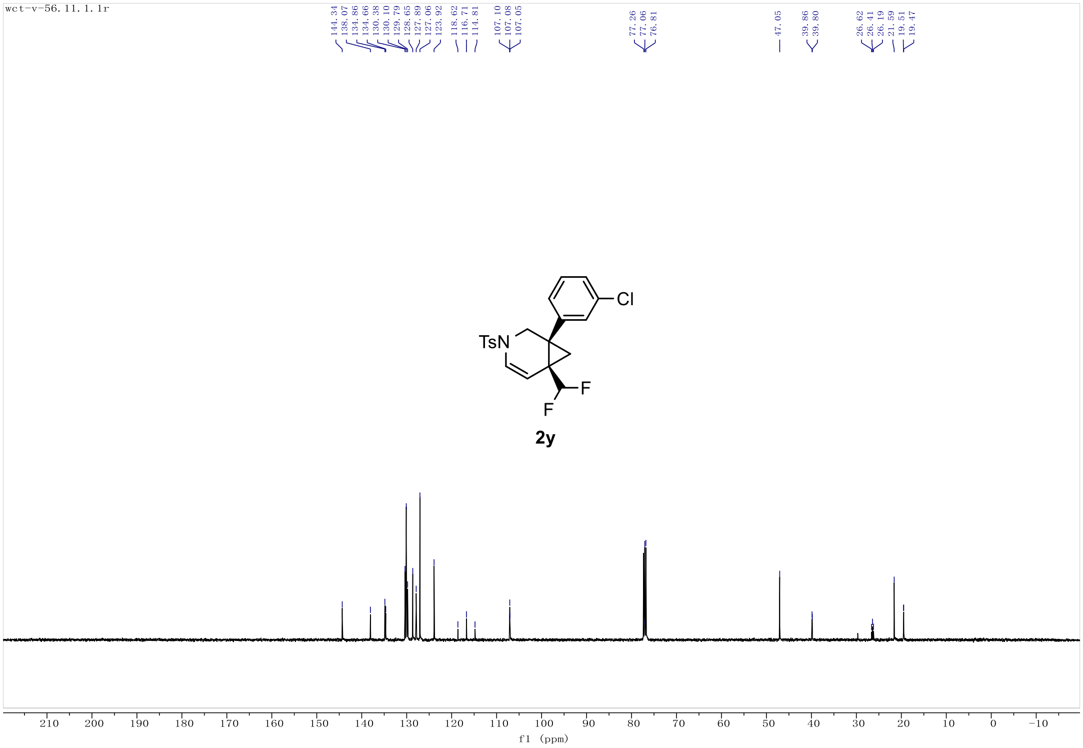
wct-v-56.11.1.1r
144.34
138.07
134.86
134.66
130.38
130.10
129.79
128.65
127.89
127.06
123.92
118.62
116.71
114.81
107.10
107.08
107.05
77.26
77.06
76.81
47.05
39.86
39.80
26.62
26.41
26.19
21.59
19.51
19.47
TsN
Cl
F
F
2y
f1 (ppm)

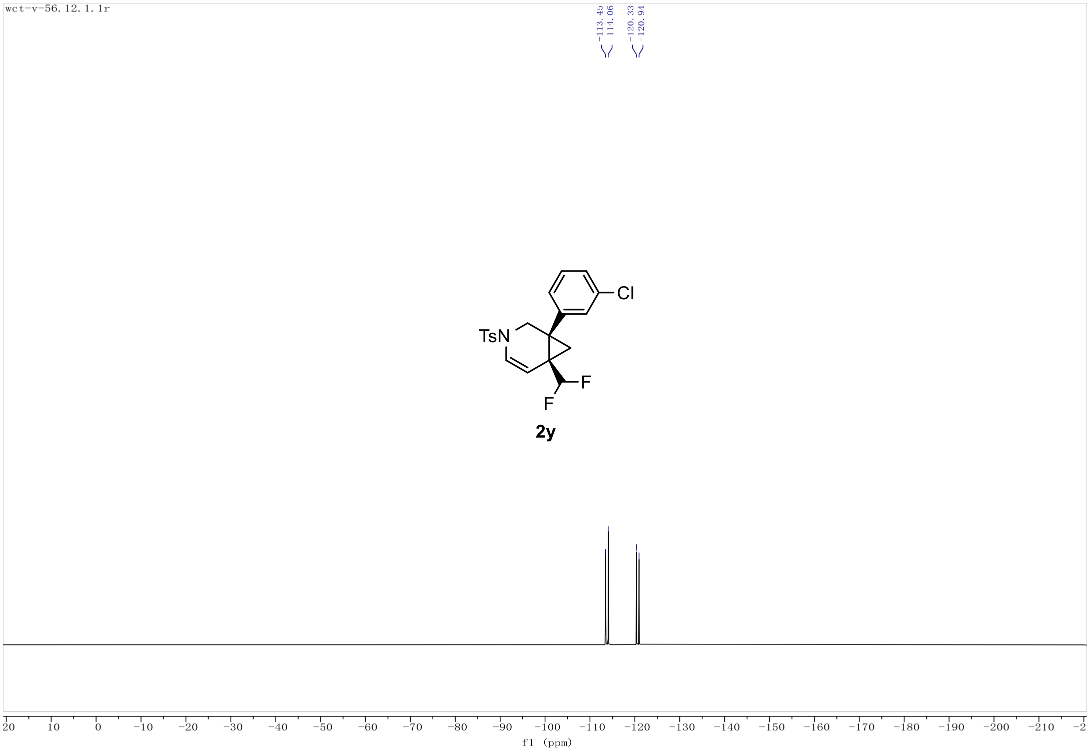
wct-v-56.12.1.1r
-113.45
-114.06
-120.33
-120.94
TsN
Cl
F
F
2y
f1 (ppm)

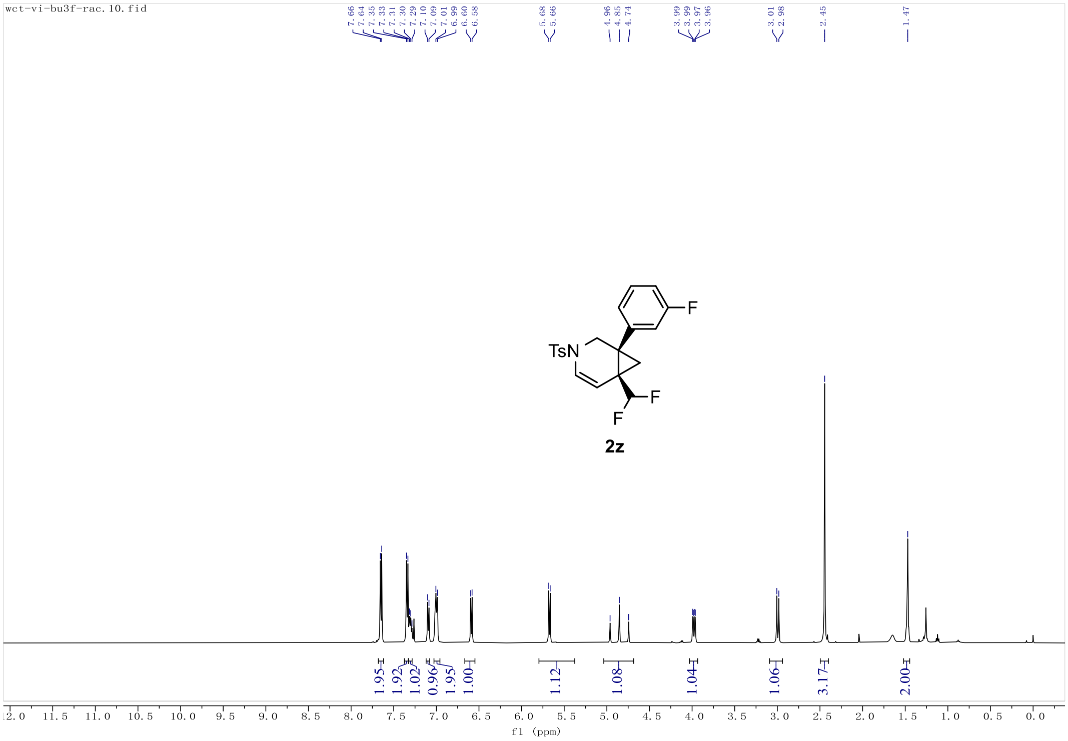
wct-vi-bu3f-rac.10.fid
7.66 7.64 7.35 7.33 7.31 7.30 7.29 7.10 7.09 7.01 6.99 6.60 6.58
5.68 5.66
4.96 4.85 4.74
3.99 3.99 3.97 3.96
3.01 2.98
2.45
1.47
TsN
F
F
F
2z
1.95 1.92 1.02 0.96 1.95 1.00 1.12 1.08 1.04 1.06 3.17 2.00
f1 (ppm)

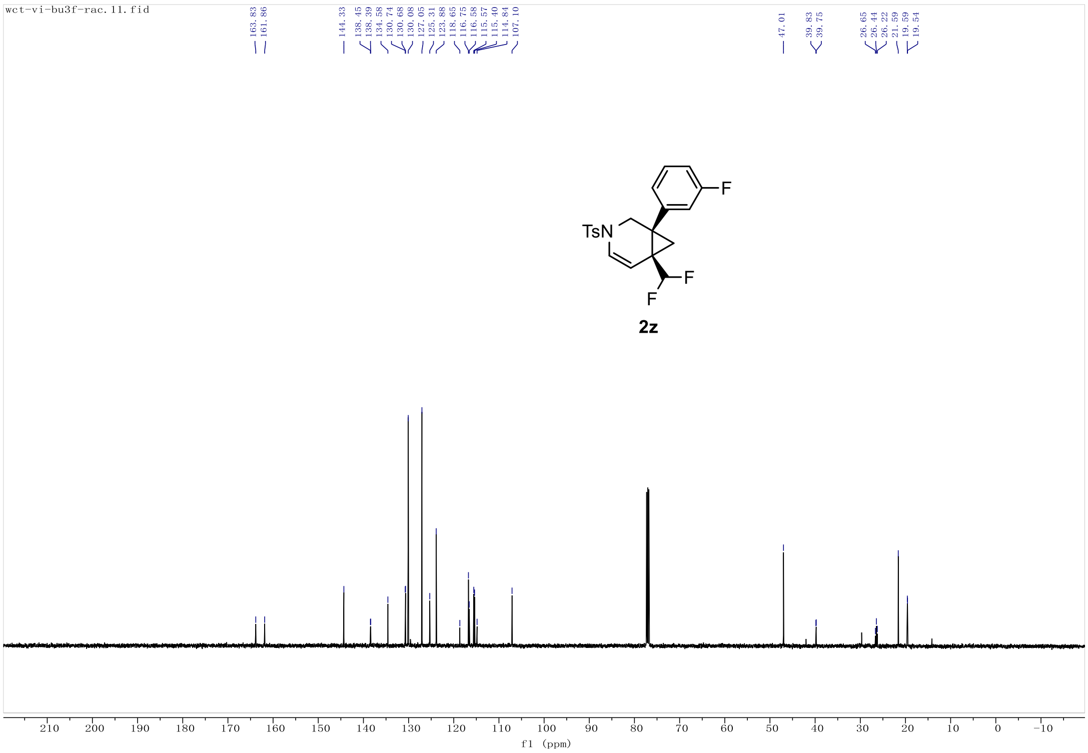
wct-vi-bu3f-rac.11.fid
163.83 161.86
144.33 138.45 138.39 134.58 130.74 130.68 130.08 127.65 125.31 123.88 118.65 116.75 116.58 115.57 115.40 114.84 107.10
47.01
39.83 39.75
26.65 26.44 26.22 21.59 19.59 19.54
TsN
F
F
F
2z
f1 (ppm)

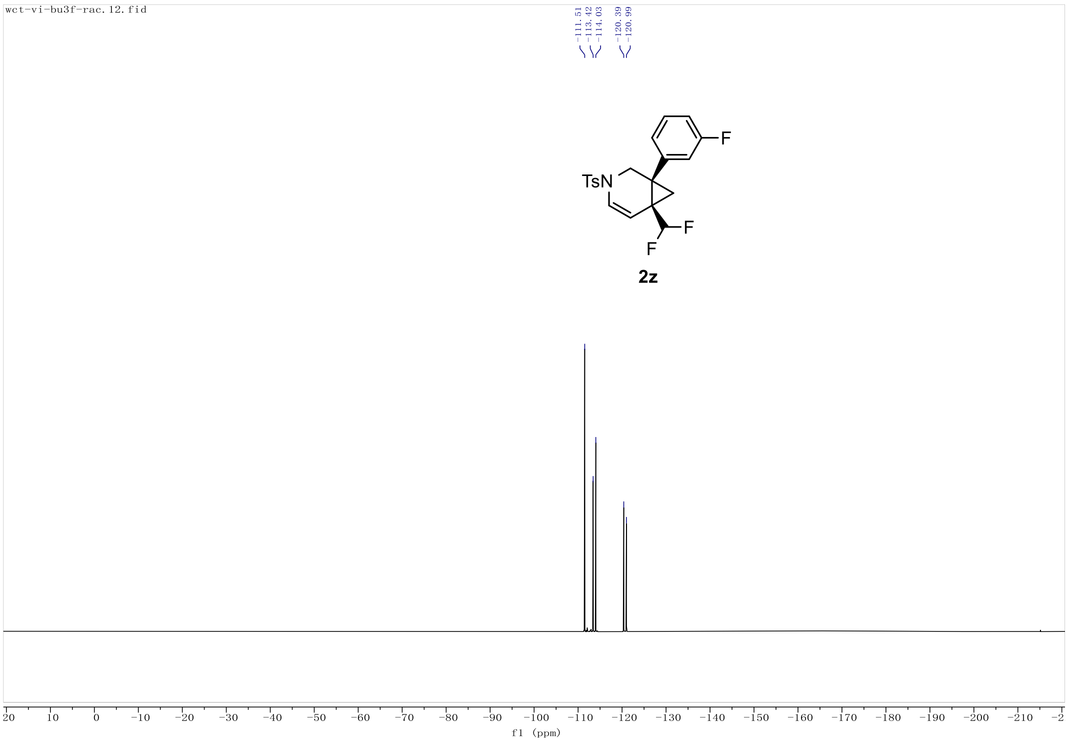
wct-vi-bu3f-rac.12.fid
-111.51
-113.42
-114.03
-120.39
-120.99
TsN
F
F
F
f1 (ppm)

2z

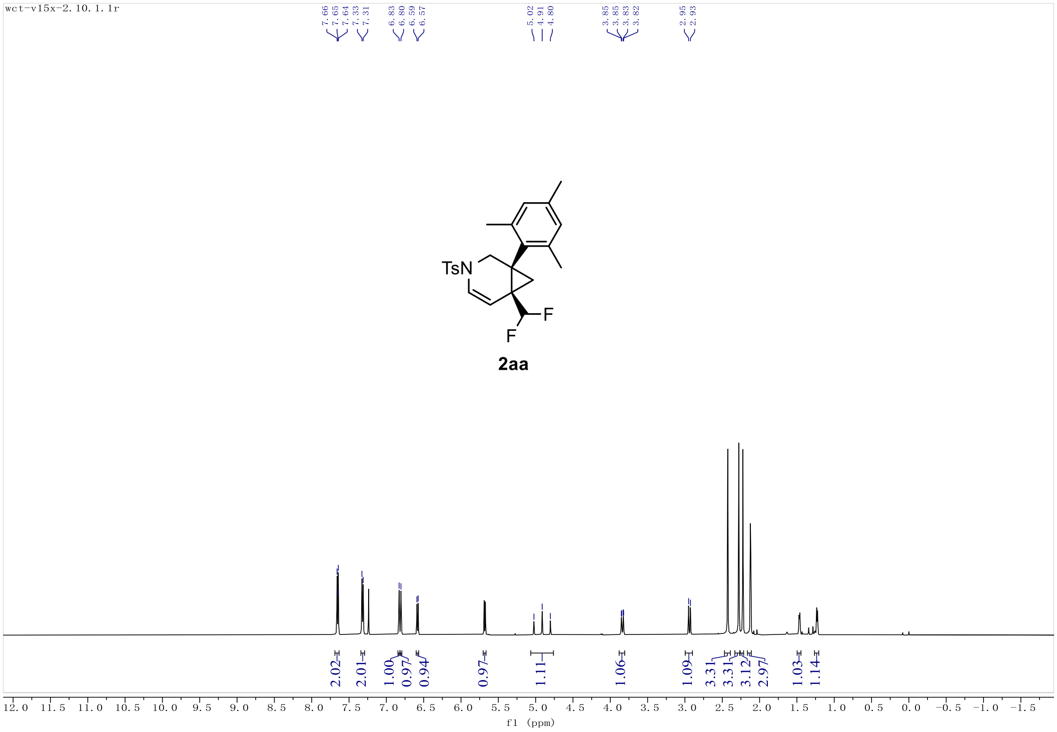
wct-v15x-2.10.1.1r
TsN
F
F
f1 (ppm)

2aa

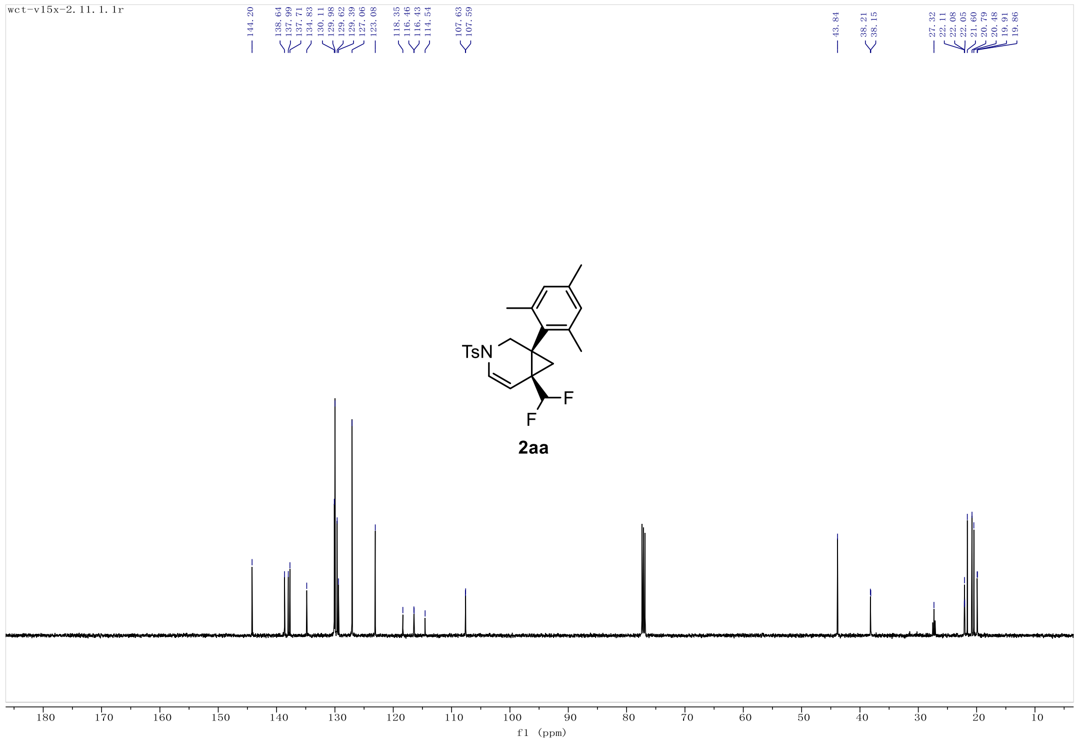

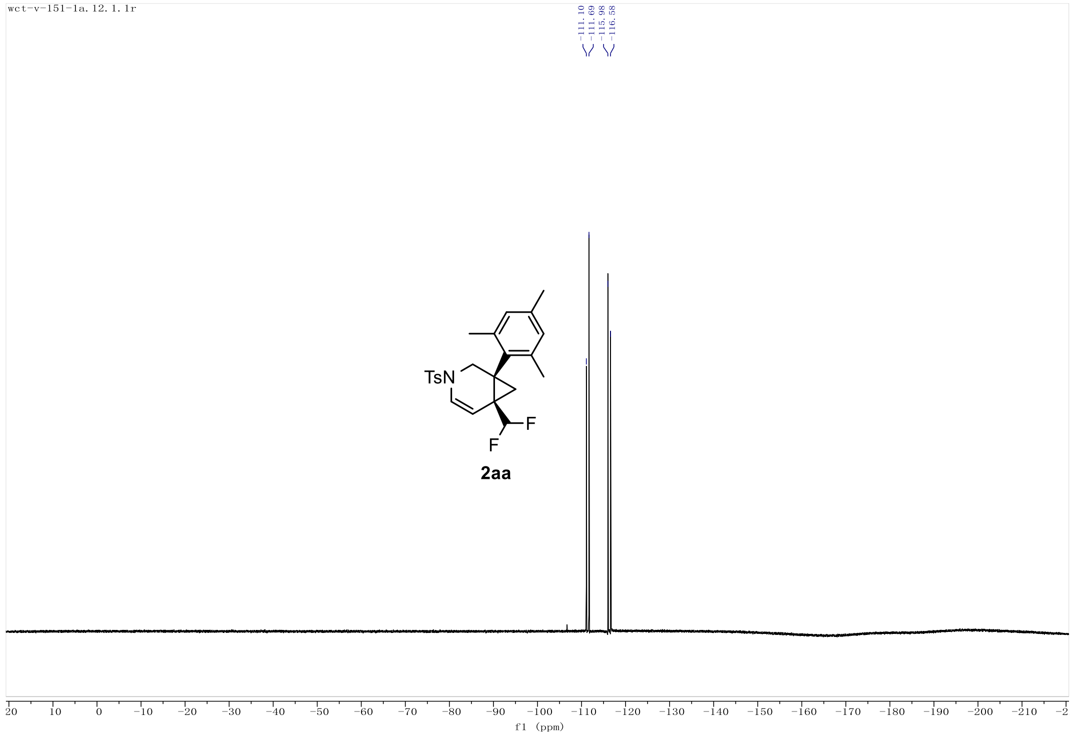

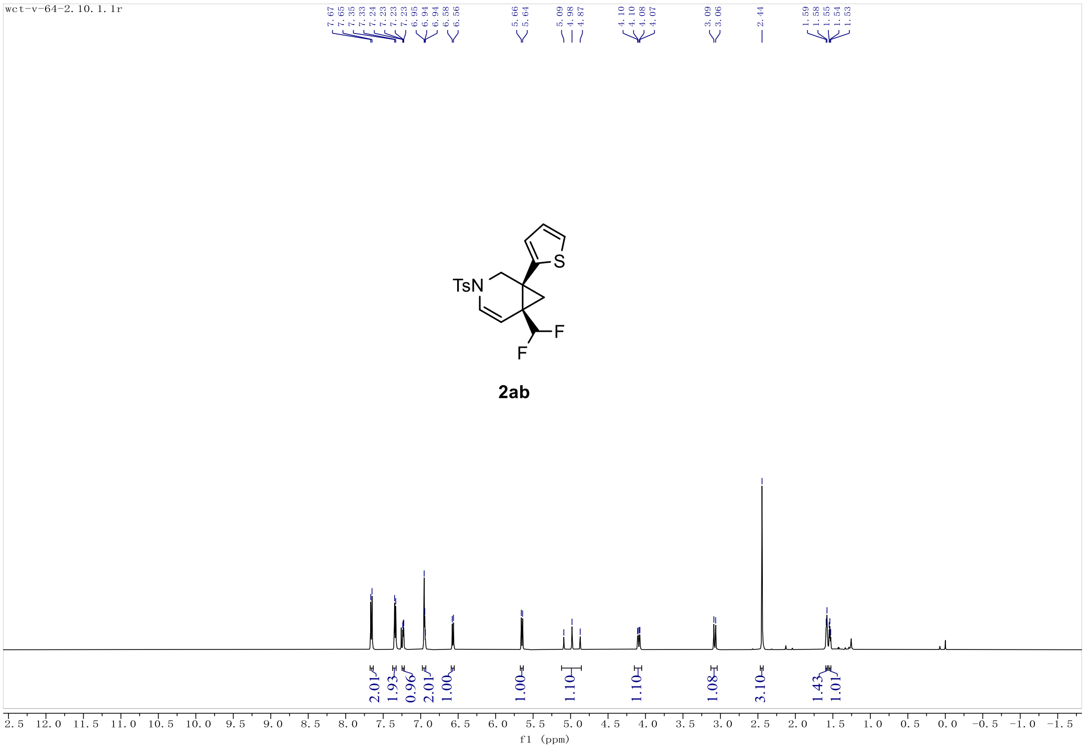
wct-v-64-2.10.1.1r
TsN
S
F
F
2ab
f1 (ppm)

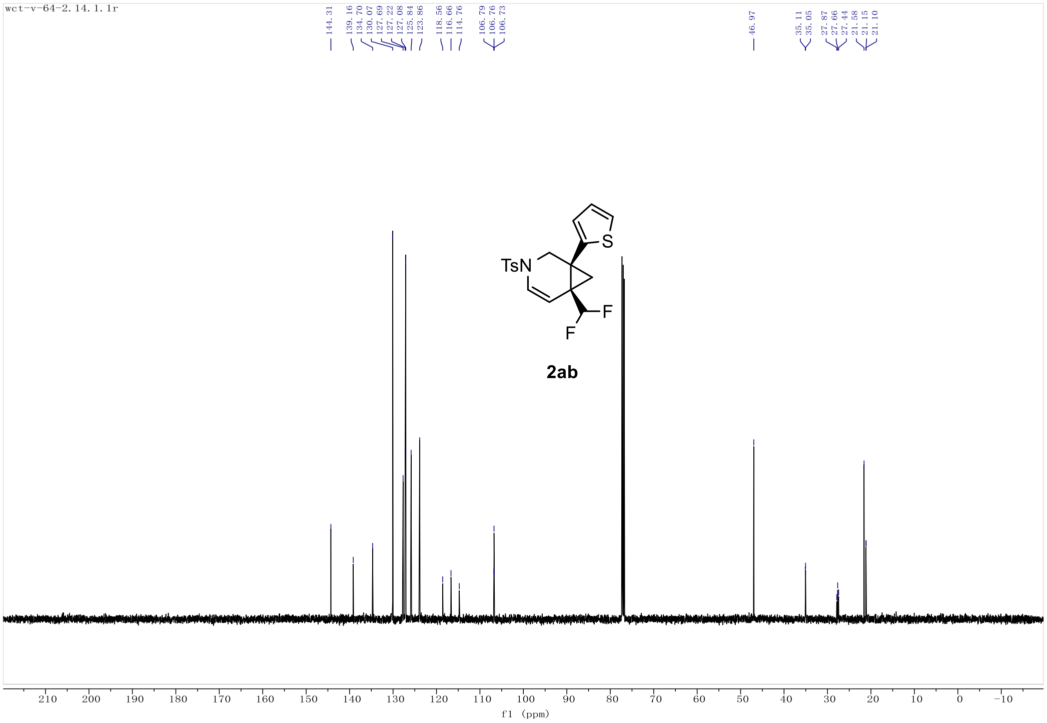
wct-v-64-2.14.1.1r
TsN
S
F
F
2ab
f1 (ppm)

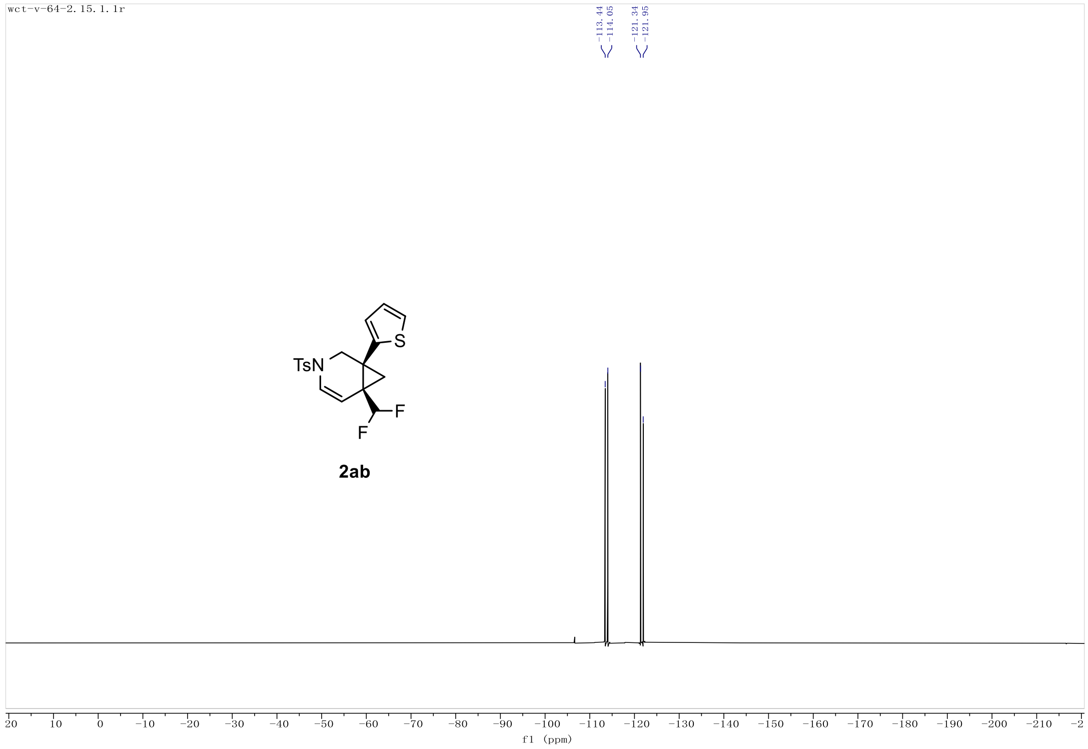
wct-v-64-2.15.1.1r
-113.44
-114.05
-121.34
-121.95
TsN
S
F
F
2ab
f1 (ppm)

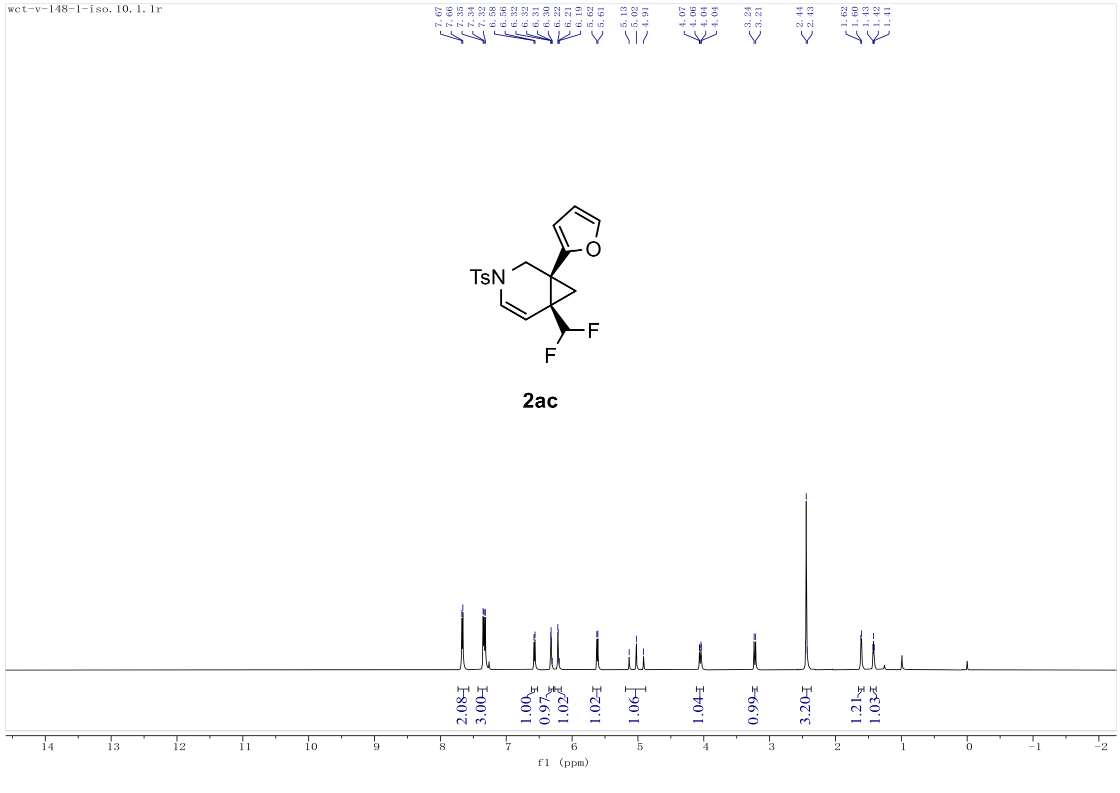
wct-v-148-1-iso.10.1.1r
7.67
7.66
7.35
7.34
7.32
6.58
6.56
6.32
6.32
6.31
6.30
6.22
6.21
6.19
5.62
5.61
5.13
5.02
4.91
4.07
4.06
4.04
4.04
3.24
3.21
2.44
2.43
1.62
1.60
1.43
1.42
1.41
TsN
O
F
F
2ac
2.08
3.00
1.00
0.97
1.02
1.02
1.06
1.04
0.99
3.20
1.21
1.03
f1 (ppm)

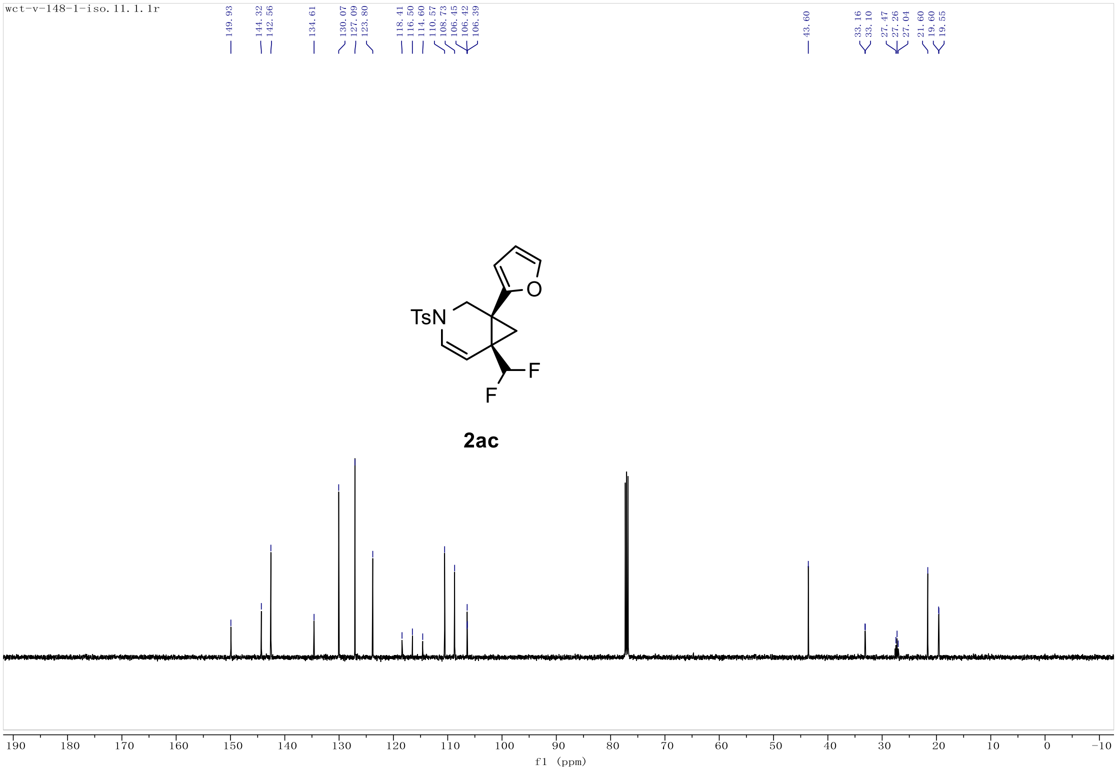
wct-v-148-1-iso.11.1.1r
149.93
144.32
142.56
134.61
130.07
127.09
123.80
118.41
116.50
114.60
110.57
108.73
106.45
106.42
106.39
43.60
33.16
33.10
27.47
27.26
27.04
21.60
19.60
19.55
TsN
O
F
2ac
f1 (ppm)

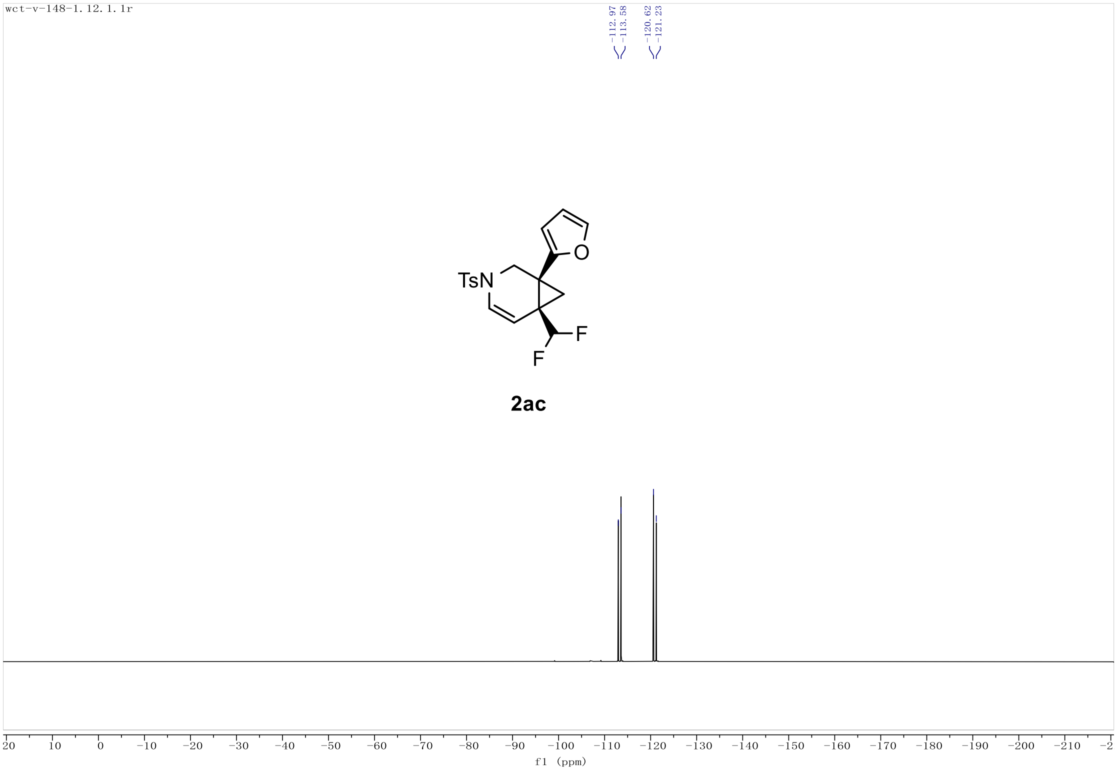
wct-v-148-1.12.1.1r
-112.97
-113.58
-120.62
-121.23
TsN
O
F
2ac
f1 (ppm)

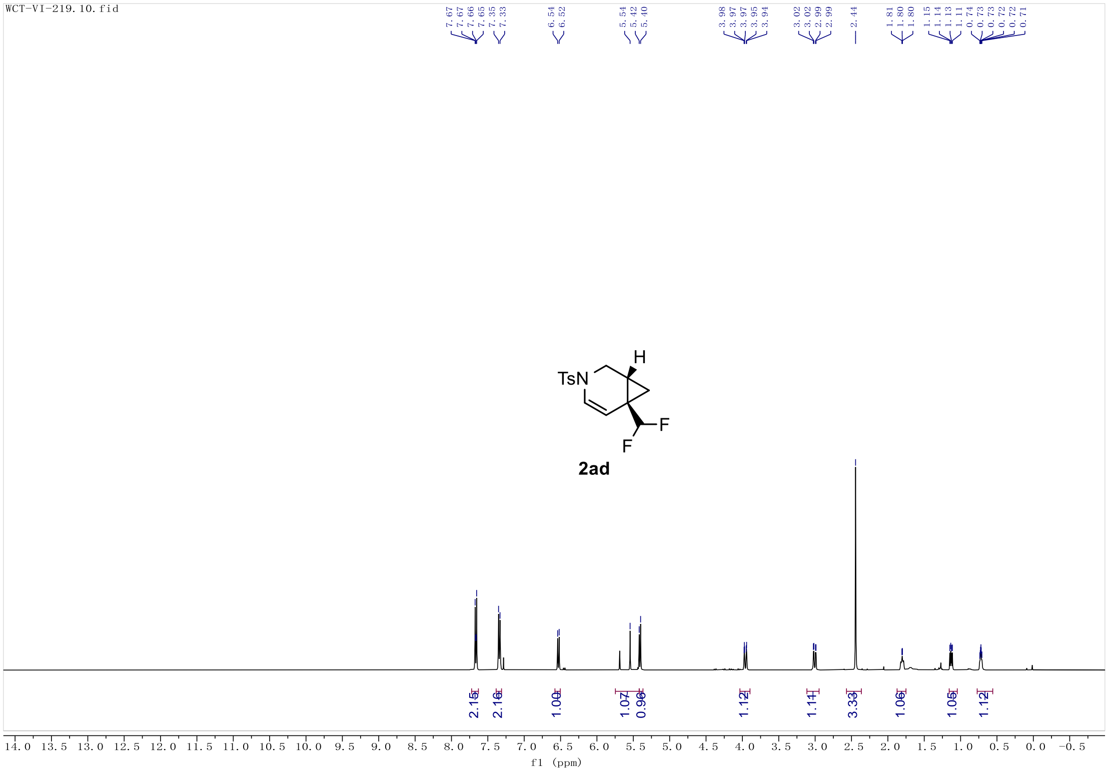
WCT-VI-219.10.fid
7.67 7.67 7.66 7.65 7.35 7.33 6.54 6.52 5.54 5.42 5.40 3.98 3.97 3.97 3.95 3.94 3.02 3.02 2.99 2.99 2.44 1.81 1.80 1.80 1.15 1.14 1.13 1.11 0.74 0.73 0.73 0.72 0.71
TsN
H
F
F
2ad
2.15 2.16 1.00 1.07 0.96 1.12 1.11 3.33 1.06 1.05 1.12
f1 (ppm)

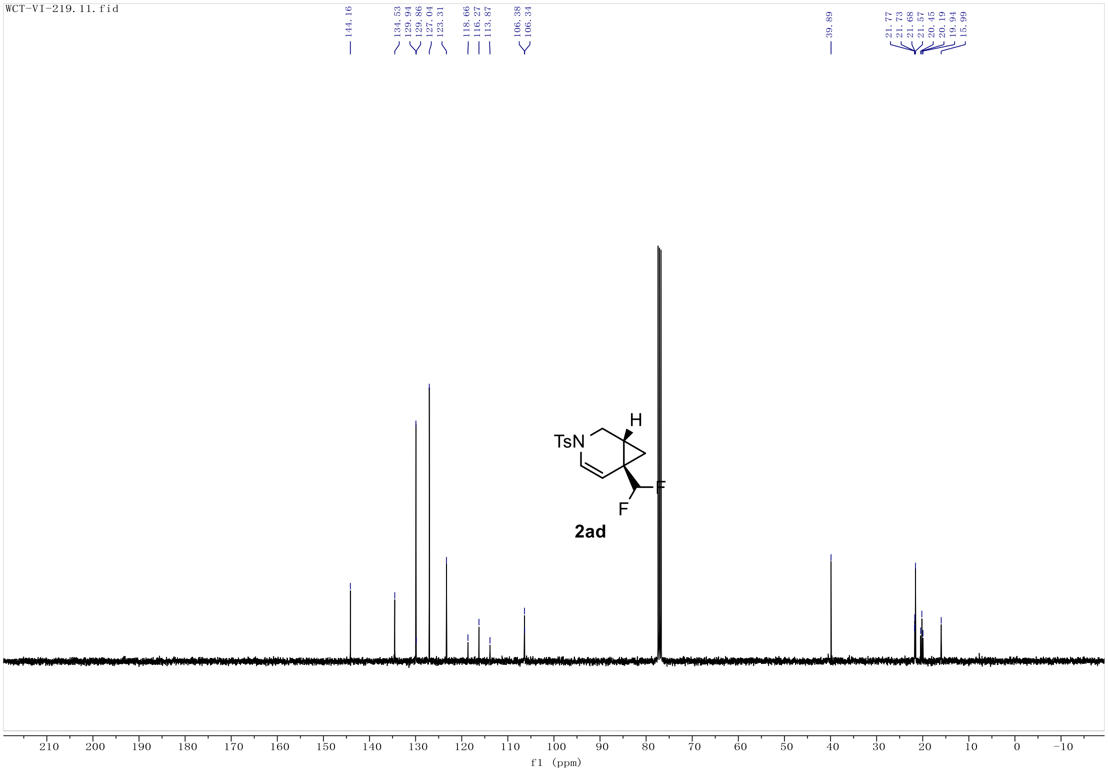
WCT-VI-219.11.fid
144.16 134.53 129.94 129.86 127.04 123.31 118.66 116.27 113.87 106.38 106.34 39.89 21.77 21.73 21.68 21.57 20.45 20.19 19.94 15.99
TsN
H
F
F
2ad
f1 (ppm)

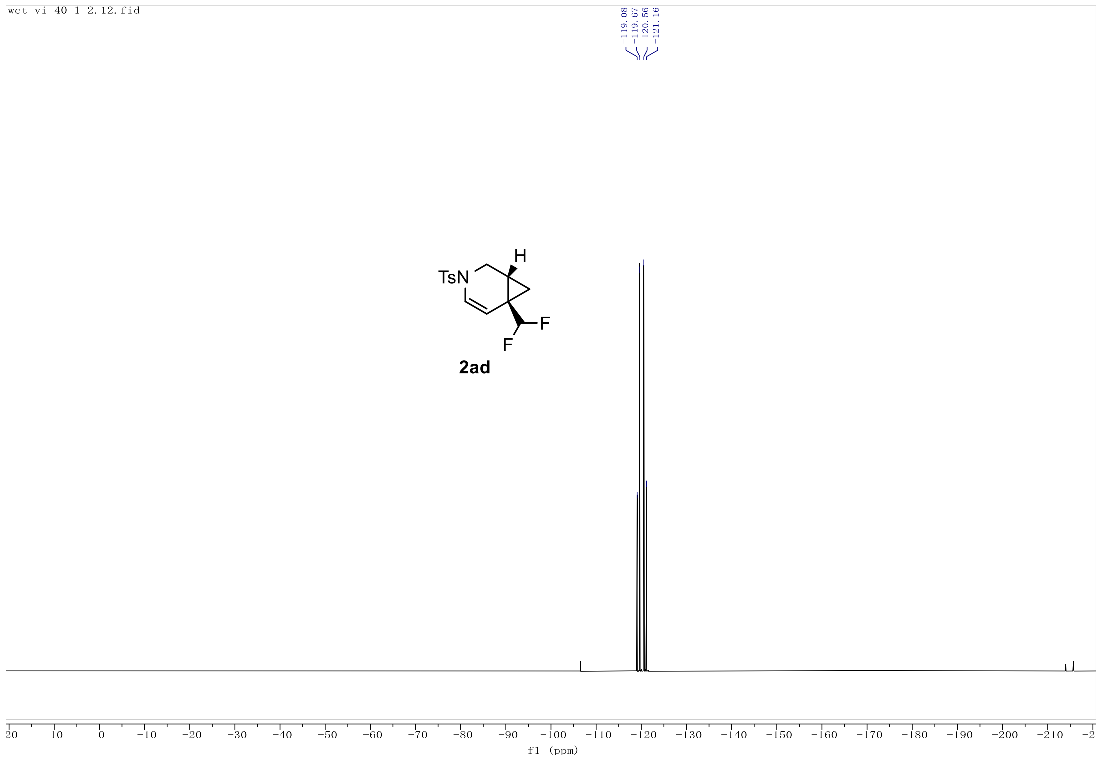
wct-vi-40-1-2.12.fid
-119.08
-119.67
-120.56
-121.16
TsN
H
F
F
2ad
f1 (ppm)

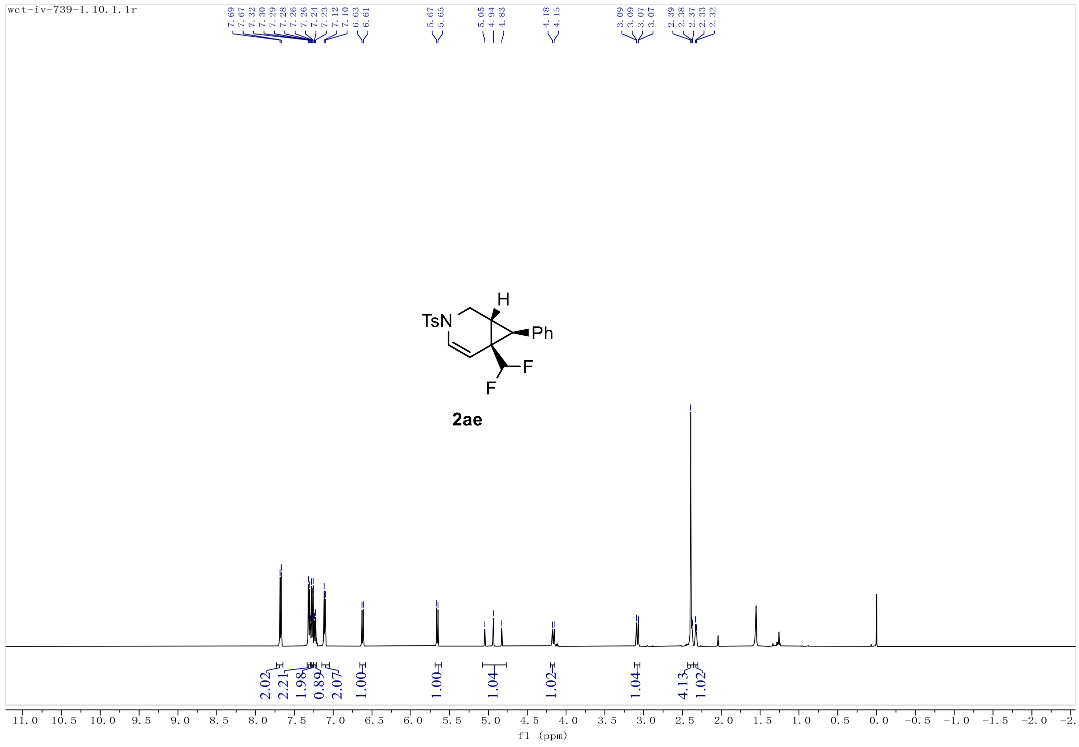
wct-iv-739-1.10.1.1r
7.69
7.67
7.32
7.30
7.29
7.28
7.26
7.24
7.23
7.12
7.10
6.63
6.61
5.67
5.65
5.05
4.94
4.83
4.18
4.15
3.09
3.09
3.07
3.07
2.39
2.38
2.37
2.33
2.32
TsN
H
Ph
F
F
2ae
2.02
2.21
1.98
0.89
2.07
1.00
1.00
1.04
1.02
1.04
4.13
1.02
f1 (ppm)

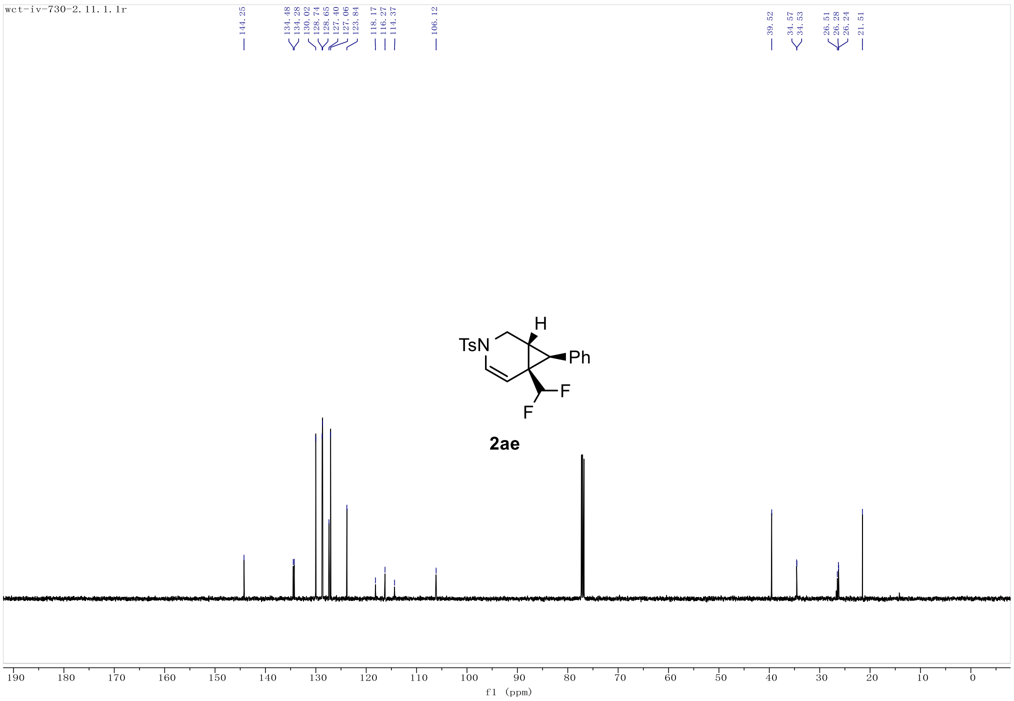
wct-iv-730-2.11.1.1r
144.25
134.48
134.28
130.02
128.74
128.65
127.40
127.06
123.84
118.17
116.27
114.37
106.12
39.52
34.57
34.53
26.51
26.28
26.24
21.51
TsN
H
Ph
F
F
2ae
f1 (ppm)

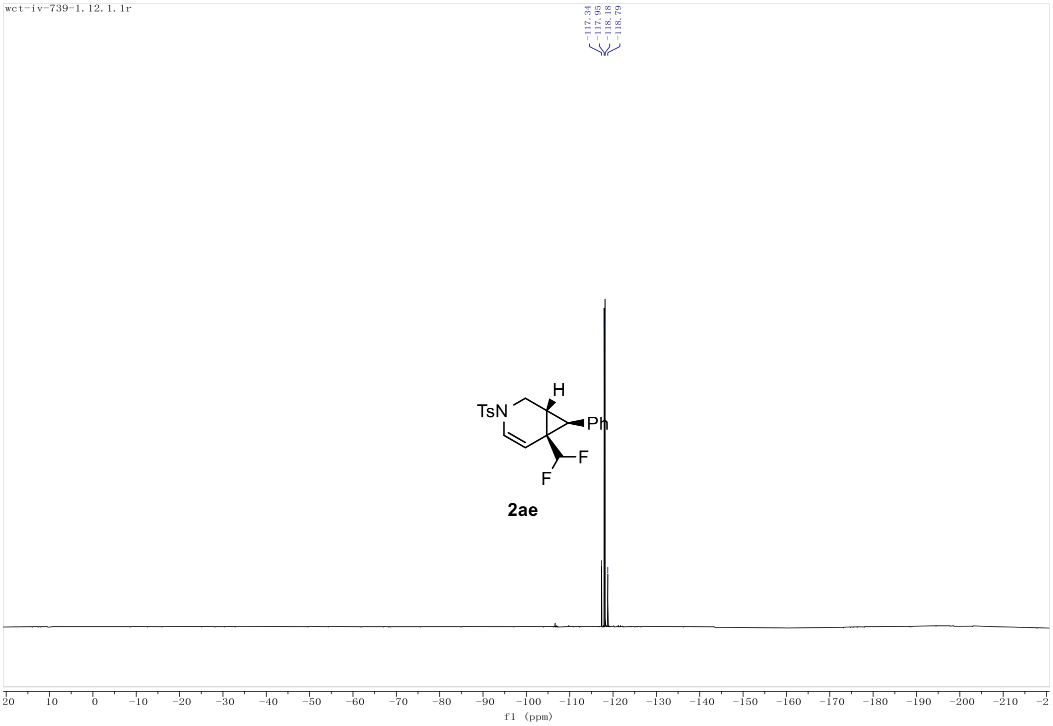
wct-iv-739-1.12.1.1r
-117.34
-117.95
-118.18
-118.79
TsN
H
Ph
F
F
2ae
f1 (ppm)

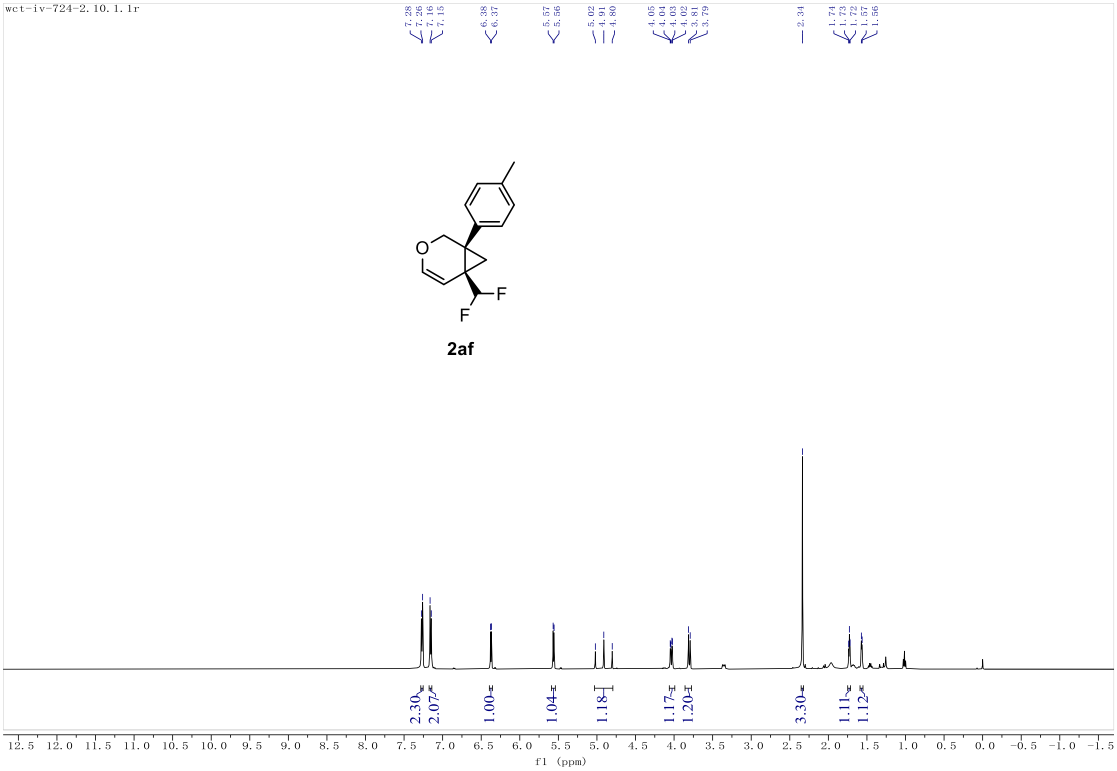
wct-iv-724-2.10.1.1r
7.28 7.26 7.16 7.15 6.38 6.37 5.57 5.56 5.02 4.91 4.80 4.05 4.04 4.03 4.02 3.81 3.79 2.34 1.74 1.73 1.72 1.57 1.56
O
F
F
2af
2.30 2.07 1.00 1.04 1.18 1.17 1.20 3.30 1.11 1.12
f1 (ppm)

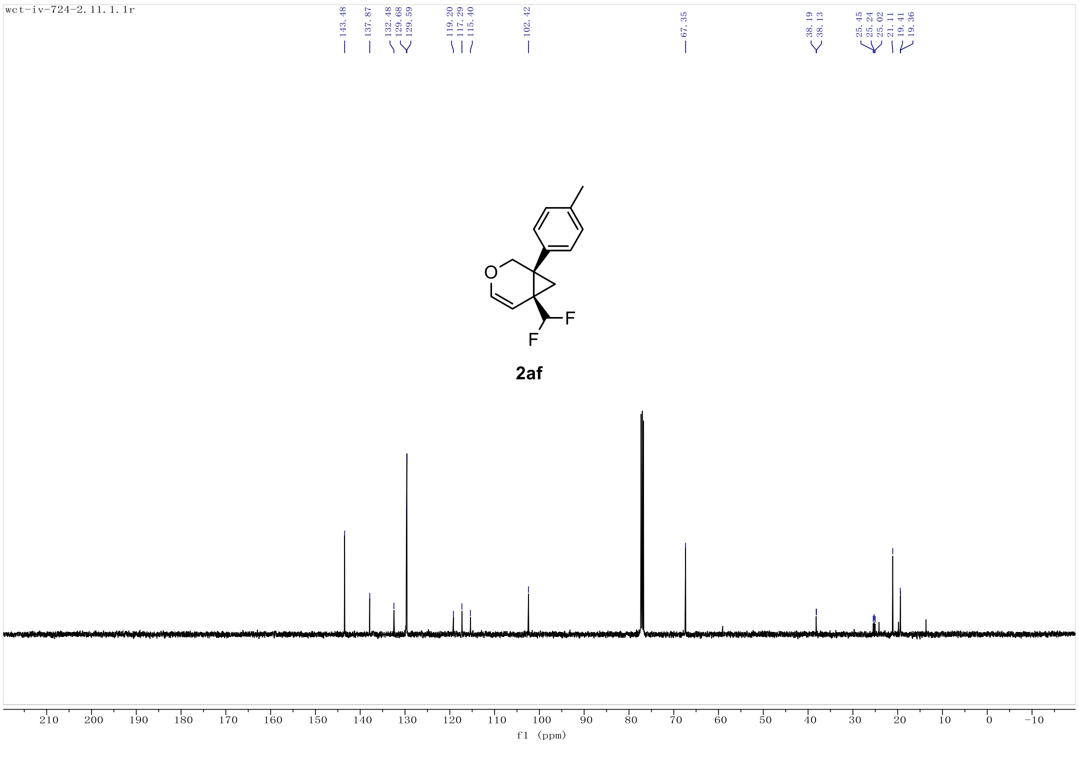
wct-iv-724-2.11.1.1r
143.48 137.87 132.48 129.68 129.59 119.20 117.29 115.40 102.42 67.35 38.19 38.13 25.45 25.24 25.02 21.11 19.41 19.36
O
F
F
2af
f1 (ppm)

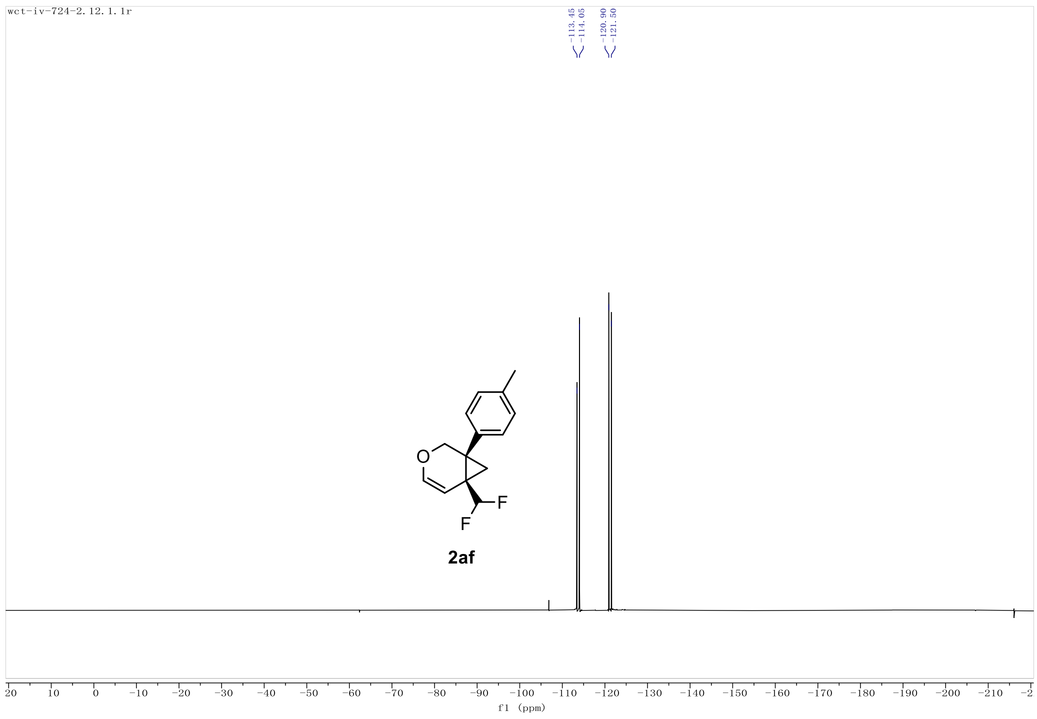
wct-iv-724-2.12.1.1r
-113.45
-114.05
-120.90
-121.50
O
F
F
2af
f1 (ppm)

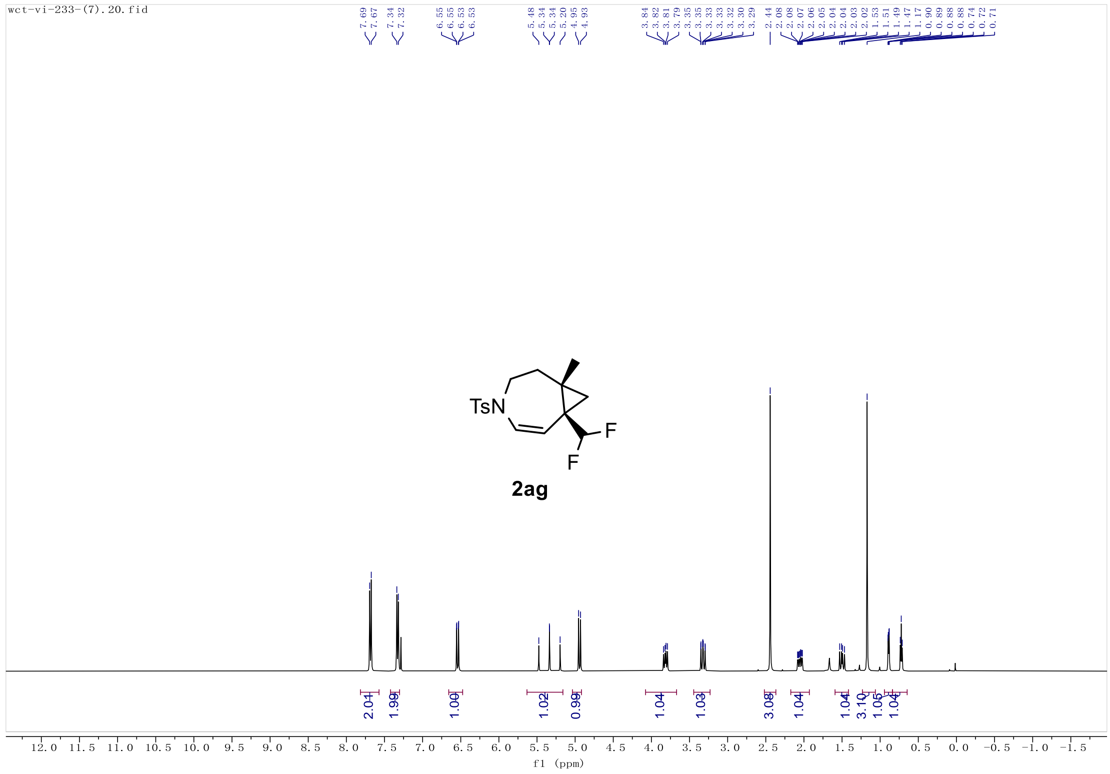
wct-vi-233-(7).20.fid
TsN
F
F
2ag
2.04
1.99
1.00
1.02
0.99
1.04
1.03
3.08
1.04
1.04
3.10
1.05
1.04
f1 (ppm)

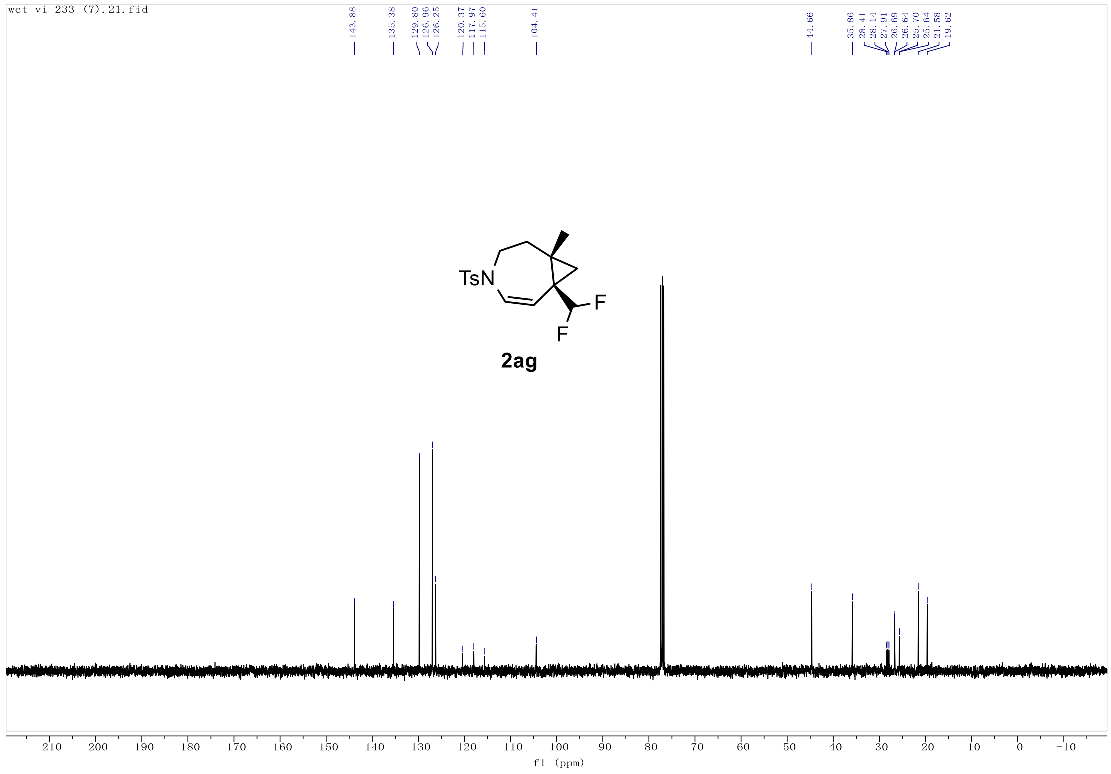
wct-vi-233-(7).21.fid
143.88
135.38
129.80
126.96
126.25
120.37
117.97
115.60
104.41
44.66
35.86
28.41
28.14
27.91
26.69
26.64
25.70
25.64
21.58
19.62
TsN
F
F
2ag
f1 (ppm)

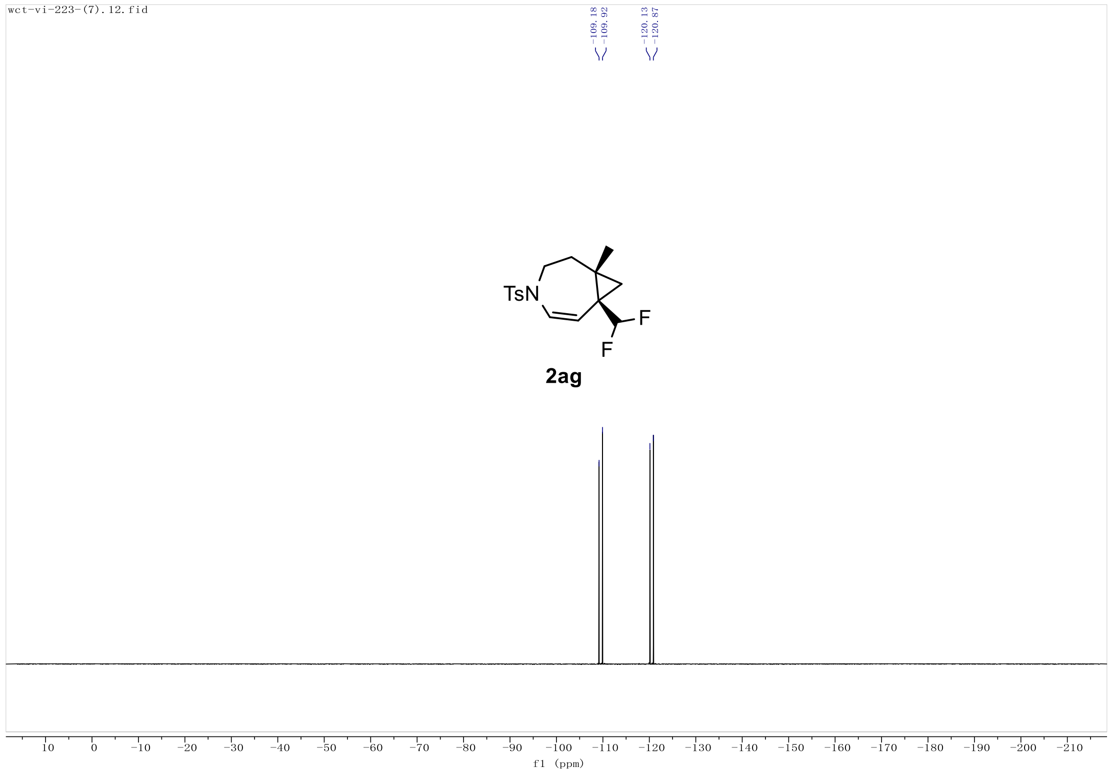
wct-vi-223-(7).12.fid
-109.18
-109.92
-120.13
-120.87
TsN
F
F
2ag
f1 (ppm)

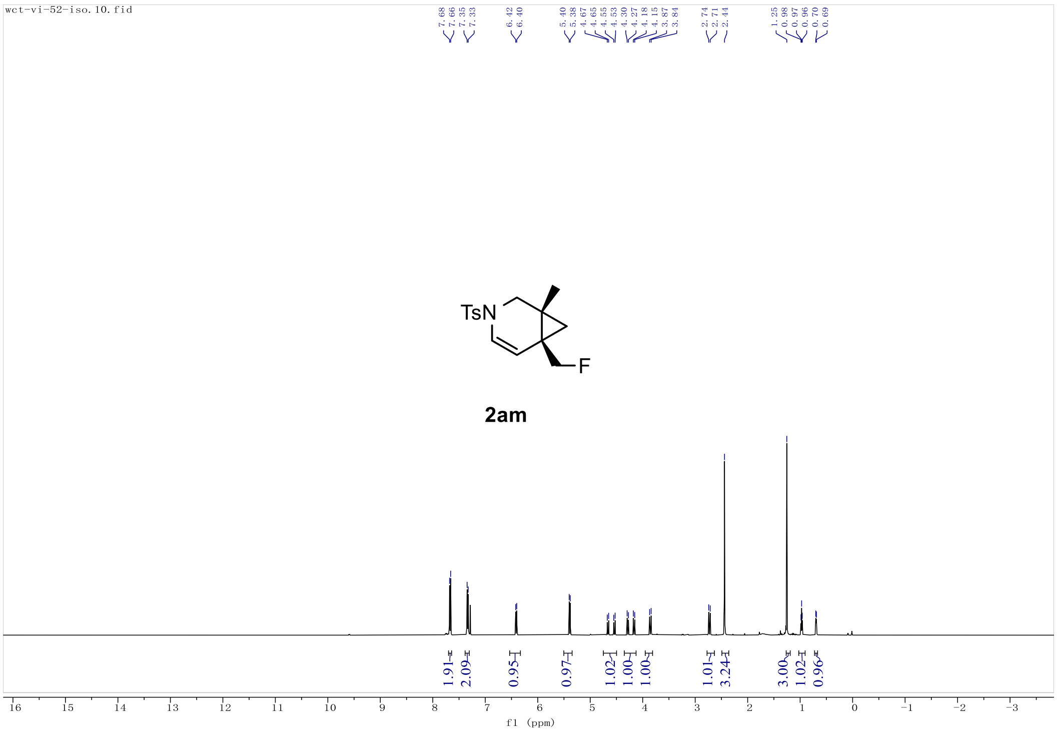
wct-vi-52-iso.10.fid
7.68 7.66 7.35 7.33 6.42 6.40 5.40 5.38 4.67 4.65 4.55 4.53 4.30 4.27 4.18 4.15 3.87 3.84 2.74 2.71 2.44 1.25 0.98 0.97 0.96 0.70 0.69
TsN
F
2am
1.91 2.09 0.95 0.97 1.02 1.00 1.00 1.01 3.24 3.00 1.02 0.96
16 15 14 13 12 11 10 9 8 7 6 5 4 3 2 1 0 −1 −2 −3
f1 (ppm)

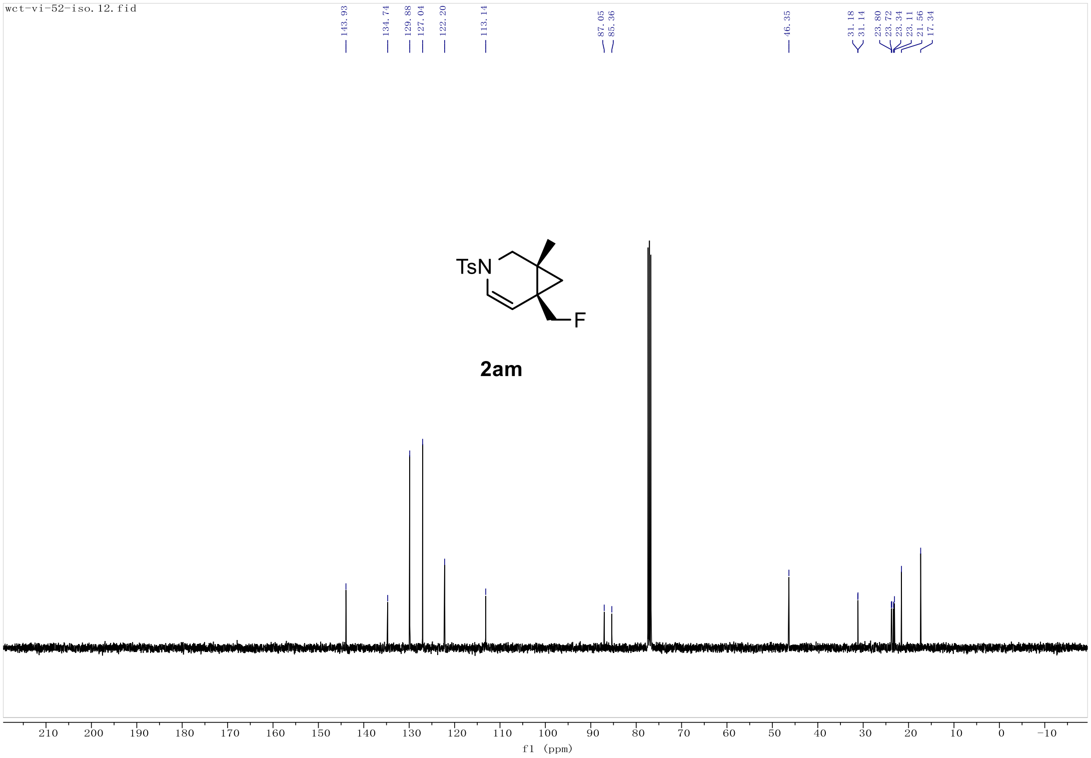
wct-vi-52-iso.12.fid
143.93 134.74 129.88 127.04 122.20 113.14 87.05 85.36 46.35 31.18 31.14 23.80 23.72 23.34 23.11 21.56 17.34
TsN
F
2am
210 200 190 180 170 160 150 140 130 120 110 100 90 80 70 60 50 40 30 20 10 0 −10
f1 (ppm)

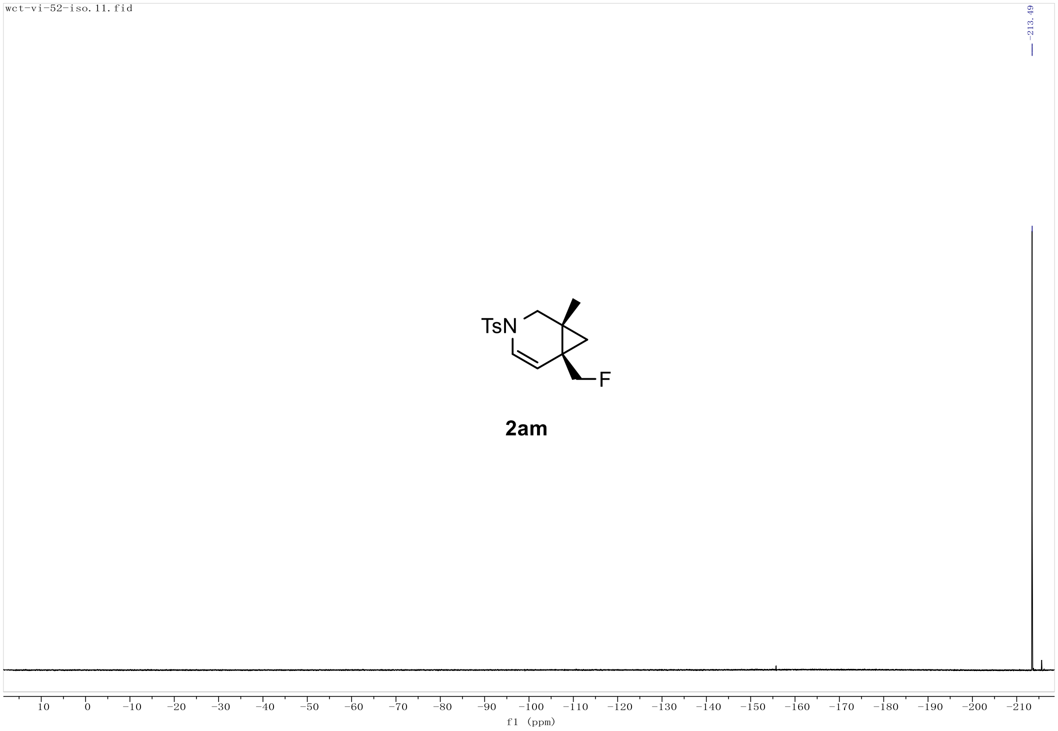
wct-vi-52-iso.11.fid
-213.49
TsN
F
2am
f1 (ppm)

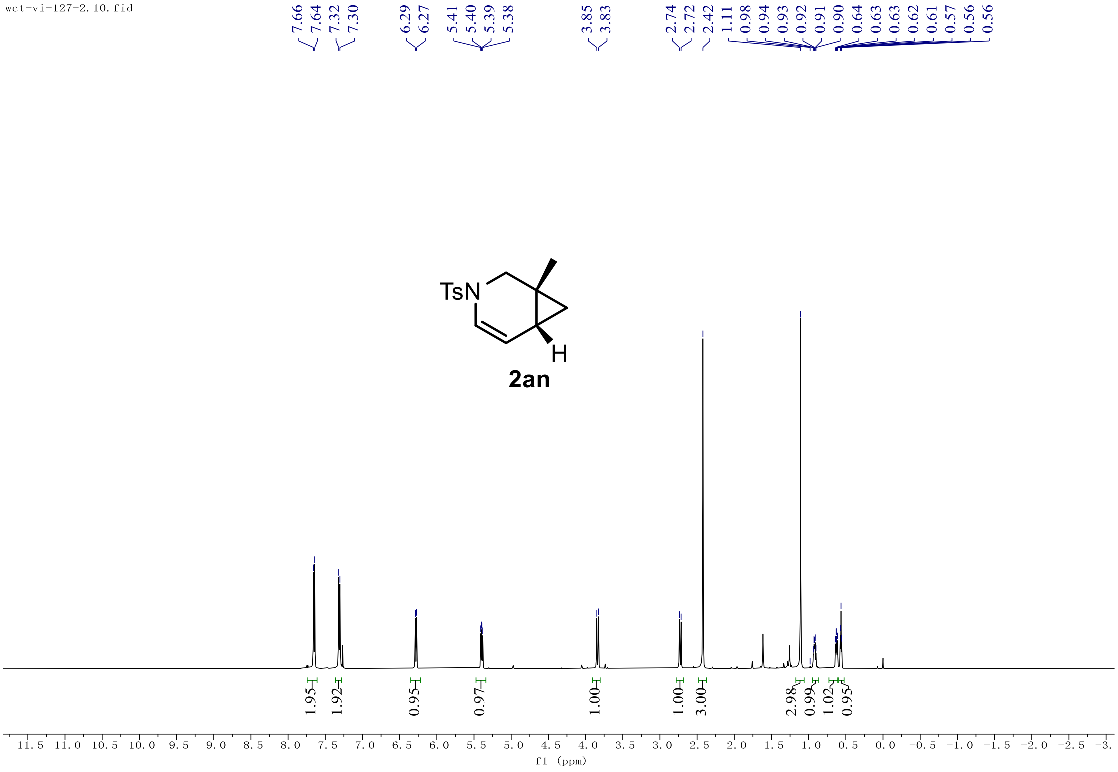
wct-vi-127-2.10.fid
7.66 7.64 7.32 7.30 6.29 6.27 5.41 5.40 5.39 5.38 3.85 3.83 2.74 2.72 2.42 1.11 0.98 0.94 0.93 0.92 0.91 0.90 0.64 0.63 0.63 0.62 0.61 0.57 0.56 0.56
TsN
H
2an
1.95 1.92 0.95 0.97 1.00 1.00 3.00 2.98 0.99 1.02 0.95
f1 (ppm)

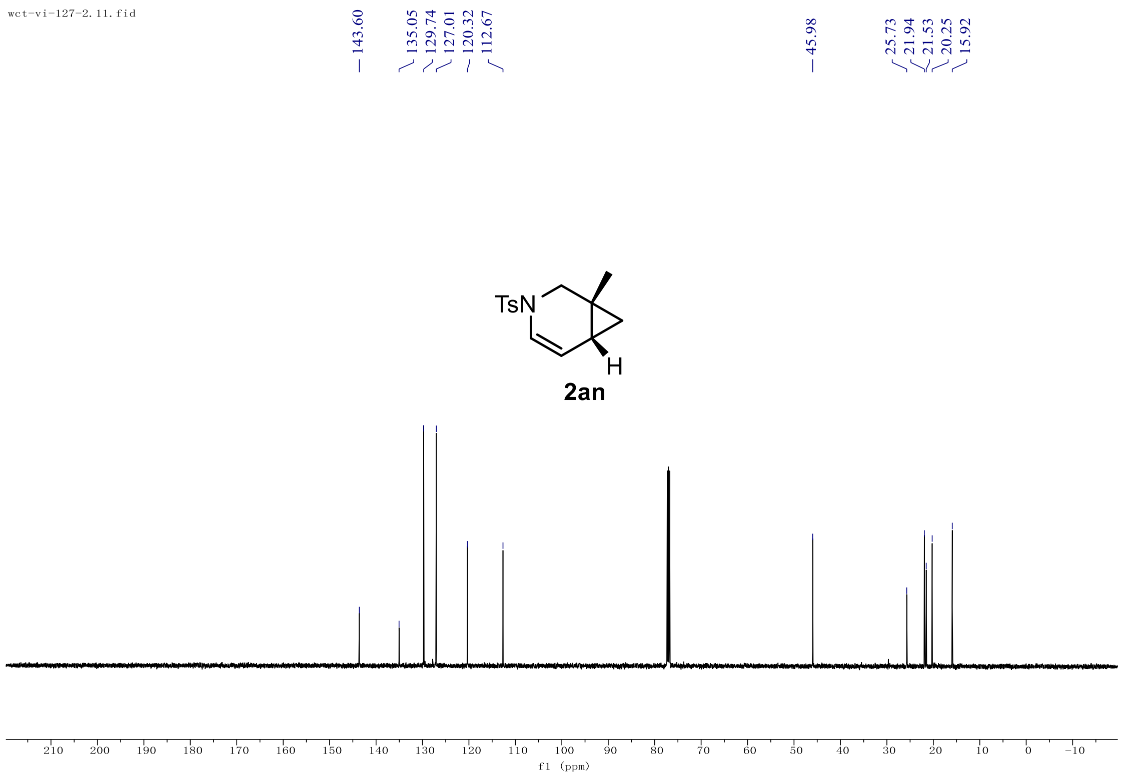
wct-vi-127-2.11.fid
143.60
135.05
129.74
127.01
120.32
112.67
45.98
25.73
21.94
21.53
20.25
15.92
TsN
H
2an
f1 (ppm)

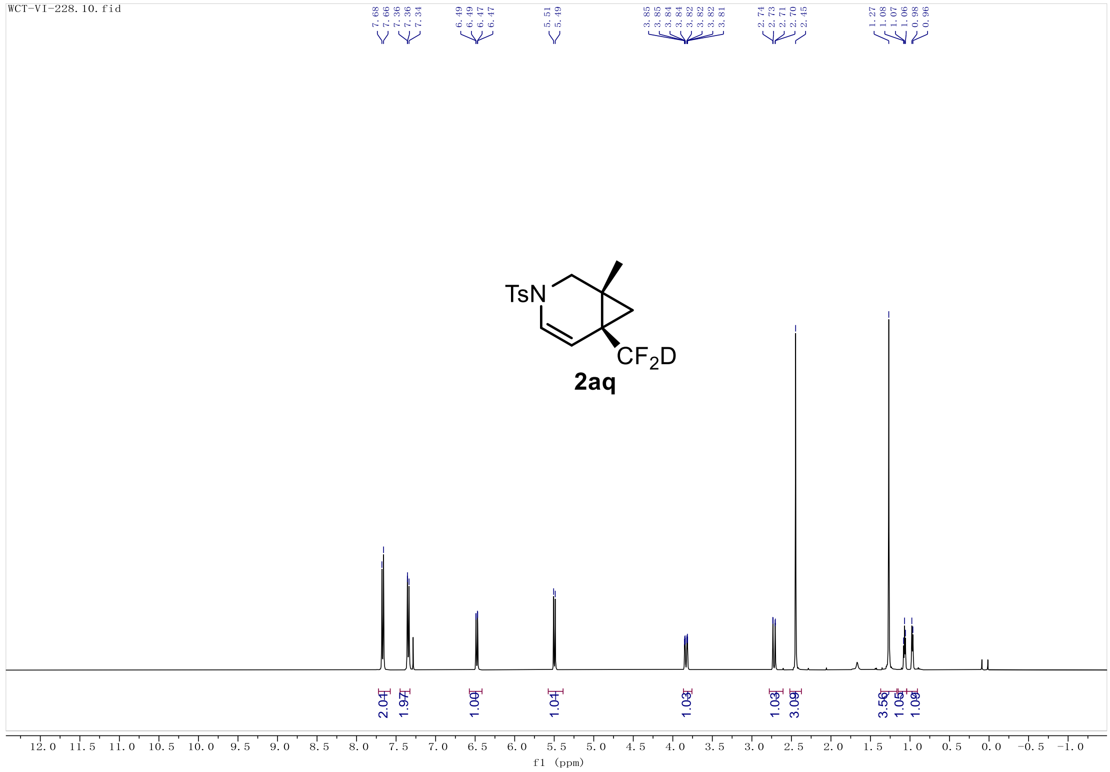
WCT-VI-228.10.fid
TsN
CF2D
2aq
2.04
1.97
1.00
1.04
1.03
1.03
3.09
3.56
1.05
1.08
f1 (ppm)

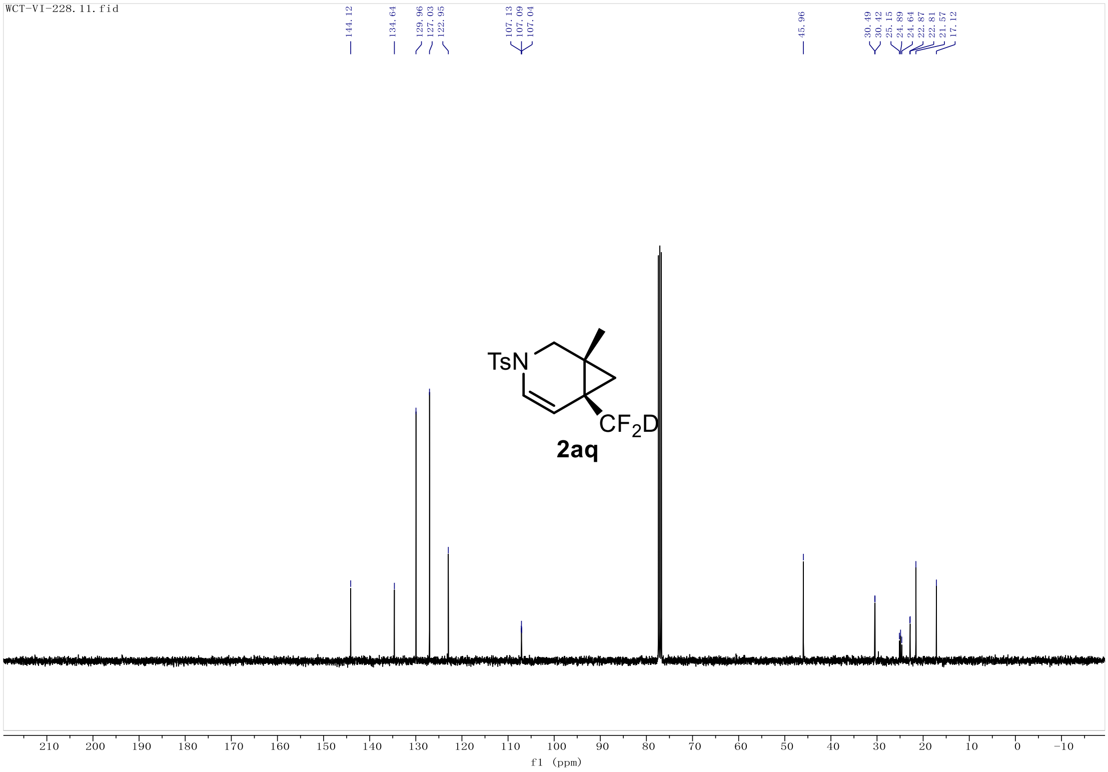
WCT-VI-228.11.fid
144.12
134.64
129.96
127.03
122.95
107.13
107.09
107.04
45.96
30.49
30.42
25.15
24.89
24.64
22.87
22.81
21.57
17.12
TsN
CF2D
2aq
f1 (ppm)

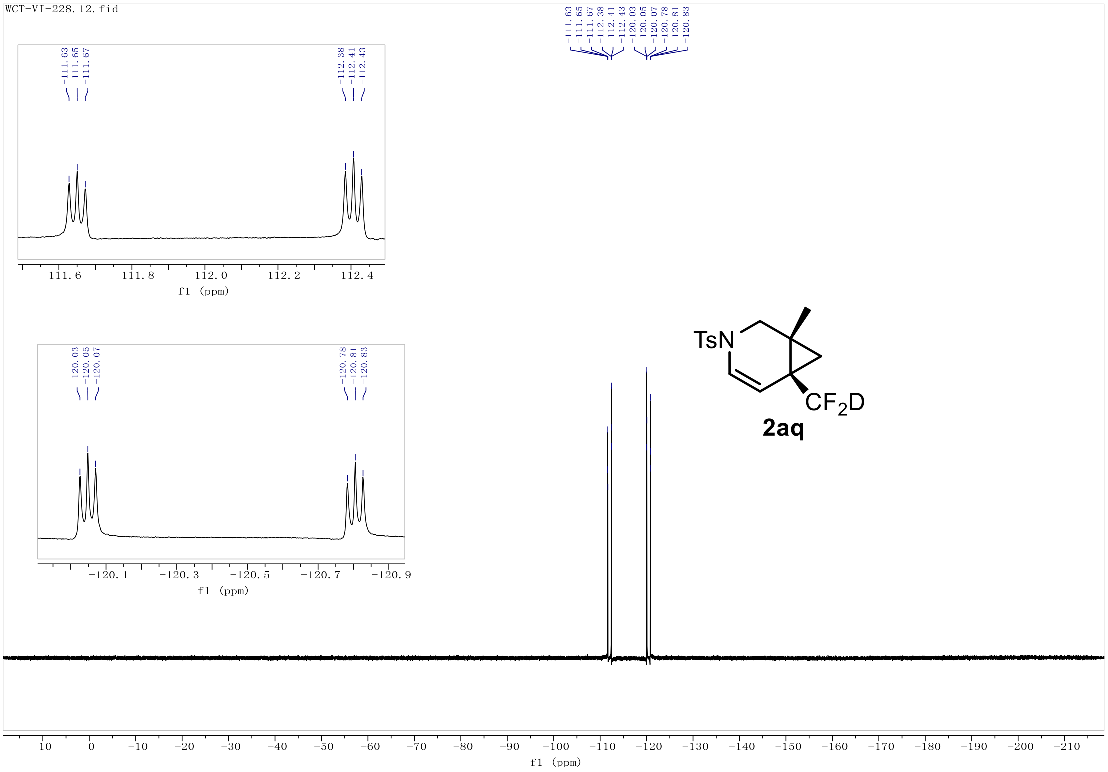
WCT-VI-228.12.fid
-111.63
-111.65
-111.67
-112.38
-112.41
-112.43
-120.03
-120.05
-120.07
-120.78
-120.81
-120.83
TsN
CF2D
2aq
f1 (ppm)

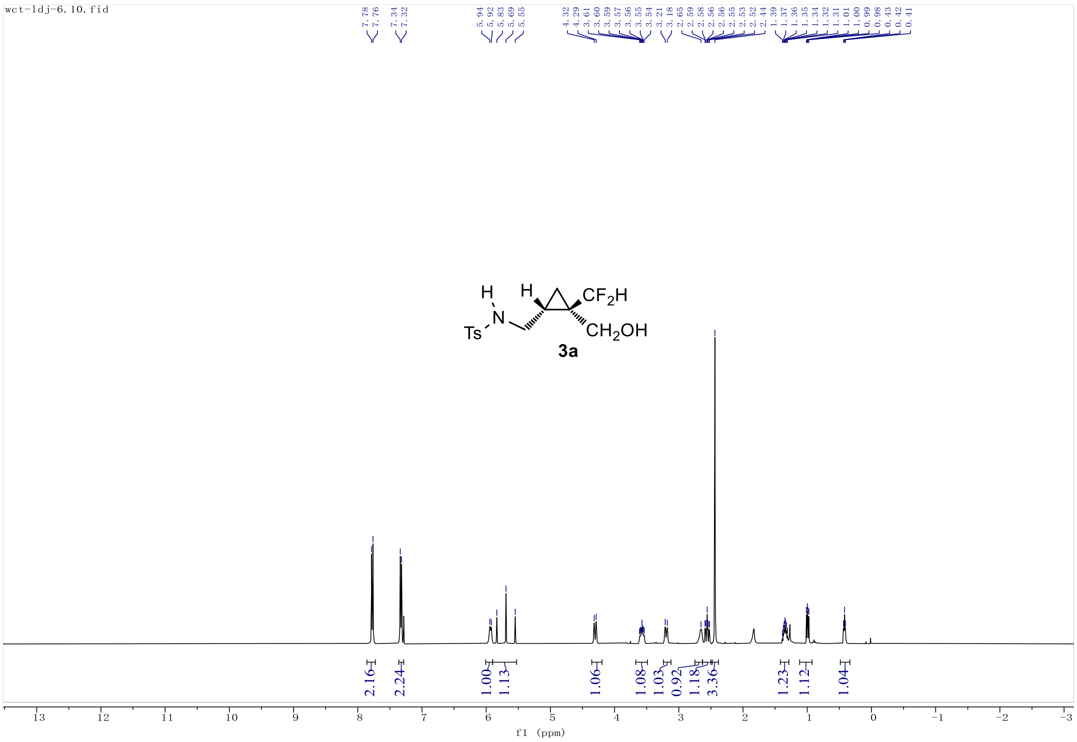
wct-ldj-6.10.fid
7.78
7.76
7.34
7.32
5.94
5.92
5.83
5.69
5.55
4.32
4.29
3.61
3.60
3.59
3.57
3.56
3.55
3.54
3.21
3.18
2.65
2.59
2.58
2.56
2.56
2.55
2.53
2.52
2.44
1.39
1.37
1.36
1.35
1.34
1.32
1.31
1.01
1.00
0.99
0.98
0.43
0.42
0.41
3a
2.16
2.24
1.00
1.13
1.06
1.08
1.03
0.92
1.18
3.36
1.23
1.12
1.04
f1 (ppm)

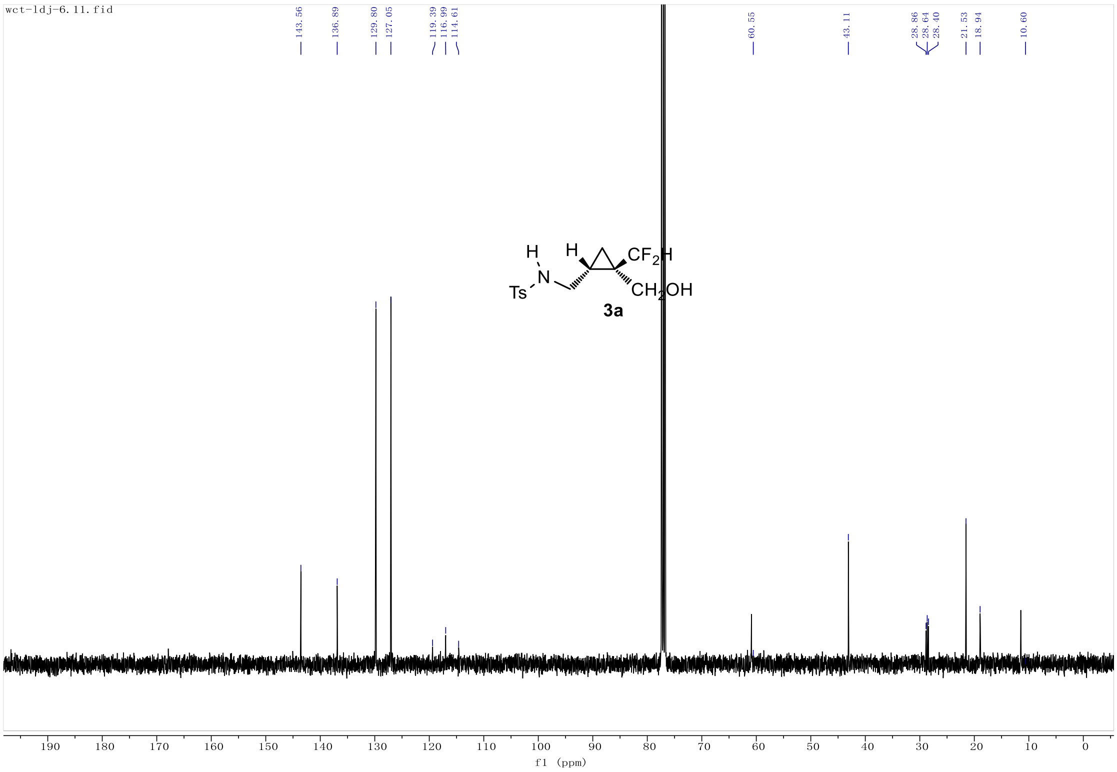
wct-ldj-6.11.fid
143.56
136.89
129.80
127.05
119.39
116.99
114.61
60.55
43.11
28.86
28.64
28.40
21.53
18.94
10.60
3a
f1 (ppm)

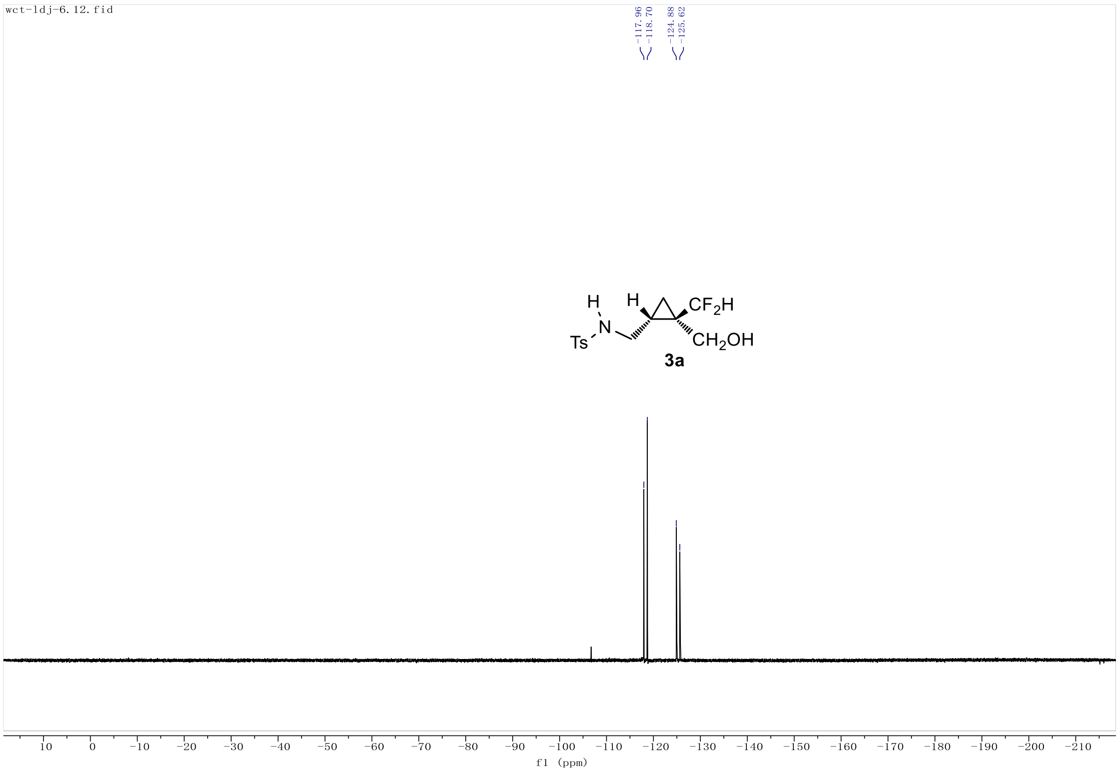
wct-ldj-6.12.fid
-117.96
-118.70
-124.88
-125.62
3a
f1 (ppm)

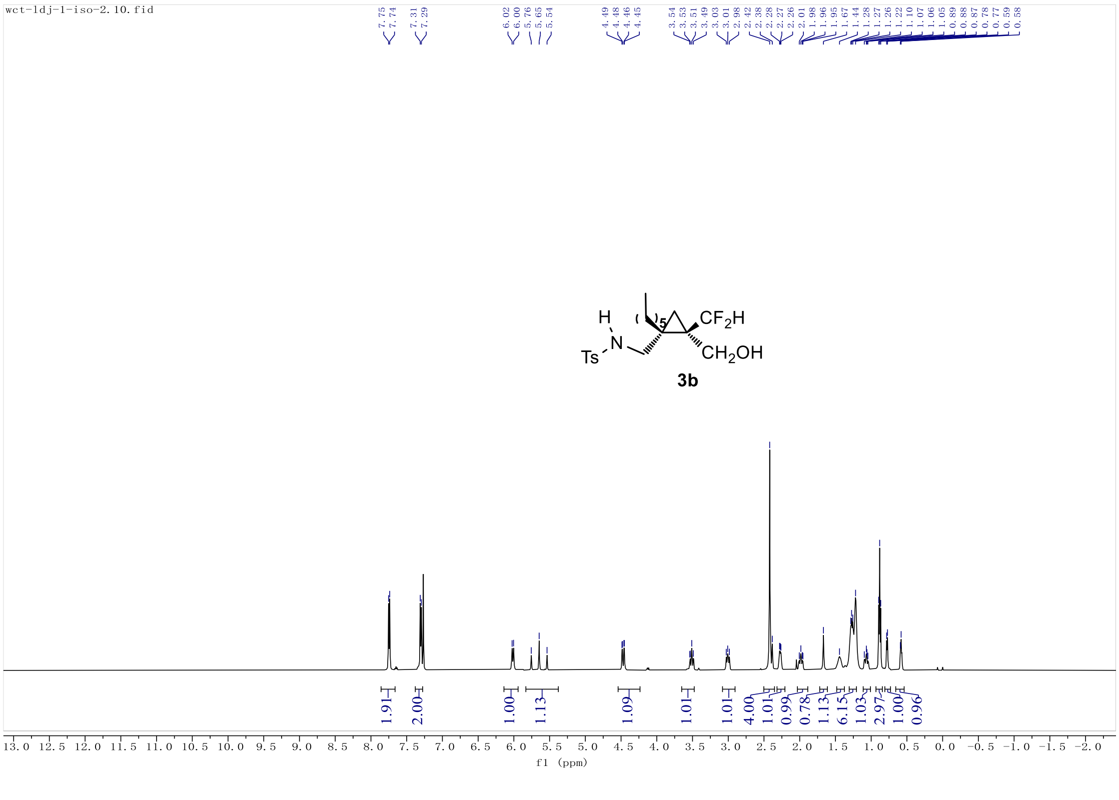
wct-ldj-1-iso-2.10.fid
3b
f1 (ppm)

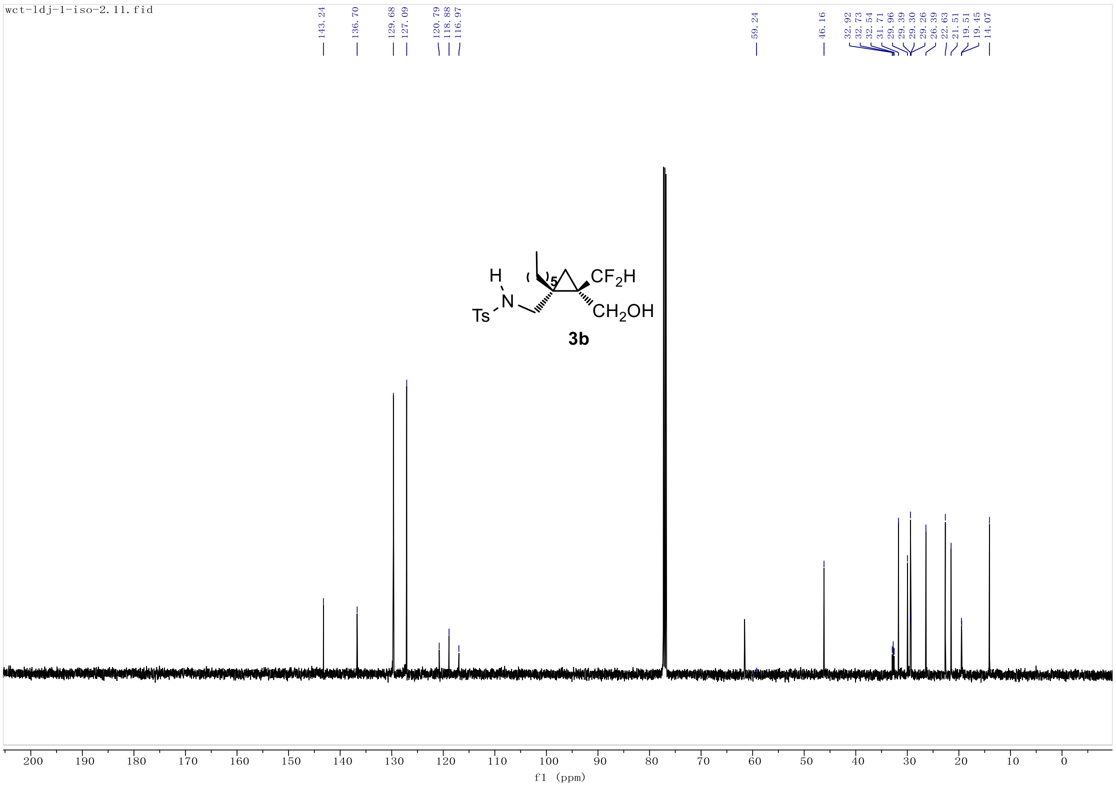
wct-ldj-1-iso-2.11.fid
143.24
136.70
129.68
127.09
120.79
118.88
116.97
59.24
46.16
32.92
32.73
32.54
31.71
29.96
29.39
29.30
29.26
26.39
22.63
21.51
19.51
19.45
14.07
H
N
Ts
( )5
CF2H
CH2OH
3b
f1 (ppm)

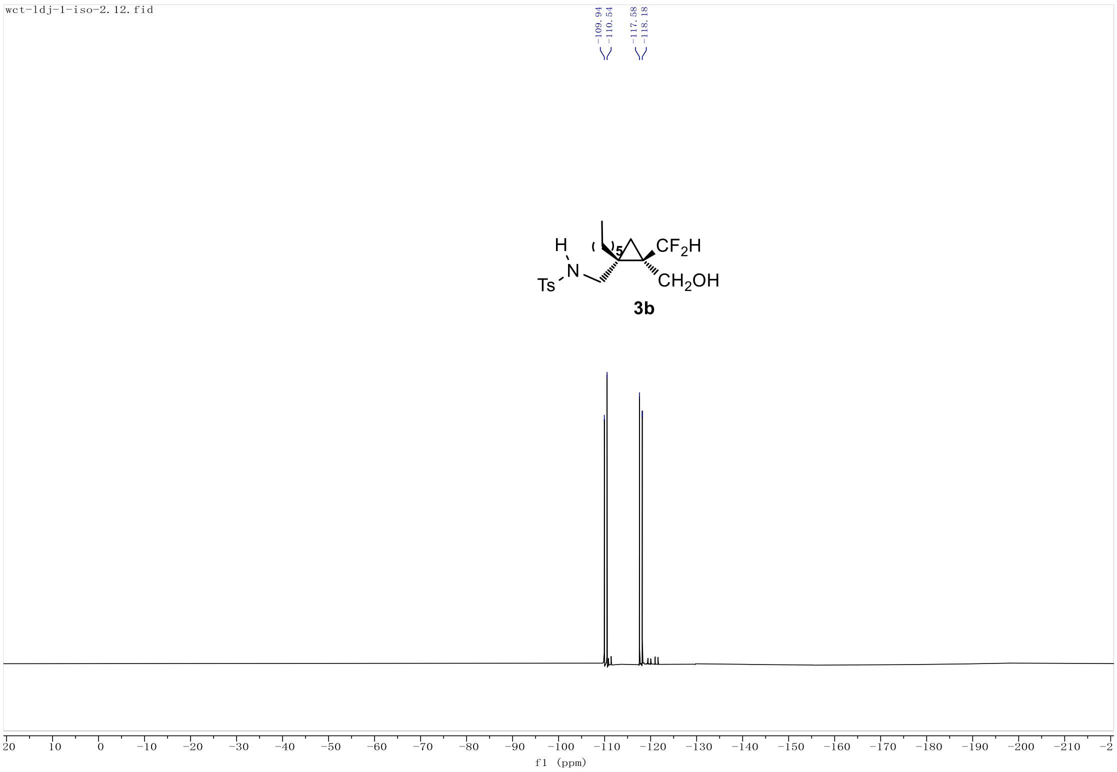
wct-ldj-1-iso-2.12.fid
-109.94
-110.54
-117.58
-118.18
H
N
Ts
( )5
CF2H
CH2OH
3b
f1 (ppm)

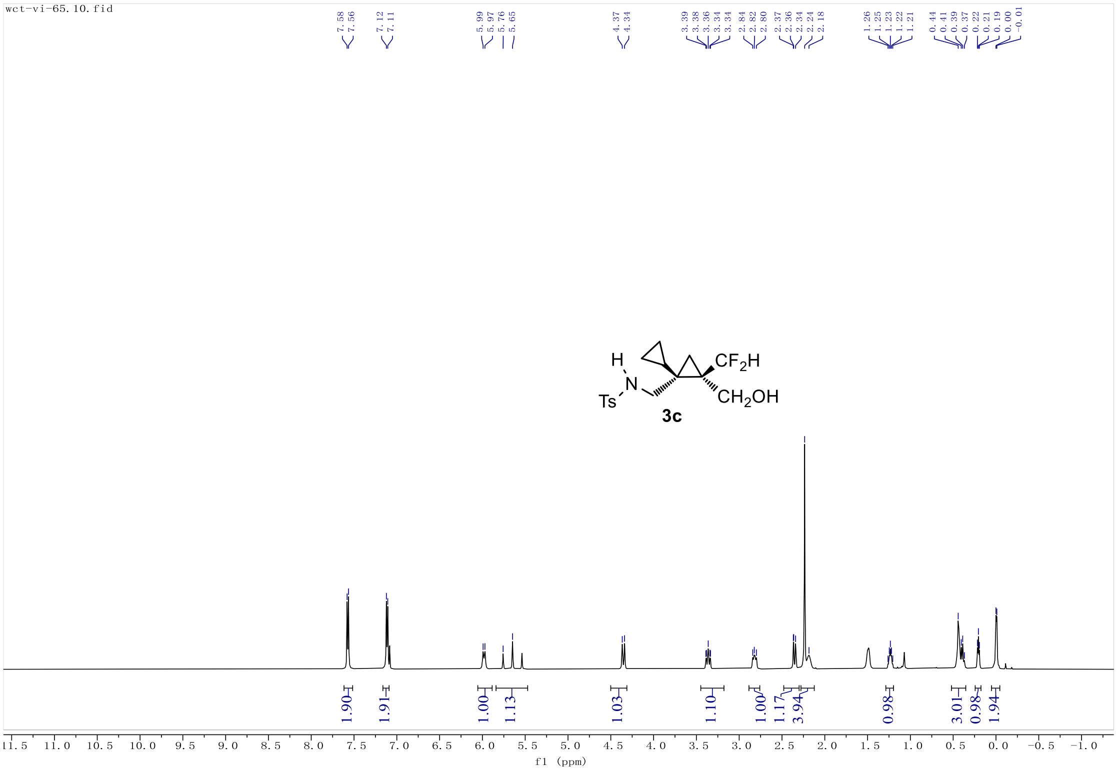
wct-vi-65.10.fid
7.58
7.56
7.12
7.11
5.99
5.97
5.76
5.65
4.37
4.34
3.39
3.38
3.36
3.34
3.34
2.84
2.82
2.80
2.37
2.36
2.34
2.24
2.18
1.26
1.25
1.23
1.22
1.21
0.44
0.41
0.39
0.37
0.22
0.21
0.19
0.00
-0.01
H
N
Ts
CF2H
CH2OH
3c
1.90
1.91
1.00
1.13
1.03
1.10
1.00
1.17
3.94
0.98
3.01
0.98
1.94
f1 (ppm)

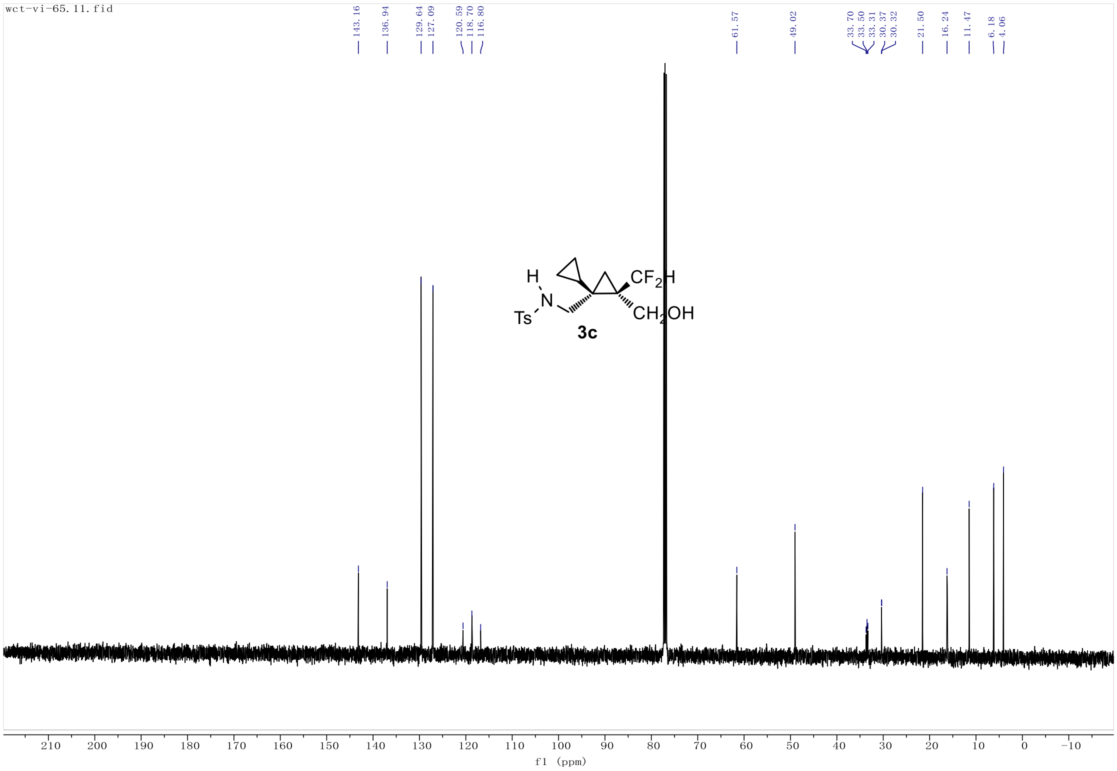
wct-vi-65.11.fid
143.16
136.94
129.64
127.09
120.59
118.70
116.80
61.57
49.02
33.70
33.50
33.31
30.37
30.32
21.50
16.24
11.47
6.18
4.06
H
N
Ts
CF2H
CH2OH
3c
f1 (ppm)

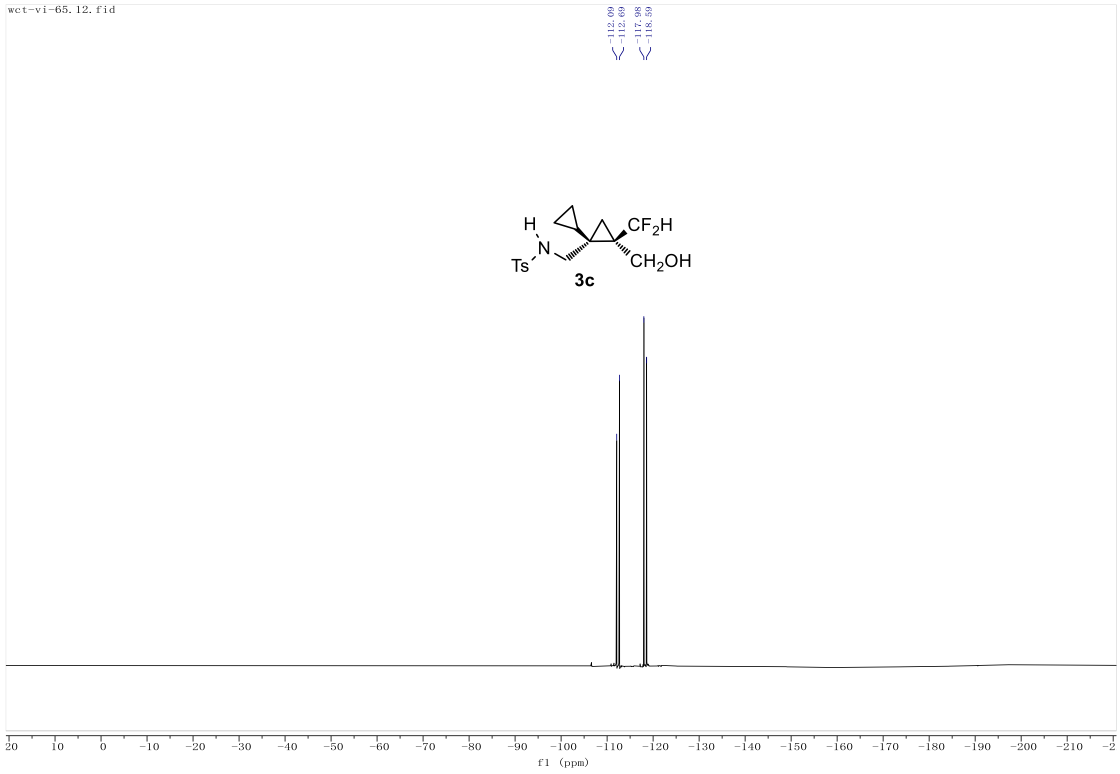
wct-vi-65.12.fid
3c
f1 (ppm)

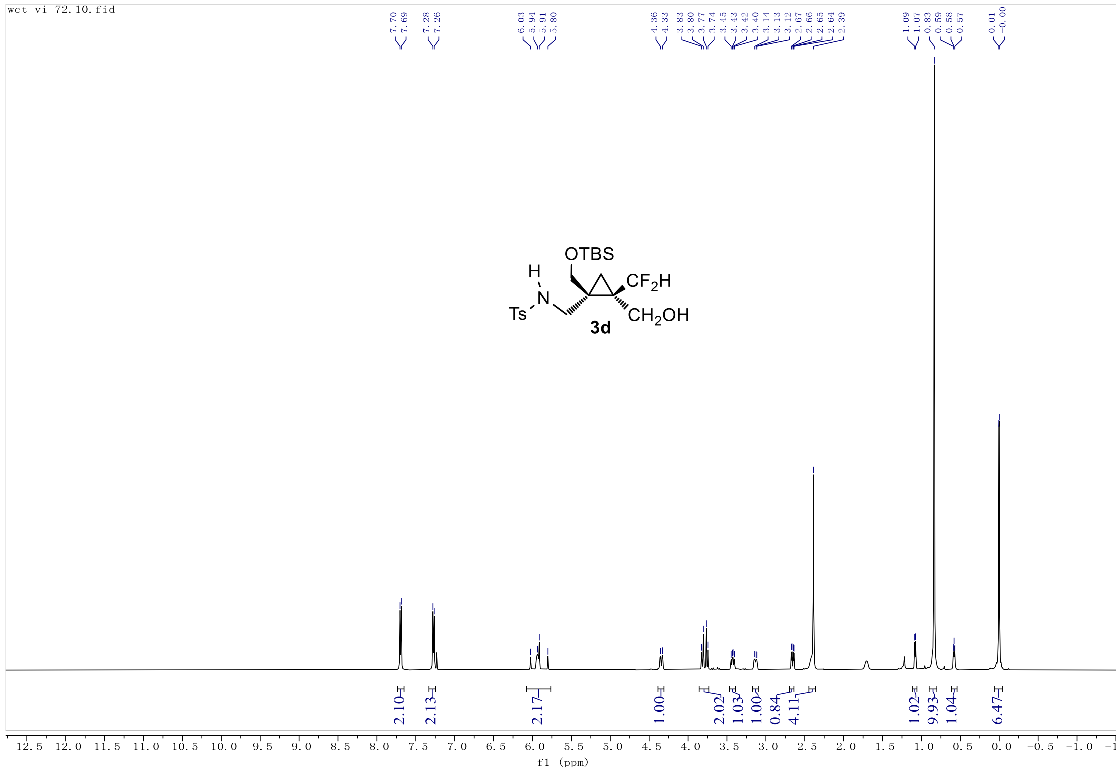
wct-vi-72.10.fid
OTBS
3d
f1 (ppm)

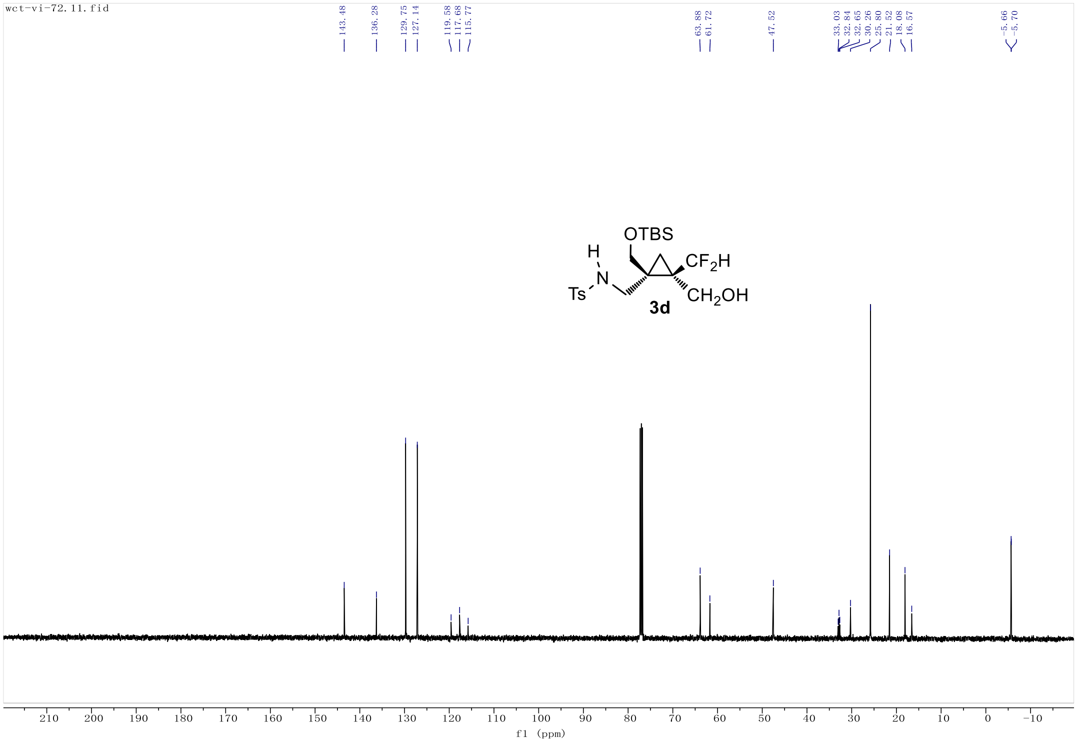
wct-vi-72.11.fid
143.48
136.28
129.75
127.14
119.58
117.68
115.77
63.88
61.72
47.52
33.03
32.84
32.65
30.26
25.80
21.52
18.08
16.57
-5.66
-5.70
OTBS
H
N
Ts
CF2H
CH2OH
3d
f1 (ppm)

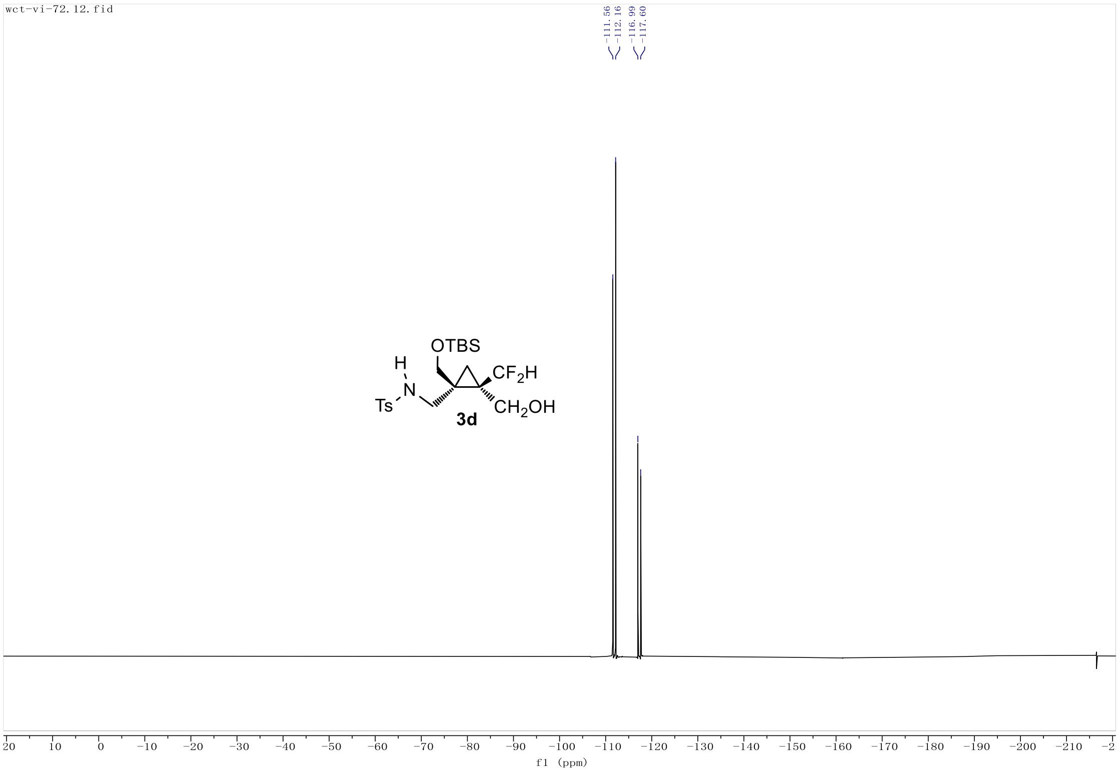
wct-vi-72.12.fid
-111.56
-112.16
-116.99
-117.60
OTBS
H
N
Ts
CF2H
CH2OH
3d
f1 (ppm)

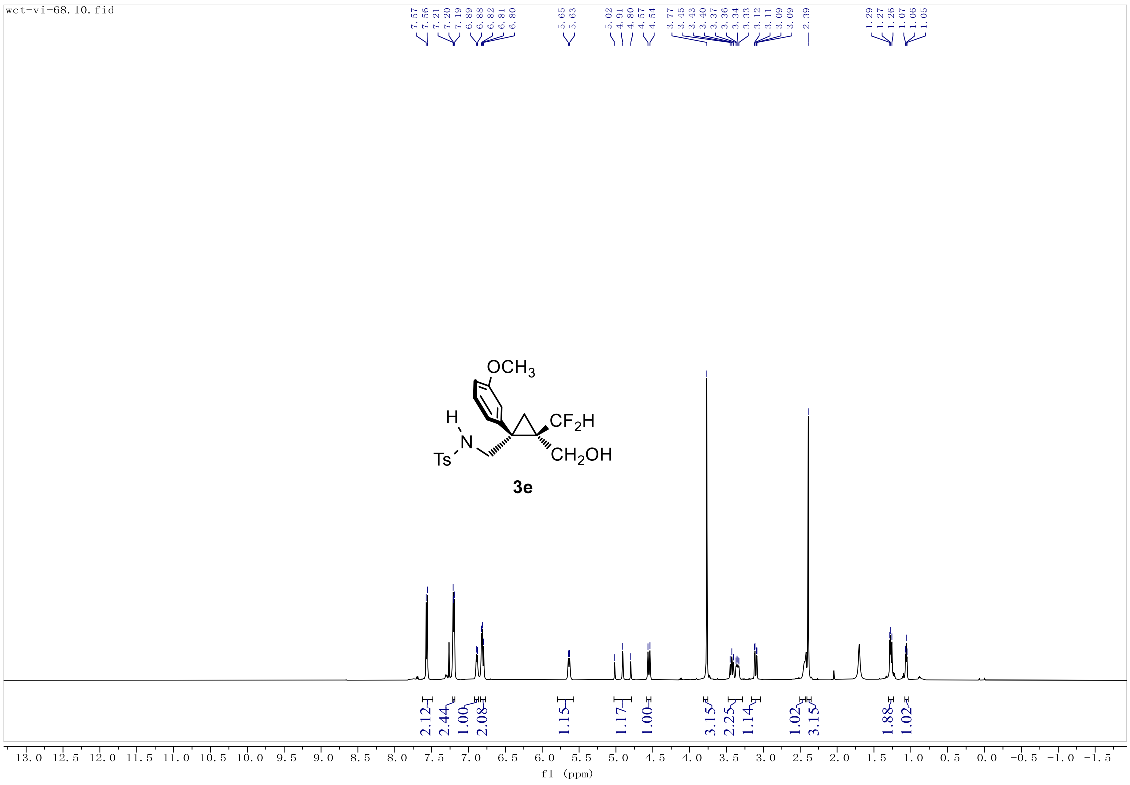
wct-vi-68.10.fid
7.57 7.56 7.21 7.20 7.19 6.89 6.88 6.82 6.81 6.80
5.65 5.63
5.02 4.91 4.80 4.57 4.54
3.77 3.45 3.43 3.40 3.37 3.36 3.34 3.33 3.12 3.11 3.09 3.09
2.39
1.29 1.27 1.26 1.07 1.06 1.05
OCH3
H
N
Ts
CF2H
CH2OH
3e
2.12 2.44 1.00 2.08 1.15 1.17 1.00 3.15 2.25 1.14 1.02 3.15 1.88 1.02
13.0 12.5 12.0 11.5 11.0 10.5 10.0 9.5 9.0 8.5 8.0 7.5 7.0 6.5 6.0 5.5 5.0 4.5 4.0 3.5 3.0 2.5 2.0 1.5 1.0 0.5 0.0 -0.5 -1.0 -1.5
f1 (ppm)

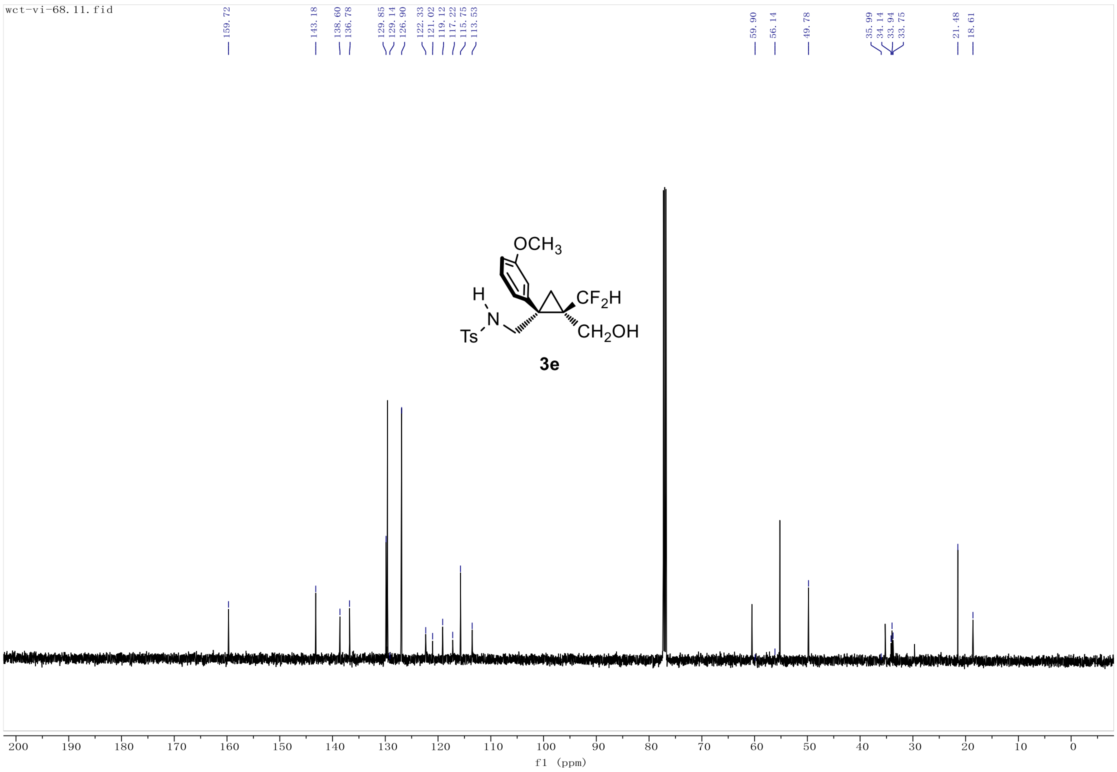
wct-vi-68.11.fid
159.72
143.18 138.60 136.78
129.85 129.14 126.90 122.33 121.02 119.12 117.22 115.75 113.53
59.90 56.14 49.78
35.99 34.14 33.94 33.75
21.48 18.61
OCH3
H
N
Ts
CF2H
CH2OH
3e
200 190 180 170 160 150 140 130 120 110 100 90 80 70 60 50 40 30 20 10 0
f1 (ppm)

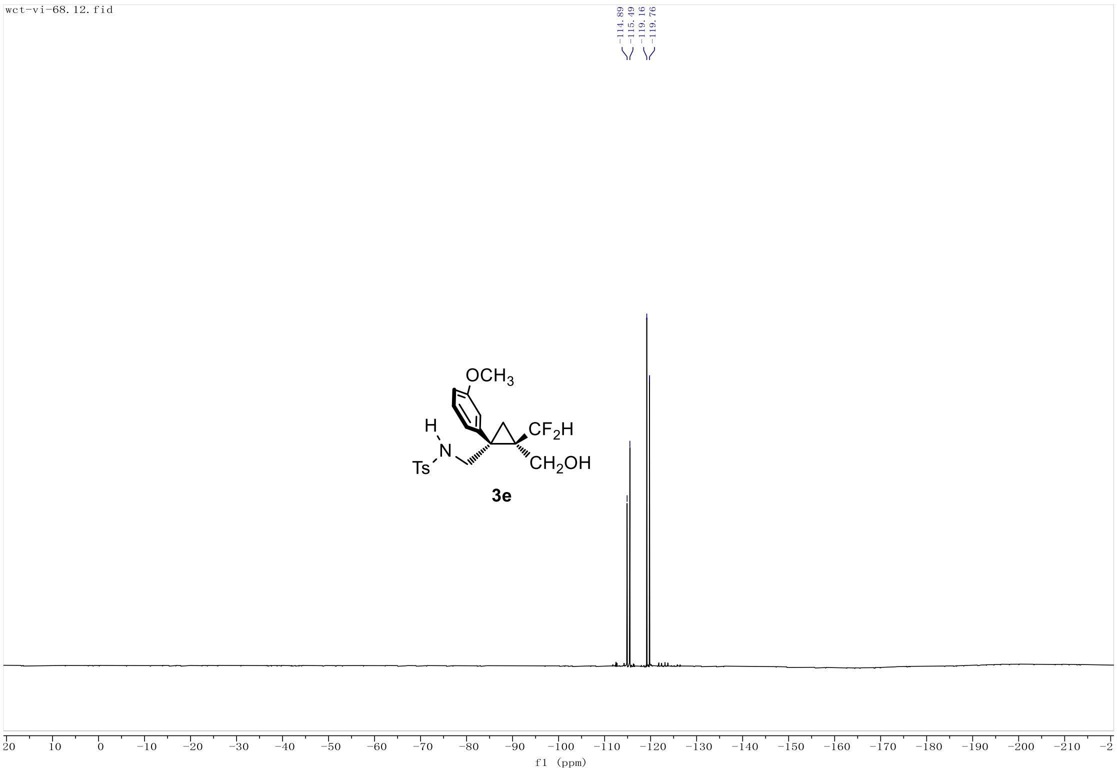
wct-vi-68.12.fid
-114.89
-115.49
-119.16
-119.76
OCH3
H
N
Ts
CF2H
CH2OH
3e
f1 (ppm)

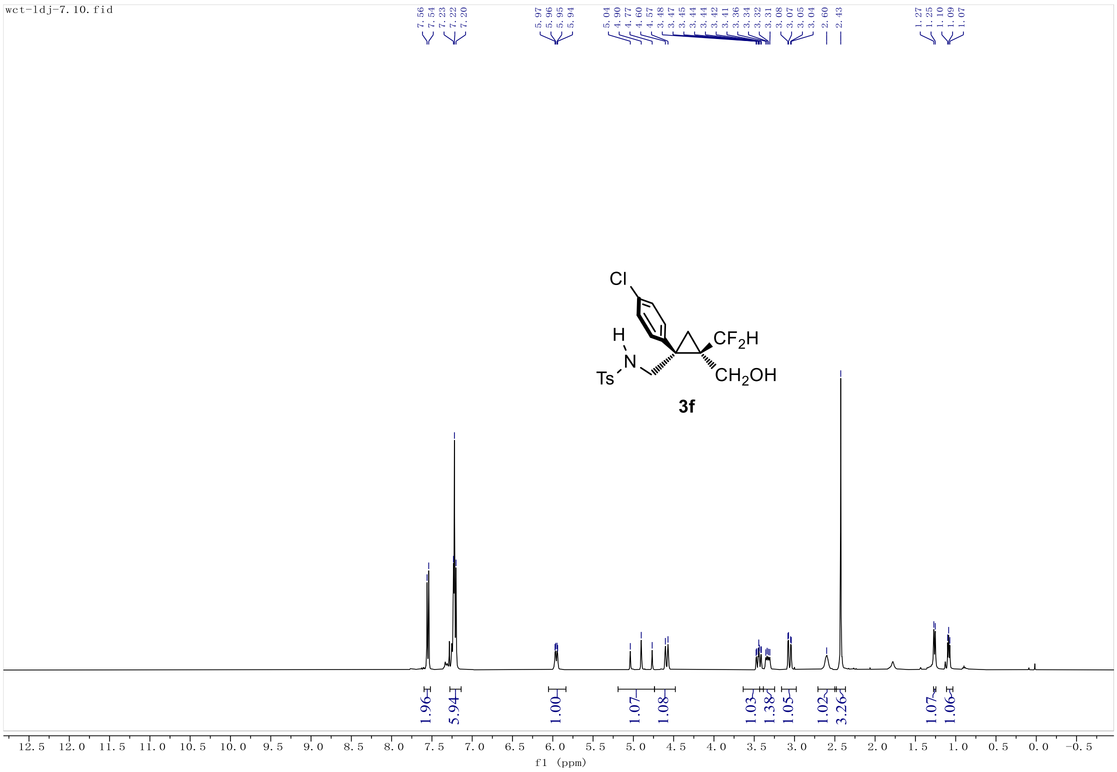
wct-ldj-7.10.fid
Cl
H
N
Ts
CF2H
CH2OH
3f
f1 (ppm)

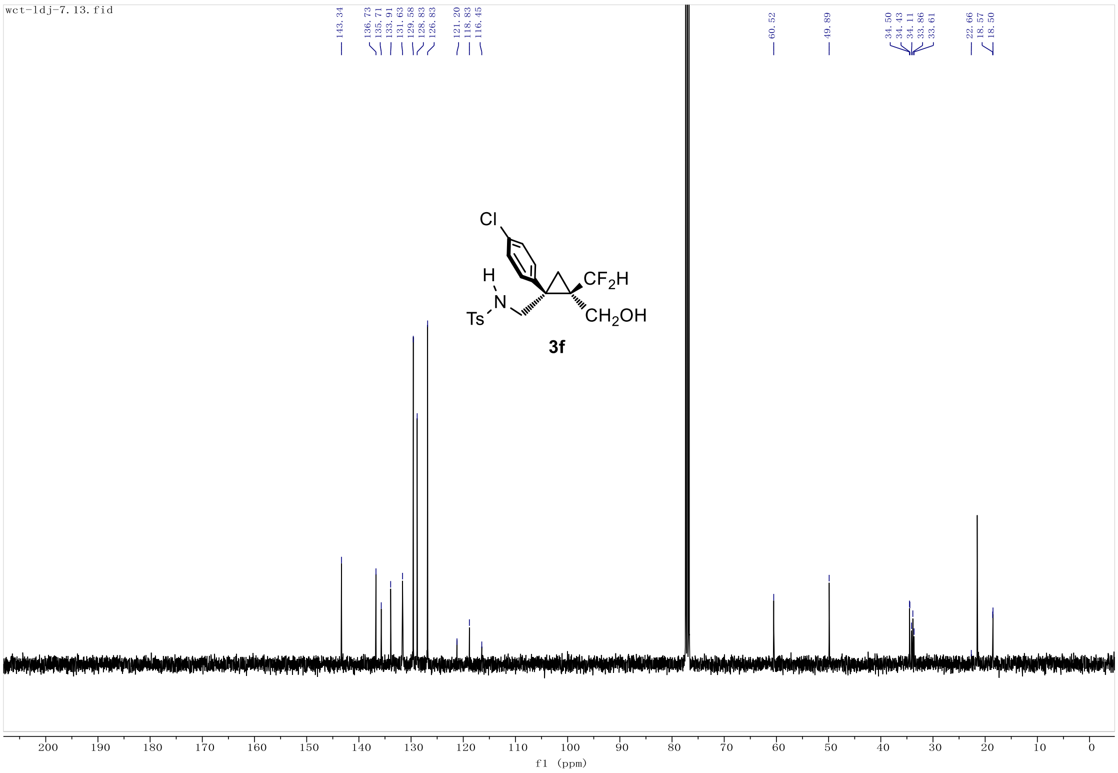
wct-ldj-7.13.fid
143.34
136.73
135.71
133.91
131.63
129.58
128.83
126.83
121.20
118.83
116.45
60.52
49.89
34.50
34.43
34.11
33.86
33.61
22.66
18.57
18.50
Cl
H
N
Ts
CF2H
CH2OH
3f
200
190
180
170
160
150
140
130
120
110
100
90
80
70
60
50
40
30
20
10
0
f1 (ppm)

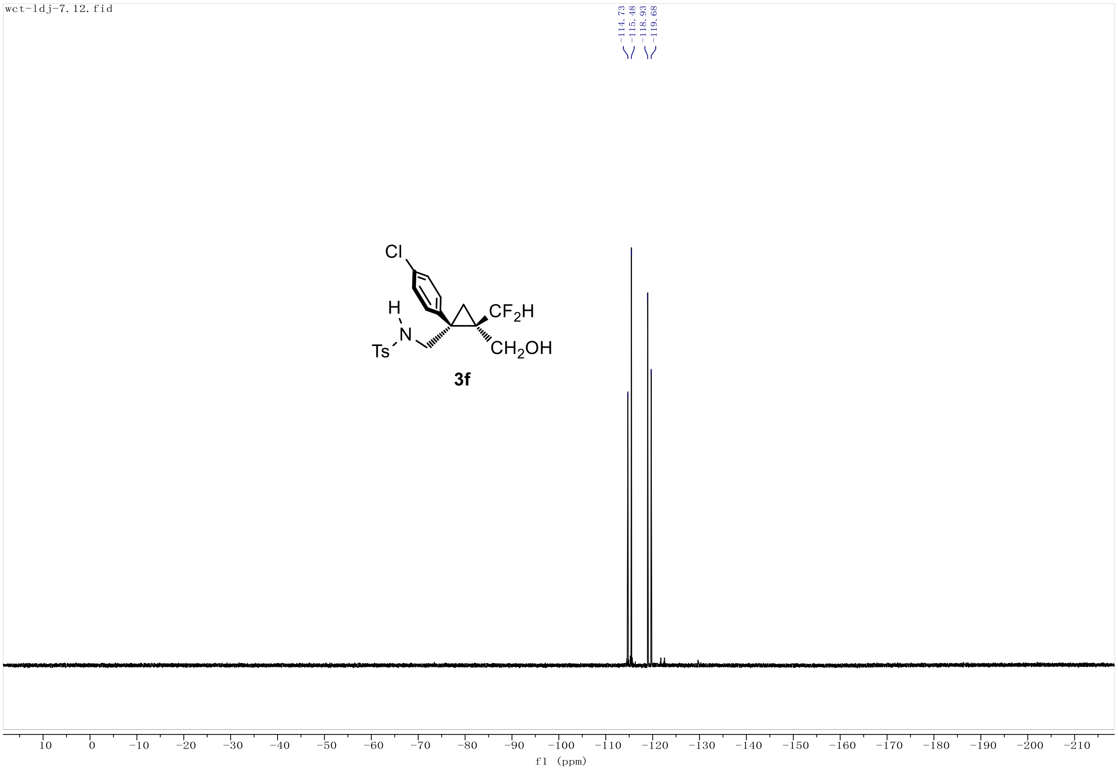
wct-ldj-7.12.fid
-114.73
-115.48
-118.93
-119.68
Cl
H
N
Ts
CF2H
CH2OH
3f
10
0
-10
-20
-30
-40
-50
-60
-70
-80
-90
-100
-110
-120
-130
-140
-150
-160
-170
-180
-190
-200
-210
f1 (ppm)

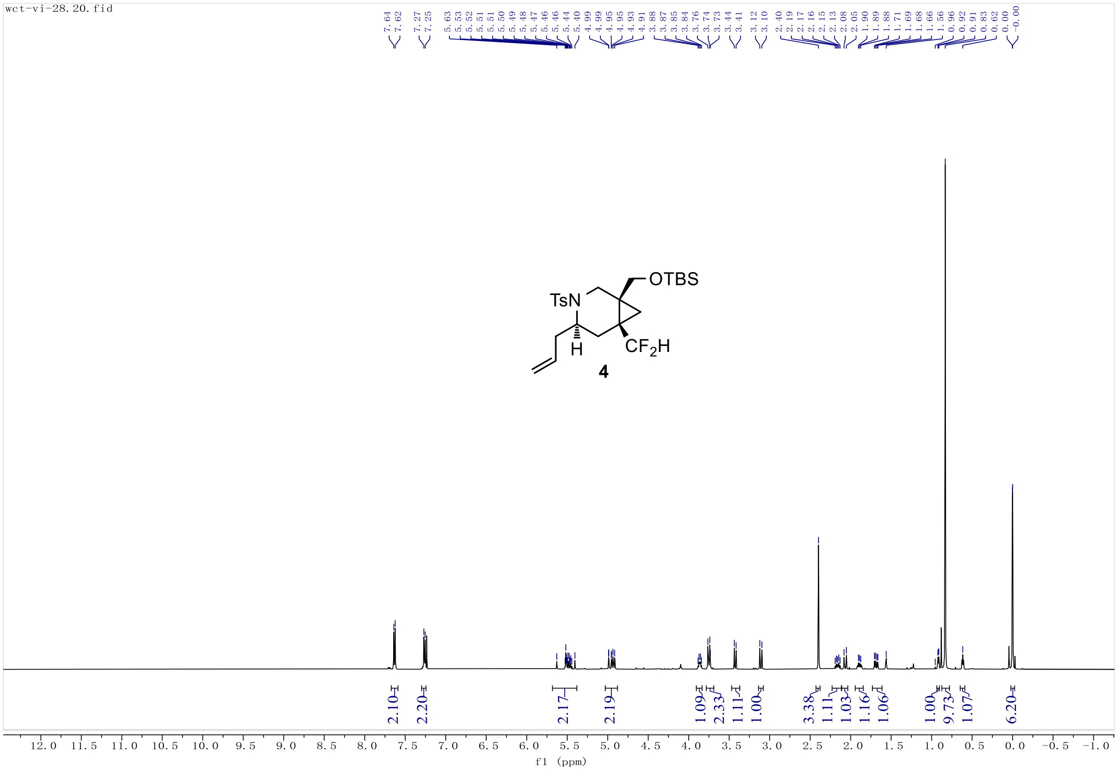
wct-vi-28.20.fid
7.64
7.62
7.27
7.25
5.63
5.53
5.52
5.51
5.51
5.50
5.49
5.48
5.47
5.46
5.46
5.44
5.40
4.99
4.99
4.95
4.95
4.93
4.91
3.88
3.87
3.85
3.84
3.76
3.74
3.73
3.44
3.41
3.12
3.10
2.40
2.19
2.17
2.16
2.15
2.13
2.08
2.05
1.90
1.89
1.88
1.71
1.69
1.68
1.66
1.56
0.96
0.92
0.91
0.83
0.62
0.00
-0.00
TsN
OTBS
H
CF2H
4
2.10
2.20
2.17
2.19
1.09
2.33
1.11
1.00
3.38
1.11
1.03
1.16
1.06
1.00
9.73
1.07
6.20
f1 (ppm)

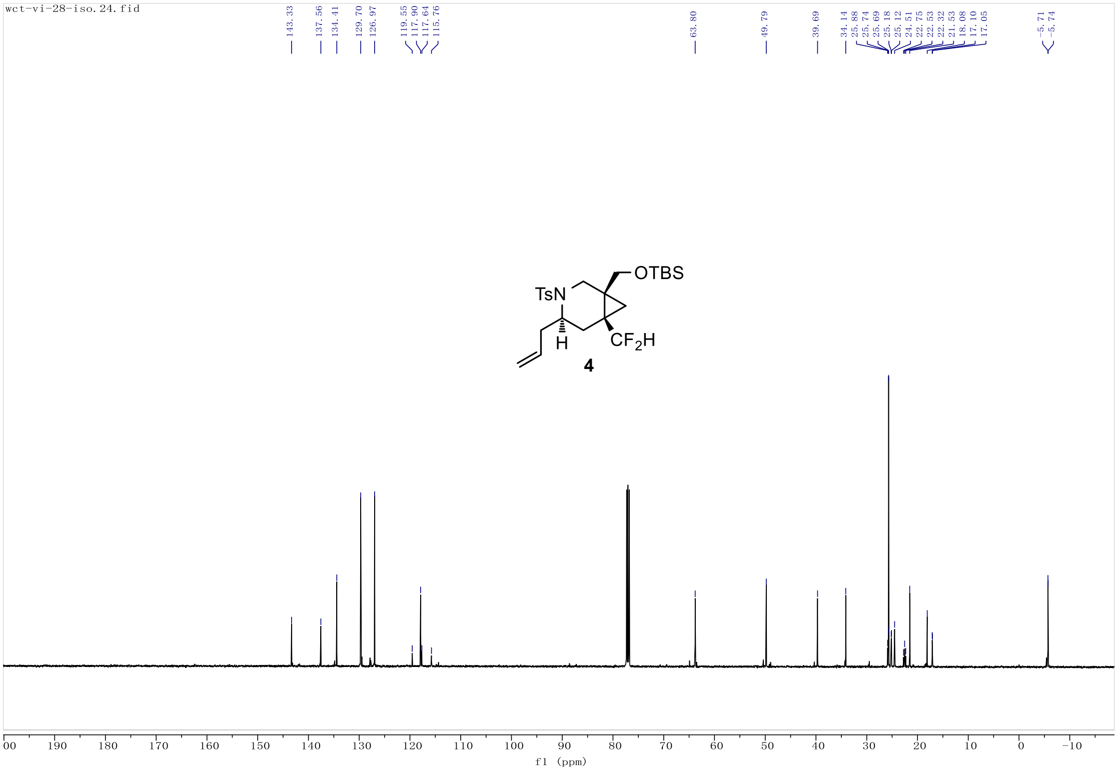
wct-vi-28-iso.24.fid
143.33
137.56
134.41
129.70
126.97
119.55
117.90
117.64
115.76
63.80
49.79
39.69
34.14
25.88
25.74
25.69
25.18
25.12
24.51
22.75
22.53
22.32
21.53
18.08
17.10
17.05
-5.71
-5.74
TsN
OTBS
H
CF2H
4
f1 (ppm)

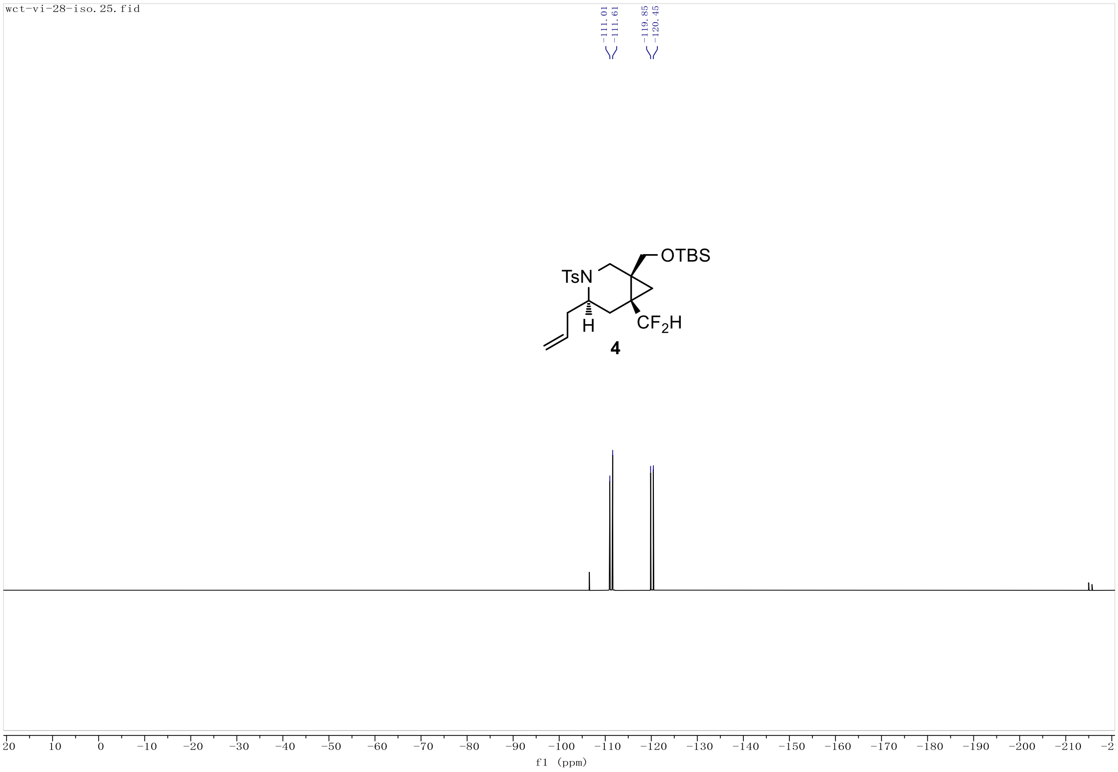
wct-vi-28-iso.25.fid
-111.01
-111.61
-119.85
-120.45
OTBS
TsN
H
CF2H
4
20 10 0 -10 -20 -30 -40 -50 -60 -70 -80 -90 -100 -110 -120 -130 -140 -150 -160 -170 -180 -190 -200 -210
f1 (ppm)

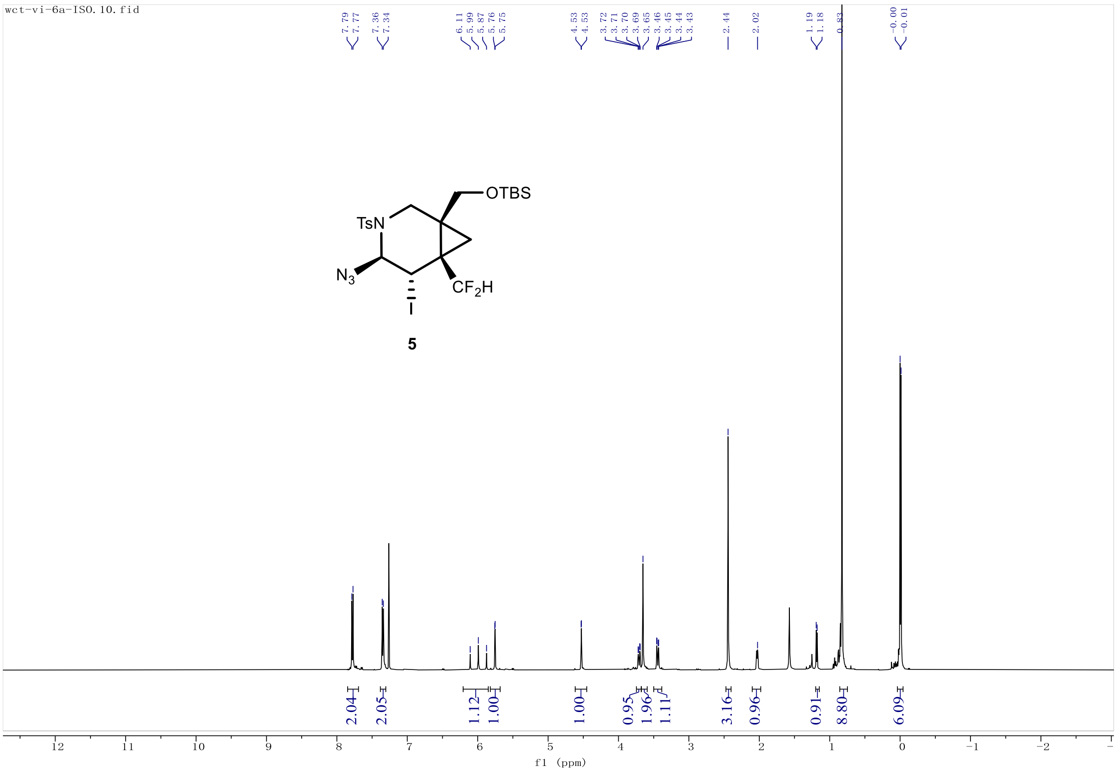
wct-vi-6a-ISO.10.fid
7.79
7.77
7.36
7.34
6.11
5.99
5.87
5.76
5.75
4.53
4.53
3.72
3.71
3.70
3.69
3.65
3.46
3.45
3.44
3.43
2.44
2.02
1.19
1.18
0.83
-0.00
-0.01
OTBS
TsN
N3
I
CF2H
5
2.04
2.05
1.12
1.00
1.00
0.95
1.96
1.11
3.16
0.96
0.91
8.80
6.09
12 11 10 9 8 7 6 5 4 3 2 1 0 -1 -2
f1 (ppm)

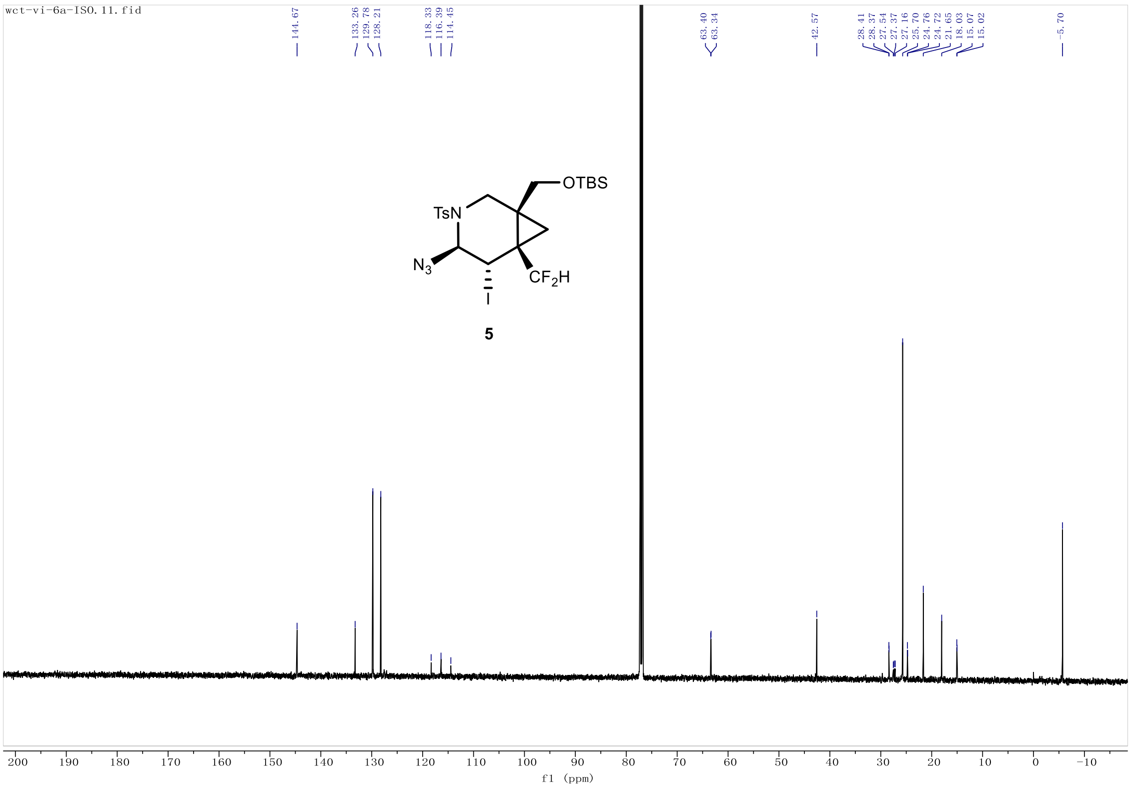
wct-vi-6a-ISO.11.fid
144.67
133.26
129.78
128.21
118.33
116.39
114.45
63.40
63.34
42.57
28.41
28.37
27.54
27.37
27.16
25.70
24.76
24.72
21.65
18.03
15.07
15.02
-5.70
TsN
OTBS
N3
CF2H
I
5
f1 (ppm)

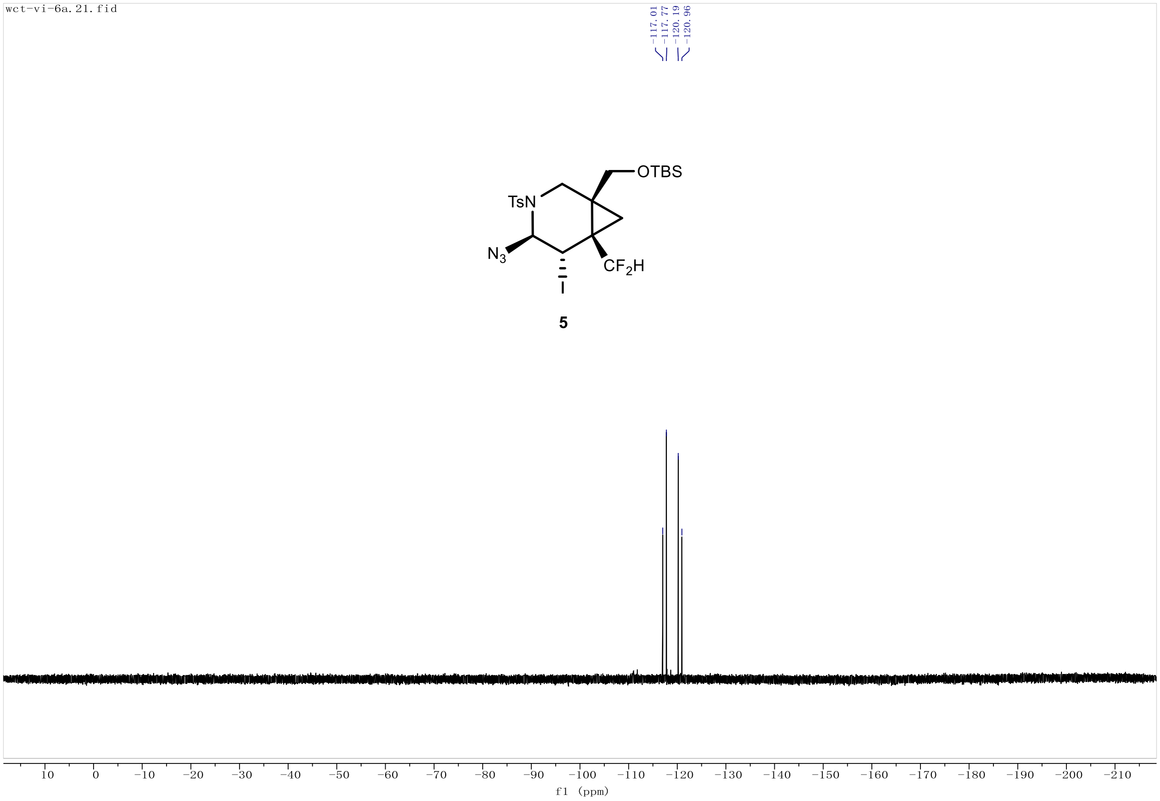
wct-vi-6a.21.fid
-117.01
-117.77
-120.19
-120.96
TsN
OTBS
N3
CF2H
I
5
f1 (ppm)

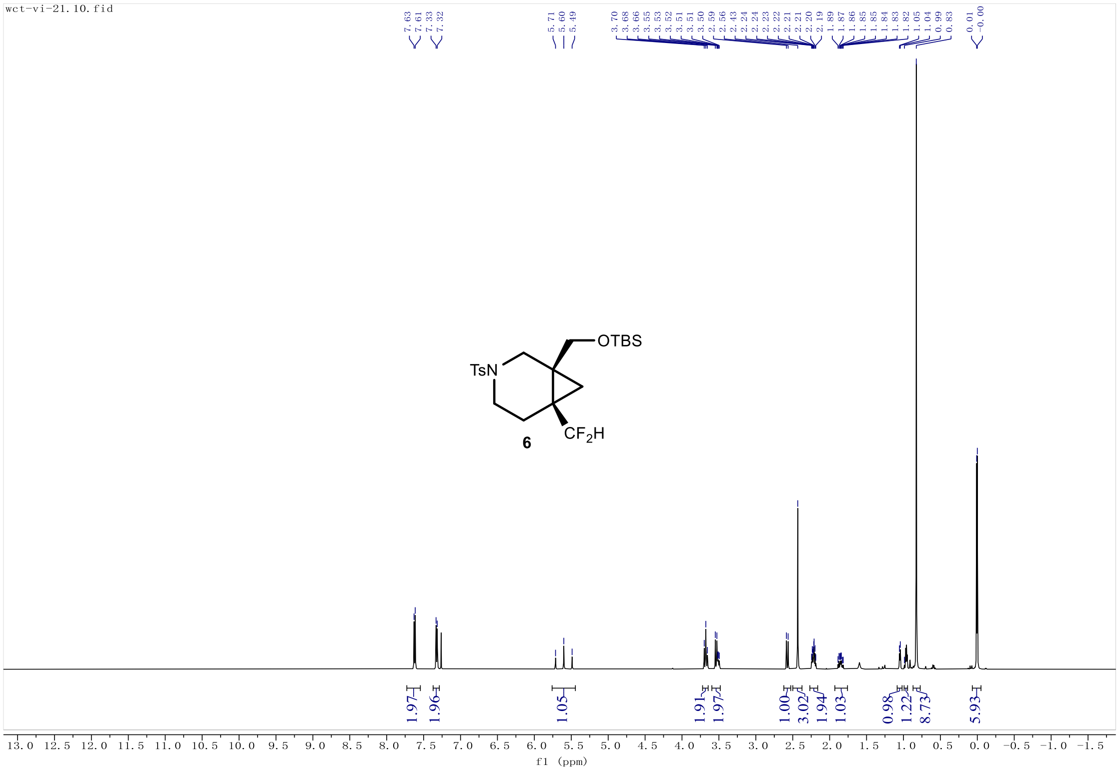
wct-vi-21.10.fid
7.63 7.61 7.33 7.32
5.71 5.60 5.49
3.70 3.68 3.66 3.55 3.53 3.52 3.51 3.51 3.50 2.59 2.56 2.43 2.24 2.24 2.23 2.22 2.21 2.21 2.20 2.19 1.89 1.87 1.86 1.85 1.85 1.84 1.83 1.82 1.05 1.04 0.99 0.83
0.01 -0.00
TsN
OTBS
CF_2H
6
1.97 1.96 1.05 1.91 1.97 1.00 3.02 1.94 1.03 0.98 1.22 8.73 5.93
f1 (ppm)
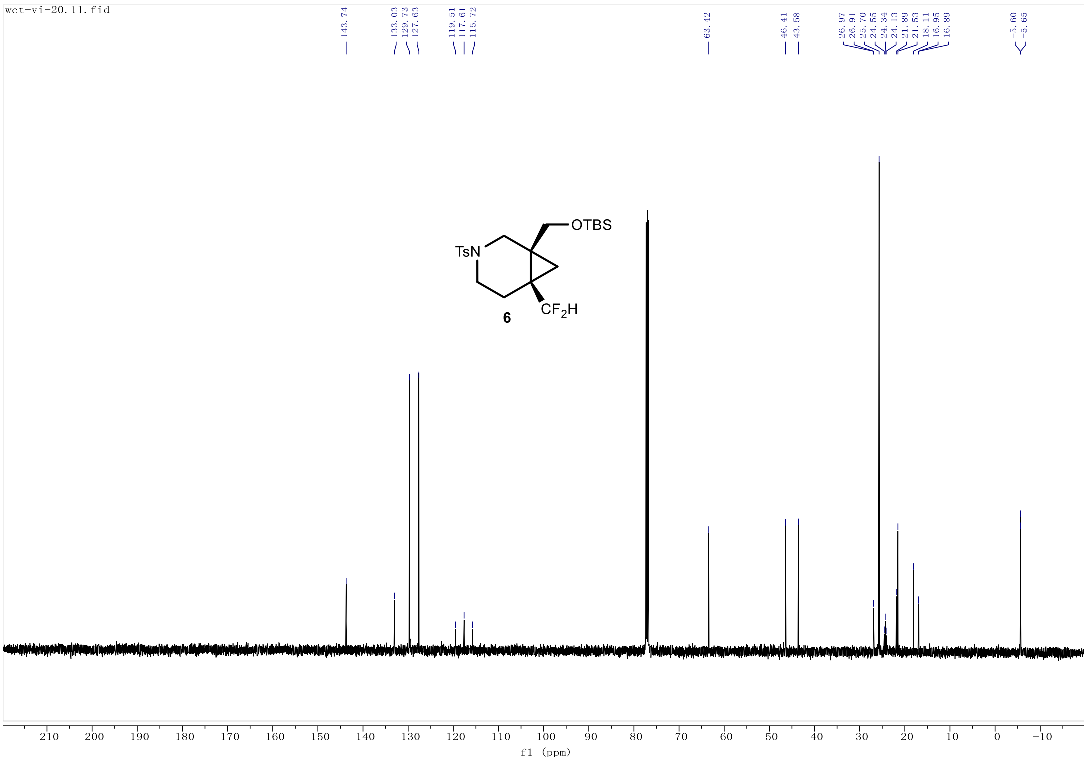
wct-vi-20.11.fid
143.74
133.03 129.73 127.63
119.51 117.61 115.72
63.42
46.41 43.58
26.97 26.91 25.70 24.55 24.34 24.13 21.89 21.53 18.11 16.95 16.89
-5.60 -5.65
TsN
OTBS
CF_2H
6
f1 (ppm)

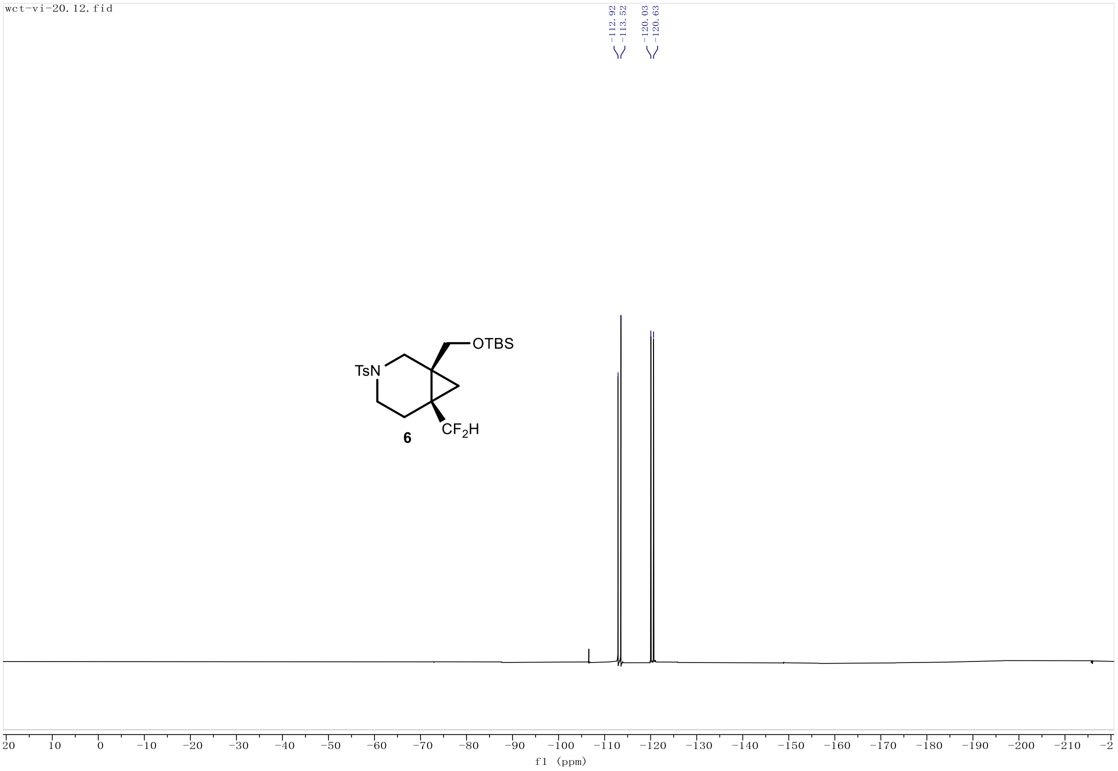

9. References

[1] a) R. Wu, K. Chen, J. Ma, Z.-X. Yu, S. Zhu, *Sci. China Chem.* **2020**, *63*, 1230-1239; b) C. Wang, R. Wu, K. Chen, S. Zhu, *Angew. Chem. Int. Ed.* **2023**, 62, e202305864.

[2] W. Huang, Q. Shen, J. Wang, X. Zhou, *J. Org. Chem.* **2008**, *73*, 1586-1589.

[3] S.-i. Ikeda, H. Miyashita, M. Taniguchi, H. Kondo, M. Okano, Y. Sato, K. Odashima, *J. Am. Chem. Soc.* **2002**, *124*, 12060-12061.

[4] T. Kitamura, Y. Sato, M. Mori, *Adv. Synth. Catal.* **2002**, *344*, 678-693.

[5] T. Nishimura, Y. Maeda, T. Hayashi, *Org. Lett.* **2011**, *13*, 3674-3677.
